# Supplementary material for: Intrinsic circadian organization of the spinal cord and dorsal root ganglia
Source: Pain Rep. 2026 Oct 8;11(6):e1505. doi: 10.1097/PR9.0000000000001505 (PMC13652437; doi:10.1097/PR9.0000000000001505)
Supplement: Supplementary file 1 [file painreports-11-e1505-s001.pdf]

GO:0008631	intrinsic apoptotic signaling pathway in response to oxidative stress	2/138	53/14464	0.03773585	3.9551545	2.11532611	0.09091715	0.63095922	0.6196921	Bag5/P4hb	2
GO:0048002	antigen processing and presentation of peptide antigen	2/138	53/14464	0.03773585	3.9551545	2.11532611	0.09091715	0.63095922	0.6196921	Calr/Pdia3	2
GO:0000301	retrograde transport, vesicle recycling within Golgi	1/138	10/14464	0.1	10.4811594	2.94356538	0.09144271	0.63095922	0.6196921	Cog6	1
GO:0002713	negative regulation of B cell mediated immunity	1/138	10/14464	0.1	10.4811594	2.94356538	0.09144271	0.63095922	0.6196921	Cr1l	1
GO:0002836	positive regulation of response to tumor cell	1/138	10/14464	0.1	10.4811594	2.94356538	0.09144271	0.63095922	0.6196921	Hspd1	1
GO:0002839	positive regulation of immune response to tumor cell	1/138	10/14464	0.1	10.4811594	2.94356538	0.09144271	0.63095922	0.6196921	Hspd1	1
GO:0002890	negative regulation of immunoglobulin mediated immune response	1/138	10/14464	0.1	10.4811594	2.94356538	0.09144271	0.63095922	0.6196921	Cr1l	1
GO:0006517	protein deglycosylation	1/138	10/14464	0.1	10.4811594	2.94356538	0.09144271	0.63095922	0.6196921	Fktn	1
GO:0006534	cysteine metabolic process	1/138	10/14464	0.1	10.4811594	2.94356538	0.09144271	0.63095922	0.6196921	Gclm	1
GO:0006551	L-leucine metabolic process	1/138	10/14464	0.1	10.4811594	2.94356538	0.09144271	0.63095922	0.6196921	Hmgcll1	1
GO:0007440	foregut morphogenesis	1/138	10/14464	0.1	10.4811594	2.94356538	0.09144271	0.63095922	0.6196921	Smad2	1
GO:0009086	methionine biosynthetic process	1/138	10/14464	0.1	10.4811594	2.94356538	0.09144271	0.63095922	0.6196921	Enoph1	1
GO:0009221	pyrimidine deoxyribonucleotide biosynthetic process	1/138	10/14464	0.1	10.4811594	2.94356538	0.09144271	0.63095922	0.6196921	Tbpl1	1
GO:0010819	regulation of T cell chemotaxis	1/138	10/14464	0.1	10.4811594	2.94356538	0.09144271	0.63095922	0.6196921	Oxsr1	1
GO:0019370	leukotriene biosynthetic process	1/138	10/14464	0.1	10.4811594	2.94356538	0.09144271	0.63095922	0.6196921	Lta4h	1
GO:0019511	peptidyl-proline hydroxylation	1/138	10/14464	0.1	10.4811594	2.94356538	0.09144271	0.63095922	0.6196921	P4hb	1
GO:0021979	hypothalamus cell differentiation	1/138	10/14464	0.1	10.4811594	2.94356538	0.09144271	0.63095922	0.6196921	Sema3e	1
GO:0035338	long-chain fatty-acyl-CoA biosynthetic process	1/138	10/14464	0.1	10.4811594	2.94356538	0.09144271	0.63095922	0.6196921	Elovl7	1
GO:0035646	endosome to melanosome transport	1/138	10/14464	0.1	10.4811594	2.94356538	0.09144271	0.63095922	0.6196921	Rab38	1
GO:0043485	endosome to pigment granule transport	1/138	10/14464	0.1	10.4811594	2.94356538	0.09144271	0.63095922	0.6196921	Rab38	1
GO:0045654	positive regulation of megakaryocyte differentiation	1/138	10/14464	0.1	10.4811594	2.94356538	0.09144271	0.63095922	0.6196921	Scin	1
GO:0045916	negative regulation of complement activation	1/138	10/14464	0.1	10.4811594	2.94356538	0.09144271	0.63095922	0.6196921	Cr1l	1
GO:0048757	pigment granule maturation	1/138	10/14464	0.1	10.4811594	2.94356538	0.09144271	0.63095922	0.6196921	Rab38	1
GO:0051775	response to redox state	1/138	10/14464	0.1	10.4811594	2.94356538	0.09144271	0.63095922	0.6196921	Bmal1	1
GO:0060049	regulation of protein glycosylation	1/138	10/14464	0.1	10.4811594	2.94356538	0.09144271	0.63095922	0.6196921	Fktn	1
GO:0060509	type I pneumocyte differentiation	1/138	10/14464	0.1	10.4811594	2.94356538	0.09144271	0.63095922	0.6196921	Lta4h	1
GO:0071073	positive regulation of phospholipid biosynthetic process	1/138	10/14464	0.1	10.4811594	2.94356538	0.09144271	0.63095922	0.6196921	Rab38	1
GO:0097067	cellular response to thyroid hormone stimulus	1/138	10/14464	0.1	10.4811594	2.94356538	0.09144271	0.63095922	0.6196921	Gclm	1
GO:0097499	protein localization to non-motile cilium	1/138	10/14464	0.1	10.4811594	2.94356538	0.09144271	0.63095922	0.6196921	Ift80	1
GO:0098700	neurotransmitter loading into synaptic vesicle	1/138	10/14464	0.1	10.4811594	2.94356538	0.09144271	0.63095922	0.6196921	Slc17a8	1
GO:0150172	regulation of phosphatidylcholine metabolic process	1/138	10/14464	0.1	10.4811594	2.94356538	0.09144271	0.63095922	0.6196921	Rab38	1
GO:2000562	negative regulation of CD4-positive, alpha-beta T cell proliferation	1/138	10/14464	0.1	10.4811594	2.94356538	0.09144271	0.63095922	0.6196921	Itch	1
GO:0006612	protein targeting to membrane	3/138	112/14464	0.02678571	2.80745342	1.88463032	0.09174265	0.63095922	0.6196921	Hspa5/Ubl4a/Srprb	3
GO:1905477	positive regulation of protein localization to membrane	3/138	113/14464	0.02654867	2.7826087	1.86706924	0.09361807	0.63095922	0.6196921	Slc5a3/Arhgef16/Cnpy4	3
GO:0034332	adherens junction organization	2/138	54/14464	0.03703704	3.8819109	2.08234029	0.09384516	0.63095922	0.6196921	Pkp2/Cdh10	2
GO:0035567	non-canonical Wnt signaling pathway	2/138	54/14464	0.03703704	3.8819109	2.08234029	0.09384516	0.63095922	0.6196921	Fzd8/Ift80	2
GO:0043506	regulation of JUN kinase activity	2/138	54/14464	0.03703704	3.8819109	2.08234029	0.09384516	0.63095922	0.6196921	Fzd8/Sash1	2
GO:0048524	positive regulation of viral process	2/138	54/14464	0.03703704	3.8819109	2.08234029	0.09384516	0.63095922	0.6196921	Srpk1/P4hb	2
GO:1901224	positive regulation of non-canonical NF-kappaB signal transduction	2/138	54/14464	0.03703704	3.8819109	2.08234029	0.09384516	0.63095922	0.6196921	Calr/Sash1	2
GO:0008637	apoptotic mitochondrial changes	3/138	114/14464	0.02631579	2.75819985	1.84969863	0.09550931	0.63095922	0.6196921	Hspd1/Gclm/Ppp2cb	3
GO:1903076	regulation of protein localization to plasma membrane	3/138	114/14464	0.02631579	2.75819985	1.84969863	0.09550931	0.63095922	0.6196921	Mmp14/Arhgef16/Cnpy4	3
GO:0009895	negative regulation of catabolic process	6/138	330/14464	0.01818182	1.90566535	1.63342215	0.09626365	0.63095922	0.6196921	Mettl16/Hnrnpc/Pkp3/Bag5/Mettl14/Sec22b	6
GO:0071805	potassium ion transmembrane transport	4/138	182/14464	0.02197802	2.30355152	1.73690331	0.09654382	0.63095922	0.6196921	Kcnn1/Kcnk13/Kcnip2/Oxsr1	4
GO:0010812	negative regulation of cell-substrate adhesion	2/138	55/14464	0.03636364	3.8113307	2.05013576	0.09680026	0.63095922	0.6196921	Mmp14/Sema3e	2
GO:0031589	cell-substrate adhesion	6/138	332/14464	0.01807229	1.89418544	1.61771154	0.09836867	0.63095922	0.6196921	Calr/Mmp14/Has2/Pkp2/Sema3e/P4hb	6
GO:0034763	negative regulation of transmembrane transport	3/138	116/14464	0.02586207	2.71064468	1.81551309	0.09933862	0.63095922	0.6196921	Rrad/Sri/Oxsr1	3
GO:0043161	proteasome-mediated ubiquitin-dependent protein catabolic process	7/138	412/14464	0.01699029	1.78077951	1.5780262	0.09957379	0.63095922	0.6196921	Bmal1/Dtx4/Klhl5/Ppp2cb/Bag5/Itch/Cul2	7
GO:1903409	reactive oxygen species biosynthetic process	2/138	56/14464	0.03571429	3.74327122	2.01867858	0.09978167	0.63095922	0.6196921	Slc5a3/Hspd1	2
GO:0000244	spliceosomal tri-snRNP complex assembly	1/138	11/14464	0.09090909	9.52832675	2.77707389	0.10011719	0.63095922	0.6196921	Prpf31	1
GO:0002407	dendritic cell chemotaxis	1/138	11/14464	0.09090909	9.52832675	2.77707389	0.10011719	0.63095922	0.6196921	Calr	1
GO:0002424	T cell mediated immune response to tumor cell	1/138	11/14464	0.09090909	9.52832675	2.77707389	0.10011719	0.63095922	0.6196921	Hspd1	1
GO:0002755	MyD88-dependent toll-like receptor signaling pathway	1/138	11/14464	0.09090909	9.52832675	2.77707389	0.10011719	0.63095922	0.6196921	Hspd1	1
GO:0002840	regulation of T cell mediated immune response to tumor cell	1/138	11/14464	0.09090909	9.52832675	2.77707389	0.10011719	0.63095922	0.6196921	Hspd1	1
GO:0003096	renal sodium ion transport	1/138	11/14464	0.09090909	9.52832675	2.77707389	0.10011719	0.63095922	0.6196921	Oxsr1	1
GO:0006555	methionine metabolic process	1/138	11/14464	0.09090909	9.52832675	2.77707389	0.10011719	0.63095922	0.6196921	Enoph1	1
GO:0006596	polyamine biosynthetic process	1/138	11/14464	0.09090909	9.52832675	2.77707389	0.10011719	0.63095922	0.6196921	Sat1	1
GO:0009950	dorsal/ventral axis specification	1/138	11/14464	0.09090909	9.52832675	2.77707389	0.10011719	0.63095922	0.6196921	Smad2	1
GO:0035630	bone mineralization involved in bone maturation	1/138	11/14464	0.09090909	9.52832675	2.77707389	0.10011719	0.63095922	0.6196921	Ift80	1
GO:0042402	cellular biogenic amine catabolic process	1/138	11/14464	0.09090909	9.52832675	2.77707389	0.10011719	0.63095922	0.6196921	Sat1	1
GO:0042989	sequestering of actin monomers	1/138	11/14464	0.09090909	9.52832675	2.77707389	0.10011719	0.63095922	0.6196921	Scin	1
GO:0048340	paraxial mesoderm morphogenesis	1/138	11/14464	0.09090909	9.52832675	2.77707389	0.10011719	0.63095922	0.6196921	Smad2	1
GO:0050655	dermatan sulfate proteoglycan metabolic process	1/138	11/14464	0.09090909	9.52832675	2.77707389	0.10011719	0.63095922	0.6196921	Hexa	1
GO:0061299	retina vasculature morphogenesis in camera-type eye	1/138	11/14464	0.09090909	9.52832675	2.77707389	0.10011719	0.63095922	0.6196921	Rhoj	1
GO:0070986	left/right axis specification	1/138	11/14464	0.09090909	9.52832675	2.77707389	0.10011719	0.63095922	0.6196921	Ift172	1
GO:0071257	cellular response to electrical stimulus	1/138	11/14464	0.09090909	9.52832675	2.77707389	0.10011719	0.63095922	0.6196921	Calr	1
GO:0090085	regulation of protein deubiquitination	1/138	11/14464	0.09090909	9.52832675	2.77707389	0.10011719	0.63095922	0.6196921	Itch	1
GO:1903441	protein localization to ciliary membrane	1/138	11/14464	0.09090909	9.52832675	2.77707389	0.10011719	0.63095922	0.6196921	Ift80	1
GO:1903727	positive regulation of phospholipid metabolic process	1/138	11/14464	0.09090909	9.52832675	2.77707389	0.10011719	0.63095922	0.6196921	Rab38	1
GO:0033363	secretory granule organization	2/138	57/14464	0.03508772	3.6775998	1.98793683	0.10278864	0.63095922	0.6196921	Rab38/Tbpl1	2
GO:0000045	autophagosome assembly	3/138	118/14464	0.02542373	2.66470155	1.78204324	0.10322922	0.63095922	0.6196921	Snx18/Wdr45b/Sec22b	3
GO:0043271	negative regulation of monoatomic ion transport	3/138	118/14464	0.02542373	2.66470155	1.78204324	0.10322922	0.63095922	0.6196921	Rrad/Sri/Oxsr1	3
GO:0006888	endoplasmic reticulum to Golgi vesicle-mediated transport	3/138	119/14464	0.02521008	2.6423091	1.76556766	0.10519706	0.63095922	0.6196921	Lman2/P4hb/Sec22b	3
GO:1903313	positive regulation of mRNA metabolic process	3/138	119/14464	0.02521008	2.6423091	1.76556766	0.10519706	0.63095922	0.6196921	Mettl16/Obi1/Mettl14	3
GO:0006023	aminoglycan biosynthetic process	2/138	58/14464	0.03448276	3.6141929	1.95788052	0.10582041	0.63095922	0.6196921	Has2/Hexa	2
GO:0044088	regulation of vacuole organization	2/138	58/14464	0.03448276	3.6141929	1.95788052	0.10582041	0.63095922	0.6196921	Snx18/Sec22b	2
GO:0055117	regulation of cardiac muscle contraction	2/138	58/14464	0.03448276	3.6141929	1.95788052	0.10582041	0.63095922	0.6196921	Pkp2/Sri	2
GO:0086003	cardiac muscle cell contraction	2/138	58/14464	0.03448276	3.6141929	1.95788052	0.10582041	0.63095922	0.6196921	Pkp2/Sri	2
GO:0006983	ER overload response	1/138	12/14464	0.08333333	8.73429952	2.63059443	0.10870944	0.63095922	0.6196921	Hspa5	1
GO:0008611	ether lipid biosynthetic process	1/138	12/14464	0.08333333	8.73429952	2.63059443	0.10870944	0.63095922	0.6196921	Selenoi	1
GO:0009310	amine catabolic process	1/138	12/14464	0.08333333	8.73429952	2.63059443	0.10870944	0.63095922	0.6196921	Sat1	1
GO:0010998	regulation of translational initiation by eIF2 alpha phosphorylation	1/138	12/14464	0.08333333	8.73429952	2.63059443	0.10870944	0.63095922	0.6196921	Dnajc3	1
GO:0015937	coenzyme A biosynthetic process	1/138	12/14464	0.08333333	8.73429952	2.63059443	0.10870944	0.63095922	0.6196921	Ppcs	1
GO:0016137	glycoside metabolic process	1/138	12/14464	0.08333333	8.73429952	2.63059443	0.10870944	0.63095922	0.6196921	Akr1a1	1
GO:0019852	L-ascorbic acid metabolic process	1/138	12/14464	0.08333333	8.73429952	2.63059443	0.10870944	0.63095922	0.6196921	Akr1a1	1
GO:0036490	regulation of translation in response to endoplasmic reticulum stress	1/138	12/14464	0.08333333	8.73429952	2.63059443	0.10870944	0.63095922	0.6196921	Dnajc3	1
GO:0042364	water-soluble vitamin biosynthetic process	1/138	12/14464	0.08333333	8.73429952	2.63059443	0.10870944	0.63095922	0.6196921	Akr1a1	1
GO:0042761	very long-chain fatty acid biosynthetic process	1/138	12/14464	0.08333333	8.73429952	2.63059443	0.10870944	0.63095922	0.6196921	Elovl7	1
GO:0043476	pigment accumulation	1/138	12/14464	0.08333333	8.73429952	2.63059443	0.10870944	0.63095922	0.6196921	Rab38	1
GO:0043482	cellular pigment accumulation	1/138	12/14464	0.08333333	8.73429952	2.63059443	0.10870944	0.63095922	0.6196921	Rab38	1
GO:0046479	glycosphingolipid catabolic process	1/138	12/14464	0.08333333	8.73429952	2.63059443	0.10870944	0.63095922	0.6196921	Hexa	1
GO:0046504	glycerol ether biosynthetic process	1/138	12/14464	0.08333333	8.73429952	2.63059443	0.10870944	0.63095922	0.6196921	Selenoi	1
GO:0046949	fatty-acyl-CoA biosynthetic process	1/138	12/14464	0.08333333	8.73429952	2.63059443	0.10870944	0.63095922	0.6196921	Elovl7	1
GO:0048291	isotype switching to IgG isotypes	1/138	12/14464	0.08333333	8.73429952	2.63059443	0.10870944	0.63095922	0.6196921	Hspd1	1
GO:0048312	intracellular distribution of mitochondria	1/138	12/14464	0.08333333	8.73429952	2.63059443	0.10870944	0.63095922	0.6196921	S2bpcox16	1
GO:0051549	positive regulation of keratinocyte migration	1/138	12/14464	0.08333333	8.73429952	2.63059443	0.10870944	0.63095922	0.6196921	Has2	1
GO:0061723	glycophagy	1/138	12/14464	0.08333333	8.73429952	2.63059443	0.10870944	0.63095922	0.6196921	Wdr45b	1
GO:0071166	ribonucleoprotein complex localization	1/138	12/14464	0.08333333	8.73429952	2.63059443	0.10870944	0.63095922	0.6196921	Prpf31	1
GO:0071476	cellular hypotonic response	1/138	12/14464	0.08333333	8.73429952	2.63059443	0.10870944	0.63095922	0.6196921	Oxsr1	1
GO:0072683	T cell extravasation	1/138	12/14464	0.08333333	8.73429952	2.63059443	0.10870944	0.63095922	0.6196921	Cd99l2	1
GO:0140627	ubiquitin-dependent protein catabolic process via the C-end degron rule pathway	1/138	12/14464	0.08333333	8.73429952	2.63059443	0.10870944	0.63095922	0.6196921	Cul2	1
GO:1901163	regulation of trophoblast cell migration	1/138	12/14464	0.08333333	8.73429952	2.63059443	0.10870944	0.63095922	0.6196921	Calr	1
GO:1902644	tertiary alcohol metabolic process	1/138	12/14464	0.08333333	8.73429952	2.63059443	0.10870944	0.63095922	0.6196921	Akr1a1	1
GO:2000322	regulation of glucocorticoid receptor signaling pathway	1/138	12/14464	0.08333333	8.73429952	2.63059443	0.10870944	0.63095922	0.6196921	Bmal1	1
GO:2000644	regulation of receptor catabolic process	1/138	12/14464	0.08333333	8.73429952	2.63059443	0.10870944	0.63095922	0.6196921	Itch	1
GO:1903078	positive regulation of protein localization to plasma membrane	2/138	59/14464	0.03389831	3.5529354	1.92848143	0.10887625	0.63095922	0.6196921	Arhgef16/Cnpy4	2
GO:0007043	cell-cell junction assembly	3/138	121/14464	0.02479339	2.59863457	1.73311806	0.10917699	0.63095922	0.6196921	Pkp3/Pkp2/Cdh10	3
GO:0043414	macromolecule methylation	3/138	121/14464	0.02479339	2.59863457	1.73311806	0.10917699	0.63095922	0.6196921	Mettl16/Tfb1m/Mettl14	3
GO:0046467	membrane lipid biosynthetic process	3/138	121/14464	0.02479339	2.59863457	1.73311806	0.10917699	0.63095922	0.6196921	Pigg/Degs1/Elovl7	3
GO:0045785	positive regulation of cell adhesion	7/138	422/14464	0.01658768	1.73858095	1.51128407	0.10932157	0.63095922	0.6196921	Card11/Calr/Has2/Hspd1/Pkp3/Pkp2/P4hb	7
GO:0002685	regulation of leukocyte migration	4/138	191/14464	0.02094241	2.19500721	1.63168319	0.1101076	0.63095922	0.6196921	Calr/Mmp14/Cd99l2/Oxsr1	4
GO:0060485	mesenchyme development	5/138	267/14464	0.01872659	1.96276394	1.55841606	0.11241274	0.63095922	0.6196921	Has2/Dand5/Smad2/Sema3e/Spred3	5
GO:0034308	primary alcohol metabolic process	2/138	61/14464	0.03278689	3.43644571	1.87155002	0.11505723	0.63095922	0.6196921	Dhrs3/Akr1a1	2
GO:0060350	endochondral bone morphogenesis	2/138	61/14464	0.03278689	3.43644571	1.87155002	0.11505723	0.63095922	0.6196921	Mmp14/Ift80	2
GO:0000097	sulfur amino acid biosynthetic process	1/138	13/14464	0.07692308	8.06243032	2.50024844	0.11722025	0.63095922	0.6196921	Enoph1	1
GO:0002643	regulation of tolerance induction	1/138	13/14464	0.07692308	8.06243032	2.50024844	0.11722025	0.63095922	0.6196921	Itch	1
GO:0002921	negative regulation of humoral immune response	1/138	13/14464	0.07692308	8.06243032	2.50024844	0.11722025	0.63095922	0.6196921	Cr1l	1
GO:0002923	regulation of humoral immune response mediated by circulating immunoglobulin	1/138	13/14464	0.07692308	8.06243032	2.50024844	0.11722025	0.63095922	0.6196921	Cr1l	1
GO:0003091	renal water homeostasis	1/138	13/14464	0.07692308	8.06243032	2.50024844	0.11722025	0.63095922	0.6196921	Has2	1
GO:0003413	chondrocyte differentiation involved in endochondral bone morphogenesis	1/138	13/14464	0.07692308	8.06243032	2.50024844	0.11722025	0.63095922	0.6196921	Ift80	1
GO:0006750	glutathione biosynthetic process	1/138	13/14464	0.07692308	8.06243032	2.50024844	0.11722025	0.63095922	0.6196921	Gclm	1
GO:0006833	water transport	1/138	13/14464	0.07692308	8.06243032	2.50024844	0.11722025	0.63095922	0.6196921	Has2	1
GO:0009078	pyruvate family amino acid metabolic process	1/138	13/14464	0.07692308	8.06243032	2.50024844	0.11722025	0.63095922	0.6196921	Hmgcll1	1
GO:0010459	negative regulation of heart rate	1/138	13/14464	0.07692308	8.06243032	2.50024844	0.11722025	0.63095922	0.6196921	Sri	1
GO:0016264	gap junction assembly	1/138	13/14464	0.07692308	8.06243032	2.50024844	0.11722025	0.63095922	0.6196921	Pkp2	1

GO:0019377	glycolipid catabolic process	1/138	13/14464	0.07692308	8.06243032	2.50024844	0.11722025	0.63095922	0.6196921	Hexa	1
GO:0030214	hyaluronan catabolic process	1/138	13/14464	0.07692308	8.06243032	2.50024844	0.11722025	0.63095922	0.6196921	Hexa	1
GO:0035437	maintenance of protein localization in endoplasmic reticulum	1/138	13/14464	0.07692308	8.06243032	2.50024844	0.11722025	0.63095922	0.6196921	Hspa5	1
GO:0046185	aldehyde catabolic process	1/138	13/14464	0.07692308	8.06243032	2.50024844	0.11722025	0.63095922	0.6196921	Akr1a1	1
GO:0046642	negative regulation of alpha-beta T cell proliferation	1/138	13/14464	0.07692308	8.06243032	2.50024844	0.11722025	0.63095922	0.6196921	Itch	1
GO:0051127	positive regulation of actin nucleation	1/138	13/14464	0.07692308	8.06243032	2.50024844	0.11722025	0.63095922	0.6196921	Scin	1
GO:0051280	negative regulation of release of sequestered calcium ion into cytosol	1/138	13/14464	0.07692308	8.06243032	2.50024844	0.11722025	0.63095922	0.6196921	Sri	1
GO:0051547	regulation of keratinocyte migration	1/138	13/14464	0.07692308	8.06243032	2.50024844	0.11722025	0.63095922	0.6196921	Has2	1
GO:0055062	phosphate ion homeostasis	1/138	13/14464	0.07692308	8.06243032	2.50024844	0.11722025	0.63095922	0.6196921	Slc17a8	1
GO:0061450	trophoblast cell migration	1/138	13/14464	0.07692308	8.06243032	2.50024844	0.11722025	0.63095922	0.6196921	Calr	1
GO:0071372	cellular response to follicle-stimulating hormone stimulus	1/138	13/14464	0.07692308	8.06243032	2.50024844	0.11722025	0.63095922	0.6196921	Gclm	1
GO:0072672	neutrophil extravasation	1/138	13/14464	0.07692308	8.06243032	2.50024844	0.11722025	0.63095922	0.6196921	Cd99l2	1
GO:0097384	cellular lipid biosynthetic process	1/138	13/14464	0.07692308	8.06243032	2.50024844	0.11722025	0.63095922	0.6196921	Selenoi	1
GO:0140131	positive regulation of lymphocyte chemotaxis	1/138	13/14464	0.07692308	8.06243032	2.50024844	0.11722025	0.63095922	0.6196921	Oxsr1	1
GO:1900044	regulation of protein K63-linked ubiquitination	1/138	13/14464	0.07692308	8.06243032	2.50024844	0.11722025	0.63095922	0.6196921	Sash1	1
GO:1901386	negative regulation of voltage-gated calcium channel activity	1/138	13/14464	0.07692308	8.06243032	2.50024844	0.11722025	0.63095922	0.6196921	Rrad	1
GO:1901503	ether biosynthetic process	1/138	13/14464	0.07692308	8.06243032	2.50024844	0.11722025	0.63095922	0.6196921	Selenoi	1
GO:1901533	negative regulation of hematopoietic progenitor cell differentiation	1/138	13/14464	0.07692308	8.06243032	2.50024844	0.11722025	0.63095922	0.6196921	Mettl14	1
GO:1902902	negative regulation of autophagosome assembly	1/138	13/14464	0.07692308	8.06243032	2.50024844	0.11722025	0.63095922	0.6196921	Sec22b	1
GO:1902950	regulation of dendritic spine maintenance	1/138	13/14464	0.07692308	8.06243032	2.50024844	0.11722025	0.63095922	0.6196921	Zfp804a	1
GO:0030336	negative regulation of cell migration	5/138	271/14464	0.01845018	1.93379325	1.52301625	0.11769657	0.63095922	0.6196921	Calr/Stard13/S2bpcox16/Pkp2/Brms1	5
GO:0045017	glycerolipid biosynthetic process	4/138	196/14464	0.02040816	2.13901213	1.57572743	0.11799377	0.63095922	0.6196921	Rab38/Dgkk/Pigg/Selenoi	4
GO:0006611	protein export from nucleus	2/138	62/14464	0.03225806	3.38101917	1.84396892	0.11818095	0.63095922	0.6196921	Calr/Frat2	2
GO:0021695	cerebellar cortex development	2/138	62/14464	0.03225806	3.38101917	1.84396892	0.11818095	0.63095922	0.6196921	Hspa5/Fktn	2
GO:0031016	pancreas development	2/138	62/14464	0.03225806	3.38101917	1.84396892	0.11818095	0.63095922	0.6196921	Bmal1/Smad2	2
GO:2000514	regulation of CD4-positive, alpha-beta T cell activation	2/138	62/14464	0.03225806	3.38101917	1.84396892	0.11818095	0.63095922	0.6196921	Card11/Itch	2
GO:0010811	positive regulation of cell-substrate adhesion	3/138	126/14464	0.02380952	2.49551415	1.65477051	0.11937665	0.63095922	0.6196921	Calr/Has2/P4hb	3
GO:1905037	autophagosome organization	3/138	126/14464	0.02380952	2.49551415	1.65477051	0.11937665	0.63095922	0.6196921	Snx18/Wdr45b/Sec22b	3
GO:0050821	protein stabilization	4/138	197/14464	0.02030457	2.1281542	1.56473756	0.11960005	0.63095922	0.6196921	Calr/Hspd1/Bag5/Ift80	4
GO:1901019	regulation of calcium ion transmembrane transporter activity	2/138	63/14464	0.03174603	3.3273522	1.81694726	0.12132589	0.63095922	0.6196921	Rrad/Sri	2
GO:0009953	dorsal/ventral pattern formation	2/138	64/14464	0.03125	3.27536232	1.79046389	0.12449139	0.63095922	0.6196921	Smad2/Ift172	2
GO:0006086	acetyl-CoA biosynthetic process from pyruvate	1/138	14/14464	0.07142857	7.48654244	2.38314036	0.12565036	0.63095922	0.6196921	Pdk3	1
GO:0006971	hypotonic response	1/138	14/14464	0.07142857	7.48654244	2.38314036	0.12565036	0.63095922	0.6196921	Oxsr1	1
GO:0010818	T cell chemotaxis	1/138	14/14464	0.07142857	7.48654244	2.38314036	0.12565036	0.63095922	0.6196921	Oxsr1	1
GO:0010831	positive regulation of myotube differentiation	1/138	14/14464	0.07142857	7.48654244	2.38314036	0.12565036	0.63095922	0.6196921	Mmp14	1
GO:0015721	bile acid and bile salt transport	1/138	14/14464	0.07142857	7.48654244	2.38314036	0.12565036	0.63095922	0.6196921	Slc51a	1
GO:0030213	hyaluronan biosynthetic process	1/138	14/14464	0.07142857	7.48654244	2.38314036	0.12565036	0.63095922	0.6196921	Has2	1
GO:0030242	autophagy of peroxisome	1/138	14/14464	0.07142857	7.48654244	2.38314036	0.12565036	0.63095922	0.6196921	Wdr45b	1
GO:0035729	cellular response to hepatocyte growth factor stimulus	1/138	14/14464	0.07142857	7.48654244	2.38314036	0.12565036	0.63095922	0.6196921	Gclm	1
GO:0035810	positive regulation of urine volume	1/138	14/14464	0.07142857	7.48654244	2.38314036	0.12565036	0.63095922	0.6196921	Has2	1
GO:0036498	IRE1-mediated unfolded protein response	1/138	14/14464	0.07142857	7.48654244	2.38314036	0.12565036	0.63095922	0.6196921	Hspa5	1
GO:0043455	regulation of secondary metabolic process	1/138	14/14464	0.07142857	7.48654244	2.38314036	0.12565036	0.63095922	0.6196921	Rab38	1
GO:0044849	estrous cycle	1/138	14/14464	0.07142857	7.48654244	2.38314036	0.12565036	0.63095922	0.6196921	Has2	1
GO:0045161	neuronal ion channel clustering	1/138	14/14464	0.07142857	7.48654244	2.38314036	0.12565036	0.63095922	0.6196921	Kcnip2	1
GO:0048021	regulation of melanin biosynthetic process	1/138	14/14464	0.07142857	7.48654244	2.38314036	0.12565036	0.63095922	0.6196921	Rab38	1
GO:0051797	regulation of hair follicle development	1/138	14/14464	0.07142857	7.48654244	2.38314036	0.12565036	0.63095922	0.6196921	Pkp3	1
GO:0071285	cellular response to lithium ion	1/138	14/14464	0.07142857	7.48654244	2.38314036	0.12565036	0.63095922	0.6196921	Calr	1
GO:0071318	cellular response to ATP	1/138	14/14464	0.07142857	7.48654244	2.38314036	0.12565036	0.63095922	0.6196921	Hsp90b1	1
GO:1900376	regulation of secondary metabolite biosynthetic process	1/138	14/14464	0.07142857	7.48654244	2.38314036	0.12565036	0.63095922	0.6196921	Rab38	1
GO:0006665	sphingolipid metabolic process	3/138	129/14464	0.02325581	2.43747893	1.60954735	0.12566068	0.63095922	0.6196921	Hexa/Degs1/Elovl7	3
GO:0050684	regulation of mRNA processing	3/138	129/14464	0.02325581	2.43747893	1.60954735	0.12566068	0.63095922	0.6196921	Mettl16/Obi1/Srpk1	3
GO:0008654	phospholipid biosynthetic process	4/138	201/14464	0.0199005	2.08580287	1.5214225	0.12611966	0.63095922	0.6196921	Rab38/Dgkk/Pigg/Selenoi	4
GO:0009749	response to glucose	4/138	201/14464	0.0199005	2.08580287	1.5214225	0.12611966	0.63095922	0.6196921	Smad2/Gclm/Sri/Pdk3	4
GO:0090287	regulation of cellular response to growth factor stimulus	5/138	278/14464	0.01798561	1.88510062	1.46248468	0.12722237	0.63095922	0.6196921	Hspa5/Dand5/Smad2/Brms1/Spred3	5
GO:0050766	positive regulation of phagocytosis	2/138	65/14464	0.03076923	3.22497213	1.76449873	0.12767675	0.63095922	0.6196921	Calr/Lman2	2
GO:0051926	negative regulation of calcium ion transport	2/138	65/14464	0.03076923	3.22497213	1.76449873	0.12767675	0.63095922	0.6196921	Rrad/Sri	2
GO:1904888	cranial skeletal system development	2/138	65/14464	0.03076923	3.22497213	1.76449873	0.12767675	0.63095922	0.6196921	Mmp14/Smad2	2
GO:0170039	proteinogenic amino acid metabolic process	3/138	130/14464	0.02307692	2.4187291	1.59475405	0.12778166	0.63095922	0.6196921	Gclm/Hmgcll1/Enoph1	3
GO:0007030	Golgi organization	3/138	131/14464	0.02290076	2.40026552	1.58009702	0.12991549	0.63095922	0.6196921	Bag5/Cog6/Sec22b	3
GO:1905515	non-motile cilium assembly	2/138	66/14464	0.03030303	3.17610892	1.7390328	0.13088134	0.63095922	0.6196921	Ift172/Ift80	2
GO:0031396	regulation of protein ubiquitination	4/138	204/14464	0.01960784	2.0551293	1.48959178	0.13110653	0.63095922	0.6196921	Hspa5/Sash1/Bag5/Ndfip2	4
GO:0009746	response to hexose	4/138	205/14464	0.0195122	2.04510428	1.47910254	0.13278693	0.63095922	0.6196921	Smad2/Gclm/Sri/Pdk3	4
GO:2000146	negative regulation of cell motility	5/138	282/14464	0.0177305	1.8583616	1.42867142	0.13282116	0.63095922	0.6196921	Calr/Stard13/S2bpcox16/Pkp2/Brms1	5
GO:0006491	N-glycan processing	1/138	15/14464	0.06666667	6.98743961	2.27705826	0.13400055	0.63095922	0.6196921	Hexa	1
GO:0006595	polyamine metabolic process	1/138	15/14464	0.06666667	6.98743961	2.27705826	0.13400055	0.63095922	0.6196921	Sat1	1
GO:0019184	nonribosomal peptide biosynthetic process	1/138	15/14464	0.06666667	6.98743961	2.27705826	0.13400055	0.63095922	0.6196921	Gclm	1
GO:0030497	fatty acid elongation	1/138	15/14464	0.06666667	6.98743961	2.27705826	0.13400055	0.63095922	0.6196921	Elovl7	1
GO:0032488	Cdc42 protein signal transduction	1/138	15/14464	0.06666667	6.98743961	2.27705826	0.13400055	0.63095922	0.6196921	Rhoj	1
GO:0032926	negative regulation of activin receptor signaling pathway	1/138	15/14464	0.06666667	6.98743961	2.27705826	0.13400055	0.63095922	0.6196921	Dand5	1
GO:0035733	hepatic stellate cell activation	1/138	15/14464	0.06666667	6.98743961	2.27705826	0.13400055	0.63095922	0.6196921	Gclm	1
GO:0043558	regulation of translational initiation in response to stress	1/138	15/14464	0.06666667	6.98743961	2.27705826	0.13400055	0.63095922	0.6196921	Dnajc3	1
GO:0051284	positive regulation of sequestering of calcium ion	1/138	15/14464	0.06666667	6.98743961	2.27705826	0.13400055	0.63095922	0.6196921	Sri	1
GO:0051546	keratinocyte migration	1/138	15/14464	0.06666667	6.98743961	2.27705826	0.13400055	0.63095922	0.6196921	Has2	1
GO:0071305	cellular response to vitamin D	1/138	15/14464	0.06666667	6.98743961	2.27705826	0.13400055	0.63095922	0.6196921	Pdia3	1
GO:0090136	epithelial cell-cell adhesion	1/138	15/14464	0.06666667	6.98743961	2.27705826	0.13400055	0.63095922	0.6196921	Pkp3	1
GO:0097094	craniofacial suture morphogenesis	1/138	15/14464	0.06666667	6.98743961	2.27705826	0.13400055	0.63095922	0.6196921	Mmp14	1
GO:0097623	potassium ion export across plasma membrane	1/138	15/14464	0.06666667	6.98743961	2.27705826	0.13400055	0.63095922	0.6196921	Kcnip2	1
GO:0098528	skeletal muscle fiber differentiation	1/138	15/14464	0.06666667	6.98743961	2.27705826	0.13400055	0.63095922	0.6196921	Fktn	1
GO:0110096	cellular response to aldehyde	1/138	15/14464	0.06666667	6.98743961	2.27705826	0.13400055	0.63095922	0.6196921	Akr1a1	1
GO:1900102	negative regulation of endoplasmic reticulum unfolded protein response	1/138	15/14464	0.06666667	6.98743961	2.27705826	0.13400055	0.63095922	0.6196921	Hspa5	1
GO:1901334	lactone metabolic process	1/138	15/14464	0.06666667	6.98743961	2.27705826	0.13400055	0.63095922	0.6196921	Akr1a1	1
GO:0070534	protein K63-linked ubiquitination	2/138	67/14464	0.02985075	3.1287043	1.71404808	0.13410449	0.63095922	0.6196921	Sash1/Itch	2
GO:0006720	isoprenoid metabolic process	2/138	68/14464	0.02941176	3.08269395	1.68952746	0.13734556	0.63095922	0.6196921	Dhrs3/Crppa	2
GO:0007492	endoderm development	2/138	68/14464	0.02941176	3.08269395	1.68952746	0.13734556	0.63095922	0.6196921	Mmp14/Smad2	2
GO:0071806	protein transmembrane transport	2/138	68/14464	0.02941176	3.08269395	1.68952746	0.13734556	0.63095922	0.6196921	Hspa5/Hspd1	2
GO:0006360	transcription by RNA polymerase I	2/138	69/14464	0.02898551	3.03801722	1.6654547	0.14060394	0.63095922	0.6196921	Rasl11a/Taf1a	2
GO:0010632	regulation of epithelial cell migration	2/138	69/14464	0.02898551	3.03801722	1.6654547	0.14060394	0.63095922	0.6196921	Has2/Sash1	2
GO:0007565	female pregnancy	3/138	136/14464	0.02205882	2.31202046	1.50877173	0.14077114	0.63095922	0.6196921	Calr/Cr1l/Tppp3	3
GO:0002837	regulation of immune response to tumor cell	1/138	16/14464	0.0625	6.55072464	2.180279	0.14227157	0.63095922	0.6196921	Hspd1	1
GO:0007398	ectoderm development	1/138	16/14464	0.0625	6.55072464	2.180279	0.14227157	0.63095922	0.6196921	Grhl3	1
GO:0009148	pyrimidine nucleoside triphosphate biosynthetic process	1/138	16/14464	0.0625	6.55072464	2.180279	0.14227157	0.63095922	0.6196921	Tbpl1	1
GO:0010644	cell communication by electrical coupling	1/138	16/14464	0.0625	6.55072464	2.180279	0.14227157	0.63095922	0.6196921	Sri	1
GO:0018410	C-terminal protein amino acid modification	1/138	16/14464	0.0625	6.55072464	2.180279	0.14227157	0.63095922	0.6196921	Wdr45b	1
GO:0030449	regulation of complement activation	1/138	16/14464	0.0625	6.55072464	2.180279	0.14227157	0.63095922	0.6196921	Cr1l	1
GO:0034497	protein localization to phagophore assembly site	1/138	16/14464	0.0625	6.55072464	2.180279	0.14227157	0.63095922	0.6196921	Wdr45b	1
GO:0035728	response to hepatocyte growth factor	1/138	16/14464	0.0625	6.55072464	2.180279	0.14227157	0.63095922	0.6196921	Gclm	1
GO:0042921	nuclear receptor-mediated glucocorticoid signaling pathway	1/138	16/14464	0.0625	6.55072464	2.180279	0.14227157	0.63095922	0.6196921	Bmal1	1
GO:0046514	ceramide catabolic process	1/138	16/14464	0.0625	6.55072464	2.180279	0.14227157	0.63095922	0.6196921	Hexa	1
GO:0050862	positive regulation of T cell receptor signaling pathway	1/138	16/14464	0.0625	6.55072464	2.180279	0.14227157	0.63095922	0.6196921	Card11	1
GO:0050891	multicellular organismal-level water homeostasis	1/138	16/14464	0.0625	6.55072464	2.180279	0.14227157	0.63095922	0.6196921	Has2	1
GO:0051014	actin filament severing	1/138	16/14464	0.0625	6.55072464	2.180279	0.14227157	0.63095922	0.6196921	Scin	1
GO:0051895	negative regulation of focal adhesion assembly	1/138	16/14464	0.0625	6.55072464	2.180279	0.14227157	0.63095922	0.6196921	Mmp14	1
GO:0061029	eyelid development in camera-type eye	1/138	16/14464	0.0625	6.55072464	2.180279	0.14227157	0.63095922	0.6196921	Grhl3	1
GO:0090051	negative regulation of cell migration involved in sprouting angiogenesis	1/138	16/14464	0.0625	6.55072464	2.180279	0.14227157	0.63095922	0.6196921	Stard13	1
GO:0098712	L-glutamate import across plasma membrane	1/138	16/14464	0.0625	6.55072464	2.180279	0.14227157	0.63095922	0.6196921	Slc17a8	1
GO:0150118	negative regulation of cell-substrate junction organization	1/138	16/14464	0.0625	6.55072464	2.180279	0.14227157	0.63095922	0.6196921	Mmp14	1
GO:1901017	negative regulation of potassium ion transmembrane transporter activity	1/138	16/14464	0.0625	6.55072464	2.180279	0.14227157	0.63095922	0.6196921	Oxsr1	1
GO:1901077	regulation of relaxation of muscle	1/138	16/14464	0.0625	6.55072464	2.180279	0.14227157	0.63095922	0.6196921	Sri	1
GO:1901985	positive regulation of protein acetylation	1/138	16/14464	0.0625	6.55072464	2.180279	0.14227157	0.63095922	0.6196921	Bmal1	1
GO:0001666	response to hypoxia	5/138	289/14464	0.01730104	1.81334938	1.37078957	0.14288216	0.63095922	0.6196921	Mmp14/Hsp90b1/Cr1l/Pdk3/P4hb	5
GO:0034284	response to monosaccharide	4/138	211/14464	0.01895735	1.98694965	1.41738994	0.14305419	0.63095922	0.6196921	Smad2/Gclm/Sri/Pdk3	4
GO:0033143	regulation of intracellular steroid hormone receptor signaling pathway	2/138	70/14464	0.02857143	2.99461698	1.64181434	0.14387899	0.63095922	0.6196921	Bmal1/Calr	2
GO:0006813	potassium ion transport	4/138	212/14464	0.01886792	1.97757725	1.40730222	0.14479549	0.63095922	0.6196921	Kcnn1/Kcnk13/Kcnip2/Oxsr1	4
GO:1905475	regulation of protein localization to membrane	4/138	212/14464	0.01886792	1.97757725	1.40730222	0.14479549	0.63095922	0.6196921	Slc5a3/Mmp14/Arhgef16/Cnpy4	4
GO:0003015	heart process	4/138	213/14464	0.01877934	1.96829285	1.39726931	0.14654518	0.63095922	0.6196921	Kcnip2/Ppcs/Pkp2/Sri	4
GO:0072593	reactive oxygen species metabolic process	4/138	213/14464	0.01877934	1.96829285	1.39726931	0.14654518	0.63095922	0.6196921	Slc5a3/Hspd1/Sh3pxd2a/Pdk3	4
GO:0072659	protein localization to plasma membrane	5/138	292/14464	0.01712329	1.79471908	1.34646713	0.14729367	0.63095922	0.6196921	Mmp14/Arhgef16/Cnpy4/Pkp3/Pkp2	5
GO:0141091	transforming growth factor beta receptor superfamily signaling pathway	5/138	292/14464	0.01712329	1.79471908	1.34646713	0.14729367	0.63095922	0.6196921	Hspa5/Dand5/Smad2/Brms1/Spred3	5
GO:0071456	cellular response to hypoxia	3/138	140/14464	0.02142857	2.24596273	1.45392893	0.14966866	0.63095922	0.6196921	Cr1l/Pdk3/P4hb	3
GO:0002834	regulation of response to tumor cell	1/138	17/14464	0.05882353	6.16538789	2.0914372	0.15046416	0.63095922	0.6196921	Hspd1	1
GO:0003184	pulmonary valve morphogenesis	1/138	17/14464	0.05882353	6.16538789	2.0914372	0.15046416	0.63095922	0.6196921	Smad2	1
GO:0003323	type B pancreatic cell development	1/138	17/14464	0.05882353	6.16538789	2.0914372	0.15046416	0.63095922	0.6196921	Bmal1	1
GO:0009263	deoxyribonucleotide biosynthetic process	1/138	17/14464	0.05882353	6.16538789	2.0914372	0.15046416	0.63095922	0.6196921	Tbpl1	1
GO:0009265	2'-deoxyribonucleotide biosynthetic process	1/138	17/14464	0.05882353	6.16538789	2.0914372	0.15046416	0.63095922	0.6196921	Tbpl1	1
GO:0010226	response to lithium ion	1/138	17/14464	0.05882353	6.16538789	2.0914372	0.15046416	0.63095922	0.6196921	Calr	1
GO:0021854	hypothalamus development	1/138	17/14464	0.05882353	6.16538789	2.0914372	0.15046416	0.63095922	0.6196921	Sema3e	1
GO:0036336	dendritic cell migration	1/138	17/14464	0.05882353	6.16538789	2.0914372	0.15046416	0.63095922	0.6196921	Calr	1
GO:0040019	positive regulation of embryonic development	1/138	17/14464	0.05882353	6.16538789	2.0914372	0.15046416	0.63095922	0.6196921	Hspa5	1
GO:0042762	regulation of sulfur metabolic process	1/138	17/14464	0.05882353	6.16538789	2.0914372	0.15046416	0.63095922	0.6196921	Pdk3	1
GO:0043217	myelin maintenance	1/138	17/14464	0.05882353	6.16538789	2.0914372	0.15046416	0.63095922	0.6196921	Degs1	1
GO:0045579	positive regulation of B cell differentiation	1/138	17/14464	0.05882353	6.16538789	2.0914372	0.15046416	0.63095922	0.6196921	Mmp14	1
GO:0046385	deoxyribose phosphate biosynthetic process	1/138	17/14464	0.05882353	6.16538789	2.0914372	0.15046416	0.63095922	0.6196921	Tbpl1	1
GO:0048532	anatomical structure arrangement	1/138	17/14464	0.05882353	6.16538789	2.0914372	0.15046416	0.63095922	0.6196921	Hspa5	1
GO:0050687	negative regulation of defense response to virus	1/138	17/14464	0.05882353	6.16538789	2.0914372	0.15046416	0.63095922	0.6196921	Itch	1
GO:0051220	cytoplasmic sequestering of protein	1/138	17/14464	0.05882353	6.16538789	2.0914372	0.15046416	0.63095922	0.6196921	Sri	1
GO:0051444	negative regulation of ubiquitin-protein transferase activity	1/138	17/14464	0.05882353	6.16538789	2.0914372	0.15046416	0.63095922	0.6196921	Bag5	1
GO:0060019	radial glial cell differentiation	1/138	17/14464	0.05882353	6.16538789	2.0914372	0.15046416	0.63095922	0.6196921	Mettl14	1
GO:0060314	regulation of ryanodine-sensitive calcium-release channel activity	1/138	17/14464	0.05882353	6.16538789	2.0914372	0.15046416	0.63095922	0.6196921	Sri	1
GO:0070417	cellular response to cold	1/138	17/14464	0.05882353	6.16538789	2.0914372	0.15046416	0.63095922	0.6196921	Dnajc3	1
GO:0070486	leukocyte aggregation	1/138	17/14464	0.05882353	6.16538789	2.0914372	0.15046416	0.63095922	0.6196921	Has2	1
GO:0071816	tail-anchored membrane protein insertion into ER membrane	1/138	17/14464	0.05882353	6.16538789	2.0914372	0.15046416	0.63095922	0.6196921	Ubl4a	1
GO:0141198	protein branched polyubiquitination	1/138	17/14464	0.05882353	6.16538789	2.0914372	0.15046416	0.63095922	0.6196921	Itch	1
GO:1901623	regulation of lymphocyte chemotaxis	1/138	17/14464	0.05882353	6.16538789	2.0914372	0.15046416	0.63095922	0.6196921	Oxsr1	1
GO:1903902	positive regulation of viral life cycle	1/138	17/14464	0.05882353	6.16538789	2.0914372	0.15046416	0.63095922	0.6196921	P4hb	1
GO:0009798	axis specification	2/138	72/14464	0.02777778	2.91143317	1.59577279	0.15047672	0.63095922	0.6196921	Smad2/Ift172	2
GO:0030968	endoplasmic reticulum unfolded protein response	2/138	72/14464	0.02777778	2.91143317	1.59577279	0.15047672	0.63095922	0.6196921	Hspa5/Dnajc3	2
GO:0070373	negative regulation of ERK1 and ERK2 cascade	2/138	72/14464	0.02777778	2.91143317	1.59577279	0.15047672	0.63095922	0.6196921	S2bpcox16/Spred3	2
GO:2000377	regulation of reactive oxygen species metabolic process	3/138	141/14464	0.0212766	2.23003392	1.44050878	0.15192112	0.63095922	0.6196921	Slc5a3/Hspd1/Pdk3	3
GO:0072676	lymphocyte migration	2/138	73/14464	0.02739726	2.87155053	1.57334427	0.15379821	0.63095922	0.6196921	Cd99l2/Oxsr1	2
GO:1903510	mucopolysaccharide metabolic process	2/138	73/14464	0.02739726	2.87155053	1.57334427	0.15379821	0.63095922	0.6196921	Has2/Hexa	2
GO:0050680	negative regulation of epithelial cell proliferation	3/138	142/14464	0.02112676	2.21432945	1.42720096	0.15418445	0.63095922	0.6196921	Ift172/S2bpcox16/Ift80	3
GO:0040013	negative regulation of locomotion	5/138	297/14464	0.01683502	1.76450495	1.30654943	0.15477481	0.63095922	0.6196921	Calr/Stard13/S2bpcox16/Pkp2/Brms1	5
GO:0006942	regulation of striated muscle contraction	2/138	74/14464	0.02702703	2.83274579	1.55129344	0.157134	0.63095922	0.6196921	Pkp2/Sri	2
GO:0034109	homotypic cell-cell adhesion	2/138	74/14464	0.02702703	2.83274579	1.55129344	0.157134	0.63095922	0.6196921	Pdia3/Pkp2	2
GO:0048762	mesenchymal cell differentiation	4/138	219/14464	0.01826484	1.91436702	1.33818959	0.15721497	0.63095922	0.6196921	Has2/Smad2/Sema3e/Spred3	4
GO:0001976	nervous system process involved in regulation of systemic arterial blood pressure	1/138	18/14464	0.05555556	5.82286634	2.00943488	0.15857906	0.63095922	0.6196921	Manf	1
GO:0002418	immune response to tumor cell	1/138	18/14464	0.05555556	5.82286634	2.00943488	0.15857906	0.63095922	0.6196921	Hspd1	1
GO:0003159	morphogenesis of an endothelium	1/138	18/14464	0.05555556	5.82286634	2.00943488	0.15857906	0.63095922	0.6196921	Stard13	1
GO:0003283	atrial septum development	1/138	18/14464	0.05555556	5.82286634	2.00943488	0.15857906	0.63095922	0.6196921	Dand5	1
GO:0006614	SRP-dependent cotranslational protein targeting to membrane	1/138	18/14464	0.05555556	5.82286634	2.00943488	0.15857906	0.63095922	0.6196921	Srprb	1
GO:0009067	aspartate family amino acid biosynthetic process	1/138	18/14464	0.05555556	5.82286634	2.00943488	0.15857906	0.63095922	0.6196921	Enoph1	1
GO:0031958	nuclear receptor-mediated corticosteroid signaling pathway	1/138	18/14464	0.05555556	5.82286634	2.00943488	0.15857906	0.63095922	0.6196921	Bmal1	1
GO:0032354	response to follicle-stimulating hormone	1/138	18/14464	0.05555556	5.82286634	2.00943488	0.15857906	0.63095922	0.6196921	Gclm	1
GO:0042438	melanin biosynthetic process	1/138	18/14464	0.05555556	5.82286634	2.00943488	0.15857906	0.63095922	0.6196921	Rab38	1
GO:0045061	thymic T cell selection	1/138	18/14464	0.05555556	5.82286634	2.00943488	0.15857906	0.63095922	0.6196921	Card11	1
GO:0051580	regulation of neurotransmitter uptake	1/138	18/14464	0.05555556	5.82286634	2.00943488	0.15857906	0.63095922	0.6196921	Slc17a8	1

GO:0061154	endothelial tube morphogenesis	1/138	18/14464	0.05555556	5.82286634	2.00943488	0.15857906	0.63095922	0.6196921	Stard13	1
GO:0071071	regulation of phospholipid biosynthetic process	1/138	18/14464	0.05555556	5.82286634	2.00943488	0.15857906	0.63095922	0.6196921	Rab38	1
GO:0071218	cellular response to misfolded protein	1/138	18/14464	0.05555556	5.82286634	2.00943488	0.15857906	0.63095922	0.6196921	Sdf2l1	1
GO:0071498	cellular response to fluid shear stress	1/138	18/14464	0.05555556	5.82286634	2.00943488	0.15857906	0.63095922	0.6196921	Has2	1
GO:0090083	regulation of inclusion body assembly	1/138	18/14464	0.05555556	5.82286634	2.00943488	0.15857906	0.63095922	0.6196921	Bag5	1
GO:1903232	melanosome assembly	1/138	18/14464	0.05555556	5.82286634	2.00943488	0.15857906	0.63095922	0.6196921	Rab38	1
GO:0016485	protein processing	4/138	220/14464	0.01818182	1.90566535	1.3285239	0.15902113	0.63095922	0.6196921	Mmp14/Hsp90b1/Ift172/P4hb	4
GO:0006493	protein O-linked glycosylation	2/138	75/14464	0.02666667	2.79497585	1.52960818	0.16048353	0.63095922	0.6196921	Fktn/Crppa	2
GO:0044283	small molecule biosynthetic process	7/138	470/14464	0.01489362	1.56102374	1.21357186	0.16276294	0.63095922	0.6196921	Degs1/Hmgcll1/Elovl7/Lta4h/Arpp19/Enoph1/Akr1a1	7
GO:0036293	response to decreased oxygen levels	5/138	304/14464	0.01644737	1.7238749	1.2519153	0.1655092	0.63095922	0.6196921	Mmp14/Hsp90b1/Cr1l/Pdk3/P4hb	5
GO:0043488	regulation of mRNA stability	3/138	147/14464	0.02040816	2.13901213	1.36228278	0.16565797	0.63095922	0.6196921	Mettl16/Hnrnpc/Mettl14	3
GO:0043547	positive regulation of GTPase activity	3/138	147/14464	0.02040816	2.13901213	1.36228278	0.16565797	0.63095922	0.6196921	Arhgef16/Grhl3/Snx18	3
GO:0002507	tolerance induction	1/138	19/14464	0.05263158	5.51639969	1.9333775	0.166617	0.63095922	0.6196921	Itch	1
GO:0003084	positive regulation of systemic arterial blood pressure	1/138	19/14464	0.05263158	5.51639969	1.9333775	0.166617	0.63095922	0.6196921	Manf	1
GO:0003417	growth plate cartilage development	1/138	19/14464	0.05263158	5.51639969	1.9333775	0.166617	0.63095922	0.6196921	Ift80	1
GO:0006613	cotranslational protein targeting to membrane	1/138	19/14464	0.05263158	5.51639969	1.9333775	0.166617	0.63095922	0.6196921	Srprb	1
GO:0006817	phosphate ion transport	1/138	19/14464	0.05263158	5.51639969	1.9333775	0.166617	0.63095922	0.6196921	Slc17a8	1
GO:0010288	response to lead ion	1/138	19/14464	0.05263158	5.51639969	1.9333775	0.166617	0.63095922	0.6196921	Ppp2cb	1
GO:0010804	negative regulation of tumor necrosis factor-mediated signaling pathway	1/138	19/14464	0.05263158	5.51639969	1.9333775	0.166617	0.63095922	0.6196921	Ppp2cb	1
GO:0015936	coenzyme A metabolic process	1/138	19/14464	0.05263158	5.51639969	1.9333775	0.166617	0.63095922	0.6196921	Ppcs	1
GO:0044550	secondary metabolite biosynthetic process	1/138	19/14464	0.05263158	5.51639969	1.9333775	0.166617	0.63095922	0.6196921	Rab38	1
GO:0045217	cell-cell junction maintenance	1/138	19/14464	0.05263158	5.51639969	1.9333775	0.166617	0.63095922	0.6196921	Pkp2	1
GO:0046641	positive regulation of alpha-beta T cell proliferation	1/138	19/14464	0.05263158	5.51639969	1.9333775	0.166617	0.63095922	0.6196921	Card11	1
GO:0048339	paraxial mesoderm development	1/138	19/14464	0.05263158	5.51639969	1.9333775	0.166617	0.63095922	0.6196921	Smad2	1
GO:0050884	neuromuscular process controlling posture	1/138	19/14464	0.05263158	5.51639969	1.9333775	0.166617	0.63095922	0.6196921	Hexa	1
GO:0051788	response to misfolded protein	1/138	19/14464	0.05263158	5.51639969	1.9333775	0.166617	0.63095922	0.6196921	Sdf2l1	1
GO:0051930	regulation of sensory perception of pain	1/138	19/14464	0.05263158	5.51639969	1.9333775	0.166617	0.63095922	0.6196921	Smr2l	1
GO:0071371	cellular response to gonadotropin stimulus	1/138	19/14464	0.05263158	5.51639969	1.9333775	0.166617	0.63095922	0.6196921	Gclm	1
GO:0071474	cellular hyperosmotic response	1/138	19/14464	0.05263158	5.51639969	1.9333775	0.166617	0.63095922	0.6196921	Oxsr1	1
GO:0097320	plasma membrane tubulation	1/138	19/14464	0.05263158	5.51639969	1.9333775	0.166617	0.63095922	0.6196921	Snx18	1
GO:1901379	regulation of potassium ion transmembrane transport	2/138	77/14464	0.02597403	2.72237907	1.48728858	0.16722157	0.63095922	0.6196921	Kcnip2/Oxsr1	2
GO:0001935	endothelial cell proliferation	3/138	148/14464	0.02027027	2.12455934	1.34961114	0.16798279	0.63095922	0.6196921	Mmp14/S2bpcox16/Aggf1	3
GO:0006402	mRNA catabolic process	4/138	226/14464	0.01769912	1.85507246	1.27156601	0.17001782	0.63095922	0.6196921	Mettl16/Hnrnpc/Pkp3/Mettl14	4
GO:0001837	epithelial to mesenchymal transition	3/138	149/14464	0.02013423	2.11030055	1.33703962	0.17031723	0.63095922	0.6196921	Has2/Smad2/Spred3	3
GO:0036294	cellular response to decreased oxygen levels	3/138	149/14464	0.02013423	2.11030055	1.33703962	0.17031723	0.63095922	0.6196921	Cr1l/Pdk3/P4hb	3
GO:0042100	B cell proliferation	2/138	78/14464	0.02564103	2.68747677	1.46663262	0.17060899	0.63095922	0.6196921	Card11/Hspd1	2
GO:0050871	positive regulation of B cell activation	2/138	78/14464	0.02564103	2.68747677	1.46663262	0.17060899	0.63095922	0.6196921	Card11/Mmp14	2
GO:0051090	regulation of DNA-binding transcription factor activity	5/138	308/14464	0.01623377	1.70148692	1.22132408	0.17177439	0.63095922	0.6196921	Card11/Sri/Ppp2cb/Brms1/Itch	5
GO:0035914	skeletal muscle cell differentiation	2/138	79/14464	0.02531646	2.65345808	1.44629893	0.17400796	0.63095922	0.6196921	Bmal1/Fktn	2
GO:0006027	glycosaminoglycan catabolic process	1/138	20/14464	0.05	5.24057971	1.86252775	0.1745787	0.63095922	0.6196921	Hexa	1
GO:0006582	melanin metabolic process	1/138	20/14464	0.05	5.24057971	1.86252775	0.1745787	0.63095922	0.6196921	Rab38	1
GO:0006656	phosphatidylcholine biosynthetic process	1/138	20/14464	0.05	5.24057971	1.86252775	0.1745787	0.63095922	0.6196921	Rab38	1
GO:0007289	spermatid nucleus differentiation	1/138	20/14464	0.05	5.24057971	1.86252775	0.1745787	0.63095922	0.6196921	Tbpl1	1
GO:0009110	vitamin biosynthetic process	1/138	20/14464	0.05	5.24057971	1.86252775	0.1745787	0.63095922	0.6196921	Akr1a1	1
GO:0009147	pyrimidine nucleoside triphosphate metabolic process	1/138	20/14464	0.05	5.24057971	1.86252775	0.1745787	0.63095922	0.6196921	Tbpl1	1
GO:0009219	pyrimidine deoxyribonucleotide metabolic process	1/138	20/14464	0.05	5.24057971	1.86252775	0.1745787	0.63095922	0.6196921	Tbpl1	1
GO:0010958	regulation of amino acid import across plasma membrane	1/138	20/14464	0.05	5.24057971	1.86252775	0.1745787	0.63095922	0.6196921	Slc17a8	1
GO:0032211	negative regulation of telomere maintenance via telomerase	1/138	20/14464	0.05	5.24057971	1.86252775	0.1745787	0.63095922	0.6196921	Hnrnpc	1
GO:0042044	fluid transport	1/138	20/14464	0.05	5.24057971	1.86252775	0.1745787	0.63095922	0.6196921	Has2	1
GO:0042753	positive regulation of circadian rhythm	1/138	20/14464	0.05	5.24057971	1.86252775	0.1745787	0.63095922	0.6196921	Bmal1	1
GO:0051931	regulation of sensory perception	1/138	20/14464	0.05	5.24057971	1.86252775	0.1745787	0.63095922	0.6196921	Smr2l	1
GO:0060155	platelet dense granule organization	1/138	20/14464	0.05	5.24057971	1.86252775	0.1745787	0.63095922	0.6196921	Rab38	1
GO:0071295	cellular response to vitamin	1/138	20/14464	0.05	5.24057971	1.86252775	0.1745787	0.63095922	0.6196921	Pdia3	1
GO:0097066	response to thyroid hormone	1/138	20/14464	0.05	5.24057971	1.86252775	0.1745787	0.63095922	0.6196921	Gclm	1
GO:1901201	regulation of extracellular matrix assembly	1/138	20/14464	0.05	5.24057971	1.86252775	0.1745787	0.63095922	0.6196921	Has2	1
GO:1903427	negative regulation of reactive oxygen species biosynthetic process	1/138	20/14464	0.05	5.24057971	1.86252775	0.1745787	0.63095922	0.6196921	Hspd1	1
GO:1903428	positive regulation of reactive oxygen species biosynthetic process	1/138	20/14464	0.05	5.24057971	1.86252775	0.1745787	0.63095922	0.6196921	Slc5a3	1
GO:1903789	regulation of amino acid transmembrane transport	1/138	20/14464	0.05	5.24057971	1.86252775	0.1745787	0.63095922	0.6196921	Slc17a8	1
GO:2000786	positive regulation of autophagosome assembly	1/138	20/14464	0.05	5.24057971	1.86252775	0.1745787	0.63095922	0.6196921	Snx18	1
GO:0043010	camera-type eye development	5/138	311/14464	0.01607717	1.68507386	1.19866991	0.17653363	0.63095922	0.6196921	Rhoj/Grhl3/Slc17a8/Ift172/Spred3	5
GO:0042445	hormone metabolic process	3/138	152/14464	0.01973684	2.06864989	1.29991016	0.17737652	0.63095922	0.6196921	Dhrs3/Hsp90b1/P4hb	3
GO:0001667	ameboidal-type cell migration	4/138	231/14464	0.01731602	1.81491938	1.22540225	0.17938181	0.63095922	0.6196921	Calr/Has2/Sash1/Sema3e	4
GO:0007178	cell surface receptor protein serine/threonine kinase signaling pathway	5/138	313/14464	0.01597444	1.67430662	1.18370174	0.17973447	0.63095922	0.6196921	Hspa5/Dand5/Smad2/Brms1/Spred3	5
GO:0002690	positive regulation of leukocyte chemotaxis	2/138	81/14464	0.02469136	2.5879406	1.40656007	0.18083848	0.63095922	0.6196921	Calr/Oxsr1	2
GO:0021510	spinal cord development	2/138	81/14464	0.02469136	2.5879406	1.40656007	0.18083848	0.63095922	0.6196921	Ift172/Ift80	2
GO:0033077	T cell differentiation in thymus	2/138	81/14464	0.02469136	2.5879406	1.40656007	0.18083848	0.63095922	0.6196921	Card11/Fzd8	2
GO:1905897	regulation of response to endoplasmic reticulum stress	2/138	81/14464	0.02469136	2.5879406	1.40656007	0.18083848	0.63095922	0.6196921	Hspa5/Manf	2
GO:0000413	protein peptidyl-prolyl isomerization	1/138	21/14464	0.04761905	4.9910283	1.79627163	0.18246489	0.63095922	0.6196921	Fkbp2	1
GO:0003177	pulmonary valve development	1/138	21/14464	0.04761905	4.9910283	1.79627163	0.18246489	0.63095922	0.6196921	Smad2	1
GO:0003309	type B pancreatic cell differentiation	1/138	21/14464	0.04761905	4.9910283	1.79627163	0.18246489	0.63095922	0.6196921	Bmal1	1
GO:0006654	phosphatidic acid biosynthetic process	1/138	21/14464	0.04761905	4.9910283	1.79627163	0.18246489	0.63095922	0.6196921	Dgkk	1
GO:0006662	glycerol ether metabolic process	1/138	21/14464	0.04761905	4.9910283	1.79627163	0.18246489	0.63095922	0.6196921	Selenoi	1
GO:0006691	leukotriene metabolic process	1/138	21/14464	0.04761905	4.9910283	1.79627163	0.18246489	0.63095922	0.6196921	Lta4h	1
GO:0010954	positive regulation of protein processing	1/138	21/14464	0.04761905	4.9910283	1.79627163	0.18246489	0.63095922	0.6196921	Mmp14	1
GO:0043576	regulation of respiratory gaseous exchange	1/138	21/14464	0.04761905	4.9910283	1.79627163	0.18246489	0.63095922	0.6196921	Slc5a3	1
GO:0045116	protein neddylation	1/138	21/14464	0.04761905	4.9910283	1.79627163	0.18246489	0.63095922	0.6196921	Aktip	1
GO:0045652	regulation of megakaryocyte differentiation	1/138	21/14464	0.04761905	4.9910283	1.79627163	0.18246489	0.63095922	0.6196921	Scin	1
GO:0045948	positive regulation of translational initiation	1/138	21/14464	0.04761905	4.9910283	1.79627163	0.18246489	0.63095922	0.6196921	Dnajc3	1
GO:0046485	ether lipid metabolic process	1/138	21/14464	0.04761905	4.9910283	1.79627163	0.18246489	0.63095922	0.6196921	Selenoi	1
GO:0072537	fibroblast activation	1/138	21/14464	0.04761905	4.9910283	1.79627163	0.18246489	0.63095922	0.6196921	Gclm	1
GO:0086019	cell-cell signaling involved in cardiac conduction	1/138	21/14464	0.04761905	4.9910283	1.79627163	0.18246489	0.63095922	0.6196921	Pkp2	1
GO:0090050	positive regulation of cell migration involved in sprouting angiogenesis	1/138	21/14464	0.04761905	4.9910283	1.79627163	0.18246489	0.63095922	0.6196921	Rhoj	1
GO:0090103	cochlea morphogenesis	1/138	21/14464	0.04761905	4.9910283	1.79627163	0.18246489	0.63095922	0.6196921	Grhl3	1
GO:1901380	negative regulation of potassium ion transmembrane transport	1/138	21/14464	0.04761905	4.9910283	1.79627163	0.18246489	0.63095922	0.6196921	Oxsr1	1
GO:1902455	negative regulation of stem cell population maintenance	1/138	21/14464	0.04761905	4.9910283	1.79627163	0.18246489	0.63095922	0.6196921	Brms1	1
GO:2000209	regulation of anoikis	1/138	21/14464	0.04761905	4.9910283	1.79627163	0.18246489	0.63095922	0.6196921	Brms1	1
GO:0061157	mRNA destabilization	2/138	82/14464	0.02439024	2.55638035	1.38713677	0.18426901	0.63208191	0.62079474	Mettl16/Mettl14	2
GO:1901222	regulation of non-canonical NF-kappaB signal transduction	2/138	82/14464	0.02439024	2.55638035	1.38713677	0.18426901	0.63208191	0.62079474	Calr/Sash1	2
GO:0009743	response to carbohydrate	4/138	234/14464	0.01709402	1.79165118	1.19824495	0.18508281	0.63208191	0.62079474	Smad2/Gclm/Sri/Pdk3	4
GO:0030510	regulation of BMP signaling pathway	2/138	83/14464	0.02409639	2.52558058	1.36799944	0.18770905	0.63208191	0.62079474	Dand5/Smad2	2
GO:0030522	intracellular receptor signaling pathway	5/138	318/14464	0.01572327	1.64798104	1.14674088	0.1878318	0.63208191	0.62079474	Bmal1/Dhrs3/Calr/Itch/Pdk3	5
GO:0001675	acrosome assembly	1/138	22/14464	0.04545455	4.76416337	1.73409305	0.19027628	0.63208191	0.62079474	Tbpl1	1
GO:0002693	positive regulation of cellular extravasation	1/138	22/14464	0.04545455	4.76416337	1.73409305	0.19027628	0.63208191	0.62079474	Cd99l2	1
GO:0009083	branched-chain amino acid catabolic process	1/138	22/14464	0.04545455	4.76416337	1.73409305	0.19027628	0.63208191	0.62079474	Hmgcll1	1
GO:0032925	regulation of activin receptor signaling pathway	1/138	22/14464	0.04545455	4.76416337	1.73409305	0.19027628	0.63208191	0.62079474	Dand5	1
GO:0035357	peroxisome proliferator activated receptor signaling pathway	1/138	22/14464	0.04545455	4.76416337	1.73409305	0.19027628	0.63208191	0.62079474	Pdk3	1
GO:0035809	regulation of urine volume	1/138	22/14464	0.04545455	4.76416337	1.73409305	0.19027628	0.63208191	0.62079474	Has2	1
GO:0045822	negative regulation of heart contraction	1/138	22/14464	0.04545455	4.76416337	1.73409305	0.19027628	0.63208191	0.62079474	Sri	1
GO:0046337	phosphatidylethanolamine metabolic process	1/138	22/14464	0.04545455	4.76416337	1.73409305	0.19027628	0.63208191	0.62079474	Selenoi	1
GO:0051900	regulation of mitochondrial depolarization	1/138	22/14464	0.04545455	4.76416337	1.73409305	0.19027628	0.63208191	0.62079474	Gclm	1
GO:0060039	pericardium development	1/138	22/14464	0.04545455	4.76416337	1.73409305	0.19027628	0.63208191	0.62079474	Smad2	1
GO:0061298	retina vasculature development in camera-type eye	1/138	22/14464	0.04545455	4.76416337	1.73409305	0.19027628	0.63208191	0.62079474	Rhoj	1
GO:2000810	regulation of bicellular tight junction assembly	1/138	22/14464	0.04545455	4.76416337	1.73409305	0.19027628	0.63208191	0.62079474	Pkp2	1
GO:0008593	regulation of Notch signaling pathway	2/138	84/14464	0.02380952	2.49551415	1.34913991	0.19115811	0.63208191	0.62079474	Mmp14/S2bpcox16	2
GO:0006664	glycolipid metabolic process	2/138	85/14464	0.02352941	2.46615516	1.33055036	0.19461572	0.63208191	0.62079474	Pigg/Hexa	2
GO:0030203	glycosaminoglycan metabolic process	2/138	85/14464	0.02352941	2.46615516	1.33055036	0.19461572	0.63208191	0.62079474	Has2/Hexa	2
GO:0060562	epithelial tube morphogenesis	5/138	323/14464	0.01547988	1.6224705	1.11041734	0.19606023	0.63208191	0.62079474	Mmp14/Grhl3/Stard13/Ift172/Sema3e	5
GO:0031647	regulation of protein stability	5/138	324/14464	0.0154321	1.61746287	1.10322708	0.1977211	0.63208191	0.62079474	Calr/Hspd1/Ubl4a/Bag5/Ift80	5
GO:0006390	mitochondrial transcription	1/138	23/14464	0.04347826	4.55702583	1.67555453	0.19801357	0.63208191	0.62079474	Tfb1m	1
GO:0010669	epithelial structure maintenance	1/138	23/14464	0.04347826	4.55702583	1.67555453	0.19801357	0.63208191	0.62079474	Pkp3	1
GO:0021884	forebrain neuron development	1/138	23/14464	0.04347826	4.55702583	1.67555453	0.19801357	0.63208191	0.62079474	Sema3e	1
GO:0031295	T cell costimulation	1/138	23/14464	0.04347826	4.55702583	1.67555453	0.19801357	0.63208191	0.62079474	Card11	1
GO:0032482	Rab protein signal transduction	1/138	23/14464	0.04347826	4.55702583	1.67555453	0.19801357	0.63208191	0.62079474	Rab9b	1
GO:0032727	positive regulation of interferon-alpha production	1/138	23/14464	0.04347826	4.55702583	1.67555453	0.19801357	0.63208191	0.62079474	Hspd1	1
GO:0035988	chondrocyte proliferation	1/138	23/14464	0.04347826	4.55702583	1.67555453	0.19801357	0.63208191	0.62079474	Mmp14	1
GO:0043032	positive regulation of macrophage activation	1/138	23/14464	0.04347826	4.55702583	1.67555453	0.19801357	0.63208191	0.62079474	Hspd1	1
GO:0043043	peptide biosynthetic process	1/138	23/14464	0.04347826	4.55702583	1.67555453	0.19801357	0.63208191	0.62079474	Gclm	1
GO:0043555	regulation of translation in response to stress	1/138	23/14464	0.04347826	4.55702583	1.67555453	0.19801357	0.63208191	0.62079474	Dnajc3	1
GO:0045109	intermediate filament organization	1/138	23/14464	0.04347826	4.55702583	1.67555453	0.19801357	0.63208191	0.62079474	Pkp2	1
GO:0050857	positive regulation of antigen receptor-mediated signaling pathway	1/138	23/14464	0.04347826	4.55702583	1.67555453	0.19801357	0.63208191	0.62079474	Card11	1
GO:0086005	ventricular cardiac muscle cell action potential	1/138	23/14464	0.04347826	4.55702583	1.67555453	0.19801357	0.63208191	0.62079474	Pkp2	1
GO:0098901	regulation of cardiac muscle cell action potential	1/138	23/14464	0.04347826	4.55702583	1.67555453	0.19801357	0.63208191	0.62079474	Pkp2	1
GO:1903319	positive regulation of protein maturation	1/138	23/14464	0.04347826	4.55702583	1.67555453	0.19801357	0.63208191	0.62079474	Mmp14	1
GO:0006672	ceramide metabolic process	2/138	86/14464	0.02325581	2.43747893	1.31222328	0.19808139	0.63208191	0.62079474	Hexa/Degs1	2
GO:0070252	actin-mediated cell contraction	2/138	86/14464	0.02325581	2.43747893	1.31222328	0.19808139	0.63208191	0.62079474	Pkp2/Sri	2
GO:1903509	liposaccharide metabolic process	2/138	86/14464	0.02325581	2.43747893	1.31222328	0.19808139	0.63208191	0.62079474	Pigg/Hexa	2
GO:0009266	response to temperature stimulus	3/138	161/14464	0.01863354	1.95301107	1.19344821	0.19901714	0.63208191	0.62079474	Chordc1/Hspd1/Dnajc3	3
GO:0003205	cardiac chamber development	3/138	162/14464	0.01851852	1.94095545	1.18204618	0.20146075	0.63208191	0.62079474	Dhrs3/Dand5/Pkp2	3
GO:0043487	regulation of RNA stability	3/138	162/14464	0.01851852	1.94095545	1.18204618	0.20146075	0.63208191	0.62079474	Mettl16/Hnrnpc/Mettl14	3
GO:0050779	RNA destabilization	2/138	87/14464	0.02298851	2.40946194	1.29415145	0.20155467	0.63208191	0.62079474	Mettl16/Mettl14	2
GO:0061014	positive regulation of mRNA catabolic process	2/138	87/14464	0.02298851	2.40946194	1.29415145	0.20155467	0.63208191	0.62079474	Mettl16/Mettl14	2
GO:0000209	protein polyubiquitination	4/138	243/14464	0.01646091	1.72529373	1.11907258	0.20253173	0.63208191	0.62079474	Sash1/Dtx4/Itch/Ube3c	4
GO:0070482	response to oxygen levels	5/138	327/14464	0.01529052	1.60262376	1.08180196	0.20273317	0.63208191	0.62079474	Mmp14/Hsp90b1/Cr1l/Pdk3/P4hb	5
GO:0030148	sphingolipid biosynthetic process	2/138	88/14464	0.02272727	2.38208169	1.27632795	0.2050351	0.63208191	0.62079474	Degs1/Elovl7	2
GO:0043406	positive regulation of MAP kinase activity	2/138	88/14464	0.02272727	2.38208169	1.27632795	0.2050351	0.63208191	0.62079474	Fzd8/Sash1	2
GO:0043954	cellular component maintenance	2/138	88/14464	0.02272727	2.38208169	1.27632795	0.2050351	0.63208191	0.62079474	Zfp804a/Pkp2	2
GO:0048704	embryonic skeletal system morphogenesis	2/138	88/14464	0.02272727	2.38208169	1.27632795	0.2050351	0.63208191	0.62079474	Mmp14/Smad2	2
GO:0001573	ganglioside metabolic process	1/138	24/14464	0.04166667	4.36714976	1.62028242	0.20567745	0.63208191	0.62079474	Hexa	1
GO:0008299	isoprenoid biosynthetic process	1/138	24/14464	0.04166667	4.36714976	1.62028242	0.20567745	0.63208191	0.62079474	Crppa	1
GO:0018126	protein hydroxylation	1/138	24/14464	0.04166667	4.36714976	1.62028242	0.20567745	0.63208191	0.62079474	P4hb	1
GO:0021522	spinal cord motor neuron differentiation	1/138	24/14464	0.04166667	4.36714976	1.62028242	0.20567745	0.63208191	0.62079474	Ift172	1
GO:0031664	regulation of lipopolysaccharide-mediated signaling pathway	1/138	24/14464	0.04166667	4.36714976	1.62028242	0.20567745	0.63208191	0.62079474	Sash1	1
GO:0035883	enteroendocrine cell differentiation	1/138	24/14464	0.04166667	4.36714976	1.62028242	0.20567745	0.63208191	0.62079474	Bmal1	1
GO:0040020	regulation of meiotic nuclear division	1/138	24/14464	0.04166667	4.36714976	1.62028242	0.20567745	0.63208191	0.62079474	Calr	1
GO:0043931	ossification involved in bone maturation	1/138	24/14464	0.04166667	4.36714976	1.62028242	0.20567745	0.63208191	0.62079474	Ift80	1
GO:0044804	nucleophagy	1/138	24/14464	0.04166667	4.36714976	1.62028242	0.20567745	0.63208191	0.62079474	Wdr45b	1
GO:0046473	phosphatidic acid metabolic process	1/138	24/14464	0.04166667	4.36714976	1.62028242	0.20567745	0.63208191	0.62079474	Dgkk	1
GO:0046580	negative regulation of Ras protein signal transduction	1/138	24/14464	0.04166667	4.36714976	1.62028242	0.20567745	0.63208191	0.62079474	Ppp2cb	1
GO:0048596	embryonic camera-type eye morphogenesis	1/138	24/14464	0.04166667	4.36714976	1.62028242	0.20567745	0.63208191	0.62079474	Ift172	1
GO:0051016	barbed-end actin filament capping	1/138	24/14464	0.04166667	4.36714976	1.62028242	0.20567745	0.63208191	0.62079474	Scin	1
GO:0062009	secondary palate development	1/138	24/14464	0.04166667	4.36714976	1.62028242	0.20567745	0.63208191	0.62079474	Smad2	1
GO:0070723	response to cholesterol	1/138	24/14464	0.04166667	4.36714976	1.62028242	0.20567745	0.63208191	0.62079474	Smad2	1
GO:0071542	dopaminergic neuron differentiation	1/138	24/14464	0.04166667	4.36714976	1.62028242	0.20567745	0.63208191	0.62079474	Manf	1
GO:0072530	purine-containing compound transmembrane transport	1/138	24/14464	0.04166667	4.36714976	1.62028242	0.20567745	0.63208191	0.62079474	Slc33a1	1
GO:0097062	dendritic spine maintenance	1/138	24/14464	0.04166667	4.36714976	1.62028242	0.20567745	0.63208191	0.62079474	Zfp804a	1
GO:1901983	regulation of protein acetylation	1/138	24/14464	0.04166667	4.36714976	1.62028242	0.20567745	0.63208191	0.62079474	Bmal1	1
GO:1902176	negative regulation of oxidative stress-induced intrinsic apoptotic signaling pathway	1/138	24/14464	0.04166667	4.36714976	1.62028242	0.20567745	0.63208191	0.62079474	Bag5	1
GO:1903523	negative regulation of blood circulation	1/138	24/14464	0.04166667	4.36714976	1.62028242	0.20567745	0.63208191	0.62079474	Sri	1
GO:2001014	regulation of skeletal muscle cell differentiation	1/138	24/14464	0.04166667	4.36714976	1.62028242	0.20567745	0.63208191	0.62079474	Bmal1	1
GO:0050673	epithelial cell proliferation	6/138	417/14464	0.01438849	1.50808049	1.03327285	0.20838761	0.63696228	0.62558795	Mmp14/Has2/Ift172/S2bpcox16/Ift80/Aggf1	6
GO:0045913	positive regulation of carbohydrate metabolic process	2/138	89/14464	0.02247191	2.35531672	1.2587461	0.20852223	0.63696228	0.62558795	Has2/Arpp19	2
GO:0008016	regulation of heart contraction	3/138	165/14464	0.01818182	1.90566535	1.14832059	0.20883403	0.63696228	0.62558795	Kcnip2/Pkp2/Sri	3
GO:0071453	cellular response to oxygen levels	3/138	165/14464	0.01818182	1.90566535	1.14832059	0.20883403	0.63696228	0.62558795	Cr1l/Pdk3/P4hb	3
GO:0006650	glycerophospholipid metabolic process	4/138	247/14464	0.01619433	1.69735375	1.08493545	0.21044155	0.63696228	0.62558795	Rab38/Dgkk/Pigg/Selenoi	4
GO:0002027	regulation of heart rate	2/138	90/14464	0.02222222	2.32914654	1.24139949	0.21201562	0.63696228	0.62558795	Pkp2/Sri	2
GO:0007498	mesoderm development	2/138	90/14464	0.02222222	2.32914654	1.24139949	0.21201562	0.63696228	0.62558795	Dand5/Smad2	2

GO:0043535	regulation of blood vessel endothelial cell migration	2/138	90/14464	0.02222222	2.32914654	1.24139949	0.21201562	0.63696228	0.62558795	Rhoj/Stard13	2
GO:0046634	regulation of alpha-beta T cell activation	2/138	90/14464	0.02222222	2.32914654	1.24139949	0.21201562	0.63696228	0.62558795	Card11/Itch	2
GO:0003180	aortic valve morphogenesis	1/138	25/14464	0.04	4.19246377	1.56795533	0.21326862	0.63696228	0.62558795	Smad2	1
GO:0003416	endochondral bone growth	1/138	25/14464	0.04	4.19246377	1.56795533	0.21326862	0.63696228	0.62558795	Ift80	1
GO:0018904	ether metabolic process	1/138	25/14464	0.04	4.19246377	1.56795533	0.21326862	0.63696228	0.62558795	Selenoi	1
GO:0030325	adrenal gland development	1/138	25/14464	0.04	4.19246377	1.56795533	0.21326862	0.63696228	0.62558795	Smad2	1
GO:0031294	lymphocyte costimulation	1/138	25/14464	0.04	4.19246377	1.56795533	0.21326862	0.63696228	0.62558795	Card11	1
GO:0033280	response to vitamin D	1/138	25/14464	0.04	4.19246377	1.56795533	0.21326862	0.63696228	0.62558795	Pdia3	1
GO:0045070	positive regulation of viral genome replication	1/138	25/14464	0.04	4.19246377	1.56795533	0.21326862	0.63696228	0.62558795	Srpk1	1
GO:0046697	decidualization	1/138	25/14464	0.04	4.19246377	1.56795533	0.21326862	0.63696228	0.62558795	Tppp3	1
GO:0048311	mitochondrion distribution	1/138	25/14464	0.04	4.19246377	1.56795533	0.21326862	0.63696228	0.62558795	S2bpcox16	1
GO:0098810	neurotransmitter reuptake	1/138	25/14464	0.04	4.19246377	1.56795533	0.21326862	0.63696228	0.62558795	Slc17a8	1
GO:0090398	cellular senescence	2/138	91/14464	0.02197802	2.30355152	1.22428195	0.21551483	0.63952318	0.62810312	Bmal1/Calr	2
GO:2001257	regulation of cation channel activity	2/138	91/14464	0.02197802	2.30355152	1.22428195	0.21551483	0.63952318	0.62810312	Rrad/Sri	2
GO:0140053	mitochondrial gene expression	3/138	168/14464	0.01785714	1.87163561	1.11529203	0.21626749	0.63952318	0.62810312	Mrps6/Tfb1m/Yars2	3
GO:0009755	hormone-mediated signaling pathway	3/138	169/14464	0.01775148	1.86056084	1.10443234	0.21875791	0.63952318	0.62810312	Bmal1/Dhrs3/Calr	3
GO:0044344	cellular response to fibroblast growth factor stimulus	2/138	92/14464	0.02173913	2.27851292	1.20738755	0.21901945	0.63952318	0.62810312	Gclm/Ift80	2
GO:0048565	digestive tract development	2/138	92/14464	0.02173913	2.27851292	1.20738755	0.21901945	0.63952318	0.62810312	Smad2/Ift172	2
GO:0022409	positive regulation of cell-cell adhesion	4/138	252/14464	0.01587302	1.6636761	1.04312201	0.22045124	0.63952318	0.62810312	Card11/Has2/Hspd1/Pkp3	4
GO:0006026	aminoglycan catabolic process	1/138	26/14464	0.03846154	4.03121516	1.51829507	0.22078776	0.63952318	0.62810312	Hexa	1
GO:0006972	hyperosmotic response	1/138	26/14464	0.03846154	4.03121516	1.51829507	0.22078776	0.63952318	0.62810312	Oxsr1	1
GO:0009081	branched-chain amino acid metabolic process	1/138	26/14464	0.03846154	4.03121516	1.51829507	0.22078776	0.63952318	0.62810312	Hmgcll1	1
GO:0030149	sphingolipid catabolic process	1/138	26/14464	0.03846154	4.03121516	1.51829507	0.22078776	0.63952318	0.62810312	Hexa	1
GO:0045932	negative regulation of muscle contraction	1/138	26/14464	0.03846154	4.03121516	1.51829507	0.22078776	0.63952318	0.62810312	Sri	1
GO:0046339	diacylglycerol metabolic process	1/138	26/14464	0.03846154	4.03121516	1.51829507	0.22078776	0.63952318	0.62810312	Dgkk	1
GO:0060479	lung cell differentiation	1/138	26/14464	0.03846154	4.03121516	1.51829507	0.22078776	0.63952318	0.62810312	Lta4h	1
GO:0060487	lung epithelial cell differentiation	1/138	26/14464	0.03846154	4.03121516	1.51829507	0.22078776	0.63952318	0.62810312	Lta4h	1
GO:0061436	establishment of skin barrier	1/138	26/14464	0.03846154	4.03121516	1.51829507	0.22078776	0.63952318	0.62810312	Grhl3	1
GO:0070977	bone maturation	1/138	26/14464	0.03846154	4.03121516	1.51829507	0.22078776	0.63952318	0.62810312	Ift80	1
GO:1900101	regulation of endoplasmic reticulum unfolded protein response	1/138	26/14464	0.03846154	4.03121516	1.51829507	0.22078776	0.63952318	0.62810312	Hspa5	1
GO:1900745	positive regulation of p38MAPK cascade	1/138	26/14464	0.03846154	4.03121516	1.51829507	0.22078776	0.63952318	0.62810312	Sash1	1
GO:1904357	negative regulation of telomere maintenance via telomere lengthening	1/138	26/14464	0.03846154	4.03121516	1.51829507	0.22078776	0.63952318	0.62810312	Hnrnpc	1
GO:1905523	positive regulation of macrophage migration	1/138	26/14464	0.03846154	4.03121516	1.51829507	0.22078776	0.63952318	0.62810312	Mmp14	1
GO:0010631	epithelial cell migration	2/138	93/14464	0.02150538	2.25401278	1.19071056	0.22252905	0.64173346	0.63027393	Has2/Sash1	2
GO:0046364	monosaccharide biosynthetic process	2/138	93/14464	0.02150538	2.25401278	1.19071056	0.22252905	0.64173346	0.63027393	Arpp19/Akr1a1	2
GO:0090132	epithelium migration	2/138	93/14464	0.02150538	2.25401278	1.19071056	0.22252905	0.64173346	0.63027393	Has2/Sash1	2
GO:0042552	myelination	3/138	171/14464	0.01754386	1.8387999	1.08293151	0.22375666	0.64173346	0.63027393	Hexa/Selenoi/Degs1	3
GO:0048562	embryonic organ morphogenesis	4/138	254/14464	0.01574803	1.65057629	1.02665449	0.22449102	0.64173346	0.63027393	Mmp14/Grhl3/Smad2/Ift172	4
GO:0001843	neural tube closure	2/138	94/14464	0.0212766	2.23003392	1.17424547	0.22604323	0.64173346	0.63027393	Grhl3/Ift172	2
GO:0010717	regulation of epithelial to mesenchymal transition	2/138	94/14464	0.0212766	2.23003392	1.17424547	0.22604323	0.64173346	0.63027393	Smad2/Spred3	2
GO:0060606	tube closure	2/138	94/14464	0.0212766	2.23003392	1.17424547	0.22604323	0.64173346	0.63027393	Grhl3/Ift172	2
GO:0006536	glutamate metabolic process	1/138	27/14464	0.03703704	3.8819109	1.47105942	0.22823556	0.64173346	0.63027393	Gclm	1
GO:0007608	sensory perception of smell	1/138	27/14464	0.03703704	3.8819109	1.47105942	0.22823556	0.64173346	0.63027393	Ric8b	1
GO:0030970	retrograde protein transport, ER to cytosol	1/138	27/14464	0.03703704	3.8819109	1.47105942	0.22823556	0.64173346	0.63027393	Hsp90b1	1
GO:0032647	regulation of interferon-alpha production	1/138	27/14464	0.03703704	3.8819109	1.47105942	0.22823556	0.64173346	0.63027393	Hspd1	1
GO:0032801	receptor catabolic process	1/138	27/14464	0.03703704	3.8819109	1.47105942	0.22823556	0.64173346	0.63027393	Itch	1
GO:0036120	cellular response to platelet-derived growth factor stimulus	1/138	27/14464	0.03703704	3.8819109	1.47105942	0.22823556	0.64173346	0.63027393	Has2	1
GO:0042572	retinol metabolic process	1/138	27/14464	0.03703704	3.8819109	1.47105942	0.22823556	0.64173346	0.63027393	Dhrs3	1
GO:0044090	positive regulation of vacuole organization	1/138	27/14464	0.03703704	3.8819109	1.47105942	0.22823556	0.64173346	0.63027393	Snx18	1
GO:0045722	positive regulation of gluconeogenesis	1/138	27/14464	0.03703704	3.8819109	1.47105942	0.22823556	0.64173346	0.63027393	Arpp19	1
GO:0051957	positive regulation of amino acid transport	1/138	27/14464	0.03703704	3.8819109	1.47105942	0.22823556	0.64173346	0.63027393	Slc17a8	1
GO:0070841	inclusion body assembly	1/138	27/14464	0.03703704	3.8819109	1.47105942	0.22823556	0.64173346	0.63027393	Bag5	1
GO:1903513	endoplasmic reticulum to cytosol transport	1/138	27/14464	0.03703704	3.8819109	1.47105942	0.22823556	0.64173346	0.63027393	Hsp90b1	1
GO:2000050	regulation of non-canonical Wnt signaling pathway	1/138	27/14464	0.03703704	3.8819109	1.47105942	0.22823556	0.64173346	0.63027393	Ift80	1
GO:0019882	antigen processing and presentation	2/138	95/14464	0.02105263	2.20655988	1.15798697	0.22956158	0.64456287	0.63305282	Calr/Pdia3	2
GO:0007272	ensheathment of neurons	3/138	174/14464	0.01724138	1.80709645	1.05121176	0.23129712	0.64625193	0.63471171	Hexa/Selenoi/Degs1	3
GO:0008366	axon ensheathment	3/138	174/14464	0.01724138	1.80709645	1.05121176	0.23129712	0.64625193	0.63471171	Hexa/Selenoi/Degs1	3
GO:0001704	formation of primary germ layer	2/138	96/14464	0.02083333	2.18357488	1.14192994	0.2330837	0.64625193	0.63471171	Mmp14/Smad2	2
GO:0006022	aminoglycan metabolic process	2/138	96/14464	0.02083333	2.18357488	1.14192994	0.2330837	0.64625193	0.63471171	Has2/Hexa	2
GO:0050764	regulation of phagocytosis	2/138	96/14464	0.02083333	2.18357488	1.14192994	0.2330837	0.64625193	0.63471171	Calr/Lman2	2
GO:0050678	regulation of epithelial cell proliferation	5/138	346/14464	0.01445087	1.51461841	0.95091795	0.2354202	0.64625193	0.63471171	Has2/Ift172/S2bpcox16/Ift80/Aggf1	5
GO:0000038	very long-chain fatty acid metabolic process	1/138	28/14464	0.03571429	3.74327122	1.42603633	0.23561268	0.64625193	0.63471171	Elovl7	1
GO:0002068	glandular epithelial cell development	1/138	28/14464	0.03571429	3.74327122	1.42603633	0.23561268	0.64625193	0.63471171	Bmal1	1
GO:0003230	cardiac atrium development	1/138	28/14464	0.03571429	3.74327122	1.42603633	0.23561268	0.64625193	0.63471171	Dand5	1
GO:0006221	pyrimidine nucleotide biosynthetic process	1/138	28/14464	0.03571429	3.74327122	1.42603633	0.23561268	0.64625193	0.63471171	Tbpl1	1
GO:0032735	positive regulation of interleukin-12 production	1/138	28/14464	0.03571429	3.74327122	1.42603633	0.23561268	0.64625193	0.63471171	Hspd1	1
GO:0034698	response to gonadotropin	1/138	28/14464	0.03571429	3.74327122	1.42603633	0.23561268	0.64625193	0.63471171	Gclm	1
GO:0036314	response to sterol	1/138	28/14464	0.03571429	3.74327122	1.42603633	0.23561268	0.64625193	0.63471171	Smad2	1
GO:0048799	animal organ maturation	1/138	28/14464	0.03571429	3.74327122	1.42603633	0.23561268	0.64625193	0.63471171	Ift80	1
GO:0060317	cardiac epithelial to mesenchymal transition	1/138	28/14464	0.03571429	3.74327122	1.42603633	0.23561268	0.64625193	0.63471171	Has2	1
GO:0086091	regulation of heart rate by cardiac conduction	1/138	28/14464	0.03571429	3.74327122	1.42603633	0.23561268	0.64625193	0.63471171	Pkp2	1
GO:1902914	regulation of protein polyubiquitination	1/138	28/14464	0.03571429	3.74327122	1.42603633	0.23561268	0.64625193	0.63471171	Sash1	1
GO:0045216	cell-cell junction organization	3/138	176/14464	0.01704545	1.78656126	1.0304085	0.23635045	0.6463471	0.63480519	Pkp3/Pkp2/Cdh10	3
GO:0043266	regulation of potassium ion transport	2/138	97/14464	0.02061856	2.1610638	1.12606943	0.23660921	0.6463471	0.63480519	Kcnip2/Oxsr1	2
GO:0090130	tissue migration	2/138	97/14464	0.02061856	2.1610638	1.12606943	0.23660921	0.6463471	0.63480519	Has2/Sash1	2
GO:0022618	protein-RNA complex assembly	3/138	177/14464	0.01694915	1.7764677	1.02010709	0.23888454	0.64778611	0.6362185	Srpk1/Prpf31/Prpf18	3
GO:0032412	regulation of monoatomic ion transmembrane transporter activity	3/138	177/14464	0.01694915	1.7764677	1.02010709	0.23888454	0.64778611	0.6362185	Rrad/Sri/Oxsr1	3
GO:0003014	renal system process	2/138	98/14464	0.02040816	2.13901213	1.11040068	0.24013772	0.64778611	0.6362185	Has2/Oxsr1	2
GO:0007040	lysosome organization	2/138	98/14464	0.02040816	2.13901213	1.11040068	0.24013772	0.64778611	0.6362185	Hexa/Aktip	2
GO:0071774	response to fibroblast growth factor	2/138	98/14464	0.02040816	2.13901213	1.11040068	0.24013772	0.64778611	0.6362185	Gclm/Ift80	2
GO:0080171	lytic vacuole organization	2/138	98/14464	0.02040816	2.13901213	1.11040068	0.24013772	0.64778611	0.6362185	Hexa/Aktip	2
GO:0002920	regulation of humoral immune response	1/138	29/14464	0.03448276	3.6141929	1.38303923	0.24291979	0.64778611	0.6362185	Cr1l	1
GO:0006506	GPI anchor biosynthetic process	1/138	29/14464	0.03448276	3.6141929	1.38303923	0.24291979	0.64778611	0.6362185	Pigg	1
GO:0015813	L-glutamate transmembrane transport	1/138	29/14464	0.03448276	3.6141929	1.38303923	0.24291979	0.64778611	0.6362185	Slc17a8	1
GO:0016339	calcium-dependent cell-cell adhesion via plasma membrane cell adhesion molecules	1/138	29/14464	0.03448276	3.6141929	1.38303923	0.24291979	0.64778611	0.6362185	Cdh10	1
GO:0032733	positive regulation of interleukin-10 production	1/138	29/14464	0.03448276	3.6141929	1.38303923	0.24291979	0.64778611	0.6362185	Hspd1	1
GO:0043276	anoikis	1/138	29/14464	0.03448276	3.6141929	1.38303923	0.24291979	0.64778611	0.6362185	Brms1	1
GO:0045048	protein insertion into ER membrane	1/138	29/14464	0.03448276	3.6141929	1.38303923	0.24291979	0.64778611	0.6362185	Ubl4a	1
GO:0046466	membrane lipid catabolic process	1/138	29/14464	0.03448276	3.6141929	1.38303923	0.24291979	0.64778611	0.6362185	Hexa	1
GO:0048247	lymphocyte chemotaxis	1/138	29/14464	0.03448276	3.6141929	1.38303923	0.24291979	0.64778611	0.6362185	Oxsr1	1
GO:0051882	mitochondrial depolarization	1/138	29/14464	0.03448276	3.6141929	1.38303923	0.24291979	0.64778611	0.6362185	Gclm	1
GO:0090382	phagosome maturation	1/138	29/14464	0.03448276	3.6141929	1.38303923	0.24291979	0.64778611	0.6362185	Rab38	1
GO:0098868	bone growth	1/138	29/14464	0.03448276	3.6141929	1.38303923	0.24291979	0.64778611	0.6362185	Ift80	1
GO:0014020	primary neural tube formation	2/138	99/14464	0.02020202	2.11740594	1.09491908	0.24366886	0.64892525	0.6373373	Grhl3/Ift172	2
GO:0001654	eye development	5/138	351/14464	0.01424501	1.49304265	0.91777166	0.24426501	0.64965471	0.63805373	Rhoj/Grhl3/Slc17a8/Ift172/Spred3	5
GO:0042593	glucose homeostasis	4/138	265/14464	0.01509434	1.5820618	0.93857594	0.24704003	0.65140066	0.6397685	Smad2/Gclm/Sri/Pdk3	4
GO:0035270	endocrine system development	2/138	100/14464	0.02	2.09623188	1.07962018	0.24720226	0.65140066	0.6397685	Bmal1/Smad2	2
GO:0033500	carbohydrate homeostasis	4/138	266/14464	0.01503759	1.5761142	0.93076935	0.24911529	0.65140066	0.6397685	Smad2/Gclm/Sri/Pdk3	4
GO:0150063	visual system development	5/138	354/14464	0.01412429	1.48038975	0.89812776	0.24961484	0.65140066	0.6397685	Rhoj/Grhl3/Slc17a8/Ift172/Spred3	5
GO:0003176	aortic valve development	1/138	30/14464	0.03333333	3.49371981	1.34190322	0.25015755	0.65140066	0.6397685	Smad2	1
GO:0006505	GPI anchor metabolic process	1/138	30/14464	0.03333333	3.49371981	1.34190322	0.25015755	0.65140066	0.6397685	Pigg	1
GO:0006891	intra-Golgi vesicle-mediated transport	1/138	30/14464	0.03333333	3.49371981	1.34190322	0.25015755	0.65140066	0.6397685	Cog6	1
GO:0021515	cell differentiation in spinal cord	1/138	30/14464	0.03333333	3.49371981	1.34190322	0.25015755	0.65140066	0.6397685	Ift172	1
GO:0032607	interferon-alpha production	1/138	30/14464	0.03333333	3.49371981	1.34190322	0.25015755	0.65140066	0.6397685	Hspd1	1
GO:0033198	response to ATP	1/138	30/14464	0.03333333	3.49371981	1.34190322	0.25015755	0.65140066	0.6397685	Hsp90b1	1
GO:0036119	response to platelet-derived growth factor	1/138	30/14464	0.03333333	3.49371981	1.34190322	0.25015755	0.65140066	0.6397685	Has2	1
GO:0048026	positive regulation of mRNA splicing, via spliceosome	1/138	30/14464	0.03333333	3.49371981	1.34190322	0.25015755	0.65140066	0.6397685	Obi1	1
GO:0048048	embryonic eye morphogenesis	1/138	30/14464	0.03333333	3.49371981	1.34190322	0.25015755	0.65140066	0.6397685	Ift172	1
GO:0051590	positive regulation of neurotransmitter transport	1/138	30/14464	0.03333333	3.49371981	1.34190322	0.25015755	0.65140066	0.6397685	Slc17a8	1
GO:0071353	cellular response to interleukin-4	1/138	30/14464	0.03333333	3.49371981	1.34190322	0.25015755	0.65140066	0.6397685	Hspa5	1
GO:0002688	regulation of leukocyte chemotaxis	2/138	101/14464	0.01980198	2.07547711	1.0644997	0.25073755	0.65140066	0.6397685	Calr/Oxsr1	2
GO:0035710	CD4-positive, alpha-beta T cell activation	2/138	101/14464	0.01980198	2.07547711	1.0644997	0.25073755	0.65140066	0.6397685	Card11/Itch	2
GO:0043255	regulation of carbohydrate biosynthetic process	2/138	101/14464	0.01980198	2.07547711	1.0644997	0.25073755	0.65140066	0.6397685	Has2/Arpp19	2
GO:0006401	RNA catabolic process	4/138	268/14464	0.01492537	1.56435215	0.91525234	0.25327732	0.65377341	0.64209888	Mettl16/Hnrnpc/Pkp3/Mettl14	4
GO:0006109	regulation of carbohydrate metabolic process	3/138	183/14464	0.01639344	1.71822286	0.95964746	0.25418324	0.65377341	0.64209888	Has2/Pdk3/Arpp19	3
GO:0016051	carbohydrate biosynthetic process	3/138	183/14464	0.01639344	1.71822286	0.95964746	0.25418324	0.65377341	0.64209888	Has2/Arpp19/Akr1a1	3
GO:0000578	embryonic axis specification	1/138	31/14464	0.03225806	3.38101917	1.3024819	0.25732662	0.65377341	0.64209888	Smad2	1
GO:0001736	establishment of planar polarity	1/138	31/14464	0.03225806	3.38101917	1.3024819	0.25732662	0.65377341	0.64209888	Grhl3	1
GO:0005980	glycogen catabolic process	1/138	31/14464	0.03225806	3.38101917	1.3024819	0.25732662	0.65377341	0.64209888	Wdr45b	1
GO:0007164	establishment of tissue polarity	1/138	31/14464	0.03225806	3.38101917	1.3024819	0.25732662	0.65377341	0.64209888	Grhl3	1
GO:0009251	glucan catabolic process	1/138	31/14464	0.03225806	3.38101917	1.3024819	0.25732662	0.65377341	0.64209888	Wdr45b	1
GO:0030513	positive regulation of BMP signaling pathway	1/138	31/14464	0.03225806	3.38101917	1.3024819	0.25732662	0.65377341	0.64209888	Smad2	1
GO:0030574	collagen catabolic process	1/138	31/14464	0.03225806	3.38101917	1.3024819	0.25732662	0.65377341	0.64209888	Mmp14	1
GO:0033687	osteoblast proliferation	1/138	31/14464	0.03225806	3.38101917	1.3024819	0.25732662	0.65377341	0.64209888	Ift80	1
GO:0034405	response to fluid shear stress	1/138	31/14464	0.03225806	3.38101917	1.3024819	0.25732662	0.65377341	0.64209888	Has2	1
GO:0043267	negative regulation of potassium ion transport	1/138	31/14464	0.03225806	3.38101917	1.3024819	0.25732662	0.65377341	0.64209888	Oxsr1	1
GO:0065002	intracellular protein transmembrane transport	1/138	31/14464	0.03225806	3.38101917	1.3024819	0.25732662	0.65377341	0.64209888	Hspa5	1
GO:0090311	regulation of protein deacetylation	1/138	31/14464	0.03225806	3.38101917	1.3024819	0.25732662	0.65377341	0.64209888	Brms1	1
GO:2000279	negative regulation of DNA biosynthetic process	1/138	31/14464	0.03225806	3.38101917	1.3024819	0.25732662	0.65377341	0.64209888	Hnrnpc	1
GO:2000515	negative regulation of CD4-positive, alpha-beta T cell activation	1/138	31/14464	0.03225806	3.38101917	1.3024819	0.25732662	0.65377341	0.64209888	Itch	1
GO:0042303	molting cycle	2/138	103/14464	0.01941748	2.03517659	1.03477749	0.25781243	0.65377341	0.64209888	Bmal1/Pkp3	2
GO:0042633	hair cycle	2/138	103/14464	0.01941748	2.03517659	1.03477749	0.25781243	0.65377341	0.64209888	Bmal1/Pkp3	2
GO:0048880	sensory system development	5/138	359/14464	0.01392758	1.45977151	0.86578288	0.25859823	0.65494225	0.64324685	Rhoj/Grhl3/Slc17a8/Ift172/Spred3	5
GO:0006814	sodium ion transport	3/138	185/14464	0.01621622	1.69964747	0.93998841	0.25931499	0.65593353	0.64422043	Slc5a3/Slc17a8/Oxsr1	3
GO:0055123	digestive system development	2/138	104/14464	0.01923077	2.01560758	1.02016788	0.26135132	0.65604385	0.64432879	Smad2/Ift172	2
GO:0022898	regulation of transmembrane transporter activity	3/138	186/14464	0.01612903	1.69050958	0.93024803	0.26188623	0.65604385	0.64432879	Rrad/Sri/Oxsr1	3
GO:0071826	protein-RNA complex organization	3/138	186/14464	0.01612903	1.69050958	0.93024803	0.26188623	0.65604385	0.64432879	Srpk1/Prpf31/Prpf18	3
GO:0007600	sensory perception	5/138	362/14464	0.01381215	1.44767395	0.84660732	0.26402616	0.65604385	0.64432879	Rbp1/Slc17a8/Hexa/Smr2l/Ric8b	5
GO:0002347	response to tumor cell	1/138	32/14464	0.03125	3.27536232	1.26464477	0.26442763	0.65604385	0.64432879	Hspd1	1
GO:0009309	amine biosynthetic process	1/138	32/14464	0.03125	3.27536232	1.26464477	0.26442763	0.65604385	0.64432879	Sat1	1
GO:0032743	positive regulation of interleukin-2 production	1/138	32/14464	0.03125	3.27536232	1.26464477	0.26442763	0.65604385	0.64432879	Card11	1
GO:0042401	biogenic amine biosynthetic process	1/138	32/14464	0.03125	3.27536232	1.26464477	0.26442763	0.65604385	0.64432879	Sat1	1
GO:0043537	negative regulation of blood vessel endothelial cell migration	1/138	32/14464	0.03125	3.27536232	1.26464477	0.26442763	0.65604385	0.64432879	Stard13	1
GO:0180046	GPI anchored protein biosynthesis	1/138	32/14464	0.03125	3.27536232	1.26464477	0.26442763	0.65604385	0.64432879	Pigg	1
GO:1901381	positive regulation of potassium ion transmembrane transport	1/138	32/14464	0.03125	3.27536232	1.26464477	0.26442763	0.65604385	0.64432879	Kcnip2	1
GO:2000677	regulation of transcription regulatory region DNA binding	1/138	32/14464	0.03125	3.27536232	1.26464477	0.26442763	0.65604385	0.64432879	Sri	1
GO:0002377	immunoglobulin production	2/138	105/14464	0.01904762	1.99641132	1.00572088	0.26489072	0.65604385	0.64432879	Card11/Hspd1	2
GO:0002832	negative regulation of response to biotic stimulus	2/138	105/14464	0.01904762	1.99641132	1.00572088	0.26489072	0.65604385	0.64432879	Dtx4/Itch	2
GO:0003279	cardiac septum development	2/138	105/14464	0.01904762	1.99641132	1.00572088	0.26489072	0.65604385	0.64432879	Dhrs3/Dand5	2
GO:0010906	regulation of glucose metabolic process	2/138	105/14464	0.01904762	1.99641132	1.00572088	0.26489072	0.65604385	0.64432879	Pdk3/Arpp19	2
GO:0038061	non-canonical NF-kappaB signal transduction	2/138	105/14464	0.01904762	1.99641132	1.00572088	0.26489072	0.65604385	0.64432879	Calr/Sash1	2
GO:0043524	negative regulation of neuron apoptotic process	3/138	188/14464	0.01595745	1.67252544	0.91094181	0.26703866	0.65720011	0.64546439	Hspd1/Gclm/Sema3e	3
GO:0010817	regulation of hormone levels	6/138	455/14464	0.01318681	1.38213091	0.81286901	0.26784113	0.65720011	0.64546439	Bmal1/Dhrs3/Hsp90b1/Smad2/Sri/P4hb	6
GO:0048024	regulation of mRNA splicing, via spliceosome	2/138	106/14464	0.01886792	1.97757725	0.99143285	0.26843032	0.65720011	0.64546439	Mettl16/Obi1	2
GO:0003203	endocardial cushion morphogenesis	1/138	33/14464	0.03030303	3.17610892	1.2282751	0.27146123	0.65720011	0.64546439	Smad2	1
GO:0010719	negative regulation of epithelial to mesenchymal transition	1/138	33/14464	0.03030303	3.17610892	1.2282751	0.27146123	0.65720011	0.64546439	Spred3	1
GO:0019731	antibacterial humoral response	1/138	33/14464	0.03030303	3.17610892	1.2282751	0.27146123	0.65720011	0.64546439	Ighg2c	1
GO:0031018	endocrine pancreas development	1/138	33/14464	0.03030303	3.17610892	1.2282751	0.27146123	0.65720011	0.64546439	Bmal1	1
GO:0032781	positive regulation of ATP-dependent activity	1/138	33/14464	0.03030303	3.17610892	1.2282751	0.27146123	0.65720011	0.64546439	Dnajb11	1
GO:0045943	positive regulation of transcription by RNA polymerase I	1/138	33/14464	0.03030303	3.17610892	1.2282751	0.27146123	0.65720011	0.64546439	Rasl11a	1
GO:0046456	icosanoid biosynthetic process	1/138	33/14464	0.03030303	3.17610892	1.2282751	0.27146123	0.65720011	0.64546439	Lta4h	1
GO:0050685	positive regulation of mRNA processing	1/138	33/14464	0.03030303	3.17610892	1.2282751	0.27146123	0.65720011	0.64546439	Obi1	1
GO:0060351	cartilage development involved in endochondral bone morphogenesis	1/138	33/14464	0.03030303	3.17610892	1.2282751	0.27146123	0.65720011	0.64546439	Ift80	1
GO:0070670	response to interleukin-4	1/138	33/14464	0.03030303	3.17610892	1.2282751	0.27146123	0.65720011	0.64546439	Hspa5	1
GO:0090075	relaxation of muscle	1/138	33/14464	0.03030303	3.17610892	1.2282751	0.27146123	0.65720011	0.64546439	Sri	1
GO:1903725	regulation of phospholipid metabolic process	1/138	33/14464	0.03030303	3.17610892	1.2282751	0.27146123	0.65720011	0.64546439	Rab38	1

GO:2000516 positive regulation of CD4-positive, alpha-beta T cell activation	1/138	33/14464	0.03030303	3.17610892	1.2282751	0.27146123	0.65720011	0.64546439	Card11	1
GO:0002062 chondrocyte differentiation	2/138	107/14464	0.01869159	1.95909522	0.97730029	0.27196977	0.65720011	0.64546439	Scin/Ift80	2
GO:0002456 T cell mediated immunity	2/138	107/14464	0.01869159	1.95909522	0.97730029	0.27196977	0.65720011	0.64546439	Hspd1/Cr1l	2
GO:0005976 polysaccharide metabolic process	2/138	107/14464	0.01869159	1.95909522	0.97730029	0.27196977	0.65720011	0.64546439	Has2/Wdr45b	2
GO:0030518 nuclear receptor-mediated steroid hormone signaling pathway	2/138	107/14464	0.01869159	1.95909522	0.97730029	0.27196977	0.65720011	0.64546439	Bmal1/Calr	2
GO:0032259 methylation	3/138	190/14464	0.01578947	1.65491991	0.89186354	0.27220342	0.65720011	0.64546439	Mettl16/Tfb1m/Mettl14	3
GO:0006605 protein targeting	4/138	278/14464	0.01438849	1.50808049	0.8395203	0.27429608	0.66146041	0.64964862	Hspa5/Hspd1/Ubl4a/Srprb	4
GO:0032970 regulation of actin filament-based process	5/138	368/14464	0.01358696	1.42407057	0.8087613	0.2749607	0.66192333	0.65010327	Arhgef16/Grhl3/Scin/Pkp2/Sri	5
GO:0030316 osteoclast differentiation	2/138	108/14464	0.01851852	1.94095545	0.96331978	0.27550877	0.66192333	0.65010327	Sh3pxd2a/Ift80	2
GO:0000272 polysaccharide catabolic process	1/138	34/14464	0.02941176	3.08269395	1.19326805	0.27842807	0.66192333	0.65010327	Wdr45b	1
GO:0001893 maternal placenta development	1/138	34/14464	0.02941176	3.08269395	1.19326805	0.27842807	0.66192333	0.65010327	Tppp3	1
GO:0006884 cell volume homeostasis	1/138	34/14464	0.02941176	3.08269395	1.19326805	0.27842807	0.66192333	0.65010327	Oxsr1	1
GO:0010043 response to zinc ion	1/138	34/14464	0.02941176	3.08269395	1.19326805	0.27842807	0.66192333	0.65010327	Lta4h	1
GO:0021517 ventral spinal cord development	1/138	34/14464	0.02941176	3.08269395	1.19326805	0.27842807	0.66192333	0.65010327	Ift172	1
GO:0035987 endodermal cell differentiation	1/138	34/14464	0.02941176	3.08269395	1.19326805	0.27842807	0.66192333	0.65010327	Mmp14	1
GO:0051125 regulation of actin nucleation	1/138	34/14464	0.02941176	3.08269395	1.19326805	0.27842807	0.66192333	0.65010327	Scin	1
GO:0070306 lens fiber cell differentiation	1/138	34/14464	0.02941176	3.08269395	1.19326805	0.27842807	0.66192333	0.65010327	Spred3	1
GO:0071398 cellular response to fatty acid	1/138	34/14464	0.02941176	3.08269395	1.19326805	0.27842807	0.66192333	0.65010327	Pdk3	1
GO:0086002 cardiac muscle cell action potential involved in contraction	1/138	34/14464	0.02941176	3.08269395	1.19326805	0.27842807	0.66192333	0.65010327	Pkp2	1
GO:0030324 lung development	3/138	194/14464	0.01546392	1.62079785	0.85436873	0.28256528	0.66499742	0.65312247	Mmp14/Smad2/Lta4h	3
GO:0030048 actin filament-based movement	2/138	110/14464	0.01818182	1.90566535	0.93580182	0.28258418	0.66499742	0.65312247	Pkp2/Sri	2
GO:0046545 development of primary female sexual characteristics	2/138	110/14464	0.01818182	1.90566535	0.93580182	0.28258418	0.66499742	0.65312247	Mmp14/Lhfpl2	2
GO:0000387 spliceosomal snRNP assembly	1/138	35/14464	0.02857143	2.99461698	1.15952917	0.28532875	0.66499742	0.65312247	Prpf31	1
GO:0016242 negative regulation of macroautophagy	1/138	35/14464	0.02857143	2.99461698	1.15952917	0.28532875	0.66499742	0.65312247	Sec22b	1
GO:0021680 cerebellar Purkinje cell layer development	1/138	35/14464	0.02857143	2.99461698	1.15952917	0.28532875	0.66499742	0.65312247	Hspa5	1
GO:0031076 embryonic camera-type eye development	1/138	35/14464	0.02857143	2.99461698	1.15952917	0.28532875	0.66499742	0.65312247	Ift172	1
GO:0032205 negative regulation of telomere maintenance	1/138	35/14464	0.02857143	2.99461698	1.15952917	0.28532875	0.66499742	0.65312247	Hnrnpc	1
GO:0032438 melanosome organization	1/138	35/14464	0.02857143	2.99461698	1.15952917	0.28532875	0.66499742	0.65312247	Rab38	1
GO:0033574 response to testosterone	1/138	35/14464	0.02857143	2.99461698	1.15952917	0.28532875	0.66499742	0.65312247	Calr	1
GO:0043392 negative regulation of DNA binding	1/138	35/14464	0.02857143	2.99461698	1.15952917	0.28532875	0.66499742	0.65312247	Sri	1
GO:0045746 negative regulation of Notch signaling pathway	1/138	35/14464	0.02857143	2.99461698	1.15952917	0.28532875	0.66499742	0.65312247	Mmp14	1
GO:0046717 acid secretion	1/138	35/14464	0.02857143	2.99461698	1.15952917	0.28532875	0.66499742	0.65312247	Slc51a	1
GO:0048546 digestive tract morphogenesis	1/138	35/14464	0.02857143	2.99461698	1.15952917	0.28532875	0.66499742	0.65312247	Smad2	1
GO:0051281 positive regulation of release of sequestered calcium ion into cytosol	1/138	35/14464	0.02857143	2.99461698	1.15952917	0.28532875	0.66499742	0.65312247	Sri	1
GO:0072528 pyrimidine-containing compound biosynthetic process	1/138	35/14464	0.02857143	2.99461698	1.15952917	0.28532875	0.66499742	0.65312247	Tbpl1	1
GO:1905314 semi-lunar valve development	1/138	35/14464	0.02857143	2.99461698	1.15952917	0.28532875	0.66499742	0.65312247	Smad2	1
GO:0031398 positive regulation of protein ubiquitination	2/138	111/14464	0.01801802	1.88849719	0.92225808	0.28611998	0.66607144	0.65417731	Hspa5/Ndfip2	2
GO:0048193 Golgi vesicle transport	4/138	284/14464	0.01408451	1.47621964	0.7954898	0.28705073	0.66746744	0.65554838	Lman2/Cog6/P4hb/Sec22b	4
GO:0051348 negative regulation of transferase activity	3/138	196/14464	0.01530612	1.60425909	0.83594161	0.28776012	0.66834609	0.65641133	Chordc1/Dnajc3/Bag5	3
GO:0001841 neural tube formation	2/138	112/14464	0.01785714	1.87163561	0.9088538	0.28965411	0.6691946	0.6572447	Grhl3/Ift172	2
GO:0030323 respiratory tube development	3/138	197/14464	0.01522843	1.59611565	0.8268059	0.29036053	0.6691946	0.6572447	Mmp14/Smad2/Lta4h	3
GO:1901293 nucleoside phosphate biosynthetic process	4/138	286/14464	0.01398601	1.46589642	0.78103621	0.29132232	0.6691946	0.6572447	Tbpl1/Ppcs/Elovl7/Pdk3	4
GO:0001523 retinoid metabolic process	1/138	36/14464	0.02777778	2.91143317	1.12697314	0.29216392	0.6691946	0.6572447	Dhrs3	1
GO:0009066 aspartate family amino acid metabolic process	1/138	36/14464	0.02777778	2.91143317	1.12697314	0.29216392	0.6691946	0.6572447	Enoph1	1
GO:0021879 forebrain neuron differentiation	1/138	36/14464	0.02777778	2.91143317	1.12697314	0.29216392	0.6691946	0.6572447	Sema3e	1
GO:0045879 negative regulation of smoothened signaling pathway	1/138	36/14464	0.02777778	2.91143317	1.12697314	0.29216392	0.6691946	0.6572447	Ift172	1
GO:0048753 pigment granule organization	1/138	36/14464	0.02777778	2.91143317	1.12697314	0.29216392	0.6691946	0.6572447	Rab38	1
GO:0050931 pigment cell differentiation	1/138	36/14464	0.02777778	2.91143317	1.12697314	0.29216392	0.6691946	0.6572447	Rab38	1
GO:0051693 actin filament capping	1/138	36/14464	0.02777778	2.91143317	1.12697314	0.29216392	0.6691946	0.6572447	Scin	1
GO:1901889 negative regulation of cell junction assembly	1/138	36/14464	0.02777778	2.91143317	1.12697314	0.29216392	0.6691946	0.6572447	Mmp14	1
GO:1902624 positive regulation of neutrophil migration	1/138	36/14464	0.02777778	2.91143317	1.12697314	0.29216392	0.6691946	0.6572447	Cd99l2	1
GO:0003013 circulatory system process	6/138	470/14464	0.01276596	1.33802035	0.73118472	0.2924407	0.6691946	0.6572447	Manf/Kcnip2/Gclm/Ppcs/Pkp2/Sri	6
GO:0001952 regulation of cell-matrix adhesion	2/138	114/14464	0.01754386	1.8387999	0.88245203	0.29671624	0.67744046	0.66534331	Mmp14/Sema3e	2
GO:0060048 cardiac muscle contraction	2/138	114/14464	0.01754386	1.8387999	0.88245203	0.29671624	0.67744046	0.66534331	Pkp2/Sri	2
GO:0001504 neurotransmitter uptake	1/138	37/14464	0.02702703	2.83274579	1.0955226	0.29893419	0.67942652	0.6672939	Slc17a8	1
GO:0042554 superoxide anion generation	1/138	37/14464	0.02702703	2.83274579	1.0955226	0.29893419	0.67942652	0.6672939	Sh3pxd2a	1
GO:0044743 protein transmembrane import into intracellular organelle	1/138	37/14464	0.02702703	2.83274579	1.0955226	0.29893419	0.67942652	0.6672939	Hspd1	1
GO:0046825 regulation of protein export from nucleus	1/138	37/14464	0.02702703	2.83274579	1.0955226	0.29893419	0.67942652	0.6672939	Frat2	1
GO:0141188 nucleic acid catabolic process	4/138	291/14464	0.0137457	1.4407092	0.74537355	0.30203918	0.68571057	0.67346574	Mettl16/Hnrnpc/Pkp3/Mettl14	4
GO:0043401 steroid hormone receptor signaling pathway	2/138	116/14464	0.01724138	1.80709645	0.85657424	0.30376832	0.68769036	0.67541017	Bmal1/Calr	2
GO:1903037 regulation of leukocyte cell-cell adhesion	4/138	292/14464	0.01369863	1.43577526	0.7383201	0.30418842	0.68769036	0.67541017	Card11/Has2/Hspd1/Itch	4
GO:0001569 branching involved in blood vessel morphogenesis	1/138	38/14464	0.02631579	2.75819985	1.06510724	0.30564016	0.68769036	0.67541017	Sema3e	1
GO:0002691 regulation of cellular extravasation	1/138	38/14464	0.02631579	2.75819985	1.06510724	0.30564016	0.68769036	0.67541017	Cd99l2	1
GO:0044273 sulfur compound catabolic process	1/138	38/14464	0.02631579	2.75819985	1.06510724	0.30564016	0.68769036	0.67541017	Hexa	1
GO:0044331 cell-cell adhesion mediated by cadherin	1/138	38/14464	0.02631579	2.75819985	1.06510724	0.30564016	0.68769036	0.67541017	Cdh10	1
GO:0046636 negative regulation of alpha-beta T cell activation	1/138	38/14464	0.02631579	2.75819985	1.06510724	0.30564016	0.68769036	0.67541017	Itch	1
GO:0051938 L-glutamate import	1/138	38/14464	0.02631579	2.75819985	1.06510724	0.30564016	0.68769036	0.67541017	Slc17a8	1
GO:0060047 heart contraction	3/138	203/14464	0.01477833	1.54893982	0.77304366	0.30599638	0.68772429	0.6754435	Kcnip2/Pkp2/Sri	3
GO:0003206 cardiac chamber morphogenesis	2/138	117/14464	0.01709402	1.79165118	0.84382524	0.30728992	0.68804526	0.67575874	Dhrs3/Pkp2	2
GO:0021549 cerebellum development	2/138	117/14464	0.01709402	1.79165118	0.84382524	0.30728992	0.68804526	0.67575874	Hspa5/Fktn	2
GO:0043534 blood vessel endothelial cell migration	2/138	117/14464	0.01709402	1.79165118	0.84382524	0.30728992	0.68804526	0.67575874	Rhoj/Stard13	2
GO:1904062 regulation of monoatomic cation transmembrane transport	4/138	294/14464	0.01360544	1.42600808	0.72429059	0.30849217	0.68804526	0.67575874	Rrad/Kcnip2/Sri/Oxsr1	4
GO:0016054 organic acid catabolic process	3/138	204/14464	0.01470588	1.54134697	0.76425325	0.30860667	0.68804526	0.67575874	Hexa/Hmgcll1/Akr1a1	3
GO:0046395 carboxylic acid catabolic process	3/138	204/14464	0.01470588	1.54134697	0.76425325	0.30860667	0.68804526	0.67575874	Hexa/Hmgcll1/Akr1a1	3
GO:0046546 development of primary male sexual characteristics	2/138	118/14464	0.01694915	1.7764677	0.83119945	0.31080821	0.68804526	0.67575874	Mmp14/Lhfpl2	2
GO:0098773 skin epidermis development	2/138	118/14464	0.01694915	1.7764677	0.83119945	0.31080821	0.68804526	0.67575874	Grhl3/Pkp3	2
GO:0010559 regulation of glycoprotein biosynthetic process	1/138	39/14464	0.02564103	2.68747677	1.035663	0.31228245	0.68804526	0.67575874	Fktn	1
GO:0010830 regulation of myotube differentiation	1/138	39/14464	0.02564103	2.68747677	1.035663	0.31228245	0.68804526	0.67575874	Mmp14	1
GO:0030890 positive regulation of B cell proliferation	1/138	39/14464	0.02564103	2.68747677	1.035663	0.31228245	0.68804526	0.67575874	Card11	1
GO:0032435 negative regulation of proteasomal ubiquitin-dependent protein catabolic process	1/138	39/14464	0.02564103	2.68747677	1.035663	0.31228245	0.68804526	0.67575874	Bag5	1
GO:0032885 regulation of polysaccharide biosynthetic process	1/138	39/14464	0.02564103	2.68747677	1.035663	0.31228245	0.68804526	0.67575874	Has2	1
GO:0043094 cellular metabolic compound salvage	1/138	39/14464	0.02564103	2.68747677	1.035663	0.31228245	0.68804526	0.67575874	Enoph1	1
GO:0046189 phenol-containing compound biosynthetic process	1/138	39/14464	0.02564103	2.68747677	1.035663	0.31228245	0.68804526	0.67575874	Rab38	1
GO:0099170 postsynaptic modulation of chemical synaptic transmission	1/138	39/14464	0.02564103	2.68747677	1.035663	0.31228245	0.68804526	0.67575874	Snx14	1
GO:1900744 regulation of p38MAPK cascade	1/138	39/14464	0.02564103	2.68747677	1.035663	0.31228245	0.68804526	0.67575874	Sash1	1
GO:1905521 regulation of macrophage migration	1/138	39/14464	0.02564103	2.68747677	1.035663	0.31228245	0.68804526	0.67575874	Mmp14	1
GO:0043087 regulation of GTPase activity	3/138	207/14464	0.01449275	1.51900861	0.73816241	0.31644238	0.69115161	0.67880962	Arhgef16/Grhl3/Snx18	3
GO:1903039 positive regulation of leukocyte cell-cell adhesion	3/138	207/14464	0.01449275	1.51900861	0.73816241	0.31644238	0.69115161	0.67880962	Card11/Has2/Hspd1	3
GO:0048706 embryonic skeletal system development	2/138	120/14464	0.01666667	1.7468599	0.80630786	0.31783383	0.69115161	0.67880962	Mmp14/Smad2	2
GO:1903900 regulation of viral life cycle	2/138	120/14464	0.01666667	1.7468599	0.80630786	0.31783383	0.69115161	0.67880962	Srpk1/P4hb	2
GO:0002823 negative regulation of adaptive immune response based on somatic recombination of immune receptors	1/138	40/14464	0.025	2.62028986	1.00713135	0.31886165	0.69115161	0.67880962	Cr1l	1
GO:0006220 pyrimidine nucleotide metabolic process	1/138	40/14464	0.025	2.62028986	1.00713135	0.31886165	0.69115161	0.67880962	Tbpl1	1
GO:0006636 unsaturated fatty acid biosynthetic process	1/138	40/14464	0.025	2.62028986	1.00713135	0.31886165	0.69115161	0.67880962	Elovl7	1
GO:0016101 diterpenoid metabolic process	1/138	40/14464	0.025	2.62028986	1.00713135	0.31886165	0.69115161	0.67880962	Dhrs3	1
GO:0042398 cellular modified amino acid biosynthetic process	1/138	40/14464	0.025	2.62028986	1.00713135	0.31886165	0.69115161	0.67880962	Gclm	1
GO:0045880 positive regulation of smoothened signaling pathway	1/138	40/14464	0.025	2.62028986	1.00713135	0.31886165	0.69115161	0.67880962	Ift172	1
GO:0051445 regulation of meiotic cell cycle	1/138	40/14464	0.025	2.62028986	1.00713135	0.31886165	0.69115161	0.67880962	Calr	1
GO:0051452 intracellular pH reduction	1/138	40/14464	0.025	2.62028986	1.00713135	0.31886165	0.69115161	0.67880962	Rab38	1
GO:0071711 basement membrane organization	1/138	40/14464	0.025	2.62028986	1.00713135	0.31886165	0.69115161	0.67880962	Fktn	1
GO:1901016 regulation of potassium ion transmembrane transporter activity	1/138	40/14464	0.025	2.62028986	1.00713135	0.31886165	0.69115161	0.67880962	Oxsr1	1
GO:1901532 regulation of hematopoietic progenitor cell differentiation	1/138	40/14464	0.025	2.62028986	1.00713135	0.31886165	0.69115161	0.67880962	Mettl14	1
GO:1903829 positive regulation of protein localization	6/138	486/14464	0.01234568	1.2939703	0.64700157	0.31917765	0.69115161	0.67880962	Slc5a3/Arhgef16/Rab38/Cnpy4/Pkp3/Sri	6
GO:0030509 BMP signaling pathway	2/138	121/14464	0.01652893	1.73242304	0.79403738	0.32134067	0.69312881	0.68075151	Dand5/Smad2	2
GO:0072329 monocarboxylic acid catabolic process	2/138	121/14464	0.01652893	1.73242304	0.79403738	0.32134067	0.69312881	0.68075151	Hexa/Akr1a1	2
GO:0070085 glycosylation	3/138	210/14464	0.01428571	1.49730849	0.71248119	0.32428267	0.69312881	0.68075151	Fktn/Cog6/Crppa	3
GO:0003231 cardiac ventricle development	2/138	122/14464	0.01639344	1.71822286	0.78188079	0.3248432	0.69312881	0.68075151	Dand5/Pkp2	2
GO:0046660 female sex differentiation	2/138	122/14464	0.01639344	1.71822286	0.78188079	0.3248432	0.69312881	0.68075151	Mmp14/Lhfpl2	2
GO:0002707 negative regulation of lymphocyte mediated immunity	1/138	41/14464	0.02439024	2.55638035	0.9794587	0.32537837	0.69312881	0.68075151	Cr1l	1
GO:0006418 tRNA aminoacylation for protein translation	1/138	41/14464	0.02439024	2.55638035	0.9794587	0.32537837	0.69312881	0.68075151	Yars2	1
GO:0009394 2'-deoxyribonucleotide metabolic process	1/138	41/14464	0.02439024	2.55638035	0.9794587	0.32537837	0.69312881	0.68075151	Tbpl1	1
GO:0030866 cortical actin cytoskeleton organization	1/138	41/14464	0.02439024	2.55638035	0.9794587	0.32537837	0.69312881	0.68075151	Calr	1
GO:0032527 protein exit from endoplasmic reticulum	1/138	41/14464	0.02439024	2.55638035	0.9794587	0.32537837	0.69312881	0.68075151	Hsp90b1	1
GO:0043268 positive regulation of potassium ion transport	1/138	41/14464	0.02439024	2.55638035	0.9794587	0.32537837	0.69312881	0.68075151	Kcnip2	1
GO:0045058 T cell selection	1/138	41/14464	0.02439024	2.55638035	0.9794587	0.32537837	0.69312881	0.68075151	Card11	1
GO:0045104 intermediate filament cytoskeleton organization	1/138	41/14464	0.02439024	2.55638035	0.9794587	0.32537837	0.69312881	0.68075151	Pkp2	1
GO:0046596 regulation of viral entry into host cell	1/138	41/14464	0.02439024	2.55638035	0.9794587	0.32537837	0.69312881	0.68075151	P4hb	1
GO:0051205 protein insertion into membrane	1/138	41/14464	0.02439024	2.55638035	0.9794587	0.32537837	0.69312881	0.68075151	Ubl4a	1
GO:0005975 carbohydrate metabolic process	6/138	491/14464	0.01221996	1.28079341	0.62128246	0.32761534	0.69312881	0.68075151	Has2/Hexa/Pdk3/Arpp19/Wdr45b/Akr1a1	6
GO:0006633 fatty acid biosynthetic process	2/138	123/14464	0.01626016	1.70425356	0.76983589	0.32834119	0.69312881	0.68075151	Degs1/Elovl7	2
GO:0050864 regulation of B cell activation	2/138	123/14464	0.01626016	1.70425356	0.76983589	0.32834119	0.69312881	0.68075151	Card11/Mmp14	2
GO:0050900 leukocyte migration	4/138	304/14464	0.01315789	1.37909992	0.65563952	0.33009645	0.69312881	0.68075151	Calr/Mmp14/Cd99l2/Oxsr1	4
GO:0022407 regulation of cell-cell adhesion	5/138	398/14464	0.01256281	1.31672857	0.62885386	0.33084366	0.69312881	0.68075151	Card11/Has2/Hspd1/Pkp3/Itch	5
GO:0001937 negative regulation of endothelial cell proliferation	1/138	42/14464	0.02380952	2.49551415	0.95259586	0.33183318	0.69312881	0.68075151	S2bpcox16	1
GO:0019692 deoxyribose phosphate metabolic process	1/138	42/14464	0.02380952	2.49551415	0.95259586	0.33183318	0.69312881	0.68075151	Tbpl1	1
GO:0030835 negative regulation of actin filament depolymerization	1/138	42/14464	0.02380952	2.49551415	0.95259586	0.33183318	0.69312881	0.68075151	Scin	1
GO:0031670 cellular response to nutrient	1/138	42/14464	0.02380952	2.49551415	0.95259586	0.33183318	0.69312881	0.68075151	Pdia3	1
GO:0032655 regulation of interleukin-12 production	1/138	42/14464	0.02380952	2.49551415	0.95259586	0.33183318	0.69312881	0.68075151	Hspd1	1
GO:0035774 positive regulation of insulin secretion involved in cellular response to glucose stimulus	1/138	42/14464	0.02380952	2.49551415	0.95259586	0.33183318	0.69312881	0.68075151	Sri	1
GO:0045103 intermediate filament-based process	1/138	42/14464	0.02380952	2.49551415	0.95259586	0.33183318	0.69312881	0.68075151	Pkp2	1
GO:0045777 positive regulation of blood pressure	1/138	42/14464	0.02380952	2.49551415	0.95259586	0.33183318	0.69312881	0.68075151	Manf	1
GO:0046677 response to antibiotic	1/138	42/14464	0.02380952	2.49551415	0.95259586	0.33183318	0.69312881	0.68075151	Ppp2cb	1
GO:0060428 lung epithelium development	1/138	42/14464	0.02380952	2.49551415	0.95259586	0.33183318	0.69312881	0.68075151	Lta4h	1
GO:0072595 maintenance of protein localization in organelle	1/138	42/14464	0.02380952	2.49551415	0.95259586	0.33183318	0.69312881	0.68075151	Hspa5	1
GO:0086009 membrane repolarization	1/138	42/14464	0.02380952	2.49551415	0.95259586	0.33183318	0.69312881	0.68075151	Kcnip2	1
GO:0090630 activation of GTPase activity	1/138	42/14464	0.02380952	2.49551415	0.95259586	0.33183318	0.69312881	0.68075151	Arhgef16	1
GO:1903573 negative regulation of response to endoplasmic reticulum stress	1/138	42/14464	0.02380952	2.49551415	0.95259586	0.33183318	0.69312881	0.68075151	Hspa5	1
GO:0030073 insulin secretion	3/138	213/14464	0.01408451	1.47621964	0.68719611	0.33212422	0.69312881	0.68075151	Bmal1/Smad2/Sri	3
GO:0034764 positive regulation of transmembrane transport	3/138	214/14464	0.01401869	1.46932141	0.67885354	0.33473779	0.69343365	0.68105091	Slc17a8/Kcnip2/Sri	3
GO:0022037 metencephalon development	2/138	125/14464	0.016	1.67698551	0.7460728	0.33532263	0.69343365	0.68105091	Hspa5/Fktn	2
GO:0045727 positive regulation of translation	2/138	125/14464	0.016	1.67698551	0.7460728	0.33532263	0.69343365	0.68105091	Dnajc3/Mettl14	2
GO:0002820 negative regulation of adaptive immune response	1/138	43/14464	0.02325581	2.43747893	0.92649758	0.33822668	0.69343365	0.68105091	Cr1l	1
GO:0003197 endocardial cushion development	1/138	43/14464	0.02325581	2.43747893	0.92649758	0.33822668	0.69343365	0.68105091	Smad2	1
GO:0006984 ER-nucleus signaling pathway	1/138	43/14464	0.02325581	2.43747893	0.92649758	0.33822668	0.69343365	0.68105091	Hspa5	1
GO:0009262 deoxyribonucleotide metabolic process	1/138	43/14464	0.02325581	2.43747893	0.92649758	0.33822668	0.69343365	0.68105091	Tbpl1	1
GO:0010803 regulation of tumor necrosis factor-mediated signaling pathway	1/138	43/14464	0.02325581	2.43747893	0.92649758	0.33822668	0.69343365	0.68105091	Ppp2cb	1
GO:0031146 SCF-dependent proteasomal ubiquitin-dependent protein catabolic process	1/138	43/14464	0.02325581	2.43747893	0.92649758	0.33822668	0.69343365	0.68105091	Cul2	1
GO:0032653 regulation of interleukin-10 production	1/138	43/14464	0.02325581	2.43747893	0.92649758	0.33822668	0.69343365	0.68105091	Hspd1	1
GO:0045022 early endosome to late endosome transport	1/138	43/14464	0.02325581	2.43747893	0.92649758	0.33822668	0.69343365	0.68105091	Aktip	1
GO:0050856 regulation of T cell receptor signaling pathway	1/138	43/14464	0.02325581	2.43747893	0.92649758	0.33822668	0.69343365	0.68105091	Card11	1
GO:0098586 cellular response to virus	1/138	43/14464	0.02325581	2.43747893	0.92649758	0.33822668	0.69343365	0.68105091	Calr	1
GO:0099560 synaptic membrane adhesion	1/138	43/14464	0.02325581	2.43747893	0.92649758	0.33822668	0.69343365	0.68105091	Cdh10	1
GO:1903018 regulation of glycoprotein metabolic process	1/138	43/14464	0.02325581	2.43747893	0.92649758	0.33822668	0.69343365	0.68105091	Fktn	1
GO:2000378 negative regulation of reactive oxygen species metabolic process	1/138	43/14464	0.02325581	2.43747893	0.92649758	0.33822668	0.69343365	0.68105091	Hspd1	1
GO:0051098 regulation of binding	4/138	308/14464	0.01298701	1.36118954	0.62884919	0.33876709	0.69343365	0.68105091	Smad2/Fktn/Sri/Aktip	4
GO:0001838 embryonic epithelial tube formation	2/138	126/14464	0.01587302	1.6636761	0.73435055	0.33880563	0.69343365	0.68105091	Grhl3/Ift172	2
GO:0006959 humoral immune response	2/138	126/14464	0.01587302	1.6636761	0.73435055	0.33880563	0.69343365	0.68105091	Ighg2c/Cr1l	2
GO:0016236 macroautophagy	4/138	309/14464	0.01294498	1.35678439	0.62220918	0.34093642	0.69393789	0.68154614	Snx14/Snx18/Wdr45b/Sec22b	4
GO:0001936 regulation of endothelial cell proliferation	2/138	127/14464	0.01574803	1.65057629	0.72273188	0.34228319	0.69393789	0.68154614	S2bpcox16/Aggf1	2
GO:0050921 positive regulation of chemotaxis	2/138	127/14464	0.01574803	1.65057629	0.72273188	0.34228319	0.69393789	0.68154614	Calr/Oxsr1	2
GO:0002066 columnar/cuboidal epithelial cell development	1/138	44/14464	0.02272727	2.38208169	0.90112219	0.34455944	0.69393789	0.68154614	Bmal1	1
GO:0006356 regulation of transcription by RNA polymerase I	1/138	44/14464	0.02272727	2.38208169	0.90112219	0.34455944	0.69393789	0.68154614	Rasl11a	1
GO:0006476 protein deacetylation	1/138	44/14464	0.02272727	2.38208169	0.90112219	0.34455944	0.69393789	0.68154614	Brms1	1
GO:0006767 water-soluble vitamin metabolic process	1/138	44/14464	0.02272727	2.38208169	0.90112219	0.34455944	0.69393789	0.68154614	Akr1a1	1
GO:0030857 negative regulation of epithelial cell differentiation	1/138	44/14464	0.02272727	2.38208169	0.90112219	0.34455944	0.69393789	0.68154614	Spred3	1
GO:0032615 interleukin-12 production	1/138	44/14464	0.02272727	2.38208169	0.90112219	0.34455944	0.69393789	0.68154614	Hspd1	1
GO:0034113 heterotypic cell-cell adhesion	1/138	44/14464	0.02272727	2.38208169	0.90112219	0.34455944	0.69393789	0.68154614	Pkp2	1

GO:0043039 tRNA aminoacylation	1/138	44/14464	0.02272727	2.38208169	0.90112219	0.34455944	0.69393789	0.68154614	Yars2	1
GO:0046148 pigment biosynthetic process	1/138	44/14464	0.02272727	2.38208169	0.90112219	0.34455944	0.69393789	0.68154614	Rab38	1
GO:0050919 negative chemotaxis	1/138	44/14464	0.02272727	2.38208169	0.90112219	0.34455944	0.69393789	0.68154614	Sema3e	1
GO:0051438 regulation of ubiquitin-protein transferase activity	1/138	44/14464	0.02272727	2.38208169	0.90112219	0.34455944	0.69393789	0.68154614	Bag5	1
GO:0051602 response to electrical stimulus	1/138	44/14464	0.02272727	2.38208169	0.90112219	0.34455944	0.69393789	0.68154614	Calr	1
GO:1902116 negative regulation of organelle assembly	1/138	44/14464	0.02272727	2.38208169	0.90112219	0.34455944	0.69393789	0.68154614	Sec22b	1
GO:0014902 myotube differentiation	2/138	128/14464	0.015625	1.63768116	0.71121488	0.3457551	0.69495741	0.68254746	Mmp14/Fktn	2
GO:0046328 regulation of JNK cascade	2/138	128/14464	0.015625	1.63768116	0.71121488	0.3457551	0.69495741	0.68254746	Fktn/Itch	2
GO:0002440 production of molecular mediator of immune response	3/138	219/14464	0.01369863	1.43577526	0.63776351	0.34779827	0.696825	0.6843817	Card11/Hspd1/Pkp3	3
GO:0030183 B cell differentiation	2/138	129/14464	0.01550388	1.62498596	0.69979773	0.34922116	0.696825	0.6843817	Card11/Mmp14	2
GO:0032543 mitochondrial translation	2/138	129/14464	0.01550388	1.62498596	0.69979773	0.34922116	0.696825	0.6843817	Mrps6/Yars2	2
GO:0060541 respiratory system development	3/138	220/14464	0.01363636	1.42924901	0.62966699	0.35040809	0.696825	0.6843817	Mmp14/Smad2/Lta4h	3
GO:0002204 somatic recombination of immunoglobulin genes involved in immune response	1/138	45/14464	0.02222222	2.32914654	0.87643117	0.35083203	0.696825	0.6843817	Hspd1	1
GO:0002208 somatic diversification of immunoglobulins involved in immune response	1/138	45/14464	0.02222222	2.32914654	0.87643117	0.35083203	0.696825	0.6843817	Hspd1	1
GO:0007606 sensory perception of chemical stimulus	1/138	45/14464	0.02222222	2.32914654	0.87643117	0.35083203	0.696825	0.6843817	Ric8b	1
GO:0007628 adult walking behavior	1/138	45/14464	0.02222222	2.32914654	0.87643117	0.35083203	0.696825	0.6843817	Hexa	1
GO:0031638 zymogen activation	1/138	45/14464	0.02222222	2.32914654	0.87643117	0.35083203	0.696825	0.6843817	Mmp14	1
GO:0043038 amino acid activation	1/138	45/14464	0.02222222	2.32914654	0.87643117	0.35083203	0.696825	0.6843817	Yars2	1
GO:0045190 isotype switching	1/138	45/14464	0.02222222	2.32914654	0.87643117	0.35083203	0.696825	0.6843817	Hspd1	1
GO:1902622 regulation of neutrophil migration	1/138	45/14464	0.02222222	2.32914654	0.87643117	0.35083203	0.696825	0.6843817	Cd99l2	1
GO:0042886 amide transport	4/138	314/14464	0.01273885	1.33517954	0.58934604	0.35178967	0.69803935	0.68557436	Bmal1/Smad2/Sri/Slc33a1	4
GO:0007266 Rho protein signal transduction	2/138	130/14464	0.01538462	1.61248606	0.68847861	0.35268115	0.69912015	0.68663586	Rhoj/Stard13	2
GO:0071772 response to BMP	2/138	131/14464	0.01526718	1.60017701	0.67725577	0.35613488	0.69951679	0.68702542	Dand5/Smad2	2
GO:0071773 cellular response to BMP stimulus	2/138	131/14464	0.01526718	1.60017701	0.67725577	0.35613488	0.69951679	0.68702542	Dand5/Smad2	2
GO:0001947 heart looping	1/138	46/14464	0.02173913	2.27851292	0.85238891	0.35704503	0.69951679	0.68702542	Ift172	1
GO:0010824 regulation of centrosome duplication	1/138	46/14464	0.02173913	2.27851292	0.85238891	0.35704503	0.69951679	0.68702542	Chordc1	1
GO:0032613 interleukin-10 production	1/138	46/14464	0.02173913	2.27851292	0.85238891	0.35704503	0.69951679	0.68702542	Hspd1	1
GO:0032892 positive regulation of organic acid transport	1/138	46/14464	0.02173913	2.27851292	0.85238891	0.35704503	0.69951679	0.68702542	Slc17a8	1
GO:0051155 positive regulation of striated muscle cell differentiation	1/138	46/14464	0.02173913	2.27851292	0.85238891	0.35704503	0.69951679	0.68702542	Mmp14	1
GO:0051954 positive regulation of amine transport	1/138	46/14464	0.02173913	2.27851292	0.85238891	0.35704503	0.69951679	0.68702542	Slc17a8	1
GO:0052372 modulation by symbiont of entry into host	1/138	46/14464	0.02173913	2.27851292	0.85238891	0.35704503	0.69951679	0.68702542	P4hb	1
GO:0055010 ventricular cardiac muscle tissue morphogenesis	1/138	46/14464	0.02173913	2.27851292	0.85238891	0.35704503	0.69951679	0.68702542	Pkp2	1
GO:0120163 negative regulation of cold-induced thermogenesis	1/138	46/14464	0.02173913	2.27851292	0.85238891	0.35704503	0.69951679	0.68702542	Bmal1	1
GO:2000772 regulation of cellular senescence	1/138	46/14464	0.02173913	2.27851292	0.85238891	0.35704503	0.69951679	0.68702542	Bmal1	1
GO:0051345 positive regulation of hydrolase activity	4/138	317/14464	0.0126183	1.32254378	0.56989177	0.35830439	0.70130257	0.68877931	Arhgef16/Grhl3/Hspd1/Snx18	4
GO:1903522 regulation of blood circulation	3/138	224/14464	0.01339286	1.40372671	0.59767179	0.3608361	0.70337095	0.69081075	Kcnip2/Pkp2/Sri	3
GO:0006644 phospholipid metabolic process	4/138	319/14464	0.01253918	1.31425196	0.55702942	0.36264755	0.70337095	0.69081075	Rab38/Dgkk/Pigg/Selenoi	4
GO:0072594 establishment of protein localization to organelle	5/138	415/14464	0.01204819	1.26279029	0.53311011	0.36305114	0.70337095	0.69081075	Bmal1/Hspa5/Hspd1/Ubl4a/Srprb	5
GO:0030514 negative regulation of BMP signaling pathway	1/138	47/14464	0.0212766	2.23003392	0.8289624	0.36319899	0.70337095	0.69081075	Dand5	1
GO:0032330 regulation of chondrocyte differentiation	1/138	47/14464	0.0212766	2.23003392	0.8289624	0.36319899	0.70337095	0.69081075	Scin	1
GO:0033120 positive regulation of RNA splicing	1/138	47/14464	0.0212766	2.23003392	0.8289624	0.36319899	0.70337095	0.69081075	Obi1	1
GO:0048066 developmental pigmentation	1/138	47/14464	0.0212766	2.23003392	0.8289624	0.36319899	0.70337095	0.69081075	Rab38	1
GO:0051955 regulation of amino acid transport	1/138	47/14464	0.0212766	2.23003392	0.8289624	0.36319899	0.70337095	0.69081075	Slc17a8	1
GO:0071526 semaphorin-plexin signaling pathway	1/138	47/14464	0.0212766	2.23003392	0.8289624	0.36319899	0.70337095	0.69081075	Sema3e	1
GO:0170038 proteinogenic amino acid biosynthetic process	1/138	47/14464	0.0212766	2.23003392	0.8289624	0.36319899	0.70337095	0.69081075	Enoph1	1
GO:1902459 positive regulation of stem cell population maintenance	1/138	47/14464	0.0212766	2.23003392	0.8289624	0.36319899	0.70337095	0.69081075	Brms1	1
GO:0046661 male sex differentiation	2/138	134/14464	0.01492537	1.56435215	0.64414813	0.36645658	0.70899852	0.69633784	Mmp14/Lhfpl2	2
GO:0030219 megakaryocyte differentiation	1/138	48/14464	0.02083333	2.18357488	0.806121	0.36929447	0.71107702	0.69837922	Scin	1
GO:0032506 cytokinetic process	1/138	48/14464	0.02083333	2.18357488	0.806121	0.36929447	0.71107702	0.69837922	Snx18	1
GO:0045071 negative regulation of viral genome replication	1/138	48/14464	0.02083333	2.18357488	0.806121	0.36929447	0.71107702	0.69837922	Srpk1	1
GO:0085029 extracellular matrix assembly	1/138	48/14464	0.02083333	2.18357488	0.806121	0.36929447	0.71107702	0.69837922	Has2	1
GO:0170034 L-amino acid biosynthetic process	1/138	48/14464	0.02083333	2.18357488	0.806121	0.36929447	0.71107702	0.69837922	Enoph1	1
GO:0071902 positive regulation of protein serine/threonine kinase activity	2/138	135/14464	0.01481481	1.55276436	0.6332938	0.36988336	0.71153135	0.69882543	Fzd8/Sash1	2
GO:0072522 purine-containing compound biosynthetic process	3/138	228/14464	0.01315789	1.37909992	0.56628283	0.37124093	0.71246793	0.69974528	Ppcs/Elovl7/Pdk3	3
GO:0034765 regulation of monoatomic ion transmembrane transport	4/138	323/14464	0.0123839	1.2979764	0.53155637	0.37133155	0.71246793	0.69974528	Rrad/Kcnip2/Sri/Oxsr1	4
GO:0072175 epithelial tube formation	2/138	136/14464	0.01470588	1.54134697	0.62252765	0.37330294	0.71246793	0.69974528	Grhl3/Ift172	2
GO:1904064 positive regulation of cation transmembrane transport	2/138	136/14464	0.01470588	1.54134697	0.62252765	0.37330294	0.71246793	0.69974528	Kcnip2/Sri	2
GO:2000241 regulation of reproductive process	2/138	136/14464	0.01470588	1.54134697	0.62252765	0.37330294	0.71246793	0.69974528	Calr/Lhfpl2	2
GO:0001755 neural crest cell migration	1/138	49/14464	0.02040816	2.13901213	0.78383622	0.37533202	0.71246793	0.69974528	Sema3e	1
GO:0003179 heart valve morphogenesis	1/138	49/14464	0.02040816	2.13901213	0.78383622	0.37533202	0.71246793	0.69974528	Smad2	1
GO:0006687 glycosphingolipid metabolic process	1/138	49/14464	0.02040816	2.13901213	0.78383622	0.37533202	0.71246793	0.69974528	Hexa	1
GO:0006721 terpenoid metabolic process	1/138	49/14464	0.02040816	2.13901213	0.78383622	0.37533202	0.71246793	0.69974528	Dhrs3	1
GO:0032729 positive regulation of type II interferon production	1/138	49/14464	0.02040816	2.13901213	0.78383622	0.37533202	0.71246793	0.69974528	Hspd1	1
GO:0032881 regulation of polysaccharide metabolic process	1/138	49/14464	0.02040816	2.13901213	0.78383622	0.37533202	0.71246793	0.69974528	Has2	1
GO:0043030 regulation of macrophage activation	1/138	49/14464	0.02040816	2.13901213	0.78383622	0.37533202	0.71246793	0.69974528	Hspd1	1
GO:0098927 vesicle-mediated transport between endosomal compartments	1/138	49/14464	0.02040816	2.13901213	0.78383622	0.37533202	0.71246793	0.69974528	Aktip	1
GO:0170040 proteinogenic amino acid catabolic process	1/138	49/14464	0.02040816	2.13901213	0.78383622	0.37533202	0.71246793	0.69974528	Hmgcll1	1
GO:0046486 glycerolipid metabolic process	4/138	325/14464	0.01230769	1.28998885	0.51894329	0.37567133	0.71246793	0.69974528	Rab38/Dgkk/Pigg/Selenoi	4
GO:0007159 leukocyte cell-cell adhesion	4/138	326/14464	0.01226994	1.28603183	0.51266709	0.37784045	0.71509338	0.70232385	Card11/Has2/Hspd1/Itch	4
GO:1903322 positive regulation of protein modification by small protein conjugation or removal	2/138	138/14464	0.01449275	1.51900861	0.60125389	0.38011984	0.71509338	0.70232385	Hspa5/Ndfip2	2
GO:0000245 spliceosomal complex assembly	1/138	50/14464	0.02	2.09623188	0.76208153	0.38131219	0.71509338	0.70232385	Srpk1	1
GO:0002712 regulation of B cell mediated immunity	1/138	50/14464	0.02	2.09623188	0.76208153	0.38131219	0.71509338	0.70232385	Cr1l	1
GO:0002889 regulation of immunoglobulin mediated immune response	1/138	50/14464	0.02	2.09623188	0.76208153	0.38131219	0.71509338	0.70232385	Cr1l	1
GO:0003254 regulation of membrane depolarization	1/138	50/14464	0.02	2.09623188	0.76208153	0.38131219	0.71509338	0.70232385	Gclm	1
GO:0010634 positive regulation of epithelial cell migration	1/138	50/14464	0.02	2.09623188	0.76208153	0.38131219	0.71509338	0.70232385	Has2	1
GO:0010718 positive regulation of epithelial to mesenchymal transition	1/138	50/14464	0.02	2.09623188	0.76208153	0.38131219	0.71509338	0.70232385	Smad2	1
GO:0030834 regulation of actin filament depolymerization	1/138	50/14464	0.02	2.09623188	0.76208153	0.38131219	0.71509338	0.70232385	Scin	1
GO:0032663 regulation of interleukin-2 production	1/138	50/14464	0.02	2.09623188	0.76208153	0.38131219	0.71509338	0.70232385	Card11	1
GO:0043903 regulation of biological process involved in symbiotic interaction	1/138	50/14464	0.02	2.09623188	0.76208153	0.38131219	0.71509338	0.70232385	P4hb	1
GO:0090497 mesenchymal cell migration	1/138	50/14464	0.02	2.09623188	0.76208153	0.38131219	0.71509338	0.70232385	Sema3e	1
GO:0007584 response to nutrient	2/138	139/14464	0.01438849	1.50808049	0.59074339	0.38351681	0.71789219	0.70507269	Pdia3/Gclm	2
GO:1901875 positive regulation of post-translational protein modification	2/138	139/14464	0.01438849	1.50808049	0.59074339	0.38351681	0.71789219	0.70507269	Hspa5/Ndfip2	2
GO:0002706 regulation of lymphocyte mediated immunity	2/138	140/14464	0.01428571	1.49730849	0.58031527	0.38690591	0.71884606	0.70600952	Hspd1/Cr1l	2
GO:0008360 regulation of cell shape	2/138	140/14464	0.01428571	1.49730849	0.58031527	0.38690591	0.71884606	0.70600952	Rhoj/Sema3e	2
GO:0043405 regulation of MAP kinase activity	2/138	140/14464	0.01428571	1.49730849	0.58031527	0.38690591	0.71884606	0.70600952	Fzd8/Sash1	2
GO:0002711 positive regulation of T cell mediated immunity	1/138	51/14464	0.01960784	2.0551293	0.74083218	0.38723552	0.71884606	0.70600952	Hspd1	1
GO:0034605 cellular response to heat	1/138	51/14464	0.01960784	2.0551293	0.74083218	0.38723552	0.71884606	0.70600952	Chordc1	1
GO:0046470 phosphatidylcholine metabolic process	1/138	51/14464	0.01960784	2.0551293	0.74083218	0.38723552	0.71884606	0.70600952	Rab38	1
GO:0070098 chemokine-mediated signaling pathway	1/138	51/14464	0.01960784	2.0551293	0.74083218	0.38723552	0.71884606	0.70600952	Oxsr1	1
GO:0072132 mesenchyme morphogenesis	1/138	51/14464	0.01960784	2.0551293	0.74083218	0.38723552	0.71884606	0.70600952	Smad2	1
GO:1902692 regulation of neuroblast proliferation	1/138	51/14464	0.01960784	2.0551293	0.74083218	0.38723552	0.71884606	0.70600952	Tafa1	1
GO:0071900 regulation of protein serine/threonine kinase activity	3/138	235/14464	0.01276596	1.33802035	0.51273841	0.38937384	0.7214712	0.70858779	Chordc1/Fzd8/Sash1	3
GO:0007605 sensory perception of sound	2/138	141/14464	0.0141844	1.48668928	0.56996817	0.39028699	0.7214712	0.70858779	Slc17a8/Hexa	2
GO:0002449 lymphocyte mediated immunity	3/138	236/14464	0.01271186	1.33235077	0.50522756	0.39195484	0.7214712	0.70858779	Ighg2c/Hspd1/Cr1l	3
GO:0001707 mesoderm formation	1/138	52/14464	0.01923077	2.01560758	0.72006507	0.39310255	0.7214712	0.70858779	Smad2	1
GO:0002704 negative regulation of leukocyte mediated immunity	1/138	52/14464	0.01923077	2.01560758	0.72006507	0.39310255	0.7214712	0.70858779	Cr1l	1
GO:0006111 regulation of gluconeogenesis	1/138	52/14464	0.01923077	2.01560758	0.72006507	0.39310255	0.7214712	0.70858779	Arpp19	1
GO:0006749 glutathione metabolic process	1/138	52/14464	0.01923077	2.01560758	0.72006507	0.39310255	0.7214712	0.70858779	Gclm	1
GO:0021587 cerebellum morphogenesis	1/138	52/14464	0.01923077	2.01560758	0.72006507	0.39310255	0.7214712	0.70858779	Hspa5	1
GO:0032210 regulation of telomere maintenance via telomerase	1/138	52/14464	0.01923077	2.01560758	0.72006507	0.39310255	0.7214712	0.70858779	Hnrnpc	1
GO:0086001 cardiac muscle cell action potential	1/138	52/14464	0.01923077	2.01560758	0.72006507	0.39310255	0.7214712	0.70858779	Pkp2	1
GO:0089718 amino acid import across plasma membrane	1/138	52/14464	0.01923077	2.01560758	0.72006507	0.39310255	0.7214712	0.70858779	Slc17a8	1
GO:2000242 negative regulation of reproductive process	1/138	52/14464	0.01923077	2.01560758	0.72006507	0.39310255	0.7214712	0.70858779	Calr	1
GO:0002253 activation of immune response	5/138	431/14464	0.01160093	1.21591177	0.446628	0.39345616	0.7214712	0.70858779	Card11/Ighg2c/Hspd1/Cr1l/Itch	5
GO:0006937 regulation of muscle contraction	2/138	142/14464	0.01408451	1.47621964	0.55970076	0.39365988	0.7214712	0.70858779	Pkp2/Sri	2
GO:0007009 plasma membrane organization	2/138	143/14464	0.01398601	1.46589642	0.54951175	0.39702444	0.72256087	0.70965799	Degs1/Snx18	2
GO:0042129 regulation of T cell proliferation	2/138	143/14464	0.01398601	1.46589642	0.54951175	0.39702444	0.72256087	0.70965799	Card11/Itch	2
GO:0001738 morphogenesis of a polarized epithelium	1/138	53/14464	0.01886792	1.97757725	0.69975857	0.39891381	0.72256087	0.70965799	Grhl3	1
GO:0002067 glandular epithelial cell differentiation	1/138	53/14464	0.01886792	1.97757725	0.69975857	0.39891381	0.72256087	0.70965799	Bmal1	1
GO:0003143 embryonic heart tube morphogenesis	1/138	53/14464	0.01886792	1.97757725	0.69975857	0.39891381	0.72256087	0.70965799	Ift172	1
GO:0033059 cellular pigmentation	1/138	53/14464	0.01886792	1.97757725	0.69975857	0.39891381	0.72256087	0.70965799	Rab38	1
GO:0042149 cellular response to glucose starvation	1/138	53/14464	0.01886792	1.97757725	0.69975857	0.39891381	0.72256087	0.70965799	Hspa5	1
GO:0045599 negative regulation of fat cell differentiation	1/138	53/14464	0.01886792	1.97757725	0.69975857	0.39891381	0.72256087	0.70965799	Bmal1	1
GO:0046578 regulation of Ras protein signal transduction	1/138	53/14464	0.01886792	1.97757725	0.69975857	0.39891381	0.72256087	0.70965799	Ppp2cb	1
GO:0060135 maternal process involved in female pregnancy	1/138	53/14464	0.01886792	1.97757725	0.69975857	0.39891381	0.72256087	0.70965799	Tppp3	1
GO:0070527 platelet aggregation	1/138	53/14464	0.01886792	1.97757725	0.69975857	0.39891381	0.72256087	0.70965799	Pdia3	1
GO:0170035 L-amino acid catabolic process	1/138	53/14464	0.01886792	1.97757725	0.69975857	0.39891381	0.72256087	0.70965799	Hmgcll1	1
GO:1901607 alpha-amino acid biosynthetic process	1/138	53/14464	0.01886792	1.97757725	0.69975857	0.39891381	0.72256087	0.70965799	Enoph1	1
GO:0016241 regulation of macroautophagy	2/138	144/14464	0.01388889	1.45571659	0.53939987	0.40038052	0.72456654	0.71162785	Snx18/Sec22b	2
GO:0002250 adaptive immune response	4/138	337/14464	0.01186944	1.24405453	0.44492122	0.40164728	0.72522947	0.71227895	Ighg2c/Pdia3/Hspd1/Cr1l	4
GO:0042113 B cell activation	3/138	240/14464	0.0125	1.31014493	0.47551661	0.40225152	0.72522947	0.71227895	Card11/Mmp14/Hspd1	3
GO:0022613 ribonucleoprotein complex biogenesis	5/138	436/14464	0.01146789	1.20196782	0.42027525	0.40294627	0.72522947	0.71227895	Mettl16/Srpk1/Prpf31/Tfb1m/Prpf18	5
GO:0002381 immunoglobulin production involved in immunoglobulin-mediated immune response	1/138	54/14464	0.01851852	1.94095545	0.67989244	0.40466982	0.72522947	0.71227895	Hspd1	1
GO:0016447 somatic recombination of immunoglobulin gene segments	1/138	54/14464	0.01851852	1.94095545	0.67989244	0.40466982	0.72522947	0.71227895	Hspd1	1
GO:0021575 hindbrain morphogenesis	1/138	54/14464	0.01851852	1.94095545	0.67989244	0.40466982	0.72522947	0.71227895	Hspa5	1
GO:0030042 actin filament depolymerization	1/138	54/14464	0.01851852	1.94095545	0.67989244	0.40466982	0.72522947	0.71227895	Scin	1
GO:0031663 lipopolysaccharide-mediated signaling pathway	1/138	54/14464	0.01851852	1.94095545	0.67989244	0.40466982	0.72522947	0.71227895	Sash1	1
GO:0035601 protein deacylation	1/138	54/14464	0.01851852	1.94095545	0.67989244	0.40466982	0.72522947	0.71227895	Brms1	1
GO:0090659 walking behavior	1/138	54/14464	0.01851852	1.94095545	0.67989244	0.40466982	0.72522947	0.71227895	Hexa	1
GO:0031669 cellular response to nutrient levels	3/138	241/14464	0.01244813	1.30470864	0.4681704	0.40481841	0.72522947	0.71227895	Hspa5/Pdia3/Wdr45b	3
GO:0000768 syncytium formation by plasma membrane fusion	1/138	55/14464	0.01818182	1.90566535	0.66044771	0.41037111	0.72522947	0.71227895	Sh3pxd2a	1
GO:0010907 positive regulation of glucose metabolic process	1/138	55/14464	0.01818182	1.90566535	0.66044771	0.41037111	0.72522947	0.71227895	Arpp19	1
GO:0030865 cortical cytoskeleton organization	1/138	55/14464	0.01818182	1.90566535	0.66044771	0.41037111	0.72522947	0.71227895	Calr	1
GO:0046605 regulation of centrosome cycle	1/138	55/14464	0.01818182	1.90566535	0.66044771	0.41037111	0.72522947	0.71227895	Chordc1	1
GO:0070542 response to fatty acid	1/138	55/14464	0.01818182	1.90566535	0.66044771	0.41037111	0.72522947	0.71227895	Pdk3	1
GO:0072527 pyrimidine-containing compound metabolic process	1/138	55/14464	0.01818182	1.90566535	0.66044771	0.41037111	0.72522947	0.71227895	Tbpl1	1
GO:0140253 cell-cell fusion	1/138	55/14464	0.01818182	1.90566535	0.66044771	0.41037111	0.72522947	0.71227895	Sh3pxd2a	1
GO:1901799 negative regulation of proteasomal protein catabolic process	1/138	55/14464	0.01818182	1.90566535	0.66044771	0.41037111	0.72522947	0.71227895	Bag5	1
GO:1905517 macrophage migration	1/138	55/14464	0.01818182	1.90566535	0.66044771	0.41037111	0.72522947	0.71227895	Mmp14	1
GO:2000059 negative regulation of ubiquitin-dependent protein catabolic process	1/138	55/14464	0.01818182	1.90566535	0.66044771	0.41037111	0.72522947	0.71227895	Bag5	1
GO:0060041 retina development in camera-type eye	2/138	147/14464	0.01360544	1.42600808	0.50951475	0.41039642	0.72522947	0.71227895	Rhoj/Slc17a8	2
GO:2001236 regulation of extrinsic apoptotic signaling pathway	2/138	147/14464	0.01360544	1.42600808	0.50951475	0.41039642	0.72522947	0.71227895	Pdia3/Gclm	2
GO:0035148 tube formation	2/138	148/14464	0.01351351	1.41637289	0.49969928	0.41371716	0.72522947	0.71227895	Grhl3/Ift172	2
GO:0045766 positive regulation of angiogenesis	2/138	148/14464	0.01351351	1.41637289	0.49969928	0.41371716	0.72522947	0.71227895	Sash1/Aggf1	2
GO:0060326 cell chemotaxis	3/138	245/14464	0.0122449	1.28340728	0.43910236	0.41505425	0.72522947	0.71227895	Calr/Arhgef16/Oxsr1	3
GO:0006497 protein lipidation	1/138	56/14464	0.01785714	1.87163561	0.64140655	0.41601819	0.72522947	0.71227895	Wdr45b	1
GO:0021536 diencephalon development	1/138	56/14464	0.01785714	1.87163561	0.64140655	0.41601819	0.72522947	0.71227895	Sema3e	1
GO:0038066 p38MAPK cascade	1/138	56/14464	0.01785714	1.87163561	0.64140655	0.41601819	0.72522947	0.71227895	Sash1	1
GO:0039532 negative regulation of cytoplasmic pattern recognition receptor signaling pathway	1/138	56/14464	0.01785714	1.87163561	0.64140655	0.41601819	0.72522947	0.71227895	Itch	1
GO:0043462 regulation of ATP-dependent activity	1/138	56/14464	0.01785714	1.87163561	0.64140655	0.41601819	0.72522947	0.71227895	Dnajb11	1
GO:0043536 positive regulation of blood vessel endothelial cell migration	1/138	56/14464	0.01785714	1.87163561	0.64140655	0.41601819	0.72522947	0.71227895	Rhoj	1
GO:0048332 mesoderm morphogenesis	1/138	56/14464	0.01785714	1.87163561	0.64140655	0.41601819	0.72522947	0.71227895	Smad2	1
GO:0050688 regulation of defense response to virus	1/138	56/14464	0.01785714	1.87163561	0.64140655	0.41601819	0.72522947	0.71227895	Itch	1
GO:0050854 regulation of antigen receptor-mediated signaling pathway	1/138	56/14464	0.01785714	1.87163561	0.64140655	0.41601819	0.72522947	0.71227895	Card11	1
GO:0051646 mitochondrion localization	1/138	56/14464	0.01785714	1.87163561	0.64140655	0.41601819	0.72522947	0.71227895	S2bpcox16	1
GO:0070830 bicellular tight junction assembly	1/138	56/14464	0.01785714	1.87163561	0.64140655	0.41601819	0.72522947	0.71227895	Pkp2	1
GO:0072348 sulfur compound transport	1/138	56/14464	0.01785714	1.87163561	0.64140655	0.41601819	0.72522947	0.71227895	Slc33a1	1
GO:2001238 positive regulation of extrinsic apoptotic signaling pathway	1/138	56/14464	0.01785714	1.87163561	0.64140655	0.41601819	0.72522947	0.71227895	Pdia3	1
GO:0008015 blood circulation	5/138	443/14464	0.01128668	1.1829751	0.38389339	0.41620664	0.72522947	0.71227895	Manf/Kcnip2/Gclm/Pkp2/Sri	5
GO:0042176 regulation of protein catabolic process	4/138	344/14464	0.01162791	1.21873947	0.40299094	0.41672213	0.72522947	0.71227895	Bmal1/Bag5/Itch/Sec22b	4
GO:0016331 morphogenesis of embryonic epithelium	2/138	149/14464	0.01342282	1.40686704	0.48995504	0.41702872	0.72522947	0.71227895	Grhl3/Ift172	2
GO:0046631 alpha-beta T cell activation	2/138	149/14464	0.01342282	1.40686704	0.48995504	0.41702872	0.72522947	0.71227895	Card11/Itch	2
GO:0050792 regulation of viral process	2/138	149/14464	0.01342282	1.40686704	0.48995504	0.41702872	0.72522947	0.71227895	Srpk1/P4hb	2
GO:0006941 striated muscle contraction	2/138	150/14464	0.01333333	1.39748792	0.48028091	0.42033099	0.72522947	0.71227895	Pkp2/Sri	2
GO:0048754 branching morphogenesis of an epithelial tube	2/138	150/14464	0.01333333	1.39748792	0.48028091	0.42033099	0.72522947	0.71227895	Mmp14/Sema3e	2
GO:0003229 ventricular cardiac muscle tissue development	1/138	57/14464	0.01754386	1.8387999	0.62275222	0.42161158	0.72522947	0.71227895	Pkp2	1

GO:0006473	protein acetylation	1/138	57/14464	0.01754386	1.8387999	0.62275222	0.42161158	0.72522947	0.71227895	Bmal1	1
GO:0006949	syncytium formation	1/138	57/14464	0.01754386	1.8387999	0.62275222	0.42161158	0.72522947	0.71227895	Sh3pxd2a	1
GO:0009064	glutamine family amino acid metabolic process	1/138	57/14464	0.01754386	1.8387999	0.62275222	0.42161158	0.72522947	0.71227895	Gclm	1
GO:0032623	interleukin-2 production	1/138	57/14464	0.01754386	1.8387999	0.62275222	0.42161158	0.72522947	0.71227895	Card11	1
GO:0042440	pigment metabolic process	1/138	57/14464	0.01754386	1.8387999	0.62275222	0.42161158	0.72522947	0.71227895	Rab38	1
GO:0045010	actin nucleation	1/138	57/14464	0.01754386	1.8387999	0.62275222	0.42161158	0.72522947	0.71227895	Scin	1
GO:0046635	positive regulation of alpha-beta T cell activation	1/138	57/14464	0.01754386	1.8387999	0.62275222	0.42161158	0.72522947	0.71227895	Card11	1
GO:0055008	cardiac muscle tissue morphogenesis	1/138	57/14464	0.01754386	1.8387999	0.62275222	0.42161158	0.72522947	0.71227895	Pkp2	1
GO:0071470	cellular response to osmotic stress	1/138	57/14464	0.01754386	1.8387999	0.62275222	0.42161158	0.72522947	0.71227895	Oxsr1	1
GO:0099024	plasma membrane invagination	1/138	57/14464	0.01754386	1.8387999	0.62275222	0.42161158	0.72522947	0.71227895	Snx18	1
GO:1902808	positive regulation of cell cycle G1/S phase transition	1/138	57/14464	0.01754386	1.8387999	0.62275222	0.42161158	0.72522947	0.71227895	Pkp3	1
GO:0007224	smoothened signaling pathway	2/138	151/14464	0.01324503	1.38823304	0.47067582	0.42362383	0.72707377	0.71409031	Ift172/Ift80	2
GO:1904018	positive regulation of vasculature development	2/138	151/14464	0.01324503	1.38823304	0.47067582	0.42362383	0.72707377	0.71409031	Sash1/Aggf1	2
GO:0034762	regulation of transmembrane transport	5/138	447/14464	0.01118568	1.1723892	0.36336453	0.42376571	0.72707377	0.71409031	Rrad/Slc17a8/Kcnip2/Sri/Oxsr1	5
GO:0070613	regulation of protein processing	1/138	58/14464	0.01724138	1.80709645	0.60446894	0.42715177	0.73226018	0.71918411	Mmp14	1
GO:0005996	monosaccharide metabolic process	3/138	250/14464	0.012	1.25773913	0.40345813	0.42777134	0.73245964	0.71938	Pdk3/Arpp19/Akr1a1	3
GO:0031333	negative regulation of protein-containing complex assembly	2/138	153/14464	0.0130719	1.3700862	0.45166858	0.43018076	0.73245964	0.71938	Hspa5/Scin	2
GO:0034767	positive regulation of monoatomic ion transmembrane transport	2/138	153/14464	0.0130719	1.3700862	0.45166858	0.43018076	0.73245964	0.71938	Kcnip2/Sri	2
GO:0016050	vesicle organization	4/138	351/14464	0.01139601	1.19443412	0.36192838	0.43171536	0.73245964	0.71938	Rab38/Tbpl1/Aktip/Sec22b	4
GO:0003170	heart valve development	1/138	59/14464	0.01694915	1.7764677	0.58654189	0.43263928	0.73245964	0.71938	Smad2	1
GO:0006278	RNA-templated DNA biosynthetic process	1/138	59/14464	0.01694915	1.7764677	0.58654189	0.43263928	0.73245964	0.71938	Hnrnpc	1
GO:0007004	telomere maintenance via telomerase	1/138	59/14464	0.01694915	1.7764677	0.58654189	0.43263928	0.73245964	0.71938	Hnrnpc	1
GO:0035176	social behavior	1/138	59/14464	0.01694915	1.7764677	0.58654189	0.43263928	0.73245964	0.71938	Gnb1l	1
GO:0042158	lipoprotein biosynthetic process	1/138	59/14464	0.01694915	1.7764677	0.58654189	0.43263928	0.73245964	0.71938	Wdr45b	1
GO:0043627	response to estrogen	1/138	59/14464	0.01694915	1.7764677	0.58654189	0.43263928	0.73245964	0.71938	Mmp14	1
GO:0098732	macromolecule deacylation	1/138	59/14464	0.01694915	1.7764677	0.58654189	0.43263928	0.73245964	0.71938	Brms1	1
GO:0098900	regulation of action potential	1/138	59/14464	0.01694915	1.7764677	0.58654189	0.43263928	0.73245964	0.71938	Pkp2	1
GO:0051223	regulation of protein transport	5/138	452/14464	0.01106195	1.15942029	0.33796289	0.43319112	0.73245964	0.71938	Bmal1/Frat2/Sri/Gapvd1/Ndfip2	5
GO:0006575	cellular modified amino acid metabolic process	2/138	154/14464	0.01298701	1.36118954	0.44226438	0.43344462	0.73245964	0.71938	Gclm/P4hb	2
GO:0035150	regulation of tube size	2/138	154/14464	0.01298701	1.36118954	0.44226438	0.43344462	0.73245964	0.71938	Manf/Gclm	2
GO:0035296	regulation of tube diameter	2/138	154/14464	0.01298701	1.36118954	0.44226438	0.43344462	0.73245964	0.71938	Manf/Gclm	2
GO:0097746	blood vessel diameter maintenance	2/138	154/14464	0.01298701	1.36118954	0.44226438	0.43344462	0.73245964	0.71938	Manf/Gclm	2
GO:0046651	lymphocyte proliferation	3/138	253/14464	0.01185771	1.24282523	0.38242653	0.43535628	0.73460294	0.72148503	Card11/Hspd1/Itch	3
GO:0001508	action potential	2/138	155/14464	0.01290323	1.35240767	0.43292515	0.43669859	0.73460294	0.72148503	Kcnip2/Pkp2	2
GO:0050777	negative regulation of immune response	2/138	155/14464	0.01290323	1.35240767	0.43292515	0.43669859	0.73460294	0.72148503	Dtx4/Cr1l	2
GO:0009880	embryonic pattern specification	1/138	60/14464	0.01666667	1.7468599	0.56895704	0.4380746	0.73460294	0.72148503	Smad2	1
GO:0016445	somatic diversification of immunoglobulins	1/138	60/14464	0.01666667	1.7468599	0.56895704	0.4380746	0.73460294	0.72148503	Hspd1	1
GO:0034331	cell junction maintenance	1/138	60/14464	0.01666667	1.7468599	0.56895704	0.4380746	0.73460294	0.72148503	Pkp2	1
GO:0046513	ceramide biosynthetic process	1/138	60/14464	0.01666667	1.7468599	0.56895704	0.4380746	0.73460294	0.72148503	Degs1	1
GO:0051703	biological process involved in intraspecies interaction between organisms	1/138	60/14464	0.01666667	1.7468599	0.56895704	0.4380746	0.73460294	0.72148503	Gnb1l	1
GO:0002822	regulation of adaptive immune response based on somatic recombination of immune receptors built from	2/138	156/14464	0.01282051	1.34373839	0.42364992	0.43994255	0.73460294	0.72148503	Hspd1/Cr1l	2
GO:0007254	JNK cascade	2/138	156/14464	0.01282051	1.34373839	0.42364992	0.43994255	0.73460294	0.72148503	Fktn/Itch	2
GO:0072330	monocarboxylic acid biosynthetic process	2/138	156/14464	0.01282051	1.34373839	0.42364992	0.43994255	0.73460294	0.72148503	Degs1/Elovl7	2
GO:0006935	chemotaxis	4/138	356/14464	0.01123596	1.17765836	0.33310733	0.44236464	0.73460294	0.72148503	Calr/Arhgef16/Sema3e/Oxsr1	4
GO:0007389	pattern specification process	4/138	356/14464	0.01123596	1.17765836	0.33310733	0.44236464	0.73460294	0.72148503	Dand5/Grhl3/Smad2/Ift172	4
GO:0030072	peptide hormone secretion	3/138	256/14464	0.01171875	1.22826087	0.36165231	0.44290472	0.73460294	0.72148503	Bmal1/Smad2/Sri	3
GO:0090150	establishment of protein localization to membrane	3/138	256/14464	0.01171875	1.22826087	0.36165231	0.44290472	0.73460294	0.72148503	Hspa5/Ubl4a/Srprb	3
GO:0051168	nuclear export	2/138	157/14464	0.01273885	1.33517954	0.41443775	0.44317642	0.73460294	0.72148503	Calr/Frat2	2
GO:0002224	toll-like receptor signaling pathway	1/138	61/14464	0.01639344	1.71822286	0.55170119	0.44345823	0.73460294	0.72148503	Hspd1	1
GO:0009247	glycolipid biosynthetic process	1/138	61/14464	0.01639344	1.71822286	0.55170119	0.44345823	0.73460294	0.72148503	Pigg	1
GO:0030888	regulation of B cell proliferation	1/138	61/14464	0.01639344	1.71822286	0.55170119	0.44345823	0.73460294	0.72148503	Card11	1
GO:0120192	tight junction assembly	1/138	61/14464	0.01639344	1.71822286	0.55170119	0.44345823	0.73460294	0.72148503	Pkp2	1
GO:1903053	regulation of extracellular matrix organization	1/138	61/14464	0.01639344	1.71822286	0.55170119	0.44345823	0.73460294	0.72148503	Has2	1
GO:1904356	regulation of telomere maintenance via telomere lengthening	1/138	61/14464	0.01639344	1.71822286	0.55170119	0.44345823	0.73460294	0.72148503	Hnrnpc	1
GO:1990868	response to chemokine	1/138	61/14464	0.01639344	1.71822286	0.55170119	0.44345823	0.73460294	0.72148503	Oxsr1	1
GO:1990869	cellular response to chemokine	1/138	61/14464	0.01639344	1.71822286	0.55170119	0.44345823	0.73460294	0.72148503	Oxsr1	1
GO:0042330	taxis	4/138	357/14464	0.01120448	1.1743596	0.32739263	0.4444878	0.73510042	0.72197363	Calr/Arhgef16/Sema3e/Oxsr1	4
GO:0071692	protein localization to extracellular region	4/138	357/14464	0.01120448	1.1743596	0.32739263	0.4444878	0.73510042	0.72197363	Bmal1/Dand5/Smad2/Sri	4
GO:0000271	polysaccharide biosynthetic process	1/138	62/14464	0.01612903	1.69050958	0.53476185	0.44879064	0.73526893	0.72213913	Has2	1
GO:0002562	somatic diversification of immune receptors via germline recombination within a single locus	1/138	62/14464	0.01612903	1.69050958	0.53476185	0.44879064	0.73526893	0.72213913	Hspd1	1
GO:0006801	superoxide metabolic process	1/138	62/14464	0.01612903	1.69050958	0.53476185	0.44879064	0.73526893	0.72213913	Sh3pxd2a	1
GO:0008652	amino acid biosynthetic process	1/138	62/14464	0.01612903	1.69050958	0.53476185	0.44879064	0.73526893	0.72213913	Enoph1	1
GO:0016444	somatic cell DNA recombination	1/138	62/14464	0.01612903	1.69050958	0.53476185	0.44879064	0.73526893	0.72213913	Hspd1	1
GO:0042130	negative regulation of T cell proliferation	1/138	62/14464	0.01612903	1.69050958	0.53476185	0.44879064	0.73526893	0.72213913	Itch	1
GO:0042551	neuron maturation	1/138	62/14464	0.01612903	1.69050958	0.53476185	0.44879064	0.73526893	0.72213913	Kcnip2	1
GO:0051893	regulation of focal adhesion assembly	1/138	62/14464	0.01612903	1.69050958	0.53476185	0.44879064	0.73526893	0.72213913	Mmp14	1
GO:0090109	regulation of cell-substrate junction assembly	1/138	62/14464	0.01612903	1.69050958	0.53476185	0.44879064	0.73526893	0.72213913	Mmp14	1
GO:1903317	regulation of protein maturation	1/138	62/14464	0.01612903	1.69050958	0.53476185	0.44879064	0.73526893	0.72213913	Mmp14	1
GO:0030041	actin filament polymerization	2/138	159/14464	0.01257862	1.31838483	0.3961989	0.44961343	0.73526893	0.72213913	Scin/Msrb2	2
GO:0008544	epidermis development	3/138	259/14464	0.01158301	1.21403391	0.34112858	0.45041468	0.73526893	0.72213913	Grhl3/Ift172/Pkp3	3
GO:0032943	mononuclear cell proliferation	3/138	259/14464	0.01158301	1.21403391	0.34112858	0.45041468	0.73526893	0.72213913	Card11/Hspd1/Itch	3
GO:0071407	cellular response to organic cyclic compound	5/138	462/14464	0.01082251	1.13432461	0.2879969	0.45194584	0.73526893	0.72213913	Bmal1/Calr/Hsp90b1/Pdia3/Gclm	5
GO:0071356	cellular response to tumor necrosis factor	2/138	160/14464	0.0125	1.31014493	0.38717043	0.45281637	0.73526893	0.72213913	Has2/Ppp2cb	2
GO:0051251	positive regulation of lymphocyte activation	3/138	260/14464	0.01153846	1.20936455	0.33434186	0.45290912	0.73526893	0.72213913	Card11/Mmp14/Hspd1	3
GO:0003208	cardiac ventricle morphogenesis	1/138	63/14464	0.01587302	1.6636761	0.5181272	0.45407233	0.73526893	0.72213913	Pkp2	1
GO:0006081	cellular aldehyde metabolic process	1/138	63/14464	0.01587302	1.6636761	0.5181272	0.45407233	0.73526893	0.72213913	Akr1a1	1
GO:0006919	activation of cysteine-type endopeptidase activity involved in apoptotic process	1/138	63/14464	0.01587302	1.6636761	0.5181272	0.45407233	0.73526893	0.72213913	Hspd1	1
GO:0007585	respiratory gaseous exchange by respiratory system	1/138	63/14464	0.01587302	1.6636761	0.5181272	0.45407233	0.73526893	0.72213913	Slc5a3	1
GO:0030837	negative regulation of actin filament polymerization	1/138	63/14464	0.01587302	1.6636761	0.5181272	0.45407233	0.73526893	0.72213913	Scin	1
GO:0051453	regulation of intracellular pH	1/138	63/14464	0.01587302	1.6636761	0.5181272	0.45407233	0.73526893	0.72213913	Rab38	1
GO:0061035	regulation of cartilage development	1/138	63/14464	0.01587302	1.6636761	0.5181272	0.45407233	0.73526893	0.72213913	Scin	1
GO:0097352	autophagosome maturation	1/138	63/14464	0.01587302	1.6636761	0.5181272	0.45407233	0.73526893	0.72213913	Snx14	1
GO:0170041	non-proteinogenic amino acid metabolic process	1/138	63/14464	0.01587302	1.6636761	0.5181272	0.45407233	0.73526893	0.72213913	P4hb	1
GO:1901657	glycosyl compound metabolic process	1/138	63/14464	0.01587302	1.6636761	0.5181272	0.45407233	0.73526893	0.72213913	Akr1a1	1
GO:0006469	negative regulation of protein kinase activity	2/138	161/14464	0.01242236	1.30200738	0.37820145	0.45600881	0.73781201	0.7246368	Chordc1/Dnajc3	2
GO:0007369	gastrulation	2/138	162/14464	0.01234568	1.2939703	0.3692911	0.45919066	0.73892953	0.72573436	Mmp14/Smad2	2
GO:0021915	neural tube development	2/138	162/14464	0.01234568	1.2939703	0.3692911	0.45919066	0.73892953	0.72573436	Grhl3/Ift172	2
GO:0001541	ovarian follicle development	1/138	64/14464	0.015625	1.63768116	0.50178606	0.45930377	0.73892953	0.72573436	Mmp14	1
GO:0003407	neural retina development	1/138	64/14464	0.015625	1.63768116	0.50178606	0.45930377	0.73892953	0.72573436	Slc17a8	1
GO:0050885	neuromuscular process controlling balance	1/138	64/14464	0.015625	1.63768116	0.50178606	0.45930377	0.73892953	0.72573436	Hexa	1
GO:0060411	cardiac septum morphogenesis	1/138	64/14464	0.015625	1.63768116	0.50178606	0.45930377	0.73892953	0.72573436	Dhrs3	1
GO:0007005	mitochondrion organization	5/138	466/14464	0.01072961	1.12458792	0.26831328	0.45940536	0.73892953	0.72573436	Rab38/Hspd1/Gclm/S2bpcox16/Ppp2cb	5
GO:0002460	adaptive immune response based on somatic recombination of immune receptors built from immunoglobu	3/138	263/14464	0.01140684	1.19556952	0.31414188	0.46036483	0.73892953	0.72573436	Ighg2c/Hspd1/Cr1l	3
GO:0002790	peptide secretion	3/138	263/14464	0.01140684	1.19556952	0.31414188	0.46036483	0.73892953	0.72573436	Bmal1/Smad2/Sri	3
GO:0007162	negative regulation of cell adhesion	3/138	263/14464	0.01140684	1.19556952	0.31414188	0.46036483	0.73892953	0.72573436	Mmp14/Itch/Sema3e	3
GO:0010608	post-transcriptional regulation of gene expression	5/138	468/14464	0.01068376	1.11978199	0.25853464	0.46312492	0.74082489	0.72759587	Calr/Mettl16/Dnajc3/Hnrnpc/Mettl14	5
GO:0010324	membrane invagination	1/138	65/14464	0.01538462	1.61248606	0.48572783	0.46448545	0.74082489	0.72759587	Snx18	1
GO:0032007	negative regulation of TOR signaling	1/138	65/14464	0.01538462	1.61248606	0.48572783	0.46448545	0.74082489	0.72759587	Bmal1	1
GO:0032922	circadian regulation of gene expression	1/138	65/14464	0.01538462	1.61248606	0.48572783	0.46448545	0.74082489	0.72759587	Bmal1	1
GO:0043297	apical junction assembly	1/138	65/14464	0.01538462	1.61248606	0.48572783	0.46448545	0.74082489	0.72759587	Pkp2	1
GO:0051058	negative regulation of small GTPase mediated signal transduction	1/138	65/14464	0.01538462	1.61248606	0.48572783	0.46448545	0.74082489	0.72759587	Ppp2cb	1
GO:0061512	protein localization to cilium	1/138	65/14464	0.01538462	1.61248606	0.48572783	0.46448545	0.74082489	0.72759587	Ift80	1
GO:0140115	export across plasma membrane	1/138	65/14464	0.01538462	1.61248606	0.48572783	0.46448545	0.74082489	0.72759587	Kcnip2	1
GO:0002009	morphogenesis of an epithelium	5/138	470/14464	0.0106383	1.11501696	0.24879757	0.46683732	0.74398737	0.73070189	Mmp14/Grhl3/Stard13/Ift172/Sema3e	5
GO:0014911	positive regulation of smooth muscle cell migration	1/138	66/14464	0.01515152	1.58805446	0.46994247	0.46961782	0.74605951	0.73273702	Has2	1
GO:0031122	cytoplasmic microtubule organization	1/138	66/14464	0.01515152	1.58805446	0.46994247	0.46961782	0.74605951	0.73273702	Ift172	1
GO:0034248	regulation of amide metabolic process	1/138	66/14464	0.01515152	1.58805446	0.46994247	0.46961782	0.74605951	0.73273702	Pdk3	1
GO:0140962	multicellular organismal-level chemical homeostasis	1/138	66/14464	0.01515152	1.58805446	0.46994247	0.46961782	0.74605951	0.73273702	Has2	1
GO:0006766	vitamin metabolic process	1/138	67/14464	0.01492537	1.56435215	0.45442043	0.47470135	0.74894039	0.73556646	Akr1a1	1
GO:0033273	response to vitamin	1/138	67/14464	0.01492537	1.56435215	0.45442043	0.47470135	0.74894039	0.73556646	Pdia3	1
GO:0045123	cellular extravasation	1/138	67/14464	0.01492537	1.56435215	0.45442043	0.47470135	0.74894039	0.73556646	Cd99l2	1
GO:0060415	muscle tissue morphogenesis	1/138	67/14464	0.01492537	1.56435215	0.45442043	0.47470135	0.74894039	0.73556646	Pkp2	1
GO:0120193	tight junction organization	1/138	67/14464	0.01492537	1.56435215	0.45442043	0.47470135	0.74894039	0.73556646	Pkp2	1
GO:0150116	regulation of cell-substrate junction organization	1/138	67/14464	0.01492537	1.56435215	0.45442043	0.47470135	0.74894039	0.73556646	Mmp14	1
GO:2000036	regulation of stem cell population maintenance	1/138	67/14464	0.01492537	1.56435215	0.45442043	0.47470135	0.74894039	0.73556646	Brms1	1
GO:0045807	positive regulation of endocytosis	2/138	167/14464	0.01197605	1.25522867	0.32559033	0.474938	0.74894039	0.73556646	Calr/Lman2	2
GO:1903169	regulation of calcium ion transmembrane transport	2/138	167/14464	0.01197605	1.25522867	0.32559033	0.474938	0.74894039	0.73556646	Rrad/Sri	2
GO:0060070	canonical Wnt signaling pathway	3/138	269/14464	0.01115242	1.16890254	0.27444219	0.47514621	0.74894039	0.73556646	Bmal1/Fzd8/Frat2	3
GO:0006446	regulation of translational initiation	1/138	68/14464	0.01470588	1.54134697	0.43915266	0.47973651	0.75381825	0.74035721	Dnajc3	1
GO:0060395	SMAD protein signal transduction	1/138	68/14464	0.01470588	1.54134697	0.43915266	0.47973651	0.75381825	0.74035721	Smad2	1
GO:1901606	alpha-amino acid catabolic process	1/138	68/14464	0.01470588	1.54134697	0.43915266	0.47973651	0.75381825	0.74035721	Hmgcll1	1
GO:1902475	L-alpha-amino acid transmembrane transport	1/138	68/14464	0.01470588	1.54134697	0.43915266	0.47973651	0.75381825	0.74035721	Slc17a8	1
GO:0010959	regulation of metal ion transport	4/138	375/14464	0.01066667	1.11799034	0.22721043	0.48225799	0.75545283	0.7419626	Rrad/Kcnip2/Sri/Oxsr1	4
GO:0015833	peptide transport	3/138	272/14464	0.01102941	1.15601023	0.25493061	0.48246854	0.75545283	0.7419626	Bmal1/Smad2/Sri	3
GO:0002819	regulation of adaptive immune response	2/138	170/14464	0.01176471	1.23307758	0.30002285	0.48425397	0.75545283	0.7419626	Hspd1/Cr1l	2
GO:0030902	hindbrain development	2/138	170/14464	0.01176471	1.23307758	0.30002285	0.48425397	0.75545283	0.7419626	Hspa5/Fktn	2
GO:0002200	somatic diversification of immune receptors	1/138	69/14464	0.01449275	1.51900861	0.42413053	0.48472376	0.75545283	0.7419626	Hspd1	1
GO:0008333	endosome to lysosome transport	1/138	69/14464	0.01449275	1.51900861	0.42413053	0.48472376	0.75545283	0.7419626	Aktip	1
GO:0008347	glial cell migration	1/138	69/14464	0.01449275	1.51900861	0.42413053	0.48472376	0.75545283	0.7419626	Mmp14	1
GO:0008543	fibroblast growth factor receptor signaling pathway	1/138	69/14464	0.01449275	1.51900861	0.42413053	0.48472376	0.75545283	0.7419626	Ift80	1
GO:0030641	regulation of cellular pH	1/138	69/14464	0.01449275	1.51900861	0.42413053	0.48472376	0.75545283	0.7419626	Rab38	1
GO:2000243	positive regulation of reproductive process	1/138	69/14464	0.01449275	1.51900861	0.42413053	0.48472376	0.75545283	0.7419626	Lhfpl2	1
GO:0051258	protein polymerization	3/138	273/14464	0.01098901	1.15177576	0.24847546	0.48489879	0.75545283	0.7419626	Scin/Tppp3/Msrb2	3
GO:0001960	negative regulation of cytokine-mediated signaling pathway	1/138	70/14464	0.01428571	1.49730849	0.40934586	0.48966354	0.7593492	0.74578939	Ppp2cb	1
GO:0042698	ovulation cycle	1/138	70/14464	0.01428571	1.49730849	0.40934586	0.48966354	0.7593492	0.74578939	Has2	1
GO:0006066	alcohol metabolic process	3/138	275/14464	0.01090909	1.14339921	0.23563699	0.48974318	0.7593492	0.74578939	Dhrs3/Slc5a3/Akr1a1	3
GO:0031929	TOR signaling	2/138	172/14464	0.01162791	1.21873947	0.28323775	0.49040822	0.7593492	0.74578939	Bmal1/Card11	2
GO:0048736	appendage development	2/138	172/14464	0.01162791	1.21873947	0.28323775	0.49040822	0.7593492	0.74578939	Ift172/Ift80	2
GO:0060173	limb development	2/138	172/14464	0.01162791	1.21873947	0.28323775	0.49040822	0.7593492	0.74578939	Ift172/Ift80	2
GO:2001242	regulation of intrinsic apoptotic signaling pathway	2/138	172/14464	0.01162791	1.21873947	0.28323775	0.49040822	0.7593492	0.74578939	Bag5/P4hb	2
GO:2001233	regulation of apoptotic signaling pathway	4/138	379/14464	0.01055409	1.10619097	0.20560564	0.49052126	0.7593492	0.74578939	Pdia3/Gclm/Bag5/P4hb	4
GO:0016197	endosomal transport	3/138	276/14464	0.01086957	1.13925646	0.22925328	0.49215721	0.7593492	0.74578939	Rab9b/Snx18/Aktip	3
GO:0030198	extracellular matrix organization	3/138	276/14464	0.01086957	1.13925646	0.22925328	0.49215721	0.7593492	0.74578939	Mmp14/Has2/Fktn	3
GO:0043062	extracellular structure organization	3/138	276/14464	0.01086957	1.13925646	0.22925328	0.49215721	0.7593492	0.74578939	Mmp14/Has2/Fktn	3
GO:0045229	external encapsulating structure organization	3/138	276/14464	0.01086957	1.13925646	0.22925328	0.49215721	0.7593492	0.74578939	Mmp14/Has2/Fktn	3
GO:0033673	negative regulation of kinase activity	2/138	173/14464	0.01156069	1.21169473	0.27492109	0.49346822	0.7593492	0.74578939	Chordc1/Dnajc3	2
GO:0043393	regulation of protein binding	2/138	173/14464	0.01156069	1.21169473	0.27492109	0.49346822	0.7593492	0.74578939	Fktn/Aktip	2
GO:0034329	cell junction assembly	5/138	485/14464	0.01030928	1.0805319	0.17705452	0.49442538	0.7593492	0.74578939	Mmp14/Lrrn1/Pkp3/Pkp2/Cdh10	5
GO:0003281	ventricular septum development	1/138	71/14464	0.01408451	1.47621964	0.39479085	0.4945563	0.7593492	0.74578939	Dand5	1
GO:0071347	cellular response to interleukin-1	1/138	71/14464	0.01408451	1.47621964	0.39479085	0.4945563	0.7593492	0.74578939	Has2	1
GO:1903051	negative regulation of proteolysis involved in protein catabolic process	1/138	71/14464	0.01408451	1.47621964	0.39479085	0.4945563	0.7593492	0.74578939	Bag5	1
GO:2000379	positive regulation of reactive oxygen species metabolic process	1/138	71/14464	0.01408451	1.47621964	0.39479085	0.4945563	0.7593492	0.74578939	Slc5a3	1
GO:0043484	regulation of RNA splicing	2/138	174/14464	0.01149425	1.20473097	0.26665413	0.49651672	0.76060574	0.74702349	Mettl16/Obi1	2
GO:0003151	outflow tract morphogenesis	1/138	72/14464	0.01388889	1.45571659	0.38045804	0.49940249	0.76060574	0.74702349	Dhrs3	1
GO:0007032	endosome organization	1/138	72/14464	0.01388889	1.45571659	0.38045804	0.49940249	0.76060574	0.74702349	Aktip	1
GO:0010833	telomere maintenance via telomere lengthening	1/138	72/14464	0.01388889	1.45571659	0.38045804	0.49940249	0.76060574	0.74702349	Hnrnpc	1
GO:0014032	neural crest cell development	1/138	72/14464	0.01388889	1.45571659	0.38045804	0.49940249	0.76060574	0.74702349	Sema3e	1
GO:0015800	acidic amino acid transport	1/138	72/14464	0.01388889	1.45571659	0.38045804	0.49940249	0.76060574	0.74702349	Slc17a8	1
GO:0019730	antimicrobial humoral response	1/138	72/14464	0.01388889	1.45571659	0.38045804	0.49940249	0.76060574	0.74702349	Ighg2c	1
GO:0051149	positive regulation of muscle cell differentiation	1/138	72/14464	0.01388889	1.45571659	0.38045804	0.49940249	0.76060574	0.74702349	Mmp14	1
GO:0021953	central nervous system neuron differentiation	2/138	175/14464	0.01142857	1.19784679	0.25843622	0.49955366	0.76060574	0.74702349	Ift172/Sema3e	2
GO:0046777	protein autophosphorylation	2/138	175/14464	0.01142857	1.19784679	0.25843622	0.49955366	0.76060574	0.74702349	Stk16/Oxsr1	2
GO:0050920	regulation of chemotaxis	2/138	175/14464	0.01142857	1.19784679	0.25843622	0.49955366	0.76060574	0.74702349	Calr/Oxsr1	2
GO:0050954	sensory perception of mechanical stimulus	2/138	176/14464	0.01136364	1.19104084	0.25026672	0.50257898	0.76060574	0.74702349	Slc17a8/Hexa	2
GO:0016055	Wnt signaling pathway	4/138	385/14464	0.01038961	1.08895163	0.17362195	0.50281582	0.76060574	0.74702349	Bmal1/Fzd8/Frat2/Ift80	4
GO:0006936	muscle contraction	3/138	281/14464	0.01067616	1.11898499	0.1976822	0.50414356	0.76060574	0.74702349	Kcnip2/Pkp2/Sri	3

GO:0032092	positive regulation of protein binding	1/138	73/14464	0.01369863	1.43577526	0.36634037	0.50420255	0.76060574	0.74702349	Aktip	1
GO:0032481	positive regulation of type I interferon production	1/138	73/14464	0.01369863	1.43577526	0.36634037	0.50420255	0.76060574	0.74702349	Hspd1	1
GO:0035050	embryonic heart tube development	1/138	73/14464	0.01369863	1.43577526	0.36634037	0.50420255	0.76060574	0.74702349	Ift172	1
GO:0045665	negative regulation of neuron differentiation	1/138	73/14464	0.01369863	1.43577526	0.36634037	0.50420255	0.76060574	0.74702349	Calr	1
GO:0048644	muscle organ morphogenesis	1/138	73/14464	0.01369863	1.43577526	0.36634037	0.50420255	0.76060574	0.74702349	Pkp2	1
GO:1902476	chloride transmembrane transport	1/138	73/14464	0.01369863	1.43577526	0.36634037	0.50420255	0.76060574	0.74702349	Slc17a8	1
GO:0006352	DNA-templated transcription initiation	2/138	177/14464	0.01129944	1.1843118	0.242145	0.50559263	0.76060574	0.74702349	Tbpl1/Tfb1m	2
GO:0010970	transport along microtubule	2/138	177/14464	0.01129944	1.1843118	0.242145	0.50559263	0.76060574	0.74702349	Ift172/Ift80	2
GO:0061138	morphogenesis of a branching epithelium	2/138	177/14464	0.01129944	1.1843118	0.242145	0.50559263	0.76060574	0.74702349	Mmp14/Sema3e	2
GO:0001764	neuron migration	2/138	178/14464	0.01123596	1.17765836	0.23407045	0.50859456	0.76060574	0.74702349	Fktn/Sema3e	2
GO:0045619	regulation of lymphocyte differentiation	2/138	178/14464	0.01123596	1.17765836	0.23407045	0.50859456	0.76060574	0.74702349	Card11/Mmp14	2
GO:0002696	positive regulation of leukocyte activation	3/138	283/14464	0.01060071	1.11107697	0.18521241	0.50889806	0.76060574	0.74702349	Card11/Mmp14/Hspd1	3
GO:0043523	regulation of neuron apoptotic process	3/138	283/14464	0.01060071	1.11107697	0.18521241	0.50889806	0.76060574	0.74702349	Hspd1/Gclm/Sema3e	3
GO:0031329	regulation of cellular catabolic process	4/138	388/14464	0.01030928	1.0805319	0.15781578	0.50891571	0.76060574	0.74702349	Snx18/Itch/Rmc1/Sec22b	4
GO:0002312	B cell activation involved in immune response	1/138	74/14464	0.01351351	1.41637289	0.35243106	0.50895691	0.76060574	0.74702349	Hspd1	1
GO:0006690	icosanoid metabolic process	1/138	74/14464	0.01351351	1.41637289	0.35243106	0.50895691	0.76060574	0.74702349	Lta4h	1
GO:0014823	response to activity	1/138	74/14464	0.01351351	1.41637289	0.35243106	0.50895691	0.76060574	0.74702349	Gclm	1
GO:0042102	positive regulation of T cell proliferation	1/138	74/14464	0.01351351	1.41637289	0.35243106	0.50895691	0.76060574	0.74702349	Card11	1
GO:0045069	regulation of viral genome replication	1/138	74/14464	0.01351351	1.41637289	0.35243106	0.50895691	0.76060574	0.74702349	Srpk1	1
GO:0051279	regulation of release of sequestered calcium ion into cytosol	1/138	74/14464	0.01351351	1.41637289	0.35243106	0.50895691	0.76060574	0.74702349	Sri	1
GO:0051298	centrosome duplication	1/138	74/14464	0.01351351	1.41637289	0.35243106	0.50895691	0.76060574	0.74702349	Chordc1	1
GO:0060761	negative regulation of response to cytokine stimulus	1/138	74/14464	0.01351351	1.41637289	0.35243106	0.50895691	0.76060574	0.74702349	Ppp2cb	1
GO:0019827	stem cell population maintenance	2/138	179/14464	0.01117318	1.17107926	0.22604246	0.5115847	0.76283636	0.74921428	Brms1/Mettl14	2
GO:0030595	leukocyte chemotaxis	2/138	179/14464	0.01117318	1.17107926	0.22604246	0.5115847	0.76283636	0.74921428	Calr/Oxsr1	2
GO:0050796	regulation of insulin secretion	2/138	179/14464	0.01117318	1.17107926	0.22604246	0.5115847	0.76283636	0.74921428	Bmal1/Sri	2
GO:0002088	lens development in camera-type eye	1/138	75/14464	0.01333333	1.39748792	0.33872365	0.51366601	0.76368044	0.75004329	Spred3	1
GO:0002709	regulation of T cell mediated immunity	1/138	75/14464	0.01333333	1.39748792	0.33872365	0.51366601	0.76368044	0.75004329	Hspd1	1
GO:0006626	protein targeting to mitochondrion	1/138	75/14464	0.01333333	1.39748792	0.33872365	0.51366601	0.76368044	0.75004329	Hspd1	1
GO:0050672	negative regulation of lymphocyte proliferation	1/138	75/14464	0.01333333	1.39748792	0.33872365	0.51366601	0.76368044	0.75004329	Itch	1
GO:0034612	response to tumor necrosis factor	2/138	180/14464	0.01111111	1.16457327	0.21806044	0.51456302	0.76388737	0.75024652	Has2/Ppp2cb	2
GO:0035265	organ growth	2/138	180/14464	0.01111111	1.16457327	0.21806044	0.51456302	0.76388737	0.75024652	Smad2/Ift80	2
GO:0048839	inner ear development	2/138	181/14464	0.01104972	1.15813916	0.21012381	0.51752946	0.76772582	0.75401643	Grhl3/Slc17a8	2
GO:0032945	negative regulation of mononuclear cell proliferation	1/138	76/14464	0.01315789	1.37909992	0.32521198	0.51833028	0.76779282	0.75408223	Itch	1
GO:0070661	leukocyte proliferation	3/138	287/14464	0.01045296	1.09559158	0.16053682	0.51833632	0.76779282	0.75408223	Card11/Hspd1/Itch	3
GO:0071396	cellular response to lipid	5/138	499/14464	0.01002004	1.05021637	0.11204215	0.51970414	0.76855635	0.75483213	Bmal1/Calr/Pdia3/Sash1/Pdk3	5
GO:1901888	regulation of cell junction assembly	3/138	288/14464	0.01041667	1.09178744	0.15442191	0.52068088	0.76855635	0.75483213	Mmp14/Lrrn1/Pkp2	3
GO:0032890	regulation of organic acid transport	1/138	77/14464	0.01298701	1.36118954	0.31189015	0.52295013	0.76855635	0.75483213	Slc17a8	1
GO:0033559	unsaturated fatty acid metabolic process	1/138	77/14464	0.01298701	1.36118954	0.31189015	0.52295013	0.76855635	0.75483213	Elovl7	1
GO:0043112	receptor metabolic process	1/138	77/14464	0.01298701	1.36118954	0.31189015	0.52295013	0.76855635	0.75483213	Itch	1
GO:0061178	regulation of insulin secretion involved in cellular response to glucose stimulus	1/138	77/14464	0.01298701	1.36118954	0.31189015	0.52295013	0.76855635	0.75483213	Sri	1
GO:1901880	negative regulation of protein depolymerization	1/138	77/14464	0.01298701	1.36118954	0.31189015	0.52295013	0.76855635	0.75483213	Scin	1
GO:0002520	immune system development	2/138	183/14464	0.01092896	1.1454819	0.19438443	0.52342652	0.76855635	0.75483213	Hspd1/Itch	2
GO:0008154	actin polymerization or depolymerization	2/138	183/14464	0.01092896	1.1454819	0.19438443	0.52342652	0.76855635	0.75483213	Scin/Msrb2	2
GO:0009267	cellular response to starvation	2/138	183/14464	0.01092896	1.1454819	0.19438443	0.52342652	0.76855635	0.75483213	Hspa5/Wdr45b	2
GO:0042180	cellular ketone metabolic process	2/138	183/14464	0.01092896	1.1454819	0.19438443	0.52342652	0.76855635	0.75483213	Pdk3/Akr1a1	2
GO:0071383	cellular response to steroid hormone stimulus	2/138	183/14464	0.01092896	1.1454819	0.19438443	0.52342652	0.76855635	0.75483213	Bmal1/Calr	2
GO:0007519	skeletal muscle tissue development	2/138	184/14464	0.01086957	1.13925646	0.18658057	0.52635706	0.77064666	0.75688511	Bmal1/Fktn	2
GO:0007422	peripheral nervous system development	1/138	78/14464	0.01282051	1.34373839	0.29875252	0.52752599	0.77064666	0.75688511	Slc5a3	1
GO:0031341	regulation of cell killing	1/138	78/14464	0.01282051	1.34373839	0.29875252	0.52752599	0.77064666	0.75688511	Cr1l	1
GO:0032649	regulation of type II interferon production	1/138	78/14464	0.01282051	1.34373839	0.29875252	0.52752599	0.77064666	0.75688511	Hspd1	1
GO:0035023	regulation of Rho protein signal transduction	1/138	78/14464	0.01282051	1.34373839	0.29875252	0.52752599	0.77064666	0.75688511	Stard13	1
GO:0045995	regulation of embryonic development	1/138	78/14464	0.01282051	1.34373839	0.29875252	0.52752599	0.77064666	0.75688511	Hspa5	1
GO:0048864	stem cell development	1/138	78/14464	0.01282051	1.34373839	0.29875252	0.52752599	0.77064666	0.75688511	Sema3e	1
GO:0042098	T cell proliferation	2/138	185/14464	0.01081081	1.13309832	0.17881988	0.52927556	0.77152532	0.75774808	Card11/Itch	2
GO:0045664	regulation of neuron differentiation	2/138	185/14464	0.01081081	1.13309832	0.17881988	0.52927556	0.77152532	0.75774808	Calr/Mettl14	2
GO:0098727	maintenance of cell number	2/138	185/14464	0.01081081	1.13309832	0.17881988	0.52927556	0.77152532	0.75774808	Brms1/Mettl14	2
GO:0007405	neuroblast proliferation	1/138	79/14464	0.01265823	1.32672904	0.28579367	0.53205827	0.77185528	0.75807215	Tafa1	1
GO:0009063	amino acid catabolic process	1/138	79/14464	0.01265823	1.32672904	0.28579367	0.53205827	0.77185528	0.75807215	Hmgcll1	1
GO:0031397	negative regulation of protein ubiquitination	1/138	79/14464	0.01265823	1.32672904	0.28579367	0.53205827	0.77185528	0.75807215	Bag5	1
GO:0032272	negative regulation of protein polymerization	1/138	79/14464	0.01265823	1.32672904	0.28579367	0.53205827	0.77185528	0.75807215	Scin	1
GO:0051965	positive regulation of synapse assembly	1/138	79/14464	0.01265823	1.32672904	0.28579367	0.53205827	0.77185528	0.75807215	Lrrn1	1
GO:0097009	energy homeostasis	1/138	79/14464	0.01265823	1.32672904	0.28579367	0.53205827	0.77185528	0.75807215	Bmal1	1
GO:0051216	cartilage development	2/138	186/14464	0.01075269	1.12700639	0.17110181	0.53218196	0.77185528	0.75807215	Scin/Ift80	2
GO:0050679	positive regulation of epithelial cell proliferation	2/138	187/14464	0.01069519	1.12097962	0.16342586	0.53507624	0.77549512	0.76164699	Has2/Aggf1	2
GO:0006885	regulation of pH	1/138	80/14464	0.0125	1.31014493	0.27300846	0.53654739	0.77593606	0.76208006	Rab38	1
GO:0010507	negative regulation of autophagy	1/138	80/14464	0.0125	1.31014493	0.27300846	0.53654739	0.77593606	0.76208006	Sec22b	1
GO:0071230	cellular response to amino acid stimulus	1/138	80/14464	0.0125	1.31014493	0.27300846	0.53654739	0.77593606	0.76208006	Gclm	1
GO:0010506	regulation of autophagy	3/138	295/14464	0.01016949	1.06588062	0.11220323	0.53692004	0.77593606	0.76208006	Snx18/Rmc1/Sec22b	3
GO:0050870	positive regulation of T cell activation	2/138	188/14464	0.0106383	1.11501696	0.15579152	0.53795837	0.77687971	0.76300685	Card11/Hspd1	2
GO:0044282	small molecule catabolic process	3/138	296/14464	0.01013514	1.06227967	0.10625372	0.53921476	0.77813669	0.7642414	Hexa/Hmgcll1/Akr1a1	3
GO:0007601	visual perception	1/138	81/14464	0.01234568	1.2939703	0.26039191	0.54099375	0.77847494	0.76457361	Rbp1	1
GO:0045824	negative regulation of innate immune response	1/138	81/14464	0.01234568	1.2939703	0.26039191	0.54099375	0.77847494	0.76457361	Dtx4	1
GO:0070664	negative regulation of leukocyte proliferation	1/138	81/14464	0.01234568	1.2939703	0.26039191	0.54099375	0.77847494	0.76457361	Itch	1
GO:1904427	positive regulation of calcium ion transmembrane transport	1/138	81/14464	0.01234568	1.2939703	0.26039191	0.54099375	0.77847494	0.76457361	Sri	1
GO:0050863	regulation of T cell activation	3/138	297/14464	0.01010101	1.05870297	0.10032418	0.54150308	0.77865207	0.76474757	Card11/Hspd1/Itch	3
GO:0006029	proteoglycan metabolic process	1/138	82/14464	0.01219512	1.27819017	0.24793928	0.54539776	0.78257785	0.76860325	Hexa	1
GO:0006576	biogenic amine metabolic process	1/138	82/14464	0.01219512	1.27819017	0.24793928	0.54539776	0.78257785	0.76860325	Sat1	1
GO:0033209	tumor necrosis factor-mediated signaling pathway	1/138	82/14464	0.01219512	1.27819017	0.24793928	0.54539776	0.78257785	0.76860325	Ppp2cb	1
GO:0032271	regulation of protein polymerization	2/138	192/14464	0.01041667	1.09178744	0.12566019	0.54936465	0.78548249	0.77145601	Scin/Tppp3	2
GO:0045732	positive regulation of protein catabolic process	2/138	192/14464	0.01041667	1.09178744	0.12566019	0.54936465	0.78548249	0.77145601	Itch/Sec22b	2
GO:0050670	regulation of lymphocyte proliferation	2/138	192/14464	0.01041667	1.09178744	0.12566019	0.54936465	0.78548249	0.77145601	Card11/Itch	2
GO:0014033	neural crest cell differentiation	1/138	83/14464	0.01204819	1.26279029	0.23564601	0.54975981	0.78548249	0.77145601	Sema3e	1
GO:0043648	dicarboxylic acid metabolic process	1/138	83/14464	0.01204819	1.26279029	0.23564601	0.54975981	0.78548249	0.77145601	Gclm	1
GO:0050953	sensory perception of light stimulus	1/138	83/14464	0.01204819	1.26279029	0.23564601	0.54975981	0.78548249	0.77145601	Rbp1	1
GO:0051249	regulation of lymphocyte activation	4/138	409/14464	0.00977995	1.02505227	0.05044284	0.55064307	0.78618727	0.77214822	Card11/Mmp14/Hspd1/Itch	4
GO:0005977	glycogen metabolic process	1/138	84/14464	0.01190476	1.24775707	0.22350772	0.55408032	0.78641539	0.77237225	Wdr45b	1
GO:0006094	gluconeogenesis	1/138	84/14464	0.01190476	1.24775707	0.22350772	0.55408032	0.78641539	0.77237225	Arpp19	1
GO:0016579	protein deubiquitination	1/138	84/14464	0.01190476	1.24775707	0.22350772	0.55408032	0.78641539	0.77237225	Itch	1
GO:0018107	peptidyl-threonine phosphorylation	1/138	84/14464	0.01190476	1.24775707	0.22350772	0.55408032	0.78641539	0.77237225	Oxsr1	1
GO:0042157	lipoprotein metabolic process	1/138	84/14464	0.01190476	1.24775707	0.22350772	0.55408032	0.78641539	0.77237225	Wdr45b	1
GO:0071901	negative regulation of protein serine/threonine kinase activity	1/138	84/14464	0.01190476	1.24775707	0.22350772	0.55408032	0.78641539	0.77237225	Chordc1	1
GO:0001763	morphogenesis of a branching structure	2/138	194/14464	0.01030928	1.0805319	0.11083147	0.55499407	0.78641539	0.77237225	Mmp14/Sema3e	2
GO:0006486	protein glycosylation	2/138	194/14464	0.01030928	1.0805319	0.11083147	0.55499407	0.78641539	0.77237225	Fktn/Crppa	2
GO:0006909	phagocytosis	2/138	194/14464	0.01030928	1.0805319	0.11083147	0.55499407	0.78641539	0.77237225	Calr/Lman2	2
GO:0043413	macromolecule glycosylation	2/138	194/14464	0.01030928	1.0805319	0.11083147	0.55499407	0.78641539	0.77237225	Fktn/Crppa	2
GO:0050867	positive regulation of cell activation	3/138	303/14464	0.00990099	1.03773856	0.06515772	0.55509654	0.78641539	0.77237225	Card11/Mmp14/Hspd1	3
GO:0003073	regulation of systemic arterial blood pressure	1/138	85/14464	0.01176471	1.23307758	0.21152022	0.55835966	0.78641539	0.77237225	Manf	1
GO:0019915	lipid storage	1/138	85/14464	0.01176471	1.23307758	0.21152022	0.55835966	0.78641539	0.77237225	Hexa	1
GO:0044042	glucan metabolic process	1/138	85/14464	0.01176471	1.23307758	0.21152022	0.55835966	0.78641539	0.77237225	Wdr45b	1
GO:0048041	focal adhesion assembly	1/138	85/14464	0.01176471	1.23307758	0.21152022	0.55835966	0.78641539	0.77237225	Mmp14	1
GO:0014706	striated muscle tissue development	4/138	414/14464	0.00966184	1.01267241	0.025676	0.56030805	0.78641539	0.77237225	Bmal1/Calr/Fktn/Pkp2	4
GO:0032944	regulation of mononuclear cell proliferation	2/138	196/14464	0.01020408	1.06950606	0.09615579	0.56057408	0.78641539	0.77237225	Card11/Itch	2
GO:0097529	myeloid leukocyte migration	2/138	196/14464	0.01020408	1.06950606	0.09615579	0.56057408	0.78641539	0.77237225	Mmp14/Cd99l2	2
GO:0051051	negative regulation of transport	4/138	415/14464	0.00963855	1.01023223	0.02075764	0.56222782	0.78641539	0.77237225	Rrad/Sri/Ndfip2/Oxsr1	4
GO:0001570	vasculogenesis	1/138	86/14464	0.01162791	1.21873947	0.19967946	0.56259823	0.78641539	0.77237225	Has2	1
GO:0001938	positive regulation of endothelial cell proliferation	1/138	86/14464	0.01162791	1.21873947	0.19967946	0.56259823	0.78641539	0.77237225	Aggf1	1
GO:0006821	chloride transport	1/138	86/14464	0.01162791	1.21873947	0.19967946	0.56259823	0.78641539	0.77237225	Slc17a8	1
GO:0030168	platelet activation	1/138	86/14464	0.01162791	1.21873947	0.19967946	0.56259823	0.78641539	0.77237225	Pdia3	1
GO:0032273	positive regulation of protein polymerization	1/138	86/14464	0.01162791	1.21873947	0.19967946	0.56259823	0.78641539	0.77237225	Tppp3	1
GO:0032609	type II interferon production	1/138	86/14464	0.01162791	1.21873947	0.19967946	0.56259823	0.78641539	0.77237225	Hspd1	1
GO:0043242	negative regulation of protein-containing complex disassembly	1/138	86/14464	0.01162791	1.21873947	0.19967946	0.56259823	0.78641539	0.77237225	Scin	1
GO:2001237	negative regulation of extrinsic apoptotic signaling pathway	1/138	86/14464	0.01162791	1.21873947	0.19967946	0.56259823	0.78641539	0.77237225	Gclm	1
GO:0002703	regulation of leukocyte mediated immunity	2/138	197/14464	0.01015228	1.0640771	0.08887424	0.5633455	0.78641539	0.77237225	Hspd1/Cr1l	2
GO:0006006	glucose metabolic process	2/138	197/14464	0.01015228	1.0640771	0.08887424	0.5633455	0.78641539	0.77237225	Pdk3/Arpp19	2
GO:0060538	skeletal muscle organ development	2/138	197/14464	0.01015228	1.0640771	0.08887424	0.5633455	0.78641539	0.77237225	Bmal1/Fktn	2
GO:1901615	organic hydroxy compound metabolic process	4/138	417/14464	0.00959233	1.00538699	0.01095542	0.56605395	0.78641539	0.77237225	Dhrs3/Slc5a3/Rab38/Akr1a1	4
GO:0030705	cytoskeleton-dependent intracellular transport	2/138	198/14464	0.01010101	1.05870297	0.08162963	0.56610451	0.78641539	0.77237225	Ift172/Ift80	2
GO:0000281	mitotic cytokinesis	1/138	87/14464	0.01149425	1.20473097	0.18798158	0.56679641	0.78641539	0.77237225	Snx18	1
GO:0006661	phosphatidylinositol biosynthetic process	1/138	87/14464	0.01149425	1.20473097	0.18798158	0.56679641	0.78641539	0.77237225	Pigg	1
GO:0009408	response to heat	1/138	87/14464	0.01149425	1.20473097	0.18798158	0.56679641	0.78641539	0.77237225	Chordc1	1
GO:0015807	L-amino acid transport	1/138	87/14464	0.01149425	1.20473097	0.18798158	0.56679641	0.78641539	0.77237225	Slc17a8	1
GO:0032755	positive regulation of interleukin-6 production	1/138	87/14464	0.01149425	1.20473097	0.18798158	0.56679641	0.78641539	0.77237225	Hspd1	1
GO:0035773	insulin secretion involved in cellular response to glucose stimulus	1/138	87/14464	0.01149425	1.20473097	0.18798158	0.56679641	0.78641539	0.77237225	Sri	1
GO:0051881	regulation of mitochondrial membrane potential	1/138	87/14464	0.01149425	1.20473097	0.18798158	0.56679641	0.78641539	0.77237225	Gclm	1
GO:0090288	negative regulation of cellular response to growth factor stimulus	1/138	87/14464	0.01149425	1.20473097	0.18798158	0.56679641	0.78641539	0.77237225	Dand5	1
GO:1902117	positive regulation of organelle assembly	1/138	87/14464	0.01149425	1.20473097	0.18798158	0.56679641	0.78641539	0.77237225	Snx18	1
GO:0006835	dicarboxylic acid transport	1/138	88/14464	0.01136364	1.19104084	0.17642283	0.57095458	0.78748612	0.77342387	Slc17a8	1
GO:0019319	hexose biosynthetic process	1/138	88/14464	0.01136364	1.19104084	0.17642283	0.57095458	0.78748612	0.77342387	Arpp19	1
GO:0043543	protein acylation	1/138	88/14464	0.01136364	1.19104084	0.17642283	0.57095458	0.78748612	0.77342387	Bmal1	1
GO:0051153	regulation of striated muscle cell differentiation	1/138	88/14464	0.01136364	1.19104084	0.17642283	0.57095458	0.78748612	0.77342387	Mmp14	1
GO:0120254	olefinic compound metabolic process	1/138	88/14464	0.01136364	1.19104084	0.17642283	0.57095458	0.78748612	0.77342387	Dhrs3	1
GO:1990748	cellular detoxification	1/138	88/14464	0.01136364	1.19104084	0.17642283	0.57095458	0.78748612	0.77342387	Akr1a1	1
GO:0007264	small GTPase-mediated signal transduction	4/138	420/14464	0.00952381	0.99820566	-0.0036626	0.57175924	0.78748612	0.77342387	Rhoj/Stard13/Rab9b/Ppp2cb	4
GO:0010038	response to metal ion	3/138	311/14464	0.0096463	1.01104432	0.01932418	0.57284834	0.78748612	0.77342387	Calr/Lta4h/Ppp2cb	3
GO:0051146	striated muscle cell differentiation	3/138	311/14464	0.0096463	1.01104432	0.01932418	0.57284834	0.78748612	0.77342387	Calr/Mmp14/Fktn	3
GO:0048568	embryonic organ development	4/138	421/14464	0.00950119	0.99583462	-0.0085128	0.57365186	0.78748612	0.77342387	Mmp14/Grhl3/Smad2/Ift172	4
GO:0007156	homophilic cell adhesion via plasma membrane adhesion molecules	1/138	89/14464	0.01123596	1.17765836	0.16499964	0.57507313	0.78748612	0.77342387	Cdh10	1
GO:0019217	regulation of fatty acid metabolic process	1/138	89/14464	0.01123596	1.17765836	0.16499964	0.57507313	0.78748612	0.77342387	Pdk3	1
GO:0021954	central nervous system neuron development	1/138	89/14464	0.01123596	1.17765836	0.16499964	0.57507313	0.78748612	0.77342387	Sema3e	1
GO:0042472	inner ear morphogenesis	1/138	89/14464	0.01123596	1.17765836	0.16499964	0.57507313	0.78748612	0.77342387	Grhl3	1
GO:0098661	inorganic anion transmembrane transport	1/138	89/14464	0.01123596	1.17765836	0.16499964	0.57507313	0.78748612	0.77342387	Slc17a8	1
GO:1903321	negative regulation of protein modification by small protein conjugation or removal	1/138	89/14464	0.01123596	1.17765836	0.16499964	0.57507313	0.78748612	0.77342387	Bag5	1
GO:2001251	negative regulation of chromosome organization	1/138	89/14464	0.01123596	1.17765836	0.16499964	0.57507313	0.78748612	0.77342387	Hnrnpc	1
GO:0007283	spermatogenesis	4/138	423/14464	0.00945626	0.99112619	-0.0181797	0.57742327	0.78748612	0.77342387	Bmal1/Calr/Tbpl1/Mettl14	4
GO:0001657	ureteric bud development	1/138	90/14464	0.01111111	1.16457327	0.15370854	0.57915243	0.78748612	0.77342387	Smad2	1
GO:0001942	hair follicle development	1/138	90/14464	0.01111111	1.16457327	0.15370854	0.57915243	0.78748612	0.77342387	Pkp3	1
GO:0008589	regulation of smoothened signaling pathway	1/138	90/14464	0.01111111	1.16457327	0.15370854	0.57915243	0.78748612	0.77342387	Ift172	1
GO:0019233	sensory perception of pain	1/138	90/14464	0.01111111	1.16457327	0.15370854	0.57915243	0.78748612	0.77342387	Smr2l	1
GO:0048525	negative regulation of viral process	1/138	90/14464	0.01111111	1.16457327	0.15370854	0.57915243	0.78748612	0.77342387	Srpk1	1
GO:0051101	regulation of DNA binding	1/138	90/14464	0.01111111	1.16457327	0.15370854	0.57915243	0.78748612	0.77342387	Sri	1
GO:0051702	biological process involved in interaction with symbiont	1/138	90/14464	0.01111111	1.16457327	0.15370854	0.57915243	0.78748612	0.77342387	Hspd1	1
GO:0070555	response to interleukin-1	1/138	90/14464	0.01111111	1.16457327	0.15370854	0.57915243	0.78748612	0.77342387	Has2	1
GO:0046879	hormone secretion	3/138	314/14464	0.00955414	1.00138466	0.00243464	0.57939271	0.78748612	0.77342387	Bmal1/Smad2/Sri	3
GO:0045137	development of primary sexual characteristics	2/138	203/14464	0.00985222	1.03262654	0.04594628	0.5797128	0.78748612	0.77342387	Mmp14/Lhfpl2	2
GO:0045859	regulation of protein kinase activity	4/138	426/14464	0.00938967	0.98414642	-0.0325976	0.58304547	0.78748612	0.77342387	Chordc1/Fzd8/Sash1/Dnajc3	4
GO:0006970	response to osmotic stress	1/138	91/14464	0.01098901	1.15177576	0.14254621	0.58319285	0.78748612	0.77342387	Oxsr1	1
GO:0010469	regulation of signaling receptor activity	1/138	91/14464	0.01098901	1.15177576	0.14254621	0.58319285	0.78748612	0.77342387	Tafa1	1
GO:0019751	polyol metabolic process	1/138	91/14464	0.01098901	1.15177576	0.14254621	0.58319285	0.78748612	0.77342387	Slc5a3	1
GO:0042310	vasoconstriction	1/138	91/14464	0.01098901	1.15177576	0.14254621	0.58319285	0.78748612	0.77342387	Manf	1
GO:0046718	symbiont entry into host cell	1/138	91/14464	0.01098901	1.15177576	0.14254621	0.58319285	0.78748612	0.77342387	P4hb	1
GO:0046785	microtubule polymerization	1/138	91/14464	0.01098901	1.15177576	0.14254621	0.58319285	0.78748612	0.77342387	Tppp3	1
GO:0051952	regulation of amine transport	1/138	91/14464	0.01098901	1.15177576	0.14254621	0.58319285	0.78748612	0.77342387	Slc17a8	1
GO:0071229	cellular response to acid chemical	1/138	91/14464	0.01098901	1.15177576	0.14254621	0.58319285	0.78748612	0.77342387	Gclm	1
GO:0072163	mesonephric epithelium development	1/138	91/14464	0.01098901	1.15177576	0.14254621	0.58319285	0.78748612	0.77342387	Smad2	1
GO:0072164	mesonephric tubule development	1/138	91/14464	0.01098901	1.15177576	0.14254621	0.58319285	0.78748612	0.77342387	Smad2	1
GO:0090100	positive regulation of transmembrane receptor protein serine/threonine kinase signaling pathway	1/138	91/14464	0.01098901	1.15177576	0.14254621	0.58319285	0.78748612	0.77342387	Smad2	1

GO:0071216 cellular response to biotic stimulus	2/138	205/14464	0.0097561	1.02255214	0.03191732	0.58506873	0.78769039	0.77362449	Hspa5/Sash1	2
GO:0001823 mesonephros development	1/138	92/14464	0.01086957	1.13925646	0.13150944	0.58719475	0.78769039	0.77362449	Smad2	1
GO:0042147 retrograde transport, endosome to Golgi	1/138	92/14464	0.01086957	1.13925646	0.13150944	0.58719475	0.78769039	0.77362449	Rab9b	1
GO:0046889 positive regulation of lipid biosynthetic process	1/138	92/14464	0.01086957	1.13925646	0.13150944	0.58719475	0.78769039	0.77362449	Rab38	1
GO:0051899 membrane depolarization	1/138	92/14464	0.01086957	1.13925646	0.13150944	0.58719475	0.78769039	0.77362449	Gclm	1
GO:1990266 neutrophil migration	1/138	92/14464	0.01086957	1.13925646	0.13150944	0.58719475	0.78769039	0.77362449	Cd99l2	1
GO:0043270 positive regulation of monoatomic ion transport	2/138	206/14464	0.00970874	1.01758829	0.02495366	0.58772794	0.78769039	0.77362449	Kcnip2/Sri	2
GO:0007015 actin filament organization	4/138	429/14464	0.00932401	0.97726428	-0.0469178	0.58862525	0.78769039	0.77362449	Rhoj/Hsp90b1/Scin/Msrb2	4
GO:0003018 vascular process in circulatory system	2/138	207/14464	0.00966184	1.01267241	0.01802339	0.59037463	0.78769039	0.77362449	Manf/Gclm	2
GO:0007411 axon guidance	2/138	207/14464	0.00966184	1.01267241	0.01802339	0.59037463	0.78769039	0.77362449	Sema3e/Crppa	2
GO:0043583 ear development	2/138	207/14464	0.00966184	1.01267241	0.01802339	0.59037463	0.78769039	0.77362449	Grhl3/Slc17a8	2
GO:0097191 extrinsic apoptotic signaling pathway	2/138	207/14464	0.00966184	1.01267241	0.01802339	0.59037463	0.78769039	0.77362449	Pdia3/Gclm	2
GO:0006112 energy reserve metabolic process	1/138	93/14464	0.01075269	1.12700639	0.12059514	0.59115851	0.78769039	0.77362449	Wdr45b	1
GO:0018210 peptidyl-threonine modification	1/138	93/14464	0.01075269	1.12700639	0.12059514	0.59115851	0.78769039	0.77362449	Oxsr1	1
GO:0022404 molting cycle process	1/138	93/14464	0.01075269	1.12700639	0.12059514	0.59115851	0.78769039	0.77362449	Pkp3	1
GO:0022405 hair cycle process	1/138	93/14464	0.01075269	1.12700639	0.12059514	0.59115851	0.78769039	0.77362449	Pkp3	1
GO:0042116 macrophage activation	1/138	93/14464	0.01075269	1.12700639	0.12059514	0.59115851	0.78769039	0.77362449	Hspd1	1
GO:0043280 positive regulation of cysteine-type endopeptidase activity involved in apoptotic process	1/138	93/14464	0.01075269	1.12700639	0.12059514	0.59115851	0.78769039	0.77362449	Hspd1	1
GO:1901874 negative regulation of post-translational protein modification	1/138	93/14464	0.01075269	1.12700639	0.12059514	0.59115851	0.78769039	0.77362449	Bag5	1
GO:1901879 regulation of protein depolymerization	1/138	93/14464	0.01075269	1.12700639	0.12059514	0.59115851	0.78769039	0.77362449	Scin	1
GO:0002697 regulation of immune effector process	3/138	320/14464	0.009375	0.9826087	-0.0308764	0.59229349	0.78816084	0.77408654	Hspd1/Cr1l/Pkp3	3
GO:0003002 regionalization	3/138	320/14464	0.009375	0.9826087	-0.0308764	0.59229349	0.78816084	0.77408654	Dand5/Smad2/Ift172	3
GO:0097485 neuron projection guidance	2/138	208/14464	0.00961538	1.00780379	0.01112614	0.59300879	0.78820471	0.77412962	Sema3e/Crppa	2
GO:0009914 hormone transport	3/138	321/14464	0.00934579	0.97954761	-0.0363691	0.59441899	0.78820471	0.77412962	Bmal1/Smad2/Sri	3
GO:0009308 amine metabolic process	1/138	94/14464	0.0106383	1.11501696	0.10980034	0.59508448	0.78820471	0.77412962	Sat1	1
GO:0032355 response to estradiol	1/138	94/14464	0.0106383	1.11501696	0.10980034	0.59508448	0.78820471	0.77412962	Calr	1
GO:0044409 symbiont entry into host	1/138	94/14464	0.0106383	1.11501696	0.10980034	0.59508448	0.78820471	0.77412962	P4hb	1
GO:0072655 establishment of protein localization to mitochondrion	1/138	94/14464	0.0106383	1.11501696	0.10980034	0.59508448	0.78820471	0.77412962	Hspd1	1
GO:0007160 cell-matrix adhesion	2/138	210/14464	0.00952381	0.99820566	-0.0025707	0.59823955	0.78820471	0.77412962	Mmp14/Sema3e	2
GO:0007163 establishment or maintenance of cell polarity	2/138	210/14464	0.00952381	0.99820566	-0.0025707	0.59823955	0.78820471	0.77412962	Rhoj/Ift80	2
GO:0031348 negative regulation of defense response	2/138	210/14464	0.00952381	0.99820566	-0.0025707	0.59823955	0.78820471	0.77412962	Dtx4/Itch	2
GO:0090276 regulation of peptide hormone secretion	2/138	210/14464	0.00952381	0.99820566	-0.0025707	0.59823955	0.78820471	0.77412962	Bmal1/Sri	2
GO:0007517 muscle organ development	3/138	323/14464	0.00928793	0.9734823	-0.0473046	0.59864871	0.78820471	0.77412962	Bmal1/Fktn/Pkp2	3
GO:0009100 glycoprotein metabolic process	3/138	323/14464	0.00928793	0.9734823	-0.0473046	0.59864871	0.78820471	0.77412962	Fktn/Hexa/Crppa	3
GO:0021700 developmental maturation	3/138	323/14464	0.00928793	0.9734823	-0.0473046	0.59864871	0.78820471	0.77412962	Rab38/Kcnip2/Ift80	3
GO:0002708 positive regulation of lymphocyte mediated immunity	1/138	95/14464	0.01052632	1.10327994	0.09912216	0.59897302	0.78820471	0.77412962	Hspd1	1
GO:0007044 cell-substrate junction assembly	1/138	95/14464	0.01052632	1.10327994	0.09912216	0.59897302	0.78820471	0.77412962	Mmp14	1
GO:0045639 positive regulation of myeloid cell differentiation	1/138	95/14464	0.01052632	1.10327994	0.09912216	0.59897302	0.78820471	0.77412962	Scin	1
GO:1990542 mitochondrial transmembrane transport	1/138	95/14464	0.01052632	1.10327994	0.09912216	0.59897302	0.78820471	0.77412962	Hspd1	1
GO:0060537 muscle tissue development	4/138	435/14464	0.0091954	0.96378477	-0.0752712	0.59965544	0.78858798	0.77450605	Bmal1/Calr/Fktn/Pkp2	4
GO:0006913 nucleocytoplasmic transport	3/138	324/14464	0.00925926	0.97047772	-0.0527477	0.60075289	0.78900184	0.77491252	Bmal1/Calr/Frat2	3
GO:0051169 nuclear transport	3/138	324/14464	0.00925926	0.97047772	-0.0527477	0.60075289	0.78900184	0.77491252	Bmal1/Calr/Frat2	3
GO:0003333 amino acid transmembrane transport	1/138	96/14464	0.01041667	1.09178744	0.08855783	0.60282449	0.7909591	0.77683483	Slc17a8	1
GO:0048232 male gamete generation	4/138	437/14464	0.00915332	0.95937386	-0.0846389	0.60329331	0.7909591	0.77683483	Bmal1/Calr/Tbpl1/Mettl14	4
GO:0099111 microtubule-based transport	2/138	212/14464	0.00943396	0.98878862	-0.0161397	0.60342019	0.7909591	0.77683483	Ift172/Ift80	2
GO:0006417 regulation of translation	3/138	326/14464	0.00920245	0.96452387	-0.0635854	0.60493979	0.7915636	0.77742854	Calr/Dnajc3/Mettl14	3
GO:0070663 regulation of leukocyte proliferation	2/138	213/14464	0.00938967	0.98414642	-0.0228771	0.60599171	0.7915636	0.77742854	Card11/Itch	2
GO:0007029 endoplasmic reticulum organization	1/138	97/14464	0.01030928	1.0805319	0.07810467	0.60663923	0.7915636	0.77742854	Ubl4a	1
GO:0008344 adult locomotory behavior	1/138	97/14464	0.01030928	1.0805319	0.07810467	0.60663923	0.7915636	0.77742854	Hexa	1
GO:0016239 positive regulation of macroautophagy	1/138	97/14464	0.01030928	1.0805319	0.07810467	0.60663923	0.7915636	0.77742854	Snx18	1
GO:0032024 positive regulation of insulin secretion	1/138	97/14464	0.01030928	1.0805319	0.07810467	0.60663923	0.7915636	0.77742854	Sri	1
GO:0098656 monoatomic anion transmembrane transport	1/138	97/14464	0.01030928	1.0805319	0.07810467	0.60663923	0.7915636	0.77742854	Slc17a8	1
GO:0002443 leukocyte mediated immunity	3/138	327/14464	0.00917431	0.96157426	-0.0689801	0.60702248	0.7915636	0.77742854	Ighg2c/Hspd1/Cr1l	3
GO:0006364 rRNA processing	2/138	214/14464	0.00934579	0.97954761	-0.0295835	0.60855071	0.79175574	0.77761724	Mettl16/Tfb1m	2
GO:0043269 regulation of monoatomic ion transport	4/138	440/14464	0.00909091	0.95283267	-0.0986138	0.60871319	0.79175574	0.77761724	Rrad/Kcnip2/Sri/Oxsr1	4
GO:0032956 regulation of actin cytoskeleton organization	3/138	328/14464	0.00914634	0.95864263	-0.0743589	0.60909798	0.79175574	0.77761724	Arhgef16/Grhl3/Scin	3
GO:0002718 regulation of cytokine production involved in immune response	1/138	98/14464	0.01020408	1.06950606	0.0677601	0.6104176	0.79175574	0.77761724	Pkp3	1
GO:0018958 phenol-containing compound metabolic process	1/138	98/14464	0.01020408	1.06950606	0.0677601	0.6104176	0.79175574	0.77761724	Rab38	1
GO:0032204 regulation of telomere maintenance	1/138	98/14464	0.01020408	1.06950606	0.0677601	0.6104176	0.79175574	0.77761724	Hnrnpc	1
GO:0043473 pigmentation	1/138	98/14464	0.01020408	1.06950606	0.0677601	0.6104176	0.79175574	0.77761724	Rab38	1
GO:0090263 positive regulation of canonical Wnt signaling pathway	1/138	98/14464	0.01020408	1.06950606	0.0677601	0.6104176	0.79175574	0.77761724	Bmal1	1
GO:0002791 regulation of peptide secretion	2/138	215/14464	0.00930233	0.97499157	-0.0362593	0.61109719	0.79175574	0.77761724	Bmal1/Sri	2
GO:0090087 regulation of peptide transport	2/138	215/14464	0.00930233	0.97499157	-0.0362593	0.61109719	0.79175574	0.77761724	Bmal1/Sri	2
GO:0042594 response to starvation	2/138	216/14464	0.00925926	0.97047772	-0.0429048	0.61363114	0.7936836	0.77951068	Hspa5/Wdr45b	2
GO:0044403 biological process involved in symbiotic interaction	2/138	216/14464	0.00925926	0.97047772	-0.0429048	0.61363114	0.7936836	0.77951068	Hspd1/P4hb	2
GO:0032760 positive regulation of tumor necrosis factor production	1/138	99/14464	0.01010101	1.05870297	0.05752162	0.61415993	0.7936836	0.77951068	Hspd1	1
GO:2001243 negative regulation of intrinsic apoptotic signaling pathway	1/138	99/14464	0.01010101	1.05870297	0.05752162	0.61415993	0.7936836	0.77951068	Bag5	1
GO:0002833 positive regulation of response to biotic stimulus	3/138	331/14464	0.00906344	0.94995403	-0.0904008	0.61528116	0.7946232	0.7804335	Sash1/Hspd1/Itch	3
GO:0002698 negative regulation of immune effector process	1/138	100/14464	0.01	1.04811594	0.04738682	0.61786658	0.79541444	0.78121061	Cr1l	1
GO:0008645 hexose transmembrane transport	1/138	100/14464	0.01	1.04811594	0.04738682	0.61786658	0.79541444	0.78121061	Slc5a3	1
GO:0014910 regulation of smooth muscle cell migration	1/138	100/14464	0.01	1.04811594	0.04738682	0.61786658	0.79541444	0.78121061	Has2	1
GO:1903557 positive regulation of tumor necrosis factor superfamily cytokine production	1/138	100/14464	0.01	1.04811594	0.04738682	0.61786658	0.79541444	0.78121061	Hspd1	1
GO:1904659 D-glucose transmembrane transport	1/138	100/14464	0.01	1.04811594	0.04738682	0.61786658	0.79541444	0.78121061	Slc5a3	1
GO:0048863 stem cell differentiation	2/138	218/14464	0.00917431	0.96157426	-0.0561061	0.61866152	0.79592956	0.78171654	Sema3e/Ift80	2
GO:0002831 regulation of response to biotic stimulus	4/138	446/14464	0.00896861	0.9400143	-0.1262927	0.6194185	0.79639521	0.78217387	Sash1/Dtx4/Hspd1/Itch	4
GO:0007338 single fertilization	1/138	101/14464	0.00990099	1.03773856	0.03735336	0.62153787	0.79658	0.78235536	Lhfpl2	1
GO:0015837 amine transport	1/138	101/14464	0.00990099	1.03773856	0.03735336	0.62153787	0.79658	0.78235536	Slc17a8	1
GO:0021761 limbic system development	1/138	101/14464	0.00990099	1.03773856	0.03735336	0.62153787	0.79658	0.78235536	Sema3e	1
GO:0050852 T cell receptor signaling pathway	1/138	101/14464	0.00990099	1.03773856	0.03735336	0.62153787	0.79658	0.78235536	Card11	1
GO:0097237 cellular response to toxic substance	1/138	101/14464	0.00990099	1.03773856	0.03735336	0.62153787	0.79658	0.78235536	Akr1a1	1
GO:0006631 fatty acid metabolic process	3/138	335/14464	0.00895522	0.93861129	-0.1115736	0.62342381	0.797186	0.78295053	Degs1/Elovl7/Pdk3	3
GO:0010976 positive regulation of neuron projection development	2/138	220/14464	0.00909091	0.95283267	-0.0691899	0.6236419	0.797186	0.78295053	Hspa5/Zfp804a	2
GO:0000422 autophagy of mitochondrion	1/138	102/14464	0.00980392	1.02756465	0.02741898	0.62517414	0.797186	0.78295053	Wdr45b	1
GO:0002824 positive regulation of adaptive immune response based on somatic recombination of immune receptors b	1/138	102/14464	0.00980392	1.02756465	0.02741898	0.62517414	0.797186	0.78295053	Hspd1	1
GO:0015749 monosaccharide transmembrane transport	1/138	102/14464	0.00980392	1.02756465	0.02741898	0.62517414	0.797186	0.78295053	Slc5a3	1
GO:0042476 odontogenesis	1/138	102/14464	0.00980392	1.02756465	0.02741898	0.62517414	0.797186	0.78295053	Ift80	1
GO:0150115 cell-substrate junction organization	1/138	102/14464	0.00980392	1.02756465	0.02741898	0.62517414	0.797186	0.78295053	Mmp14	1
GO:1901989 positive regulation of cell cycle phase transition	1/138	102/14464	0.00980392	1.02756465	0.02741898	0.62517414	0.797186	0.78295053	Pkp3	1
GO:0043588 skin development	2/138	222/14464	0.00900901	0.9442486	-0.0821586	0.62857235	0.79975511	0.78547377	Grhl3/Pkp3	2
GO:0042752 regulation of circadian rhythm	1/138	103/14464	0.00970874	1.01758829	0.01758151	0.62877572	0.79975511	0.78547377	Bmal1	1
GO:0070585 protein localization to mitochondrion	1/138	103/14464	0.00970874	1.01758829	0.01758151	0.62877572	0.79975511	0.78547377	Hspd1	1
GO:2000278 regulation of DNA biosynthetic process	1/138	103/14464	0.00970874	1.01758829	0.01758151	0.62877572	0.79975511	0.78547377	Hnrnpc	1
GO:0051402 neuron apoptotic process	3/138	339/14464	0.00884956	0.92753623	-0.1325052	0.63144965	0.80226771	0.7879415	Hspd1/Gclm/Sema3e	3
GO:0031330 negative regulation of cellular catabolic process	1/138	104/14464	0.00961538	1.00780379	0.00783883	0.63234295	0.80226771	0.7879415	Sec22b	1
GO:0070646 protein modification by small protein removal	1/138	104/14464	0.00961538	1.00780379	0.00783883	0.63234295	0.80226771	0.7879415	Itch	1
GO:0098754 detoxification	1/138	104/14464	0.00961538	1.00780379	0.00783883	0.63234295	0.80226771	0.7879415	Akr1a1	1
GO:0031667 response to nutrient levels	4/138	455/14464	0.00879121	0.92142061	-0.1671541	0.6351346	0.80472461	0.79035453	Hspa5/Pdia3/Gclm/Wdr45b	4
GO:0015711 organic anion transport	3/138	341/14464	0.00879765	0.92209614	-0.1428825	0.63541855	0.80472461	0.79035453	Slc51a/Slc17a8/Slc33a1	3
GO:2001234 negative regulation of apoptotic signaling pathway	2/138	225/14464	0.00888889	0.93165862	-0.1014008	0.6358746	0.80472461	0.79035453	Gclm/Bag5	2
GO:0051092 positive regulation of NF-kappaB transcription factor activity	1/138	105/14464	0.00952381	0.99820566	-0.0018111	0.63587614	0.80472461	0.79035453	Card11	1
GO:0043549 regulation of kinase activity	4/138	456/14464	0.00877193	0.91939995	-0.1716469	0.63685517	0.80545799	0.79107481	Chordc1/Fzd8/Sash1/Dnajc3	4
GO:0006275 regulation of DNA replication	1/138	106/14464	0.00943396	0.98878862	-0.0113703	0.63937563	0.80763237	0.79321037	Obi1	1
GO:0097061 dendritic spine organization	1/138	106/14464	0.00943396	0.98878862	-0.0113703	0.63937563	0.80763237	0.79321037	Zfp804a	1
GO:0019058 viral life cycle	2/138	227/14464	0.00881057	0.92345017	-0.1140917	0.64068064	0.80826794	0.79383458	Srpk1/P4hb	2
GO:0051056 regulation of small GTPase mediated signal transduction	2/138	227/14464	0.00881057	0.92345017	-0.1140917	0.64068064	0.80826794	0.79383458	Stard13/Ppp2cb	2
GO:0032963 collagen metabolic process	1/138	107/14464	0.00934579	0.97954761	-0.0208406	0.64284172	0.80998057	0.79551663	Mmp14	1
GO:0042542 response to hydrogen peroxide	1/138	107/14464	0.00934579	0.97954761	-0.0208406	0.64284172	0.80998057	0.79551663	Ppp2cb	1
GO:0008585 female gonad development	1/138	108/14464	0.00925926	0.97047772	-0.030224	0.64627474	0.81065074	0.79617483	Mmp14	1
GO:0019079 viral genome replication	1/138	108/14464	0.00925926	0.97047772	-0.030224	0.64627474	0.81065074	0.79617483	Srpk1	1
GO:0042177 negative regulation of protein catabolic process	1/138	108/14464	0.00925926	0.97047772	-0.030224	0.64627474	0.81065074	0.79617483	Bag5	1
GO:0042471 ear morphogenesis	1/138	108/14464	0.00925926	0.97047772	-0.030224	0.64627474	0.81065074	0.79617483	Grhl3	1
GO:0050671 positive regulation of lymphocyte proliferation	1/138	108/14464	0.00925926	0.97047772	-0.030224	0.64627474	0.81065074	0.79617483	Card11	1
GO:2001056 positive regulation of cysteine-type endopeptidase activity	1/138	108/14464	0.00925926	0.97047772	-0.030224	0.64627474	0.81065074	0.79617483	Hspd1	1
GO:0001894 tissue homeostasis	2/138	230/14464	0.00869565	0.91140517	-0.1329276	0.64779679	0.81065074	0.79617483	Pkp3/Ift80	2
GO:0019318 hexose metabolic process	2/138	230/14464	0.00869565	0.91140517	-0.1329276	0.64779679	0.81065074	0.79617483	Pdk3/Arpp19	2
GO:0031331 positive regulation of cellular catabolic process	2/138	230/14464	0.00869565	0.91140517	-0.1329276	0.64779679	0.81065074	0.79617483	Snx18/Itch	2
GO:0060249 anatomical structure homeostasis	2/138	230/14464	0.00869565	0.91140517	-0.1329276	0.64779679	0.81065074	0.79617483	Pkp3/Ift80	2
GO:1902115 regulation of organelle assembly	2/138	230/14464	0.00869565	0.91140517	-0.1329276	0.64779679	0.81065074	0.79617483	Snx18/Sec22b	2
GO:0009410 response to xenobiotic stimulus	3/138	348/14464	0.00862069	0.90354823	-0.1787517	0.6490775	0.81098749	0.79650557	Calr/Gclm/Oxsr1	3
GO:0001578 microtubule bundle formation	1/138	109/14464	0.00917431	0.96157426	-0.0395221	0.649675	0.81098749	0.79650557	Tppp3	1
GO:0039531 regulation of cytoplasmic pattern recognition receptor signaling pathway	1/138	109/14464	0.00917431	0.96157426	-0.0395221	0.649675	0.81098749	0.79650557	Itch	1
GO:0046683 response to organophosphorus	1/138	109/14464	0.00917431	0.96157426	-0.0395221	0.649675	0.81098749	0.79650557	Hsp90b1	1
GO:0042110 T cell activation	4/138	464/14464	0.00862069	0.90354823	-0.2072581	0.65043269	0.81143088	0.79694104	Card11/Fzd8/Hspd1/Itch	4
GO:0002221 pattern recognition receptor signaling pathway	2/138	232/14464	0.00862069	0.90354823	-0.1453542	0.65247917	0.81217415	0.79767104	Hspd1/Itch	2
GO:0002573 myeloid leukocyte differentiation	2/138	232/14464	0.00862069	0.90354823	-0.1453542	0.65247917	0.81217415	0.79767104	Sh3pxd2a/Ift80	2
GO:0002821 positive regulation of adaptive immune response	1/138	110/14464	0.00909091	0.95283267	-0.0487368	0.6530428	0.81217415	0.79767104	Hspd1	1
GO:0034219 carbohydrate transmembrane transport	1/138	110/14464	0.00909091	0.95283267	-0.0487368	0.6530428	0.81217415	0.79767104	Slc5a3	1
GO:0050868 negative regulation of T cell activation	1/138	110/14464	0.00909091	0.95283267	-0.0487368	0.6530428	0.81217415	0.79767104	Itch	1
GO:0002694 regulation of leukocyte activation	4/138	467/14464	0.00856531	0.89774385	-0.2204635	0.6554379	0.81290922	0.79839299	Card11/Mmp14/Hspd1/Itch	4
GO:0014909 smooth muscle cell migration	1/138	111/14464	0.00900901	0.9442486	-0.0578698	0.65637846	0.81290922	0.79839299	Has2	1
GO:0032946 positive regulation of mononuclear cell proliferation	1/138	111/14464	0.00900901	0.9442486	-0.0578698	0.65637846	0.81290922	0.79839299	Card11	1
GO:0051928 positive regulation of calcium ion transport	1/138	111/14464	0.00900901	0.9442486	-0.0578698	0.65637846	0.81290922	0.79839299	Sri	1
GO:2000177 regulation of neural precursor cell proliferation	1/138	111/14464	0.00900901	0.9442486	-0.0578698	0.65637846	0.81290922	0.79839299	Tafa1	1
GO:0009306 protein secretion	3/138	352/14464	0.00852273	0.89328063	-0.198942	0.65671984	0.81290922	0.79839299	Bmal1/Smad2/Sri	3
GO:0002758 innate immune response-activating signaling pathway	2/138	235/14464	0.00851064	0.89201357	-0.1638031	0.65941053	0.81290922	0.79839299	Hspd1/Itch	2
GO:0015850 organic hydroxy compound transport	2/138	235/14464	0.00851064	0.89201357	-0.1638031	0.65941053	0.81290922	0.79839299	Slc5a3/Slc51a	2
GO:0090257 regulation of muscle system process	2/138	235/14464	0.00851064	0.89201357	-0.1638031	0.65941053	0.81290922	0.79839299	Pkp2/Sri	2
GO:0006413 translational initiation	1/138	112/14464	0.00892857	0.93581781	-0.0669227	0.65968229	0.81290922	0.79839299	Dnajc3	1
GO:0007265 Ras protein signal transduction	1/138	112/14464	0.00892857	0.93581781	-0.0669227	0.65968229	0.81290922	0.79839299	Ppp2cb	1
GO:0009791 post-embryonic development	1/138	112/14464	0.00892857	0.93581781	-0.0669227	0.65968229	0.81290922	0.79839299	Smad2	1
GO:0035249 synaptic transmission, glutamatergic	1/138	112/14464	0.00892857	0.93581781	-0.0669227	0.65968229	0.81290922	0.79839299	Slc17a8	1
GO:0051209 release of sequestered calcium ion into cytosol	1/138	112/14464	0.00892857	0.93581781	-0.0669227	0.65968229	0.81290922	0.79839299	Sri	1
GO:0061640 cytoskeleton-dependent cytokinesis	1/138	112/14464	0.00892857	0.93581781	-0.0669227	0.65968229	0.81290922	0.79839299	Snx18	1
GO:0035592 establishment of protein localization to extracellular region	3/138	354/14464	0.00847458	0.88823385	-0.2089559	0.6604965	0.81341537	0.79889009	Bmal1/Smad2/Sri	3
GO:0007249 canonical NF-kappaB signal transduction	2/138	236/14464	0.00847458	0.88823385	-0.1699028	0.66169647	0.81395641	0.79942147	Card11/Ift80	2
GO:0034655 nucleobase-containing compound catabolic process	4/138	471/14464	0.00849257	0.8901197	-0.2379475	0.66203768	0.81395641	0.79942147	Mettl16/Hnrnpc/Pkp3/Mettl14	4
GO:0010639 negative regulation of organelle organization	3/138	355/14464	0.0084507	0.88573178	-0.2139429	0.6623737	0.81395641	0.79942147	Scin/Hnrnpc/Sec22b	3
GO:0038202 TORC1 signaling	1/138	113/14464	0.00884956	0.92753623	-0.0758971	0.66295457	0.81395641	0.79942147	Card11	1
GO:0051283 negative regulation of sequestering of calcium ion	1/138	113/14464	0.00884956	0.92753623	-0.0758971	0.66295457	0.81395641	0.79942147	Sri	1
GO:0007548 sex differentiation	2/138	237/14464	0.00843882	0.88448603	-0.175978	0.6639702	0.81421164	0.79967214	Mmp14/Lhfpl2	2
GO:0048738 cardiac muscle tissue development	2/138	237/14464	0.00843882	0.88448603	-0.175978	0.6639702	0.81421164	0.79967214	Calr/Pkp2	2
GO:0000077 DNA damage checkpoint signaling	1/138	114/14464	0.00877193	0.91939995	-0.0847946	0.66619562	0.81446354	0.79991954	Gnb1l	1
GO:0008643 carbohydrate transport	1/138	114/14464	0.00877193	0.91939995	-0.0847946	0.66619562	0.81446354	0.79991954	Slc5a3	1
GO:0045621 positive regulation of lymphocyte differentiation	1/138	114/14464	0.00877193	0.91939995	-0.0847946	0.66619562	0.81446354	0.79991954	Mmp14	1
GO:0072089 stem cell proliferation	1/138	114/14464	0.00877193	0.91939995	-0.0847946	0.66619562	0.81446354	0.79991954	Ift80	1
GO:1990830 cellular response to leukemia inhibitory factor	1/138	114/14464	0.00877193	0.91939995	-0.0847946	0.66619562	0.81446354	0.79991954	Gclm	1
GO:0002705 positive regulation of leukocyte mediated immunity	1/138	115/14464	0.00869565	0.91140517	-0.0936166	0.66940573	0.81492655	0.80037429	Hspd1	1
GO:0006639 acylglycerol metabolic process	1/138	115/14464	0.00869565	0.91140517	-0.0936166	0.66940573	0.81492655	0.80037429	Dgkk	1
GO:0030282 bone mineralization	1/138	115/14464	0.00869565	0.91140517	-0.0936166	0.66940573	0.81492655	0.80037429	Ift80	1
GO:0043200 response to amino acid	1/138	115/14464	0.00869565	0.91140517	-0.0936166	0.66940573	0.81492655	0.80037429	Gclm	1
GO:0051053 negative regulation of DNA metabolic process	1/138	115/14464	0.00869565	0.91140517	-0.0936166	0.66940573	0.81492655	0.80037429	Hnrnpc	1
GO:0051282 regulation of sequestering of calcium ion	1/138	115/14464	0.00869565	0.91140517	-0.0936166	0.66940573	0.81492655	0.80037429	Sri	1
GO:0001503 ossification	3/138	359/14464	0.00835655	0.8758629	-0.2337591	0.66980818	0.81492655	0.80037429	Dhrs3/Mmp14/Ift80	3
GO:0030098 lymphocyte differentiation	3/138	359/14464	0.00835655	0.8758629	-0.2337591	0.66980818	0.81492655	0.80037429	Card11/Mmp14/Fzd8	3
GO:0051924 regulation of calcium ion transport	2/138	240/14464	0.00833333	0.87342995	-0.1940582	0.67071833	0.81554171	0.80097847	Rrad/Sri	2
GO:0006638 neutral lipid metabolic process	1/138	116/14464	0.00862069	0.90354823	-0.1023646	0.67258518	0.81573671	0.80116998	Dgkk	1

GO:0051208	sequestering of calcium ion	1/138	116/14464	0.00862069	0.90354823	-0.1023646	0.67258518	0.81573671	0.80116998	Sri	1
GO:1990823	response to leukemia inhibitory factor	1/138	116/14464	0.00862069	0.90354823	-0.1023646	0.67258518	0.81573671	0.80116998	Gclm	1
GO:0071695	anatomical structure maturation	2/138	242/14464	0.00826446	0.86621152	-0.2059929	0.67515645	0.81573671	0.80116998	Kcnip2/Ift80	2
GO:0090596	sensory organ morphogenesis	2/138	242/14464	0.00826446	0.86621152	-0.2059929	0.67515645	0.81573671	0.80116998	Grhl3/Ift172	2
GO:1903706	regulation of hemopoiesis	3/138	362/14464	0.00828729	0.86860437	-0.2484855	0.67530602	0.81573671	0.80116998	Card11/Mmp14/Scin	3
GO:0006820	monoatomic anion transport	1/138	117/14464	0.00854701	0.89582559	-0.1110401	0.67573428	0.81573671	0.80116998	Slc17a8	1
GO:0008584	male gonad development	1/138	117/14464	0.00854701	0.89582559	-0.1110401	0.67573428	0.81573671	0.80116998	Mmp14	1
GO:0015695	organic cation transport	1/138	117/14464	0.00854701	0.89582559	-0.1110401	0.67573428	0.81573671	0.80116998	Slc17a8	1
GO:0015698	inorganic anion transport	1/138	117/14464	0.00854701	0.89582559	-0.1110401	0.67573428	0.81573671	0.80116998	Slc17a8	1
GO:0017148	negative regulation of translation	1/138	117/14464	0.00854701	0.89582559	-0.1110401	0.67573428	0.81573671	0.80116998	Calr	1
GO:0106027	neuron projection organization	1/138	117/14464	0.00854701	0.89582559	-0.1110401	0.67573428	0.81573671	0.80116998	Zfp804a	1
GO:0002367	cytokine production involved in immune response	1/138	118/14464	0.00847458	0.88823385	-0.1196443	0.6788533	0.81754376	0.80294477	Pkp3	1
GO:0009142	nucleoside triphosphate biosynthetic process	1/138	118/14464	0.00847458	0.88823385	-0.1196443	0.6788533	0.81754376	0.80294477	Tbpl1	1
GO:0032479	regulation of type I interferon production	1/138	118/14464	0.00847458	0.88823385	-0.1196443	0.6788533	0.81754376	0.80294477	Hspd1	1
GO:0051588	regulation of neurotransmitter transport	1/138	118/14464	0.00847458	0.88823385	-0.1196443	0.6788533	0.81754376	0.80294477	Slc17a8	1
GO:0030278	regulation of ossification	1/138	119/14464	0.00840336	0.8807697	-0.1281788	0.68194254	0.82028411	0.80563618	Dhrs3	1
GO:0048593	camera-type eye morphogenesis	1/138	119/14464	0.00840336	0.8807697	-0.1281788	0.68194254	0.82028411	0.80563618	Ift172	1
GO:0045860	positive regulation of protein kinase activity	2/138	246/14464	0.00813008	0.85212678	-0.2295853	0.68388808	0.82200274	0.80732412	Fzd8/Sash1	2
GO:0031507	heterochromatin formation	1/138	120/14464	0.00833333	0.87342995	-0.1366447	0.68500228	0.82200274	0.80732412	Cbx1	1
GO:0051224	negative regulation of protein transport	1/138	120/14464	0.00833333	0.87342995	-0.1366447	0.68500228	0.82200274	0.80732412	Ndfip2	1
GO:0051783	regulation of nuclear division	1/138	120/14464	0.00833333	0.87342995	-0.1366447	0.68500228	0.82200274	0.80732412	Calr	1
GO:0016072	rRNA metabolic process	2/138	247/14464	0.00809717	0.84867688	-0.2354269	0.68604103	0.82221346	0.80753108	Mettl16/Tfb1m	2
GO:0032984	protein-containing complex disassembly	2/138	247/14464	0.00809717	0.84867688	-0.2354269	0.68604103	0.82221346	0.80753108	Scin/Snx14	2
GO:0031570	DNA integrity checkpoint signaling	1/138	121/14464	0.00826446	0.86621152	-0.1450433	0.6880328	0.82221346	0.80753108	Gnb1l	1
GO:0032434	regulation of proteasomal ubiquitin-dependent protein catabolic process	1/138	121/14464	0.00826446	0.86621152	-0.1450433	0.6880328	0.82221346	0.80753108	Bag5	1
GO:0051261	protein depolymerization	1/138	121/14464	0.00826446	0.86621152	-0.1450433	0.6880328	0.82221346	0.80753108	Scin	1
GO:0090277	positive regulation of peptide hormone secretion	1/138	121/14464	0.00826446	0.86621152	-0.1450433	0.6880328	0.82221346	0.80753108	Sri	1
GO:1901890	positive regulation of cell junction assembly	1/138	121/14464	0.00826446	0.86621152	-0.1450433	0.6880328	0.82221346	0.80753108	Lrrn1	1
GO:0070372	regulation of ERK1 and ERK2 cascade	2/138	249/14464	0.00803213	0.84186019	-0.2470437	0.69031118	0.82433448	0.80961422	S2bpcox16/Spred3	2
GO:0032606	type I interferon production	1/138	122/14464	0.00819672	0.85911143	-0.1533759	0.69103436	0.82433448	0.80961422	Hspd1	1
GO:0045834	positive regulation of lipid metabolic process	1/138	122/14464	0.00819672	0.85911143	-0.1533759	0.69103436	0.82433448	0.80961422	Rab38	1
GO:0070665	positive regulation of leukocyte proliferation	1/138	123/14464	0.00813008	0.85212678	-0.1616436	0.69400726	0.82690226	0.81213615	Card11	1
GO:1903038	negative regulation of leukocyte cell-cell adhesion	1/138	123/14464	0.00813008	0.85212678	-0.1616436	0.69400726	0.82690226	0.81213615	Itch	1
GO:0031503	protein-containing complex localization	2/138	251/14464	0.00796813	0.83515215	-0.2585732	0.69453385	0.82698023	0.81221273	Ift172/Ift80	2
GO:0030900	forebrain development	3/138	373/14464	0.0080429	0.84298869	-0.3015232	0.69489311	0.82698023	0.81221273	Fktn/Sema3e/Mettl14	3
GO:0045088	regulation of innate immune response	3/138	374/14464	0.00802139	0.84073471	-0.3062723	0.6966293	0.82747628	0.81269992	Dtx4/Hspd1/Itch	3
GO:0021987	cerebral cortex development	1/138	124/14464	0.00806452	0.84525479	-0.1698477	0.69695175	0.82747628	0.81269992	Fktn	1
GO:0051100	negative regulation of binding	1/138	124/14464	0.00806452	0.84525479	-0.1698477	0.69695175	0.82747628	0.81269992	Sri	1
GO:0097530	granulocyte migration	1/138	124/14464	0.00806452	0.84525479	-0.1698477	0.69695175	0.82747628	0.81269992	Cd99l2	1
GO:0000086	G2/M transition of mitotic cell cycle	1/138	125/14464	0.008	0.83849275	-0.1779893	0.69986812	0.82923638	0.81442858	Arpp19	1
GO:0002793	positive regulation of peptide secretion	1/138	125/14464	0.008	0.83849275	-0.1779893	0.69986812	0.82923638	0.81442858	Sri	1
GO:0010950	positive regulation of endopeptidase activity	1/138	125/14464	0.008	0.83849275	-0.1779893	0.69986812	0.82923638	0.81442858	Hspd1	1
GO:0050803	regulation of synapse structure or activity	3/138	376/14464	0.00797872	0.83626272	-0.3157353	0.70007952	0.82923638	0.81442858	Lrrn1/Slc17a8/Zfp804a	3
GO:0014812	muscle cell migration	1/138	126/14464	0.00793651	0.83183805	-0.1860694	0.70275662	0.83143036	0.81658339	Has2	1
GO:0015718	monocarboxylic acid transport	1/138	126/14464	0.00793651	0.83183805	-0.1860694	0.70275662	0.83143036	0.81658339	Slc51a	1
GO:0031400	negative regulation of protein modification process	3/138	378/14464	0.00793651	0.83183805	-0.3251517	0.70350022	0.83182197	0.816968	Chordc1/Dnajc3/Bag5	3
GO:0050878	regulation of body fluid levels	2/138	256/14464	0.0078125	0.81884058	-0.2870256	0.70488452	0.83188591	0.81703081	Has2/Pdia3	2
GO:0007041	lysosomal transport	1/138	127/14464	0.00787402	0.82528814	-0.1940892	0.70561752	0.83188591	0.81703081	Aktip	1
GO:0010565	regulation of cellular ketone metabolic process	1/138	127/14464	0.00787402	0.82528814	-0.1940892	0.70561752	0.83188591	0.81703081	Pdk3	1
GO:0030177	positive regulation of Wnt signaling pathway	1/138	127/14464	0.00787402	0.82528814	-0.1940892	0.70561752	0.83188591	0.81703081	Bmal1	1
GO:0046822	regulation of nucleocytoplasmic transport	1/138	127/14464	0.00787402	0.82528814	-0.1940892	0.70561752	0.83188591	0.81703081	Frat2	1
GO:0002757	immune response-activating signaling pathway	3/138	380/14464	0.00789474	0.82745995	-0.3345222	0.70689147	0.83239908	0.81753481	Card11/Hspd1/Itch	3
GO:0010927	cellular component assembly involved in morphogenesis	1/138	128/14464	0.0078125	0.81884058	-0.2020497	0.70845108	0.83239908	0.81753481	Tbpl1	1
GO:0014074	response to purine-containing compound	1/138	128/14464	0.0078125	0.81884058	-0.2020497	0.70845108	0.83239908	0.81753481	Hsp90b1	1
GO:0030856	regulation of epithelial cell differentiation	1/138	128/14464	0.0078125	0.81884058	-0.2020497	0.70845108	0.83239908	0.81753481	Spred3	1
GO:0032989	cellular anatomical entity morphogenesis	1/138	128/14464	0.0078125	0.81884058	-0.2020497	0.70845108	0.83239908	0.81753481	Tbpl1	1
GO:0002218	activation of innate immune response	2/138	258/14464	0.00775194	0.81249298	-0.2982619	0.70894307	0.83239908	0.81753481	Hspd1/Itch	2
GO:0033674	positive regulation of kinase activity	2/138	258/14464	0.00775194	0.81249298	-0.2982619	0.70894307	0.83239908	0.81753481	Fzd8/Sash1	2
GO:0006090	pyruvate metabolic process	1/138	129/14464	0.00775194	0.81249298	-0.2099519	0.71125756	0.83366003	0.81877324	Pdk3	1
GO:0015748	organophosphate ester transport	1/138	129/14464	0.00775194	0.81249298	-0.2099519	0.71125756	0.83366003	0.81877324	Slc33a1	1
GO:0016482	cytosolic transport	1/138	129/14464	0.00775194	0.81249298	-0.2099519	0.71125756	0.83366003	0.81877324	Rab9b	1
GO:0006906	vesicle fusion	1/138	130/14464	0.00769231	0.80624303	-0.2177968	0.71403722	0.83643175	0.82149547	Sec22b	1
GO:0046883	regulation of hormone secretion	2/138	261/14464	0.00766284	0.80315398	-0.3149656	0.71494417	0.83700781	0.82206124	Bmal1/Sri	2
GO:0007098	centrosome cycle	1/138	131/14464	0.00763359	0.80008851	-0.2255855	0.71679032	0.83722438	0.82227395	Chordc1	1
GO:0009566	fertilization	1/138	131/14464	0.00763359	0.80008851	-0.2255855	0.71679032	0.83722438	0.82227395	Lhfpl2	1
GO:0030833	regulation of actin filament polymerization	1/138	131/14464	0.00763359	0.80008851	-0.2255855	0.71679032	0.83722438	0.82227395	Scin	1
GO:0043244	regulation of protein-containing complex disassembly	1/138	131/14464	0.00763359	0.80008851	-0.2255855	0.71679032	0.83722438	0.82227395	Scin	1
GO:0030217	T cell differentiation	2/138	263/14464	0.00760456	0.79704634	-0.326003	0.7188875	0.83749796	0.82254264	Card11/Fzd8	2
GO:0050708	regulation of protein secretion	2/138	263/14464	0.00760456	0.79704634	-0.326003	0.7188875	0.83749796	0.82254264	Bmal1/Sri	2
GO:0001101	response to acid chemical	1/138	132/14464	0.00757576	0.79402723	-0.2333187	0.7195171	0.83749796	0.82254264	Gclm	1
GO:0010952	positive regulation of peptidase activity	1/138	132/14464	0.00757576	0.79402723	-0.2333187	0.7195171	0.83749796	0.82254264	Hspd1	1
GO:0031109	microtubule polymerization or depolymerization	1/138	132/14464	0.00757576	0.79402723	-0.2333187	0.7195171	0.83749796	0.82254264	Tppp3	1
GO:0090174	organelle membrane fusion	1/138	132/14464	0.00757576	0.79402723	-0.2333187	0.7195171	0.83749796	0.82254264	Sec22b	1
GO:0032675	regulation of interleukin-6 production	1/138	133/14464	0.0075188	0.7880571	-0.2409976	0.72221781	0.83918796	0.82420246	Hspd1	1
GO:0055007	cardiac muscle cell differentiation	1/138	133/14464	0.0075188	0.7880571	-0.2409976	0.72221781	0.83918796	0.82420246	Calr	1
GO:1905039	carboxylic acid transmembrane transport	1/138	133/14464	0.0075188	0.7880571	-0.2409976	0.72221781	0.83918796	0.82420246	Slc17a8	1
GO:0001959	regulation of cytokine-mediated signaling pathway	1/138	134/14464	0.00746269	0.78217608	-0.2486229	0.72489271	0.84084218	0.82582714	Ppp2cb	1
GO:0003158	endothelium development	1/138	134/14464	0.00746269	0.78217608	-0.2486229	0.72489271	0.84084218	0.82582714	Stard13	1
GO:0045598	regulation of fat cell differentiation	1/138	134/14464	0.00746269	0.78217608	-0.2486229	0.72489271	0.84084218	0.82582714	Bmal1	1
GO:0051701	biological process involved in interaction with host	1/138	135/14464	0.00740741	0.77638218	-0.2561956	0.72754203	0.84294525	0.82789266	P4hb	1
GO:1904950	negative regulation of establishment of protein localization	1/138	135/14464	0.00740741	0.77638218	-0.2561956	0.72754203	0.84294525	0.82789266	Ndfip2	1
GO:0022604	regulation of cell morphogenesis	2/138	268/14464	0.00746269	0.78217608	-0.3532614	0.72854706	0.84362486	0.82856013	Rhoj/Sema3e	2
GO:1903825	organic acid transmembrane transport	1/138	136/14464	0.00735294	0.77067349	-0.2637164	0.73016603	0.84501418	0.82992465	Slc17a8	1
GO:0003012	muscle system process	3/138	395/14464	0.00759494	0.79603742	-0.4033887	0.73139313	0.84559479	0.83049488	Kcnip2/Pkp2/Sri	3
GO:0035725	sodium ion transmembrane transport	1/138	137/14464	0.00729927	0.76504813	-0.2711863	0.73276493	0.84559479	0.83049488	Slc17a8	1
GO:0046488	phosphatidylinositol metabolic process	1/138	137/14464	0.00729927	0.76504813	-0.2711863	0.73276493	0.84559479	0.83049488	Pigg	1
GO:0072073	kidney epithelium development	1/138	137/14464	0.00729927	0.76504813	-0.2711863	0.73276493	0.84559479	0.83049488	Smad2	1
GO:1902600	proton transmembrane transport	1/138	137/14464	0.00729927	0.76504813	-0.2711863	0.73276493	0.84559479	0.83049488	Atp6v0b	1
GO:0002764	immune response-regulating signaling pathway	3/138	397/14464	0.00755668	0.79202716	-0.4123893	0.73453669	0.8460507	0.83094265	Card11/Hspd1/Itch	3
GO:0050768	negative regulation of neurogenesis	1/138	138/14464	0.00724638	0.75950431	-0.2786061	0.73533898	0.8460507	0.83094265	Dnajb11	1
GO:0050851	antigen receptor-mediated signaling pathway	1/138	138/14464	0.00724638	0.75950431	-0.2786061	0.73533898	0.8460507	0.83094265	Card11	1
GO:0051099	positive regulation of binding	1/138	138/14464	0.00724638	0.75950431	-0.2786061	0.73533898	0.8460507	0.83094265	Aktip	1
GO:0009101	glycoprotein biosynthetic process	2/138	272/14464	0.00735294	0.77067349	-0.3747341	0.73607273	0.8460507	0.83094265	Fktn/Crppa	2
GO:0061448	connective tissue development	2/138	272/14464	0.00735294	0.77067349	-0.3747341	0.73607273	0.8460507	0.83094265	Scin/Ift80	2
GO:0043086	negative regulation of catalytic activity	3/138	398/14464	0.00753769	0.79003714	-0.4168742	0.73609768	0.8460507	0.83094265	Chordc1/Dnajc3/Bag5	3
GO:0006997	nucleus organization	1/138	139/14464	0.00719424	0.75404025	-0.2859765	0.73788842	0.84622278	0.83111166	Tbpl1	1
GO:0032635	interleukin-6 production	1/138	139/14464	0.00719424	0.75404025	-0.2859765	0.73788842	0.84622278	0.83111166	Hspd1	1
GO:0120161	regulation of cold-induced thermogenesis	1/138	139/14464	0.00719424	0.75404025	-0.2859765	0.73788842	0.84622278	0.83111166	Bmal1	1
GO:0070371	ERK1 and ERK2 cascade	2/138	273/14464	0.00732601	0.76785051	-0.3800571	0.73792641	0.84622278	0.83111166	S2bpcox16/Spred3	2
GO:0051250	negative regulation of lymphocyte activation	1/138	140/14464	0.00714286	0.74865424	-0.2932984	0.74041347	0.84859213	0.8334387	Itch	1
GO:0031023	microtubule organizing center organization	1/138	141/14464	0.0070922	0.74334464	-0.3005724	0.74291438	0.84952659	0.83435647	Chordc1	1
GO:0031644	regulation of nervous system process	1/138	141/14464	0.0070922	0.74334464	-0.3005724	0.74291438	0.84952659	0.83435647	Smr2l	1
GO:0044839	cell cycle G2/M phase transition	1/138	141/14464	0.0070922	0.74334464	-0.3005724	0.74291438	0.84952659	0.83435647	Arpp19	1
GO:0055088	lipid homeostasis	1/138	141/14464	0.0070922	0.74334464	-0.3005724	0.74291438	0.84952659	0.83435647	Pkp2	1
GO:0048592	eye morphogenesis	1/138	142/14464	0.00704225	0.73810982	-0.3077994	0.74539136	0.85130141	0.8360996	Ift172	1
GO:0140013	meiotic nuclear division	1/138	142/14464	0.00704225	0.73810982	-0.3077994	0.74539136	0.85130141	0.8360996	Calr	1
GO:0043491	phosphatidylinositol 3-kinase/protein kinase B signal transduction	2/138	278/14464	0.00719424	0.75404025	-0.4064085	0.74703039	0.85130141	0.8360996	Sema3e/Ift80	2
GO:0000723	telomere maintenance	1/138	143/14464	0.00699301	0.73294821	-0.3149801	0.74784465	0.85130141	0.8360996	Hnrnpc	1
GO:0001890	placenta development	1/138	143/14464	0.00699301	0.73294821	-0.3149801	0.74784465	0.85130141	0.8360996	Tppp3	1
GO:0032006	regulation of TOR signaling	1/138	143/14464	0.00699301	0.73294821	-0.3149801	0.74784465	0.85130141	0.8360996	Bmal1	1
GO:0043123	positive regulation of canonical NF-kappaB signal transduction	1/138	143/14464	0.00699301	0.73294821	-0.3149801	0.74784465	0.85130141	0.8360996	Card11	1
GO:0106106	cold-induced thermogenesis	1/138	143/14464	0.00699301	0.73294821	-0.3149801	0.74784465	0.85130141	0.8360996	Bmal1	1
GO:0030178	negative regulation of Wnt signaling pathway	1/138	144/14464	0.00694444	0.72785829	-0.3221151	0.75027446	0.85262307	0.83739765	Ift80	1
GO:0043281	regulation of cysteine-type endopeptidase activity involved in apoptotic process	1/138	144/14464	0.00694444	0.72785829	-0.3221151	0.75027446	0.85262307	0.83739765	Hspd1	1
GO:0051147	regulation of muscle cell differentiation	1/138	144/14464	0.00694444	0.72785829	-0.3221151	0.75027446	0.85262307	0.83739765	Mmp14	1
GO:0001933	negative regulation of protein phosphorylation	2/138	281/14464	0.00711744	0.74598999	-0.4220134	0.75236268	0.85343362	0.83819373	Chordc1/Dnajc3	2
GO:0046942	carboxylic acid transport	2/138	281/14464	0.00711744	0.74598999	-0.4220134	0.75236268	0.85343362	0.83819373	Slc51a/Slc17a8	2
GO:0007596	blood coagulation	1/138	145/14464	0.00689655	0.72283858	-0.3292052	0.75268104	0.85343362	0.83819373	Pdia3	1
GO:0060759	regulation of response to cytokine stimulus	1/138	145/14464	0.00689655	0.72283858	-0.3292052	0.75268104	0.85343362	0.83819373	Ppp2cb	1
GO:0030111	regulation of Wnt signaling pathway	2/138	282/14464	0.0070922	0.74334464	-0.4271815	0.75411867	0.85458305	0.83932264	Bmal1/Ift80	2
GO:0007286	spermatid development	1/138	146/14464	0.00684932	0.71788763	-0.336251	0.75506459	0.85469411	0.83943171	Tbpl1	1
GO:2001235	positive regulation of apoptotic signaling pathway	1/138	146/14464	0.00684932	0.71788763	-0.336251	0.75506459	0.85469411	0.83943171	Pdia3	1
GO:0015849	organic acid transport	2/138	283/14464	0.00706714	0.74071798	-0.4323331	0.75586402	0.85511889	0.83984891	Slc51a/Slc17a8	2
GO:0051961	negative regulation of nervous system development	1/138	147/14464	0.00680272	0.71300404	-0.3432533	0.75742533	0.85640463	0.84111169	Dnajb11	1
GO:0043254	regulation of protein-containing complex assembly	3/138	413/14464	0.00726392	0.7613433	-0.482949	0.75865906	0.85731876	0.8420095	Hspa5/Scin/Tppp3	3
GO:0002244	hematopoietic progenitor cell differentiation	1/138	148/14464	0.00675676	0.70818645	-0.3502126	0.75976348	0.85760536	0.84229098	Mettl14	1
GO:0007599	hemostasis	1/138	148/14464	0.00675676	0.70818645	-0.3502126	0.75976348	0.85760536	0.84229098	Pdia3	1
GO:1902105	regulation of leukocyte differentiation	2/138	286/14464	0.00699301	0.73294821	-0.4476899	0.76103656	0.85856167	0.84323021	Card11/Mmp14	2
GO:0032200	telomere organization	1/138	149/14464	0.00671141	0.70343352	-0.3571295	0.76207925	0.85902776	0.84368798	Hnrnpc	1
GO:0008064	regulation of actin polymerization or depolymerization	1/138	150/14464	0.00666667	0.69874396	-0.3640048	0.76437286	0.85902776	0.84368798	Scin	1
GO:0010977	negative regulation of neuron projection development	1/138	150/14464	0.00666667	0.69874396	-0.3640048	0.76437286	0.85902776	0.84368798	Bag5	1
GO:0031214	biomineral tissue development	1/138	150/14464	0.00666667	0.69874396	-0.3640048	0.76437286	0.85902776	0.84368798	Ift80	1
GO:0031623	receptor internalization	1/138	150/14464	0.00666667	0.69874396	-0.3640048	0.76437286	0.85902776	0.84368798	Itch	1
GO:0050817	coagulation	1/138	150/14464	0.00666667	0.69874396	-0.3640048	0.76437286	0.85902776	0.84368798	Pdia3	1
GO:0045787	positive regulation of cell cycle	2/138	288/14464	0.00694444	0.72785829	-0.4578474	0.76443244	0.85902776	0.84368798	Calr/Pkp3	2
GO:0030832	regulation of actin filament length	1/138	151/14464	0.00662252	0.69411652	-0.370839	0.76664452	0.86055421	0.84518717	Scin	1
GO:0045580	regulation of T cell differentiation	1/138	151/14464	0.00662252	0.69411652	-0.370839	0.76664452	0.86055421	0.84518717	Card11	1
GO:0048545	response to steroid hormone	2/138	290/14464	0.00689655	0.72283858	-0.4679418	0.76778673	0.86087766	0.84550485	Bmal1/Calr	2
GO:0070588	calcium ion transmembrane transport	2/138	290/14464	0.00689655	0.72283858	-0.4679418	0.76778673	0.86087766	0.84550485	Rrad/Sri	2
GO:0050714	positive regulation of protein secretion	1/138	152/14464	0.00657895	0.68954996	-0.3776327	0.76889444	0.86164046	0.84625403	Sri	1
GO:0048511	rhythmic process	2/138	291/14464	0.00687285	0.7203546	-0.4729656	0.76944838	0.86178218	0.84639322	Bmal1/Has2	2
GO:0045814	negative regulation of gene expression, epigenetic	1/138	153/14464	0.00653595	0.6850431	-0.3843865	0.77112282	0.862699	0.84729367	Cbx1	1
GO:0046887	positive regulation of hormone secretion	1/138	153/14464	0.00653595	0.6850431	-0.3843865	0.77112282	0.862699	0.84729367	Sri	1
GO:0042692	muscle cell differentiation	3/138	423/14464	0.0070922	0.74334464	-0.5258087	0.77282637	0.8637302	0.84830645	Calr/Mmp14/Fktn	3
GO:0048515	spermatid differentiation	1/138	154/14464	0.00649351	0.68059477	-0.3911009	0.77332987	0.8637302	0.84830645	Tbpl1	1
GO:0062013	positive regulation of small molecule metabolic process	1/138	154/14464	0.00649351	0.68059477	-0.3911009	0.77332987	0.8637302	0.84830645	Arpp19	1
GO:0045862	positive regulation of proteolysis	2/138	296/14464	0.00675676	0.70818645	-0.4978555	0.77760346	0.86666332	0.85118719	Mmp14/Hspd1	2
GO:0001906	cell killing	1/138	156/14464	0.00641026	0.67186919	-0.4044139	0.77768077	0.86666332	0.85118719	Cr1l	1
GO:0030010	establishment of cell polarity	1/138	156/14464	0.00641026	0.67186919	-0.4044139	0.77768077	0.86666332	0.85118719	Rhoj	1
GO:0030099	myeloid cell differentiation	3/138	428/14464	0.00700935	0.73466071	-0.5469001	0.77965384	0.86666332	0.85118719	Scin/Sh3pxd2a/Ift80	3
GO:0002700	regulation of production of molecular mediator of immune response	1/138	157/14464	0.00636943	0.66758977	-0.4110136	0.77982503	0.86666332	0.85118719	Pkp3	1
GO:0002753	cytoplasmic pattern recognition receptor signaling pathway	1/138	157/14464	0.00636943	0.66758977	-0.4110136	0.77982503	0.86666332	0.85118719	Itch	1
GO:0062207	regulation of pattern recognition receptor signaling pathway	1/138	157/14464	0.00636943	0.66758977	-0.4110136	0.77982503	0.86666332	0.85118719	Itch	1
GO:1990845	adaptive thermogenesis	1/138	157/14464	0.00636943	0.66758977	-0.4110136	0.77982503	0.86666332	0.85118719	Bmal1	1
GO:2000058	regulation of ubiquitin-dependent protein catabolic process	1/138	157/14464	0.00636943	0.66758977	-0.4110136	0.77982503	0.86666332	0.85118719	Bag5	1
GO:0019216	regulation of lipid metabolic process	2/138	298/14464	0.00671141	0.70343352	-0.5077066	0.78079481	0.86726299	0.85177615	Rab38/Pdk3	2
GO:0006865	amino acid transport	1/138	158/14464	0.00632911	0.66336452	-0.4175761	0.78194876	0.86804814	0.85254728	Slc17a8	1
GO:0061025	membrane fusion	1/138	159/14464	0.00628931	0.65919242	-0.4241018	0.78405215	0.86804814	0.85254728	Sec22b	1
GO:0071897	DNA biosynthetic process	1/138	159/14464	0.00628931	0.65919242	-0.4241018	0.78405215	0.86804814	0.85254728	Hnrnpc	1
GO:0097553	calcium ion transmembrane import into cytosol	1/138	159/14464	0.00628931	0.65919242	-0.4241018	0.78405215	0.86804814	0.85254728	Sri	1
GO:0016032	viral process	2/138	301/14464	0.00664452	0.69642255	-0.5223738	0.78550719	0.86804814	0.85254728	Srpk1/P4hb	2
GO:0042326	negative regulation of phosphorylation	2/138	301/14464	0.00664452	0.69642255	-0.5223738	0.78550719	0.86804814	0.85254728	Chordc1/Dnajc3	2
GO:0071214	cellular response to abiotic stimulus	2/138	301/14464	0.00664452	0.69642255	-0.5223738	0.78550719	0.86804814	0.85254728	Calr/Oxsr1	2
GO:0104004	cellular response to environmental stimulus	2/138	301/14464	0.00664452	0.69642255	-0.5223738	0.78550719	0.86804814	0.85254728	Calr/Oxsr1	2
GO:0002683	negative regulation of immune system process	3/138	433/14464	0.00692841	0.72617733	-0.5677737	0.78631338	0.86804814	0.85254728	Dtx4/Cr1l/Itch	3
GO:0097193	intrinsic apoptotic signaling pathway	2/138	302/14464	0.00662252	0.69411652	-0.5272341	0.78705826	0.86804814	0.85254728	Bag5/P4hb	2

GO:0002695	negative regulation of leukocyte activation	1/138	161/14464	0.00621118	0.65100369	-0.4370453	0.78819868	0.86804814	0.85254728	Itch	1
GO:0008217	regulation of blood pressure	1/138	161/14464	0.00621118	0.65100369	-0.4370453	0.78819868	0.86804814	0.85254728	Manf	1
GO:0032680	regulation of tumor necrosis factor production	1/138	161/14464	0.00621118	0.65100369	-0.4370453	0.78819868	0.86804814	0.85254728	Hspd1	1
GO:0033157	regulation of intracellular protein transport	1/138	161/14464	0.00621118	0.65100369	-0.4370453	0.78819868	0.86804814	0.85254728	Frat2	1
GO:0051897	positive regulation of phosphatidylinositol 3-kinase/protein kinase B signal transduction	1/138	161/14464	0.00621118	0.65100369	-0.4370453	0.78819868	0.86804814	0.85254728	Sema3e	1
GO:0001822	kidney development	2/138	303/14464	0.00660066	0.6918257	-0.5320801	0.78859953	0.86804814	0.85254728	Has2/Smad2	2
GO:0007034	vacuolar transport	1/138	162/14464	0.00617284	0.64698515	-0.443464	0.79024221	0.86804814	0.85254728	Aktip	1
GO:0042391	regulation of membrane potential	3/138	437/14464	0.00686499	0.71953039	-0.5843195	0.79152162	0.86804814	0.85254728	Kcnip2/Gclm/Pkp2	3
GO:0062197	cellular response to chemical stress	2/138	305/14464	0.00655738	0.68728914	-0.54173	0.79165288	0.86804814	0.85254728	Bmal1/Oxsr1	2
GO:0002285	lymphocyte activation involved in immune response	1/138	163/14464	0.00613497	0.64301592	-0.4498479	0.79226616	0.86804814	0.85254728	Hspd1	1
GO:0006606	protein import into nucleus	1/138	163/14464	0.00613497	0.64301592	-0.4498479	0.79226616	0.86804814	0.85254728	Bmal1	1
GO:0010508	positive regulation of autophagy	1/138	163/14464	0.00613497	0.64301592	-0.4498479	0.79226616	0.86804814	0.85254728	Snx18	1
GO:0051017	actin filament bundle assembly	1/138	163/14464	0.00613497	0.64301592	-0.4498479	0.79226616	0.86804814	0.85254728	Hsp90b1	1
GO:1903555	regulation of tumor necrosis factor superfamily cytokine production	1/138	163/14464	0.00613497	0.64301592	-0.4498479	0.79226616	0.86804814	0.85254728	Hspd1	1
GO:2000116	regulation of cysteine-type endopeptidase activity	1/138	163/14464	0.00613497	0.64301592	-0.4498479	0.79226616	0.86804814	0.85254728	Hspd1	1
GO:0034504	protein localization to nucleus	2/138	306/14464	0.00653595	0.6850431	-0.546534	0.79316504	0.86808943	0.85258783	Bmal1/Calr	2
GO:0051347	positive regulation of transferase activity	2/138	306/14464	0.00653595	0.6850431	-0.546534	0.79316504	0.86808943	0.85258783	Fzd8/Sash1	2
GO:0030534	adult behavior	1/138	164/14464	0.00609756	0.63909509	-0.4561976	0.79427073	0.86882788	0.8533131	Hexa	1
GO:0032102	negative regulation of response to external stimulus	2/138	308/14464	0.00649351	0.68059477	-0.5561006	0.79616057	0.87005543	0.85451873	Dtx4/Itch	2
GO:0035051	cardiocyte differentiation	1/138	165/14464	0.00606061	0.63522178	-0.4625134	0.79625609	0.87005543	0.85451873	Calr	1
GO:0042254	ribosome biogenesis	2/138	309/14464	0.00647249	0.6783922	-0.5608635	0.79764402	0.87008768	0.8545504	Mettl16/Tfb1m	2
GO:0032640	tumor necrosis factor production	1/138	166/14464	0.0060241	0.63139515	-0.4687958	0.79822242	0.87008768	0.8545504	Hspd1	1
GO:0061572	actin filament bundle organization	1/138	166/14464	0.0060241	0.63139515	-0.4687958	0.79822242	0.87008768	0.8545504	Hsp90b1	1
GO:1903046	meiotic cell cycle process	1/138	166/14464	0.0060241	0.63139515	-0.4687958	0.79822242	0.87008768	0.8545504	Calr	1
GO:0030100	regulation of endocytosis	2/138	310/14464	0.00645161	0.67620383	-0.5656128	0.79911798	0.87008768	0.8545504	Calr/Lman2	2
GO:0045089	positive regulation of innate immune response	2/138	310/14464	0.00645161	0.67620383	-0.5656128	0.79911798	0.87008768	0.8545504	Hspd1/Itch	2
GO:0071248	cellular response to metal ion	1/138	167/14464	0.00598802	0.62761434	-0.4750453	0.80016992	0.87008768	0.8545504	Calr	1
GO:0098739	import across plasma membrane	1/138	167/14464	0.00598802	0.62761434	-0.4750453	0.80016992	0.87008768	0.8545504	Slc17a8	1
GO:1902905	positive regulation of supramolecular fiber organization	1/138	167/14464	0.00598802	0.62761434	-0.4750453	0.80016992	0.87008768	0.8545504	Scin	1
GO:0009615	response to virus	2/138	312/14464	0.00641026	0.67186919	-0.5750712	0.80203765	0.87030737	0.85476617	Calr/Itch	2
GO:0000910	cytokinesis	1/138	168/14464	0.00595238	0.62387854	-0.4812622	0.80209875	0.87030737	0.85476617	Snx18	1
GO:0016052	carbohydrate catabolic process	1/138	168/14464	0.00595238	0.62387854	-0.4812622	0.80209875	0.87030737	0.85476617	Wdr45b	1
GO:0071706	tumor necrosis factor superfamily cytokine production	1/138	168/14464	0.00595238	0.62387854	-0.4812622	0.80209875	0.87030737	0.85476617	Hspd1	1
GO:0009952	anterior/posterior pattern specification	1/138	169/14464	0.00591716	0.62018695	-0.487447	0.80400911	0.87105743	0.85550283	Smad2	1
GO:0051170	import into nucleus	1/138	169/14464	0.00591716	0.62018695	-0.487447	0.80400911	0.87105743	0.85550283	Bmal1	1
GO:1903131	mononuclear cell differentiation	3/138	447/14464	0.00671141	0.70343352	-0.6251073	0.80408625	0.87105743	0.85550283	Card11/Mmp14/Fzd8	3
GO:0046890	regulation of lipid biosynthetic process	1/138	170/14464	0.00588235	0.61653879	-0.4936001	0.80590115	0.87177436	0.85620696	Rab38	1
GO:0072001	renal system development	2/138	315/14464	0.00634921	0.66547044	-0.5891599	0.80634713	0.87177436	0.85620696	Has2/Smad2	2
GO:0022408	negative regulation of cell-cell adhesion	1/138	171/14464	0.00584795	0.6129333	-0.499722	0.80777506	0.87177436	0.85620696	Itch	1
GO:0030308	negative regulation of cell growth	1/138	171/14464	0.00584795	0.6129333	-0.499722	0.80777506	0.87177436	0.85620696	Rrad	1
GO:0051494	negative regulation of cytoskeleton organization	1/138	171/14464	0.00584795	0.6129333	-0.499722	0.80777506	0.87177436	0.85620696	Scin	1
GO:1902107	positive regulation of leukocyte differentiation	1/138	171/14464	0.00584795	0.6129333	-0.499722	0.80777506	0.87177436	0.85620696	Mmp14	1
GO:1903708	positive regulation of hemopoiesis	1/138	171/14464	0.00584795	0.6129333	-0.499722	0.80777506	0.87177436	0.85620696	Mmp14	1
GO:1902904	negative regulation of supramolecular fiber organization	1/138	172/14464	0.00581395	0.60936973	-0.5058129	0.809631	0.87330984	0.85771503	Scin	1
GO:0030307	positive regulation of cell growth	1/138	173/14464	0.00578035	0.60584737	-0.5118733	0.81146916	0.87405693	0.85844877	Mmp14	1
GO:0071222	cellular response to lipopolysaccharide	1/138	173/14464	0.00578035	0.60584737	-0.5118733	0.81146916	0.87405693	0.85844877	Sash1	1
GO:0062012	regulation of small molecule metabolic process	2/138	319/14464	0.00626959	0.65712598	-0.6077636	0.81196427	0.87405693	0.85844877	Pdk3/Arpp19	2
GO:0001659	temperature homeostasis	1/138	174/14464	0.00574713	0.60236548	-0.5179035	0.81328969	0.87405693	0.85844877	Bmal1	1
GO:0048771	tissue remodeling	1/138	174/14464	0.00574713	0.60236548	-0.5179035	0.81328969	0.87405693	0.85844877	Mmp14	1
GO:0042060	wound healing	2/138	321/14464	0.00623053	0.65303174	-0.6169895	0.81471843	0.87405693	0.85844877	Pdia3/Grhl3	2
GO:0006839	mitochondrial transport	1/138	175/14464	0.00571429	0.5989234	-0.523904	0.81509277	0.87405693	0.85844877	Hspd1	1
GO:0021543	pallium development	1/138	175/14464	0.00571429	0.5989234	-0.523904	0.81509277	0.87405693	0.85844877	Fktn	1
GO:0042770	signal transduction in response to DNA damage	1/138	175/14464	0.00571429	0.5989234	-0.523904	0.81509277	0.87405693	0.85844877	Gnb1l	1
GO:0048469	cell maturation	1/138	175/14464	0.00571429	0.5989234	-0.523904	0.81509277	0.87405693	0.85844877	Kcnip2	1
GO:1902806	regulation of cell cycle G1/S phase transition	1/138	175/14464	0.00571429	0.5989234	-0.523904	0.81509277	0.87405693	0.85844877	Pkp3	1
GO:0048284	organelle fusion	1/138	176/14464	0.00568182	0.59552042	-0.5298751	0.81687857	0.87550621	0.85987217	Sec22b	1
GO:0050866	negative regulation of cell activation	1/138	178/14464	0.00561798	0.58882918	-0.5417304	0.82039895	0.87810828	0.86242778	Itch	1
GO:0051495	positive regulation of cytoskeleton organization	1/138	178/14464	0.00561798	0.58882918	-0.5417304	0.82039895	0.87810828	0.86242778	Scin	1
GO:0001701	in utero embryonic development	3/138	461/14464	0.00650759	0.68207111	-0.6808888	0.8206131	0.87810828	0.86242778	Smad2/Cr1l/Sh3pxd2a	3
GO:0051091	positive regulation of DNA-binding transcription factor activity	1/138	179/14464	0.00558659	0.58553963	-0.5476154	0.82213386	0.87926889	0.86356766	Card11	1
GO:0071219	cellular response to molecule of bacterial origin	1/138	180/14464	0.00555556	0.58228663	-0.5534724	0.82385213	0.88063939	0.86491369	Sash1	1
GO:0000075	cell cycle checkpoint signaling	1/138	181/14464	0.00552486	0.57906958	-0.5593017	0.82555392	0.88152368	0.86578219	Gnb1l	1
GO:0051260	protein homooligomerization	1/138	181/14464	0.00552486	0.57906958	-0.5593017	0.82555392	0.88152368	0.86578219	Card11	1
GO:0042742	defense response to bacterium	1/138	182/14464	0.00549451	0.57588788	-0.5651037	0.82723939	0.88238869	0.86663174	Ighg2c	1
GO:0098742	cell-cell adhesion via plasma-membrane adhesion molecules	1/138	182/14464	0.00549451	0.57588788	-0.5651037	0.82723939	0.88238869	0.86663174	Cdh10	1
GO:0061136	regulation of proteasomal protein catabolic process	1/138	184/14464	0.00543478	0.56962823	-0.5766268	0.83056198	0.88546428	0.86965242	Bag5	1
GO:0044242	cellular lipid catabolic process	1/138	185/14464	0.00540541	0.56654916	-0.5823487	0.83219941	0.88674102	0.87090636	Hexa	1
GO:0001649	osteoblast differentiation	1/138	186/14464	0.00537634	0.56350319	-0.5880444	0.83382112	0.88753083	0.87168206	Ift80	1
GO:0099175	regulation of postsynapse organization	1/138	186/14464	0.00537634	0.56350319	-0.5880444	0.83382112	0.88753083	0.87168206	Zfp804a	1
GO:0006399	tRNA metabolic process	1/138	187/14464	0.00534759	0.56048981	-0.5937143	0.83542728	0.88830242	0.87243988	Yars2	1
GO:1901617	organic hydroxy compound biosynthetic process	1/138	187/14464	0.00534759	0.56048981	-0.5937143	0.83542728	0.88830242	0.87243988	Rab38	1
GO:0018105	peptidyl-serine phosphorylation	1/138	188/14464	0.00531915	0.55750848	-0.5993588	0.83701802	0.88905602	0.87318002	Pdk3	1
GO:0050905	neuromuscular process	1/138	188/14464	0.00531915	0.55750848	-0.5993588	0.83701802	0.88905602	0.87318002	Hexa	1
GO:0060271	cilium assembly	2/138	339/14464	0.00589971	0.61835749	-0.6978605	0.83793712	0.88956358	0.87367851	Ift172/Ift80	2
GO:0061351	neural precursor cell proliferation	1/138	189/14464	0.00529101	0.5545587	-0.604978	0.8385935	0.88979184	0.8739027	Tafa1	1
GO:0015931	nucleobase-containing compound transport	1/138	192/14464	0.00520833	0.54589372	-0.6216873	0.84322978	0.89330071	0.87734891	Slc33a1	1
GO:0031334	positive regulation of protein-containing complex assembly	1/138	192/14464	0.00520833	0.54589372	-0.6216873	0.84322978	0.89330071	0.87734891	Tppp3	1
GO:1901654	response to ketone	1/138	192/14464	0.00520833	0.54589372	-0.6216873	0.84322978	0.89330071	0.87734891	Calr	1
GO:0010563	negative regulation of phosphorus metabolic process	2/138	345/14464	0.0057971	0.60760344	-0.7240008	0.84507657	0.89431725	0.8783473	Chordc1/Dnajc3	2
GO:0045936	negative regulation of phosphate metabolic process	2/138	345/14464	0.0057971	0.60760344	-0.7240008	0.84507657	0.89431725	0.8783473	Chordc1/Dnajc3	2
GO:0045637	regulation of myeloid cell differentiation	1/138	194/14464	0.00515464	0.54026595	-0.6327058	0.84624694	0.89508596	0.87910228	Scin	1
GO:0008361	regulation of cell size	1/138	195/14464	0.00512821	0.53749535	-0.6381796	0.84773383	0.89571877	0.87972379	Oxsr1	1
GO:0045165	cell fate commitment	1/138	195/14464	0.00512821	0.53749535	-0.6381796	0.84773383	0.89571877	0.87972379	Smad2	1
GO:0051963	regulation of synapse assembly	1/138	197/14464	0.00507614	0.53203855	-0.6490574	0.85066492	0.89834494	0.88230306	Lrrn1	1
GO:0002064	epithelial cell development	1/138	198/14464	0.00505051	0.52935149	-0.654462	0.85210939	0.89939923	0.88333853	Bmal1	1
GO:0018209	peptidyl-serine modification	1/138	199/14464	0.00502513	0.52669143	-0.659844	0.85353999	0.90043779	0.88435854	Pdk3	1
GO:0019221	cytokine-mediated signaling pathway	2/138	353/14464	0.00566572	0.59383339	-0.7582614	0.85415496	0.90061528	0.88453286	Ppp2cb/Oxsr1	2
GO:0008406	gonad development	1/138	200/14464	0.005	0.52405797	-0.6652037	0.85495685	0.90098955	0.88490045	Mmp14	1
GO:0000302	response to reactive oxygen species	1/138	201/14464	0.00497512	0.52145072	-0.6705413	0.8563601	0.9010553	0.88496503	Ppp2cb	1
GO:0009636	response to toxic substance	1/138	201/14464	0.00497512	0.52145072	-0.6705413	0.8563601	0.9010553	0.88496503	Akr1a1	1
GO:0031345	negative regulation of cell projection organization	1/138	201/14464	0.00497512	0.52145072	-0.6705413	0.8563601	0.9010553	0.88496503	Bag5	1
GO:0009612	response to mechanical stimulus	1/138	202/14464	0.0049505	0.51886928	-0.675857	0.85774987	0.9015765	0.88547692	Mmp14	1
GO:0035264	multicellular organism growth	1/138	202/14464	0.0049505	0.51886928	-0.675857	0.85774987	0.9015765	0.88547692	Ift80	1
GO:0044770	cell cycle phase transition	3/138	498/14464	0.0060241	0.63139515	-0.8215726	0.85871626	0.90212193	0.88601261	Gnb1l/Pkp3/Arpp19	3
GO:0007623	circadian rhythm	1/138	204/14464	0.00490196	0.51378232	-0.6864238	0.86048949	0.90320685	0.88707815	Bmal1	1
GO:0007018	microtubule-based movement	2/138	359/14464	0.00557103	0.5839086	-0.7835301	0.86064502	0.90320685	0.88707815	Ift172/Ift80	2
GO:0032535	regulation of cellular component size	2/138	365/14464	0.00547945	0.57431011	-0.8084476	0.8668719	0.90926834	0.89303141	Scin/Oxsr1	2
GO:0042063	gliogenesis	2/138	366/14464	0.00546448	0.57274095	-0.8125673	0.86788476	0.90985734	0.89360989	Mmp14/Mettl14	2
GO:0051321	meiotic cell cycle	1/138	211/14464	0.00473934	0.49673741	-0.7227515	0.86967208	0.91125723	0.89498478	Calr	1
GO:0044782	cilium organization	2/138	369/14464	0.00542005	0.56808452	-0.8248703	0.87088134	0.9115201	0.89524296	Ift172/Ift80	2
GO:0002699	positive regulation of immune effector process	1/138	212/14464	0.00471698	0.49439431	-0.7278606	0.87093394	0.9115201	0.89524296	Hspd1	1
GO:0050807	regulation of synapse organization	2/138	370/14464	0.00540541	0.56654916	-0.8289529	0.87186635	0.9115201	0.89524296	Lrrn1/Zfp804a	2
GO:0002274	myeloid leukocyte activation	1/138	213/14464	0.00469484	0.49207321	-0.7329503	0.87218367	0.9115201	0.89524296	Hspd1	1
GO:0043122	regulation of canonical NF-kappaB signal transduction	1/138	213/14464	0.00469484	0.49207321	-0.7329503	0.87218367	0.9115201	0.89524296	Card11	1
GO:0009165	nucleotide biosynthetic process	1/138	214/14464	0.0046729	0.4897738	-0.7380206	0.87342138	0.91234068	0.89604888	Tbpl1	1
GO:0002429	immune response-activating cell surface receptor signaling pathway	1/138	216/14464	0.00462963	0.48523886	-0.7481039	0.87586122	0.91394215	0.89762175	Card11	1
GO:0045926	negative regulation of growth	1/138	216/14464	0.00462963	0.48523886	-0.7481039	0.87586122	0.91394215	0.89762175	Rrad	1
GO:0052548	regulation of endopeptidase activity	1/138	218/14464	0.00458716	0.48078713	-0.7581122	0.87825437	0.91534893	0.89900341	Hspd1	1
GO:1903050	regulation of proteolysis involved in protein catabolic process	1/138	218/14464	0.00458716	0.48078713	-0.7581122	0.87825437	0.91534893	0.89900341	Bag5	1
GO:0006816	calcium ion transport	2/138	377/14464	0.00530504	0.55602968	-0.8572779	0.87857151	0.91534893	0.89900341	Rrad/Sri	2
GO:0006836	neurotransmitter transport	1/138	219/14464	0.00456621	0.47859175	-0.7630886	0.87943371	0.91577395	0.89942084	Slc17a8	1
GO:1903828	negative regulation of protein localization	1/138	221/14464	0.00452489	0.47426061	-0.772987	0.88175848	0.91772075	0.90133288	Ndfip2	1
GO:0090068	positive regulation of cell cycle process	1/138	222/14464	0.0045045	0.4721243	-0.7779092	0.88290412	0.91843896	0.90203826	Pkp3	1
GO:0045861	negative regulation of proteolysis	1/138	224/14464	0.00446429	0.4679089	-0.7877008	0.88516244	0.92031329	0.90387913	Bag5	1
GO:0001819	positive regulation of cytokine production	2/138	386/14464	0.00518135	0.54306525	-0.8930661	0.88672023	0.92145772	0.90500312	Card11/Hspd1	2
GO:0060828	regulation of canonical Wnt signaling pathway	1/138	226/14464	0.00442478	0.46376812	-0.7974227	0.88737751	0.92166567	0.90520735	Bmal1	1
GO:0002768	immune response-regulating cell surface receptor signaling pathway	1/138	227/14464	0.00440529	0.46172508	-0.8022581	0.88846909	0.92232425	0.90585417	Card11	1
GO:0001558	regulation of cell growth	2/138	389/14464	0.00514139	0.53887709	-0.9048437	0.88932282	0.92273536	0.90625794	Mmp14/Rrad	2
GO:1901136	carbohydrate derivative catabolic process	1/138	229/14464	0.00436681	0.45769255	-0.8118781	0.89062084	0.9236068	0.90711382	Hexa	1
GO:0040029	epigenetic regulation of gene expression	1/138	230/14464	0.00434783	0.45570258	-0.8166632	0.89168121	0.92423101	0.90772688	Cbx1	1
GO:0010876	lipid localization	2/138	393/14464	0.00508906	0.53339234	-0.9204325	0.89270737	0.92481915	0.90830452	Slc51a/Hexa	2
GO:0044843	cell cycle G1/S phase transition	1/138	233/14464	0.00429185	0.44983517	-0.8309202	0.89480146	0.92651245	0.90996759	Pkp3	1
GO:0033044	regulation of chromosome organization	1/138	236/14464	0.00423729	0.44411692	-0.8450332	0.89783247	0.92917364	0.91258125	Hnrnpc	1
GO:0022411	cellular component disassembly	2/138	400/14464	0.005	0.52405797	-0.9474055	0.89840167	0.92928567	0.91269128	Scin/Snx14	2
GO:1901987	regulation of cell cycle phase transition	2/138	402/14464	0.00497512	0.52145072	-0.9550419	0.89997658	0.93043732	0.91382237	Gnb1l/Pkp3	2
GO:0007626	locomotory behavior	1/138	240/14464	0.00416667	0.43671498	-0.863633	0.90173934	0.93082771	0.91420578	Hexa	1
GO:0051896	regulation of phosphatidylinositol 3-kinase/protein kinase B signal transduction	1/138	240/14464	0.00416667	0.43671498	-0.863633	0.90173934	0.93082771	0.91420578	Sema3e	1
GO:1901988	negative regulation of cell cycle phase transition	1/138	240/14464	0.00416667	0.43671498	-0.863633	0.90173934	0.93082771	0.91420578	Gnb1l	1
GO:0002366	leukocyte activation involved in immune response	1/138	241/14464	0.00414938	0.43490288	-0.868245	0.90269266	0.93085852	0.91423604	Hspd1	1
GO:0045444	fat cell differentiation	1/138	241/14464	0.00414938	0.43490288	-0.868245	0.90269266	0.93085852	0.91423604	Bmal1	1
GO:0034599	cellular response to oxidative stress	1/138	243/14464	0.00411523	0.43132343	-0.8774243	0.90457183	0.93183712	0.91519717	Bmal1	1
GO:0051259	protein complex oligomerization	1/138	244/14464	0.00409836	0.42955571	-0.8819919	0.90549786	0.93183712	0.91519717	Card11	1
GO:0031349	positive regulation of defense response	2/138	410/14464	0.00487805	0.51127607	-0.9852849	0.90605298	0.93183712	0.91519717	Hspd1/Itch	2
GO:0002263	cell activation involved in immune response	1/138	245/14464	0.00408163	0.42780243	-0.886545	0.90641497	0.93183712	0.91519717	Hspd1	1
GO:0006260	DNA replication	1/138	245/14464	0.00408163	0.42780243	-0.886545	0.90641497	0.93183712	0.91519717	Obi1	1
GO:0032886	regulation of microtubule-based process	1/138	245/14464	0.00408163	0.42780243	-0.886545	0.90641497	0.93183712	0.91519717	Chordc1	1
GO:0097305	response to alcohol	1/138	247/14464	0.00404858	0.42433844	-0.8956081	0.90822277	0.93321972	0.91655508	Smad2	1
GO:0051607	defense response to virus	1/138	248/14464	0.00403226	0.4226274	-0.9001183	0.90911362	0.93365922	0.91698674	Itch	1
GO:0099504	synaptic vesicle cycle	1/138	249/14464	0.00401606	0.4209301	-0.9046144	0.90999589	0.93408946	0.9174093	Slc17a8	1
GO:0050767	regulation of neurogenesis	2/138	418/14464	0.00478469	0.50149088	-1.0150587	0.91178553	0.93532561	0.91862336	Bmal1/Dnajb11	2
GO:0043410	positive regulation of MAPK cascade	2/138	419/14464	0.00477327	0.50029401	-1.0187485	0.91247879	0.93532561	0.91862336	Fzd8/Sash1	2
GO:0010721	negative regulation of cell development	1/138	252/14464	0.00396825	0.41591902	-0.9180197	0.912592	0.93532561	0.91862336	Dnajb11	1
GO:0006898	receptor-mediated endocytosis	1/138	254/14464	0.00393701	0.41264407	-0.9268884	0.9142813	0.93658084	0.91985618	Itch	1
GO:0021537	telencephalon development	1/138	257/14464	0.00389105	0.40782721	-0.9400914	0.91675466	0.93803628	0.92128563	Fktn	1
GO:0031346	positive regulation of cell projection organization	2/138	426/14464	0.00469484	0.49207321	-1.0443824	0.91719141	0.93803628	0.92128563	Hspa5/Zfp804a	2
GO:0045927	positive regulation of growth	1/138	258/14464	0.00387597	0.40624649	-0.9444662	0.91756326	0.93803628	0.92128563	Mmp14	1
GO:0110053	regulation of actin filament organization	1/138	258/14464	0.00387597	0.40624649	-0.9444662	0.91756326	0.93803628	0.92128563	Scin	1
GO:0016042	lipid catabolic process	1/138	259/14464	0.003861	0.40467797	-0.948828	0.91836407	0.9383791	0.92162233	Hexa	1
GO:0051222	positive regulation of protein transport	1/138	262/14464	0.00381679	0.40004425	-0.9618368	0.92072045	0.93983414	0.92305139	Sri	1
GO:0052547	regulation of peptidase activity	1/138	262/14464	0.00381679	0.40004425	-0.9618368	0.92072045	0.93983414	0.92305139	Hspd1	1
GO:0009141	nucleoside triphosphate metabolic process	1/138	265/14464	0.00377358	0.39551545	-0.9747327	0.92300929	0.94169369	0.92487773	Tbpl1	1
GO:0048608	reproductive structure development	1/138	268/14464	0.00373134	0.39108804	-0.9875183	0.92523251	0.94348444	0.92663651	Mmp14	1
GO:0051052	regulation of DNA metabolic process	2/138	443/14464	0.00451467	0.47319004	-1.1052808	0.92766902	0.94497247	0.92809797	Obi1/Hnrnpc	2
GO:0010001	glial cell differentiation	1/138	272/14464	0.00367647	0.38533674	-1.0043988	0.92809797	0.94497247	0.92809797	Mettl14	1
GO:0061458	reproductive system development	1/138	272/14464	0.00367647	0.38533674	-1.0043988	0.92809797	0.94497247	0.92809797	Mmp14	1
GO:0007409	axonogenesis	2/138	446/14464	0.0044843	0.47000715	-1.1158368	0.929384	0.94520812	0.9283294	Sema3e/Crppa	2
GO:0030003	intracellular monoatomic cation homeostasis	2/138	447/14464	0.00447427	0.46895568	-1.1193432	0.92994713	0.94520812	0.9283294	Rab38/Sri	2
GO:0007281	germ cell development	1/138	276/14464	0.00362319	0.37975215	-1.0210934	0.93085437	0.94520812	0.9283294	Tbpl1	1
GO:0006091	generation of precursor metabolites and energy	2/138	449/14464	0.00445434	0.46686679	-1.1263377	0.93106079	0.94520812	0.9283294	Hmgcll1/Wdr45b	2
GO:0010948	negative regulation of cell cycle process	1/138	277/14464	0.00361011	0.37838121	-1.0252387	0.93152691	0.94520812	0.9283294	Gnb1l	1
GO:0032386	regulation of intracellular transport	1/138	277/14464	0.00361011	0.37838121	-1.0252387	0.93152691	0.94520812	0.9283294	Frat2	1
GO:0009117	nucleotide metabolic process	2/138	450/14464	0.00444444	0.46582931	-1.1298259	0.93161137	0.94520812	0.9283294	Tbpl1/Nt5c3b	2
GO:0006874	intracellular calcium ion homeostasis	1/138	278/14464	0.00359712	0.37702012	-1.0293728	0.93219297	0.94532244	0.92844169	Sri	1
GO:0006310	DNA recombination	1/138	279/14464	0.00358423	0.3756688	-1.0334958	0.93285259	0.9454976	0.92861372	Hspd1	1
GO:0006873	intracellular monoatomic ion homeostasis	2/138	454/14464	0.00440529	0.46172508	-1.1437188	0.93377268	0.9454976	0.92861372	Rab38/Sri	2
GO:0098771	inorganic ion homeostasis	2/138	454/14464	0.00440529	0.46172508	-1.1437188	0.93377268	0.9454976	0.92861372	Slc17a8/Sri	2

GO:0007416	synapse assembly	1/138	283/14464	0.00353357	0.37035899	-1.0498785	0.93542797	0.94622317	0.92932633	Lrrn1	1
GO:0032496	response to lipopolysaccharide	1/138	283/14464	0.00353357	0.37035899	-1.0498785	0.93542797	0.94622317	0.92932633	Sash1	1
GO:0009611	response to wounding	2/138	465/14464	0.00430108	0.45080256	-1.1814429	0.93938997	0.94975436	0.93279446	Pdia3/Grhl3	2
GO:0022412	cellular process involved in reproduction in multicellular organism	1/138	295/14464	0.00338983	0.35529354	-1.0980186	0.94258028	0.95202498	0.93502453	Tbpl1	1
GO:0055074	calcium ion homeostasis	1/138	295/14464	0.00338983	0.35529354	-1.0980186	0.94258028	0.95202498	0.93502453	Sri	1
GO:0002237	response to molecule of bacterial origin	1/138	297/14464	0.003367	0.35290099	-1.1059011	0.94369336	0.95267192	0.93565992	Sash1	1
GO:0016049	cell growth	2/138	476/14464	0.00420168	0.44038485	-1.2184884	0.94455624	0.95306576	0.93604672	Mmp14/Rrad	2
GO:0099003	vesicle-mediated transport in synapse	1/138	303/14464	0.00330033	0.34591285	-1.129318	0.94690569	0.95448093	0.93743663	Slc17a8	1
GO:0099173	postsynapse organization	1/138	303/14464	0.00330033	0.34591285	-1.129318	0.94690569	0.95448093	0.93743663	Zfp804a	1
GO:1903532	positive regulation of secretion by cell	1/138	315/14464	0.0031746	0.33273522	-1.1751596	0.95279454	0.95992107	0.94277963	Sri	1
GO:1904951	positive regulation of establishment of protein localization	1/138	316/14464	0.00316456	0.33168226	-1.1789225	0.95325495	0.95992107	0.94277963	Sri	1
GO:0015980	energy derivation by oxidation of organic compounds	1/138	330/14464	0.0030303	0.31761089	-1.23073	0.95925154	0.96547733	0.94823667	Wdr45b	1
GO:0051047	positive regulation of secretion	1/138	334/14464	0.00299401	0.31380717	-1.2452444	0.96081997	0.96657338	0.94931314	Sri	1
GO:0006869	lipid transport	1/138	342/14464	0.00292398	0.30646665	-1.2739095	0.96377925	0.96893945	0.95163696	Slc51a	1
GO:0045786	negative regulation of cell cycle	1/138	343/14464	0.00291545	0.30557316	-1.2774594	0.9641332	0.96893945	0.95163696	Gnb1l	1
GO:0051962	positive regulation of nervous system development	1/138	351/14464	0.002849	0.29860853	-1.3056015	0.96684391	0.97117953	0.95383704	Lrrn1	1
GO:0000280	nuclear division	1/138	360/14464	0.00277778	0.29114332	-1.3367334	0.96965098	0.97302956	0.95565403	Calr	1
GO:0048872	homeostasis of number of cells	1/138	360/14464	0.00277778	0.29114332	-1.3367334	0.96965098	0.97302956	0.95565403	Card11	1
GO:0007059	chromosome segregation	1/138	364/14464	0.00274725	0.28794394	-1.3503974	0.97082158	0.97371955	0.9563317	Srpk1	1
GO:1902903	regulation of supramolecular fiber organization	1/138	374/14464	0.0026738	0.2802449	-1.3841123	0.97355563	0.9759762	0.95854806	Scin	1
GO:0044772	mitotic cell cycle phase transition	1/138	399/14464	0.00250627	0.2626857	-1.4658026	0.97932819	0.98127516	0.96375239	Arpp19	1
GO:0048732	gland development	1/138	408/14464	0.00245098	0.25689116	-1.4943692	0.98108405	0.9824283	0.96488494	Smad2	1
GO:0043434	response to peptide hormone	1/138	410/14464	0.00243902	0.25563803	-1.5006603	0.98145367	0.9824283	0.96488494	Lta4h	1
GO:0048285	organelle fission	1/138	416/14464	0.00240385	0.25195095	-1.5194127	0.98252005	0.98300765	0.96545394	Calr	1
GO:0010720	positive regulation of cell development	1/138	473/14464	0.00211416	0.22158899	-1.6893569	0.99005352	0.99005352	0.97237399	Mmp14	1

ID	Description	GeneRatio	BgRatio	RichFactor	FoldEnrichm	zScore	pvalue	p.adjust	qvalue	geneID	Count
GO:0005504	fatty acid binding	2/23	39/14323	0.05128205	31.93534	7.75818284	0.00176293	0.08015468	0.04256673	Fabp7/Crabp1	2
GO:1901682	sulfur compound transmembrane transporter activity	2/23	52/14323	0.03846154	23.951505	6.64941398	0.00311498	0.08015468	0.04256673	Slc6a13/Slc47a1	2
GO:0033293	monocarboxylic acid binding	2/23	53/14323	0.03773585	23.4995898	6.58109687	0.00323398	0.08015468	0.04256673	Fabp7/Crabp1	2
GO:0022853	active monoatomic ion transmembrane transporter activity	3/23	194/14323	0.01546392	9.62998655	4.85347733	0.00354866	0.08015468	0.04256673	Slc6a13/Slc47a1/Slc9b2	3
GO:0015291	secondary active transmembrane transporter activity	3/23	218/14323	0.01376147	8.56980455	4.5167297	0.00491913	0.08015468	0.04256673	Slc6a13/Slc47a1/Slc9b2	3
GO:0019003	GDP binding	2/23	79/14323	0.02531646	15.7655476	5.27770189	0.00704995	0.08015468	0.04256673	Rab8a/Arl3	2
GO:0015171	amino acid transmembrane transporter activity	2/23	86/14323	0.02325581	14.4823054	5.02923439	0.00830664	0.08015468	0.04256673	Slc6a13/Slc47a1	2
GO:0003924	GTPase activity	3/23	275/14323	0.01090909	6.79351779	3.89044407	0.00933109	0.08015468	0.04256673	Rab8a/Rab13/Arl3	3
GO:0015297	antiporter activity	2/23	107/14323	0.01869159	11.6399837	4.4303892	0.0126281	0.08015468	0.04256673	Slc47a1/Slc9b2	2
GO:0005525	GTP binding	3/23	329/14323	0.00911854	5.67847231	3.44293491	0.01513081	0.08015468	0.04256673	Rab8a/Rab13/Arl3	3
GO:0015078	proton transmembrane transporter activity	2/23	118/14323	0.01694915	10.5549005	4.17969208	0.01520832	0.08015468	0.04256673	Slc47a1/Slc9b2	2
GO:0015081	sodium ion transmembrane transporter activity	2/23	118/14323	0.01694915	10.5549005	4.17969208	0.01520832	0.08015468	0.04256673	Slc6a13/Slc9b2	2
GO:0015203	polyamine transmembrane transporter activity	1/23	10/14323	0.1	62.273913	7.77334627	0.01594752	0.08015468	0.04256673	Slc47a1	1
GO:0019841	retinol binding	1/23	10/14323	0.1	62.273913	7.77334627	0.01594752	0.08015468	0.04256673	Crabp1	1
GO:0140161	monocarboxylate:sodium symporter activity	1/23	10/14323	0.1	62.273913	7.77334627	0.01594752	0.08015468	0.04256673	Slc6a13	1
GO:0022804	active transmembrane transporter activity	3/23	336/14323	0.00892857	5.56017081	3.39223707	0.01600329	0.08015468	0.04256673	Slc6a13/Slc47a1/Slc9b2	3
GO:0019001	guanyl nucleotide binding	3/23	345/14323	0.00869565	5.41512287	3.3291055	0.01716645	0.08015468	0.04256673	Rab8a/Rab13/Arl3	3
GO:0032561	guanyl ribonucleotide binding	3/23	345/14323	0.00869565	5.41512287	3.3291055	0.01716645	0.08015468	0.04256673	Rab8a/Rab13/Arl3	3
GO:1901474	azole transmembrane transporter activity	1/23	11/14323	0.09090909	56.6126482	7.39975753	0.01752882	0.08015468	0.04256673	Slc47a1	1
GO:0015378	sodium:chloride symporter activity	1/23	12/14323	0.08333333	51.8949275	7.07339427	0.0191077	0.08015468	0.04256673	Slc6a13	1
GO:0015385	sodium:proton antiporter activity	1/23	12/14323	0.08333333	51.8949275	7.07339427	0.0191077	0.08015468	0.04256673	Slc9b2	1
GO:0016918	retinal binding	1/23	12/14323	0.08333333	51.8949275	7.07339427	0.0191077	0.08015468	0.04256673	Crabp1	1
GO:0015355	secondary active monocarboxylate transmembrane transporter activity	1/23	13/14323	0.07692308	47.90301	6.78500712	0.02068414	0.08015468	0.04256673	Slc6a13	1
GO:0015373	monoatomic anion:sodium symporter activity	1/23	13/14323	0.07692308	47.90301	6.78500712	0.02068414	0.08015468	0.04256673	Slc6a13	1
GO:0061459	L-arginine transmembrane transporter activity	1/23	13/14323	0.07692308	47.90301	6.78500712	0.02068414	0.08015468	0.04256673	Slc47a1	1
GO:0031406	carboxylic acid binding	2/23	141/14323	0.0141844	8.83317916	3.74866481	0.0212658	0.08015468	0.04256673	Fabp7/Crabp1	2
GO:0046943	carboxylic acid transmembrane transporter activity	2/23	144/14323	0.01388889	8.64915459	3.69972647	0.02211932	0.08015468	0.04256673	Slc6a13/Slc47a1	2
GO:0001846	opsonin binding	1/23	14/14323	0.07142857	44.4813665	6.52770161	0.02225817	0.08015468	0.04256673	Megf10	1
GO:0015929	hexosaminidase activity	1/23	14/14323	0.07142857	44.4813665	6.52770161	0.02225817	0.08015468	0.04256673	Hexb	1
GO:0046703	natural killer cell lectin-like receptor binding	1/23	14/14323	0.07142857	44.4813665	6.52770161	0.02225817	0.08015468	0.04256673	Mill2	1
GO:0005342	organic acid transmembrane transporter activity	2/23	145/14323	0.0137931	8.58950525	3.68372935	0.02240699	0.08015468	0.04256673	Slc6a13/Slc47a1	2
GO:0043177	organic acid binding	2/23	149/14323	0.01342282	8.3589145	3.62124919	0.02357327	0.08015468	0.04256673	Fabp7/Crabp1	2
GO:0034236	protein kinase A catalytic subunit binding	1/23	15/14323	0.06666667	41.515942	6.29621919	0.02382977	0.08015468	0.04256673	Rab13	1
GO:0015174	basic amino acid transmembrane transporter activity	1/23	16/14323	0.0625	38.9211957	6.08646962	0.02539896	0.08292012	0.04403534	Slc47a1	1
GO:0031489	myosin V binding	1/23	17/14323	0.05882353	36.6317136	5.89521696	0.02696573	0.08551988	0.04541597	Rab8a	1
GO:0001848	complement binding	1/23	18/14323	0.05555556	34.5966184	5.71986293	0.02853009	0.08559028	0.04545335	Megf10	1
GO:0015377	chloride:monoatomic cation symporter activity	1/23	18/14323	0.05555556	34.5966184	5.71986293	0.02853009	0.08559028	0.04545335	Slc6a13	1
GO:0005283	amino acid:sodium symporter activity	1/23	20/14323	0.05	31.1369565	5.4087705	0.03165161	0.08636412	0.0458643	Slc6a13	1
GO:0015296	monoatomic anion:monoatomic cation symporter activity	1/23	20/14323	0.05	31.1369565	5.4087705	0.03165161	0.08636412	0.0458643	Slc6a13	1
GO:0051139	metal cation:proton antiporter activity	1/23	20/14323	0.05	31.1369565	5.4087705	0.03165161	0.08636412	0.0458643	Slc9b2	1
GO:0005044	scavenger receptor activity	1/23	21/14323	0.04761905	29.6542443	5.26984636	0.03320876	0.08636412	0.0458643	Megf10	1
GO:0019956	chemokine binding	1/23	21/14323	0.04761905	29.6542443	5.26984636	0.03320876	0.08636412	0.0458643	Plp2	1
GO:0022890	inorganic cation transmembrane transporter activity	3/23	446/14323	0.00672646	4.18882823	2.74377501	0.03345637	0.08636412	0.0458643	Slc6a13/Slc47a1/Slc9b2	3
GO:0005501	retinoid binding	1/23	22/14323	0.04545455	28.3063241	5.14030764	0.03476352	0.08756484	0.04650195	Crabp1	1
GO:0005112	Notch binding	1/23	23/14323	0.04347826	27.0756144	5.01912698	0.03631589	0.08756484	0.04650195	Megf10	1
GO:0019840	isoprenoid binding	1/23	23/14323	0.04347826	27.0756144	5.01912698	0.03631589	0.08756484	0.04650195	Crabp1	1
GO:0005416	amino acid:monoatomic cation symporter activity	1/23	24/14323	0.04166667	25.9474638	4.90542819	0.03786588	0.08756484	0.04650195	Slc6a13	1
GO:0090482	vitamin transmembrane transporter activity	1/23	24/14323	0.04166667	25.9474638	4.90542819	0.03786588	0.08756484	0.04650195	Slc47a1	1
GO:0005343	organic acid:sodium symporter activity	1/23	25/14323	0.04	24.9095652	4.79845881	0.03941347	0.08802377	0.04674567	Slc6a13	1
GO:0008324	monoatomic cation transmembrane transporter activity	3/23	477/14323	0.00628931	3.9165983	2.59819282	0.03965035	0.08802377	0.04674567	Slc6a13/Slc47a1/Slc9b2	3
GO:0072349	modified amino acid transmembrane transporter activity	1/23	26/14323	0.03846154	23.951505	4.69756847	0.04095869	0.08901263	0.04727081	Slc6a13	1
GO:0008514	organic anion transmembrane transporter activity	2/23	204/14323	0.00980392	6.10528559	2.94530918	0.04197956	0.08901263	0.04727081	Slc6a13/Slc47a1	2
GO:0042910	xenobiotic transmembrane transporter activity	1/23	27/14323	0.03703704	23.0644122	4.60219175	0.04250153	0.08901263	0.04727081	Slc47a1	1
GO:0016757	glycosyltransferase activity	2/23	217/14323	0.00921659	5.73953116	2.82137649	0.0469234	0.09645365	0.05122242	Glt1d1/Hexb	2
GO:0042887	amide transmembrane transporter activity	1/23	31/14323	0.03225806	20.088359	4.26678414	0.04864917	0.09818287	0.05214074	Slc47a1	1
GO:0017080	sodium channel regulator activity	1/23	33/14323	0.03030303	18.8708827	4.12177798	0.05170881	0.10069611	0.05347541	Fxyd3	1
GO:0140828	metal cation:monoatomic cation antiporter activity	1/23	33/14323	0.03030303	18.8708827	4.12177798	0.05170881	0.10069611	0.05347541	Slc9b2	1
GO:0008375	acetylglucosaminyltransferase activity	1/23	36/14323	0.02777778	17.2983092	3.92663935	0.05628064	0.1077095	0.05719993	Hexb	1
GO:0030291	protein serine/threonine kinase inhibitor activity	1/23	38/14323	0.02631579	16.3878719	3.80914909	0.0593168	0.11159602	0.05926389	Inka1	1
GO:0051879	Hsp90 protein binding	1/23	46/14323	0.02173913	13.5378072	3.41570029	0.07136828	0.13203133	0.07011621	Npas2	1
GO:0015370	solute:sodium symporter activity	1/23	49/14323	0.02040816	12.7089618	3.29261778	0.0758494	0.13590416	0.07217291	Slc6a13	1
GO:0004857	enzyme inhibitor activity	2/23	285/14323	0.00701754	4.37009916	2.30467783	0.07591043	0.13590416	0.07217291	Angptl4/Inka1	2
GO:0016597	amino acid binding	1/23	50/14323	0.02	12.4547826	3.25395809	0.0773385	0.13626308	0.07236351	Slc6a13	1
GO:0001786	phosphatidylserine binding	1/23	53/14323	0.01886792	11.7497949	3.14429917	0.08179206	0.13967567	0.07417579	Marcks	1
GO:0051018	protein kinase A binding	1/23	53/14323	0.01886792	11.7497949	3.14429917	0.08179206	0.13967567	0.07417579	Rab13	1
GO:0038024	cargo receptor activity	1/23	54/14323	0.01851852	11.5322061	3.1096907	0.083272	0.14004836	0.07437372	Megf10	1
GO:0008028	monocarboxylic acid transmembrane transporter activity	1/23	56/14323	0.01785714	11.1203416	3.04313059	0.08622504	0.14285044	0.07586178	Slc6a13	1
GO:0005080	protein kinase C binding	1/23	58/14323	0.01724138	10.7368816	2.9798591	0.08916899	0.14555526	0.07729819	Marcks	1
GO:0015179	L-amino acid transmembrane transporter activity	1/23	60/14323	0.01666667	10.3789855	2.91960288	0.09210386	0.14816707	0.07868522	Slc47a1	1
GO:0004553	hydrolase activity, hydrolyzing O-glycosyl compounds	1/23	67/14323	0.01492537	9.29461389	2.7291818	0.10230483	0.16212696	0.08609872	Hexb	1
GO:0017022	myosin binding	1/23	68/14323	0.01470588	9.15792839	2.70426015	0.10375313	0.16212696	0.08609872	Rab8a	1
GO:0046873	metal ion transmembrane transporter activity	2/23	346/14323	0.00578035	3.59964815	1.9631043	0.10562165	0.16212696	0.08609872	Slc6a13/Slc9b2	2
GO:0015101	organic cation transmembrane transporter activity	1/23	70/14323	0.01428571	8.89627329	2.65592485	0.10664303	0.16212696	0.08609872	Slc47a1	1
GO:0004860	protein kinase inhibitor activity	1/23	71/14323	0.01408451	8.77097367	2.63247611	0.10808464	0.16212696	0.08609872	Inka1	1
GO:0019210	kinase inhibitor activity	1/23	74/14323	0.01351351	8.41539365	2.56481244	0.11239612	0.16634626	0.08833941	Inka1	1
GO:0015294	solute:monoatomic cation symporter activity	1/23	77/14323	0.01298701	8.08752117	2.5008693	0.11668766	0.17026788	0.09042201	Slc6a13	1
GO:0043178	alcohol binding	1/23	78/14323	0.01282051	7.98383501	2.48032036	0.11811376	0.17026788	0.09042201	Crabp1	1
GO:0015108	chloride transmembrane transporter activity	1/23	80/14323	0.0125	7.78423913	2.44029954	0.12095935	0.17213445	0.09141327	Slc6a13	1
GO:0072341	modified amino acid binding	1/23	86/14323	0.01162791	7.24115268	2.32810479	0.12944352	0.18187634	0.09658677	Marcks	1
GO:0140678	molecular function inhibitor activity	2/23	399/14323	0.00501253	3.1214994	1.72363414	0.13361652	0.18539292	0.09845427	Angptl4/Inka1	2
GO:0051117	ATPase binding	1/23	93/14323	0.01075269	6.69611968	2.21012005	0.13924273	0.19081411	0.10133324	Fxyd3	1
GO:0008509	monoatomic anion transmembrane transporter activity	1/23	100/14323	0.01	6.2273913	2.10371615	0.14893639	0.19680879	0.10451676	Slc6a13	1
GO:0016798	hydrolase activity, acting on glycosyl bonds	1/23	100/14323	0.01	6.2273913	2.10371615	0.14893639	0.19680879	0.10451676	Hexb	1
GO:0019955	cytokine binding	1/23	100/14323	0.01	6.2273913	2.10371615	0.14893639	0.19680879	0.10451676	Plp2	1
GO:0015103	inorganic anion transmembrane transporter activity	1/23	103/14323	0.00970874	6.04601098	2.0611744	0.15305876	0.19987674	0.10614602	Slc6a13	1
GO:0015293	symporter activity	1/23	106/14323	0.00943396	5.87489746	2.02028282	0.15716204	0.20284868	0.10772429	Slc6a13	1
GO:1990782	protein tyrosine kinase binding	1/23	108/14323	0.00925926	5.76610306	1.99388272	0.15988698	0.20399374	0.10833238	Rab8a	1
GO:0019842	vitamin binding	1/23	110/14323	0.00909091	5.66126482	1.96813541	0.1626035	0.20510214	0.10892101	Crabp1	1
GO:0008194	UDP-glycosyltransferase activity	1/23	114/14323	0.00877193	5.46262395	1.91848627	0.16801135	0.20954225	0.11127896	Hexb	1
GO:0099106	ion channel regulator activity	1/23	138/14323	0.00724638	4.51260239	1.66284435	0.19976361	0.24637511	0.13083932	Fxyd3	1
GO:0031072	heat shock protein binding	1/23	144/14323	0.00694444	4.32457729	1.60802432	0.20751865	0.25312714	0.13442503	Npas2	1
GO:0016247	channel regulator activity	1/23	148/14323	0.00675676	4.20769683	1.57311464	0.21264868	0.25412124	0.13495296	Fxyd3	1
GO:0016758	hexosyltransferase activity	1/23	150/14323	0.00666667	4.1515942	1.55611885	0.21520177	0.25412124	0.13495296	Hexb	1
GO:0141108	transporter regulator activity	1/23	150/14323	0.00666667	4.1515942	1.55611885	0.21520177	0.25412124	0.13495296	Fxyd3	1
GO:0005516	calmodulin binding	1/23	178/14323	0.00561798	3.49853444	1.34521468	0.25012403	0.29225018	0.15520161	Marcks	1
GO:0051015	actin filament binding	1/23	201/14323	0.00497512	3.09820463	1.20142178	0.27769329	0.32108287	0.17051342	Marcks	1
GO:0030246	carbohydrate binding	1/23	204/14323	0.00490196	3.0526428	1.18419774	0.28121698	0.321805	0.17089692	Hexb	1
GO:0019887	protein kinase regulator activity	1/23	210/14323	0.0047619	2.96542443	1.15067866	0.2882151	0.32574944	0.17299164	Inka1	1
GO:0000287	magnesium ion binding	1/23	212/14323	0.00471698	2.93744873	1.13976938	0.29053329	0.32574944	0.17299164	Arl3	1
GO:0019207	kinase regulator activity	1/23	242/14323	0.00413223	2.57330219	0.98992163	0.32445129	0.36014093	0.1912555	Inka1	1
GO:0008017	microtubule binding	1/23	248/14323	0.00403226	2.51104488	0.96266845	0.33104615	0.363823	0.19321089	Arl3	1
GO:0031267	small GTPase binding	1/23	284/14323	0.00352113	2.19274342	0.8142089	0.36934065	0.3980273	0.21137533	Rab8a	1
GO:0048018	receptor ligand activity	1/23	284/14323	0.00352113	2.19274342	0.8142089	0.36934065	0.3980273	0.21137533	Mill2	1
GO:0030546	signaling receptor activator activity	1/23	289/14323	0.00346021	2.15480668	0.79536268	0.37449051	0.3996966	0.21226183	Mill2	1
GO:0030545	signaling receptor regulator activity	1/23	319/14323	0.0031348	1.95216028	0.68972678	0.40455507	0.4276725	0.22711864	Mill2	1
GO:0051020	GTPase binding	1/23	324/14323	0.00308642	1.92203435	0.67323833	0.40942949	0.4287422	0.22768671	Rab8a	1
GO:0015631	tubulin binding	1/23	353/14323	0.00283286	1.76413351	0.58298332	0.43695711	0.45289675	0.24051416	Arl3	1
GO:0001228	DNA-binding transcription activator activity, RNA polymerase II-specific	1/23	357/14323	0.00280112	1.74436731	0.57119332	0.4406563	0.45289675	0.24051416	Pbx3	1
GO:0001216	DNA-binding transcription activator activity	1/23	363/14323	0.00275482	1.71553479	0.55378233	0.44616153	0.45434798	0.24128485	Pbx3	1
GO:0003779	actin binding	1/23	402/14323	0.00248756	1.54910231	0.44784612	0.48070231	0.48507233	0.25760124	Marcks	1
GO:0005543	phospholipid binding	1/23	428/14323	0.00233645	1.45499797	0.38326556	0.50257419	0.50257419	0.26689573	Marcks	1

ID	Description	GeneRatio	BgRatio	RichFactor	FoldEnrichm	zScore	pvalue	p.adjust	qvalue	geneID	Count
GO:0031492	nucleosomal DNA binding	2/29	14/14323	0.14285714	70.5566502	11.7279466	0.00035482	0.04259452	0.02756829	H3f3b/H1f0	2
GO:1901681	sulfur compound binding	4/29	225/14323	0.01777778	8.78038314	5.29831085	0.00103442	0.04259452	0.02756829	Fbn1/Gstm7/Ccn3/Comp	4
GO:0005201	extracellular matrix structural constituent	3/29	121/14323	0.02479339	12.2453691	5.59519966	0.00183059	0.04259452	0.02756829	Fbn1/Comp/Col6a1	3
GO:0019838	growth factor binding	3/29	124/14323	0.02419355	11.9491101	5.515498	0.00196334	0.04259452	0.02756829	Igfbp5/Col6a1/Kl	3
GO:0001968	fibronectin binding	2/29	33/14323	0.06060606	29.9331243	7.49480148	0.00201023	0.04259452	0.02756829	Igfbp5/Comp	2
GO:0008201	heparin binding	3/29	129/14323	0.02325581	11.4859663	5.38858649	0.00219769	0.04259452	0.02756829	Fbn1/Ccn3/Comp	3
GO:0005178	integrin binding	3/29	133/14323	0.02255639	11.1405237	5.29198925	0.0023972	0.04259452	0.02756829	Fbn1/Ccn3/Comp	3
GO:0048018	receptor ligand activity	4/29	284/14323	0.01408451	6.95628946	4.5665774	0.00243158	0.04259452	0.02756829	Fbn1/Igfbp5/Ccn3/Bmp6	4
GO:0030546	signaling receptor activator activity	4/29	289/14323	0.01384083	6.83593843	4.5143251	0.00259021	0.04259452	0.02756829	Fbn1/Igfbp5/Ccn3/Bmp6	4
GO:0030545	signaling receptor regulator activity	4/29	319/14323	0.01253918	6.19306021	4.22490155	0.00369466	0.04995604	0.03233286	Fbn1/Igfbp5/Ccn3/Bmp6	4
GO:0031491	nucleosome binding	2/29	45/14323	0.04444444	21.9509579	6.3401626	0.00371295	0.04995604	0.03233286	H3f3b/H1f0	2
GO:0005539	glycosaminoglycan binding	3/29	178/14323	0.01685393	8.32409919	4.42880631	0.00543801	0.06706878	0.04340867	Fbn1/Ccn3/Comp	3
GO:0005179	hormone activity	2/29	61/14323	0.03278689	16.1933296	5.35613611	0.00672733	0.07658807	0.0495698	Fbn1/Ccn3	2
GO:0005518	collagen binding	2/29	64/14323	0.03125	15.4342672	5.2127169	0.00738336	0.07805268	0.05051774	Comp/Col6a1	2
GO:0050839	cell adhesion molecule binding	3/29	257/14323	0.01167315	5.7653294	3.47214718	0.01478761	0.10738273	0.06950091	Fbn1/Ccn3/Comp	3
GO:0008083	growth factor activity	2/29	92/14323	0.02173913	10.7368816	4.22007371	0.01480449	0.10738273	0.06950091	Ccn3/Bmp6	2
GO:0031490	chromatin DNA binding	2/29	96/14323	0.02083333	10.2895115	4.11335102	0.01604708	0.10738273	0.06950091	H3f3b/H1f0	2
GO:0019955	cytokine binding	2/29	100/14323	0.02	9.87793103	4.01273155	0.01733302	0.10738273	0.06950091	Cx3cr1/Comp	2
GO:0016493	C-C chemokine receptor activity	1/29	10/14323	0.1	49.3896552	6.89463273	0.02006992	0.10738273	0.06950091	Cx3cr1	1
GO:0019870	potassium channel inhibitor activity	1/29	10/14323	0.1	49.3896552	6.89463273	0.02006992	0.10738273	0.06950091	Kcnk2	1
GO:0001637	G protein-coupled chemoattractant receptor activity	1/29	11/14323	0.09090909	44.8996865	6.56041847	0.02205539	0.10738273	0.06950091	Cx3cr1	1
GO:0004950	chemokine receptor activity	1/29	11/14323	0.09090909	44.8996865	6.56041847	0.02205539	0.10738273	0.06950091	Cx3cr1	1
GO:0019957	C-C chemokine binding	1/29	11/14323	0.09090909	44.8996865	6.56041847	0.02205539	0.10738273	0.06950091	Cx3cr1	1
GO:0031994	insulin-like growth factor I binding	1/29	11/14323	0.09090909	44.8996865	6.56041847	0.02205539	0.10738273	0.06950091	Igfbp5	1
GO:0070700	BMP receptor binding	1/29	11/14323	0.09090909	44.8996865	6.56041847	0.02205539	0.10738273	0.06950091	Bmp6	1
GO:0022841	potassium ion leak channel activity	1/29	12/14323	0.08333333	41.158046	6.26833429	0.02403697	0.10738273	0.06950091	Kcnk2	1
GO:0048407	platelet-derived growth factor binding	1/29	12/14323	0.08333333	41.158046	6.26833429	0.02403697	0.10738273	0.06950091	Col6a1	1
GO:0005520	insulin-like growth factor binding	1/29	14/14323	0.07142857	35.2783251	5.7796683	0.02798851	0.10738273	0.06950091	Igfbp5	1
GO:0015149	hexose transmembrane transporter activity	1/29	14/14323	0.07142857	35.2783251	5.7796683	0.02798851	0.10738273	0.06950091	Slc2a10	1
GO:0016641	oxidoreductase activity, acting on the CH-NH2 group of donors, oxygen as acceptor	1/29	14/14323	0.07142857	35.2783251	5.7796683	0.02798851	0.10738273	0.06950091	Loxl1	1
GO:0016805	dipeptidase activity	1/29	14/14323	0.07142857	35.2783251	5.7796683	0.02798851	0.10738273	0.06950091	Cpq	1
GO:0036122	BMP binding	1/29	14/14323	0.07142857	35.2783251	5.7796683	0.02798851	0.10738273	0.06950091	Comp	1
GO:0055056	D-glucose transmembrane transporter activity	1/29	14/14323	0.07142857	35.2783251	5.7796683	0.02798851	0.10738273	0.06950091	Slc2a10	1
GO:0008381	mechanosensitive monoatomic ion channel activity	1/29	15/14323	0.06666667	32.9264368	5.57224957	0.02995848	0.10738273	0.06950091	Kcnk2	1
GO:0015145	monosaccharide transmembrane transporter activity	1/29	15/14323	0.06666667	32.9264368	5.57224957	0.02995848	0.10738273	0.06950091	Slc2a10	1
GO:0022840	leak channel activity	1/29	15/14323	0.06666667	32.9264368	5.57224957	0.02995848	0.10738273	0.06950091	Kcnk2	1
GO:0022842	narrow pore channel activity	1/29	15/14323	0.06666667	32.9264368	5.57224957	0.02995848	0.10738273	0.06950091	Kcnk2	1
GO:0030527	structural constituent of chromatin	1/29	15/14323	0.06666667	32.9264368	5.57224957	0.02995848	0.10738273	0.06950091	H1f0	1
GO:0042813	Wnt receptor activity	1/29	15/14323	0.06666667	32.9264368	5.57224957	0.02995848	0.10738273	0.06950091	Fzd7	1
GO:0043295	glutathione binding	1/29	15/14323	0.06666667	32.9264368	5.57224957	0.02995848	0.10738273	0.06950091	Gstm7	1
GO:0055105	ubiquitin-protein transferase inhibitor activity	1/29	15/14323	0.06666667	32.9264368	5.57224957	0.02995848	0.10738273	0.06950091	Gbp4	1
GO:0016638	oxidoreductase activity, acting on the CH-NH2 group of donors	1/29	16/14323	0.0625	30.8685345	5.38422948	0.0319246	0.10738273	0.06950091	Loxl1	1
GO:0051119	sugar transmembrane transporter activity	1/29	16/14323	0.0625	30.8685345	5.38422948	0.0319246	0.10738273	0.06950091	Slc2a10	1
GO:1900750	oligopeptide binding	1/29	16/14323	0.0625	30.8685345	5.38422948	0.0319246	0.10738273	0.06950091	Gstm7	1
GO:0005104	fibroblast growth factor receptor binding	1/29	17/14323	0.05882353	29.0527383	5.2127219	0.03388687	0.11145013	0.07213344	Kl	1
GO:0043395	heparan sulfate proteoglycan binding	1/29	18/14323	0.05555556	27.4386973	5.05540925	0.03584529	0.11417109	0.07389452	Comp	1
GO:0004602	glutathione peroxidase activity	1/29	19/14323	0.05263158	25.9945554	4.91040609	0.03779989	0.11417109	0.07389452	Gstm7	1
GO:0005537	D-mannose binding	1/29	19/14323	0.05263158	25.9945554	4.91040609	0.03779989	0.11417109	0.07389452	Asgr1	1
GO:0070696	transmembrane receptor protein serine/threonine kinase binding	1/29	19/14323	0.05263158	25.9945554	4.91040609	0.03779989	0.11417109	0.07389452	Bmp6	1
GO:0015271	outward rectifier potassium channel activity	1/29	21/14323	0.04761905	23.5188834	4.65138253	0.04169761	0.12100483	0.07831749	Kcnk2	1
GO:0019956	chemokine binding	1/29	21/14323	0.04761905	23.5188834	4.65138253	0.04169761	0.12100483	0.07831749	Cx3cr1	1
GO:0015144	carbohydrate transmembrane transporter activity	1/29	22/14323	0.04545455	22.4498433	4.53498865	0.04364075	0.12265186	0.07938349	Slc2a10	1
GO:0000979	RNA polymerase II core promoter sequence-specific DNA binding	1/29	23/14323	0.04347826	21.4737631	4.42606226	0.04558008	0.12265186	0.07938349	H3f3b	1
GO:0005112	Notch binding	1/29	23/14323	0.04347826	21.4737631	4.42606226	0.04558008	0.12265186	0.07938349	Ccn3	1
GO:0017134	fibroblast growth factor binding	1/29	23/14323	0.04347826	21.4737631	4.42606226	0.04558008	0.12265186	0.07938349	Kl	1
GO:0004364	glutathione transferase activity	1/29	24/14323	0.04166667	20.579023	4.32382142	0.04751562	0.12337389	0.07985081	Gstm7	1
GO:0090482	vitamin transmembrane transporter activity	1/29	24/14323	0.04166667	20.579023	4.32382142	0.04751562	0.12337389	0.07985081	Slc2a10	1
GO:0033612	receptor serine/threonine kinase binding	1/29	26/14323	0.03846154	18.9960212	4.13680126	0.05137533	0.13109567	0.08484855	Bmp6	1
GO:0017147	Wnt-protein binding	1/29	28/14323	0.03571429	17.6391626	3.96955977	0.05521994	0.13851781	0.08965235	Fzd7	1
GO:0005109	frizzled binding	1/29	31/14323	0.03225806	15.9321468	3.74869874	0.06095865	0.15036468	0.09731996	Fzd7	1
GO:0004180	carboxypeptidase activity	1/29	32/14323	0.03125	15.4342672	3.68181842	0.06286407	0.15214811	0.09847424	Cpq	1
GO:0000217	DNA secondary structure binding	1/29	33/14323	0.03030303	14.9665622	3.61788135	0.06476575	0.15214811	0.09847424	H1f0	1
GO:0055106	ubiquitin-protein transferase regulator activity	1/29	33/14323	0.03030303	14.9665622	3.61788135	0.06476575	0.15214811	0.09847424	Gbp4	1
GO:0042379	chemokine receptor binding	1/29	35/14323	0.02857143	14.11133	3.49799348	0.06855794	0.15797536	0.10224578	Cx3cr1	1
GO:0038187	pattern recognition receptor activity	1/29	36/14323	0.02777778	13.7193487	3.44167253	0.07044847	0.15797536	0.10224578	Asgr1	1
GO:0043394	proteoglycan binding	1/29	36/14323	0.02777778	13.7193487	3.44167253	0.07044847	0.15797536	0.10224578	Comp	1
GO:0042162	telomeric DNA binding	1/29	37/14323	0.02702703	13.3485555	3.38754942	0.07233529	0.15970299	0.10336396	Telo2	1
GO:0030020	extracellular matrix structural constituent conferring tensile strength	1/29	38/14323	0.02631579	12.9972777	3.33547993	0.07421841	0.15970299	0.10336396	Col6a1	1
GO:0001664	G protein-coupled receptor binding	2/29	223/14323	0.00896861	4.42956549	2.32490879	0.07445613	0.15970299	0.10336396	Cx3cr1/Fzd7	2
GO:0005504	fatty acid binding	1/29	39/14323	0.02564103	12.6640141	3.28533277	0.07609784	0.16064495	0.10397362	Gstm7	1
GO:0004930	G protein-coupled receptor activity	2/29	231/14323	0.00865801	4.27616062	2.2610429	0.07912684	0.16064495	0.10397362	Cx3cr1/Fzd7	2
GO:0004601	peroxidase activity	1/29	41/14323	0.02439024	12.0462574	3.19033667	0.07984566	0.16064495	0.10397362	Gstm7	1
GO:0008200	ion channel inhibitor activity	1/29	41/14323	0.02439024	12.0462574	3.19033667	0.07984566	0.16064495	0.10397362	Kcnk2	1
GO:0016248	channel inhibitor activity	1/29	42/14323	0.02380952	11.7594417	3.14527786	0.08171406	0.16064495	0.10397362	Kcnk2	1
GO:0141110	transporter inhibitor activity	1/29	42/14323	0.02380952	11.7594417	3.14527786	0.08171406	0.16064495	0.10397362	Kcnk2	1
GO:0003777	microtubule motor activity	1/29	43/14323	0.02325581	11.4859663	3.10171955	0.08357879	0.16064495	0.10397362	Kifc3	1
GO:0016684	oxidoreductase activity, acting on peroxide as acceptor	1/29	43/14323	0.02325581	11.4859663	3.10171955	0.08357879	0.16064495	0.10397362	Gstm7	1
GO:0001046	core promoter sequence-specific DNA binding	1/29	45/14323	0.02222222	10.9754789	3.01877169	0.0872973	0.16564103	0.10720721	H3f3b	1
GO:0051879	Hsp90 protein binding	1/29	46/14323	0.02173913	10.7368816	2.97923162	0.08915109	0.16627387	0.1076168	Telo2	1
GO:0046982	protein heterodimerization activity	2/29	249/14323	0.00803213	3.96704058	2.12734698	0.08996402	0.16627387	0.1076168	Bmp6/Kcnk2	2
GO:0005507	copper ion binding	1/29	47/14323	0.0212766	10.5084373	2.94088979	0.09100124	0.16627387	0.1076168	Loxl1	1
GO:0008235	metalloexopeptidase activity	1/29	48/14323	0.02083333	10.2895115	2.9036842	0.09284776	0.16757889	0.10846144	Cpq	1
GO:0033293	monocarboxylic acid binding	1/29	53/14323	0.01886792	9.31880286	2.7328181	0.10202619	0.18082306	0.11703342	Gstm7	1
GO:0015459	potassium channel regulator activity	1/29	54/14323	0.01851852	9.14623244	2.70134998	0.10385108	0.18082306	0.11703342	Kcnk2	1
GO:0038024	cargo receptor activity	1/29	54/14323	0.01851852	9.14623244	2.70134998	0.10385108	0.18082306	0.11703342	Asgr1	1
GO:0003924	GTPase activity	2/29	275/14323	0.00727273	3.59197492	1.9548518	0.10634614	0.18285678	0.11834969	Gbp4/Gpn3	2
GO:0016765	transferase activity, transferring alkyl or aryl (other than methyl) groups	1/29	56/14323	0.01785714	8.81958128	2.64079766	0.10749013	0.18285678	0.11834969	Gstm7	1
GO:0004896	cytokine receptor activity	1/29	59/14323	0.01694915	8.371128	2.55542558	0.11292197	0.18991423	0.12291746	Cx3cr1	1
GO:0016209	antioxidant activity	1/29	63/14323	0.01587302	7.83962781	2.45056895	0.1201148	0.19974146	0.1292779	Gstm7	1
GO:0004553	hydrolase activity, hydrolyzing O-glycosyl compounds	1/29	67/14323	0.01492537	7.37159032	2.35456317	0.12725129	0.20925768	0.13543705	Kl	1
GO:0048029	monosaccharide binding	1/29	69/14323	0.01449275	7.15792104	2.30948012	0.13079855	0.21272731	0.13768268	Asgr1	1
GO:0005546	phosphatidylinositol-4,5-bisphosphate binding	1/29	72/14323	0.01388889	6.85967433	2.24512746	0.13609335	0.21657866	0.14017538	Fzd7	1
GO:0019843	rRNA binding	1/29	72/14323	0.01388889	6.85967433	2.24512746	0.13609335	0.21657866	0.14017538	Rpl8	1
GO:0005525	GTP binding	2/29	329/14323	0.00607903	3.00241065	1.65502327	0.14263883	0.2236626	0.14476029	Gbp4/Gpn3	2
GO:0003774	cytoskeletal motor activity	1/29	77/14323	0.01298701	6.41424093	2.14565758	0.14484888	0.2236626	0.14476029	Kifc3	1
GO:0005249	voltage-gated potassium channel activity	1/29	78/14323	0.01282051	6.33200707	2.12682	0.14658968	0.2236626	0.14476029	Kcnk2	1
GO:0008238	exopeptidase activity	1/29	78/14323	0.01282051	6.33200707	2.12682	0.14658968	0.2236626	0.14476029	Cpq	1
GO:0140375	immune receptor activity	1/29	80/14323	0.0125	6.1737069	2.09011423	0.15006101	0.22662275	0.14667618	Cx3cr1	1
GO:0019001	guanyl nucleotide binding	2/29	345/14323	0.0057971	2.86316842	1.57784046	0.15386824	0.22772499	0.14738958	Gbp4/Gpn3	2
GO:0032561	guanyl ribonucleotide binding	2/29	345/14323	0.0057971	2.86316842	1.57784046	0.15386824	0.22772499	0.14738958	Gbp4/Gpn3	2
GO:0005496	steroid binding	1/29	86/14323	0.01162791	5.74298316	1.98708072	0.16039341	0.23272769	0.15062745	Comp	1
GO:0072341	modified amino acid binding	1/29	86/14323	0.01162791	5.74298316	1.98708072	0.16039341	0.23272769	0.15062745	Gstm7	1
GO:1902936	phosphatidylinositol bisphosphate binding	1/29	97/14323	0.01030928	5.09171703	1.8212686	0.17902202	0.25723552	0.16648956	Fzd7	1
GO:0016798	hydrolase activity, acting on glycosyl bonds	1/29	100/14323	0.01	4.93896552	1.78037103	0.18403288	0.26159285	0.16930974	Kl	1
GO:0005267	potassium channel activity	1/29	101/14323	0.00990099	4.89006487	1.76710005	0.1856966	0.26159285	0.16930974	Kcnk2	1
GO:0005125	cytokine activity	1/29	102/14323	0.00980392	4.84212306	1.7540026	0.18735704	0.26159285	0.16930974	Bmp6	1
GO:0140678	molecular function inhibitor activity	2/29	399/14323	0.00501253	2.47567194	1.34653673	0.19294078	0.26559935	0.17190285	Gbp4/Kcnk2	2
GO:0015293	symporter activity	1/29	106/14323	0.00943396	4.65940143	1.70326785	0.19396618	0.26559935	0.17190285	Slc2a10	1
GO:0070851	growth factor receptor binding	1/29	107/14323	0.00934579	4.61585562	1.69097897	0.19561034	0.26559935	0.17190285	Kl	1
GO:0030165	PDZ domain binding	1/29	109/14323	0.00917431	4.53116102	1.66685016	0.19888894	0.26736453	0.17304532	Fzd7	1
GO:0019842	vitamin binding	1/29	110/14323	0.00909091	4.48996865	1.65500357	0.2005234	0.26736453	0.17304532	Comp	1
GO:0022843	voltage-gated monoatomic cation channel activity	1/29	117/14323	0.00854701	4.22133805	1.5758587	0.21187481	0.27997742	0.18120872	Kcnk2	1
GO:0022834	ligand-gated channel activity	1/29	121/14323	0.00826446	4.08178968	1.53336262	0.21829129	0.28590364	0.18504432	Kcnk2	1
GO:0002020	protease binding	1/29	130/14323	0.00769231	3.79920424	1.44408617	0.23254449	0.30129274	0.19500455	Comp	1
GO:0015079	potassium ion transmembrane transporter activity	1/29	131/14323	0.00763359	3.77020268	1.43466115	0.23411261	0.30129274	0.19500455	Kcnk2	1
GO:0008237	metallopeptidase activity	1/29	136/14323	0.00735294	3.63159229	1.38888638	0.24190687	0.30532587	0.1976149	Cpq	1
GO:0005509	calcium ion binding	2/29	466/14323	0.00429185	2.11972769	1.10686515	0.24305842	0.30532587	0.1976149	Fbn1/Comp	2
GO:0099106	ion channel regulator activity	1/29	138/14323	0.00724638	3.57896052	1.37117697	0.24500309	0.30532587	0.1976149	Kcnk2	1
GO:0005244	voltage-gated monoatomic ion channel activity	1/29	139/14323	0.00719424	3.5532126	1.36244495	0.24654662	0.30532587	0.1976149	Kcnk2	1
GO:0022832	voltage-gated channel activity	1/29	140/14323	0.00714286	3.52783251	1.35379282	0.24808709	0.30532587	0.1976149	Kcnk2	1
GO:0031406	carboxylic acid binding	1/29	141/14323	0.0070922	3.50281242	1.34521922	0.24962453	0.30532587	0.1976149	Gstm7	1
GO:0031072	heat shock protein binding	1/29	144/14323	0.00694444	3.42983716	1.31995632	0.25421865	0.3083964	0.19960222	Telo2	1
GO:0016247	channel regulator activity	1/29	148/14323	0.00675676	3.33713886	1.28729412	0.26030192	0.31177747	0.20179054	Kcnk2	1
GO:0043177	organic acid binding	1/29	149/14323	0.00671141	3.31474196	1.27930309	0.26181524	0.31177747	0.20179054	Gstm7	1
GO:0141108	transporter regulator activity	1/29	150/14323	0.00666667	3.29264368	1.27137961	0.26332557	0.31177747	0.20179054	Kcnk2	1
GO:0003735	structural constituent of ribosome	1/29	164/14323	0.00609756	3.01156434	1.16698025	0.2841594	0.33377454	0.21602762	Rpl8	1
GO:1901981	phosphatidylinositol phosphate binding	1/29	170/14323	0.00588235	2.90527383	1.12559344	0.29291299	0.34134742	0.22092898	Fzd7	1
GO:0005126	cytokine receptor binding	1/29	172/14323	0.00581395	2.87149158	1.11219895	0.29580781	0.34202778	0.22136933	Cx3cr1	1
GO:0033218	amide binding	1/29	193/14323	0.00518135	2.55904949	0.98218075	0.32552081	0.37346574	0.2417168	Gstm7	1
GO:0008514	organic anion transmembrane transporter activity	1/29	204/14323	0.00490196	2.42106153	0.92076741	0.34059789	0.38479761	0.24905109	Slc2a10	1
GO:0030246	carbohydrate binding	1/29	204/14323	0.00490196	2.42106153	0.92076741	0.34059789	0.38479761	0.24905109	Asgr1	1
GO:0015291	secondary active transmembrane transporter activity	1/29	218/14323	0.00458716	2.26558051	0.84811633	0.3593168	0.40287035	0.26074823	Slc2a10	1
GO:0022836	gated channel activity	1/29	231/14323	0.004329	2.13808031	0.78544635	0.3762385	0.41867141	0.2709751	Kcnk2	1
GO:0008017	microtubule binding	1/29	248/14323	0.00403226	1.99151835	0.70945743	0.39771682	0.43926932	0.2843066	Kifc3	1
GO:0005261	monoatomic cation channel activity	1/29	257/14323	0.00389105	1.92177647	0.67163113	0.40879679	0.4481624	0.29006244	Kcnk2	1
GO:0035091	phosphatidylinositol binding	1/29	265/14323	0.00377358	1.86376057	0.63926141	0.41848022	0.45540494	0.29475	Fzd7	1
GO:0004857	enzyme inhibitor activity	1/29	285/14323	0.00350877	1.73297036	0.56296341	0.44202354	0.47751449	0.30905987	Gbp4	1
GO:0005216	monoatomic ion channel activity	1/29	325/14323	0.00307692	1.5196817	0.42684486	0.48638025	0.5216252	0.33760948	Kcnk2	1
GO:0022804	active transmembrane transporter activity	1/29	336/14323	0.00297619	1.46993021	0.39261299	0.49796872	0.53021129	0.34316663	Slc2a10	1
GO:0046873	metal ion transmembrane transporter activity	1/29	346/14323	0.00289017	1.42744668	0.36252417	0.50828431	0.53732913	0.34777347	Kcnk2	1
GO:0015631	tubulin binding	1/29	353/14323	0.00283286	1.39914037	0.34200996	0.51538313	0.54096953	0.35012964	Kifc3	1
GO:0015267	channel activity	1/29	362/14323	0.00276243	1.36435512	0.31626042	0.52436491	0.54269935	0.35124922	Kcnk2	1
GO:0022803	passive transmembrane transporter activity	1/29	362/14323	0.00276243	1.36435512	0.31626042	0.52436491	0.54269935	0.35124922	Kcnk2	1
GO:0008233	peptidase activity	1/29	416/14323	0.00240385	1.18725133	0.17457369	0.57497382	0.59094532	0.38247528	Cpq	1
GO:0005543	phospholipid binding	1/29	428/14323	0.00233645	1.15396391	0.14565838	0.58549239	0.59760602	0.38678626	Fzd7	1
GO:0022890	inorganic cation transmembrane transporter activity	1/29	446/14323	0.00224215	1.10739137	0.10377983	0.60080039	0.60903053	0.3941805	Kcnk2	1
GO:0008324	monoatomic cation transmembrane transporter activity	1/29	477/14323	0.00209644	1.03542254	0.03544057	0.62589368	0.63015146	0.40785052	Kcnk2	1
GO:0140657	ATP-dependent activity	1/29	491/14323	0.00203666	1.00589929	0.00599131	0.63672034	0.63672034	0.41210207	Kifc3	1

ID	Description	GeneRatio	BgRatio	RichFactor	FoldEnrichm	zScore	pvalue	p.adjust	qvalue	geneID	Count
GO:0004364	glutathione transferase activity	2/69	24/14323	0.08333333	17.2983092	5.55972541	0.00589508	0.35746543	0.34623427	Gstt2/Gstt3	2
GO:0046875	ephrin receptor binding	2/69	31/14323	0.06451613	13.3922394	4.80553937	0.00971893	0.35746543	0.34623427	Ngef/Tiam1	2
GO:0001965	G-protein alpha-subunit binding	2/69	33/14323	0.06060606	12.5805885	4.63371681	0.01096765	0.35746543	0.34623427	Ccdc88c/Gpsm1	2
GO:0016798	hydrolase activity, acting on glycosyl bonds	3/69	100/14323	0.03	6.2273913	3.64961799	0.01240349	0.35746543	0.34623427	Hexd/Il1rapl1/Man2b1	3
GO:0042162	telomeric DNA binding	2/69	37/14323	0.05405405	11.2205249	4.33088589	0.01366432	0.35746543	0.34623427	Hnrnpd/Rpa2	2
GO:0070888	E-box binding	2/69	37/14323	0.05405405	11.2205249	4.33088589	0.01366432	0.35746543	0.34623427	Nr1d1/Bhlhe22	2
GO:0005085	guanyl-nucleotide exchange factor activity	4/69	197/14323	0.02030457	4.21481645	3.16109608	0.01497882	0.35746543	0.34623427	Ccdc88c/Ngef/Dennd6b/Tiam1	4
GO:0098631	cell adhesion mediator activity	2/69	41/14323	0.04878049	10.1258395	4.07125443	0.01661765	0.35746543	0.34623427	Ager/Cd200	2
GO:0001227	DNA-binding transcription repressor activity, RNA polymerase II-specific	4/69	224/14323	0.01785714	3.70678054	2.84080159	0.02283132	0.35746543	0.34623427	Nr1d1/Nfatc4/Nfe2l3/Nfx1	4
GO:0001217	DNA-binding transcription repressor activity	4/69	230/14323	0.0173913	3.61008192	2.77635075	0.02486072	0.35746543	0.34623427	Nr1d1/Nfatc4/Nfe2l3/Nfx1	4
GO:0001222	transcription corepressor binding	2/69	56/14323	0.03571429	7.41356108	3.34568401	0.02980239	0.35746543	0.34623427	Nr1d1/Ehmt2	2
GO:0016765	transferase activity, transferring alkyl or aryl (other than methyl) groups	2/69	56/14323	0.03571429	7.41356108	3.34568401	0.02980239	0.35746543	0.34623427	Gstt2/Gstt3	2
GO:0004553	hydrolase activity, hydrolyzing O-glycosyl compounds	2/69	67/14323	0.02985075	6.19640926	2.96619644	0.04137168	0.35746543	0.34623427	Hexd/Man2b1	2
GO:0002039	p53 binding	2/69	70/14323	0.02857143	5.93084886	2.87723657	0.04477706	0.35746543	0.34623427	Ehmt2/Dazap2	2
GO:0004527	exonuclease activity	2/69	72/14323	0.02777778	5.76610306	2.82075268	0.04710328	0.35746543	0.34623427	Enpp1/Xrn2	2
GO:0030971	receptor tyrosine kinase binding	2/69	72/14323	0.02777778	5.76610306	2.82075268	0.04710328	0.35746543	0.34623427	Tiam1/Dazap2	2
GO:0005005	transmembrane-ephrin receptor activity	1/69	10/14323	0.1	20.757971	4.34845531	0.04715772	0.35746543	0.34623427	Epha8	1
GO:0008503	benzodiazepine receptor activity	1/69	10/14323	0.1	20.757971	4.34845531	0.04715772	0.35746543	0.34623427	Gabrg1	1
GO:0015347	sodium-independent organic anion transmembrane transporter activity	1/69	10/14323	0.1	20.757971	4.34845531	0.04715772	0.35746543	0.34623427	Slco1a4	1
GO:0030695	GTPase regulator activity	5/69	414/14323	0.01207729	2.50700133	2.16483029	0.04908528	0.35746543	0.34623427	Ccdc88c/Ngef/Dennd6b/Tiam1/Gpsm1	5
GO:0060589	nucleoside-triphosphatase regulator activity	5/69	414/14323	0.01207729	2.50700133	2.16483029	0.04908528	0.35746543	0.34623427	Ccdc88c/Ngef/Dennd6b/Tiam1/Gpsm1	5
GO:0016783	sulfurtransferase activity	1/69	11/14323	0.09090909	18.8708827	4.12524918	0.05175118	0.35746543	0.34623427	Tst	1
GO:0034511	U3 snoRNA binding	1/69	11/14323	0.09090909	18.8708827	4.12524918	0.05175118	0.35746543	0.34623427	Rrp9	1
GO:0050815	phosphoserine residue binding	1/69	11/14323	0.09090909	18.8708827	4.12524918	0.05175118	0.35746543	0.34623427	Pcif1	1
GO:0061809	NAD+ nucleotidase, cyclic ADP-ribose generating	1/69	11/14323	0.09090909	18.8708827	4.12524918	0.05175118	0.35746543	0.34623427	Il1rapl1	1
GO:0070003	threonine-type peptidase activity	1/69	11/14323	0.09090909	18.8708827	4.12524918	0.05175118	0.35746543	0.34623427	Psmb9	1
GO:0098505	G-rich strand telomeric DNA binding	1/69	11/14323	0.09090909	18.8708827	4.12524918	0.05175118	0.35746543	0.34623427	Rpa2	1
GO:0099122	RNA polymerase II C-terminal domain binding	1/69	11/14323	0.09090909	18.8708827	4.12524918	0.05175118	0.35746543	0.34623427	Pcif1	1
GO:0004890	GABA-A receptor activity	1/69	12/14323	0.08333333	17.2983092	3.92967096	0.05632281	0.35746543	0.34623427	Gabrg1	1
GO:0005243	gap junction channel activity	1/69	12/14323	0.08333333	17.2983092	3.92967096	0.05632281	0.35746543	0.34623427	Gja4	1
GO:0015125	bile acid transmembrane transporter activity	1/69	12/14323	0.08333333	17.2983092	3.92967096	0.05632281	0.35746543	0.34623427	Slco1a4	1
GO:0022851	GABA-gated chloride ion channel activity	1/69	12/14323	0.08333333	17.2983092	3.92967096	0.05632281	0.35746543	0.34623427	Gabrg1	1
GO:0008174	mRNA methyltransferase activity	1/69	13/14323	0.07692308	15.96767	3.75633279	0.06087272	0.35746543	0.34623427	Pcif1	1
GO:0019855	calcium channel inhibitor activity	1/69	13/14323	0.07692308	15.96767	3.75633279	0.06087272	0.35746543	0.34623427	Slc30a1	1
GO:0030881	beta-2-microglobulin binding	1/69	13/14323	0.07692308	15.96767	3.75633279	0.06087272	0.35746543	0.34623427	H2-T23	1
GO:0043047	single-stranded telomeric DNA binding	1/69	13/14323	0.07692308	15.96767	3.75633279	0.06087272	0.35746543	0.34623427	Rpa2	1
GO:0046974	histone H3K9 methyltransferase activity	1/69	13/14323	0.07692308	15.96767	3.75633279	0.06087272	0.35746543	0.34623427	Ehmt2	1
GO:0004559	alpha-mannosidase activity	1/69	14/14323	0.07142857	14.8271222	3.60121606	0.065401	0.35746543	0.34623427	Man2b1	1
GO:0008097	5S rRNA binding	1/69	14/14323	0.07142857	14.8271222	3.60121606	0.065401	0.35746543	0.34623427	Tst	1
GO:0015929	hexosaminidase activity	1/69	14/14323	0.07142857	14.8271222	3.60121606	0.065401	0.35746543	0.34623427	Hexd	1
GO:0044548	S100 protein binding	1/69	14/14323	0.07142857	14.8271222	3.60121606	0.065401	0.35746543	0.34623427	Ager	1
GO:0046703	natural killer cell lectin-like receptor binding	1/69	14/14323	0.07142857	14.8271222	3.60121606	0.065401	0.35746543	0.34623427	H2-T23	1
GO:0070742	C2H2 zinc finger domain binding	1/69	14/14323	0.07142857	14.8271222	3.60121606	0.065401	0.35746543	0.34623427	Ehmt2	1
GO:0005003	ephrin receptor activity	1/69	15/14323	0.06666667	13.8386473	3.46125373	0.06990777	0.35746543	0.34623427	Epha8	1
GO:0015923	mannosidase activity	1/69	15/14323	0.06666667	13.8386473	3.46125373	0.06990777	0.35746543	0.34623427	Man2b1	1
GO:0016917	GABA receptor activity	1/69	15/14323	0.06666667	13.8386473	3.46125373	0.06990777	0.35746543	0.34623427	Gabrg1	1
GO:0034236	protein kinase A catalytic subunit binding	1/69	15/14323	0.06666667	13.8386473	3.46125373	0.06990777	0.35746543	0.34623427	Pkia	1
GO:0098847	sequence-specific single stranded DNA binding	1/69	15/14323	0.06666667	13.8386473	3.46125373	0.06990777	0.35746543	0.34623427	Rpa2	1
GO:0005092	GDP-dissociation inhibitor activity	1/69	16/14323	0.0625	12.9737319	3.33405865	0.07439312	0.35746543	0.34623427	Gpsm1	1
GO:0016646	oxidoreductase activity, acting on the CH-NH group of donors, NAD or NADP as acceptor	1/69	16/14323	0.0625	12.9737319	3.33405865	0.07439312	0.35746543	0.34623427	Pycr1	1
GO:0008409	5'-3' exonuclease activity	1/69	17/14323	0.05882353	12.2105712	3.21774107	0.07885715	0.35746543	0.34623427	Xrn2	1
GO:0043422	protein kinase B binding	1/69	17/14323	0.05882353	12.2105712	3.21774107	0.07885715	0.35746543	0.34623427	Srsf5	1
GO:0061133	endopeptidase activator activity	1/69	17/14323	0.05882353	12.2105712	3.21774107	0.07885715	0.35746543	0.34623427	Aim2	1
GO:0050811	GABA receptor binding	1/69	18/14323	0.05555556	11.5322061	3.11078269	0.08329996	0.35746543	0.34623427	Gabrg1	1
GO:0004115	3',5'-cyclic-AMP phosphodiesterase activity	1/69	19/14323	0.05263158	10.9252479	3.01194762	0.08772165	0.35746543	0.34623427	Enpp1	1
GO:0004602	glutathione peroxidase activity	1/69	19/14323	0.05263158	10.9252479	3.01194762	0.08772165	0.35746543	0.34623427	Gstt2	1
GO:0005537	D-mannose binding	1/69	19/14323	0.05263158	10.9252479	3.01194762	0.08772165	0.35746543	0.34623427	Man2b1	1
GO:0042608	T cell receptor binding	1/69	19/14323	0.05263158	10.9252479	3.01194762	0.08772165	0.35746543	0.34623427	H2-T23	1
GO:0050699	WW domain binding	1/69	19/14323	0.05263158	10.9252479	3.01194762	0.08772165	0.35746543	0.34623427	Dazap2	1
GO:0051959	dynein light intermediate chain binding	1/69	19/14323	0.05263158	10.9252479	3.01194762	0.08772165	0.35746543	0.34623427	Ccdc88c	1
GO:0004114	3',5'-cyclic-nucleotide phosphodiesterase activity	1/69	20/14323	0.05	10.3789855	2.92021815	0.09212232	0.35746543	0.34623427	Enpp1	1
GO:0030247	polysaccharide binding	1/69	20/14323	0.05	10.3789855	2.92021815	0.09212232	0.35746543	0.34623427	Enpp1	1
GO:0042975	peroxisome proliferator activated receptor binding	1/69	20/14323	0.05	10.3789855	2.92021815	0.09212232	0.35746543	0.34623427	Nfatc4	1
GO:0051139	metal cation:proton antiporter activity	1/69	20/14323	0.05	10.3789855	2.92021815	0.09212232	0.35746543	0.34623427	Slc30a1	1
GO:0070628	proteasome binding	1/69	20/14323	0.05	10.3789855	2.92021815	0.09212232	0.35746543	0.34623427	Psmb9	1
GO:0140517	protein-RNA adaptor activity	1/69	20/14323	0.05	10.3789855	2.92021815	0.09212232	0.35746543	0.34623427	Zfp598	1
GO:1904047	S-adenosyl-L-methionine binding	1/69	20/14323	0.05	10.3789855	2.92021815	0.09212232	0.35746543	0.34623427	Pcif1	1
GO:1990782	protein tyrosine kinase binding	2/69	108/14323	0.01851852	3.84406871	2.06412756	0.09548603	0.35746543	0.34623427	Tiam1/Dazap2	2
GO:0005044	scavenger receptor activity	1/69	21/14323	0.04761905	9.8847481	2.83474751	0.09650207	0.35746543	0.34623427	Enpp1	1
GO:0005164	tumor necrosis factor receptor binding	1/69	21/14323	0.04761905	9.8847481	2.83474751	0.09650207	0.35746543	0.34623427	Tnfsf10	1
GO:0022829	wide pore channel activity	1/69	21/14323	0.04761905	9.8847481	2.83474751	0.09650207	0.35746543	0.34623427	Gja4	1
GO:0004112	cyclic-nucleotide phosphodiesterase activity	1/69	22/14323	0.04545455	9.43544137	2.75482459	0.100861	0.35846544	0.34720287	Enpp1	1
GO:0005385	zinc ion transmembrane transporter activity	1/69	22/14323	0.04545455	9.43544137	2.75482459	0.100861	0.35846544	0.34720287	Slc30a1	1
GO:0008143	poly(A) binding	1/69	22/14323	0.04545455	9.43544137	2.75482459	0.100861	0.35846544	0.34720287	Hnrnpdl	1
GO:0005158	insulin receptor binding	1/69	23/14323	0.04347826	9.02520479	2.67984716	0.1051992	0.36003638	0.34872445	Enpp1	1
GO:0120283	protein serine/threonine kinase binding	1/69	23/14323	0.04347826	9.02520479	2.67984716	0.1051992	0.36003638	0.34872445	Dazap2	1
GO:0016645	oxidoreductase activity, acting on the CH-NH group of donors	1/69	24/14323	0.04166667	8.64915459	2.60930129	0.10951677	0.36003638	0.34872445	Pycr1	1
GO:0042288	MHC class I protein binding	1/69	24/14323	0.04166667	8.64915459	2.60930129	0.10951677	0.36003638	0.34872445	H2-T23	1
GO:0042605	peptide antigen binding	1/69	24/14323	0.04166667	8.64915459	2.60930129	0.10951677	0.36003638	0.34872445	H2-T23	1
GO:0070412	R-SMAD binding	1/69	24/14323	0.04166667	8.64915459	2.60930129	0.10951677	0.36003638	0.34872445	Ldlrad4	1
GO:0003697	single-stranded DNA binding	2/69	119/14323	0.01680672	3.48873462	1.89672663	0.11226127	0.36450263	0.35305038	Hnrnpdl/Rpa2	2
GO:0031435	mitogen-activated protein kinase kinase kinase binding	1/69	26/14323	0.03846154	7.98383501	2.47979733	0.11809041	0.37561894	0.36381743	Dazap2	1
GO:0001221	transcription coregulator binding	2/69	123/14323	0.01626016	3.37527984	1.84069219	0.11854134	0.37561894	0.36381743	Nr1d1/Ehmt2	2
GO:0099095	ligand-gated monoatomic anion channel activity	1/69	27/14323	0.03703704	7.68813741	2.42012503	0.12234667	0.38306159	0.37102624	Gabrg1	1
GO:0000175	3'-5'-RNA exonuclease activity	1/69	29/14323	0.03448276	7.15792104	2.30948012	0.13079855	0.38651706	0.37437314	Xrn2	1
GO:0004693	cyclin-dependent protein serine/threonine kinase activity	1/69	29/14323	0.03448276	7.15792104	2.30948012	0.13079855	0.38651706	0.37437314	Cdk2	1
GO:0070717	poly-purine tract binding	1/69	29/14323	0.03448276	7.15792104	2.30948012	0.13079855	0.38651706	0.37437314	Hnrnpdl	1
GO:0097472	cyclin-dependent protein kinase activity	1/69	29/14323	0.03448276	7.15792104	2.30948012	0.13079855	0.38651706	0.37437314	Cdk2	1
GO:0098632	cell-cell adhesion mediator activity	1/69	29/14323	0.03448276	7.15792104	2.30948012	0.13079855	0.38651706	0.37437314	Cd200	1
GO:0035925	mRNA 3'-UTR AU-rich region binding	1/69	30/14323	0.03333333	6.91932367	2.25802643	0.13499436	0.39174352	0.37943539	Hnrnpd	1
GO:0005109	frizzled binding	1/69	31/14323	0.03225806	6.69611968	2.20887659	0.13917021	0.39174352	0.37943539	Ccdc88c	1
GO:0016896	RNA exonuclease activity, producing 5'-phosphomonoesters	1/69	32/14323	0.03125	6.48686594	2.16185236	0.14332619	0.39174352	0.37943539	Xrn2	1
GO:0042287	MHC protein binding	1/69	32/14323	0.03125	6.48686594	2.16185236	0.14332619	0.39174352	0.37943539	H2-T23	1
GO:0000217	DNA secondary structure binding	1/69	33/14323	0.03030303	6.29029425	2.11679444	0.14746239	0.39174352	0.37943539	Hnrnpd	1
GO:0004532	RNA exonuclease activity	1/69	33/14323	0.03030303	6.29029425	2.11679444	0.14746239	0.39174352	0.37943539	Xrn2	1
GO:0005484	SNAP receptor activity	1/69	33/14323	0.03030303	6.29029425	2.11679444	0.14746239	0.39174352	0.37943539	Stx17	1
GO:0016799	hydrolase activity, hydrolyzing N-glycosyl compounds	1/69	33/14323	0.03030303	6.29029425	2.11679444	0.14746239	0.39174352	0.37943539	Il1rapl1	1
GO:0030515	snoRNA binding	1/69	33/14323	0.03030303	6.29029425	2.11679444	0.14746239	0.39174352	0.37943539	Rrp9	1
GO:0140828	metal cation:monoatomic cation antiporter activity	1/69	33/14323	0.03030303	6.29029425	2.11679444	0.14746239	0.39174352	0.37943539	Slc30a1	1
GO:1901618	organic hydroxy compound transmembrane transporter activity	1/69	34/14323	0.02941176	6.10528559	2.0735599	0.15157891	0.39769868	0.38520345	Slco1a4	1
GO:0030332	cyclin binding	1/69	35/14323	0.02857143	5.93084886	2.03202008	0.15567584	0.39769868	0.38520345	Cdk2	1
GO:0032813	tumor necrosis factor receptor superfamily binding	1/69	35/14323	0.02857143	5.93084886	2.03202008	0.15567584	0.39769868	0.38520345	Tnfsf10	1
GO:0038187	pattern recognition receptor activity	1/69	36/14323	0.02777778	5.76610306	1.99205883	0.15975328	0.39769868	0.38520345	Aim2	1
GO:0008757	S-adenosylmethionine-dependent methyltransferase activity	2/69	149/14323	0.01342282	2.78630483	1.52496718	0.16124246	0.39769868	0.38520345	Pcif1/Ehmt2	2
GO:0046915	transition metal ion transmembrane transporter activity	1/69	37/14323	0.02702703	5.61026244	1.95357096	0.1638113	0.39769868	0.38520345	Slc30a1	1
GO:0000993	RNA polymerase II complex binding	1/69	38/14323	0.02631579	5.46262395	1.91646093	0.16785001	0.39769868	0.38520345	Pcif1	1
GO:0022824	transmitter-gated monoatomic ion channel activity	1/69	38/14323	0.02631579	5.46262395	1.91646093	0.16785001	0.39769868	0.38520345	Gabrg1	1
GO:0022835	transmitter-gated channel activity	1/69	38/14323	0.02631579	5.46262395	1.91646093	0.16785001	0.39769868	0.38520345	Gabrg1	1
GO:0030291	protein serine/threonine kinase inhibitor activity	1/69	38/14323	0.02631579	5.46262395	1.91646093	0.16785001	0.39769868	0.38520345	Pkia	1
GO:0036002	pre-mRNA binding	1/69	38/14323	0.02631579	5.46262395	1.91646093	0.16785001	0.39769868	0.38520345	Arglu1	1
GO:0170055	lipid transmembrane transporter activity	1/69	38/14323	0.02631579	5.46262395	1.91646093	0.16785001	0.39769868	0.38520345	Slco1a4	1
GO:0004601	peroxidase activity	1/69	41/14323	0.02439024	5.06291976	1.8125653	0.17985115	0.41131177	0.39838883	Gstt2	1
GO:0005246	calcium channel regulator activity	1/69	41/14323	0.02439024	5.06291976	1.8125653	0.17985115	0.41131177	0.39838883	Slc30a1	1
GO:0008200	ion channel inhibitor activity	1/69	41/14323	0.02439024	5.06291976	1.8125653	0.17985115	0.41131177	0.39838883	Slc30a1	1
GO:0140938	histone H3 methyltransferase activity	1/69	41/14323	0.02439024	5.06291976	1.8125653	0.17985115	0.41131177	0.39838883	Ehmt2	1
GO:0016248	channel inhibitor activity	1/69	42/14323	0.02380952	4.94237405	1.78016872	0.1838135	0.41318762	0.40020574	Slc30a1	1
GO:0141110	transporter inhibitor activity	1/69	42/14323	0.02380952	4.94237405	1.78016872	0.1838135	0.41318762	0.40020574	Slc30a1	1
GO:0016684	oxidoreductase activity, acting on peroxide as acceptor	1/69	43/14323	0.02325581	4.82743512	1.74878317	0.18775699	0.41495872	0.40192119	Gstt2	1
GO:0043175	RNA polymerase core enzyme binding	1/69	43/14323	0.02325581	4.82743512	1.74878317	0.18775699	0.41495872	0.40192119	Pcif1	1
GO:0004879	nuclear receptor activity	1/69	44/14323	0.02272727	4.71772069	1.71835227	0.19168169	0.41663045	0.4035404	Nr1d1	1
GO:0005547	phosphatidylinositol-3,4,5-trisphosphate binding	1/69	44/14323	0.02272727	4.71772069	1.71835227	0.19168169	0.41663045	0.4035404	Fundc2	1
GO:0098531	ligand-activated transcription factor activity	1/69	45/14323	0.02222222	4.61288245	1.68882404	0.1955877	0.41969564	0.40650928	Nr1d1	1
GO:0019903	protein phosphatase binding	2/69	171/14323	0.01169591	2.42783287	1.30684633	0.19922513	0.41969564	0.40650928	Stx17/Rpa2	2
GO:0005230	extracellular ligand-gated monoatomic ion channel activity	1/69	46/14323	0.02173913	4.51260239	1.66015039	0.19947511	0.41969564	0.40650928	Gabrg1	1
GO:0060590	ATPase regulator activity	1/69	46/14323	0.02173913	4.51260239	1.66015039	0.19947511	0.41969564	0.40650928	Fnip1	1
GO:0008408	3'-5' exonuclease activity	1/69	47/14323	0.0212766	4.41658958	1.63228682	0.20334401	0.42444027	0.41110485	Xrn2	1
GO:0004518	nuclease activity	2/69	175/14323	0.01142857	2.37233954	1.2708406	0.20625236	0.42712103	0.41370138	Enpp1/Xrn2	2
GO:0003823	antigen binding	1/69	49/14323	0.02040816	4.23632062	1.5788277	0.2110266	0.43132094	0.41776933	H2-T23	1
GO:0008168	methyltransferase activity	2/69	179/14323	0.01117318	2.31932637	1.23580696	0.21330731	0.43132094	0.41776933	Pcif1/Ehmt2	2
GO:0004714	transmembrane receptor protein tyrosine kinase activity	1/69	50/14323	0.02	4.1515942	1.5531581	0.21484047	0.43132094	0.41776933	Epha8	1
GO:0016796	exonuclease activity, active with either ribo- or deoxyribonucleic acids and producing 5'-phosphomonoest	1/69	50/14323	0.02	4.1515942	1.5531581	0.21484047	0.43132094	0.41776933	Xrn2	1
GO:0005254	chloride channel activity	1/69	51/14323	0.01960784	4.07019039	1.52814998	0.21863617	0.43234069	0.41875705	Gabrg1	1
GO:0016504	peptidase activator activity	1/69	51/14323	0.01960784	4.07019039	1.52814998	0.21863617	0.43234069	0.41875705	Aim2	1
GO:0030374	nuclear receptor coactivator activity	1/69	53/14323	0.01886792	3.9165983	1.4799961	0.2261734	0.43958983	0.42577842	Arglu1	1
GO:0051018	protein kinase A binding	1/69	53/14323	0.01886792	3.9165983	1.4799961	0.2261734	0.43958983	0.42577842	Pkia	1
GO:0016741	transferase activity, transferring one-carbon groups	2/69	188/14323	0.0106383	2.20829479	1.16027764	0.22926403	0.43958983	0.42577842	Pcif1/Ehmt2	2
GO:0038024	cargo receptor activity	1/69	54/14323	0.01851852	3.84406871	1.45679418	0.22991509	0.43958983	0.42577842	Enpp1	1
GO:0016782	transferase activity, transferring sulphur-containing groups	1/69	55/14323	0.01818182	3.77417655	1.43414114	0.23363896	0.43958983	0.42577842	Tst	1
GO:0140299	molecular sensor activity	1/69	55/14323	0.01818182	3.77417655	1.43414114	0.23363896	0.43958983	0.42577842	Zfp598	1
GO:0008028	monocarboxylic acid transmembrane transporter activity	1/69	56/14323	0.01785714	3.70678054	1.41201312	0.23734508	0.43958983	0.42577842	Slco1a4	1
GO:0016278	lysine N-methyltransferase activity	1/69	56/14323	0.01785714	3.70678054	1.41201312	0.23734508	0.43958983	0.42577842	Ehmt2	1
GO:0016279	protein-lysine N-methyltransferase activity	1/69	56/14323	0.01785714	3.70678054	1.41201312	0.23734508	0.43958983	0.42577842	Ehmt2	1
GO:0045309	protein phosphorylated amino acid binding	1/69	59/14323	0.01694915	3.51830017	1.34856175	0.24835778	0.44906414	0.43495506	Pcif1	1
GO:0042277	peptide binding	2/69	200/14323	0.01	2.0757971	1.0659575	0.25066425	0.44906414	0.43495506	H2-T23/Ager	2
GO:0008173	RNA methyltransferase activity	1/69	61/14323	0.01639344	3.40294607	1.30850904	0.25561237	0.44906414	0.43495506	Pcif1	1
GO:0030246	carbohydrate binding	2/69	204/14323	0.00980392	2.0350952	1.03598009	0.25781637	0.44906414	0.43495506	Enpp1/Man2b1	2
GO:0001540	amyloid-beta binding	1/69	62/14323	0.01612903	3.34805984	1.28910419	0.25921374	0.44906414	0.43495506	Ager	1
GO:0070063	RNA polymerase binding	1/69	62/14323	0.01612903	3.34805984	1.28910419	0.25921374	0.44906414	0.43495506	Pcif1	1
GO:0001098	basal transcription machinery binding	1/69	63/14323	0.01587302	3.29491603	1.27009239	0.26279794	0.44906414	0.43495506	Pcif1	1
GO:0001099	basal RNA polymerase II transcription machinery binding	1/69	63/14323	0.01587302	3.29491603	1.27009239	0.26279794	0.44906414	0.43495506	Pcif1	1
GO:0004497	monooxygenase activity	1/69	63/14323	0.01587302	3.29491603	1.27009239	0.26279794	0.44906414	0.43495506	Foxred2	1
GO:0016209	antioxidant activity	1/69	63/14323	0.01587302	3.29491603	1.27009239	0.26279794	0.44906414	0.43495506	Gstt2	1
GO:0030594	neurotransmitter receptor activity	1/69	63/14323	0.01587302	3.29491603	1.27009239	0.26279794	0.44906414	0.43495506	Gabrg1	1
GO:0005253	monoatomic anion channel activity	1/69	64/14323	0.015625	3.24343297	1.25145879	0.26636504	0.44906414	0.43495506	Gabrg1	1
GO:0005518	collagen binding	1/69	64/14323	0.015625	3.24343297	1.25145879	0.26636504	0.44906414	0.43495506	Abi3bp	1
GO:0043539	protein serine/threonine kinase activator activity	1/69	64/14323	0.015625	3.24343297	1.25145879	0.26636504	0.44906414	0.43495506	Dazap2	1
GO:0019887	protein kinase regulator activity	2/69	210/14323	0.00952381	1.97694962	0.99227066	0.26855214	0.44928912	0.43517297	Pkia/Dazap2	2
GO:0042054	histone methyltransferase activity	1/69	65/14323	0.01538462	3.193534	1.2331893	0.26991514	0.44928912	0.43517297	Ehmt2	1
GO:0019199	transmembrane receptor protein kinase activity	1/69	66/14323	0.01515152	3.14514712	1.21527057	0.2734483	0.45230757	0.43809658	Epha8	1
GO:0015291	secondary active transmembrane transporter activity	2/69	218/14323	0.00917431	1.90440101	0.93618298	0.2828665	0.45814762	0.44375314	Slco1a4/Slc30a1	2
GO:0019902	phosphatase binding	2/69	218/14323	0.00917431	1.90440101	0.93618298	0.2828665	0.45814762	0.44375314	Stx17/Rpa2	2
GO:0003725	double-stranded RNA binding	1/69	69/14323	0.01449275	3.0084016	1.16349556	0.283947	0.45814762	0.44375314	Rftn1	1
GO:0048029	monosaccharide binding	1/69	69/14323	0.01449275	3.0084016	1.16349556	0.283947	0.45814762	0.44375314	Man2b1	1
GO:0004860	protein kinase inhibitor activity	1/69	71/14323	0.01408451	2.92365789	1.13051703	0.29086293	0.46361788	0.44905153	Pkia	1
GO:1990841	promoter-specific chromatin binding	1/69	71/14323	0.01408451	2.92365789	1.13051703	0.29086293	0.46361788	0.44905153	Ehmt2	1

GO:0019843	rRNA binding	1/69	72/14323	0.01388889	2.88305153	1.1144582	0.29429616	0.4651745	0.45055925	Tst	1
GO:1901681	sulfur compound binding	2/69	225/14323	0.00888889	1.84515298	0.88900834	0.29537696	0.4651745	0.45055925	Pcif1/Abi3bp	2
GO:0003684	damaged DNA binding	1/69	74/14323	0.01351351	2.80513122	1.0831546	0.30111356	0.46701844	0.45234525	Rpa2	1
GO:0019210	kinase inhibitor activity	1/69	74/14323	0.01351351	2.80513122	1.0831546	0.30111356	0.46701844	0.45234525	Pkia	1
GO:0140678	molecular function inhibitor activity	3/69	399/14323	0.0075188	1.5607497	0.7903719	0.30187504	0.46701844	0.45234525	Pkia/Slc30a1/Fnip1	3
GO:0008081	phosphoric diester hydrolase activity	1/69	76/14323	0.01315789	2.73131198	1.05287889	0.30786605	0.47350158	0.4586247	Enpp1	1
GO:0046332	SMAD binding	1/69	77/14323	0.01298701	2.69584039	1.0381064	0.31121814	0.47587425	0.46092282	Ldlrad4	1
GO:0015108	chloride transmembrane transporter activity	1/69	80/14323	0.0125	2.59474638	0.99516223	0.32117871	0.48545977	0.47020719	Gabrg1	1
GO:0050660	flavin adenine dinucleotide binding	1/69	80/14323	0.0125	2.59474638	0.99516223	0.32117871	0.48545977	0.47020719	Foxred2	1
GO:0019207	kinase regulator activity	2/69	242/14323	0.00826446	1.71553479	0.78105154	0.32562368	0.48936587	0.47399056	Pkia/Dazap2	2
GO:0003727	single-stranded RNA binding	1/69	83/14323	0.01204819	2.50096036	0.95413819	0.3309973	0.49461528	0.47907504	Hnrnpdl	1
GO:0020037	heme binding	1/69	84/14323	0.01190476	2.47118703	0.94086169	0.33423895	0.49663754	0.48103376	Nr1d1	1
GO:0008017	microtubule binding	2/69	248/14323	0.00806452	1.67402992	0.74497006	0.33623021	0.49678958	0.48118102	Ccdc88c/Tiam1	2
GO:0019888	protein phosphatase regulator activity	1/69	88/14323	0.01136364	2.35886034	0.88960134	0.3470515	0.50991365	0.49389275	Ppp2r3a	1
GO:0050839	cell adhesion molecule binding	2/69	257/14323	0.0077821	1.6154063	0.69262984	0.35205113	0.51063376	0.49459024	Ager/Cd200	2
GO:0008170	N-methyltransferase activity	1/69	90/14323	0.01111111	2.30644122	0.86500956	0.35336633	0.51063376	0.49459024	Ehmt2	1
GO:0008276	protein methyltransferase activity	1/69	90/14323	0.01111111	2.30644122	0.86500956	0.35336633	0.51063376	0.49459024	Ehmt2	1
GO:0046906	tetrapyrrole binding	1/69	91/14323	0.01098901	2.28109572	0.85295734	0.35650114	0.51234863	0.49625123	Nr1d1	1
GO:0019208	phosphatase regulator activity	1/69	96/14323	0.01041667	2.16228865	0.79497038	0.37195191	0.53164866	0.51494487	Ppp2r3a	1
GO:0004540	RNA nuclease activity	1/69	99/14323	0.01010101	2.09676475	0.76186533	0.38104628	0.5401328	0.52316245	Xrn2	1
GO:0008509	monoatomic anion transmembrane transporter activity	1/69	100/14323	0.01	2.0757971	0.75109136	0.3840488	0.5401328	0.52316245	Gabrg1	1
GO:0019955	cytokine binding	1/69	100/14323	0.01	2.0757971	0.75109136	0.3840488	0.5401328	0.52316245	Ager	1
GO:0003730	mRNA 3'-UTR binding	1/69	101/14323	0.00990099	2.05524465	0.7404428	0.38703696	0.54110596	0.52410503	Hnrnpd	1
GO:0005125	cytokine activity	1/69	102/14323	0.00980392	2.0350952	0.72991672	0.39001083	0.54110596	0.52410503	Tnfsf10	1
GO:0015103	inorganic anion transmembrane transporter activity	1/69	103/14323	0.00970874	2.01533699	0.71951032	0.39297049	0.54110596	0.52410503	Gabrg1	1
GO:0051219	phosphoprotein binding	1/69	103/14323	0.00970874	2.01533699	0.71951032	0.39297049	0.54110596	0.52410503	Pcif1	1
GO:0008013	beta-catenin binding	1/69	104/14323	0.00961538	1.99595875	0.70922085	0.39591599	0.54232242	0.52528328	Ajap1	1
GO:0015297	antiporter activity	1/69	107/14323	0.00934579	1.93999729	0.67902816	0.40466824	0.55073478	0.53343133	Slc30a1	1
GO:0035591	signaling adaptor activity	1/69	108/14323	0.00925926	1.92203435	0.66918092	0.4075578	0.55073478	0.53343133	Aim2	1
GO:0030165	PDZ domain binding	1/69	109/14323	0.00917431	1.90440101	0.65943825	0.41043353	0.55073478	0.53343133	Ccdc88c	1
GO:0061135	endopeptidase regulator activity	1/69	109/14323	0.00917431	1.90440101	0.65943825	0.41043353	0.55073478	0.53343133	Aim2	1
GO:0000149	SNARE binding	1/69	110/14323	0.00909091	1.88708827	0.6497979	0.4132955	0.55175998	0.53442432	Stx17	1
GO:0043022	ribosome binding	1/69	112/14323	0.00892857	1.85339027	0.63081554	0.41897843	0.55312732	0.5357487	Zfp598	1
GO:0031625	ubiquitin protein ligase binding	2/69	297/14323	0.00673401	1.39784317	0.48203793	0.42062914	0.55312732	0.5357487	Rpa2/Dazap2	2
GO:0061630	ubiquitin protein ligase activity	2/69	297/14323	0.00673401	1.39784317	0.48203793	0.42062914	0.55312732	0.5357487	Nfx1/Zfp598	2
GO:0004713	protein tyrosine kinase activity	1/69	115/14323	0.00869565	1.80504096	0.60305709	0.42740126	0.55923648	0.54166591	Epha8	1
GO:0015276	ligand-gated monoatomic ion channel activity	1/69	116/14323	0.00862069	1.78948026	0.59398719	0.43018204	0.5600885	0.54249116	Gabrg1	1
GO:0030295	protein kinase activator activity	1/69	117/14323	0.00854701	1.77418556	0.58500569	0.43294951	0.56091488	0.54329158	Dazap2	1
GO:0015078	proton transmembrane transporter activity	1/69	118/14323	0.00847458	1.75915009	0.57611082	0.43570373	0.56171609	0.54406761	Slc30a1	1
GO:0042826	histone deacetylase binding	1/69	119/14323	0.00840336	1.74436731	0.56730086	0.43844477	0.56249256	0.54481969	Hnrnpd	1
GO:0022834	ligand-gated channel activity	1/69	121/14323	0.00826446	1.71553479	0.54992909	0.44388754	0.56601903	0.54823536	Gabrg1	1
GO:0044389	ubiquitin-like protein ligase binding	2/69	313/14323	0.00638978	1.32638792	0.40620493	0.44706809	0.56601903	0.54823536	Rpa2/Dazap2	2
GO:0061659	ubiquitin-like protein ligase activity	2/69	314/14323	0.00636943	1.32216376	0.40160207	0.448699	0.56601903	0.54823536	Nfx1/Zfp598	2
GO:0003729	mRNA binding	2/69	316/14323	0.00632911	1.31379563	0.39244236	0.45195297	0.56601903	0.54823536	Hnrnpd/Luc7l2	2
GO:0019838	growth factor binding	1/69	124/14323	0.00806452	1.67402992	0.52446818	0.45195436	0.56601903	0.54823536	Epha8	1
GO:0005319	lipid transporter activity	1/69	125/14323	0.008	1.66063768	0.51613435	0.45461758	0.56665604	0.54885236	Slco1a4	1
GO:0019209	kinase activator activity	1/69	126/14323	0.00793651	1.64745802	0.50787472	0.45726805	0.56727122	0.54944821	Dazap2	1
GO:0008201	heparin binding	1/69	129/14323	0.00775194	1.60914504	0.48352749	0.46514354	0.57433216	0.5562873	Abi3bp	1
GO:0022804	active transmembrane transporter activity	2/69	336/14323	0.00595238	1.23559351	0.30403857	0.48389642	0.59430747	0.57563501	Slco1a4/Slc30a1	2
GO:0051087	protein-folding chaperone binding	1/69	138/14323	0.00724638	1.5042008	0.41408205	0.48810043	0.59430747	0.57563501	Fnip1	1
GO:0099106	ion channel regulator activity	1/69	138/14323	0.00724638	1.5042008	0.41408205	0.48810043	0.59430747	0.57563501	Slc30a1	1
GO:0140098	catalytic activity, acting on RNA	2/69	343/14323	0.0058309	1.21037732	0.2743787	0.49481075	0.59970151	0.58085958	Pcif1/Xrn2	2
GO:0046943	carboxylic acid transmembrane transporter activity	1/69	144/14323	0.00694444	1.44152576	0.3704866	0.50286267	0.60666459	0.58760389	Slco1a4	1
GO:0005342	organic acid transmembrane transporter activity	1/69	145/14323	0.00689655	1.43158421	0.36341267	0.50528191	0.60679974	0.58773479	Slco1a4	1
GO:0061134	peptidase regulator activity	1/69	146/14323	0.00684932	1.42177884	0.35639126	0.50768955	0.60691979	0.58785106	Aim2	1
GO:0015631	tubulin binding	2/69	353/14323	0.00566572	1.17608901	0.23306626	0.51015392	0.60710625	0.58803167	Ccdc88c/Tiam1	2
GO:0016247	channel regulator activity	1/69	148/14323	0.00675676	1.40256561	0.3425027	0.51247025	0.60711565	0.58804078	Slc30a1	1
GO:0141108	transporter regulator activity	1/69	150/14323	0.00666667	1.38386473	0.32881449	0.51720518	0.60997741	0.59081262	Slc30a1	1
GO:0015267	channel activity	2/69	362/14323	0.00552486	1.14684923	0.19689092	0.52370704	0.61215534	0.59292212	Gja4/Gabrg1	2
GO:0022803	passive transmembrane transporter activity	2/69	362/14323	0.00552486	1.14684923	0.19689092	0.52370704	0.61215534	0.59292212	Gja4/Gabrg1	2
GO:0004842	ubiquitin-protein transferase activity	2/69	376/14323	0.00531915	1.10414739	0.14238359	0.54429722	0.63340783	0.61350688	Nfx1/Zfp598	2
GO:1901981	phosphatidylinositol phosphate binding	1/69	170/14323	0.00588235	1.22105712	0.20172639	0.56213087	0.65127937	0.63081692	Fundc2	1
GO:0005126	cytokine receptor binding	1/69	172/14323	0.00581395	1.20685878	0.18989023	0.56639009	0.65333593	0.63280887	Tnfsf10	1
GO:0005539	glycosaminoglycan binding	1/69	178/14323	0.00561798	1.16617815	0.15521741	0.57892428	0.66269107	0.64187008	Abi3bp	1
GO:0019787	ubiquitin-like protein transferase activity	2/69	401/14323	0.00498753	1.03531028	0.04989769	0.57953972	0.66269107	0.64187008	Nfx1/Zfp598	2
GO:0140993	histone modifying activity	2/69	404/14323	0.0049505	1.02762233	0.03918365	0.58363557	0.66448552	0.64360815	Cdk2/Ehmt2	2
GO:0016755	aminoacyltransferase activity	2/69	411/14323	0.00486618	1.01012024	0.01448355	0.59308076	0.67232862	0.65120483	Nfx1/Zfp598	2
GO:0043021	ribonucleoprotein complex binding	1/69	190/14323	0.00526316	1.09252479	0.08932556	0.60293104	0.68056165	0.65917918	Zfp598	1
GO:0033218	amide binding	1/69	193/14323	0.00518135	1.07554254	0.07351182	0.6087188	0.68338537	0.66191419	Ager	1
GO:0022853	active monoatomic ion transmembrane transporter activity	1/69	194/14323	0.00515464	1.06999851	0.06829547	0.61062952	0.68338537	0.66191419	Slc30a1	1
GO:0005543	phospholipid binding	2/69	428/14323	0.0046729	0.96999865	-0.0438421	0.61536602	0.68576806	0.66422202	Fundc2/Tiam1	2
GO:0051015	actin filament binding	1/69	201/14323	0.00497512	1.03273488	0.03251761	0.62374946	0.69217767	0.67043025	Abi3bp	1
GO:0008514	organic anion transmembrane transporter activity	1/69	204/14323	0.00490196	1.0175476	0.0175626	0.62923802	0.69533445	0.67348784	Slco1a4	1
GO:0000287	magnesium ion binding	1/69	212/14323	0.00471698	0.97914958	-0.0212795	0.64349137	0.70810975	0.68586175	Cdk2	1
GO:0005509	calcium ion binding	2/69	466/14323	0.00429185	0.89090004	-0.1665869	0.66184397	0.7226625	0.69995728	Ppp2r3a/Enpp1	2
GO:0001664	G protein-coupled receptor binding	1/69	223/14323	0.0044843	0.93085072	-0.0724083	0.66221165	0.7226625	0.69995728	Ccdc88c	1
GO:0035173	histone kinase activity	1/69	226/14323	0.00442478	0.91849429	-0.0859284	0.66714679	0.72503969	0.70225978	Cdk2	1
GO:0022836	gated channel activity	1/69	231/14323	0.004329	0.89861346	-0.1080831	0.67521458	0.7307878	0.70782728	Gabrg1	1
GO:0016791	phosphatase activity	1/69	239/14323	0.0041841	0.86853435	-0.1425956	0.68772395	0.74127623	0.71798618	Enpp1	1
GO:0004672	protein kinase activity	2/69	497/14323	0.00402414	0.83533083	-0.2599562	0.69640233	0.74756658	0.7240789	Epha8/Cdk2	2
GO:0004175	endopeptidase activity	1/69	255/14323	0.00392157	0.81403808	-0.2084663	0.71133521	0.7604214	0.73652983	Psmb9	1
GO:0003713	transcription coactivator activity	1/69	257/14323	0.00389105	0.80770315	-0.216427	0.71416002	0.7604214	0.73652983	Arglu1	1
GO:0035091	phosphatidylinositol binding	1/69	265/14323	0.00377358	0.78331966	-0.2477072	0.72518939	0.76905165	0.74488893	Fundc2	1
GO:0048018	receptor ligand activity	1/69	284/14323	0.00352113	0.73091447	-0.3186687	0.74973108	0.7900111	0.76518986	Tnfsf10	1
GO:0004857	enzyme inhibitor activity	1/69	285/14323	0.00350877	0.72834986	-0.3222833	0.75096112	0.7900111	0.76518986	Pkia	1
GO:0030546	signaling receptor activator activity	1/69	289/14323	0.00346021	0.71826889	-0.3366286	0.75582199	0.79195691	0.76707453	Tnfsf10	1
GO:0042578	phosphoric ester hydrolase activity	1/69	318/14323	0.00314465	0.65276638	-0.4356641	0.78837403	0.82061892	0.79483601	Enpp1	1
GO:0030545	signaling receptor regulator activity	1/69	319/14323	0.0031348	0.65072009	-0.4389357	0.78941668	0.82061892	0.79483601	Tnfsf10	1
GO:0005216	monoatomic ion channel activity	1/69	325/14323	0.00307692	0.6387068	-0.4583809	0.79556705	0.82259961	0.79675447	Gabrg1	1
GO:0106310	protein serine kinase activity	1/69	327/14323	0.0030581	0.63480034	-0.4647938	0.79757757	0.82259961	0.79675447	Cdk2	1
GO:0046873	metal ion transmembrane transporter activity	1/69	346/14323	0.00289017	0.59994136	-0.5240983	0.81573132	0.83803647	0.81170633	Slc30a1	1
GO:0004674	protein serine/threonine kinase activity	1/69	388/14323	0.00257732	0.53499925	-0.6460618	0.85035968	0.87021243	0.84287136	Cdk2	1
GO:0003779	actin binding	1/69	402/14323	0.00248756	0.51636744	-0.6843076	0.86041021	0.87708483	0.84952783	Abi3bp	1
GO:0008233	peptidase activity	1/69	416/14323	0.00240385	0.49898969	-0.7214971	0.86979484	0.88322797	0.85547796	Psmb9	1
GO:0022890	inorganic cation transmembrane transporter activity	1/69	446/14323	0.00224215	0.46542536	-0.7979689	0.88786105	0.8981056	0.86988815	Slc30a1	1
GO:0140297	DNA-binding transcription factor binding	1/69	471/14323	0.00212314	0.44072125	-0.8586982	0.90101047	0.90393293	0.87553239	Dazap2	1
GO:0003712	transcription coregulator activity	1/69	473/14323	0.00211416	0.43885774	-0.8634489	0.90199422	0.90393293	0.87553239	Arglu1	1
GO:0008324	monoatomic cation transmembrane transporter activity	1/69	477/14323	0.00209644	0.43517759	-0.8729049	0.90393293	0.90393293	0.87553239	Slc30a1	1

ID	Description	GeneRatio	BgRatio	RichFactor	FoldEnrichm	zScore	pvalue	p.adjust	qvalue	geneID	Count
GO:0070888	E-box binding	4/96	37/14323	0.10810811	16.1295045	7.56921828	0.00010564	0.03422717	0.03347102	Ciart/Per1/Bhlhe40/Bhlhe41	4
GO:0004715	non-membrane spanning protein tyrosine kinase activity	3/96	34/14323	0.08823529	13.1645221	5.83330086	0.00150189	0.24330629	0.2379311	Wee1/Abl1/Lyn	3
GO:0001227	DNA-binding transcription repressor activity, RNA polymerase II-specific	6/96	224/14323	0.02678571	3.99637277	3.71284211	0.00394003	0.34408649	0.33648484	Nr1d2/Bhlhe40/Bhlhe41/Zbtb49/Zfp281/Ylpm1	6
GO:0001217	DNA-binding transcription repressor activity	6/96	230/14323	0.02608696	3.89211957	3.63211191	0.00448008	0.34408649	0.33648484	Nr1d2/Bhlhe40/Bhlhe41/Zbtb49/Zfp281/Ylpm1	6
GO:0000400	four-way junction DNA binding	2/96	17/14323	0.11764706	17.5526961	5.6093802	0.00566275	0.34408649	0.33648484	Abl1/Xrcc3	2
GO:0004536	DNA nuclease activity	3/96	57/14323	0.05263158	7.85252193	4.25811648	0.00657239	0.34408649	0.33648484	Pole/Xrcc3/Fan1	3
GO:0004713	protein tyrosine kinase activity	4/96	115/14323	0.03478261	5.18949275	3.70531108	0.00743397	0.34408649	0.33648484	Wee1/Rps6ka5/Abl1/Lyn	4
GO:0010485	histone H4 acetyltransferase activity	2/96	21/14323	0.0952381	14.2093254	4.97591193	0.00859316	0.34802291	0.3403343	Brpf1/Naa50	2
GO:0051861	glycolipid binding	2/96	27/14323	0.07407407	11.0516975	4.29432401	0.01399385	0.39245647	0.38378622	Trem2/Lyn	2
GO:0043425	bHLH transcription factor binding	2/96	29/14323	0.06896552	10.2895115	4.11335102	0.01604708	0.39245647	0.38378622	Bhlhe40/Bhlhe41	2
GO:0046875	ephrin receptor binding	2/96	31/14323	0.06451613	9.62567204	3.94919038	0.01822077	0.39245647	0.38378622	Abl1/Lyn	2
GO:0000217	DNA secondary structure binding	2/96	33/14323	0.06060606	9.04229798	3.79928437	0.02051141	0.39245647	0.38378622	Abl1/Xrcc3	2
GO:0004520	DNA endonuclease activity	2/96	37/14323	0.05405405	8.06475225	3.53446155	0.02542982	0.39245647	0.38378622	Xrcc3/Fan1	2
GO:0004402	histone acetyltransferase activity	2/96	41/14323	0.04878049	7.27794715	3.30670807	0.03077557	0.39245647	0.38378622	Brpf1/Naa50	2
GO:0051219	phosphoprotein binding	3/96	103/14323	0.02912621	4.34557039	2.79910875	0.03189074	0.39245647	0.38378622	Abl1/Dpysl4/Lyn	3
GO:0051539	4 iron, 4 sulfur cluster binding	2/96	43/14323	0.04651163	6.93943798	3.2040277	0.03360064	0.39245647	0.38378622	Pole/Rsad1	2
GO:0061733	peptide-lysine-N-acetyltransferase activity	2/96	44/14323	0.04545455	6.78172348	3.15511755	0.03504985	0.39245647	0.38378622	Brpf1/Naa50	2
GO:0009055	electron transfer activity	2/96	47/14323	0.04255319	6.34884752	3.01707886	0.03953962	0.39245647	0.38378622	Por/ND5	2
GO:0034212	peptide N-acetyltransferase activity	2/96	49/14323	0.04081633	6.08971088	2.93156206	0.04264751	0.39245647	0.38378622	Brpf1/Naa50	2
GO:0030246	carbohydrate binding	4/96	204/14323	0.01960784	2.92544935	2.27523363	0.04824302	0.39245647	0.38378622	Galnt15/Clec1a/Alpk1/Gpcpd1	4
GO:0016651	oxidoreductase activity, acting on NAD(P)H	2/96	53/14323	0.03773585	5.63011006	2.77394629	0.04912545	0.39245647	0.38378622	Por/ND5	2
GO:0004672	protein kinase activity	7/96	497/14323	0.01408451	2.10137911	2.05280425	0.04927351	0.39245647	0.38378622	Mylk4/Wee1/Alpk1/Rps6ka5/Abl1/Lyn/Cdk19	7
GO:0001221	transcription coregulator binding	3/96	123/14323	0.02439024	3.63897358	2.41448333	0.0496498	0.39245647	0.38378622	Per3/Per1/Zbtb49	3
GO:0140299	molecular sensor activity	2/96	55/14323	0.03636364	5.42537879	2.70104009	0.05248986	0.39245647	0.38378622	Sar1b/Xrcc3	2
GO:0001222	transcription corepressor binding	2/96	56/14323	0.03571429	5.32849702	2.66591066	0.0542022	0.39245647	0.38378622	Per3/Per1	2
GO:0004896	cytokine receptor activity	2/96	59/14323	0.03389831	5.0575565	2.56537408	0.05945579	0.39245647	0.38378622	Fzd4/Il15ra	2
GO:0005178	integrin binding	3/96	133/14323	0.02255639	3.36536654	2.25119885	0.05999947	0.39245647	0.38378622	Tspan4/Lyn/Jam2	3
GO:0016705	oxidoreductase activity, acting on paired donors, with incorporation or reduction of molecular oxygen	3/96	134/14323	0.02238806	3.34025187	2.23573276	0.0610862	0.39245647	0.38378622	Por/Cyp3a13/Tet3	3
GO:0001540	amyloid-beta binding	2/96	62/14323	0.03225806	4.81283602	2.47143827	0.06487729	0.39245647	0.38378622	Fzd4/Trem2	2
GO:0005225	volume-sensitive anion channel activity	1/96	10/14323	0.1	14.9197917	3.61699731	0.06505904	0.39245647	0.38378622	Clcn2	1
GO:0005247	voltage-gated chloride channel activity	1/96	10/14323	0.1	14.9197917	3.61699731	0.06505904	0.39245647	0.38378622	Clcn2	1
GO:0008035	high-density lipoprotein particle binding	1/96	10/14323	0.1	14.9197917	3.61699731	0.06505904	0.39245647	0.38378622	Trem2	1
GO:0016653	oxidoreductase activity, acting on NAD(P)H, heme protein as acceptor	1/96	10/14323	0.1	14.9197917	3.61699731	0.06505904	0.39245647	0.38378622	Por	1
GO:0016832	aldehyde-lyase activity	1/96	10/14323	0.1	14.9197917	3.61699731	0.06505904	0.39245647	0.38378622	Tha1	1
GO:0033691	sialic acid binding	1/96	10/14323	0.1	14.9197917	3.61699731	0.06505904	0.39245647	0.38378622	Agrn	1
GO:0070097	delta-catenin binding	1/96	10/14323	0.1	14.9197917	3.61699731	0.06505904	0.39245647	0.38378622	Abl1	1
GO:0140161	monocarboxylate:sodium symporter activity	1/96	10/14323	0.1	14.9197917	3.61699731	0.06505904	0.39245647	0.38378622	Slc6a6	1
GO:0008080	N-acetyltransferase activity	2/96	65/14323	0.03076923	4.59070513	2.38335042	0.07045806	0.39245647	0.38378622	Brpf1/Naa50	2
GO:0008395	steroid hydroxylase activity	1/96	11/14323	0.09090909	13.563447	3.42401592	0.07132987	0.39245647	0.38378622	Cyp3a13	1
GO:0010181	FMN binding	1/96	11/14323	0.09090909	13.563447	3.42401592	0.07132987	0.39245647	0.38378622	Por	1
GO:0016894	endonuclease activity, active with either ribo- or deoxyribonucleic acids and producing 3'-phosphomonoe	1/96	11/14323	0.09090909	13.563447	3.42401592	0.07132987	0.39245647	0.38378622	Xrcc3	1
GO:0043995	histone H4K5 acetyltransferase activity	1/96	11/14323	0.09090909	13.563447	3.42401592	0.07132987	0.39245647	0.38378622	Brpf1	1
GO:0043996	histone H4K8 acetyltransferase activity	1/96	11/14323	0.09090909	13.563447	3.42401592	0.07132987	0.39245647	0.38378622	Brpf1	1
GO:0004861	cyclin-dependent protein serine/threonine kinase inhibitor activity	1/96	12/14323	0.08333333	12.4331597	3.25463798	0.07755907	0.39245647	0.38378622	Inca1	1
GO:0015378	sodium:chloride symporter activity	1/96	12/14323	0.08333333	12.4331597	3.25463798	0.07755907	0.39245647	0.38378622	Slc6a6	1
GO:0015643	toxic substance binding	1/96	12/14323	0.08333333	12.4331597	3.25463798	0.07755907	0.39245647	0.38378622	Gsta4	1
GO:0016888	endodeoxyribonuclease activity, producing 5'-phosphomonoesters	1/96	12/14323	0.08333333	12.4331597	3.25463798	0.07755907	0.39245647	0.38378622	Fan1	1
GO:0043997	histone H4K12 acetyltransferase activity	1/96	12/14323	0.08333333	12.4331597	3.25463798	0.07755907	0.39245647	0.38378622	Brpf1	1
GO:0140031	phosphorylation-dependent protein binding	1/96	12/14323	0.08333333	12.4331597	3.25463798	0.07755907	0.39245647	0.38378622	Lyn	1
GO:0140996	histone H3 kinase activity	1/96	12/14323	0.08333333	12.4331597	3.25463798	0.07755907	0.39245647	0.38378622	Rps6ka5	1
GO:0051536	iron-sulfur cluster binding	2/96	69/14323	0.02898551	4.32457729	2.27391061	0.0781324	0.39245647	0.38378622	Pole/Rsad1	2
GO:0051540	metal cluster binding	2/96	70/14323	0.02857143	4.26279762	2.24784727	0.08009065	0.39245647	0.38378622	Pole/Rsad1	2
GO:0005545	1-phosphatidylinositol binding	1/96	13/14323	0.07692308	11.4767628	3.10427192	0.08374692	0.39245647	0.38378622	Adap2	1
GO:0008296	3'-5'-DNA exonuclease activity	1/96	13/14323	0.07692308	11.4767628	3.10427192	0.08374692	0.39245647	0.38378622	Pole	1
GO:0015355	secondary active monocarboxylate transmembrane transporter activity	1/96	13/14323	0.07692308	11.4767628	3.10427192	0.08374692	0.39245647	0.38378622	Slc6a6	1
GO:0015373	monoatomic anion:sodium symporter activity	1/96	13/14323	0.07692308	11.4767628	3.10427192	0.08374692	0.39245647	0.38378622	Slc6a6	1
GO:0016812	hydrolase activity, acting on carbon-nitrogen (but not peptide) bonds, in cyclic amides	1/96	13/14323	0.07692308	11.4767628	3.10427192	0.08374692	0.39245647	0.38378622	Dpysl4	1
GO:0017154	semaphorin receptor activity	1/96	13/14323	0.07692308	11.4767628	3.10427192	0.08374692	0.39245647	0.38378622	Trem2	1
GO:0140664	ATP-dependent DNA damage sensor activity	1/96	13/14323	0.07692308	11.4767628	3.10427192	0.08374692	0.39245647	0.38378622	Xrcc3	1
GO:0004527	exonuclease activity	2/96	72/14323	0.02777778	4.14438657	2.19715298	0.08405269	0.39245647	0.38378622	Pole/Fan1	2
GO:0005161	platelet-derived growth factor receptor binding	1/96	14/14323	0.07142857	10.656994	2.96949176	0.08989369	0.39245647	0.38378622	Lyn	1
GO:0008308	voltage-gated monoatomic anion channel activity	1/96	14/14323	0.07142857	10.656994	2.96949176	0.08989369	0.39245647	0.38378622	Clcn2	1
GO:0008429	phosphatidylethanolamine binding	1/96	14/14323	0.07142857	10.656994	2.96949176	0.08989369	0.39245647	0.38378622	Trem2	1
GO:0022858	alanine transmembrane transporter activity	1/96	14/14323	0.07142857	10.656994	2.96949176	0.08989369	0.39245647	0.38378622	Slc6a6	1
GO:0034450	ubiquitin-ubiquitin ligase activity	1/96	14/14323	0.07142857	10.656994	2.96949176	0.08989369	0.39245647	0.38378622	Peli1	1
GO:0036122	BMP binding	1/96	14/14323	0.07142857	10.656994	2.96949176	0.08989369	0.39245647	0.38378622	Agrn	1
GO:0048038	quinone binding	1/96	14/14323	0.07142857	10.656994	2.96949176	0.08989369	0.39245647	0.38378622	Coq10b	1
GO:0070742	C2H2 zinc finger domain binding	1/96	14/14323	0.07142857	10.656994	2.96949176	0.08989369	0.39245647	0.38378622	Srrm2	1
GO:0008081	phosphoric diester hydrolase activity	2/96	76/14323	0.02631579	3.92626096	2.10106227	0.09215102	0.39245647	0.38378622	Gpcpd1/Fan1	2
GO:0051213	dioxygenase activity	2/96	77/14323	0.02597403	3.87527056	2.0780615	0.09421028	0.39245647	0.38378622	Por/Tet3	2
GO:0004622	lysophospholipase activity	1/96	15/14323	0.06666667	9.94652778	2.84768183	0.09599966	0.39245647	0.38378622	Pnpla7	1
GO:0030169	low-density lipoprotein particle binding	1/96	15/14323	0.06666667	9.94652778	2.84768183	0.09599966	0.39245647	0.38378622	Trem2	1
GO:0030548	acetylcholine receptor regulator activity	1/96	15/14323	0.06666667	9.94652778	2.84768183	0.09599966	0.39245647	0.38378622	Agrn	1
GO:0031005	filamin binding	1/96	15/14323	0.06666667	9.94652778	2.84768183	0.09599966	0.39245647	0.38378622	Dpysl4	1
GO:0035014	phosphatidylinositol 3-kinase regulator activity	1/96	15/14323	0.06666667	9.94652778	2.84768183	0.09599966	0.39245647	0.38378622	Lyn	1
GO:0042813	Wnt receptor activity	1/96	15/14323	0.06666667	9.94652778	2.84768183	0.09599966	0.39245647	0.38378622	Fzd4	1
GO:0097110	scaffold protein binding	2/96	78/14323	0.02564103	3.82558761	2.05544394	0.09628287	0.39245647	0.38378622	Trem2/Lyn	2
GO:0015108	chloride transmembrane transporter activity	2/96	80/14323	0.025	3.72994792	2.01131181	0.10046698	0.39245647	0.38378622	Slc6a6/Clcn2	2
GO:0016410	N-acyltransferase activity	2/96	80/14323	0.025	3.72994792	2.01131181	0.10046698	0.39245647	0.38378622	Brpf1/Naa50	2
GO:0050660	flavin adenine dinucleotide binding	2/96	80/14323	0.025	3.72994792	2.01131181	0.10046698	0.39245647	0.38378622	Por/Pipox	2
GO:0140375	immune receptor activity	2/96	80/14323	0.025	3.72994792	2.01131181	0.10046698	0.39245647	0.38378622	Fzd4/Il15ra	2
GO:0099602	neurotransmitter receptor regulator activity	1/96	16/14323	0.0625	9.32486979	2.73680552	0.10206508	0.39245647	0.38378622	Agrn	1
GO:0140612	DNA damage sensor activity	1/96	16/14323	0.0625	9.32486979	2.73680552	0.10206508	0.39245647	0.38378622	Xrcc3	1
GO:0004653	polypeptide N-acetylgalactosaminyltransferase activity	1/96	17/14323	0.05882353	8.77634804	2.63525002	0.10809022	0.39245647	0.38378622	Galnt15	1
GO:0008137	NADH dehydrogenase (ubiquinone) activity	1/96	17/14323	0.05882353	8.77634804	2.63525002	0.10809022	0.39245647	0.38378622	ND5	1
GO:0008409	5'-3' exonuclease activity	1/96	17/14323	0.05882353	8.77634804	2.63525002	0.10809022	0.39245647	0.38378622	Fan1	1
GO:0140036	ubiquitin-modified protein reader activity	1/96	17/14323	0.05882353	8.77634804	2.63525002	0.10809022	0.39245647	0.38378622	Fan1	1
GO:0020037	heme binding	2/96	84/14323	0.02380952	3.55233135	1.92715985	0.10898361	0.39245647	0.38378622	Cyp3a13/Rsad1	2
GO:0004518	nuclease activity	3/96	175/14323	0.01714286	2.55767857	1.70306071	0.11300749	0.39245647	0.38378622	Pole/Xrcc3/Fan1	3
GO:0015377	chloride:monoatomic cation symporter activity	1/96	18/14323	0.05555556	8.28877315	2.54171903	0.11407536	0.39245647	0.38378622	Slc6a6	1
GO:0034185	apolipoprotein binding	1/96	18/14323	0.05555556	8.28877315	2.54171903	0.11407536	0.39245647	0.38378622	Trem2	1
GO:0043395	heparan sulfate proteoglycan binding	1/96	18/14323	0.05555556	8.28877315	2.54171903	0.11407536	0.39245647	0.38378622	Agrn	1
GO:0046527	glucosyltransferase activity	1/96	18/14323	0.05555556	8.28877315	2.54171903	0.11407536	0.39245647	0.38378622	Alg10b	1
GO:0016407	acetyltransferase activity	2/96	88/14323	0.02272727	3.39086174	1.84798248	0.11768467	0.39245647	0.38378622	Brpf1/Naa50	2
GO:0046906	tetrapyrrole binding	2/96	91/14323	0.02197802	3.27907509	1.79154264	0.12432213	0.39245647	0.38378622	Cyp3a13/Rsad1	2
GO:0005283	amino acid:sodium symporter activity	1/96	20/14323	0.05	7.45989583	2.3746944	0.12592664	0.39245647	0.38378622	Slc6a6	1
GO:0015296	monoatomic anion:monoatomic cation symporter activity	1/96	20/14323	0.05	7.45989583	2.3746944	0.12592664	0.39245647	0.38378622	Slc6a6	1
GO:0030247	polysaccharide binding	1/96	20/14323	0.05	7.45989583	2.3746944	0.12592664	0.39245647	0.38378622	Gpcpd1	1
GO:0070064	proline-rich region binding	1/96	20/14323	0.05	7.45989583	2.3746944	0.12592664	0.39245647	0.38378622	Abl1	1
GO:0140035	ubiquitin-like protein reader activity	1/96	20/14323	0.05	7.45989583	2.3746944	0.12592664	0.39245647	0.38378622	Fan1	1
GO:0003714	transcription corepressor activity	3/96	184/14323	0.01630435	2.43257473	1.60656254	0.12611646	0.39245647	0.38378622	Bhlhe41/Rere/Eid1	3
GO:0140030	modification-dependent protein binding	3/96	184/14323	0.01630435	2.43257473	1.60656254	0.12611646	0.39245647	0.38378622	Ncapd3/Fan1/Lyn	3
GO:0003887	DNA-directed DNA polymerase activity	1/96	21/14323	0.04761905	7.1046627	2.29960751	0.13179332	0.39245647	0.38378622	Pole	1
GO:0016895	DNA exonuclease activity, producing 5'-phosphomonoesters	1/96	21/14323	0.04761905	7.1046627	2.29960751	0.13179332	0.39245647	0.38378622	Pole	1
GO:0038191	neuropilin binding	1/96	21/14323	0.04761905	7.1046627	2.29960751	0.13179332	0.39245647	0.38378622	Abl1	1
GO:0140993	histone modifying activity	5/96	404/14323	0.01237624	1.84650887	1.41774774	0.13480656	0.39245647	0.38378622	Mylk4/Alpk1/Rps6ka5/Brpf1/Naa50	5
GO:0003950	NAD+-protein poly-ADP-ribosyltransferase activity	1/96	22/14323	0.04545455	6.78172348	2.22928832	0.13762102	0.39245647	0.38378622	Art5	1
GO:0004529	DNA exonuclease activity	1/96	22/14323	0.04545455	6.78172348	2.22928832	0.13762102	0.39245647	0.38378622	Pole	1
GO:0008327	methyl-CpG binding	1/96	22/14323	0.04545455	6.78172348	2.22928832	0.13762102	0.39245647	0.38378622	Tet3	1
GO:0030215	semaphorin receptor binding	1/96	22/14323	0.04545455	6.78172348	2.22928832	0.13762102	0.39245647	0.38378622	Trem2	1
GO:0031625	ubiquitin protein ligase binding	4/96	297/14323	0.01346801	2.00939955	1.44396012	0.13865317	0.39245647	0.38378622	Per3/Per1/Fzd4/Lyn	4
GO:0005112	Notch binding	1/96	23/14323	0.04347826	6.48686594	2.16322172	0.14341002	0.39245647	0.38378622	Tcim	1
GO:0050431	transforming growth factor beta binding	1/96	23/14323	0.04347826	6.48686594	2.16322172	0.14341002	0.39245647	0.38378622	Agrn	1
GO:0008509	monoatomic anion transmembrane transporter activity	2/96	100/14323	0.02	2.98395833	1.6353764	0.14474248	0.39245647	0.38378622	Slc6a6/Clcn2	2
GO:0019955	cytokine binding	2/96	100/14323	0.02	2.98395833	1.6353764	0.14474248	0.39245647	0.38378622	Fzd4/Agrn	2
GO:0004364	glutathione transferase activity	1/96	24/14323	0.04166667	6.21657986	2.10096798	0.14916055	0.39245647	0.38378622	Gsta4	1
GO:0005416	amino acid:monoatomic cation symporter activity	1/96	24/14323	0.04166667	6.21657986	2.10096798	0.14916055	0.39245647	0.38378622	Slc6a6	1
GO:0016645	oxidoreductase activity, acting on the CH-NH group of donors	1/96	24/14323	0.04166667	6.21657986	2.10096798	0.14916055	0.39245647	0.38378622	Pipox	1
GO:0016712	oxidoreductase activity, acting on paired donors, with incorporation or reduction of molecular oxygen, red	1/96	24/14323	0.04166667	6.21657986	2.10096798	0.14916055	0.39245647	0.38378622	Cyp3a13	1
GO:0030742	GTP-dependent protein binding	1/96	24/14323	0.04166667	6.21657986	2.10096798	0.14916055	0.39245647	0.38378622	Rab34	1
GO:0004519	endonuclease activity	2/96	103/14323	0.01941748	2.89704693	1.58718545	0.15169896	0.39245647	0.38378622	Xrcc3/Fan1	2
GO:0015103	inorganic anion transmembrane transporter activity	2/96	103/14323	0.01941748	2.89704693	1.58718545	0.15169896	0.39245647	0.38378622	Slc6a6/Clcn2	2
GO:0005343	organic acid:sodium symporter activity	1/96	25/14323	0.04	5.96791667	2.04214909	0.15487288	0.39245647	0.38378622	Slc6a6	1
GO:0016866	intramolecular transferase activity	1/96	25/14323	0.04	5.96791667	2.04214909	0.15487288	0.39245647	0.38378622	Lss	1
GO:0035035	histone acetyltransferase binding	1/96	25/14323	0.04	5.96791667	2.04214909	0.15487288	0.39245647	0.38378622	Eid1	1
GO:1990404	NAD+-protein ADP-ribosyltransferase activity	1/96	25/14323	0.04	5.96791667	2.04214909	0.15487288	0.39245647	0.38378622	Art5	1
GO:0044389	ubiquitin-like protein ligase binding	4/96	313/14323	0.01277955	1.90668264	1.33226061	0.15846714	0.39245647	0.38378622	Per3/Per1/Fzd4/Lyn	4
GO:0034061	DNA polymerase activity	1/96	26/14323	0.03846154	5.73838141	1.98643801	0.16054725	0.39245647	0.38378622	Pole	1
GO:0071813	lipoprotein particle binding	1/96	26/14323	0.03846154	5.73838141	1.98643801	0.16054725	0.39245647	0.38378622	Trem2	1
GO:0071814	protein-lipid complex binding	1/96	26/14323	0.03846154	5.73838141	1.98643801	0.16054725	0.39245647	0.38378622	Trem2	1
GO:0016810	hydrolase activity, acting on carbon-nitrogen (but not peptide) bonds	2/96	107/14323	0.01869159	2.78874611	1.52557186	0.16107526	0.39245647	0.38378622	Acer2/Dpysl4	2
GO:0043325	phosphatidylinositol-3,4-bisphosphate binding	1/96	27/14323	0.03703704	5.52584877	1.93355013	0.16618392	0.39245647	0.38378622	Adap2	1
GO:0046625	sphingolipid binding	1/96	27/14323	0.03703704	5.52584877	1.93355013	0.16618392	0.39245647	0.38378622	Lyn	1
GO:0000287	magnesium ion binding	3/96	212/14323	0.01415094	2.11129127	1.33905287	0.17003049	0.39245647	0.38378622	Wee1/Rps6ka5/Abl1	3
GO:0017147	Wnt-protein binding	1/96	28/14323	0.03571429	5.32849702	1.88323642	0.17178314	0.39245647	0.38378622	Fzd4	1
GO:0106310	protein serine kinase activity	4/96	327/14323	0.01223242	1.82505097	1.23974816	0.17661973	0.39245647	0.38378622	Mylk4/Alpk1/Rps6ka5/Cdk19	4
GO:0044024	histone H2AS1 kinase activity	3/96	216/14323	0.01388889	2.07219329	1.30425771	0.17663671	0.39245647	0.38378622	Mylk4/Alpk1/Rps6ka5	3
GO:0140097	catalytic activity, acting on DNA	3/96	216/14323	0.01388889	2.07219329	1.30425771	0.17663671	0.39245647	0.38378622	Pole/Xrcc3/Fan1	3
GO:0140995	histone H2A kinase activity	3/96	216/14323	0.01388889	2.07219329	1.30425771	0.17663671	0.39245647	0.38378622	Mylk4/Alpk1/Rps6ka5	3
GO:0000146	microfilament motor activity	1/96	29/14323	0.03448276	5.14475575	1.83527791	0.17734514	0.39245647	0.38378622	Myo5b	1
GO:0001530	lipopolysaccharide binding	1/96	29/14323	0.03448276	5.14475575	1.83527791	0.17734514	0.39245647	0.38378622	Trem2	1
GO:0004693	cyclin-dependent protein serine/threonine kinase activity	1/96	29/14323	0.03448276	5.14475575	1.83527791	0.17734514	0.39245647	0.38378622	Cdk19	1
GO:0008376	acetylgalactosaminyltransferase activity	1/96	29/14323	0.03448276	5.14475575	1.83527791	0.17734514	0.39245647	0.38378622	Galnt15	1
GO:0016655	oxidoreductase activity, acting on NAD(P)H, quinone or similar compound as acceptor	1/96	29/14323	0.03448276	5.14475575	1.83527791	0.17734514	0.39245647	0.38378622	ND5	1
GO:0043236	laminin binding	1/96	29/14323	0.03448276	5.14475575	1.83527791	0.17734514	0.39245647	0.38378622	Agrn	1
GO:0097472	cyclin-dependent protein kinase activity	1/96	29/14323	0.03448276	5.14475575	1.83527791	0.17734514	0.39245647	0.38378622	Cdk19	1
GO:0016757	glycosyltransferase activity	3/96	217/14323	0.01382488	2.06264401	1.29567629	0.17829957	0.39245647	0.38378622	Art5/Galnt15/Alg10b	3
GO:0005525	GTP binding	4/96	329/14323	0.01215805	1.81395643	1.22689942	0.17927024	0.39245647	0.38378622	Gimap5/Sar1b/Rab34/Arl11	4
GO:0035173	histone kinase activity	3/96	226/14323	0.01327434	1.98050332	1.22045206	0.19345628	0.41762554	0.40839924	Mylk4/Alpk1/Rps6ka5	3
GO:0015453	oxidoreduction-driven active transmembrane transporter activity	1/96	32/14323	0.03125	4.6624349	1.70370492	0.19381032	0.41762554	0.40839924	ND5	1
GO:0017124	SH3 domain binding	2/96	121/14323	0.01652893	2.46608127	1.33032196	0.19463412	0.41762554	0.40839924	Abl1/Lyn	2
GO:0043015	gamma-tubulin binding	1/96	33/14323	0.03030303	4.52114899	1.66343579	0.19922591	0.42004067	0.41076102	Lyn	1
GO:0051019	mitogen-activated protein kinase binding	1/96	33/14323	0.03030303	4.52114899	1.66343579	0.19922591	0.42004067	0.41076102	Abl1	1
GO:0019001	guanyl nucleotide binding	4/96	345/14323	0.0115942	1.72983092	1.12717169	0.20094538	0.42004067	0.41076102	Gimap5/Sar1b/Rab34/Arl11	4
GO:0032561	guanyl ribonucleotide binding	4/96	345/14323	0.0115942	1.72983092	1.12717169	0.20094538	0.42004067	0.41076102	Gimap5/Sar1b/Rab34/Arl11	4
GO:0016779	nucleotidyltransferase activity	2/96	127/14323	0.01574803	2.34957349	1.25486264	0.20928568	0.43052898	0.42101762	Art5/Pole	2
GO:0016893	endonuclease activity, active with either ribo- or deoxyribonucleic acids and producing 5'-phosphomonoe	1/96	35/14323	0.02857143	4.26279762	1.58752007	0.20994932	0.43052898	0.42101762	Fan1	1
GO:0030332	cyclin binding	1/96	35/14323	0.02857143	4.26279762	1.58752007	0.20994932	0.43052898	0.42101762	Inca1	1
GO:0015175	neutral L-amino acid transmembrane transporter activity	1/96	36/14323	0.02777778	4.14438657	1.55166315	0.21525761	0.43589666	0.42626672	Slc6a6	1
GO:0043394	proteoglycan binding	1/96	36/14323	0.02777778	4.14438657	1.55166315	0.21525761	0.43589666	0.42626672	Agrn	1
GO:0001228	DNA-binding transcription activator activity, RNA polymerase II-specific	4/96	357/14323	0.01120448	1.67168534	1.05571027	0.21770196	0.43810829	0.42842949	Dbp/Tef/Hlf/Klf9	4
GO:0019207	kinase regulator activity	3/96	242/14323	0.01239669	1.84956095	1.09487934	0.22114721	0.44229443	0.43252314	Trem2/Inca1/Lyn	3
GO:0030291	protein serine/threonine kinase inhibitor activity	1/96	38/14323	0.02631579	3.92626096	1.48369801	0.22576853	0.44692837	0.4370547	Inca1	1
GO:0001216	DNA-binding transcription activator activity	4/96	363/14323	0.01101928	1.64405418	1.0209719	0.226223	0.44692837	0.4370547	Dbp/Tef/Hlf/Klf9	4
GO:0046982	protein heterodimerization activity	3/96	249/14323	0.01204819	1.79756526	1.04288859	0.23351234	0.45853332	0.44840328	Bhlhe40/Fzd4/Bhlhe41	3

GO:0016538	cyclin-dependent protein serine/threonine kinase regulator activity	1/96	40/14323	0.025	3.72994792	1.42021935	0.23614012	0.4609	0.45071767	Inca1	1
GO:0050839	cell adhesion molecule binding	3/96	257/14323	0.01167315	1.74160992	0.98545676	0.24779574	0.47958663	0.46899147	Tspan4/Lyn/Jam2	3
GO:0042169	SH2 domain binding	1/96	43/14323	0.02325581	3.46971899	1.33228903	0.25144024	0.47958663	0.46899147	Abl1	1
GO:0001784	phosphotyrosine residue binding	1/96	44/14323	0.02272727	3.39086174	1.30470609	0.25647257	0.47958663	0.46899147	Abl1	1
GO:0004879	nuclear receptor activity	1/96	44/14323	0.02272727	3.39086174	1.30470609	0.25647257	0.47958663	0.46899147	Nr1d2	1
GO:0005547	phosphatidylinositol-3,4,5-trisphosphate binding	1/96	44/14323	0.02272727	3.39086174	1.30470609	0.25647257	0.47958663	0.46899147	Adap2	1
GO:0001223	transcription coactivator binding	1/96	45/14323	0.02222222	3.31550926	1.27790884	0.26147142	0.47958663	0.46899147	Zbtb49	1
GO:0050661	NADP binding	1/96	45/14323	0.02222222	3.31550926	1.27790884	0.26147142	0.47958663	0.46899147	Por	1
GO:0098531	ligand-activated transcription factor activity	1/96	45/14323	0.02222222	3.31550926	1.27790884	0.26147142	0.47958663	0.46899147	Nr1d2	1
GO:0004674	protein serine/threonine kinase activity	4/96	388/14323	0.01030928	1.53812285	0.88272331	0.26258871	0.47958663	0.46899147	Mylk4/Alpk1/Rps6ka5/Cdk19	4
GO:0043177	organic acid binding	2/96	149/14323	0.01342282	2.0026566	1.01060146	0.26373122	0.47958663	0.46899147	Slc6a6/Agrn	2
GO:0016758	hexosyltransferase activity	2/96	150/14323	0.01333333	1.98930556	1.00052045	0.26621817	0.47958663	0.46899147	Galnt15/Alg10b	2
GO:0005544	calcium-dependent phospholipid binding	1/96	46/14323	0.02173913	3.24343297	1.25185584	0.26643702	0.47958663	0.46899147	Cpne7	1
GO:0016830	carbon-carbon lyase activity	1/96	46/14323	0.02173913	3.24343297	1.25185584	0.26643702	0.47958663	0.46899147	Tha1	1
GO:0060590	ATPase regulator activity	1/96	46/14323	0.02173913	3.24343297	1.25185584	0.26643702	0.47958663	0.46899147	Agrn	1
GO:0005507	copper ion binding	1/96	47/14323	0.0212766	3.17442376	1.22650869	0.27136957	0.48309748	0.47242476	Mt1	1
GO:0008408	3'-5' exonuclease activity	1/96	47/14323	0.0212766	3.17442376	1.22650869	0.27136957	0.48309748	0.47242476	Pole	1
GO:0016763	pentosyltransferase activity	1/96	48/14323	0.02083333	3.10828993	1.20183177	0.2762693	0.48913253	0.47832648	Art5	1
GO:0003924	GTPase activity	3/96	275/14323	0.01090909	1.62761364	0.86324139	0.2803881	0.4897215	0.47890244	Sar1b/Rab34/Arl11	3
GO:0003823	antigen binding	1/96	49/14323	0.02040816	3.04485544	1.17779198	0.28113642	0.4897215	0.47890244	Tspan4	1
GO:0015370	solute:sodium symporter activity	1/96	49/14323	0.02040816	3.04485544	1.17779198	0.28113642	0.4897215	0.47890244	Slc6a6	1
GO:0016796	exonuclease activity, active with either ribo- or deoxyribonucleic acids and producing 5'-phosphomonoes	1/96	50/14323	0.02	2.98395833	1.15435849	0.28597115	0.49547942	0.48453316	Pole	1
GO:0005254	chloride channel activity	1/96	51/14323	0.01960784	2.92544935	1.13150259	0.29077369	0.50112062	0.49004973	Clcn2	1
GO:0035255	ionotropic glutamate receptor binding	1/96	52/14323	0.01923077	2.86919071	1.10919746	0.29554427	0.50217851	0.49108425	Myo5b	1
GO:1901682	sulfur compound transmembrane transporter activity	1/96	52/14323	0.01923077	2.86919071	1.10919746	0.29554427	0.50217851	0.49108425	Slc6a6	1
GO:0044325	transmembrane transporter binding	2/96	162/14323	0.01234568	1.84194959	0.88527341	0.29603733	0.50217851	0.49108425	Agrn/Lyn	2
GO:0001786	phosphatidylserine binding	1/96	53/14323	0.01886792	2.81505503	1.08741803	0.3002831	0.5041022	0.49296544	Trem2	1
GO:0004867	serine-type endopeptidase inhibitor activity	1/96	53/14323	0.01886792	2.81505503	1.08741803	0.3002831	0.5041022	0.49296544	Serpine1	1
GO:0003925	G protein activity	1/96	54/14323	0.01851852	2.76292438	1.06614084	0.30499037	0.50936537	0.49811233	Sar1b	1
GO:0008028	monocarboxylic acid transmembrane transporter activity	1/96	56/14323	0.01785714	2.66424851	1.02500656	0.31431111	0.51693807	0.50551774	Slc6a6	1
GO:0016627	oxidoreductase activity, acting on the CH-CH group of donors	1/96	56/14323	0.01785714	2.66424851	1.02500656	0.31431111	0.51693807	0.50551774	Rsad1	1
GO:0016765	transferase activity, transferring alkyl or aryl (other than methyl) groups	1/96	56/14323	0.01785714	2.66424851	1.02500656	0.31431111	0.51693807	0.50551774	Gsta4	1
GO:0005543	phospholipid binding	4/96	428/14323	0.00934579	1.39437305	0.68042557	0.3227839	0.52671676	0.51508039	Trem2/Cpne7/Syt16/Adap2	4
GO:0005080	protein kinase C binding	1/96	58/14323	0.01724138	2.57237787	0.98563432	0.32350813	0.52671676	0.51508039	Abl1	1
GO:0016706	2-oxoglutarate-dependent dioxygenase activity	1/96	59/14323	0.01694915	2.52877825	0.96656401	0.32806076	0.52881436	0.51713165	Tet3	1
GO:0045309	protein phosphorylated amino acid binding	1/96	59/14323	0.01694915	2.52877825	0.96656401	0.32806076	0.52881436	0.51713165	Abl1	1
GO:0005539	glycosaminoglycan binding	2/96	178/14323	0.01123596	1.67638109	0.74590027	0.33551255	0.53814884	0.52625991	Trem2/Agrn	2
GO:0004497	monooxygenase activity	1/96	63/14323	0.01587302	2.3682209	0.89402058	0.34597005	0.54680144	0.53472136	Cyp3a13	1
GO:0016811	hydrolase activity, acting on carbon-nitrogen (but not peptide) bonds, in linear amides	1/96	63/14323	0.01587302	2.3682209	0.89402058	0.34597005	0.54680144	0.53472136	Acer2	1
GO:0050840	extracellular matrix binding	1/96	63/14323	0.01587302	2.3682209	0.89402058	0.34597005	0.54680144	0.53472136	Agrn	1
GO:0005253	monoatomic anion channel activity	1/96	64/14323	0.015625	2.33121745	0.87674892	0.35037305	0.54841	0.53629438	Clcn2	1
GO:0030145	manganese ion binding	1/96	64/14323	0.015625	2.33121745	0.87674892	0.35037305	0.54841	0.53629438	Abl1	1
GO:0042578	phosphoric ester hydrolase activity	3/96	318/14323	0.00943396	1.40752752	0.60368408	0.35932797	0.55972241	0.54735687	Tns2/Gpcpd1/Fan1	3
GO:0016747	acyltransferase activity, transferring groups other than amino-acyl groups	2/96	189/14323	0.01058201	1.57881393	0.65798822	0.36229728	0.56164746	0.5492394	Brpf1/Naa50	2
GO:0019905	syntaxin binding	1/96	68/14323	0.01470588	2.19408701	0.81075121	0.36769366	0.56578601	0.55328652	Abl1	1
GO:0033218	amide binding	2/96	193/14323	0.01036269	1.5460924	0.62741441	0.37194272	0.56578601	0.55328652	Fzd4/Trem2	2
GO:0004620	phospholipase activity	1/96	69/14323	0.01449275	2.16228865	0.79497038	0.37195191	0.56578601	0.55328652	Pnpla7	1
GO:0048029	monosaccharide binding	1/96	69/14323	0.01449275	2.16228865	0.79497038	0.37195191	0.56578601	0.55328652	Alpk1	1
GO:0022853	active monoatomic ion transmembrane transporter activity	2/96	194/14323	0.01030928	1.53812285	0.61987964	0.37434534	0.56676584	0.5542447	Slc6a6/ND5	2
GO:0015101	organic cation transmembrane transporter activity	1/96	70/14323	0.01428571	2.13139881	0.77945739	0.37618179	0.56689721	0.55437316	Slc6a6	1
GO:0004860	protein kinase inhibitor activity	1/96	71/14323	0.01408451	2.10137911	0.76420326	0.38038347	0.5705752	0.5579699	Inca1	1
GO:0005546	phosphatidylinositol-4,5-bisphosphate binding	1/96	72/14323	0.01388889	2.07219329	0.74919945	0.38455714	0.57330662	0.56064098	Adap2	1
GO:0140297	DNA-binding transcription factor binding	4/96	471/14323	0.00849257	1.2670736	0.48413442	0.38853355	0.57330662	0.56064098	Per1/Bhlhe40/Bhlhe41/Zbtb49	4
GO:0042277	peptide binding	2/96	200/14323	0.01	1.49197917	0.57554464	0.38868302	0.57330662	0.56064098	Fzd4/Trem2	2
GO:0051015	actin filament binding	2/96	201/14323	0.00995025	1.48455638	0.56829656	0.39105909	0.57330662	0.56064098	Myo5b/Abl1	2
GO:0019210	kinase inhibitor activity	1/96	74/14323	0.01351351	2.01618806	0.71991056	0.3928212	0.57330662	0.56064098	Inca1	1
GO:0035064	methylated histone binding	1/96	74/14323	0.01351351	2.01618806	0.71991056	0.3928212	0.57330662	0.56064098	Ncapd3	1
GO:0140034	methylation-dependent protein binding	1/96	76/14323	0.01315789	1.96313048	0.69152996	0.40097544	0.58258315	0.56971256	Ncapd3	1
GO:0003774	cytoskeletal motor activity	1/96	77/14323	0.01298701	1.93763528	0.67766276	0.40501182	0.58321702	0.57033244	Myo5b	1
GO:0015294	solute:monoatomic cation symporter activity	1/96	77/14323	0.01298701	1.93763528	0.67766276	0.40501182	0.58321702	0.57033244	Slc6a6	1
GO:0035254	glutamate receptor binding	1/96	78/14323	0.01282051	1.9127938	0.66400224	0.40902129	0.58380131	0.57090381	Myo5b	1
GO:0043178	alcohol binding	1/96	78/14323	0.01282051	1.9127938	0.66400224	0.40902129	0.58380131	0.57090381	Adap2	1
GO:0003727	single-stranded RNA binding	1/96	83/14323	0.01204819	1.79756526	0.59859222	0.42867105	0.60650401	0.59310496	Rbmx	1
GO:0004252	serine-type endopeptidase activity	1/96	83/14323	0.01204819	1.79756526	0.59859222	0.42867105	0.60650401	0.59310496	Tmprss5	1
GO:0016298	lipase activity	1/96	85/14323	0.01176471	1.75526961	0.57367743	0.43634866	0.61205281	0.59853118	Pnpla7	1
GO:0005496	steroid binding	1/96	86/14323	0.01162791	1.7348595	0.56146809	0.44014909	0.61205281	0.59853118	Cyp3a13	1
GO:0015171	amino acid transmembrane transporter activity	1/96	86/14323	0.01162791	1.7348595	0.56146809	0.44014909	0.61205281	0.59853118	Slc6a6	1
GO:0072341	modified amino acid binding	1/96	86/14323	0.01162791	1.7348595	0.56146809	0.44014909	0.61205281	0.59853118	Trem2	1
GO:0004725	protein tyrosine phosphatase activity	1/96	87/14323	0.01149425	1.71491858	0.54941816	0.44392416	0.61466422	0.6010849	Tns2	1
GO:1901681	sulfur compound binding	2/96	225/14323	0.00888889	1.3262037	0.4051186	0.44676932	0.61597132	0.60236311	Slc6a6/Agrn	2
GO:0004866	endopeptidase inhibitor activity	1/96	90/14323	0.01111111	1.65775463	0.51418302	0.45509889	0.62216051	0.60841557	Serpine1	1
GO:0008170	N-methyltransferase activity	1/96	90/14323	0.01111111	1.65775463	0.51418302	0.45509889	0.62216051	0.60841557	Inmt	1
GO:0004930	G protein-coupled receptor activity	2/96	231/14323	0.00865801	1.29175685	0.36721594	0.46026255	0.62657591	0.61273343	Fzd4/Celsr3	2
GO:0008083	growth factor activity	1/96	92/14323	0.01086957	1.62171649	0.49141609	0.46242495	0.62688571	0.61303638	Gdf10	1
GO:0008236	serine-type peptidase activity	1/96	95/14323	0.01052632	1.57050439	0.45827867	0.47323166	0.63621186	0.6221565	Tmprss5	1
GO:0030414	peptidase inhibitor activity	1/96	95/14323	0.01052632	1.57050439	0.45827867	0.47323166	0.63621186	0.6221565	Serpine1	1
GO:0031490	chromatin DNA binding	1/96	96/14323	0.01041667	1.55414497	0.44748979	0.4767859	0.63834146	0.62423905	Per1	1
GO:1902936	phosphatidylinositol bisphosphate binding	1/96	97/14323	0.01030928	1.53812285	0.4368242	0.48031641	0.64042188	0.62627351	Adap2	1
GO:0017171	serine hydrolase activity	1/96	99/14323	0.01010101	1.50704966	0.41585125	0.48730686	0.6470796	0.63278414	Tmprss5	1
GO:0005125	cytokine activity	1/96	102/14323	0.00980392	1.46272467	0.38524629	0.49761841	0.65807496	0.64353659	Gdf10	1
GO:0140678	molecular function inhibitor activity	3/96	399/14323	0.0075188	1.12178885	0.20267153	0.50295232	0.66161389	0.64699734	Serpine1/Inca1/Agrn	3
GO:0008013	beta-catenin binding	1/96	104/14323	0.00961538	1.43459535	0.36538272	0.50437849	0.66161389	0.64699734	Trem2	1
GO:0015293	symporter activity	1/96	106/14323	0.00943396	1.40752752	0.34592876	0.51104854	0.66594487	0.65123264	Slc6a6	1
GO:0070851	growth factor receptor binding	1/96	107/14323	0.00934579	1.39437305	0.33634982	0.51435017	0.66594487	0.65123264	Lyn	1
GO:1990782	protein tyrosine kinase binding	1/96	108/14323	0.00925926	1.38146219	0.32686674	0.51762974	0.66594487	0.65123264	Trem2	1
GO:0008094	ATP-dependent activity, acting on DNA	1/96	109/14323	0.00917431	1.36878823	0.31747748	0.5208874	0.66594487	0.65123264	Xrcc3	1
GO:0030165	PDZ domain binding	1/96	109/14323	0.00917431	1.36878823	0.31747748	0.5208874	0.66594487	0.65123264	Fzd4	1
GO:0061135	endopeptidase regulator activity	1/96	109/14323	0.00917431	1.36878823	0.31747748	0.5208874	0.66594487	0.65123264	Serpine1	1
GO:0000149	SNARE binding	1/96	110/14323	0.00909091	1.3563447	0.30818007	0.52412328	0.66594487	0.65123264	Abl1	1
GO:0005506	iron ion binding	1/96	110/14323	0.00909091	1.3563447	0.30818007	0.52412328	0.66594487	0.65123264	Cyp3a13	1
GO:0008194	UDP-glycosyltransferase activity	1/96	114/14323	0.00877193	1.30875365	0.27187142	0.53685194	0.67945324	0.66444257	Galnt15	1
GO:0015399	primary active transmembrane transporter activity	1/96	115/14323	0.00869565	1.29737319	0.26300563	0.5399811	0.68075438	0.66571497	ND5	1
GO:0015078	proton transmembrane transporter activity	1/96	118/14323	0.00847458	1.26438912	0.23688892	0.54924359	0.68708465	0.67190539	ND5	1
GO:0015081	sodium ion transmembrane transporter activity	1/96	118/14323	0.00847458	1.26438912	0.23688892	0.54924359	0.68708465	0.67190539	Slc6a6	1
GO:0042826	histone deacetylase binding	1/96	119/14323	0.00840336	1.25376401	0.2283384	0.55228989	0.68823816	0.67303342	Bhlhe41	1
GO:0005201	extracellular matrix structural constituent	1/96	121/14323	0.00826446	1.23304063	0.21146104	0.55832149	0.69308874	0.67777684	Agrn	1
GO:0019838	growth factor binding	1/96	124/14323	0.00806452	1.20320901	0.1866834	0.56721843	0.69800191	0.68258147	Agrn	1
GO:0031267	small GTPase binding	2/96	284/14323	0.00704225	1.05068955	0.07087455	0.57066728	0.69800191	0.68258147	Rab34/Myo5b	2
GO:0048018	receptor ligand activity	2/96	284/14323	0.00704225	1.05068955	0.07087455	0.57066728	0.69800191	0.68258147	Gdf10/Agrn	2
GO:0004857	enzyme inhibitor activity	2/96	285/14323	0.00701754	1.04700292	0.06583782	0.57258944	0.69800191	0.68258147	Serpine1/Inca1	2
GO:0019209	kinase activator activity	1/96	126/14323	0.00793651	1.18411045	0.17050857	0.57305095	0.69800191	0.68258147	Trem2	1
GO:0030546	signaling receptor activator activity	2/96	289/14323	0.00692042	1.03251153	0.04586448	0.58021655	0.704083	0.68852821	Gdf10/Agrn	2
GO:0002020	protease binding	1/96	130/14323	0.00769231	1.14767628	0.13893962	0.58448365	0.70661456	0.69100384	Serpine1	1
GO:0099106	ion channel regulator activity	1/96	138/14323	0.00724638	1.08114432	0.0786799	0.60644788	0.7304428	0.71430566	Clcn2	1
GO:0005244	voltage-gated monoatomic ion channel activity	1/96	139/14323	0.00719424	1.07336631	0.07139791	0.60911133	0.73093359	0.71478561	Clcn2	1
GO:0022832	voltage-gated channel activity	1/96	140/14323	0.00714286	1.0656994	0.06416854	0.61175694	0.73139944	0.71524116	Clcn2	1
GO:0031406	carboxylic acid binding	1/96	141/14323	0.0070922	1.05814125	0.05699096	0.61438482	0.73184075	0.71567272	Agrn	1
GO:0003712	transcription coregulator activity	3/96	473/14323	0.00634249	0.946287	-0.0975811	0.61882971	0.73192167	0.71575186	Bhlhe41/Rere/Eid1	3
GO:0052689	carboxylic ester hydrolase activity	1/96	143/14323	0.00699301	1.04334207	0.04278784	0.61958791	0.73192167	0.71575186	Pnpla7	1
GO:0046943	carboxylic acid transmembrane transporter activity	1/96	144/14323	0.00694444	1.03609664	0.0357607	0.62216333	0.73192167	0.71575186	Slc6a6	1
GO:0005342	organic acid transmembrane transporter activity	1/96	145/14323	0.00689655	1.02895115	0.02878214	0.62472151	0.73192167	0.71575186	Slc6a6	1
GO:0061134	peptidase regulator activity	1/96	146/14323	0.00684932	1.02190354	0.0218514	0.62726254	0.73192167	0.71575186	Serpine1	1
GO:0003729	mRNA binding	2/96	316/14323	0.00632911	0.94429061	-0.0822584	0.62911454	0.73192167	0.71575186	Srrm2/Rbmx	2
GO:0016247	channel regulator activity	1/96	148/14323	0.00675676	1.00809403	0.00813046	0.63229363	0.73192167	0.71575186	Clcn2	1
GO:0030545	signaling receptor regulator activity	2/96	319/14323	0.00626959	0.93541014	-0.0958329	0.63426982	0.73192167	0.71575186	Gdf10/Agrn	2
GO:0008757	S-adenosylmethionine-dependent methyltransferase activity	1/96	149/14323	0.00671141	1.0013283	0.00133882	0.63478391	0.73192167	0.71575186	Inmt	1
GO:0141108	transporter regulator activity	1/96	150/14323	0.00666667	0.99465278	-0.0054078	0.63725751	0.7321682	0.71599295	Clcn2	1
GO:0051020	GTPase binding	2/96	324/14323	0.00617284	0.92097479	-0.1181872	0.64273911	0.73487941	0.71864426	Rab34/Myo5b	2
GO:0140657	ATP-dependent activity	3/96	491/14323	0.00610998	0.91159623	-0.1637381	0.64415356	0.73487941	0.71864426	Myo5b/Nav3/Xrcc3	3
GO:0004721	phosphoprotein phosphatase activity	1/96	155/14323	0.00645161	0.9625672	-0.0384897	0.64937897	0.73824136	0.72193193	Tns2	1
GO:0016853	isomerase activity	1/96	157/14323	0.00636943	0.9503052	-0.0514302	0.65411454	0.74102487	0.72465395	Lss	1
GO:0022804	active transmembrane transporter activity	2/96	336/14323	0.00595238	0.88808284	-0.1705237	0.66244263	0.74524796	0.72878375	Slc6a6/ND5	2
GO:0061629	RNA polymerase II-specific DNA-binding transcription factor binding	2/96	336/14323	0.00595238	0.88808284	-0.1705237	0.66244263	0.74524796	0.72878375	Bhlhe40/Bhlhe41	2
GO:0016887	ATP hydrolysis activity	2/96	342/14323	0.00584795	0.87250244	-0.1960318	0.67196743	0.75334757	0.73670442	Myo5b/Nav3	2
GO:1901981	phosphatidylinositol phosphate binding	1/96	170/14323	0.00588235	0.8776348	-0.1318375	0.68338961	0.76175472	0.74492583	Adap2	1
GO:0016829	lyase activity	1/96	171/14323	0.00584795	0.87250244	-0.1377754	0.68553719	0.76175472	0.74492583	Tha1	1
GO:0005126	cytokine receptor binding	1/96	172/14323	0.00581395	0.86742975	-0.1436804	0.68767034	0.76175472	0.74492583	Lyn	1
GO:0015631	tubulin binding	2/96	353/14323	0.00566572	0.84531398	-0.2417247	0.68887078	0.76175472	0.74492583	Nav3/Lyn	2
GO:0005516	calmodulin binding	1/96	178/14323	0.00561798	0.83819054	-0.1784404	0.70017186	0.77123724	0.75419886	Myo5b	1
GO:0008168	methyltransferase activity	1/96	179/14323	0.00558659	0.83350791	-0.1841259	0.70220675	0.77123724	0.75419886	Inmt	1
GO:0016741	transferase activity, transferring one-carbon groups	1/96	188/14323	0.00531915	0.79360594	-0.2339963	0.71991648	0.78801669	0.77060761	Inmt	1
GO:0005085	guanyl-nucleotide exchange factor activity	1/96	197/14323	0.00507614	0.75734983	-0.2816987	0.73658334	0.80354546	0.78579332	Net1	1
GO:0008514	organic anion transmembrane transporter activity	1/96	204/14323	0.00490196	0.73136234	-0.3174394	0.7488645	0.81420167	0.79621411	Slc6a6	1
GO:0003779	actin binding	2/96	402/14323	0.00497512	0.74227819	-0.4305363	0.75572926	0.81891732	0.80082558	Myo5b/Abl1	2
GO:0019887	protein kinase regulator activity	1/96	210/14323	0.0047619	0.71046627	-0.3472002	0.75893902	0.81965414	0.80154612	Inca1	1
GO:0030695	GTPase regulator activity	2/96	414/14323	0.00483092	0.72076288	-0.473594	0.77011289	0.82523982	0.8070084	Net1/Adap2	2
GO:0060589	nucleoside-triphosphatase regulator activity	2/96	414/14323	0.00483092	0.72076288	-0.473594	0.77011289	0.82523982	0.8070084	Net1/Adap2	2
GO:0015291	secondary active transmembrane transporter activity	1/96	218/14323	0.00458716	0.68439411	-0.3857159	0.77175206	0.82523982	0.8070084	Slc6a6	1
GO:0005096	GTPase activator activity	1/96	225/14323	0.00444444	0.66310185	-0.4184002	0.78240909	0.83114933	0.81278735	Adap2	1
GO:0042393	histone binding	1/96	225/14323	0.00444444	0.66310185	-0.4184002	0.78240909	0.83114933	0.81278735	Ncapd3	1
GO:0022836	gated channel activity	1/96	231/14323	0.004329	0.64587843	-0.4457105	0.79115072	0.837689	0.81918255	Clcn2	1
GO:0016791	phosphatase activity	1/96	239/14323	0.0041841	0.62425907	-0.4811774	0.80226777	0.84669302	0.82798765	Tns2	1
GO:0022890	inorganic cation transmembrane transporter activity	2/96	446/14323	0.0044843	0.66904895	-0.5832627	0.80495352	0.84676929	0.82806223	Slc6a6/ND5	2
GO:0008017	microtubule binding	1/96	248/14323	0.00403226	0.6016045	-0.5198725	0.81407564	0.85359387	0.83473605	Nav3	1
GO:0004175	endopeptidase activity	1/96	255/14323	0.00392157	0.58508987	-0.5491472	0.82277468	0.85692318	0.83799181	Tmprss5	1
GO:0005509	calcium ion binding	2/96	466/14323	0.00429185	0.64033441	-0.6483926	0.82430064	0.85692318	0.83799181	Agrn/Celsr3	2
GO:0003713	transcription coactivator activity	1/96	257/14323	0.00389105	0.58053664	-0.557386	0.82518529	0.85692318	0.83799181	Rere	1
GO:0008324	monoatomic cation transmembrane transporter activity	2/96	477/14323	0.00419287	0.62556778	-0.683205	0.83420033	0.86108415	0.84206084	Slc6a6/ND5	2
GO:0035091	phosphatidylinositol binding	1/96	265/14323	0.00377358	0.56301101	-0.5898104	0.83450747	0.86108415	0.84206084	Adap2	1
GO:0061630	ubiquitin protein ligase activity	1/96	297/14323	0.003367	0.50234989	-0.7118954	0.86712481	0.8918998	0.87219572	Peli1	1
GO:0061659	ubiquitin-like protein ligase activity	1/96	314/14323	0.00318471	0.4751526	-0.7724584	0.88177518	0.90409861	0.88412502	Peli1	1
GO:0005216	monoatomic ion channel activity	1/96	325/14323	0.00307692	0.45907051	-0.8102707	0.8903908	0.91005242	0.8899473	Clcn2	1
GO:0046873	metal ion transmembrane transporter activity	1/96	346/14323	0.00289017	0.43120785	-0.8797625	0.9051496	0.92222789	0.90185379	Slc6a6	1
GO:0015267	channel activity	1/96	362/14323	0.00276243	0.41214894	-0.9305594	0.91505809	0.92649632	0.90602792	Clcn2	1
GO:0022803	passive transmembrane transporter activity	1/96	362/14323	0.00276243	0.41214894	-0.9305594	0.91505809	0.92649632	0.90602792	Clcn2	1
GO:0004842	ubiquitin-protein transferase activity	1/96	376/14323	0.00265957	0.39680297	-0.973629	0.92288316	0.93150823	0.9109291	Peli1	1
GO:0019787	ubiquitin-like protein transferase activity	1/96	401/14323	0.00249377	0.37206463	-1.0476521	0.93512272	0.94093094	0.92014364	Peli1	1
GO:0016755	aminoacyltransferase activity	1/96	411/14323	0.00243309	0.36301196	-1.076312	0.93946144	0.94152184	0.92072148	Peli1	1
GO:0008233	peptidase activity	1/96	416/14323	0.00240385	0.35864884	-1.0904521	0.94152184	0.94152184	0.92072148	Tmprss5	1

ID	Description	GeneRatio	BgRatio	RichFactor	FoldEnrichm	zScore	pvalue	p.adjust	qvalue	geneID	Count
GO:0050660	flavin adenine dinucleotide binding	4/46	80/14323	0.05	15.5684783	7.41687086	0.0001232	0.01971189	0.01530265	Fmo2/Fmo1/Xdh/Prodh	4
GO:0061459	L-arginine transmembrane transporter activity	2/46	13/14323	0.15384615	47.90301	9.60317504	0.00076956	0.03765425	0.02923159	Slc7a2/Slc25a15	2
GO:0015179	L-amino acid transmembrane transporter activity	3/46	60/14323	0.05	15.5684783	6.4186936	0.00093332	0.03765425	0.02923159	Slc1a5/Slc7a2/Slc25a15	3
GO:0004497	monooxygenase activity	3/46	63/14323	0.04761905	14.8271222	6.24316168	0.00107582	0.03765425	0.02923159	Fmo2/Fmo1/Cyp2f2	3
GO:0015174	basic amino acid transmembrane transporter activity	2/46	16/14323	0.125	38.9211957	8.61449842	0.0011767	0.03765425	0.02923159	Slc7a2/Slc25a15	2
GO:0015101	organic cation transmembrane transporter activity	3/46	70/14323	0.04285714	13.3444099	5.87663184	0.0014599	0.03893061	0.03022245	Slc1a5/Slc7a2/Slc25a15	3
GO:0016709	oxidoreductase activity, acting on paired donors, with incorporation or reduction of molecular oxygen, NA	2/46	23/14323	0.08695652	27.0756144	7.10383261	0.00244564	0.05266738	0.04088652	Fmo2/Fmo1	2
GO:0015171	amino acid transmembrane transporter activity	3/46	86/14323	0.03488372	10.861729	5.20661324	0.00263337	0.05266738	0.04088652	Slc1a5/Slc7a2/Slc25a15	3
GO:0071949	FAD binding	2/46	39/14323	0.05128205	15.96767	5.31280472	0.00693308	0.1232547	0.09568457	Xdh/Prodh	2
GO:0016705	oxidoreductase activity, acting on paired donors, with incorporation or reduction of molecular oxygen	3/46	134/14323	0.02238806	6.97096042	3.94169456	0.00906462	0.12830889	0.09960822	Fmo2/Fmo1/Cyp2f2	3
GO:0050661	NADP binding	2/46	45/14323	0.04444444	13.8386473	4.89614317	0.00915048	0.12830889	0.09960822	Fmo2/Fmo1	2
GO:0046943	carboxylic acid transmembrane transporter activity	3/46	144/14323	0.02083333	6.48686594	3.75616849	0.0110192	0.12830889	0.09960822	Slc1a5/Slc7a2/Slc25a15	3
GO:0016597	amino acid binding	2/46	50/14323	0.04	12.4547826	4.60549687	0.01120816	0.12830889	0.09960822	Prodh/Sesn1	2
GO:0005342	organic acid transmembrane transporter activity	3/46	145/14323	0.02068966	6.44212894	3.73858804	0.01122703	0.12830889	0.09960822	Slc1a5/Slc7a2/Slc25a15	3
GO:0008514	organic anion transmembrane transporter activity	3/46	204/14323	0.01470588	4.57896419	2.92235343	0.02760177	0.18391686	0.14277756	Slc1a5/Slc7a2/Slc25a15	3
GO:0008392	arachidonate epoxygenase activity	1/46	10/14323	0.1	31.1369565	5.41123415	0.03166578	0.18391686	0.14277756	Cyp2f2	1
GO:0015183	L-aspartate transmembrane transporter activity	1/46	10/14323	0.1	31.1369565	5.41123415	0.03166578	0.18391686	0.14277756	Slc1a5	1
GO:0042609	CD4 receptor binding	1/46	10/14323	0.1	31.1369565	5.41123415	0.03166578	0.18391686	0.14277756	Plscr4	1
GO:0048185	activin binding	1/46	10/14323	0.1	31.1369565	5.41123415	0.03166578	0.18391686	0.14277756	Fstl3	1
GO:0180030	inositol phosphate kinase activity	1/46	10/14323	0.1	31.1369565	5.41123415	0.03166578	0.18391686	0.14277756	Ip6k2	1
GO:0008391	arachidonate monooxygenase activity	1/46	11/14323	0.09090909	28.3063241	5.14246909	0.03477788	0.18391686	0.14277756	Cyp2f2	1
GO:0052650	all-trans-retinol dehydrogenase (NADP+) activity	1/46	11/14323	0.09090909	28.3063241	5.14246909	0.03477788	0.18391686	0.14277756	Rdh10	1
GO:0097371	MDM2/MDM4 family protein binding	1/46	11/14323	0.09090909	28.3063241	5.14246909	0.03477788	0.18391686	0.14277756	Bcl2l1	1
GO:0106018	phosphatidylinositol-3,5-bisphosphate phosphatase activity	1/46	11/14323	0.09090909	28.3063241	5.14246909	0.03477788	0.18391686	0.14277756	Mtmr2	1
GO:0004022	alcohol dehydrogenase (NAD+) activity	1/46	12/14323	0.08333333	25.9474638	4.90731915	0.03788018	0.18391686	0.14277756	Rdh10	1
GO:0004438	phosphatidylinositol-3-phosphate phosphatase activity	1/46	12/14323	0.08333333	25.9474638	4.90731915	0.03788018	0.18391686	0.14277756	Mtmr2	1
GO:0016661	oxidoreductase activity, acting on other nitrogenous compounds as donors	1/46	12/14323	0.08333333	25.9474638	4.90731915	0.03788018	0.18391686	0.14277756	Xdh	1
GO:0022841	potassium ion leak channel activity	1/46	12/14323	0.08333333	25.9474638	4.90731915	0.03788018	0.18391686	0.14277756	Kcnk4	1
GO:0070513	death domain binding	1/46	12/14323	0.08333333	25.9474638	4.90731915	0.03788018	0.18391686	0.14277756	Bcl2l1	1
GO:0015556	C4-dicarboxylate transmembrane transporter activity	1/46	13/14323	0.07692308	23.951505	4.69921482	0.04097274	0.18391686	0.14277756	Slc1a5	1
GO:0019825	oxygen binding	1/46	13/14323	0.07692308	23.951505	4.69921482	0.04097274	0.18391686	0.14277756	Cyp2f2	1
GO:0016500	protein-hormone receptor activity	1/46	14/14323	0.07142857	22.2406832	4.51325767	0.04405556	0.18391686	0.14277756	Mchr1	1
GO:0052744	phosphatidylinositol monophosphate phosphatase activity	1/46	14/14323	0.07142857	22.2406832	4.51325767	0.04405556	0.18391686	0.14277756	Mtmr2	1
GO:0015297	antiporter activity	2/46	107/14323	0.01869159	5.81999187	2.84061096	0.0462503	0.18391686	0.14277756	Slc1a5/Slc25a15	2
GO:0008381	mechanosensitive monoatomic ion channel activity	1/46	15/14323	0.06666667	20.757971	4.34571037	0.04712869	0.18391686	0.14277756	Kcnk4	1
GO:0016530	metallochaperone activity	1/46	15/14323	0.06666667	20.757971	4.34571037	0.04712869	0.18391686	0.14277756	Pcbp2	1
GO:0017128	phospholipid scramblase activity	1/46	15/14323	0.06666667	20.757971	4.34571037	0.04712869	0.18391686	0.14277756	Plscr4	1
GO:0022840	leak channel activity	1/46	15/14323	0.06666667	20.757971	4.34571037	0.04712869	0.18391686	0.14277756	Kcnk4	1
GO:0022842	narrow pore channel activity	1/46	15/14323	0.06666667	20.757971	4.34571037	0.04712869	0.18391686	0.14277756	Kcnk4	1
GO:0051400	BH domain binding	1/46	15/14323	0.06666667	20.757971	4.34571037	0.04712869	0.18391686	0.14277756	Bcl2l1	1
GO:1990226	histone methyltransferase binding	1/46	15/14323	0.06666667	20.757971	4.34571037	0.04712869	0.18391686	0.14277756	Per2	1
GO:0005506	iron ion binding	2/46	110/14323	0.01818182	5.66126482	2.78560487	0.04859964	0.18514147	0.14372824	Xdh/Cyp2f2	2
GO:0004713	protein tyrosine kinase activity	2/46	115/14323	0.0173913	5.41512287	2.69828309	0.05261004	0.18931908	0.14697139	Mertk/Tesk2	2
GO:0015645	fatty acid ligase activity	1/46	17/14323	0.05882353	18.3158568	4.05482024	0.05324599	0.18931908	0.14697139	Acsf3	1
GO:0120515	fatty acid-CoA ligase activity	1/46	17/14323	0.05882353	18.3158568	4.05482024	0.05324599	0.18931908	0.14697139	Acsf3	1
GO:0016405	CoA-ligase activity	1/46	18/14323	0.05555556	17.2983092	3.92732773	0.05629022	0.19371385	0.15038312	Acsf3	1
GO:0010314	phosphatidylinositol-5-phosphate binding	1/46	19/14323	0.05263158	16.3878719	3.80968336	0.05932487	0.19371385	0.15038312	Phlda3	1
GO:0015172	acidic amino acid transmembrane transporter activity	1/46	19/14323	0.05263158	16.3878719	3.80968336	0.05932487	0.19371385	0.15038312	Slc1a5	1
GO:0043027	cysteine-type endopeptidase inhibitor activity involved in apoptotic process	1/46	19/14323	0.05263158	16.3878719	3.80968336	0.05932487	0.19371385	0.15038312	Bcl2l1	1
GO:1990380	K48-linked deubiquitinase activity	1/46	20/14323	0.05	15.5684783	3.70064895	0.06234997	0.19732997	0.15319037	Otud7b	1
GO:0005044	scavenger receptor activity	1/46	21/14323	0.04761905	14.8271222	3.59919462	0.06536555	0.19732997	0.15319037	Cd163	1
GO:0015271	outward rectifier potassium channel activity	1/46	21/14323	0.04761905	14.8271222	3.59919462	0.06536555	0.19732997	0.15319037	Kcnk4	1
GO:0042923	neuropeptide binding	1/46	21/14323	0.04761905	14.8271222	3.59919462	0.06536555	0.19732997	0.15319037	Mchr1	1
GO:0005310	dicarboxylic acid transmembrane transporter activity	1/46	22/14323	0.04545455	14.1531621	3.50445574	0.06837165	0.19889934	0.1544087	Slc1a5	1
GO:0016878	acid-thiol ligase activity	1/46	22/14323	0.04545455	14.1531621	3.50445574	0.06837165	0.19889934	0.1544087	Acsf3	1
GO:0008106	alcohol dehydrogenase (NADP+) activity	1/46	23/14323	0.04347826	13.5378072	3.41570029	0.07136828	0.20075433	0.15584876	Rdh10	1
GO:0016645	oxidoreductase activity, acting on the CH-NH group of donors	1/46	24/14323	0.04166667	12.9737319	3.3323038	0.07435549	0.20075433	0.15584876	Prodh	1
GO:0016712	oxidoreductase activity, acting on paired donors, with incorporation or reduction of molecular oxygen, red	1/46	24/14323	0.04166667	12.9737319	3.3323038	0.07435549	0.20075433	0.15584876	Cyp2f2	1
GO:0034593	phosphatidylinositol bisphosphate phosphatase activity	1/46	24/14323	0.04166667	12.9737319	3.3323038	0.07435549	0.20075433	0.15584876	Mtmr2	1
GO:0004033	aldo-keto reductase (NADPH) activity	1/46	25/14323	0.04	12.4547826	3.25372976	0.0773333	0.20075433	0.15584876	Rdh10	1
GO:0070530	K63-linked polyubiquitin modification-dependent protein binding	1/46	25/14323	0.04	12.4547826	3.25372976	0.0773333	0.20075433	0.15584876	Otud7b	1
GO:0051537	2 iron, 2 sulfur cluster binding	1/46	26/14323	0.03846154	11.9757525	3.17951437	0.08030173	0.20075433	0.15584876	Xdh	1
GO:0052866	phosphatidylinositol phosphate phosphatase activity	1/46	26/14323	0.03846154	11.9757525	3.17951437	0.08030173	0.20075433	0.15584876	Mtmr2	1
GO:0106222	lncRNA binding	1/46	26/14323	0.03846154	11.9757525	3.17951437	0.08030173	0.20075433	0.15584876	Pcbp2	1
GO:0043325	phosphatidylinositol-3,4-bisphosphate binding	1/46	27/14323	0.03703704	11.5322061	3.10925422	0.08326082	0.20494972	0.1591057	Phlda3	1
GO:0080025	phosphatidylinositol-3,5-bisphosphate binding	1/46	28/14323	0.03571429	11.1203416	3.04259662	0.0862106	0.20899539	0.16224642	Phlda3	1
GO:0016776	phosphotransferase activity, phosphate group as acceptor	1/46	30/14323	0.03333333	10.3789855	2.91888555	0.09208232	0.21989808	0.17071035	Ip6k2	1
GO:0016877	ligase activity, forming carbon-sulfur bonds	1/46	32/14323	0.03125	9.73029891	2.80630625	0.09791713	0.22705422	0.17626577	Acsf3	1
GO:0018455	alcohol dehydrogenase [NAD(P)+] activity	1/46	32/14323	0.03125	9.73029891	2.80630625	0.09791713	0.22705422	0.17626577	Rdh10	1
GO:0001968	fibronectin binding	1/46	33/14323	0.03030303	9.43544137	2.75366394	0.10082076	0.22720172	0.17638028	Fstl3	1
GO:0043028	cysteine-type endopeptidase regulator activity involved in apoptotic process	1/46	33/14323	0.03030303	9.43544137	2.75366394	0.10082076	0.22720172	0.17638028	Bcl2l1	1
GO:0015175	neutral L-amino acid transmembrane transporter activity	1/46	36/14323	0.02777778	8.64915459	2.60829337	0.10947691	0.2376652	0.18450325	Slc1a5	1
GO:0015267	channel activity	3/46	362/14323	0.00828729	2.58041076	1.72873439	0.10992015	0.2376652	0.18450325	Slc1a5/Kcnk4/Bcl2l1	3
GO:0022803	passive transmembrane transporter activity	3/46	362/14323	0.00828729	2.58041076	1.72873439	0.10992015	0.2376652	0.18450325	Slc1a5/Kcnk4/Bcl2l1	3
GO:0004712	protein serine/threonine/tyrosine kinase activity	1/46	38/14323	0.02631579	8.19393593	2.52046408	0.11520233	0.23938147	0.18583562	Tesk2	1
GO:0036002	pre-mRNA binding	1/46	38/14323	0.02631579	8.19393593	2.52046408	0.11520233	0.23938147	0.18583562	Per2	1
GO:0140303	intramembrane lipid transporter activity	1/46	38/14323	0.02631579	8.19393593	2.52046408	0.11520233	0.23938147	0.18583562	Plscr4	1
GO:0016684	oxidoreductase activity, acting on peroxide as acceptor	1/46	43/14323	0.02325581	7.24115268	2.32646864	0.1293588	0.26432678	0.20520105	Sesn1	1
GO:0005547	phosphatidylinositol-3,4,5-trisphosphate binding	1/46	44/14323	0.02272727	7.07658103	2.29138996	0.13216339	0.26432678	0.20520105	Phlda3	1
GO:0032266	phosphatidylinositol-3-phosphate binding	1/46	44/14323	0.02272727	7.07658103	2.29138996	0.13216339	0.26432678	0.20520105	Phlda3	1
GO:0030276	clathrin binding	1/46	48/14323	0.02083333	6.48686594	2.16132054	0.14329362	0.28304913	0.21973551	Bcl2l1	1
GO:0004714	transmembrane receptor protein tyrosine kinase activity	1/46	50/14323	0.02	6.2273913	2.101719	0.14880625	0.29035366	0.22540613	Mertk	1
GO:0015291	secondary active transmembrane transporter activity	2/46	218/14323	0.00917431	2.85660152	1.56791557	0.15486151	0.29852821	0.23175216	Slc1a5/Slc25a15	2
GO:0038024	cargo receptor activity	1/46	54/14323	0.01851852	5.76610306	1.99170866	0.1597276	0.30024863	0.23308775	Cd163	1
GO:0001227	DNA-binding transcription repressor activity, RNA polymerase II-specific	2/46	224/14323	0.00892857	2.7800854	1.52416844	0.16162047	0.30024863	0.23308775	Mynn/Mxi1	2
GO:0005548	phospholipid transporter activity	1/46	55/14323	0.01818182	5.66126482	1.96592001	0.16243645	0.30024863	0.23308775	Plscr4	1
GO:0001222	transcription corepressor binding	1/46	56/14323	0.01785714	5.56017081	1.94075656	0.16513675	0.30024863	0.23308775	Per2	1
GO:0031593	polyubiquitin modification-dependent protein binding	1/46	56/14323	0.01785714	5.56017081	1.94075656	0.16513675	0.30024863	0.23308775	Otud7b	1
GO:0001217	DNA-binding transcription repressor activity	2/46	230/14323	0.00869565	2.70756144	1.48183825	0.16843435	0.30146672	0.23403337	Mynn/Mxi1	2
GO:0022836	gated channel activity	2/46	231/14323	0.00865801	2.69584039	1.47491475	0.16957503	0.30146672	0.23403337	Slc1a5/Kcnk4	2
GO:0070063	RNA polymerase binding	1/46	62/14323	0.01612903	5.02208976	1.80150256	0.18116062	0.31852417	0.24727535	Per2	1
GO:0042562	hormone binding	1/46	64/14323	0.015625	4.86514946	1.75903311	0.18643475	0.32423435	0.25170825	Mchr1	1
GO:0008017	microtubule binding	2/46	248/14323	0.00806452	2.51104488	1.36251494	0.18916202	0.32544004	0.25264424	Cfap157/Maco1	2
GO:0019199	transmembrane receptor protein kinase activity	1/46	66/14323	0.01515152	4.71772069	1.71829182	0.19167565	0.32625642	0.25327801	Mertk	1
GO:0051536	iron-sulfur cluster binding	1/46	69/14323	0.01449275	4.51260239	1.66015039	0.19947511	0.33595809	0.26080957	Xdh	1
GO:0051540	metal cluster binding	1/46	70/14323	0.01428571	4.44813665	1.64150654	0.20205854	0.33676423	0.26143539	Xdh	1
GO:0005546	phosphatidylinositol-4,5-bisphosphate binding	1/46	72/14323	0.01388889	4.32457729	1.60524854	0.20720095	0.34177476	0.26532514	Phlda3	1
GO:0005249	voltage-gated potassium channel activity	1/46	78/14323	0.01282051	3.9919175	1.50393117	0.22243441	0.35948996	0.27907773	Kcnk4	1
GO:0097110	scaffold protein binding	1/46	78/14323	0.01282051	3.9919175	1.50393117	0.22243441	0.35948996	0.27907773	Cd163	1
GO:0004843	cysteine-type deubiquitinase activity	1/46	79/14323	0.01265823	3.9413869	1.48803103	0.22494533	0.35991253	0.27940578	Otud7b	1
GO:0020037	heme binding	1/46	84/14323	0.01190476	3.70678054	1.41226181	0.23738142	0.37604977	0.29193338	Cyp2f2	1
GO:0101005	deubiquitinase activity	1/46	87/14323	0.01149425	3.57896052	1.36953331	0.24474915	0.38392023	0.29804334	Otud7b	1
GO:0046906	tetrapyrrole binding	1/46	91/14323	0.01098901	3.42164357	1.31540722	0.25446456	0.39148393	0.30391516	Cyp2f2	1
GO:1990756	ubiquitin-like ligase-substrate adaptor activity	1/46	91/14323	0.01098901	3.42164357	1.31540722	0.25446456	0.39148393	0.30391516	Herpud1	1
GO:0140104	molecular carrier activity	1/46	94/14323	0.0106383	3.31244218	1.27676183	0.26167077	0.39805234	0.30901432	Pcbp2	1
GO:0016616	oxidoreductase activity, acting on the CH-OH group of donors, NAD or NADP as acceptor	1/46	95/14323	0.01052632	3.27757437	1.26422598	0.26405767	0.39805234	0.30901432	Rdh10	1
GO:0031490	chromatin DNA binding	1/46	96/14323	0.01041667	3.24343297	1.25185584	0.26643702	0.39805234	0.30901432	Pcbp2	1
GO:1902936	phosphatidylinositol bisphosphate binding	1/46	97/14323	0.01030928	3.20999552	1.23964727	0.26880884	0.39805234	0.30901432	Phlda3	1
GO:0019783	ubiquitin-like protein peptidase activity	1/46	98/14323	0.01020408	3.17724046	1.22759629	0.27117316	0.39805234	0.30901432	Otud7b	1
GO:0005267	potassium channel activity	1/46	101/14323	0.00990099	3.08286698	1.1923511	0.27822133	0.4007941	0.31114279	Kcnk4	1
GO:0140767	enzyme-substrate adaptor activity	1/46	101/14323	0.00990099	3.08286698	1.1923511	0.27822133	0.4007941	0.31114279	Herpud1	1
GO:0016614	oxidoreductase activity, acting on CH-OH group of donors	1/46	102/14323	0.00980392	3.0526428	1.18089333	0.28055587	0.4007941	0.31114279	Rdh10	1
GO:0015293	symporter activity	1/46	106/14323	0.00943396	2.93744873	1.13642673	0.28982038	0.40859257	0.31719686	Slc1a5	1
GO:0022804	active transmembrane transporter activity	2/46	336/14323	0.00595238	1.85339027	0.8984984	0.29367591	0.40859257	0.31719686	Slc1a5/Slc25a15	2
GO:0061629	RNA polymerase II-specific DNA-binding transcription factor binding	2/46	336/14323	0.00595238	1.85339027	0.8984984	0.29367591	0.40859257	0.31719686	Per2/Tead3	2
GO:0015631	tubulin binding	2/46	353/14323	0.00566572	1.76413351	0.82512675	0.31395013	0.429084	0.33310469	Cfap157/Maco1	2
GO:0022843	voltage-gated monoatomic cation channel activity	1/46	117/14323	0.00854701	2.66127834	1.02414559	0.31470016	0.429084	0.33310469	Kcnk4	1
GO:0003697	single-stranded DNA binding	1/46	119/14323	0.00840336	2.61655097	1.00512441	0.31913123	0.429084	0.33310469	Pcbp2	1
GO:0042826	histone deacetylase binding	1/46	119/14323	0.00840336	2.61655097	1.00512441	0.31913123	0.429084	0.33310469	Per2	1
GO:0022834	ligand-gated channel activity	1/46	121/14323	0.00826446	2.57330219	0.98648923	0.32353426	0.43137902	0.33488634	Slc1a5	1
GO:0001221	transcription coregulator binding	1/46	123/14323	0.00813008	2.53145988	0.96822497	0.32790944	0.43359926	0.33660995	Per2	1
GO:0005319	lipid transporter activity	1/46	125/14323	0.008	2.49095652	0.95031738	0.33225693	0.43574679	0.33827711	Plscr4	1
GO:0015079	potassium ion transmembrane transporter activity	1/46	131/14323	0.00763359	2.37686691	0.89860345	0.34513499	0.44895608	0.34853169	Kcnk4	1
GO:0005244	voltage-gated monoatomic ion channel activity	1/46	139/14323	0.00719424	2.24006881	0.83390407	0.36192841	0.46014588	0.35721851	Kcnk4	1
GO:0022832	voltage-gated channel activity	1/46	140/14323	0.00714286	2.22406832	0.82612905	0.36399773	0.46014588	0.35721851	Kcnk4	1
GO:0008234	cysteine-type peptidase activity	1/46	141/14323	0.0070922	2.20829479	0.81841954	0.36606049	0.46014588	0.35721851	Otud7b	1
GO:0031406	carboxylic acid binding	1/46	141/14323	0.0070922	2.20829479	0.81841954	0.36606049	0.46014588	0.35721851	Sesn1	1
GO:0016922	nuclear receptor binding	1/46	142/14323	0.00704225	2.19274342	0.81077443	0.3681167	0.46014588	0.35721851	Per2	1
GO:0016874	ligase activity	1/46	149/14323	0.00671141	2.08972863	0.75897514	0.38232863	0.47055831	0.36530185	Acsf3	1
GO:0043177	organic acid binding	1/46	149/14323	0.00671141	2.08972863	0.75897514	0.38232863	0.47055831	0.36530185	Sesn1	1
GO:0044325	transmembrane transporter binding	1/46	162/14323	0.00617284	1.92203435	0.66991533	0.4078973	0.49819518	0.38675678	Herpud1	1
GO:1901981	phosphatidylinositol phosphate binding	1/46	170/14323	0.00588235	1.83158568	0.61911237	0.42311411	0.51286559	0.39814565	Phlda3	1
GO:0140030	modification-dependent protein binding	1/46	184/14323	0.00543478	1.6922259	0.53642583	0.44882813	0.53674296	0.41668203	Otud7b	1
GO:0140297	DNA-binding transcription factor binding	2/46	471/14323	0.00424628	1.32216376	0.40354611	0.44952223	0.53674296	0.41668203	Per2/Tead3	2
GO:0042277	peptide binding	1/46	200/14323	0.005	1.55684783	0.45014281	0.47684411	0.56180541	0.43613841	Mchr1	1
GO:0004672	protein kinase activity	2/46	497/14323	0.00402414	1.25299624	0.32584139	0.4775346	0.56180541	0.43613841	Mertk/Tesk2	2
GO:0030246	carbohydrate binding	1/46	204/14323	0.00490196	1.5263214	0.42976042	0.48362748	0.56482041	0.438479	Lgalsl	1
GO:0004930	G protein-coupled receptor activity	1/46	231/14323	0.004329	1.3479202	0.30259488	0.52721681	0.61126587	0.47453535	Mchr1	1
GO:0016791	phosphatase activity	1/46	239/14323	0.0041841	1.30280153	0.26795155	0.53942598	0.620922	0.48203155	Mtmr2	1
GO:0003713	transcription coactivator activity	1/46	257/14323	0.00389105	1.21155473	0.19425234	0.56577987	0.64201971	0.49841004	Per2	1
GO:0005261	monoatomic cation channel activity	1/46	257/14323	0.00389105	1.21155473	0.19425234	0.56577987	0.64201971	0.49841004	Kcnk4	1
GO:0035091	phosphatidylinositol binding	1/46	265/14323	0.00377358	1.17497949	0.16319641	0.57701368	0.65015626	0.50472657	Phlda3	1
GO:0031625	ubiquitin protein ligase binding	1/46	297/14323	0.003367	1.04838237	0.04782564	0.61917381	0.69278189	0.53781752	Pcbp2	1
GO:0044389	ubiquitin-like protein ligase binding	1/46	313/14323	0.00319489	0.99479094	-0.005289	0.63868336	0.70330159	0.54598413	Pcbp2	1
GO:0003729	mRNA binding	1/46	316/14323	0.00316456	0.98534673	-0.0149509	0.64223095	0.70330159	0.54598413	Pcbp2	1
GO:0042578	phosphoric ester hydrolase activity	1/46	318/14323	0.00314465	0.97914958	-0.0213427	0.64457705	0.70330159	0.54598413	Mtmr2	1
GO:0051020	GTPase binding	1/46	324/14323	0.00308642	0.96101718	-0.0402865	0.65152543	0.70330159	0.54598413	Bcl2l1	1
GO:0005216	monoatomic ion channel activity	1/46	325/14323	0.00307692	0.9580602	-0.0434108	0.6526705	0.70330159	0.54598413	Kcnk4	1
GO:0106310	protein serine kinase activity	1/46	327/14323	0.0030581	0.95220051	-0.0496315	0.6549496	0.70330159	0.54598413	Tesk2	1
GO:0046873	metal ion transmembrane transporter activity	1/46	346/14323	0.00289017	0.89991204	-0.1069732	0.67588419	0.72094313	0.55967954	Kcnk4	1
GO:0001228	DNA-binding transcription activator activity, RNA polymerase II-specific	1/46	357/14323	0.00280112	0.87218366	-0.1388183	0.68743081	0.7284035	0.56547114	Klf15	1
GO:0001216	DNA-binding transcription activator activity	1/46	363/14323	0.00275482	0.8577674	-0.1558016	0.69355833	0.7300614	0.56675819	Klf15	1
GO:0004674	protein serine/threonine kinase activity	1/46	388/14323	0.00257732	0.80249888	-0.2238691	0.7178491	0.75069187	0.58277395	Tesk2	1
GO:0003779	actin binding	1/46	402/14323	0.00248756	0.77455116	-0.2602484	0.73061829	0.75908393	0.58928884	Fam107a	1
GO:0008233	peptidase activity	1/46	416/14323	0.00240385	0.74848453	-0.2954996	0.74282159	0.76678357	0.5952662	Otud7b	1
GO:0005543	phospholipid binding	1/46	428/14323	0.00233645	0.72749898	-0.32488	0.7528498	0.77215364	0.59943506	Phlda3	1
GO:0022890	inorganic cation transmembrane transporter activity	1/46	446/14323	0.00224215	0.69813804	-0.3676125	0.76717874	0.78183821	0.60695335	Kcnk4	1
GO:0003712	transcription coregulator activity	1/46	473/14323	0.00211416	0.65828661	-0.428973	0.78716068	0.79494293	0.61712675	Per2	1
GO:0008324	monoatomic cation transmembrane transporter activity	1/46	477/14323	0.00209644	0.65276638	-0.4378053	0.78997454	0.79494293	0.61712675	Kcnk4	1
GO:0140657	ATP-dependent activity	1/46	491/14323	0.00203666	0.6341539	-0.4682297	0.79953939	0.79953939	0.62069505	Acsf3	1

ID	Description	GeneRatio	BgRatio	RichFactor	FoldEnrichm	zScore	pvalue	p.adjust	qvalue	geneID	Count
GO:0170055	lipid transmembrane transporter activity	2/40	38/14323	0.05263158	18.8460526	5.82929398	0.00501699	0.3393819	0.30722993	Slc10a6/Slc43a3	2
GO:0019894	kinesin binding	2/40	49/14323	0.04081633	14.6153061	5.05213232	0.00823184	0.3393819	0.30722993	Arhgef10/Fam83d	2
GO:0008028	monocarboxylic acid transmembrane transporter activity	2/40	56/14323	0.03571429	12.7883929	4.67739876	0.01064815	0.3393819	0.30722993	Slc10a6/Slc43a3	2
GO:0001882	nucleoside binding	1/40	10/14323	0.1	35.8075	5.82679325	0.02758731	0.3393819	0.30722993	Pnp	1
GO:0004726	non-membrane spanning protein tyrosine phosphatase activity	1/40	10/14323	0.1	35.8075	5.82679325	0.02758731	0.3393819	0.30722993	Ptpn1	1
GO:0036435	K48-linked polyubiquitin modification-dependent protein binding	1/40	10/14323	0.1	35.8075	5.82679325	0.02758731	0.3393819	0.30722993	Zfand2b	1
GO:0042301	phosphate ion binding	1/40	10/14323	0.1	35.8075	5.82679325	0.02758731	0.3393819	0.30722993	Pnp	1
GO:0098882	structural constituent of presynaptic active zone	1/40	10/14323	0.1	35.8075	5.82679325	0.02758731	0.3393819	0.30722993	Ctbp2	1
GO:0140161	monocarboxylate:sodium symporter activity	1/40	10/14323	0.1	35.8075	5.82679325	0.02758731	0.3393819	0.30722993	Slc10a6	1
GO:0004198	calcium-dependent cysteine-type endopeptidase activity	1/40	11/14323	0.09090909	32.5522727	5.5398619	0.03030487	0.3393819	0.30722993	Capn10	1
GO:0070878	primary miRNA binding	1/40	11/14323	0.09090909	32.5522727	5.5398619	0.03030487	0.3393819	0.30722993	Smad1	1
GO:0015125	bile acid transmembrane transporter activity	1/40	12/14323	0.08333333	29.8395833	5.28891669	0.03301503	0.3393819	0.30722993	Slc10a6	1
GO:0015355	secondary active monocarboxylate transmembrane transporter activity	1/40	13/14323	0.07692308	27.5442308	5.0669208	0.0357178	0.3393819	0.30722993	Slc10a6	1
GO:0015245	fatty acid transmembrane transporter activity	1/40	15/14323	0.06666667	23.8716667	4.69003506	0.04110127	0.3393819	0.30722993	Slc43a3	1
GO:0030169	low-density lipoprotein particle binding	1/40	15/14323	0.06666667	23.8716667	4.69003506	0.04110127	0.3393819	0.30722993	Samd1	1
GO:0051400	BH domain binding	1/40	15/14323	0.06666667	23.8716667	4.69003506	0.04110127	0.3393819	0.30722993	Bok	1
GO:0071933	Arp2/3 complex binding	1/40	15/14323	0.06666667	23.8716667	4.69003506	0.04110127	0.3393819	0.30722993	Iqgap2	1
GO:0043138	3'-5' DNA helicase activity	1/40	16/14323	0.0625	22.3796875	4.52802867	0.04378201	0.3393819	0.30722993	Fbh1	1
GO:0070411	I-SMAD binding	1/40	16/14323	0.0625	22.3796875	4.52802867	0.04378201	0.3393819	0.30722993	Smad1	1
GO:0140994	RNA polymerase II CTD heptapeptide repeat modifying activity	1/40	17/14323	0.05882353	21.0632353	4.38014441	0.04645544	0.3393819	0.30722993	Ctdspl	1
GO:0005319	lipid transporter activity	2/40	125/14323	0.016	5.7292	2.81029049	0.04751347	0.3393819	0.30722993	Slc10a6/Slc43a3	2
GO:0050699	WW domain binding	1/40	19/14323	0.05263158	18.8460526	4.11919481	0.05178044	0.33963206	0.30745639	Wbp1	1
GO:0016922	nuclear receptor binding	2/40	142/14323	0.01408451	5.04330986	2.56241744	0.05959706	0.33963206	0.30745639	Cry1/Ctbp2	2
GO:0046943	carboxylic acid transmembrane transporter activity	2/40	144/14323	0.01388889	4.97326389	2.53587573	0.06108246	0.33963206	0.30745639	Slc10a6/Slc43a3	2
GO:0005342	organic acid transmembrane transporter activity	2/40	145/14323	0.0137931	4.93896552	2.52278829	0.06182997	0.33963206	0.30745639	Slc10a6/Slc43a3	2
GO:0005158	insulin receptor binding	1/40	23/14323	0.04347826	15.5684783	3.70025967	0.06234362	0.33963206	0.30745639	Ptpn1	1
GO:0005452	solute:inorganic anion antiporter activity	1/40	23/14323	0.04347826	15.5684783	3.70025967	0.06234362	0.33963206	0.30745639	Slc25a30	1
GO:0005343	organic acid:sodium symporter activity	1/40	25/14323	0.04	14.323	3.52822683	0.0675821	0.33963206	0.30745639	Slc10a6	1
GO:0004721	phosphoprotein phosphatase activity	2/40	155/14323	0.01290323	4.62032258	2.39817547	0.06947591	0.33963206	0.30745639	Ctdspl/Ptpn1	2
GO:0071813	lipoprotein particle binding	1/40	26/14323	0.03846154	13.7721154	3.44944449	0.07019063	0.33963206	0.30745639	Samd1	1
GO:0071814	protein-lipid complex binding	1/40	26/14323	0.03846154	13.7721154	3.44944449	0.07019063	0.33963206	0.30745639	Samd1	1
GO:0042910	xenobiotic transmembrane transporter activity	1/40	27/14323	0.03703704	13.262037	3.37488787	0.07279204	0.34121267	0.30888726	Slc43a3	1
GO:0042974	nuclear retinoic acid receptor binding	1/40	30/14323	0.03333333	11.9358333	3.17302085	0.08055377	0.3530082	0.31956532	Ctbp2	1
GO:0046875	ephrin receptor binding	1/40	31/14323	0.03225806	11.5508065	3.11201808	0.0831269	0.3530082	0.31956532	Ptpn1	1
GO:0008168	methyltransferase activity	2/40	179/14323	0.01117318	4.00083799	2.13798696	0.08899102	0.3530082	0.31956532	Rrp8/Prdm15	2
GO:1901618	organic hydroxy compound transmembrane transporter activity	1/40	34/14323	0.02941176	10.5316176	2.94460649	0.09080426	0.3530082	0.31956532	Slc10a6	1
GO:0003714	transcription corepressor activity	2/40	184/14323	0.01086957	3.89211957	2.08947856	0.09324418	0.3530082	0.31956532	Cys1/Ctbp2	2
GO:0140030	modification-dependent protein binding	2/40	184/14323	0.01086957	3.89211957	2.08947856	0.09324418	0.3530082	0.31956532	Rrp8/Zfand2b	2
GO:0016741	transferase activity, transferring one-carbon groups	2/40	188/14323	0.0106383	3.80930851	2.05188287	0.09668935	0.3530082	0.31956532	Rrp8/Prdm15	2
GO:0070888	E-box binding	1/40	37/14323	0.02702703	9.6777027	2.79687089	0.09841892	0.3530082	0.31956532	Cry1	1
GO:0030291	protein serine/threonine kinase inhibitor activity	1/40	38/14323	0.02631579	9.42302632	2.75132533	0.1009433	0.3530082	0.31956532	Pkib	1
GO:0098918	structural constituent of synapse	1/40	38/14323	0.02631579	9.42302632	2.75132533	0.1009433	0.3530082	0.31956532	Ctbp2	1
GO:0071949	FAD binding	1/40	39/14323	0.02564103	9.18141026	2.70743267	0.10346079	0.3530082	0.31956532	Cry1	1
GO:0051721	protein phosphatase 2A binding	1/40	40/14323	0.025	8.951875	2.66509034	0.1059714	0.3530082	0.31956532	Ptpn1	1
GO:0030695	GTPase regulator activity	3/40	414/14323	0.00724638	2.59474638	1.74247258	0.10825585	0.3530082	0.31956532	Iqgap2/Arhgef10/Sipa1l2	3
GO:0060589	nucleoside-triphosphatase regulator activity	3/40	414/14323	0.00724638	2.59474638	1.74247258	0.10825585	0.3530082	0.31956532	Iqgap2/Arhgef10/Sipa1l2	3
GO:0008514	organic anion transmembrane transporter activity	2/40	204/14323	0.00980392	3.51053922	1.91118284	0.11082422	0.35369431	0.32018643	Slc10a6/Slc43a3	2
GO:0005547	phosphatidylinositol-3,4,5-trisphosphate binding	1/40	44/14323	0.02272727	8.13806818	2.50945982	0.11594547	0.36150506	0.32725721	Iqgap2	1
GO:0015291	secondary active transmembrane transporter activity	2/40	218/14323	0.00917431	3.28509174	1.79915034	0.12361445	0.36150506	0.32725721	Slc10a6/Slc25a30	2
GO:0019902	phosphatase binding	2/40	218/14323	0.00917431	3.28509174	1.79915034	0.12361445	0.36150506	0.32725721	Cry1/Ptpn1	2
GO:0003743	translation initiation factor activity	1/40	48/14323	0.02083333	7.45989583	2.37235779	0.12581102	0.36150506	0.32725721	Eif3j1	1
GO:0016763	pentosyltransferase activity	1/40	48/14323	0.02083333	7.45989583	2.37235779	0.12581102	0.36150506	0.32725721	Pnp	1
GO:0015370	solute:sodium symporter activity	1/40	49/14323	0.02040816	7.30765306	2.34053481	0.12826059	0.36150506	0.32725721	Slc10a6	1
GO:0005096	GTPase activator activity	2/40	225/14323	0.00888889	3.18288889	1.74649058	0.13014182	0.36150506	0.32725721	Iqgap2/Sipa1l2	2
GO:0003678	DNA helicase activity	1/40	54/14323	0.01851852	6.63101852	2.19386136	0.14040838	0.37557193	0.33999144	Fbh1	1
GO:0016791	phosphatase activity	2/40	239/14323	0.0083682	2.99644351	1.64708147	0.14343337	0.37557193	0.33999144	Ctdspl/Ptpn1	2
GO:0001222	transcription corepressor binding	1/40	56/14323	0.01785714	6.39419643	2.14030937	0.14522115	0.37557193	0.33999144	Ctbp2	1
GO:0031593	polyubiquitin modification-dependent protein binding	1/40	56/14323	0.01785714	6.39419643	2.14030937	0.14522115	0.37557193	0.33999144	Zfand2b	1
GO:0045296	cadherin binding	1/40	59/14323	0.01694915	6.0690678	2.06469332	0.15239107	0.38691997	0.3502644	Cdh19	1
GO:0051287	NAD binding	1/40	60/14323	0.01666667	5.96791667	2.04064096	0.15476799	0.38691997	0.3502644	Ctbp2	1
GO:0004197	cysteine-type endopeptidase activity	1/40	66/14323	0.01515152	5.42537879	1.90691114	0.16889362	0.40254077	0.36440533	Capn10	1
GO:0017018	myosin phosphatase activity	1/40	68/14323	0.01470588	5.26580882	1.8659257	0.17355081	0.40254077	0.36440533	Ctdspl	1
GO:0003924	GTPase activity	2/40	275/14323	0.00727273	2.60418182	1.42146051	0.17880629	0.40254077	0.36440533	Rhot1/Rab24	2
GO:0004860	protein kinase inhibitor activity	1/40	71/14323	0.01408451	5.04330986	1.80738388	0.18048892	0.40254077	0.36440533	Pkib	1
GO:1990841	promoter-specific chromatin binding	1/40	71/14323	0.01408451	5.04330986	1.80738388	0.18048892	0.40254077	0.36440533	Prdm15	1
GO:0030971	receptor tyrosine kinase binding	1/40	72/14323	0.01388889	4.97326389	1.78859948	0.18278898	0.40254077	0.36440533	Ptpn1	1
GO:0019210	kinase inhibitor activity	1/40	74/14323	0.01351351	4.83885135	1.75205233	0.18737023	0.40254077	0.36440533	Pkib	1
GO:0035064	methylated histone binding	1/40	74/14323	0.01351351	4.83885135	1.75205233	0.18737023	0.40254077	0.36440533	Rrp8	1
GO:0140034	methylation-dependent protein binding	1/40	76/14323	0.01315789	4.71151316	1.71679406	0.19192644	0.40254077	0.36440533	Rrp8	1
GO:0008135	translation factor activity, RNA binding	1/40	77/14323	0.01298701	4.65032468	1.69962261	0.19419519	0.40254077	0.36440533	Eif3j1	1
GO:0015294	solute:monoatomic cation symporter activity	1/40	77/14323	0.01298701	4.65032468	1.69962261	0.19419519	0.40254077	0.36440533	Slc10a6	1
GO:0046332	SMAD binding	1/40	77/14323	0.01298701	4.65032468	1.69962261	0.19419519	0.40254077	0.36440533	Smad1	1
GO:0050660	flavin adenine dinucleotide binding	1/40	80/14323	0.0125	4.4759375	1.6498267	0.20096426	0.40254077	0.36440533	Cry1	1
GO:0031625	ubiquitin protein ligase binding	2/40	297/14323	0.00673401	2.41127946	1.30060806	0.2010613	0.40254077	0.36440533	Bok/Smad1	2
GO:0004722	protein serine/threonine phosphatase activity	1/40	82/14323	0.01219512	4.36676829	1.61797581	0.20544613	0.40254077	0.36440533	Ctdspl	1
GO:0004725	protein tyrosine phosphatase activity	1/40	87/14323	0.01149425	4.1158046	1.54261647	0.21654385	0.40254077	0.36440533	Ptpn1	1
GO:0044389	ubiquitin-like protein ligase binding	2/40	313/14323	0.00638978	2.28801917	1.21926341	0.21747878	0.40254077	0.36440533	Bok/Smad1	2
GO:0042578	phosphoric ester hydrolase activity	2/40	318/14323	0.00628931	2.25204403	1.1948509	0.22261121	0.40254077	0.36440533	Ctdspl/Ptpn1	2
GO:1990756	ubiquitin-like ligase-substrate adaptor activity	1/40	91/14323	0.01098901	3.93489011	1.4862836	0.22531308	0.40254077	0.36440533	Lrrc75a	1
GO:0008083	growth factor activity	1/40	92/14323	0.01086957	3.89211957	1.47270093	0.22749039	0.40254077	0.36440533	Cntf	1
GO:0090079	translation regulator activity, nucleic acid binding	1/40	93/14323	0.01075269	3.85026882	1.45930798	0.22966173	0.40254077	0.36440533	Eif3j1	1
GO:0005525	GTP binding	2/40	329/14323	0.00607903	2.17674772	1.1427007	0.23398406	0.40254077	0.36440533	Rhot1/Rab24	2
GO:0016616	oxidoreductase activity, acting on the CH-OH group of donors, NAD or NADP as acceptor	1/40	95/14323	0.01052632	3.76921053	1.43307183	0.23398658	0.40254077	0.36440533	Ctbp2	1
GO:0022804	active transmembrane transporter activity	2/40	336/14323	0.00595238	2.13139881	1.11056807	0.24124338	0.40254077	0.36440533	Slc10a6/Slc25a30	2
GO:0061629	RNA polymerase II-specific DNA-binding transcription factor binding	2/40	336/14323	0.00595238	2.13139881	1.11056807	0.24124338	0.40254077	0.36440533	Cry1/Ctbp2	2
GO:0043130	ubiquitin binding	1/40	99/14323	0.01010101	3.61691919	1.38267194	0.24256538	0.40254077	0.36440533	Zfand2b	1
GO:0140767	enzyme-substrate adaptor activity	1/40	101/14323	0.00990099	3.54529703	1.35844148	0.24681957	0.40254077	0.36440533	Lrrc75a	1
GO:0005125	cytokine activity	1/40	102/14323	0.00980392	3.51053922	1.34655513	0.24893793	0.40254077	0.36440533	Cntf	1
GO:0016614	oxidoreductase activity, acting on CH-OH group of donors	1/40	102/14323	0.00980392	3.51053922	1.34655513	0.24893793	0.40254077	0.36440533	Ctbp2	1
GO:0019001	guanyl nucleotide binding	2/40	345/14323	0.0057971	2.0757971	1.07038376	0.2505958	0.40254077	0.36440533	Rhot1/Rab24	2
GO:0032561	guanyl ribonucleotide binding	2/40	345/14323	0.0057971	2.0757971	1.07038376	0.2505958	0.40254077	0.36440533	Rhot1/Rab24	2
GO:0015103	inorganic anion transmembrane transporter activity	1/40	103/14323	0.00970874	3.47645631	1.33481659	0.25105047	0.40254077	0.36440533	Slc25a30	1
GO:0008013	beta-catenin binding	1/40	104/14323	0.00961538	3.44302885	1.32322242	0.25315722	0.40254077	0.36440533	Cdh19	1
GO:0015293	symporter activity	1/40	106/14323	0.00943396	3.37806604	1.30045394	0.2573534	0.40254077	0.36440533	Slc10a6	1
GO:0015297	antiporter activity	1/40	107/14323	0.00934579	3.34649533	1.2892733	0.25944286	0.40254077	0.36440533	Slc25a30	1
GO:0070851	growth factor receptor binding	1/40	107/14323	0.00934579	3.34649533	1.2892733	0.25944286	0.40254077	0.36440533	Cntf	1
GO:1990782	protein tyrosine kinase binding	1/40	108/14323	0.00925926	3.31550926	1.27822433	0.26152659	0.40254077	0.36440533	Ptpn1	1
GO:0008094	ATP-dependent activity, acting on DNA	1/40	109/14323	0.00917431	3.28509174	1.26730412	0.2636046	0.40254077	0.36440533	Fbh1	1
GO:0000149	SNARE binding	1/40	110/14323	0.00909091	3.25522727	1.25650983	0.26567691	0.40254077	0.36440533	Capn10	1
GO:0015081	sodium ion transmembrane transporter activity	1/40	118/14323	0.00847458	3.0345339	1.1743767	0.28205196	0.41157193	0.3725809	Slc10a6	1
GO:0003697	single-stranded DNA binding	1/40	119/14323	0.00840336	3.00903361	1.16460182	0.28407363	0.41157193	0.3725809	Fbh1	1
GO:0042826	histone deacetylase binding	1/40	119/14323	0.00840336	3.00903361	1.16460182	0.28407363	0.41157193	0.3725809	Cry1	1
GO:0032182	ubiquitin-like protein binding	1/40	120/14323	0.00833333	2.98395833	1.15492887	0.28608976	0.41157193	0.3725809	Zfand2b	1
GO:0005201	extracellular matrix structural constituent	1/40	121/14323	0.00826446	2.95929752	1.14535583	0.28810035	0.41157193	0.3725809	Matn2	1
GO:0045182	translation regulator activity	1/40	121/14323	0.00826446	2.95929752	1.14535583	0.28810035	0.41157193	0.3725809	Eif3j1	1
GO:0001221	transcription coregulator binding	1/40	123/14323	0.00813008	2.91117886	1.12650164	0.29210498	0.4133561	0.37419605	Ctbp2	1
GO:0004386	helicase activity	1/40	139/14323	0.00719424	2.57607914	0.98811655	0.323361	0.45330981	0.41036467	Fbh1	1
GO:0008234	cysteine-type peptidase activity	1/40	141/14323	0.0070922	2.53953901	0.97219554	0.3271721	0.4544057	0.41135674	Capn10	1
GO:0016853	isomerase activity	1/40	157/14323	0.00636943	2.28073248	0.85389947	0.35691672	0.4911698	0.44463792	Fbh1	1
GO:0005509	calcium ion binding	2/40	466/14323	0.00429185	1.53680258	0.62344056	0.37556286	0.50039423	0.45298846	Matn2/Rhot1	2
GO:1901981	phosphatidylinositol phosphate binding	1/40	170/14323	0.00588235	2.10632353	0.76789965	0.38013662	0.50039423	0.45298846	Iqgap2	1
GO:0140297	DNA-binding transcription factor binding	2/40	471/14323	0.00424628	1.52048832	0.60783725	0.3806031	0.50039423	0.45298846	Cry1/Ctbp2	2
GO:0019903	protein phosphatase binding	1/40	171/14323	0.00584795	2.09400585	0.76160695	0.38188851	0.50039423	0.45298846	Ptpn1	1
GO:0003712	transcription coregulator activity	2/40	473/14323	0.00422833	1.5140592	0.60164586	0.38261484	0.50039423	0.45298846	Cys1/Ctbp2	2
GO:0005126	cytokine receptor binding	1/40	172/14323	0.00581395	2.0818314	0.75535716	0.38363558	0.50039423	0.45298846	Cntf	1
GO:0005516	calmodulin binding	1/40	178/14323	0.00561798	2.0116573	0.71872716	0.3940173	0.50950513	0.46123623	Iqgap2	1
GO:0022853	active monoatomic ion transmembrane transporter activity	1/40	194/14323	0.00515464	1.84574742	0.62763615	0.42087544	0.53958389	0.48846542	Slc10a6	1
GO:0005085	guanyl-nucleotide exchange factor activity	1/40	197/14323	0.00507614	1.81763959	0.6115156	0.42578047	0.54124637	0.48997039	Arhgef10	1
GO:0051015	actin filament binding	1/40	201/14323	0.00497512	1.78146766	0.59044989	0.43225756	0.54486247	0.49324392	Iqgap2	1
GO:0019887	protein kinase regulator activity	1/40	210/14323	0.0047619	1.70511905	0.54473407	0.44657164	0.55821455	0.50533107	Pkib	1
GO:0140097	catalytic activity, acting on DNA	1/40	216/14323	0.00462963	1.65775463	0.51546071	0.45591823	0.562452	0.50916707	Fbh1	1
GO:0016757	glycosyltransferase activity	1/40	217/14323	0.00460829	1.65011521	0.51067002	0.45746096	0.562452	0.50916707	Pnp	1
GO:0042393	histone binding	1/40	225/14323	0.00444444	1.59144444	0.47320418	0.4696502	0.57274414	0.51848417	Rrp8	1
GO:0019207	kinase regulator activity	1/40	242/14323	0.00413223	1.47964876	0.3982322	0.49467238	0.598394	0.54170405	Pkib	1
GO:0008017	microtubule binding	1/40	248/14323	0.00403226	1.44385081	0.37313051	0.50322588	0.59975819	0.542939	Fam83d	1
GO:0046982	protein heterodimerization activity	1/40	249/14323	0.00401606	1.43805221	0.36901064	0.50463767	0.59975819	0.542939	Bok	1
GO:0004175	endopeptidase activity	1/40	255/14323	0.00392157	1.40421569	0.34465865	0.51302663	0.59975819	0.542939	Capn10	1
GO:0003713	transcription coactivator activity	1/40	257/14323	0.00389105	1.39328794	0.33667742	0.51579205	0.59975819	0.542939	Ctbp2	1
GO:0050839	cell adhesion molecule binding	1/40	257/14323	0.00389105	1.39328794	0.33667742	0.51579205	0.59975819	0.542939	Cdh19	1
GO:0035091	phosphatidylinositol binding	1/40	265/14323	0.00377358	1.35122642	0.305401	0.52670139	0.60773238	0.55015773	Iqgap2	1
GO:0031267	small GTPase binding	1/40	284/14323	0.00352113	1.26082746	0.23494517	0.55165952	0.62361308	0.56453395	Iqgap2	1
GO:0048018	receptor ligand activity	1/40	284/14323	0.00352113	1.26082746	0.23494517	0.55165952	0.62361308	0.56453395	Cntf	1
GO:0004857	enzyme inhibitor activity	1/40	285/14323	0.00350877	1.25640351	0.23137471	0.55293693	0.62361308	0.56453395	Pkib	1
GO:0030546	signaling receptor activator activity	1/40	289/14323	0.00346021	1.23901384	0.21722177	0.5580112	0.6246394	0.56546303	Cntf	1
GO:0030545	signaling receptor regulator activity	1/40	319/14323	0.0031348	1.12249216	0.11708458	0.59431901	0.66035446	0.59779456	Cntf	1
GO:0051020	GTPase binding	1/40	324/14323	0.00308642	1.10516975	0.10132975	0.60008061	0.66185362	0.59915169	Iqgap2	1
GO:0016887	ATP hydrolysis activity	1/40	342/14323	0.00292398	1.04700292	0.04655764	0.62016924	0.67380866	0.60997415	Fbh1	1
GO:0046873	metal ion transmembrane transporter activity	1/40	346/14323	0.00289017	1.03489884	0.03477477	0.6244979	0.67380866	0.60997415	Slc10a6	1
GO:0015631	tubulin binding	1/40	353/14323	0.00283286	1.01437677	0.01447347	0.63195762	0.67380866	0.60997415	Fam83d	1
GO:0001228	DNA-binding transcription activator activity, RNA polymerase II-specific	1/40	357/14323	0.00280112	1.0030112	0.00304902	0.63615523	0.67380866	0.60997415	Smad1	1
GO:0015267	channel activity	1/40	362/14323	0.00276243	0.98915746	-0.0110573	0.64133664	0.67380866	0.60997415	Bok	1
GO:0022803	passive transmembrane transporter activity	1/40	362/14323	0.00276243	0.98915746	-0.0110573	0.64133664	0.67380866	0.60997415	Bok	1
GO:0001216	DNA-binding transcription activator activity	1/40	363/14323	0.00275482	0.98643251	-0.0138558	0.64236425	0.67380866	0.60997415	Smad1	1
GO:0140678	molecular function inhibitor activity	1/40	399/14323	0.00250627	0.89743108	-0.1099619	0.67750579	0.70373518	0.63706553	Pkib	1
GO:0003779	actin binding	1/40	402/14323	0.00248756	0.89073383	-0.1175941	0.68027734	0.70373518	0.63706553	Iqgap2	1
GO:0008233	peptidase activity	1/40	416/14323	0.00240385	0.86075721	-0.1525193	0.69290714	0.7118909	0.6444486	Capn10	1
GO:0005543	phospholipid binding	1/40	428/14323	0.00233645	0.83662383	-0.1815948	0.70334443	0.7176984	0.64970592	Iqgap2	1
GO:0022890	inorganic cation transmembrane transporter activity	1/40	446/14323	0.00224215	0.80285874	-0.2238305	0.71835483	0.72806233	0.659088	Slc10a6	1
GO:0008324	monoatomic cation transmembrane transporter activity	1/40	477/14323	0.00209644	0.75068134	-0.2930717	0.74248821	0.74747136	0.67665828	Slc10a6	1
GO:0140657	ATP-dependent activity	1/40	491/14323	0.00203666	0.72927699	-0.323032	0.75271463	0.75271463	0.68140482	Fbh1	1

ID	Description	GeneRatio	BgRatio	RichFactor	FoldEnrichm	zScore	pvalue	p.adjust	qvalue	geneID	Count
GO:0140311	protein sequestering activity	3/94	26/14323	0.11538462	17.5814239	6.87803861	0.00063794	0.14884586	0.14386059	Cdkn1a/Sfn/Ctnnd1	3
GO:0051059	NF-kappaB binding	3/94	35/14323	0.08571429	13.0604863	5.80619602	0.00153879	0.14884586	0.14386059	Hspa1b/Commd7/Taf4b	3
GO:0051082	unfolded protein binding	4/94	80/14323	0.05	7.61861702	4.82491959	0.00188014	0.14884586	0.14386059	Hspa1b/Ptges3/Uggt1/Cct7	4
GO:0001637	G protein-coupled chemoattractant receptor activity	2/94	11/14323	0.18181818	27.7040619	7.20115363	0.00225524	0.14884586	0.14386059	Gpr35/Gpr75	2
GO:0004950	chemokine receptor activity	2/94	11/14323	0.18181818	27.7040619	7.20115363	0.00225524	0.14884586	0.14386059	Gpr35/Gpr75	2
GO:0140313	molecular sequestering activity	3/94	44/14323	0.06818182	10.3890232	5.06963189	0.00298399	0.16411961	0.15862279	Cdkn1a/Sfn/Ctnnd1	3
GO:0015216	purine nucleotide transmembrane transporter activity	2/94	19/14323	0.10526316	16.0391937	5.33152163	0.00677651	0.31946383	0.30876409	Abcc5/Slc25a53	2
GO:0015215	nucleotide transmembrane transporter activity	2/94	22/14323	0.09090909	13.8520309	4.90318717	0.00903814	0.33139858	0.32029911	Abcc5/Slc25a53	2
GO:0070182	DNA polymerase binding	2/94	22/14323	0.09090909	13.8520309	4.90318717	0.00903814	0.33139858	0.32029911	Ptges3/Nhej1	2
GO:0140662	ATP-dependent protein folding chaperone	2/94	29/14323	0.06896552	10.5084373	4.16590947	0.01542032	0.39733168	0.38402392	Hspa1b/Cct7	2
GO:0016776	phosphotransferase activity, phosphate group as acceptor	2/94	30/14323	0.06666667	10.158156	4.08117804	0.01645195	0.39733168	0.38402392	Pmvk/Nme1	2
GO:0005154	epidermal growth factor receptor binding	2/94	32/14323	0.0625	9.52327128	3.92309263	0.01860092	0.39733168	0.38402392	Shc1/Nrg2	2
GO:0101005	deubiquitinase activity	3/94	87/14323	0.03448276	5.25421864	3.23492349	0.01951562	0.39733168	0.38402392	Stambpl1/Otub2/Usp31	3
GO:0016853	isomerase activity	4/94	157/14323	0.02547771	3.88209785	2.95130097	0.01979074	0.39733168	0.38402392	Pdia6/Cyp2j13/Fkbp1b/Ptges3	4
GO:0046982	protein heterodimerization activity	5/94	249/14323	0.02008032	3.05968555	2.66483543	0.02397944	0.39733168	0.38402392	Mettl3/Tuba1a/Pafah1b2/Taf4b/Smc1a	5
GO:0001727	lipid kinase activity	2/94	38/14323	0.05263158	8.01959686	3.52161941	0.02570422	0.39733168	0.38402392	Sphk2/Pip4k2c	2
GO:0019001	guanyl nucleotide binding	6/94	345/14323	0.0173913	2.64995375	2.52137459	0.02593956	0.39733168	0.38402392	Rab39/Tuba1a/Nme1/Mmaa/Mief1/Hbs1l	6
GO:0032561	guanyl ribonucleotide binding	6/94	345/14323	0.0173913	2.64995375	2.52137459	0.02593956	0.39733168	0.38402392	Rab39/Tuba1a/Nme1/Mmaa/Mief1/Hbs1l	6
GO:0019783	ubiquitin-like protein peptidase activity	3/94	98/14323	0.03061224	4.6644594	2.95852597	0.0266052	0.39733168	0.38402392	Stambpl1/Otub2/Usp31	3
GO:0008200	ion channel inhibitor activity	2/94	41/14323	0.04878049	7.43279709	3.35255444	0.0296056	0.39733168	0.38402392	Fkbp1b/Pcsk9	2
GO:0015605	organophosphate ester transmembrane transporter activity	2/94	42/14323	0.04761905	7.25582573	3.29995904	0.03095533	0.39733168	0.38402392	Abcc5/Slc25a53	2
GO:0016248	channel inhibitor activity	2/94	42/14323	0.04761905	7.25582573	3.29995904	0.03095533	0.39733168	0.38402392	Fkbp1b/Pcsk9	2
GO:0016860	intramolecular oxidoreductase activity	2/94	42/14323	0.04761905	7.25582573	3.29995904	0.03095533	0.39733168	0.38402392	Pdia6/Ptges3	2
GO:0141110	transporter inhibitor activity	2/94	42/14323	0.04761905	7.25582573	3.29995904	0.03095533	0.39733168	0.38402392	Fkbp1b/Pcsk9	2
GO:0140223	general transcription initiation factor activity	2/94	43/14323	0.04651163	7.0870856	3.24906279	0.03232907	0.39733168	0.38402392	Ubtf/Taf4b	2
GO:0015932	nucleobase-containing compound transmembrane transporter activity	2/94	47/14323	0.04255319	6.48392938	3.06066703	0.03805688	0.39733168	0.38402392	Abcc5/Slc25a53	2
GO:0031625	ubiquitin protein ligase binding	5/94	297/14323	0.01683502	2.56519092	2.21542574	0.04577516	0.39733168	0.38402392	Hspa1b/Cdkn1a/Traf2/Becn1/Caml	5
GO:0140097	catalytic activity, acting on DNA	4/94	216/14323	0.01851852	2.82171001	2.19264013	0.05374554	0.39733168	0.38402392	Dnase1l3/Ptges3/Lig4/Nme1	4
GO:0004536	DNA nuclease activity	2/94	57/14323	0.03508772	5.34639791	2.67235637	0.05388433	0.39733168	0.38402392	Dnase1l3/Nme1	2
GO:0044389	ubiquitin-like protein ligase binding	5/94	313/14323	0.01597444	2.43406295	2.08497037	0.05502404	0.39733168	0.38402392	Hspa1b/Cdkn1a/Traf2/Becn1/Caml	5
GO:0004896	cytokine receptor activity	2/94	59/14323	0.03389831	5.16516408	2.60564969	0.05728693	0.39733168	0.38402392	Gpr35/Gpr75	2
GO:0044183	protein folding chaperone	2/94	59/14323	0.03389831	5.16516408	2.60564969	0.05728693	0.39733168	0.38402392	Hspa1b/Cct7	2
GO:0045309	protein phosphorylated amino acid binding	2/94	59/14323	0.03389831	5.16516408	2.60564969	0.05728693	0.39733168	0.38402392	Shc1/Sfn	2
GO:0008093	cytoskeletal anchor activity	1/94	10/14323	0.1	15.237234	3.66048693	0.06374348	0.39733168	0.38402392	Bicd2	1
GO:0008392	arachidonate epoxygenase activity	1/94	10/14323	0.1	15.237234	3.66048693	0.06374348	0.39733168	0.38402392	Cyp2j13	1
GO:0016493	C-C chemokine receptor activity	1/94	10/14323	0.1	15.237234	3.66048693	0.06374348	0.39733168	0.38402392	Gpr75	1
GO:0019215	intermediate filament binding	1/94	10/14323	0.1	15.237234	3.66048693	0.06374348	0.39733168	0.38402392	Nme1	1
GO:0031996	thioesterase binding	1/94	10/14323	0.1	15.237234	3.66048693	0.06374348	0.39733168	0.38402392	Traf2	1
GO:0034452	dynactin binding	1/94	10/14323	0.1	15.237234	3.66048693	0.06374348	0.39733168	0.38402392	Bicd2	1
GO:0045125	bioactive lipid receptor activity	1/94	10/14323	0.1	15.237234	3.66048693	0.06374348	0.39733168	0.38402392	Sphk2	1
GO:0097177	mitochondrial ribosome binding	1/94	10/14323	0.1	15.237234	3.66048693	0.06374348	0.39733168	0.38402392	Mrps27	1
GO:0005525	GTP binding	5/94	329/14323	0.01519757	2.31568906	1.96227039	0.06528477	0.39733168	0.38402392	Rab39/Tuba1a/Nme1/Mmaa/Hbs1l	5
GO:0052689	carboxylic ester hydrolase activity	3/94	143/14323	0.02097902	3.19662253	2.14567484	0.06781629	0.39733168	0.38402392	Lipa/Smpd4/Pafah1b2	3
GO:0008391	arachidonate monooxygenase activity	1/94	11/14323	0.09090909	13.8520309	3.46574427	0.0698923	0.39733168	0.38402392	Cyp2j13	1
GO:0045503	dynein light chain binding	1/94	11/14323	0.09090909	13.8520309	3.46574427	0.0698923	0.39733168	0.38402392	Dnai4	1
GO:0050815	phosphoserine residue binding	1/94	11/14323	0.09090909	13.8520309	3.46574427	0.0698923	0.39733168	0.38402392	Sfn	1
GO:0016887	ATP hydrolysis activity	5/94	342/14323	0.01461988	2.2276658	1.86768084	0.07436384	0.39733168	0.38402392	Hspa1b/Abcc5/Cct7/Mmaa/Smc1a	5
GO:0048029	monosaccharide binding	2/94	69/14323	0.02898551	4.41658958	2.31221147	0.07534796	0.39733168	0.38402392	P4ha1/Cln5	2
GO:0004706	JUN kinase kinase kinase activity	1/94	12/14323	0.08333333	12.697695	3.29484128	0.07600117	0.39733168	0.38402392	Map3k21	1
GO:0004861	cyclin-dependent protein serine/threonine kinase inhibitor activity	1/94	12/14323	0.08333333	12.697695	3.29484128	0.07600117	0.39733168	0.38402392	Cdkn1a	1
GO:0005159	insulin-like growth factor receptor binding	1/94	12/14323	0.08333333	12.697695	3.29484128	0.07600117	0.39733168	0.38402392	Shc1	1
GO:0008559	ABC-type xenobiotic transporter activity	1/94	12/14323	0.08333333	12.697695	3.29484128	0.07600117	0.39733168	0.38402392	Abcc5	1
GO:0016888	endodeoxyribonuclease activity, producing 5'-phosphomonoesters	1/94	12/14323	0.08333333	12.697695	3.29484128	0.07600117	0.39733168	0.38402392	Dnase1l3	1
GO:0022841	potassium ion leak channel activity	1/94	12/14323	0.08333333	12.697695	3.29484128	0.07600117	0.39733168	0.38402392	Kcnk10	1
GO:0031543	peptidyl-proline dioxygenase activity	1/94	12/14323	0.08333333	12.697695	3.29484128	0.07600117	0.39733168	0.38402392	P4ha1	1
GO:0035251	UDP-glucosyltransferase activity	1/94	12/14323	0.08333333	12.697695	3.29484128	0.07600117	0.39733168	0.38402392	Uggt1	1
GO:0035673	oligopeptide transmembrane transporter activity	1/94	12/14323	0.08333333	12.697695	3.29484128	0.07600117	0.39733168	0.38402392	Abcc5	1
GO:0140776	protein-containing complex destabilizing activity	1/94	12/14323	0.08333333	12.697695	3.29484128	0.07600117	0.39733168	0.38402392	Hspa1b	1
GO:0008174	mRNA methyltransferase activity	1/94	13/14323	0.07692308	11.7209493	3.14313961	0.08207034	0.39733168	0.38402392	Mettl3	1
GO:0019855	calcium channel inhibitor activity	1/94	13/14323	0.07692308	11.7209493	3.14313961	0.08207034	0.39733168	0.38402392	Fkbp1b	1
GO:0052816	long-chain fatty acyl-CoA hydrolase activity	1/94	13/14323	0.07692308	11.7209493	3.14313961	0.08207034	0.39733168	0.38402392	Cln5	1
GO:0061578	K63-linked deubiquitinase activity	1/94	13/14323	0.07692308	11.7209493	3.14313961	0.08207034	0.39733168	0.38402392	Stambpl1	1
GO:0004559	alpha-mannosidase activity	1/94	14/14323	0.07142857	10.8837386	3.00717844	0.08810007	0.39733168	0.38402392	Edem1	1
GO:0005068	transmembrane receptor protein tyrosine kinase adaptor activity	1/94	14/14323	0.07142857	10.8837386	3.00717844	0.08810007	0.39733168	0.38402392	Shc1	1
GO:0008081	phosphoric diester hydrolase activity	2/94	76/14323	0.02631579	4.00979843	2.13826274	0.08892072	0.39733168	0.38402392	Smpd4/Pde6d	2
GO:0003756	protein disulfide isomerase activity	1/94	15/14323	0.06666667	10.158156	2.88431557	0.0940906	0.39733168	0.38402392	Pdia6	1
GO:0005165	neurotrophin receptor binding	1/94	15/14323	0.06666667	10.158156	2.88431557	0.0940906	0.39733168	0.38402392	Shc1	1
GO:0008179	adenylate cyclase binding	1/94	15/14323	0.06666667	10.158156	2.88431557	0.0940906	0.39733168	0.38402392	Cap1	1
GO:0008301	DNA binding, bending	1/94	15/14323	0.06666667	10.158156	2.88431557	0.0940906	0.39733168	0.38402392	Sap30l	1
GO:0015562	efflux transmembrane transporter activity	1/94	15/14323	0.06666667	10.158156	2.88431557	0.0940906	0.39733168	0.38402392	Abcc5	1
GO:0015923	mannosidase activity	1/94	15/14323	0.06666667	10.158156	2.88431557	0.0940906	0.39733168	0.38402392	Edem1	1
GO:0016864	intramolecular oxidoreductase activity, transposing S-S bonds	1/94	15/14323	0.06666667	10.158156	2.88431557	0.0940906	0.39733168	0.38402392	Pdia6	1
GO:0022840	leak channel activity	1/94	15/14323	0.06666667	10.158156	2.88431557	0.0940906	0.39733168	0.38402392	Kcnk10	1
GO:0022842	narrow pore channel activity	1/94	15/14323	0.06666667	10.158156	2.88431557	0.0940906	0.39733168	0.38402392	Kcnk10	1
GO:0030169	low-density lipoprotein particle binding	1/94	15/14323	0.06666667	10.158156	2.88431557	0.0940906	0.39733168	0.38402392	Pcsk9	1
GO:0045504	dynein heavy chain binding	1/94	15/14323	0.06666667	10.158156	2.88431557	0.0940906	0.39733168	0.38402392	Dnai4	1
GO:0051787	misfolded protein binding	1/94	15/14323	0.06666667	10.158156	2.88431557	0.0940906	0.39733168	0.38402392	Edem1	1
GO:0004843	cysteine-type deubiquitinase activity	2/94	79/14323	0.02531646	3.85752761	2.069982	0.09494471	0.39733168	0.38402392	Otub2/Usp31	2
GO:0140375	immune receptor activity	2/94	80/14323	0.025	3.80930851	2.04796374	0.09697843	0.39733168	0.38402392	Gpr35/Gpr75	2
GO:0004550	nucleoside diphosphate kinase activity	1/94	16/14323	0.0625	9.52327128	2.77249381	0.1000422	0.39733168	0.38402392	Nme1	1
GO:0120020	cholesterol transfer activity	1/94	16/14323	0.0625	9.52327128	2.77249381	0.1000422	0.39733168	0.38402392	Stard3	1
GO:0019903	protein phosphatase binding	3/94	171/14323	0.01754386	2.67319895	1.78902044	0.10241779	0.39733168	0.38402392	Shc1/Traf2/Ctnnd1	3
GO:0042626	ATPase-coupled transmembrane transporter activity	2/94	83/14323	0.02409639	3.67162266	1.98398207	0.10315323	0.39733168	0.38402392	Abcc5/Atp1b3	2
GO:0140657	ATP-dependent activity	6/94	491/14323	0.01221996	1.86198379	1.57970477	0.10368935	0.39733168	0.38402392	Hspa1b/Abcc5/Atp1b3/Cct7/Mmaa/Smc1a	6
GO:0000295	adenine nucleotide transmembrane transporter activity	1/94	17/14323	0.05882353	8.96307885	2.67008409	0.10595511	0.39733168	0.38402392	Slc25a53	1
GO:0003746	translation elongation factor activity	1/94	17/14323	0.05882353	8.96307885	2.67008409	0.10595511	0.39733168	0.38402392	Hbs1l	1
GO:0061133	endopeptidase activator activity	1/94	17/14323	0.05882353	8.96307885	2.67008409	0.10595511	0.39733168	0.38402392	Aph1b	1
GO:0120015	sterol transfer activity	1/94	17/14323	0.05882353	8.96307885	2.67008409	0.10595511	0.39733168	0.38402392	Stard3	1
GO:0016298	lipase activity	2/94	85/14323	0.02352941	3.58523154	1.94295609	0.10732872	0.39796041	0.38463159	Lipa/Smpd4	2
GO:0034185	apolipoprotein binding	1/94	18/14323	0.05555556	8.46513002	2.57577705	0.11182959	0.40030657	0.38689917	Pcsk9	1
GO:0043024	ribosomal small subunit binding	1/94	18/14323	0.05555556	8.46513002	2.57577705	0.11182959	0.40030657	0.38689917	Nme1	1
GO:0046527	glucosyltransferase activity	1/94	18/14323	0.05555556	8.46513002	2.57577705	0.11182959	0.40030657	0.38689917	Uggt1	1
GO:0005537	D-mannose binding	1/94	19/14323	0.05263158	8.01959686	2.48850658	0.11766587	0.40030657	0.38689917	Cln5	1
GO:0010314	phosphatidylinositol-5-phosphate binding	1/94	19/14323	0.05263158	8.01959686	2.48850658	0.11766587	0.40030657	0.38689917	Sap30l	1
GO:0031418	L-ascorbic acid binding	1/94	19/14323	0.05263158	8.01959686	2.48850658	0.11766587	0.40030657	0.38689917	P4ha1	1
GO:0047555	3',5'-cyclic-GMP phosphodiesterase activity	1/94	19/14323	0.05263158	8.01959686	2.48850658	0.11766587	0.40030657	0.38689917	Pde6d	1
GO:0051959	dynein light intermediate chain binding	1/94	19/14323	0.05263158	8.01959686	2.48850658	0.11766587	0.40030657	0.38689917	Bicd2	1
GO:0140678	molecular function inhibitor activity	5/94	399/14323	0.01253133	1.90942782	1.49744628	0.12174642	0.40144351	0.38799803	Cdkn1a/Serpinb8/Fkbp1b/Pcsk9/Pde6d	5
GO:0004114	3',5'-cyclic-nucleotide phosphodiesterase activity	1/94	20/14323	0.05	7.61861702	2.40739443	0.12346421	0.40144351	0.38799803	Pde6d	1
GO:0070840	dynein complex binding	1/94	20/14323	0.05	7.61861702	2.40739443	0.12346421	0.40144351	0.38799803	Bicd2	1
GO:1904047	S-adenosyl-L-methionine binding	1/94	20/14323	0.05	7.61861702	2.40739443	0.12346421	0.40144351	0.38799803	Mettl3	1
GO:0005164	tumor necrosis factor receptor binding	1/94	21/14323	0.04761905	7.25582573	2.33170967	0.12922484	0.40144351	0.38799803	Traf2	1
GO:0015271	outward rectifier potassium channel activity	1/94	21/14323	0.04761905	7.25582573	2.33170967	0.12922484	0.40144351	0.38799803	Kcnk10	1
GO:0004112	cyclic-nucleotide phosphodiesterase activity	1/94	22/14323	0.04545455	6.92601547	2.26083824	0.13494802	0.40144351	0.38799803	Pde6d	1
GO:0016289	acyl-CoA hydrolase activity	1/94	22/14323	0.04545455	6.92601547	2.26083824	0.13494802	0.40144351	0.38799803	Cln5	1
GO:0047617	fatty acyl-CoA hydrolase activity	1/94	22/14323	0.04545455	6.92601547	2.26083824	0.13494802	0.40144351	0.38799803	Cln5	1
GO:0008233	peptidase activity	5/94	416/14323	0.01201923	1.83139832	1.39867771	0.13811325	0.40144351	0.38799803	Stambpl1/Otub2/Adamts8/Pcsk9/Usp31	5
GO:0016798	hydrolase activity, acting on glycosyl bonds	2/94	100/14323	0.02	3.04744681	1.66991973	0.13995269	0.40144351	0.38799803	Edem1/Cln5	2
GO:0004709	MAP kinase kinase kinase activity	1/94	23/14323	0.04347826	6.62488437	2.19425992	0.14063398	0.40144351	0.38799803	Map3k21	1
GO:0005158	insulin receptor binding	1/94	23/14323	0.04347826	6.62488437	2.19425992	0.14063398	0.40144351	0.38799803	Shc1	1
GO:0052742	phosphatidylinositol kinase activity	1/94	23/14323	0.04347826	6.62488437	2.19425992	0.14063398	0.40144351	0.38799803	Pip4k2c	1
GO:0120283	protein serine/threonine kinase binding	1/94	23/14323	0.04347826	6.62488437	2.19425992	0.14063398	0.40144351	0.38799803	Cdkn1a	1
GO:0016712	oxidoreductase activity, acting on paired donors, with incorporation or reduction of molecular oxygen, red	1/94	24/14323	0.04166667	6.34884752	2.13153063	0.14628296	0.40144351	0.38799803	Cyp2j13	1
GO:0050750	low-density lipoprotein particle receptor binding	1/94	24/14323	0.04166667	6.34884752	2.13153063	0.14628296	0.40144351	0.38799803	Pcsk9	1
GO:0051219	phosphoprotein binding	2/94	103/14323	0.01941748	2.95868622	1.62148246	0.14671557	0.40144351	0.38799803	Shc1/Sfn	2
GO:0030246	carbohydrate binding	3/94	204/14323	0.01470588	2.24076971	1.45071757	0.15027151	0.40144351	0.38799803	P4ha1/Mlec/Cln5	3
GO:0022884	macromolecule transmembrane transporter activity	1/94	25/14323	0.04	6.09489362	2.07226862	0.15189519	0.40144351	0.38799803	Abcc5	1
GO:0030547	signaling receptor inhibitor activity	1/94	25/14323	0.04	6.09489362	2.07226862	0.15189519	0.40144351	0.38799803	Pcsk9	1
GO:0070851	growth factor receptor binding	2/94	107/14323	0.01869159	2.84808113	1.55956251	0.15583545	0.40144351	0.38799803	Shc1/Nrg2	2
GO:0001671	ATPase activator activity	1/94	26/14323	0.03846154	5.86047463	2.01614363	0.15747093	0.40144351	0.38799803	Atp1b3	1
GO:0031435	mitogen-activated protein kinase kinase kinase binding	1/94	26/14323	0.03846154	5.86047463	2.01614363	0.15747093	0.40144351	0.38799803	Traf2	1
GO:0034061	DNA polymerase activity	1/94	26/14323	0.03846154	5.86047463	2.01614363	0.15747093	0.40144351	0.38799803	Ptges3	1
GO:0070325	lipoprotein particle receptor binding	1/94	26/14323	0.03846154	5.86047463	2.01614363	0.15747093	0.40144351	0.38799803	Pcsk9	1
GO:0071813	lipoprotein particle binding	1/94	26/14323	0.03846154	5.86047463	2.01614363	0.15747093	0.40144351	0.38799803	Pcsk9	1
GO:0071814	protein-lipid complex binding	1/94	26/14323	0.03846154	5.86047463	2.01614363	0.15747093	0.40144351	0.38799803	Pcsk9	1
GO:0072349	modified amino acid transmembrane transporter activity	1/94	26/14323	0.03846154	5.86047463	2.01614363	0.15747093	0.40144351	0.38799803	Abcc5	1
GO:0035591	signaling adaptor activity	2/94	108/14323	0.01851852	2.82171001	1.54452969	0.15813249	0.40144351	0.38799803	Shc1/Traf2	2
GO:1990782	protein tyrosine kinase binding	2/94	108/14323	0.01851852	2.82171001	1.54452969	0.15813249	0.40144351	0.38799803	Shc1/Ctnnd1	2
GO:0061135	endopeptidase regulator activity	2/94	109/14323	0.01834862	2.79582276	1.52966784	0.160436	0.40144351	0.38799803	Serpinb8/Aph1b	2
GO:0000287	magnesium ion binding	3/94	212/14323	0.01415094	2.15621236	1.37849507	0.16281082	0.40144351	0.38799803	Lig4/Nudt3/Nme1	3
GO:0015662	P-type ion transporter activity	1/94	27/14323	0.03703704	5.64342002	1.96286826	0.16301039	0.40144351	0.38799803	Atp1b3	1
GO:0042910	xenobiotic transmembrane transporter activity	1/94	27/14323	0.03703704	5.64342002	1.96286826	0.16301039	0.40144351	0.38799803	Abcc5	1
GO:0046625	sphingolipid binding	1/94	27/14323	0.03703704	5.64342002	1.96286826	0.16301039	0.40144351	0.38799803	Traf2	1
GO:0140358	P-type transmembrane transporter activity	1/94	27/14323	0.03703704	5.64342002	1.96286826	0.16301039	0.40144351	0.38799803	Atp1b3	1
GO:0043022	ribosome binding	2/94	112/14323	0.01785714	2.72093465	1.48607128	0.16738351	0.40590921	0.39231416	Nme1/Mrps27	2
GO:0017147	Wnt-protein binding	1/94	28/14323	0.03571429	5.4418693	1.91219106	0.16851382	0.40590921	0.39231416	Sfrp5	1
GO:0070273	phosphatidylinositol-4-phosphate binding	1/94	28/14323	0.03571429	5.4418693	1.91219106	0.16851382	0.40590921	0.39231416	Sap30l	1
GO:0019902	phosphatase binding	3/94	218/14323	0.01376147	2.09686707	1.32639926	0.1724175	0.40812991	0.39446049	Shc1/Traf2/Ctnnd1	3
GO:0030371	translation repressor activity	1/94	29/14323	0.03448276	5.25421864	1.8638909	0.17398145	0.40812991	0.39446049	Nhlrc1	1
GO:0030551	cyclic nucleotide binding	1/94	29/14323	0.03448276	5.25421864	1.8638909	0.17398145	0.40812991	0.39446049	Fkbp1b	1
GO:0015399	primary active transmembrane transporter activity	2/94	115/14323	0.0173913	2.64995375	1.44388561	0.17438278	0.40812991	0.39446049	Abcc5/Atp1b3	2
GO:0015035	protein-disulfide reductase activity	1/94	30/14323	0.03333333	5.07907801	1.81777244	0.1794135	0.41694686	0.40298214	Pdia6	1
GO:0042887	amide transmembrane transporter activity	1/94	31/14323	0.03225806	4.91523679	1.77366235	0.18481021	0.42352339	0.4093384	Abcc5	1
GO:0046875	ephrin receptor binding	1/94	31/14323	0.03225806	4.91523679	1.77366235	0.18481021	0.42352339	0.4093384	Shc1	1
GO:0045182	translation regulator activity	2/94	121/14323	0.01652893	2.51855108	1.36340822	0.18851833	0.42904173	0.41467191	Nhlrc1/Hbs1l	2
GO:0004930	G protein-coupled receptor activity	3/94	231/14323	0.01298701	1.97886156	1.21904439	0.19376287	0.43298324	0.41848141	Gpr35/Gpr75/Sphk2	3
GO:0003755	peptidyl-prolyl cis-trans isomerase activity	1/94	33/14323	0.03030303	4.61734365	1.69086602	0.19549849	0.43298324	0.41848141	Fkbp1b	1
GO:0017080	sodium channel regulator activity	1/94	33/14323	0.03030303	4.61734365	1.69086602	0.19549849	0.43298324	0.41848141	Pcsk9	1
GO:0043015	gamma-tubulin binding	1/94	33/14323	0.03030303	4.61734365	1.69086602	0.19549849	0.43298324	0.41848141	Nme1	1
GO:0016251	RNA polymerase II general transcription initiation factor activity	1/94	35/14323	0.02857143	4.35349544	1.61444998	0.20604812	0.44153168	0.42674354	Taf4b	1
GO:0016859	cis-trans isomerase activity	1/94	35/14323	0.02857143	4.35349544	1.61444998	0.20604812	0.44153168	0.42674354	Fkbp1b	1
GO:0016893	endonuclease activity, active with either ribo- or deoxyribonucleic acids and producing 5'-phosphomonoe	1/94	35/14323	0.02857143	4.35349544	1.61444998	0.20604812	0.44153168	0.42674354	Dnase1l3	1
GO:0030332	cyclin binding	1/94	35/14323	0.02857143	4.35349544	1.61444998	0.20604812	0.44153168	0.42674354	Cdkn1a	1
GO:0032813	tumor necrosis factor receptor superfamily binding	1/94	35/14323	0.02857143	4.35349544	1.61444998	0.20604812	0.44153168	0.42674354	Traf2	1
GO:0140359	ABC-type transporter activity	1/94	36/14323	0.02777778	4.23256501	1.57836182	0.21127148	0.44931399	0.4342652	Abcc5	1
GO:0015079	potassium ion transmembrane transporter activity	2/94	131/14323	0.01526718	2.32629527	1.23946036	0.21240298	0.44931399	0.4342652	Kcnk10/Atp1b3	2
GO:0004520	DNA endonuclease activity	1/94	37/14323	0.02702703	4.11817136	1.54356204	0.21646085	0.45210178	0.43695961	Dnase1l3	1
GO:0015036	disulfide oxidoreductase activity	1/94	37/14323	0.02702703	4.11817136	1.54356204	0.21646085	0.45210178	0.43695961	Pdia6	1
GO:0016705	oxidoreductase activity, acting on paired donors, with incorporation or reduction of molecular oxygen	2/94	134/14323	0.01492537	2.27421404	1.20447409	0.21962927	0.45268019	0.43751865	P4ha1/Cyp2j13	2
GO:0019205	nucleobase-containing compound kinase activity	1/94	38/14323	0.02631579	4.00979843	1.50996771	0.22161643	0.45268019	0.43751865	Nme1	1
GO:0030291	protein serine/threonine kinase inhibitor activity	1/94	38/14323	0.02631579	4.00979843	1.50996771	0.22161643	0.45268019	0.43751865	Cdkn1a	1
GO:0008237	metallopeptidase activity	2/94	136/14323	0.01470588	2.24076971	1.18166378	0.22445892	0.45268019	0.43751865	Stambpl1/Adamts8	2
GO:0030159	signaling receptor complex adaptor activity	1/94	39/14323	0.02564103	3.90698309	1.4775033	0.22673845	0.45268019	0.43751865	Shc1	1
GO:0043531	ADP binding	1/94	39/14323	0.02564103	3.90698309	1.4775033	0.22673845	0.45268019	0.43751865	Mief1	1
GO:0043548	phosphatidylinositol 3-kinase binding	1/94	39/14323	0.02564103	3.90698309	1.4775033	0.22673845	0.45268019	0.43751865	Becn1	1

GO:0099106	ion channel regulator activity	2/94	138/14323	0.01449275	2.20829479	1.15924798	0.22929702	0.45268019	0.43751865	Fkbp1b/Pcsk9	2
GO:0016538	cyclin-dependent protein serine/threonine kinase regulator activity	1/94	40/14323	0.025	3.80930851	1.44609986	0.23182713	0.45268019	0.43751865	Cdkn1a	1
GO:0016790	thiolester hydrolase activity	1/94	40/14323	0.025	3.80930851	1.44609986	0.23182713	0.45268019	0.43751865	Cln5	1
GO:0051721	protein phosphatase 2A binding	1/94	40/14323	0.025	3.80930851	1.44609986	0.23182713	0.45268019	0.43751865	Shc1	1
GO:0008234	cysteine-type peptidase activity	2/94	141/14323	0.0141844	2.16130979	1.12633473	0.23656777	0.45714198	0.44183101	Otub2/Usp31	2
GO:0005246	calcium channel regulator activity	1/94	41/14323	0.02439024	3.71639855	1.41569427	0.23688266	0.45714198	0.44183101	Fkbp1b	1
GO:0031072	heat shock protein binding	2/94	144/14323	0.01388889	2.11628251	1.09423634	0.24385183	0.4642546	0.4487054	Hspa1b/Ptges3	2
GO:1901505	carbohydrate derivative transmembrane transporter activity	1/94	43/14323	0.02325581	3.5435428	1.35764976	0.24689519	0.4642546	0.4487054	Abcc5	1
GO:0061134	peptidase regulator activity	2/94	146/14323	0.01369863	2.08729233	1.07327041	0.24871365	0.4642546	0.4487054	Serpinb8/Aph1b	2
GO:0001784	phosphotyrosine residue binding	1/94	44/14323	0.02272727	3.46300774	1.32990858	0.2518526	0.4642546	0.4487054	Shc1	1
GO:0032266	phosphatidylinositol-3-phosphate binding	1/94	44/14323	0.02272727	3.46300774	1.32990858	0.2518526	0.4642546	0.4487054	Sap30l	1
GO:0120013	lipid transfer activity	1/94	44/14323	0.02272727	3.46300774	1.32990858	0.2518526	0.4642546	0.4487054	Stard3	1
GO:0016247	channel regulator activity	2/94	148/14323	0.01351351	2.05908568	1.05263787	0.25357899	0.4642546	0.4487054	Fkbp1b/Pcsk9	2
GO:0001046	core promoter sequence-specific DNA binding	1/94	45/14323	0.02222222	3.38605201	1.30295989	0.25677773	0.4642546	0.4487054	Ubtf	1
GO:0015485	cholesterol binding	1/94	45/14323	0.02222222	3.38605201	1.30295989	0.25677773	0.4642546	0.4487054	Stard3	1
GO:0031491	nucleosome binding	1/94	45/14323	0.02222222	3.38605201	1.30295989	0.25677773	0.4642546	0.4487054	Sap30l	1
GO:0016758	hexosyltransferase activity	2/94	150/14323	0.01333333	2.03163121	1.03232817	0.25844694	0.4642546	0.4487054	Uggt1/Fut11	2
GO:0141108	transporter regulator activity	2/94	150/14323	0.01333333	2.03163121	1.03232817	0.25844694	0.4642546	0.4487054	Fkbp1b/Pcsk9	2
GO:0043014	alpha-tubulin binding	1/94	46/14323	0.02173913	3.31244218	1.27676183	0.26167077	0.4642546	0.4487054	Efhc1	1
GO:0051879	Hsp90 protein binding	1/94	46/14323	0.02173913	3.31244218	1.27676183	0.26167077	0.4642546	0.4487054	Ptges3	1
GO:0060590	ATPase regulator activity	1/94	46/14323	0.02173913	3.31244218	1.27676183	0.26167077	0.4642546	0.4487054	Atp1b3	1
GO:0008408	3'-5' exonuclease activity	1/94	47/14323	0.0212766	3.24196469	1.25127567	0.26653195	0.46784863	0.45217906	Nme1	1
GO:0019829	ATPase-coupled monoatomic cation transmembrane transporter activity	1/94	47/14323	0.0212766	3.24196469	1.25127567	0.26653195	0.46784863	0.45217906	Atp1b3	1
GO:0003924	GTPase activity	3/94	275/14323	0.01090909	1.66224371	0.90127124	0.26995094	0.47134291	0.45555631	Rab39/Mmaa/Hbs1l	3
GO:0004714	transmembrane receptor protein tyrosine kinase activity	1/94	50/14323	0.02	3.04744681	1.17874149	0.28092627	0.48536999	0.46911358	Nrg2	1
GO:0016667	oxidoreductase activity, acting on a sulfur group of donors	1/94	50/14323	0.02	3.04744681	1.17874149	0.28092627	0.48536999	0.46911358	Pdia6	1
GO:0016504	peptidase activator activity	1/94	51/14323	0.01960784	2.98769295	1.15576766	0.28566199	0.48822025	0.47186838	Aph1b	1
GO:0004857	enzyme inhibitor activity	3/94	285/14323	0.01052632	1.60391937	0.83700364	0.28780277	0.48822025	0.47186838	Cdkn1a/Serpinb8/Pde6d	3
GO:0030695	GTPase regulator activity	4/94	414/14323	0.00966184	1.47219653	0.79241662	0.28849378	0.48822025	0.47186838	Agfg1/Tbck/Pde6d/Rasgef1a	4
GO:0060589	nucleoside-triphosphatase regulator activity	4/94	414/14323	0.00966184	1.47219653	0.79241662	0.28849378	0.48822025	0.47186838	Agfg1/Tbck/Pde6d/Rasgef1a	4
GO:1901682	sulfur compound transmembrane transporter activity	1/94	52/14323	0.01923077	2.93023732	1.13334921	0.29036685	0.48888296	0.47250889	Abcc5	1
GO:0004867	serine-type endopeptidase inhibitor activity	1/94	53/14323	0.01886792	2.87494982	1.11146085	0.29504105	0.4942312	0.477678	Serpinb8	1
GO:0008028	monocarboxylic acid transmembrane transporter activity	1/94	56/14323	0.01785714	2.72093465	1.04874676	0.30888166	0.51480277	0.49756057	Slc16a5	1
GO:0005126	cytokine receptor binding	2/94	172/14323	0.01162791	1.7717714	0.82763321	0.31192749	0.51716806	0.49984664	Shc1/Traf2	2
GO:0032934	sterol binding	1/94	57/14323	0.01754386	2.67319895	1.02875622	0.31343519	0.51716806	0.49984664	Stard3	1
GO:0004518	nuclease activity	2/94	175/14323	0.01142857	1.74139818	0.80205026	0.31917864	0.52402463	0.50647357	Dnase1l3/Nme1	2
GO:0016706	2-oxoglutarate-dependent dioxygenase activity	1/94	59/14323	0.01694915	2.58258204	0.99003408	0.32245337	0.52418528	0.50662884	P4ha1	1
GO:0045296	cadherin binding	1/94	59/14323	0.01694915	2.58258204	0.99003408	0.32245337	0.52418528	0.50662884	Ctnnd1	1
GO:0008173	RNA methyltransferase activity	1/94	61/14323	0.01639344	2.49790722	0.95287995	0.33135434	0.53601438	0.51806175	Mettl3	1
GO:0004497	monooxygenase activity	1/94	63/14323	0.01587302	2.41860858	0.91717385	0.34013961	0.54393051	0.52571274	Cyp2j13	1
GO:0050840	extracellular matrix binding	1/94	63/14323	0.01587302	2.41860858	0.91717385	0.34013961	0.54393051	0.52571274	Vwa1	1
GO:0005200	structural constituent of cytoskeleton	1/94	64/14323	0.015625	2.38081782	0.89982987	0.34448932	0.54393051	0.52571274	Tuba1a	1
GO:0030145	manganese ion binding	1/94	64/14323	0.015625	2.38081782	0.89982987	0.34448932	0.54393051	0.52571274	Nudt3	1
GO:0042562	hormone binding	1/94	64/14323	0.015625	2.38081782	0.89982987	0.34448932	0.54393051	0.52571274	Shc1	1
GO:0016747	acyltransferase activity, transferring groups other than amino-acyl groups	2/94	189/14323	0.01058201	1.61240572	0.68883653	0.35275673	0.55199069	0.53350297	Cln5/Pafah1b2	2
GO:0019199	transmembrane receptor protein kinase activity	1/94	66/14323	0.01515152	2.30867182	0.86609836	0.35310382	0.55199069	0.53350297	Nrg2	1
GO:0043021	ribonucleoprotein complex binding	2/94	190/14323	0.01052632	1.60391937	0.68110984	0.3551355	0.55199069	0.53350297	Nme1/Mrps27	2
GO:0004553	hydrolase activity, hydrolyzing O-glycosyl compounds	1/94	67/14323	0.01492537	2.27421404	0.84968806	0.35736897	0.55199069	0.53350297	Edem1	1
GO:0051020	GTPase binding	3/94	324/14323	0.00925926	1.410855	0.6079826	0.3579576	0.55199069	0.53350297	Bicd2/Becn1/Pde6d	3
GO:0004620	phospholipase activity	1/94	69/14323	0.01449275	2.20829479	0.81772569	0.36581598	0.56148499	0.54267927	Smpd4	1
GO:0002039	p53 binding	1/94	70/14323	0.01428571	2.17674772	0.80215411	0.36999819	0.56407203	0.54517967	Ptges3	1
GO:0004860	protein kinase inhibitor activity	1/94	71/14323	0.01408451	2.1460893	0.78684338	0.37415312	0.56407203	0.54517967	Cdkn1a	1
GO:0004527	exonuclease activity	1/94	72/14323	0.01388889	2.11628251	0.77178488	0.37828093	0.56407203	0.54517967	Nme1	1
GO:0019843	rRNA binding	1/94	72/14323	0.01388889	2.11628251	0.77178488	0.37828093	0.56407203	0.54517967	Mrps27	1
GO:0030971	receptor tyrosine kinase binding	1/94	72/14323	0.01388889	2.11628251	0.77178488	0.37828093	0.56407203	0.54517967	Shc1	1
GO:0022804	active transmembrane transporter activity	3/94	336/14323	0.00892857	1.36046733	0.54344028	0.37946664	0.56407203	0.54517967	Abcc5/Atp1b3/Slc16a5	3
GO:0061629	RNA polymerase II-specific DNA-binding transcription factor binding	3/94	336/14323	0.00892857	1.36046733	0.54344028	0.37946664	0.56407203	0.54517967	Hspa1b/Commd7/Taf4b	3
GO:0019210	kinase inhibitor activity	1/94	74/14323	0.01351351	2.05908568	0.74239209	0.38645592	0.57004763	0.55095513	Cdkn1a	1
GO:0008514	organic anion transmembrane transporter activity	2/94	204/14323	0.00980392	1.49384647	0.57740913	0.38809785	0.57004763	0.55095513	Abcc5/Slc16a5	2
GO:0000049	tRNA binding	1/94	76/14323	0.01315789	2.00489922	0.71391443	0.39452455	0.57004763	0.55095513	Mrps27	1
GO:0004222	metalloendopeptidase activity	1/94	76/14323	0.01315789	2.00489922	0.71391443	0.39452455	0.57004763	0.55095513	Adamts8	1
GO:0046873	metal ion transmembrane transporter activity	3/94	346/14323	0.00867052	1.32114746	0.49148937	0.39728394	0.57004763	0.55095513	Trpv2/Kcnk10/Atp1b3	3
GO:0008135	translation factor activity, RNA binding	1/94	77/14323	0.01298701	1.97886156	0.70000113	0.3985194	0.57004763	0.55095513	Hbs1l	1
GO:0051213	dioxygenase activity	1/94	77/14323	0.01298701	1.97886156	0.70000113	0.3985194	0.57004763	0.55095513	P4ha1	1
GO:0019887	protein kinase regulator activity	2/94	210/14323	0.00952381	1.45116515	0.53532077	0.40200647	0.57004763	0.55095513	Cdkn1a/Ankrd54	2
GO:0005249	voltage-gated potassium channel activity	1/94	78/14323	0.01282051	1.95349154	0.68629604	0.40248818	0.57004763	0.55095513	Kcnk10	1
GO:0043178	alcohol binding	1/94	78/14323	0.01282051	1.95349154	0.68629604	0.40248818	0.57004763	0.55095513	Stard3	1
GO:0097110	scaffold protein binding	1/94	78/14323	0.01282051	1.95349154	0.68629604	0.40248818	0.57004763	0.55095513	Ubtf	1
GO:0019003	GDP binding	1/94	79/14323	0.01265823	1.9287638	0.67279293	0.40643104	0.57317198	0.55397484	Mief1	1
GO:0015631	tubulin binding	3/94	353/14323	0.00849858	1.29494907	0.45605241	0.40968027	0.5752957	0.55602743	Efhc1/Togaram2/Nme1	3
GO:0016757	glycosyltransferase activity	2/94	217/14323	0.00921659	1.40435337	0.48782894	0.4180472	0.58455753	0.56497905	Uggt1/Fut11	2
GO:0004252	serine-type endopeptidase activity	1/94	83/14323	0.01204819	1.83581133	0.62068447	0.42194673	0.58752076	0.56784303	Pcsk9	1
GO:0020037	heme binding	1/94	84/14323	0.01190476	1.81395643	0.60810648	0.42576253	0.590343	0.57057075	Cyp2j13	1
GO:0005496	steroid binding	1/94	86/14323	0.01162791	1.7717714	0.58345483	0.43331953	0.59470361	0.57478531	Stard3	1
GO:0005096	GTPase activator activity	2/94	225/14323	0.00888889	1.3544208	0.43552219	0.43611598	0.59470361	0.57478531	Agfg1/Tbck	2
GO:0042393	histone binding	2/94	225/14323	0.00888889	1.3544208	0.43552219	0.43611598	0.59470361	0.57478531	Sphk2/Sap30l	2
GO:1901681	sulfur compound binding	2/94	225/14323	0.00888889	1.3544208	0.43552219	0.43611598	0.59470361	0.57478531	Adamts8/Mettl3	2
GO:0016407	acetyltransferase activity	1/94	88/14323	0.01136364	1.73150387	0.55944552	0.44077812	0.59858756	0.57853918	Pafah1b2	1
GO:0004866	endopeptidase inhibitor activity	1/94	90/14323	0.01111111	1.693026	0.53604486	0.44813957	0.60591007	0.58561643	Serpinb8	1
GO:0004842	ubiquitin-protein transferase activity	3/94	376/14323	0.00797872	1.21573676	0.34455316	0.44984233	0.60591007	0.58561643	Traf2/Nhlrc1/Rnf103	3
GO:0046906	tetrapyrrole binding	1/94	91/14323	0.01098901	1.67442132	0.52456293	0.45178426	0.60605205	0.58575366	Cyp2j13	1
GO:0008083	growth factor activity	1/94	92/14323	0.01086957	1.65622109	0.51322164	0.45540513	0.60831636	0.58794213	Nrg2	1
GO:0051117	ATPase binding	1/94	93/14323	0.01075269	1.63841226	0.50201745	0.45900234	0.60831636	0.58794213	Atp1b3	1
GO:0090079	translation regulator activity, nucleic acid binding	1/94	93/14323	0.01075269	1.63841226	0.50201745	0.45900234	0.60831636	0.58794213	Hbs1l	1
GO:0140104	molecular carrier activity	1/94	94/14323	0.0106383	1.62098234	0.49094695	0.46257604	0.6104036	0.58995947	Mmaa	1
GO:0008236	serine-type peptidase activity	1/94	95/14323	0.01052632	1.60391937	0.48000682	0.46612639	0.6104036	0.58995947	Pcsk9	1
GO:0030414	peptidase inhibitor activity	1/94	95/14323	0.01052632	1.60391937	0.48000682	0.46612639	0.6104036	0.58995947	Serpinb8	1
GO:0005262	calcium channel activity	1/94	96/14323	0.01041667	1.58721188	0.4691939	0.46965353	0.61259156	0.59207414	Trpv2	1
GO:0019207	kinase regulator activity	2/94	242/14323	0.00826446	1.25927554	0.33062147	0.47349557	0.61517142	0.59456759	Cdkn1a/Ankrd54	2
GO:0017171	serine hydrolase activity	1/94	99/14323	0.01010101	1.53911455	0.43748821	0.4800972	0.61887529	0.59814741	Pcsk9	1
GO:0043130	ubiquitin binding	1/94	99/14323	0.01010101	1.53911455	0.43748821	0.4800972	0.61887529	0.59814741	Otub2	1
GO:0019955	cytokine binding	1/94	100/14323	0.01	1.5237234	0.42715446	0.483533	0.62087895	0.60008396	Traf2	1
GO:0005267	potassium channel activity	1/94	101/14323	0.00990099	1.50863703	0.4169336	0.48694634	0.62283834	0.60197773	Kcnk10	1
GO:0019787	ubiquitin-like protein transferase activity	3/94	401/14323	0.0074813	1.1399427	0.23102032	0.49223931	0.6266271	0.60563959	Traf2/Nhlrc1/Rnf103	3
GO:0004519	endonuclease activity	1/94	103/14323	0.00970874	1.47934311	0.3968203	0.4937062	0.6266271	0.60563959	Dnase1l3	1
GO:0008013	beta-catenin binding	1/94	104/14323	0.00961538	1.46511866	0.38692294	0.49705301	0.62845782	0.607409	Ctnnd1	1
GO:0004175	endopeptidase activity	2/94	255/14323	0.00784314	1.19507718	0.25546944	0.50107377	0.62916097	0.60808859	Adamts8/Pcsk9	2
GO:0015293	symporter activity	1/94	106/14323	0.00943396	1.43747491	0.36743513	0.50368109	0.62916097	0.60808859	Slc16a5	1
GO:0005261	monoatomic cation channel activity	2/94	257/14323	0.0077821	1.18577697	0.24425963	0.50523532	0.62916097	0.60808859	Trpv2/Kcnk10	2
GO:0050839	cell adhesion molecule binding	2/94	257/14323	0.0077821	1.18577697	0.24425963	0.50523532	0.62916097	0.60808859	Ctnnd1/Caml	2
GO:0016755	aminoacyltransferase activity	3/94	411/14323	0.00729927	1.11220686	0.18759624	0.50876084	0.63116946	0.61002981	Traf2/Nhlrc1/Rnf103	3
GO:0005506	iron ion binding	1/94	110/14323	0.00909091	1.38520309	0.32962626	0.51667909	0.63620933	0.61490088	P4ha1	1
GO:0019842	vitamin binding	1/94	110/14323	0.00909091	1.38520309	0.32962626	0.51667909	0.63620933	0.61490088	P4ha1	1
GO:0008194	UDP-glycosyltransferase activity	1/94	114/14323	0.00877193	1.33659948	0.29326665	0.52934022	0.64697138	0.62530248	Uggt1	1
GO:0015085	calcium ion transmembrane transporter activity	1/94	114/14323	0.00877193	1.33659948	0.29326665	0.52934022	0.64697138	0.62530248	Trpv2	1
GO:0004713	protein tyrosine kinase activity	1/94	115/14323	0.00869565	1.32497687	0.28438944	0.53245388	0.64837557	0.62665964	Nrg2	1
GO:0022843	voltage-gated monoatomic cation channel activity	1/94	117/14323	0.00854701	1.3023277	0.26687847	0.53862019	0.65107935	0.62927287	Kcnk10	1
GO:0030295	protein kinase activator activity	1/94	117/14323	0.00854701	1.3023277	0.26687847	0.53862019	0.65107935	0.62927287	Cdkn1a	1
GO:0015081	sodium ion transmembrane transporter activity	1/94	118/14323	0.00847458	1.29129102	0.25824151	0.54167311	0.65127895	0.62946578	Atp1b3	1
GO:0003697	single-stranded DNA binding	1/94	119/14323	0.00840336	1.28043984	0.24968153	0.54470603	0.65127895	0.62946578	Nme1	1
GO:0042826	histone deacetylase binding	1/94	119/14323	0.00840336	1.28043984	0.24968153	0.54470603	0.65127895	0.62946578	Hspa1b	1
GO:0032182	ubiquitin-like protein binding	1/94	120/14323	0.00833333	1.2697695	0.24119706	0.5477191	0.65251734	0.6306627	Otub2	1
GO:0005201	extracellular matrix structural constituent	1/94	121/14323	0.00826446	1.25927554	0.23278664	0.55071244	0.6537234	0.63182836	Vwa1	1
GO:0031267	small GTPase binding	2/94	284/14323	0.00704225	1.07304465	0.10105506	0.55920406	0.65928595	0.6372046	Bicd2/Pde6d	2
GO:0019838	growth factor binding	1/94	124/14323	0.00806452	1.2288092	0.20798596	0.55957536	0.65928595	0.6372046	Shc1	1
GO:0005319	lipid transporter activity	1/94	125/14323	0.008	1.21897872	0.19985817	0.56249105	0.65928595	0.6372046	Stard3	1
GO:0022890	inorganic cation transmembrane transporter activity	3/94	446/14323	0.00672646	1.02492606	0.04346621	0.56432164	0.65928595	0.6372046	Trpv2/Kcnk10/Atp1b3	3
GO:0019209	kinase activator activity	1/94	126/14323	0.00793651	1.20930429	0.19179783	0.56538765	0.65928595	0.6372046	Cdkn1a	1
GO:0016779	nucleotidyltransferase activity	1/94	127/14323	0.00787402	1.19978221	0.1838037	0.56826527	0.66030823	0.63819264	Ptges3	1
GO:0008201	heparin binding	1/94	129/14323	0.00775194	1.18118093	0.16800938	0.57396407	0.66458998	0.64233098	Adamts8	1
GO:0002020	protease binding	1/94	130/14323	0.00769231	1.17209493	0.1602069	0.5767855	0.66552173	0.64323153	Hspa1b	1
GO:0061630	ubiquitin protein ligase activity	2/94	297/14323	0.00673401	1.02607637	0.0369094	0.58367971	0.67112998	0.64865194	Traf2/Nhlrc1	2
GO:0051087	protein-folding chaperone binding	1/94	138/14323	0.00724638	1.10414739	0.09991987	0.59870136	0.68462871	0.66169856	Ptges3	1
GO:0005244	voltage-gated monoatomic ion channel activity	1/94	139/14323	0.00719424	1.09620389	0.09263588	0.60136066	0.68462871	0.66169856	Kcnk10	1
GO:0140297	DNA-binding transcription factor binding	3/94	471/14323	0.00636943	0.97052446	-0.0528682	0.60164341	0.68462871	0.66169856	Hspa1b/Commd7/Taf4b	3
GO:0022832	voltage-gated channel activity	1/94	140/14323	0.00714286	1.08837386	0.08540482	0.60400251	0.6849513	0.66201035	Kcnk10	1
GO:0031406	carboxylic acid binding	1/94	141/14323	0.0070922	1.0806549	0.07822582	0.60662705	0.68557166	0.66260993	P4ha1	1
GO:0008324	monoatomic cation transmembrane transporter activity	3/94	477/14323	0.00628931	0.95831661	-0.0752556	0.61028943	0.68729249	0.66427312	Trpv2/Kcnk10/Atp1b3	3
GO:0061659	ubiquitin-like protein ligase activity	2/94	314/14323	0.00636943	0.97052446	-0.0429241	0.61417792	0.68729249	0.66427312	Traf2/Nhlrc1	2
GO:0046943	carboxylic acid transmembrane transporter activity	1/94	144/14323	0.00694444	1.05814125	0.05699298	0.61439783	0.68729249	0.66427312	Slc16a5	1
GO:0005342	organic acid transmembrane transporter activity	1/94	145/14323	0.00689655	1.05084373	0.0500141	0.61695419	0.68742292	0.66439918	Slc16a5	1
GO:0042578	phosphoric ester hydrolase activity	2/94	318/14323	0.00628931	0.95831661	-0.0610961	0.62110496	0.68742292	0.66439918	Smpd4/Pde6d	2
GO:0030545	signaling receptor regulator activity	2/94	319/14323	0.00626959	0.95531248	-0.0656046	0.62282192	0.68742292	0.66439918	Nrg2/Pcsk9	2
GO:0008757	S-adenosylmethionine-dependent methyltransferase activity	1/94	149/14323	0.00671141	1.02263316	0.02257203	0.62701302	0.68742292	0.66439918	Mettl3	1
GO:0016874	ligase activity	1/94	149/14323	0.00671141	1.02263316	0.02257203	0.62701302	0.68742292	0.66439918	Lig4	1
GO:0043177	organic acid binding	1/94	149/14323	0.00671141	1.02263316	0.02257203	0.62701302	0.68742292	0.66439918	P4ha1	1
GO:0005216	monoatomic ion channel activity	2/94	325/14323	0.00615385	0.93767594	-0.0923726	0.63299955	0.69168825	0.66852166	Trpv2/Kcnk10	2
GO:0004672	protein kinase activity	3/94	497/14323	0.00603622	0.91975256	-0.1479925	0.63821418	0.69508475	0.6718044	Cdkn1a/Nrg2/Map3k21	3
GO:0044325	transmembrane transporter binding	1/94	162/14323	0.00617284	0.94057	-0.0618294	0.65793342	0.71420404	0.69028333	Fkbp1b	1
GO:1901981	phosphatidylinositol phosphate binding	1/94	170/14323	0.00588235	0.89630788	-0.1105413	0.6756862	0.73107032	0.70658471	Sap30l	1
GO:0001228	DNA-binding transcription activator activity, RNA polymerase II-specific	2/94	357/14323	0.00560224	0.85362656	-0.227635	0.68372467	0.73700379	0.71231945	Myt1/Rfxap	2
GO:0015267	channel activity	2/94	362/14323	0.00552486	0.84183613	-0.2477319	0.69111847	0.73700379	0.71231945	Trpv2/Kcnk10	2
GO:0022803	passive transmembrane transporter activity	2/94	362/14323	0.00552486	0.84183613	-0.2477319	0.69111847	0.73700379	0.71231945	Trpv2/Kcnk10	2
GO:0005539	glycosaminoglycan binding	1/94	178/14323	0.00561798	0.85602438	-0.1570999	0.69252694	0.73700379	0.71231945	Adamts8	1
GO:0001216	DNA-binding transcription activator activity	2/94	363/14323	0.00550964	0.83951703	-0.2517202	0.6925803	0.73700379	0.71231945	Myt1/Rfxap	2
GO:0008168	methyltransferase activity	1/94	179/14323	0.00558659	0.85124213	-0.1627792	0.69457024	0.73700379	0.71231945	Mettl3	1
GO:0016741	transferase activity, transferring one-carbon groups	1/94	188/14323	0.00531915	0.81049117	-0.2125881	0.71236572	0.75346374	0.72822811	Mettl3	1
GO:0033218	amide binding	1/94	193/14323	0.00518135	0.78949399	-0.2393044	0.72180474	0.7605296	0.73505732	Fkbp1b	1
GO:0022853	active monoatomic ion transmembrane transporter activity	1/94	194/14323	0.00515464	0.78542444	-0.2445705	0.72365544	0.7605296	0.73505732	Atp1b3	1
GO:0005085	guanyl-nucleotide exchange factor activity	1/94	197/14323	0.00507614	0.77346366	-0.2602196	0.72913476	0.76385546	0.73827179	Rasgef1a	1
GO:0015291	secondary active transmembrane transporter activity	1/94	218/14323	0.00458716	0.69895569	-0.3640413	0.76459383	0.79846824	0.77172529	Slc16a5	1
GO:0001664	G protein-coupled receptor binding	1/94	223/14323	0.0044843	0.68328404	-0.3874284	0.77233515	0.8040082	0.7770797	Hspa1b	1
GO:0022836	gated channel activity	1/94	231/14323	0.004329	0.65962052	-0.4238982	0.78420058	0.81379306	0.78653683	Kcnk10	1
GO:0008017	microtubule binding	1/94	248/14323	0.00403226	0.6144046	-0.4978655	0.80742091	0.83526301	0.80728769	Togaram2	1
GO:0005509	calcium ion binding	2/94	466/14323	0.00429185	0.65395854	-0.6172556	0.81521328	0.8406887	0.81253166	Efhc1/Edem1	2
GO:0035091	phosphatidylinositol binding	1/94	265/14323	0.00377358	0.57498996	-0.5675955	0.82816642	0.85138604	0.82287072	Sap30l	1
GO:0048018	receptor ligand activity	1/94	284/14323	0.00352113	0.53652233	-0.6412073	0.84874415	0.86983096	0.84069787	Nrg2	1
GO:0030546	signaling receptor activator activity	1/94	289/14323	0.00346021	0.52723993	-0.6598991	0.85374127	0.8722434	0.84302951	Nrg2	1
GO:0003729	mRNA binding	1/94	316/14323	0.00316456	0.48219095	-0.7565179	0.87803264	0.89429251	0.86434013	Mettl3	1
GO:0106310	protein serine kinase activity	1/94	327/14323	0.0030581	0.46597046	-0.7939913	0.88674319	0.9003854	0.87022895	Map3k21	1
GO:0140098	catalytic activity, acting on RNA	1/94	343/14323	0.00291545	0.44423423	-0.8467668	0.89832534	0.90934774	0.87889112	Mettl3	1
GO:0004674	protein serine/threonine kinase activity	1/94	388/14323	0.00257732	0.39271222	-0.985679	0.92498405	0.93347014	0.90220559	Map3k21	1
GO:0003779	actin binding	1/94	402/14323	0.00248756	0.37903567	-1.0264151	0.93176907	0.93745059	0.90605272	Cap1	1
GO:0005543	phospholipid binding	1/94	428/14323	0.00233645	0.35601014	-1.099386	0.94279842	0.94566407	0.91399111	Sap30l	1
GO:0003712	transcription coregulator activity	1/94	473/14323	0.00211416	0.32214025	-1.218496	0.95787528	0.95787528	0.92579333	Sap30l	1

ID	Description	GeneRatio	BgRatio	RichFactor	FoldEnrichm	zScore	pvalue	p.adjust	qvalue	geneID	Count
GO:0051787	misfolded protein binding	4/135	15/14323	0.26666667	28.2923457	10.3158533	9.5059E-06	0.00331756	0.00296184	Sdf2l1/Hspa5/Dnajc3/Dnajb11	4
GO:0070006	metalloaminopeptidase activity	4/135	20/14323	0.2	21.2192593	8.82622102	3.2528E-05	0.00417356	0.00372605	Mmp14/Lta4h/Lap3/Trhde	4
GO:1902936	phosphatidylinositol bisphosphate binding	7/135	97/14323	0.07216495	7.65643375	6.41644104	3.5876E-05	0.00417356	0.00372605	Rlbp1/Snx21/Scin/Sh3pxd2a/Snx14/Snx18/Wdr45b	7
GO:0004177	aminopeptidase activity	4/135	33/14323	0.12121212	12.8601571	6.65333556	0.00024984	0.02179837	0.01946107	Mmp14/Lta4h/Lap3/Trhde	4
GO:0051087	protein-folding chaperone binding	7/135	138/14323	0.05072464	5.38169619	5.04516372	0.00032878	0.02294887	0.02048821	Sdf2l1/Stip1/Hspd1/St13/Dnajc3/Ubl4a/Bag5	7
GO:0044389	ubiquitin-like protein ligase binding	10/135	313/14323	0.03194888	3.38965803	4.16962488	0.00076175	0.03968681	0.03543144	Hspa5/Calr/Fzd8/Hspd1/Smad2/Bag5/Itch/Srprb/Arih1/Cul2	10
GO:0051082	unfolded protein binding	5/135	80/14323	0.0625	6.63101852	4.92650969	0.00094267	0.03968681	0.03543144	Hspa5/Calr/Hsp90b1/St13/Dnajb11	5
GO:0016780	phosphotransferase activity, for other substituted phosphate groups	3/135	21/14323	0.14285714	15.1566138	6.33255212	0.00096181	0.03968681	0.03543144	Fktn/Pigg/Selenoi	3
GO:0008235	metalloexopeptidase activity	4/135	48/14323	0.08333333	8.84135802	5.30801294	0.00106516	0.03968681	0.03543144	Mmp14/Lta4h/Lap3/Trhde	4
GO:1901981	phosphatidylinositol phosphate binding	7/135	170/14323	0.04117647	4.36867102	4.30989746	0.00113716	0.03968681	0.03543144	Rlbp1/Snx21/Scin/Sh3pxd2a/Snx14/Snx18/Wdr45b	7
GO:0031625	ubiquitin protein ligase binding	9/135	297/14323	0.03030303	3.21503928	3.76271105	0.00200806	0.06234736	0.05566225	Hspa5/Calr/Fzd8/Hspd1/Smad2/Bag5/Srprb/Arih1/Cul2	9
GO:0044183	protein folding chaperone	4/135	59/14323	0.06779661	7.19296924	4.64956574	0.00230044	0.06234736	0.05566225	Hspa5/Hsp90b1/Hspd1/Ric8b	4
GO:0031072	heat shock protein binding	6/135	144/14323	0.04166667	4.42067901	4.02419389	0.00241737	0.06234736	0.05566225	Bmal1/Hspa5/Chordc1/Stip1/St13/Lman2	6
GO:0140662	ATP-dependent protein folding chaperone	3/135	29/14323	0.10344828	10.9754789	5.24522346	0.00250104	0.06234736	0.05566225	Hspa5/Hsp90b1/Hspd1	3
GO:0016853	isomerase activity	6/135	157/14323	0.03821656	4.05463553	3.75399539	0.00371031	0.08632648	0.07707024	Pdia3/Hspd1/Fkbp2/Degs1/Enoph1/P4hb	6
GO:0016671	oxidoreductase activity, acting on a sulfur group of donors, disulfide as acceptor	2/135	11/14323	0.18181818	19.2902357	5.91934441	0.00458732	0.09178291	0.08194161	Pdia3/Msrb2	2
GO:0045294	alpha-catenin binding	2/135	11/14323	0.18181818	19.2902357	5.91934441	0.00458732	0.09178291	0.08194161	Pkp3/Pkp2	2
GO:0005546	phosphatidylinositol-4,5-bisphosphate binding	4/135	72/14323	0.05555556	5.89423868	4.06103612	0.00473379	0.09178291	0.08194161	Snx21/Scin/Sh3pxd2a/Snx18	4
GO:0008238	exopeptidase activity	4/135	78/14323	0.05128205	5.44083571	3.83609108	0.00628571	0.1121321	0.10010889	Mmp14/Lta4h/Lap3/Trhde	4
GO:0008174	mRNA methyltransferase activity	2/135	13/14323	0.15384615	16.3225071	5.39125433	0.00642591	0.1121321	0.10010889	Mettl16/Mettl14	2
GO:0016860	intramolecular oxidoreductase activity	3/135	42/14323	0.07142857	7.57830688	4.16454533	0.00718881	0.11819759	0.10552401	Pdia3/Enoph1/P4hb	3
GO:0001846	opsonin binding	2/135	14/14323	0.14285714	15.1566138	5.16924228	0.00745085	0.11819759	0.10552401	Calr/Cr1l	2
GO:0032266	phosphatidylinositol-3-phosphate binding	3/135	44/14323	0.06818182	7.23383838	4.03962554	0.00818106	0.11927948	0.1064899	Snx21/Sh3pxd2a/Wdr45b	3
GO:0003756	protein disulfide isomerase activity	2/135	15/14323	0.13333333	14.1461728	4.9689386	0.00854438	0.11927948	0.1064899	Pdia3/P4hb	2
GO:0016864	intramolecular oxidoreductase activity, transposing S-S bonds	2/135	15/14323	0.13333333	14.1461728	4.9689386	0.00854438	0.11927948	0.1064899	Pdia3/P4hb	2
GO:0051879	Hsp90 protein binding	3/135	46/14323	0.06521739	6.91932367	3.9222986	0.00925003	0.12416392	0.11085061	Bmal1/Chordc1/Stip1	3
GO:0016667	oxidoreductase activity, acting on a sulfur group of donors	3/135	50/14323	0.06	6.36577778	3.7073892	0.01162267	0.15023371	0.13412511	Pdia3/P4hb/Msrb2	3
GO:0001848	complement binding	2/135	18/14323	0.11111111	11.7884774	4.46745931	0.0122229	0.15234968	0.13601419	Calr/Cr1l	2
GO:0035091	phosphatidylinositol binding	7/135	265/14323	0.02641509	2.80254368	2.88905023	0.01279334	0.15396127	0.13745298	Rlbp1/Snx21/Scin/Sh3pxd2a/Snx14/Snx18/Wdr45b	7
GO:0015459	potassium channel regulator activity	3/135	54/14323	0.05555556	5.89423868	3.51474147	0.01431433	0.16352454	0.14599085	Slc5a3/Kcnip2/Arpp19	3
GO:0008649	rRNA methyltransferase activity	2/135	20/14323	0.1	10.6096296	4.19484779	0.01499365	0.16352454	0.14599085	Mettl16/Tfb1m	2
GO:0016881	acid-amino acid ligase activity	2/135	20/14323	0.1	10.6096296	4.19484779	0.01499365	0.16352454	0.14599085	Gclm/Ppcs	2
GO:0015271	outward rectifier potassium channel activity	2/135	21/14323	0.0952381	10.1044092	4.07259438	0.01647069	0.1690668	0.15093884	Kcnk13/Kcnip2	2
GO:0140102	catalytic activity, acting on a rRNA	2/135	21/14323	0.0952381	10.1044092	4.07259438	0.01647069	0.1690668	0.15093884	Mettl16/Tfb1m	2
GO:0045296	cadherin binding	3/135	59/14323	0.05084746	5.39472693	3.29947897	0.01813566	0.18083844	0.16144828	Pkp3/Pkp2/Cdh10	3
GO:0008106	alcohol dehydrogenase (NADP+) activity	2/135	23/14323	0.08695652	9.2257649	3.85106032	0.01960182	0.18683449	0.16680142	Dhrs3/Akr1a1	2
GO:0008173	RNA methyltransferase activity	3/135	61/14323	0.04918033	5.21785064	3.22013461	0.01980767	0.18683449	0.16680142	Mettl16/Tfb1m/Mettl14	3
GO:0004033	aldo-keto reductase (NADPH) activity	2/135	25/14323	0.08	8.4877037	3.65501478	0.02296088	0.21087753	0.18826647	Dhrs3/Akr1a1	2
GO:0080025	phosphatidylinositol-3,5-bisphosphate binding	2/135	28/14323	0.07142857	7.57830688	3.39867154	0.02840608	0.25419804	0.226942	Snx14/Wdr45b	2
GO:0015035	protein-disulfide reductase activity	2/135	30/14323	0.06666667	7.07308642	3.24800374	0.03229391	0.28176436	0.25155255	Pdia3/P4hb	2
GO:0050839	cell adhesion molecule binding	6/135	257/14323	0.0233463	2.47695633	2.33054257	0.03486316	0.29525005	0.26359226	Calr/Mmp14/Pkp3/Pkp2/Cdh10/P4hb	6
GO:0005525	GTP binding	7/135	329/14323	0.0212766	2.25736801	2.25059603	0.0362528	0.29525005	0.26359226	Rhoj/Rasl10a/Rab38/Rrad/Rasl11a/Rab9b/Srprb	7
GO:0018455	alcohol dehydrogenase [NAD(P)+] activity	2/135	32/14323	0.0625	6.63101852	3.1105613	0.03637751	0.29525005	0.26359226	Dhrs3/Akr1a1	2
GO:0005249	voltage-gated potassium channel activity	3/135	78/14323	0.03846154	4.08062678	2.66111284	0.03733869	0.29616371	0.26440796	Kcnn1/Kcnk13/Kcnip2	3
GO:0001965	G-protein alpha-subunit binding	2/135	33/14323	0.06060606	6.43007856	3.04617674	0.03849004	0.29851161	0.26650411	Sash1/Ric8b	2
GO:0008237	metallopeptidase activity	4/135	136/14323	0.02941176	3.1204793	2.4236301	0.03972084	0.30136025	0.2690473	Mmp14/Lta4h/Lap3/Trhde	4
GO:1901618	organic hydroxy compound transmembrane transporter activity	2/135	34/14323	0.05882353	6.24095861	2.98440253	0.0406483	0.30183524	0.26947137	Slc5a3/Slc51a	2
GO:0099106	ion channel regulator activity	4/135	138/14323	0.02898551	3.07525497	2.38948585	0.04154567	0.30207167	0.26968244	Slc5a3/Rrad/Kcnip2/Arpp19	4
GO:0016859	cis-trans isomerase activity	2/135	35/14323	0.05714286	6.0626455	2.92505441	0.04285127	0.30520596	0.27248066	Fkbp2/Degs1	2
GO:0019001	guanyl nucleotide binding	7/135	345/14323	0.02028986	2.15267848	2.11399201	0.04490544	0.30729406	0.27434487	Rhoj/Rasl10a/Rab38/Rrad/Rasl11a/Rab9b/Srprb	7
GO:0032561	guanyl ribonucleotide binding	7/135	345/14323	0.02028986	2.15267848	2.11399201	0.04490544	0.30729406	0.27434487	Rhoj/Rasl10a/Rab38/Rrad/Rasl11a/Rab9b/Srprb	7
GO:0003924	GTPase activity	6/135	275/14323	0.02181818	2.31482828	2.14751529	0.04592945	0.3082573	0.27520483	Rhoj/Rasl10a/Rab38/Rrad/Rasl11a/Rab9b	6
GO:0015036	disulfide oxidoreductase activity	2/135	37/14323	0.05405405	5.73493493	2.81298673	0.04738725	0.31204055	0.27858242	Pdia3/P4hb	2
GO:0016247	channel regulator activity	4/135	148/14323	0.02702703	2.86746747	2.22756558	0.0513734	0.33202436	0.2964235	Slc5a3/Rrad/Kcnip2/Arpp19	4
GO:0016874	ligase activity	4/135	149/14323	0.02684564	2.84822272	2.21212342	0.05242047	0.33263173	0.29696574	Gclm/Ppcs/Itch/Yars2	4
GO:0141108	transporter regulator activity	4/135	150/14323	0.02666667	2.82923457	2.19680883	0.05347919	0.33328994	0.29755338	Slc5a3/Rrad/Kcnip2/Arpp19	4
GO:0004864	protein phosphatase inhibitor activity	2/135	41/14323	0.04878049	5.17542909	2.61159997	0.05695174	0.34870454	0.31131517	Hsp90b1/Arpp19	2
GO:0017069	snRNA binding	2/135	43/14323	0.04651163	4.93471146	2.52052577	0.06196499	0.37285829	0.33287906	Mettl16/Prpf31	2
GO:0016879	ligase activity, forming carbon-nitrogen bonds	2/135	45/14323	0.04444444	4.71539095	2.43492291	0.06712242	0.39704616	0.35447343	Gclm/Ppcs	2
GO:0019212	phosphatase inhibitor activity	2/135	46/14323	0.04347826	4.61288245	2.39399035	0.06975298	0.40139727	0.35835799	Hsp90b1/Arpp19	2
GO:0005267	potassium channel activity	3/135	101/14323	0.02970297	3.15137514	2.11643349	0.07015826	0.40139727	0.35835799	Kcnn1/Kcnk13/Kcnip2	3
GO:0015370	solute:sodium symporter activity	2/135	49/14323	0.04081633	4.33046107	2.27791589	0.07784171	0.41670987	0.37202872	Slc5a3/Slc17a8	2
GO:0019787	ubiquitin-like protein transferase activity	7/135	401/14323	0.01745636	1.85205505	1.68809605	0.08510044	0.41670987	0.37202872	Dtx4/Obi1/Pja1/Itch/Ube3c/Aktip/Arih1	7
GO:0046982	protein heterodimerization activity	5/135	249/14323	0.02008032	2.13044772	1.75529255	0.08662182	0.41670987	0.37202872	Bmal1/Slc51a/Hexa/Srl/P4hb	5
GO:0008035	high-density lipoprotein particle binding	1/135	10/14323	0.1	10.6096296	2.96516895	0.09038233	0.41670987	0.37202872	Hspd1	1
GO:0019215	intermediate filament binding	1/135	10/14323	0.1	10.6096296	2.96516895	0.09038233	0.41670987	0.37202872	Pkp2	1
GO:0019841	retinol binding	1/135	10/14323	0.1	10.6096296	2.96516895	0.09038233	0.41670987	0.37202872	Rlbp1	1
GO:1990763	arrestin family protein binding	1/135	10/14323	0.1	10.6096296	2.96516895	0.09038233	0.41670987	0.37202872	Itch	1
GO:0003925	G protein activity	2/135	54/14323	0.03703704	3.92949246	2.10378146	0.09193013	0.41670987	0.37202872	Rasl10a/Rasl11a	2
GO:0016755	aminoacyltransferase activity	7/135	411/14323	0.01703163	1.80699288	1.61921291	0.09393281	0.41670987	0.37202872	Dtx4/Obi1/Pja1/Itch/Ube3c/Aktip/Arih1	7
GO:0022843	voltage-gated monoatomic cation channel activity	3/135	117/14323	0.02564103	2.72041785	1.82263244	0.09852989	0.41670987	0.37202872	Kcnn1/Kcnk13/Kcnip2	3
GO:0004143	ATP-dependent diacylglycerol kinase activity	1/135	11/14323	0.09090909	9.64511785	2.79785514	0.09896183	0.41670987	0.37202872	Dgkk	1
GO:0005049	nuclear export signal receptor activity	1/135	11/14323	0.09090909	9.64511785	2.79785514	0.09896183	0.41670987	0.37202872	Calr	1
GO:0008253	5'-nucleotidase activity	1/135	11/14323	0.09090909	9.64511785	2.79785514	0.09896183	0.41670987	0.37202872	Nt5c3b	1
GO:0015269	calcium-activated potassium channel activity	1/135	11/14323	0.09090909	9.64511785	2.79785514	0.09896183	0.41670987	0.37202872	Kcnn1	1
GO:0016670	oxidoreductase activity, acting on a sulfur group of donors, oxygen as acceptor	1/135	11/14323	0.09090909	9.64511785	2.79785514	0.09896183	0.41670987	0.37202872	P4hb	1
GO:0016717	oxidoreductase activity, acting on paired donors, with oxidation of a pair of donors resulting in the reduct	1/135	11/14323	0.09090909	9.64511785	2.79785514	0.09896183	0.41670987	0.37202872	Degs1	1
GO:0017070	U6 snRNA binding	1/135	11/14323	0.09090909	9.64511785	2.79785514	0.09896183	0.41670987	0.37202872	Mettl16	1
GO:0052650	all-trans-retinol dehydrogenase (NADP+) activity	1/135	11/14323	0.09090909	9.64511785	2.79785514	0.09896183	0.41670987	0.37202872	Dhrs3	1
GO:1990247	N6-methyladenosine-containing RNA reader activity	1/135	11/14323	0.09090909	9.64511785	2.79785514	0.09896183	0.41670987	0.37202872	Hnrnpc	1
GO:0030544	Hsp70 protein binding	2/135	57/14323	0.03508772	3.72267706	2.00904923	0.10071273	0.41670987	0.37202872	Stip1/St13	2
GO:0043021	ribonucleoprotein complex binding	4/135	190/14323	0.02105263	2.23360624	1.66972253	0.10502004	0.41670987	0.37202872	Hspa5/Prpf31/Hnrnpc/Itch	4
GO:0004022	alcohol dehydrogenase (NAD+) activity	1/135	12/14323	0.08333333	8.84135802	2.65066623	0.107461	0.41670987	0.37202872	Dhrs3	1
GO:0005326	neurotransmitter transmembrane transporter activity	1/135	12/14323	0.08333333	8.84135802	2.65066623	0.107461	0.41670987	0.37202872	Slc17a8	1
GO:0015125	bile acid transmembrane transporter activity	1/135	12/14323	0.08333333	8.84135802	2.65066623	0.107461	0.41670987	0.37202872	Slc51a	1
GO:0016918	retinal binding	1/135	12/14323	0.08333333	8.84135802	2.65066623	0.107461	0.41670987	0.37202872	Rlbp1	1
GO:0022841	potassium ion leak channel activity	1/135	12/14323	0.08333333	8.84135802	2.65066623	0.107461	0.41670987	0.37202872	Kcnk13	1
GO:0031543	peptidyl-proline dioxygenase activity	1/135	12/14323	0.08333333	8.84135802	2.65066623	0.107461	0.41670987	0.37202872	P4hb	1
GO:0034713	type I transforming growth factor beta receptor binding	1/135	12/14323	0.08333333	8.84135802	2.65066623	0.107461	0.41670987	0.37202872	Smad2	1
GO:0034987	immunoglobulin receptor binding	1/135	12/14323	0.08333333	8.84135802	2.65066623	0.107461	0.41670987	0.37202872	Ighg2c	1
GO:0005543	phospholipid binding	7/135	428/14323	0.01635514	1.7352198	1.50632239	0.11008206	0.42218283	0.37691485	Rlbp1/Snx21/Scin/Sh3pxd2a/Snx14/Snx18/Wdr45b	7
GO:0008252	nucleotidase activity	1/135	13/14323	0.07692308	8.16125356	2.51970118	0.11588059	0.42419182	0.37870843	Nt5c3b	1
GO:0050700	CARD domain binding	1/135	13/14323	0.07692308	8.16125356	2.51970118	0.11588059	0.42419182	0.37870843	Card11	1
GO:0000774	adenyl-nucleotide exchange factor activity	1/135	14/14323	0.07142857	7.57830688	2.40204774	0.12422134	0.42419182	0.37870843	Bag5	1
GO:0005313	L-glutamate transmembrane transporter activity	1/135	14/14323	0.07142857	7.57830688	2.40204774	0.12422134	0.42419182	0.37870843	Slc17a8	1
GO:0015149	hexose transmembrane transporter activity	1/135	14/14323	0.07142857	7.57830688	2.40204774	0.12422134	0.42419182	0.37870843	Slc5a3	1
GO:0015929	hexosaminidase activity	1/135	14/14323	0.07142857	7.57830688	2.40204774	0.12422134	0.42419182	0.37870843	Hexa	1
GO:0034450	ubiquitin-ubiquitin ligase activity	1/135	14/14323	0.07142857	7.57830688	2.40204774	0.12422134	0.42419182	0.37870843	Itch	1
GO:0055056	D-glucose transmembrane transporter activity	1/135	14/14323	0.07142857	7.57830688	2.40204774	0.12422134	0.42419182	0.37870843	Slc5a3	1
GO:0070990	snRNP binding	1/135	14/14323	0.07142857	7.57830688	2.40204774	0.12422134	0.42419182	0.37870843	Prpf31	1
GO:0008080	N-acetyltransferase activity	2/135	65/14323	0.03076923	3.26450142	1.78487621	0.12516913	0.42419182	0.37870843	Sat1/Nat9	2
GO:0015079	potassium ion transmembrane transporter activity	3/135	131/14323	0.02290076	2.42968617	1.60347685	0.12652937	0.42419182	0.37870843	Kcnn1/Kcnk13/Kcnip2	3
GO:0005178	integrin binding	3/135	133/14323	0.02255639	2.39314954	1.57449211	0.13074266	0.42419182	0.37870843	Calr/Mmp14/P4hb	3
GO:0004857	enzyme inhibitor activity	5/135	285/14323	0.01754386	1.86133853	1.43268863	0.13215221	0.42419182	0.37870843	Hsp90b1/Dnajc3/Hnrnpc/Smr2l/Arpp19	5
GO:0015145	monosaccharide transmembrane transporter activity	1/135	15/14323	0.06666667	7.07308642	2.29548127	0.13248398	0.42419182	0.37870843	Slc5a3	1
GO:0022840	leak channel activity	1/135	15/14323	0.06666667	7.07308642	2.29548127	0.13248398	0.42419182	0.37870843	Kcnk13	1
GO:0022842	narrow pore channel activity	1/135	15/14323	0.06666667	7.07308642	2.29548127	0.13248398	0.42419182	0.37870843	Kcnk13	1
GO:0042813	Wnt receptor activity	1/135	15/14323	0.06666667	7.07308642	2.29548127	0.13248398	0.42419182	0.37870843	Fzd8	1
GO:1990226	histone methyltransferase binding	1/135	15/14323	0.06666667	7.07308642	2.29548127	0.13248398	0.42419182	0.37870843	Cbx1	1
GO:0000287	magnesium ion binding	4/135	212/14323	0.01886792	2.00181691	1.43345981	0.14032316	0.42690055	0.38112672	Srpk1/Enoph1/Nt5c3b/Oxsr1	4
GO:0005315	phosphate transmembrane transporter activity	1/135	16/14323	0.0625	6.63101852	2.19826876	0.14066924	0.42690055	0.38112672	Slc17a8	1
GO:0035613	RNA stem-loop binding	1/135	16/14323	0.0625	6.63101852	2.19826876	0.14066924	0.42690055	0.38112672	Mettl16	1
GO:0043176	amine binding	1/135	16/14323	0.0625	6.63101852	2.19826876	0.14066924	0.42690055	0.38112672	Sat1	1
GO:0051119	sugar transmembrane transporter activity	1/135	16/14323	0.0625	6.63101852	2.19826876	0.14066924	0.42690055	0.38112672	Slc5a3	1
GO:0070411	I-SMAD binding	1/135	16/14323	0.0625	6.63101852	2.19826876	0.14066924	0.42690055	0.38112672	Smad2	1
GO:0005244	voltage-gated monoatomic ion channel activity	3/135	139/14323	0.02158273	2.28984812	1.49057812	0.14367224	0.43142196	0.38516333	Kcnn1/Kcnk13/Kcnip2	3
GO:0004842	ubiquitin-protein transferase activity	6/135	376/14323	0.01595745	1.693026	1.32834591	0.14467474	0.43142196	0.38516333	Dtx4/Obi1/Pja1/Itch/Ube3c/Arih1	6
GO:0022832	voltage-gated channel activity	3/135	140/14323	0.02142857	2.27349206	1.47701307	0.1458676	0.43142196	0.38516333	Kcnn1/Kcnk13/Kcnip2	3
GO:0061630	ubiquitin protein ligase activity	5/135	297/14323	0.01683502	1.78613293	1.33541247	0.149345	0.43799499	0.39103157	Dtx4/Pja1/Itch/Ube3c/Arih1	5
GO:0034185	apolipoprotein binding	1/135	18/14323	0.05555556	5.89423868	2.02668193	0.15681047	0.44233836	0.39490923	Hspd1	1
GO:0042625	ATPase-coupled ion transmembrane transporter activity	1/135	18/14323	0.05555556	5.89423868	2.02668193	0.15681047	0.44233836	0.39490923	Atp6v0b	1
GO:0044769	ATPase activity, coupled to transmembrane movement of ions, rotational mechanism	1/135	18/14323	0.05555556	5.89423868	2.02668193	0.15681047	0.44233836	0.39490923	Atp6v0b	1
GO:0046961	proton-transporting ATPase activity, rotational mechanism	1/135	18/14323	0.05555556	5.89423868	2.02668193	0.15681047	0.44233836	0.39490923	Atp6v0b	1
GO:0015294	solute:monoatomic cation symporter activity	2/135	77/14323	0.02597403	2.75574796	1.50684712	0.164074	0.44233836	0.39490923	Slc5a3/Slc17a8	2
GO:0005242	inward rectifier potassium channel activity	1/135	19/14323	0.05263158	5.58401559	1.95030379	0.16476787	0.44233836	0.39490923	Kcnn1	1
GO:0005537	D-mannose binding	1/135	19/14323	0.05263158	5.58401559	1.95030379	0.16476787	0.44233836	0.39490923	Lman2	1
GO:0010314	phosphatidylinositol-5-phosphate binding	1/135	19/14323	0.05263158	5.58401559	1.95030379	0.16476787	0.44233836	0.39490923	Sh3pxd2a	1
GO:0015172	acidic amino acid transmembrane transporter activity	1/135	19/14323	0.05263158	5.58401559	1.95030379	0.16476787	0.44233836	0.39490923	Slc17a8	1
GO:0032794	GTPase activating protein binding	1/135	19/14323	0.05263158	5.58401559	1.95030379	0.16476787	0.44233836	0.39490923	Gapvd1	1
GO:0050699	WW domain binding	1/135	19/14323	0.05263158	5.58401559	1.95030379	0.16476787	0.44233836	0.39490923	Ndfip2	1
GO:0008757	S-adenosylmethionine-dependent methyltransferase activity	3/135	149/14323	0.02013423	2.13616704	1.35986951	0.16610406	0.4425215	0.39507273	Mettl16/Tfb1m/Mettl14	3
GO:0005227	calcium-activated cation channel activity	1/135	20/14323	0.05	5.30481481	1.87916118	0.17265073	0.44965383	0.40144031	Kcnn1	1
GO:0140517	protein-RNA adaptor activity	1/135	20/14323	0.05	5.30481481	1.87916118	0.17265073	0.44965383	0.40144031	Hnrnpc	1
GO:1904047	S-adenosyl-L-methionine binding	1/135	20/14323	0.05	5.30481481	1.87916118	0.17265073	0.44965383	0.40144031	Tfb1m	1
GO:0016410	N-acyltransferase activity	2/135	80/14323	0.025	2.65240741	1.44567116	0.17411422	0.44965383	0.40144031	Sat1/Nat9	2
GO:0061659	ubiquitin-like protein ligase activity	5/135	314/14323	0.01592357	1.68943147	1.20492631	0.17522327	0.44965383	0.40144031	Dtx4/Pja1/Itch/Ube3c/Arih1	5
GO:0038191	neuropilin binding	1/135	21/14323	0.04761905	5.05220459	1.81263664	0.18045973	0.45638004	0.4074453	Sema3e	1
GO:0070034	telomerase RNA binding	1/135	21/14323	0.04761905	5.05220459	1.81263664	0.18045973	0.45638004	0.4074453	Hnrnpc	1
GO:0005501	retinoid binding	1/135	22/14323	0.04545455	4.82255892	1.75021118	0.18819556	0.4610663	0.41162909	Rlbp1	1
GO:0008266	poly(U) RNA binding	1/135	22/14323	0.04545455	4.82255892	1.75021118	0.18819556	0.4610663	0.41162909	Hnrnpc	1
GO:0015144	carbohydrate transmembrane transporter activity	1/135	22/14323	0.04545455	4.82255892	1.75021118	0.18819556	0.4610663	0.41162909	Slc5a3	1
GO:0030215	semaphorin receptor binding	1/135	22/14323	0.04545455	4.82255892	1.75021118	0.18819556	0.4610663	0.41162909	Sema3e	1
GO:0000979	RNA polymerase II core promoter sequence-specific DNA binding	1/135	23/14323	0.04347826	4.61288245	1.69144492	0.19585892	0.4610663	0.41162909	Tbpl1	1
GO:0019840	isoprenoid binding	1/135	23/14323	0.04347826	4.61288245	1.69144492	0.19585892	0.4610663	0.41162909	Rlbp1	1
GO:0048156	tau protein binding	1/135	23/14323	0.04347826	4.61288245	1.69144492	0.19585892	0.4610663	0.41162909	Smad2	1
GO:0044325	transmembrane transporter binding	3/135	162/14323	0.01851852	1.96474623	1.20456923	0.19666604	0.4610663	0.41162909	Slc5a3/Kcnip2/Ppp2cb	3
GO:0008233	peptidase activity	6/135	416/14323	0.01442308	1.53023504	1.07054886	0.19936868	0.4610663	0.41162909	Mmp14/Pgpep1/Pdia3/Lta4h/Lap3/Trhde	6
GO:0016407	acetyltransferase activity	2/135	88/14323	0.02272727	2.41127946	1.29533992	0.20132605	0.4610663	0.41162909	Sat1/Nat9	2
GO:0019888	protein phosphatase regulator activity	2/135	88/14323	0.02272727	2.41127946	1.29533992	0.20132605	0.4610663	0.41162909	Hsp90b1/Arpp19	2
GO:0022839	monoatomic ion-gated channel activity	1/135	24/14323	0.04166667	4.42067901	1.63596211	0.20345046	0.4610663	0.41162909	Kcnn1	1
GO:0030742	GTP-dependent protein binding	1/135	24/14323	0.04166667	4.42067901	1.63596211	0.20345046	0.4610663	0.41162909	Rab38	1
GO:0042288	MHC class I protein binding	1/135	24/14323	0.04166667	4.42067901	1.63596211	0.20345046	0.4610663	0.41162909	Pdia3	1
GO:0050750	low-density lipoprotein particle receptor binding	1/135	24/14323	0.04166667	4.42067901	1.63596211	0.20345046	0.4610663	0.41162909	Hsp90b1	1
GO:0070412	R-SMAD binding	1/135	24/14323	0.04166667	4.42067901	1.63596211	0.20345046	0.4610663	0.41162909	Smad2	1
GO:0008170	N-methyltransferase activity	2/135	90/14323	0.02222222	2.35769547	1.26032784	0.20820817	0.46880421	0.41853731	Mettl16/Tfb1m	2
GO:0005160	transforming growth factor beta receptor binding	1/135	25/14323	0.04	4.24385185	1.58343959	0.21097086	0.47188325	0.42128621	Smad2	1
GO:0008187	poly-pyrimidine tract binding	1/135	26/14323	0.03846154	4.08062678	1.53359764	0.21842078	0.47188325	0.42128621	Hnrnpc	1
GO:0031435	mitogen-activated protein kinase kinase kinase binding	1/135	26/14323	0.03846154	4.08062678	1.53359764	0.21842078	0.47188325	0.42128621	Sash1	1
GO:0070325	lipoprotein particle receptor binding	1/135	26/14323	0.03846154	4.08062678	1.53359764	0.21842078	0.47188325	0.42128621	Hsp90b1	1
GO:0071813	lipoprotein particle binding	1/135	26/14323	0.03846154	4.08062678	1.53359764	0.21842078	0.47188325	0.42128621	Hspd1	1
GO:0071814	protein-lipid complex binding	1/135	26/14323	0.03846154	4.08062678	1.53359764	0.21842078	0.47188325	0.42128621	Hspd1	1
GO:0016616	oxidoreductase activity, acting on the CH-OH group of donors, NAD or NADP as acceptor	2/135	95/14323	0.02105263	2.23360624	1.17672386	0.22551399	0.47188325	0.42128621	Dhrs3/Akr1a1	2
GO:0015662	P-type ion transporter activity	1/135	27/14323	0.03703704	3.92949246	1.48619271	0.22580087	0.47188325	0.42128621	Atp6v0b	1
GO:0043325	phosphatidylinositol-3,4-bisphosphate binding	1/135	27/14323	0.03703704	3.92949246	1.48619271	0.22580087	0.47188325	0.42128621	Sh3pxd2a	1
GO:0045499	chemorepellent activity	1/135	27/14323	0.03703704	3.92949246	1.48619271	0.22580087	0.47188325	0.42128621	Sema3e	1

GO:0051861 glycolipid binding	1/135	27/14323	0.03703704	3.92949246	1.48619271	0.22580087	0.47188325	0.42128621	Manf	1
GO:0140358 P-type transmembrane transporter activity	1/135	27/14323	0.03703704	3.92949246	1.48619271	0.22580087	0.47188325	0.42128621	Atp6v0b	1
GO:0046873 metal ion transmembrane transporter activity	5/135	346/14323	0.01445087	1.53318347	0.97930027	0.22805267	0.47288288	0.42217866	Slc5a3/Kcnn1/Kcnk13/Slc17a8/Kcnip2	5
GO:0019208 phosphatase regulator activity	2/135	96/14323	0.02083333	2.21033951	1.16063133	0.22898913	0.47288288	0.42217866	Hsp90b1/Arpp19	2
GO:0017147 Wnt-protein binding	1/135	28/14323	0.03571429	3.78915344	1.44101158	0.23311179	0.47576616	0.42475278	Fzd8	1
GO:0070273 phosphatidylinositol-4-phosphate binding	1/135	28/14323	0.03571429	3.78915344	1.44101158	0.23311179	0.47576616	0.42475278	Sh3pxd2a	1
GO:0008168 methyltransferase activity	3/135	179/14323	0.01675978	1.77815022	1.02190839	0.23845292	0.48208969	0.43039828	Mettl16/Tfb1m/Mettl14	3
GO:0001530 lipopolysaccharide binding	1/135	29/14323	0.03448276	3.65849298	1.39786669	0.24035417	0.48208969	0.43039828	Hspd1	1
GO:0043425 bHLH transcription factor binding	1/135	29/14323	0.03448276	3.65849298	1.39786669	0.24035417	0.48208969	0.43039828	Bmal1	1
GO:0022890 inorganic cation transmembrane transporter activity	6/135	446/14323	0.01345291	1.42730443	0.8942628	0.24472706	0.48805568	0.43572457	Slc5a3/Kcnn1/Kcnk13/Slc17a8/Kcnip2/Atp6v0b	6
GO:0140142 nucleocytoplasmic carrier activity	1/135	30/14323	0.03333333	3.53654321	1.35659223	0.24752866	0.49083809	0.43820864	Calr	1
GO:0016614 oxidoreductase activity, acting on CH-OH group of donors	2/135	102/14323	0.01960784	2.08031954	1.06805933	0.24990585	0.49275221	0.43991752	Dhrs3/Akr1a1	2
GO:0042887 amide transmembrane transporter activity	1/135	31/14323	0.03225806	3.42246117	1.31704101	0.25463589	0.49646887	0.44323566	Slc33a1	1
GO:0050681 nuclear androgen receptor binding	1/135	31/14323	0.03225806	3.42246117	1.31704101	0.25463589	0.49646887	0.44323566	Calr	1
GO:0016741 transferase activity, transferring one-carbon groups	3/135	188/14323	0.01595745	1.693026	0.93301511	0.26116944	0.49781926	0.44444127	Mettl16/Tfb1m/Mettl14	3
GO:0004180 carboxypeptidase activity	1/135	32/14323	0.03125	3.31550926	1.27908183	0.26167649	0.49781926	0.44444127	Lap3	1
GO:0042287 MHC protein binding	1/135	32/14323	0.03125	3.31550926	1.27908183	0.26167649	0.49781926	0.44444127	Pdia3	1
GO:0097718 disordered domain specific binding	1/135	32/14323	0.03125	3.31550926	1.27908183	0.26167649	0.49781926	0.44444127	Smad2	1
GO:0016747 acyltransferase activity, transferring groups other than amino-acyl groups	3/135	189/14323	0.01587302	1.6840682	0.92343405	0.2637121	0.49781926	0.44444127	Sat1/Nat9/Elovl7	3
GO:0015293 symporter activity	2/135	106/14323	0.01886792	2.00181691	1.00982342	0.263887	0.49781926	0.44444127	Slc5a3/Slc17a8	2
GO:0003755 peptidyl-prolyl cis-trans isomerase activity	1/135	33/14323	0.03030303	3.21503928	1.24259733	0.26865106	0.50138621	0.44762575	Fkbp2	1
GO:0005484 SNAP receptor activity	1/135	33/14323	0.03030303	3.21503928	1.24259733	0.26865106	0.50138621	0.44762575	Sec22b	1
GO:0030165 PDZ domain binding	2/135	109/14323	0.01834862	1.94672103	0.96779918	0.27437696	0.50543186	0.45123761	Arhgef16/Fzd8	2
GO:0004715 non-membrane spanning protein tyrosine kinase activity	1/135	34/14323	0.02941176	3.1204793	1.20748212	0.27556024	0.50543186	0.45123761	Stk16	1
GO:0022853 active monoatomic ion transmembrane transporter activity	3/135	194/14323	0.01546392	1.64066438	0.87636264	0.27647054	0.50543186	0.45123761	Slc5a3/Slc17a8/Atp6v0b	3
GO:0048018 receptor ligand activity	4/135	284/14323	0.01408451	1.49431403	0.82073348	0.27971656	0.50543186	0.45123761	Manf/Dand5/Tafa1/Sema3e	4
GO:0016251 RNA polymerase II general transcription initiation factor activity	1/135	35/14323	0.02857143	3.03132275	1.17364124	0.28240462	0.50543186	0.45123761	Tbpl1	1
GO:0031624 ubiquitin conjugating enzyme binding	1/135	35/14323	0.02857143	3.03132275	1.17364124	0.28240462	0.50543186	0.45123761	Arih1	1
GO:0042379 chemokine receptor binding	1/135	35/14323	0.02857143	3.03132275	1.17364124	0.28240462	0.50543186	0.45123761	Itch	1
GO:0051059 NF-kappaB binding	1/135	35/14323	0.02857143	3.03132275	1.17364124	0.28240462	0.50543186	0.45123761	Brms1	1
GO:0005085 guanyl-nucleotide exchange factor activity	3/135	197/14323	0.01522843	1.61567964	0.84876299	0.2841567	0.5059729	0.45172064	Arhgef16/Gapvd1/Ric8b	3
GO:0008375 acetylglucosaminyltransferase activity	1/135	36/14323	0.02777778	2.94711934	1.14098892	0.28918481	0.51159162	0.4567369	Hexa	1
GO:0030546 signaling receptor activator activity	4/135	289/14323	0.01384083	1.46846085	0.78476498	0.29024395	0.51159162	0.4567369	Manf/Dand5/Tafa1/Sema3e	4
GO:0008194 UDP-glycosyltransferase activity	2/135	114/14323	0.01754386	1.86133853	0.90064299	0.29184352	0.51182607	0.45694621	Has2/Hexa	2
GO:0008324 monoatomic cation transmembrane transporter activity	6/135	477/14323	0.01257862	1.33454461	0.72486844	0.29444107	0.51377907	0.4586898	Slc5a3/Kcnn1/Kcnk13/Slc17a8/Kcnip2/Atp6v0b	6
GO:0070888 E-box binding	1/135	37/14323	0.02702703	2.86746747	1.10944739	0.29590141	0.51377907	0.4586898	Bmal1	1
GO:0008514 organic anion transmembrane transporter activity	3/135	204/14323	0.01470588	1.56023965	0.7861313	0.30215818	0.51760636	0.46210672	Slc51a/Slc17a8/Slc33a1	3
GO:0001727 lipid kinase activity	1/135	38/14323	0.02631579	2.7920078	1.07894598	0.30255501	0.51760636	0.46210672	Dgkk	1
GO:0170055 lipid transmembrane transporter activity	1/135	38/14323	0.02631579	2.7920078	1.07894598	0.30255501	0.51760636	0.46210672	Slc51a	1
GO:0015081 sodium ion transmembrane transporter activity	2/135	118/14323	0.01694915	1.79824231	0.84930417	0.30578126	0.52057395	0.46475612	Slc5a3/Slc17a8	2
GO:0016620 oxidoreductase activity, acting on the aldehyde or oxo group of donors, NAD or NADP as acceptor	1/135	39/14323	0.02564103	2.72041785	1.04942028	0.30914619	0.5212175	0.46533066	Far2	1
GO:0043531 ADP binding	1/135	39/14323	0.02564103	2.72041785	1.04942028	0.30914619	0.5212175	0.46533066	Chordc1	1
GO:0051721 protein phosphatase 2A binding	1/135	40/14323	0.025	2.65240741	1.02081147	0.31567555	0.52966714	0.4728743	Arpp19	1
GO:0004812 aminoacyl-tRNA ligase activity	1/135	41/14323	0.02439024	2.58771454	0.99306566	0.32214364	0.53023236	0.47337891	Yars2	1
GO:0005246 calcium channel regulator activity	1/135	41/14323	0.02439024	2.58771454	0.99306566	0.32214364	0.53023236	0.47337891	Rrad	1
GO:0016875 ligase activity, forming carbon-oxygen bonds	1/135	41/14323	0.02439024	2.58771454	0.99306566	0.32214364	0.53023236	0.47337891	Yars2	1
GO:0044390 ubiquitin-like protein conjugating enzyme binding	1/135	41/14323	0.02439024	2.58771454	0.99306566	0.32214364	0.53023236	0.47337891	Arih1	1
GO:0140678 molecular function inhibitor activity	5/135	399/14323	0.01253133	1.32952752	0.65118481	0.32360886	0.53023236	0.47337891	Hsp90b1/Dnajc3/Hnrnpc/Smr2l/Arpp19	5
GO:0015605 organophosphate ester transmembrane transporter activity	1/135	42/14323	0.02380952	2.52610229	0.96613343	0.32855105	0.53581456	0.47836257	Slc33a1	1
GO:0044024 histone H2AS1 kinase activity	3/135	216/14323	0.01388889	1.47355967	0.68405714	0.33312985	0.53825147	0.48053819	Srpk1/Stk16/Oxsr1	3
GO:0140995 histone H2A kinase activity	3/135	216/14323	0.01388889	1.47355967	0.68405714	0.33312985	0.53825147	0.48053819	Srpk1/Stk16/Oxsr1	3
GO:0140223 general transcription initiation factor activity	1/135	43/14323	0.02325581	2.46735573	0.93996929	0.33489834	0.5386153	0.480863	Tbpl1	1
GO:0019902 phosphatase binding	3/135	218/14323	0.01376147	1.46004077	0.66764585	0.33829368	0.54158024	0.48351004	Hsp90b1/Smad2/Arpp19	3
GO:0140313 molecular sequestering activity	1/135	44/14323	0.02272727	2.41127946	0.91453134	0.34118606	0.5437166	0.48541733	Calr	1
GO:0002020 protease binding	2/135	130/14323	0.01538462	1.63225071	0.70636965	0.34723505	0.54863239	0.48980603	Sh3pxd2a/Sri	2
GO:0001046 core promoter sequence-specific DNA binding	1/135	45/14323	0.02222222	2.35769547	0.88978088	0.34741478	0.54863239	0.48980603	Tbpl1	1
GO:0016830 carbon-carbon lyase activity	1/135	46/14323	0.02173913	2.30644122	0.86568211	0.35358504	0.54949499	0.49057613	Hmgcll1	1
GO:0016903 oxidoreductase activity, acting on the aldehyde or oxo group of donors	1/135	46/14323	0.02173913	2.30644122	0.86568211	0.35358504	0.54949499	0.49057613	Far2	1
GO:0060590 ATPase regulator activity	1/135	46/14323	0.02173913	2.30644122	0.86568211	0.35358504	0.54949499	0.49057613	Bag5	1
GO:0030545 signaling receptor regulator activity	4/135	319/14323	0.01253918	1.33036108	0.58205805	0.35425895	0.54949499	0.49057613	Manf/Dand5/Tafa1/Sema3e	4
GO:0035173 histone kinase activity	3/135	226/14323	0.01327434	1.40835792	0.60358714	0.35892107	0.54999582	0.49102326	Srpk1/Stk16/Oxsr1	3
GO:0015932 nucleobase-containing compound transmembrane transporter activity	1/135	47/14323	0.0212766	2.25736801	0.84220186	0.35969739	0.54999582	0.49102326	Slc33a1	1
GO:0019829 ATPase-coupled monoatomic cation transmembrane transporter activity	1/135	47/14323	0.0212766	2.25736801	0.84220186	0.35969739	0.54999582	0.49102326	Atp6v0b	1
GO:0016705 oxidoreductase activity, acting on paired donors, with incorporation or reduction of molecular oxygen	2/135	134/14323	0.01492537	1.58352681	0.66198088	0.36088551	0.54999582	0.49102326	Degs1/P4hb	2
GO:0005216 monoatomic ion channel activity	4/135	325/14323	0.01230769	1.30580057	0.54394515	0.3671326	0.55708381	0.49735126	Kcnn1/Kcnk13/Slc17a8/Kcnip2	4
GO:0003823 antigen binding	1/135	49/14323	0.02040816	2.16523054	0.79697588	0.37175051	0.55926236	0.49929621	Ighg2c	1
GO:0022836 gated channel activity	3/135	231/14323	0.01298701	1.37787398	0.56477412	0.37177326	0.55926236	0.49929621	Kcnn1/Kcnk13/Kcnip2	3
GO:0016597 amino acid binding	1/135	50/14323	0.02	2.12192593	0.77517487	0.37769234	0.56572801	0.50506859	Yars2	1
GO:0005254 chloride channel activity	1/135	51/14323	0.01960784	2.08031954	0.7538814	0.38357839	0.57104579	0.50981618	Slc17a8	1
GO:0008234 cysteine-type peptidase activity	2/135	141/14323	0.0141844	1.504912	0.58771211	0.38451508	0.57104579	0.50981618	Pgpep1/Trhde	2
GO:1901682 sulfur compound transmembrane transporter activity	1/135	52/14323	0.01923077	2.04031339	0.73307226	0.38940917	0.57517024	0.51349839	Slc33a1	1
GO:0061629 RNA polymerase II-specific DNA-binding transcription factor binding	4/135	336/14323	0.01190476	1.26305115	0.47594381	0.39069892	0.57517024	0.51349839	Bmal1/Calr/Smad2/Brms1	4
GO:0016791 phosphatase activity	3/135	239/14323	0.0125523	1.33175267	0.5044969	0.39223644	0.57517024	0.51349839	Ppp2cb/Enoph1/Nt5c3b	3
GO:0046943 carboxylic acid transmembrane transporter activity	2/135	144/14323	0.01388889	1.47355967	0.55711042	0.39452946	0.57611206	0.51433923	Slc51a/Slc17a8	2
GO:0005342 organic acid transmembrane transporter activity	2/135	145/14323	0.0137931	1.46339719	0.54706388	0.39785145	0.57854232	0.5165089	Slc51a/Slc17a8	2
GO:0140098 catalytic activity, acting on RNA	4/135	343/14323	0.01166181	1.23727459	0.43386322	0.40564133	0.5874225	0.52443692	Mettl16/Tfb1m/Mettl14/Yars2	4
GO:0008028 monocarboxylic acid transmembrane transporter activity	1/135	56/14323	0.01785714	1.89457672	0.65426457	0.41218988	0.59443913	0.5307012	Slc51a	1
GO:0016758 hexosyltransferase activity	2/135	150/14323	0.01333333	1.41461728	0.49793226	0.41433403	0.59507233	0.5312665	Has2/Hexa	2
GO:0005080 protein kinase C binding	1/135	58/14323	0.01724138	1.82924649	0.61726248	0.42326182	0.60540318	0.54048964	Pkp2	1
GO:0016706 2-oxoglutarate-dependent dioxygenase activity	1/135	59/14323	0.01694915	1.79824231	0.59930543	0.42871991	0.61070714	0.54522489	P4hb	1
GO:0015179 L-amino acid transmembrane transporter activity	1/135	60/14323	0.01666667	1.7682716	0.58169201	0.43412673	0.61589523	0.5498567	Slc17a8	1
GO:0005261 monoatomic cation channel activity	3/135	257/14323	0.01167315	1.23847817	0.37630328	0.43760977	0.61832311	0.55202426	Kcnn1/Kcnk13/Kcnip2	3
GO:0015267 channel activity	4/135	362/14323	0.01104972	1.17233477	0.3239493	0.44581553	0.62485791	0.55785837	Kcnn1/Kcnk13/Slc17a8/Kcnip2	4
GO:0022803 passive transmembrane transporter activity	4/135	362/14323	0.01104972	1.17233477	0.3239493	0.44581553	0.62485791	0.55785837	Kcnn1/Kcnk13/Slc17a8/Kcnip2	4
GO:0005253 monoatomic anion channel activity	1/135	64/14323	0.015625	1.65775463	0.51441959	0.45525074	0.63048614	0.56288312	Slc17a8	1
GO:0030145 manganese ion binding	1/135	64/14323	0.015625	1.65775463	0.51441959	0.45525074	0.63048614	0.56288312	Lap3	1
GO:0042562 hormone binding	1/135	64/14323	0.015625	1.65775463	0.51441959	0.45525074	0.63048614	0.56288312	Calr	1
GO:0140297 DNA-binding transcription factor binding	5/135	471/14323	0.01061571	1.12628765	0.27184617	0.45812147	0.63195413	0.5641937	Bmal1/Calr/Smad2/Sri/Brms1	5
GO:0004553 hydrolase activity, hydrolyzing O-glycosyl compounds	1/135	67/14323	0.01492537	1.58352681	0.46698991	0.47057835	0.64658206	0.57725318	Hexa	1
GO:0017018 myosin phosphatase activity	1/135	68/14323	0.01470588	1.56023965	0.45170284	0.47559181	0.64808249	0.57859272	Ppp2cb	1
GO:0019905 syntaxin binding	1/135	68/14323	0.01470588	1.56023965	0.45170284	0.47559181	0.64808249	0.57859272	Sec22b	1
GO:0003725 double-stranded RNA binding	1/135	69/14323	0.01449275	1.53762748	0.43666236	0.48055815	0.64808249	0.57859272	Hspd1	1
GO:0048029 monosaccharide binding	1/135	69/14323	0.01449275	1.53762748	0.43666236	0.48055815	0.64808249	0.57859272	Lman2	1
GO:0019903 protein phosphatase binding	2/135	171/14323	0.01169591	1.24089235	0.30911515	0.4809552	0.64808249	0.57859272	Hsp90b1/Arpp19	2
GO:0005126 cytokine receptor binding	2/135	172/14323	0.01162791	1.23367786	0.30074358	0.48401303	0.64916379	0.57955808	Smad2/Itch	2
GO:0002039 p53 binding	1/135	70/14323	0.01428571	1.51566138	0.42186025	0.48547779	0.64916379	0.57955808	Hspd1	1
GO:0004860 protein kinase inhibitor activity	1/135	71/14323	0.01408451	1.49431403	0.40728868	0.49035119	0.65317773	0.58314163	Dnajc3	1
GO:0019843 rRNA binding	1/135	72/14323	0.01388889	1.47355967	0.39294016	0.49517876	0.65461132	0.58442151	Mrps6	1
GO:0030971 receptor tyrosine kinase binding	1/135	72/14323	0.01388889	1.47355967	0.39294016	0.49517876	0.65461132	0.58442151	Arhgef16	1
GO:0004674 protein serine/threonine kinase activity	4/135	388/14323	0.01030928	1.09377625	0.18266838	0.49938219	0.65723752	0.58676612	Srpk1/Stk16/Pdk3/Oxsr1	4
GO:0005516 calmodulin binding	2/135	178/14323	0.01123596	1.19209322	0.25155253	0.50212625	0.65723752	0.58676612	Kcnn1/Rrad	2
GO:0019210 kinase inhibitor activity	1/135	74/14323	0.01351351	1.43373373	0.36488415	0.50469816	0.65723752	0.58676612	Dnajc3	1
GO:0035064 methylated histone binding	1/135	74/14323	0.01351351	1.43373373	0.36488415	0.50469816	0.65723752	0.58676612	Cbx1	1
GO:0000049 tRNA binding	1/135	76/14323	0.01315789	1.3960039	0.33763905	0.51403935	0.66199163	0.59101048	Yars2	1
GO:0004222 metalloendopeptidase activity	1/135	76/14323	0.01315789	1.3960039	0.33763905	0.51403935	0.66199163	0.59101048	Mmp14	1
GO:0140034 methylation-dependent protein binding	1/135	76/14323	0.01315789	1.3960039	0.33763905	0.51403935	0.66199163	0.59101048	Cbx1	1
GO:0046332 SMAD binding	1/135	77/14323	0.01298701	1.37787398	0.32430527	0.51864415	0.66211911	0.59112428	Smad2	1
GO:0051213 dioxygenase activity	1/135	77/14323	0.01298701	1.37787398	0.32430527	0.51864415	0.66211911	0.59112428	P4hb	1
GO:0140030 modification-dependent protein binding	2/135	184/14323	0.01086957	1.15322061	0.2040445	0.5198299	0.66211911	0.59112428	Hspd1/Cbx1	2
GO:0043178 alcohol binding	1/135	78/14323	0.01282051	1.36020893	0.31115635	0.52320565	0.66399553	0.59279951	Rlbp1	1
GO:0003779 actin binding	4/135	402/14323	0.00995025	1.05568454	0.11046395	0.52731903	0.66489443	0.59360203	Scin/P4hb/Msrb2/Fbxo25	4
GO:0019003 GDP binding	1/135	79/14323	0.01265823	1.34299109	0.29818688	0.52772423	0.66489443	0.59360203	Rab9b	1
GO:0015108 chloride transmembrane transporter activity	1/135	80/14323	0.0125	1.3262037	0.28539166	0.53220031	0.66572727	0.59434556	Slc17a8	1
GO:0140375 immune receptor activity	1/135	80/14323	0.0125	1.3262037	0.28539166	0.53220031	0.66572727	0.59434556	Crl1	1
GO:0004722 protein serine/threonine phosphatase activity	1/135	82/14323	0.01219512	1.29385727	0.26030425	0.54102652	0.67435091	0.60204455	Ppp2cb	1
GO:0003727 single-stranded RNA binding	1/135	83/14323	0.01204819	1.27826863	0.24800271	0.54537744	0.67495293	0.60258201	Hnrnpc	1
GO:0042626 ATPase-coupled transmembrane transporter activity	1/135	83/14323	0.01204819	1.27826863	0.24800271	0.54537744	0.67495293	0.60258201	Atp6v0b	1
GO:0030695 GTPase regulator activity	4/135	414/14323	0.00966184	1.02508499	0.05052127	0.5506655	0.6766981	0.60414006	Arhgef16/Stard13/Gapvd1/Ric8b	4
GO:0060589 nucleoside-triphosphatase regulator activity	4/135	414/14323	0.00966184	1.02508499	0.05052127	0.5506655	0.6766981	0.60414006	Arhgef16/Stard13/Gapvd1/Ric8b	4
GO:0015171 amino acid transmembrane transporter activity	1/135	86/14323	0.01162791	1.23367786	0.21201456	0.55818606	0.68353311	0.61024219	Slc17a8	1
GO:0042277 peptide binding	2/135	200/14323	0.01	1.06096296	0.08468875	0.56496648	0.68891078	0.61504325	Calr/Trhde	2
GO:0099094 ligand-gated monoatomic cation channel activity	1/135	88/14323	0.01136364	1.20563973	0.188746	0.56652548	0.68891078	0.61504325	Kcnn1	1
GO:0004866 endopeptidase inhibitor activity	1/135	90/14323	0.01111111	1.17884774	0.1660216	0.57470864	0.69290855	0.61861237	Smr2l	1
GO:0003729 mRNA binding	3/135	316/14323	0.00949367	1.00724332	0.01270038	0.57545711	0.69290855	0.61861237	Calr/Hnrnpc/Mettl14	3
GO:0030246 carbohydrate binding	2/135	204/14323	0.00980392	1.04015977	0.0563524	0.57576928	0.69290855	0.61861237	Calr/Lman2	2
GO:1990756 ubiquitin-like ligase-substrate adaptor activity	1/135	91/14323	0.01098901	1.16589337	0.15485487	0.57874253	0.69293121	0.6186326	Klhl5	1
GO:0042578 phosphoric ester hydrolase activity	3/135	318/14323	0.00943396	1.00090846	0.00159803	0.57975906	0.69293121	0.6186326	Ppp2cb/Enoph1/Nt5c3b	3
GO:0008083 growth factor activity	1/135	92/14323	0.01086957	1.15322061	0.14381412	0.58273843	0.69411506	0.61968952	Manf	1
GO:0051117 ATPase binding	1/135	93/14323	0.01075269	1.14082039	0.13289624	0.58669672	0.6964529	0.62177668	Sdf2l1	1
GO:0140104 molecular carrier activity	1/135	94/14323	0.0106383	1.128684	0.12209825	0.59061772	0.6987308	0.62381034	Calr	1
GO:0030414 peptidase inhibitor activity	1/135	95/14323	0.01052632	1.11680312	0.11141725	0.59450181	0.70094977	0.62579138	Smr2l	1
GO:0031490 chromatin DNA binding	1/135	96/14323	0.01041667	1.10516975	0.10085047	0.59834931	0.7012557	0.6260645	Grhl3	1
GO:0106310 protein serine kinase activity	3/135	327/14323	0.00917431	0.97336052	-0.0475342	0.59877994	0.7012557	0.6260645	Srpk1/Stk16/Oxsr1	3
GO:0016757 glycosyltransferase activity	2/135	217/14323	0.00921659	0.97784605	-0.0320765	0.60953045	0.70628169	0.63055159	Has2/Hexa	2
GO:0015291 secondary active transmembrane transporter activity	2/135	218/14323	0.00917431	0.97336052	-0.0386612	0.6120419	0.70628169	0.63055159	Slc5a3/Slc17a8	2
GO:0008509 monoatomic anion transmembrane transporter activity	1/135	100/14323	0.01	1.06096296	0.0596731	0.6133803	0.70628169	0.63055159	Slc17a8	1
GO:0016798 hydrolase activity, acting on glycosyl bonds	1/135	100/14323	0.01	1.06096296	0.0596731	0.6133803	0.70628169	0.63055159	Hexa	1
GO:0003730 mRNA 3'-UTR binding	1/135	101/14323	0.00990099	1.05045838	0.04963886	0.61704997	0.70628169	0.63055159	Hnrnpc	1
GO:0140767 enzyme-substrate adaptor activity	1/135	101/14323	0.00990099	1.05045838	0.04963886	0.61704997	0.70628169	0.63055159	Klhl5	1
GO:0022804 active transmembrane transporter activity	3/135	336/14323	0.00892857	0.94728836	-0.0953722	0.61723758	0.70628169	0.63055159	Slc5a3/Slc17a8/Atp6v0b	3
GO:0015103 inorganic anion transmembrane transporter activity	1/135	103/14323	0.00970874	1.03006113	0.02986636	0.6242859	0.71201235	0.63566779	Slc17a8	1
GO:0008013 beta-catenin binding	1/135	104/14323	0.00961538	1.0201567	0.02012378	0.6278528	0.7130482	0.63659257	Cdh10	1
GO:0005096 GTPase activator activity	2/135	225/14323	0.00888889	0.94307819	-0.0839459	0.62928036	0.7130482	0.63659257	Stard13/Gapvd1	2
GO:1990782 protein tyrosine kinase binding	1/135	108/14323	0.00925926	0.98237311	-0.0179359	0.64178741	0.72085087	0.64355861	Arhgef16	1
GO:0005509 calcium ion binding	4/135	466/14323	0.00858369	0.91069782	-0.1911739	0.64436646	0.72085087	0.64355861	Calr/Kcnip2/Sri/Cdh10	4
GO:0061135 endopeptidase regulator activity	1/135	109/14323	0.00917431	0.97336052	-0.0272326	0.64518936	0.72085087	0.64355861	Smr2l	1
GO:0000149 SNARE binding	1/135	110/14323	0.00909091	0.96451178	-0.0364457	0.64855924	0.72085087	0.64355861	Sec22b	1
GO:0005506 iron ion binding	1/135	110/14323	0.00909091	0.96451178	-0.0364457	0.64855924	0.72085087	0.64355861	Calr	1
GO:0019842 vitamin binding	1/135	110/14323	0.00909091	0.96451178	-0.0364457	0.64855924	0.72085087	0.64355861	Rlbp1	1
GO:0043022 ribosome binding	1/135	112/14323	0.00892857	0.94728836	-0.0546275	0.65520398	0.72592441	0.64808814	Hspa5	1
GO:0001228 DNA-binding transcription activator activity, RNA polymerase II-specific	3/135	357/14323	0.00840336	0.89156552	-0.2023828	0.65806038	0.72678188	0.64885367	Bmal1/Grhl3/Smad2	3
GO:0004713 protein tyrosine kinase activity	1/135	115/14323	0.00869565	0.92257649	-0.0813136	0.66493796	0.72975896	0.65151154	Stk16	1
GO:0015399 primary active transmembrane transporter activity	1/135	115/14323	0.00869565	0.92257649	-0.0813136	0.66493796	0.72975896	0.65151154	Atp6v0b	1
GO:0015276 ligand-gated monoatomic ion channel activity	1/135	116/14323	0.00862069	0.91462324	-0.0900587	0.66812162	0.72977997	0.6515303	Kcnn1	1
GO:0001216 DNA-binding transcription activator activity	3/135	363/14323	0.00826446	0.8768289	-0.2318609	0.66913922	0.72977997	0.6515303	Bmal1/Grhl3/Smad2	3
GO:0015078 proton transmembrane transporter activity	1/135	118/14323	0.00847458	0.89912116	-0.1073318	0.67439912	0.73322522	0.65460614	Atp6v0b	1
GO:0042826 histone deacetylase binding	1/135	119/14323	0.00840336	0.89156552	-0.1158627	0.67749354	0.734302	0.65556746	Brms1	1
GO:0022834 ligand-gated channel activity	1/135	121/14323	0.00826446	0.8768289	-0.1327195	0.68359506	0.7374654	0.65839167	Kcnn1	1
GO:0140657 ATP-dependent activity	4/135	491/14323	0.00814664	0.86432828	-0.2983979	0.68463836	0.7374654	0.65839167	Hspa5/Hsp90b1/Hspd1/Atp6v0b	4
GO:0004672 protein kinase activity	4/135	497/14323	0.00804829	0.85389373	-0.3233753	0.69381917	0.74452239	0.66469198	Srpk1/Stk16/Pdk3/Oxsr1	4
GO:0005319 lipid transporter activity	1/135	125/14323	0.008	0.84877037	-0.1656481	0.69545644	0.74452239	0.66469198	Slc51a	1
GO:0016779 nucleotidyltransferase activity	1/135	127/14323	0.00787402	0.83540391	-0.1817383	0.70122054	0.74839746	0.66815156	Crppa	1
GO:0140101 catalytic activity, acting on a tRNA	1/135	134/14323	0.00746269	0.79176341	-0.2362336	0.72055517	0.76668827	0.68448116	Yars2	1
GO:0140993 histone modifying activity	3/135	404/14323	0.00742574	0.78784378	-0.4219399	0.73789336	0.78117545	0.69741497	Srpk1/Stk16/Oxsr1	3
GO:0031406 carboxylic acid binding	1/135	141/14323	0.0070922	0.752456	-0.2881385	0.73864727	0.78117545	0.69741497	Yars2	1
GO:0016922 nuclear receptor binding	1/135	142/14323	0.00704225	0.74715702	-0.2953587	0.74113512	0.78143854	0.69764985	Calr	1

GO:0061134	peptidase regulator activity	1/135	146/14323	0.00684932	0.72668696	-0.323782	0.75085361	0.78775287	0.70328713	Smr2l	1
GO:0031267	small GTPase binding	2/135	284/14323	0.00704225	0.74715702	-0.4198074	0.75163812	0.78775287	0.70328713	Arhgef16/Gapvd1	2
GO:0043177	organic acid binding	1/135	149/14323	0.00671141	0.71205568	-0.3446383	0.75790401	0.79194161	0.70702674	Yars2	1
GO:0004721	phosphoprotein phosphatase activity	1/135	155/14323	0.00645161	0.68449223	-0.3852386	0.77141609	0.80365438	0.71748362	Ppp2cb	1
GO:0003735	structural constituent of ribosome	1/135	164/14323	0.00609756	0.64692864	-0.4435844	0.79029305	0.82086986	0.7328532	Mrps6	1
GO:0016829	lyase activity	1/135	171/14323	0.00584795	0.62044618	-0.4870467	0.80389807	0.83252352	0.74325731	Hmgcll1	1
GO:0051020	GTPase binding	2/135	324/14323	0.00617284	0.65491541	-0.6128549	0.81350801	0.83998313	0.74991707	Arhgef16/Gapvd1	2
GO:0016887	ATP hydrolysis activity	2/135	342/14323	0.00584795	0.62044618	-0.6929876	0.83660459	0.86128319	0.76893327	Hspa5/Hsp90b1	2
GO:0051015	actin filament binding	1/135	201/14323	0.00497512	0.52784227	-0.6575748	0.85294807	0.87552611	0.78164901	Scin	1
GO:0019887	protein kinase regulator activity	1/135	210/14323	0.0047619	0.50522046	-0.704563	0.86513	0.88542631	0.79048767	Dnajc3	1
GO:0001664	G protein-coupled receptor binding	1/135	223/14323	0.0044843	0.47576814	-0.7696166	0.88097829	0.89608449	0.80000304	Itch	1
GO:0042393	histone binding	1/135	225/14323	0.00444444	0.47153909	-0.7793517	0.8832466	0.89608449	0.80000304	Cbx1	1
GO:1901681	sulfur compound binding	1/135	225/14323	0.00444444	0.47153909	-0.7793517	0.8832466	0.89608449	0.80000304	Tfb1m	1
GO:0004930	G protein-coupled receptor activity	1/135	231/14323	0.004329	0.45929133	-0.8081484	0.88979724	0.90011373	0.80360026	Fzd8	1
GO:0019207	kinase regulator activity	1/135	242/14323	0.00413223	0.43841445	-0.8594389	0.90087346	0.9086845	0.81125204	Dnajc3	1
GO:0004175	endopeptidase activity	1/135	255/14323	0.00392157	0.41606391	-0.9177563	0.91254659	0.91780622	0.81939569	Mmp14	1
GO:0015631	tubulin binding	1/135	353/14323	0.00283286	0.30055608	-1.2979267	0.96611936	0.96889557	0.86500705	Tppp3	1
GO:0003712	transcription coregulator activity	1/135	473/14323	0.00211416	0.22430507	-1.6734193	0.98948755	0.98948755	0.88339108	Bmal1	1

ID	Description	GeneRatio	BgRatio	RichFactor	FoldEnrichm	zScore	pvalue	p.adjust	qvalue	geneID	Count
GO:0055038	recycling endosome membrane	3/23	92/14612	0.0326087	20.7164461	7.53234785	0.00039045	0.0433403	0.03164725	Slc9b2/Rab8a/Rab13	3
GO:0030140	trans-Golgi network transport vesicle	2/23	34/14612	0.05882353	37.370844	8.43014396	0.00128948	0.07156612	0.05225786	Rab8a/Rab13	2
GO:0055037	recycling endosome	3/23	187/14612	0.01604278	10.1920484	5.02303633	0.00302504	0.11192633	0.08172904	Slc9b2/Rab8a/Rab13	3
GO:0016323	basolateral plasma membrane	3/23	235/14612	0.01276596	8.11026827	4.36291657	0.0057346	0.12507276	0.09132862	Slc6a13/Slc47a1/Slc9b2	3
GO:0009925	basal plasma membrane	3/23	258/14612	0.01162791	7.38725986	4.10987955	0.00742059	0.12507276	0.09132862	Slc6a13/Slc47a1/Slc9b2	3
GO:0045178	basal part of cell	3/23	275/14612	0.01090909	6.93059289	3.94208916	0.00883776	0.12507276	0.09132862	Slc6a13/Slc47a1/Slc9b2	3
GO:0005798	Golgi-associated vesicle	2/23	91/14612	0.02197802	13.9627329	4.9250442	0.008914	0.12507276	0.09132862	Rab8a/Rab13	2
GO:0008021	synaptic vesicle	3/23	277/14612	0.01083032	6.8805525	3.92328894	0.00901425	0.12507276	0.09132862	Slc9b2/Rab8a/Rab13	3
GO:0070382	exocytic vesicle	3/23	293/14612	0.01023891	6.50482267	3.77930486	0.01050145	0.12951788	0.09457446	Slc9b2/Rab8a/Rab13	3
GO:0097730	non-motile cilium	2/23	117/14612	0.01709402	10.8599034	4.25155309	0.01441071	0.139728	0.10202993	Rab8a/Arl3	2
GO:0032593	insulin-responsive compartment	1/23	10/14612	0.1	63.5304348	7.85373788	0.01563424	0.139728	0.10202993	Rab13	1
GO:0005766	primary lysosome	1/23	11/14612	0.09090909	57.7549407	7.47652591	0.01718474	0.139728	0.10202993	Hexb	1
GO:0042582	azurophil granule	1/23	11/14612	0.09090909	57.7549407	7.47652591	0.01718474	0.139728	0.10202993	Hexb	1
GO:0001674	female germ cell nucleus	1/23	12/14612	0.08333333	52.942029	7.1470074	0.01873291	0.139728	0.10202993	Marcks	1
GO:0036126	sperm flagellum	2/23	135/14612	0.01481481	9.41191626	3.89864476	0.01888216	0.139728	0.10202993	Slc9b2/Marcks	2
GO:0033391	chromatoid body	1/23	13/14612	0.07692308	48.8695652	6.85584091	0.02027874	0.14068375	0.10272782	Marcks	1
GO:0097729	9+2 motile cilium	2/23	152/14612	0.01315789	8.35926773	3.6212906	0.02357368	0.14456815	0.10556423	Slc9b2/Marcks	2
GO:0030136	clathrin-coated vesicle	2/23	155/14612	0.01290323	8.19747546	3.57682817	0.0244469	0.14456815	0.10556423	Rab8a/Rab13	2
GO:0030133	transport vesicle	3/23	411/14612	0.00729927	4.63725801	2.96979644	0.02577254	0.14456815	0.10556423	Slc9b2/Rab8a/Rab13	3
GO:0036064	ciliary basal body	2/23	170/14612	0.01176471	7.4741688	3.371211	0.02900907	0.14456815	0.10556423	Rab8a/Arl3	2
GO:0005686	U2 snRNP	1/23	21/14612	0.04761905	30.252588	5.32626217	0.03256181	0.14456815	0.10556423	Phf5a	1
GO:0097228	sperm principal piece	1/23	21/14612	0.04761905	30.252588	5.32626217	0.03256181	0.14456815	0.10556423	Slc9b2	1
GO:0010008	endosome membrane	3/23	456/14612	0.00657895	4.17963387	2.73892654	0.03364635	0.14456815	0.10556423	Slc9b2/Rab8a/Rab13	3
GO:0030496	midbody	2/23	190/14612	0.01052632	6.68741419	3.13306957	0.03557902	0.14456815	0.10556423	Rab8a/Arl3	2
GO:0099571	postsynaptic cytoskeleton	1/23	25/14612	0.04	25.4121739	4.85047862	0.03864797	0.14456815	0.10556423	Marcks	1
GO:0000323	lytic vacuole	3/23	487/14612	0.00616016	3.91357914	2.59649923	0.03972983	0.14456815	0.10556423	Slc9b2/Rab8a/Hexb	3
GO:0005764	lysosome	3/23	487/14612	0.00616016	3.91357914	2.59649923	0.03972983	0.14456815	0.10556423	Slc9b2/Rab8a/Hexb	3
GO:0030139	endocytic vesicle	2/23	203/14612	0.00985222	6.25915614	2.99597572	0.04013194	0.14456815	0.10556423	Rab8a/Rab13	2
GO:0005689	U12-type spliceosomal complex	1/23	26/14612	0.03846154	24.4347826	4.74865507	0.04016378	0.14456815	0.10556423	Phf5a	1
GO:0031514	motile cilium	2/23	204/14612	0.00980392	6.228474	2.98592787	0.04049102	0.14456815	0.10556423	Slc9b2/Marcks	2
GO:0001891	phagocytic cup	1/23	27/14612	0.03703704	23.5297907	4.65239894	0.0416773	0.14456815	0.10556423	Megf10	1
GO:0043202	lysosomal lumen	1/23	27/14612	0.03703704	23.5297907	4.65239894	0.0416773	0.14456815	0.10556423	Hexb	1
GO:0005775	vacuolar lumen	1/23	31/14612	0.03225806	20.4936886	4.31392255	0.0477086	0.16024509	0.11701159	Hexb	1
GO:0043197	dendritic spine	2/23	227/14612	0.00881057	5.59739514	2.77179402	0.04908408	0.16024509	0.11701159	Rab8a/Marcks	2
GO:0044309	neuron spine	2/23	232/14612	0.00862069	5.47676162	2.72910123	0.05103399	0.16185064	0.11818397	Rab8a/Marcks	2
GO:0030135	coated vesicle	2/23	240/14612	0.00833333	5.2942029	2.66330377	0.054212	0.16715366	0.12205626	Rab8a/Rab13	2
GO:0032391	photoreceptor connecting cilium	1/23	39/14612	0.02564103	16.2898551	3.79622825	0.05966243	0.1789873	0.13069722	Arl3	1
GO:0097546	ciliary base	1/23	42/14612	0.02380952	15.126294	3.64010772	0.06410801	0.18726287	0.13674007	Rab8a	1
GO:0099523	presynaptic cytosol	1/23	49/14612	0.02040816	12.9653949	3.33112382	0.07440297	0.21176231	0.15462966	Marcks	1
GO:0030670	phagocytic vesicle membrane	1/23	52/14612	0.01923077	12.2173913	3.21739202	0.07878186	0.21461962	0.15671608	Rab8a	1
GO:0071005	U2-type precatalytic spliceosome	1/23	53/14612	0.01886792	11.9868745	3.18154046	0.08023708	0.21461962	0.15671608	Phf5a	1
GO:0060170	ciliary membrane	1/23	54/14612	0.01851852	11.7648953	3.14663928	0.08169011	0.21461962	0.15671608	Rab8a	1
GO:0071011	precatalytic spliceosome	1/23	55/14612	0.01818182	11.5509881	3.11264552	0.08314093	0.21461962	0.15671608	Phf5a	1
GO:0030141	secretory granule	2/23	318/14612	0.00628931	3.99562483	2.14444652	0.08854654	0.21958725	0.16034346	Rab13/Hexb	2
GO:0016324	apical plasma membrane	2/23	319/14612	0.00626959	3.98309936	2.1389099	0.08902186	0.21958725	0.16034346	Slc47a1/Slc9b2	2
GO:0016328	lateral plasma membrane	1/23	63/14612	0.01587302	10.084196	2.86900513	0.09466887	0.22357968	0.16325875	Rab13	1
GO:0097525	spliceosomal snRNP complex	1/23	63/14612	0.01587302	10.084196	2.86900513	0.09466887	0.22357968	0.16325875	Phf5a	1
GO:0043073	germ cell nucleus	1/23	66/14612	0.01515152	9.62582345	2.78863624	0.098956	0.22883575	0.16709675	Marcks	1
GO:0030532	small nuclear ribonucleoprotein complex	1/23	71/14612	0.01408451	8.94794856	2.66549708	0.10605809	0.23083232	0.16855466	Phf5a	1
GO:0035869	ciliary transition zone	1/23	71/14612	0.01408451	8.94794856	2.66549708	0.10605809	0.23083232	0.16855466	Arl3	1
GO:0099522	cytosolic region	1/23	71/14612	0.01408451	8.94794856	2.66549708	0.10605809	0.23083232	0.16855466	Marcks	1
GO:0030666	endocytic vesicle membrane	1/23	73/14612	0.01369863	8.70279929	2.61959329	0.10888391	0.23242527	0.16971784	Rab8a	1
GO:0005876	spindle microtubule	1/23	77/14612	0.01298701	8.25070582	2.53284874	0.11450994	0.2398227	0.17511947	Arl3	1
GO:0097733	photoreceptor cell cilium	1/23	79/14612	0.01265823	8.04182719	2.4917955	0.11731019	0.24113762	0.17607962	Arl3	1
GO:0120114	Sm-like protein family complex	1/23	83/14612	0.01204819	7.65426925	2.41386335	0.12288531	0.2480049	0.18109414	Phf5a	1
GO:0045177	apical part of cell	2/23	393/14612	0.00508906	3.23310101	1.78180938	0.12617851	0.25010384	0.1826268	Slc47a1/Slc9b2	2
GO:0097731	9+0 non-motile cilium	1/23	92/14612	0.01086957	6.90548204	2.25609345	0.13530645	0.2604016	0.19014626	Arl3	1
GO:0005881	cytoplasmic microtubule	1/23	94/14612	0.0106383	6.75855689	2.22390033	0.13804376	0.2604016	0.19014626	Arl3	1
GO:0005874	microtubule	2/23	416/14612	0.00480769	3.05434783	1.68783028	0.13841166	0.2604016	0.19014626	Marcks/Arl3	2
GO:0005684	U2-type spliceosomal complex	1/23	96/14612	0.01041667	6.61775362	2.19263303	0.14077278	0.26042964	0.19016674	Phf5a	1
GO:0001669	acrosomal vesicle	1/23	99/14612	0.01010101	6.41721563	2.14736687	0.14485082	0.26358101	0.19246788	Hexb	1
GO:0005923	bicellular tight junction	1/23	104/14612	0.00961538	6.10869565	2.07593644	0.15160645	0.27142445	0.19819519	Rab13	1
GO:0032432	actin filament bundle	1/23	109/14612	0.00917431	5.82848026	2.00902764	0.15831101	0.2752096	0.20095912	Marcks	1
GO:0005667	transcription regulator complex	2/23	458/14612	0.00436681	2.77425479	1.53179362	0.16140811	0.2752096	0.20095912	Npas2/Pbx3	2
GO:0070160	tight junction	1/23	112/14612	0.00892857	5.67236025	1.97084494	0.16230939	0.2752096	0.20095912	Rab13	1
GO:0019897	extrinsic component of plasma membrane	1/23	113/14612	0.00884956	5.62216237	1.95842309	0.16363814	0.2752096	0.20095912	Rab13	1
GO:0005911	cell-cell junction	2/23	467/14612	0.00428266	2.7207895	1.50063765	0.16643178	0.27573027	0.20133931	Fabp7/Rab13	2
GO:0045335	phagocytic vesicle	1/23	117/14612	0.00854701	5.42995169	1.91017843	0.16893301	0.2757583	0.20135978	Rab8a	1
GO:0016363	nuclear matrix	1/23	121/14612	0.00826446	5.25044916	1.86410108	0.17419581	0.28022804	0.2046236	Phf5a	1
GO:0043296	apical junction complex	1/23	127/14612	0.00787402	5.00239644	1.79867973	0.18203027	0.28864801	0.2107719	Rab13	1
GO:0005930	axoneme	1/23	134/14612	0.00746269	4.74107722	1.72737215	0.19108063	0.29656378	0.21655203	Arl3	1
GO:0097014	ciliary plasm	1/23	135/14612	0.00740741	4.70595813	1.71758893	0.1923657	0.29656378	0.21655203	Arl3	1
GO:0005814	centriole	1/23	148/14612	0.00675676	4.29259694	1.59851365	0.20889484	0.31334226	0.22880373	Rab8a	1
GO:0034399	nuclear periphery	1/23	148/14612	0.00675676	4.29259694	1.59851365	0.20889484	0.31334226	0.22880373	Phf5a	1
GO:0030672	synaptic vesicle membrane	1/23	164/14612	0.00609756	3.873807	1.4694905	0.22879444	0.3341603	0.24400515	Slc9b2	1
GO:0099501	exocytic vesicle membrane	1/23	164/14612	0.00609756	3.873807	1.4694905	0.22879444	0.3341603	0.24400515	Slc9b2	1
GO:0019898	extrinsic component of membrane	1/23	174/14612	0.00574713	3.65117441	1.39685244	0.24098758	0.34739768	0.25367113	Rab13	1
GO:0043679	axon terminus	1/23	182/14612	0.00549451	3.49068323	1.34249338	0.2506091	0.35663603	0.26041702	Marcks	1
GO:0005681	spliceosomal complex	1/23	194/14612	0.00515464	3.27476468	1.26641474	0.26482295	0.372093	0.27170376	Phf5a	1
GO:0044306	neuron projection terminus	1/23	204/14612	0.00490196	3.114237	1.20741906	0.27647048	0.38360279	0.28010825	Marcks	1
GO:0030027	lamellipodium	1/23	212/14612	0.00471698	2.99671862	1.1627723	0.28566121	0.39146165	0.28584682	Rab13	1
GO:0090575	RNA polymerase II transcription regulator complex	1/23	227/14612	0.00440529	2.79869757	1.08444435	0.30259368	0.3977311	0.2904248	Npas2	1
GO:0036464	cytoplasmic ribonucleoprotein granule	1/23	229/14612	0.00436681	2.77425479	1.0744844	0.30482213	0.3977311	0.2904248	Marcks	1
GO:0030658	transport vesicle membrane	1/23	231/14612	0.004329	2.75023527	1.06463075	0.30704376	0.3977311	0.2904248	Slc9b2	1
GO:0030426	growth cone	1/23	232/14612	0.00431034	2.73838081	1.05974309	0.30815202	0.3977311	0.2904248	Marcks	1
GO:0032838	plasma membrane bounded cell projection cytoplasm	1/23	232/14612	0.00431034	2.73838081	1.05974309	0.30815202	0.3977311	0.2904248	Arl3	1
GO:0030427	site of polarized growth	1/23	238/14612	0.00420168	2.669346	1.03094891	0.31476611	0.40159814	0.29324852	Marcks	1
GO:0005802	trans-Golgi network	1/23	243/14612	0.00411523	2.61442118	1.00762246	0.3202316	0.4039285	0.29495016	Rab13	1
GO:0035770	ribonucleoprotein granule	1/23	248/14612	0.00403226	2.56171108	0.98487238	0.32565539	0.40430305	0.29522366	Marcks	1
GO:0042734	presynaptic membrane	1/23	250/14612	0.004	2.54121739	0.97592753	0.32781329	0.40430305	0.29522366	Marcks	1
GO:0099568	cytoplasmic region	1/23	270/14612	0.0037037	2.35297907	0.89096209	0.34903214	0.4257425	0.31087883	Arl3	1
GO:0005765	lysosomal membrane	1/23	280/14612	0.00357143	2.2689441	0.85125413	0.35939981	0.42896106	0.31322903	Slc9b2	1
GO:0098852	lytic vacuole membrane	1/23	280/14612	0.00357143	2.2689441	0.85125413	0.35939981	0.42896106	0.31322903	Slc9b2	1
GO:0009897	external side of plasma membrane	1/23	298/14612	0.0033557	2.13189378	0.78383459	0.37766486	0.44596595	0.32564607	Mill2	1
GO:0062023	collagen-containing extracellular matrix	1/23	306/14612	0.00326797	2.076158	0.75538571	0.38562179	0.45056861	0.32900695	Angptl4	1
GO:0031253	cell projection membrane	1/23	324/14612	0.00308642	1.96081589	0.69441339	0.40317061	0.46574766	0.34009075	Rab8a	1
GO:0005938	cell cortex	1/23	328/14612	0.00304878	1.9369035	0.6813935	0.40700471	0.46574766	0.34009075	Marcks	1
GO:0005774	vacuolar membrane	1/23	334/14612	0.00299401	1.90210883	0.66220065	0.41271172	0.46745919	0.34134052	Slc9b2	1
GO:0098791	Golgi apparatus subcompartment	1/23	347/14612	0.00288184	1.83084826	0.62193027	0.4248972	0.47574341	0.34738969	Rab13	1
GO:0005759	mitochondrial matrix	1/23	351/14612	0.002849	1.8099839	0.60988237	0.42859767	0.47574341	0.34738969	Hmgcs2	1
GO:0045211	postsynaptic membrane	1/23	365/14612	0.00273973	1.74055986	0.5689001	0.44137078	0.48507086	0.35420063	Marcks	1
GO:0016607	nuclear speck	1/23	389/14612	0.00257069	1.63317313	0.50256565	0.46263461	0.49259616	0.35969563	Phf5a	1
GO:0150034	distal axon	1/23	393/14612	0.00254453	1.6165505	0.49195064	0.46610244	0.49259616	0.35969563	Marcks	1
GO:0005819	spindle	1/23	394/14612	0.00253807	1.61244758	0.48931543	0.46696605	0.49259616	0.35969563	Arl3	1
GO:0030312	external encapsulating structure	1/23	398/14612	0.00251256	1.59624208	0.4788474	0.47040714	0.49259616	0.35969563	Angptl4	1
GO:0031012	extracellular matrix	1/23	398/14612	0.00251256	1.59624208	0.4788474	0.47040714	0.49259616	0.35969563	Angptl4	1
GO:0014069	postsynaptic density	1/23	443/14612	0.00225734	1.43409559	0.36839079	0.50768395	0.52666279	0.38457121	Rab8a	1
GO:0031252	cell leading edge	1/23	454/14612	0.00220264	1.39934878	0.34321832	0.51640674	0.53075137	0.38755671	Rab13	1
GO:0032279	asymmetric synapse	1/23	465/14612	0.00215054	1.36624591	0.31868249	0.52498158	0.53461427	0.39037742	Rab8a	1
GO:0099572	postsynaptic specialization	1/23	487/14612	0.00205339	1.30452638	0.27138529	0.54169699	0.54662151	0.39914515	Rab8a	1
GO:0015629	actin cytoskeleton	1/23	495/14612	0.0020202	1.28344313	0.2547349	0.54763465	0.54763465	0.39988495	Marcks	1

ID	Description	GeneRatio	BgRatio	RichFactor	FoldEnrichm	zScore	pvalue	p.adjust	qvalue	geneID	Count
GO:0062023	collagen-containing extracellular matrix	7/30	306/14612	0.02287582	11.1420479	8.13241087	2.2199E-06	0.00014652	0.00012385	Fmod/Fbn1/Cilp/Ccn3/Loxl1/Comp/Col6a1	7
GO:0030312	external encapsulating structure	7/30	398/14612	0.01758794	8.56649916	6.94177319	1.2492E-05	0.00027483	0.00023231	Fmod/Fbn1/Cilp/Ccn3/Loxl1/Comp/Col6a1	7
GO:0031012	extracellular matrix	7/30	398/14612	0.01758794	8.56649916	6.94177319	1.2492E-05	0.00027483	0.00023231	Fmod/Fbn1/Cilp/Ccn3/Loxl1/Comp/Col6a1	7
GO:0005604	basement membrane	3/30	98/14612	0.03061224	14.9102041	6.2667884	0.00104144	0.01718368	0.01452528	Fbn1/Loxl1/Col6a1	3
GO:0000786	nucleosome	2/30	55/14612	0.03636364	17.7115152	5.63187856	0.00565604	0.07465976	0.06310953	H3f3b/H1f0	2
GO:0016529	sarcoplasmic reticulum	2/30	80/14612	0.025	12.1766667	4.54659896	0.01165984	0.12825824	0.10841606	Gstm7/Col6a1	2
GO:0016528	sarcoplasm	2/30	108/14612	0.01851852	9.01975309	3.79420708	0.02057765	0.17569698	0.14851579	Gstm7/Col6a1	2
GO:0000781	chromosome, telomeric region	2/30	110/14612	0.01818182	8.85575758	3.75113346	0.0212966	0.17569698	0.14851579	H3f3b/Telo2	2
GO:0001527	microfibril	1/30	12/14612	0.08333333	40.5888889	6.22270996	0.02437004	0.17871366	0.15106577	Fbn1	1
GO:0032993	protein-DNA complex	2/30	154/14612	0.01298701	6.32554113	3.0134365	0.03959424	0.24218844	0.20472069	H3f3b/H1f0	2
GO:0005921	gap junction	1/30	21/14612	0.04761905	23.1936508	4.61623509	0.04226979	0.24218844	0.20472069	Ccn3	1
GO:0097449	astrocyte projection	1/30	23/14612	0.04347826	21.1768116	4.39233793	0.04620419	0.24218844	0.20472069	Kcnk2	1
GO:0033268	node of Ranvier	1/30	24/14612	0.04166667	20.2944444	4.29073875	0.04816552	0.24218844	0.20472069	Kcnk2	1
GO:0042383	sarcolemma	2/30	178/14612	0.01123596	5.47265918	2.72316845	0.0513733	0.24218844	0.20472069	Col6a1/Kcnk2	2
GO:0032809	neuronal cell body membrane	1/30	35/14612	0.02857143	13.9161905	3.46997573	0.06948448	0.28277988	0.23903244	Cx3cr1	1
GO:0044305	calyx of Held	1/30	36/14612	0.02777778	13.5296296	3.41399087	0.07139951	0.28277988	0.23903244	Kcnk2	1
GO:0044298	cell body membrane	1/30	38/14612	0.02631579	12.8175439	3.30842801	0.07521816	0.28277988	0.23903244	Cx3cr1	1
GO:0005871	kinesin complex	1/30	39/14612	0.02564103	12.4888889	3.25857635	0.07712179	0.28277988	0.23903244	Kifc3	1
GO:0097386	glial cell projection	1/30	43/14612	0.02325581	11.3271318	3.07603396	0.08469848	0.29421579	0.24869915	Kcnk2	1
GO:0000791	euchromatin	1/30	61/14612	0.01639344	7.98469945	2.47946665	0.11805594	0.34567708	0.29219913	H1f0	1
GO:0022625	cytosolic large ribosomal subunit	1/30	62/14612	0.01612903	7.85591398	2.45370167	0.11987425	0.34567708	0.29219913	Rpl8	1
GO:0009897	external side of plasma membrane	2/30	298/14612	0.00671141	3.2689038	1.79488141	0.12431799	0.34567708	0.29219913	Asgr1/Cx3cr1	2
GO:0005581	collagen trimer	1/30	68/14612	0.01470588	7.1627451	2.31035533	0.13070829	0.34567708	0.29219913	Col6a1	1
GO:0008076	voltage-gated potassium channel complex	1/30	71/14612	0.01408451	6.8600939	2.24506367	0.13607685	0.34567708	0.29219913	Kcnk2	1
GO:0016324	apical plasma membrane	2/30	319/14612	0.00626959	3.05370951	1.68214996	0.13885049	0.34567708	0.29219913	Kcnk2/Kl	2
GO:0098687	chromosomal region	2/30	321/14612	0.00623053	3.03468328	1.67189914	0.14025444	0.34567708	0.29219913	H3f3b/Telo2	2
GO:0017053	transcription repressor complex	1/30	74/14612	0.01351351	6.58198198	2.18345381	0.14141335	0.34567708	0.29219913	H1f0	1
GO:0045171	intercellular bridge	1/30	82/14612	0.01219512	5.9398374	2.03459899	0.15548871	0.35356035	0.29886281	Gstm7	1
GO:0034705	potassium channel complex	1/30	85/14612	0.01176471	5.73019608	1.98377626	0.16070925	0.35356035	0.29886281	Kcnk2	1
GO:0044304	main axon	1/30	85/14612	0.01176471	5.73019608	1.98377626	0.16070925	0.35356035	0.29886281	Kcnk2	1
GO:0055038	recycling endosome membrane	1/30	92/14612	0.01086957	5.2942029	1.87406745	0.17276947	0.36783177	0.31092638	Fzd7	1
GO:0001669	acrosomal vesicle	1/30	99/14612	0.01010101	4.91986532	1.77501569	0.18466209	0.38086555	0.32194377	Loxl1	1
GO:0045177	apical part of cell	2/30	393/14612	0.00508906	2.47871077	1.34783595	0.19261507	0.38523014	0.32563313	Kcnk2/Kl	2
GO:0015934	large ribosomal subunit	1/30	120/14612	0.00833333	4.05888889	1.52609726	0.21935667	0.3874078	0.3274739	Rpl8	1
GO:0005875	microtubule associated complex	1/30	121/14612	0.00826446	4.02534435	1.51568995	0.22097269	0.3874078	0.3274739	Kifc3	1
GO:0022626	cytosolic ribosome	1/30	123/14612	0.00813008	3.9598916	1.4952066	0.22419502	0.3874078	0.3274739	Rpl8	1
GO:0014069	postsynaptic density	2/30	443/14612	0.00451467	2.19894658	1.16231581	0.23041301	0.3874078	0.3274739	Kcnk2/Rpl8	2
GO:0043296	apical junction complex	1/30	127/14612	0.00787402	3.8351706	1.45550553	0.23060109	0.3874078	0.3274739	Kifc3	1
GO:0032279	asymmetric synapse	2/30	465/14612	0.00430108	2.09491039	1.08834085	0.24724536	0.3874078	0.3274739	Kcnk2/Rpl8	2
GO:0005911	cell-cell junction	2/30	467/14612	0.00428266	2.08593862	1.08181822	0.24877932	0.3874078	0.3274739	Ccn3/Kifc3	2
GO:0098685	Schaffer collateral - CA1 synapse	1/30	144/14612	0.00694444	3.38240741	1.303122	0.25726098	0.3874078	0.3274739	Kcnk2	1
GO:0098839	postsynaptic density membrane	1/30	147/14612	0.00680272	3.31337868	1.27861039	0.26187203	0.3874078	0.3274739	Kcnk2	1
GO:0000323	lytic vacuole	2/30	487/14612	0.00410678	2.00027379	1.01831277	0.26414168	0.3874078	0.3274739	Col6a1/Cpq	2
GO:0005764	lysosome	2/30	487/14612	0.00410678	2.00027379	1.01831277	0.26414168	0.3874078	0.3274739	Col6a1/Cpq	2
GO:0099572	postsynaptic specialization	2/30	487/14612	0.00410678	2.00027379	1.01831277	0.26414168	0.3874078	0.3274739	Kcnk2/Rpl8	2
GO:0005912	adherens junction	1/30	161/14612	0.00621118	3.0252588	1.17202416	0.28302686	0.40608201	0.34325912	Kifc3	1
GO:0034703	cation channel complex	1/30	176/14612	0.00568182	2.76742424	1.0699556	0.30504217	0.42248629	0.35712557	Kcnk2	1
GO:0099634	postsynaptic specialization membrane	1/30	180/14612	0.00555556	2.70592593	1.04454033	0.31080165	0.42248629	0.35712557	Kcnk2	1
GO:0043679	axon terminus	1/30	182/14612	0.00549451	2.67619048	1.0320909	0.31366407	0.42248629	0.35712557	Kcnk2	1
GO:0055037	recycling endosome	1/30	187/14612	0.00534759	2.60463458	1.00168482	0.32076991	0.42341628	0.35791169	Fzd7	1
GO:0044391	ribosomal subunit	1/30	193/14612	0.00518135	2.52366149	0.96647722	0.32920308	0.42602752	0.36011896	Rpl8	1
GO:0044306	neuron projection terminus	1/30	204/14612	0.00490196	2.3875817	0.90524023	0.34440176	0.43712531	0.36949986	Kcnk2	1
GO:0005840	ribosome	1/30	240/14612	0.00416667	2.02944444	0.72936007	0.39185078	0.48048028	0.4061476	Rpl8	1
GO:0030016	myofibril	1/30	241/14612	0.00414938	2.02102351	0.72492457	0.39312023	0.48048028	0.4061476	Col6a1	1
GO:0043292	contractile muscle fiber	1/30	249/14612	0.00401606	1.95609103	0.69018956	0.40318399	0.48382079	0.40897132	Col6a1	1
GO:0034702	monoatomic ion channel complex	1/30	271/14612	0.00369004	1.79729397	0.6009045	0.43003457	0.50682645	0.42841789	Kcnk2	1
GO:0030141	secretory granule	1/30	318/14612	0.00314465	1.53165618	0.43477002	0.48354153	0.55989019	0.47327241	Loxl1	1
GO:0045211	postsynaptic membrane	1/30	365/14612	0.00273973	1.33442922	0.29348168	0.53217747	0.60558125	0.51189484	Kcnk2	1
GO:1902495	transmembrane transporter complex	1/30	388/14612	0.00257732	1.25532646	0.23120268	0.55433426	0.61491642	0.51978581	Kcnk2	1
GO:0150034	distal axon	1/30	393/14612	0.00254453	1.23935539	0.21817099	0.55901493	0.61491642	0.51978581	Kcnk2	1
GO:1990351	transporter complex	1/30	412/14612	0.00242718	1.18220065	0.17015566	0.57637187	0.61735641	0.52184832	Kcnk2	1
GO:0005874	microtubule	1/30	416/14612	0.00240385	1.17083333	0.160335	0.57994087	0.61735641	0.52184832	Kifc3	1
GO:0010008	endosome membrane	1/30	456/14612	0.00219298	1.06812865	0.06704	0.614069	0.62929385	0.53193898	Fzd7	1
GO:0005667	transcription regulator complex	1/30	458/14612	0.00218341	1.06346434	0.06259144	0.61570308	0.62929385	0.53193898	H1f0	1
GO:0098797	plasma membrane protein complex	1/30	463/14612	0.00215983	1.05197984	0.05155308	0.61975909	0.62929385	0.53193898	Kcnk2	1
GO:0015629	actin cytoskeleton	1/30	495/14612	0.0020202	0.98397306	-0.0164541	0.64475451	0.64475451	0.5450078	H1f0	1

ID	Description	GeneRatio	BgRatio	RichFactor	FoldEnrichm	zScore	pvalue	p.adjust	qvalue	geneID	Count
GO:0009925	basal plasma membrane	5/69	258/14612	0.01937984	4.10403325	3.46487252	0.00740463	0.40527483	0.38692089	Slco1a4/Ajap1/Ager/Slc30a1/Enpp1	5
GO:0045178	basal part of cell	5/69	275/14612	0.01818182	3.85032938	3.28677343	0.00960597	0.40527483	0.38692089	Slco1a4/Ajap1/Ager/Slc30a1/Enpp1	5
GO:0016607	nuclear speck	6/69	389/14612	0.01542416	3.26634626	3.12063499	0.01002119	0.40527483	0.38692089	Srsf5/Arglu1/Nfatc4/Luc7l2/Ehmt2/Dazap2	6
GO:0014069	postsynaptic density	6/69	443/14612	0.01354402	2.86819119	2.75036983	0.01803228	0.40527483	0.38692089	Hnrnpd/Cd200/Slc30a1/Tiam1/Abi3bp/Il1rapl1	6
GO:0032279	asymmetric synapse	6/69	465/14612	0.01290323	2.73249182	2.61518855	0.02229592	0.40527483	0.38692089	Hnrnpd/Cd200/Slc30a1/Tiam1/Abi3bp/Il1rapl1	6
GO:0016323	basolateral plasma membrane	4/69	235/14612	0.01702128	3.60456368	2.77250468	0.02498972	0.40527483	0.38692089	Slco1a4/Ajap1/Slc30a1/Enpp1	4
GO:0001673	male germ cell nucleus	2/69	54/14612	0.03703704	7.84326355	3.47013845	0.02685684	0.40527483	0.38692089	Spata24/Cdk2	2
GO:0099572	postsynaptic specialization	6/69	487/14612	0.01232033	2.60905276	2.4875857	0.02719833	0.40527483	0.38692089	Hnrnpd/Cd200/Slc30a1/Tiam1/Abi3bp/Il1rapl1	6
GO:0098839	postsynaptic density membrane	3/69	147/14612	0.02040816	4.32179828	2.78811202	0.03227004	0.40527483	0.38692089	Slc30a1/Tiam1/Il1rapl1	3
GO:0043073	germ cell nucleus	2/69	66/14612	0.03030303	6.41721563	3.03818219	0.03883279	0.40527483	0.38692089	Spata24/Cdk2	2
GO:0005922	connexin complex	1/69	10/14612	0.1	21.1768116	4.39626517	0.0462445	0.40527483	0.38692089	Gja4	1
GO:0019774	proteasome core complex, beta-subunit complex	1/69	10/14612	0.1	21.1768116	4.39626517	0.0462445	0.40527483	0.38692089	Psmb9	1
GO:0061702	canonical inflammasome complex	1/69	10/14612	0.1	21.1768116	4.39626517	0.0462445	0.40527483	0.38692089	Aim2	1
GO:0097441	basal dendrite	1/69	10/14612	0.1	21.1768116	4.39626517	0.0462445	0.40527483	0.38692089	Enpp1	1
GO:0098553	lumenal side of endoplasmic reticulum membrane	1/69	10/14612	0.1	21.1768116	4.39626517	0.0462445	0.40527483	0.38692089	H2-T23	1
GO:0099147	extrinsic component of postsynaptic density membrane	1/69	10/14612	0.1	21.1768116	4.39626517	0.0462445	0.40527483	0.38692089	Tiam1	1
GO:0032398	MHC class Ib protein complex	1/69	11/14612	0.09090909	19.2516469	4.17104249	0.05075135	0.40527483	0.38692089	H2-T23	1
GO:0099634	postsynaptic specialization membrane	3/69	180/14612	0.01666667	3.5294686	2.35201344	0.0533517	0.40527483	0.38692089	Slc30a1/Tiam1/Il1rapl1	3
GO:0042612	MHC class I protein complex	1/69	12/14612	0.08333333	17.647343	3.97371427	0.05523722	0.40527483	0.38692089	H2-T23	1
GO:0098892	extrinsic component of postsynaptic specialization membrane	1/69	12/14612	0.08333333	17.647343	3.97371427	0.05523722	0.40527483	0.38692089	Tiam1	1
GO:1902711	GABA-A receptor complex	1/69	12/14612	0.08333333	17.647343	3.97371427	0.05523722	0.40527483	0.38692089	Gabrg1	1
GO:0044291	cell-cell contact zone	2/69	82/14612	0.02439024	5.165076	2.60516097	0.05731743	0.40527483	0.38692089	Ajap1/Tiam1	2
GO:0005662	DNA replication factor A complex	1/69	13/14612	0.07692308	16.2898551	3.79884002	0.0597022	0.40527483	0.38692089	Rpa2	1
GO:0030897	HOPS complex	1/69	13/14612	0.07692308	16.2898551	3.79884002	0.0597022	0.40527483	0.38692089	Stx17	1
GO:1902710	GABA receptor complex	1/69	14/14612	0.07142857	15.126294	3.64236188	0.06414637	0.40527483	0.38692089	Gabrg1	1
GO:0005614	interstitial matrix	1/69	15/14612	0.06666667	14.1178744	3.50118297	0.06856985	0.40527483	0.38692089	Abi3bp	1
GO:0035861	site of double-strand break	2/69	93/14612	0.02150538	4.55415303	2.36835477	0.07145968	0.40527483	0.38692089	Aim2/Rpa2	2
GO:0005839	proteasome core complex	1/69	17/14612	0.05882353	12.456948	3.25558371	0.07735508	0.40527483	0.38692089	Psmb9	1
GO:0071004	U2-type prespliceosome	1/69	17/14612	0.05882353	12.456948	3.25558371	0.07735508	0.40527483	0.38692089	Luc7l2	1
GO:0071010	prespliceosome	1/69	17/14612	0.05882353	12.456948	3.25558371	0.07735508	0.40527483	0.38692089	Luc7l2	1
GO:0098576	lumenal side of membrane	1/69	17/14612	0.05882353	12.456948	3.25558371	0.07735508	0.40527483	0.38692089	H2-T23	1
GO:0000159	protein phosphatase type 2A complex	1/69	18/14612	0.05555556	11.7648953	3.14772227	0.08171701	0.40527483	0.38692089	Ppp2r3a	1
GO:0005685	U1 snRNP	1/69	18/14612	0.05555556	11.7648953	3.14772227	0.08171701	0.40527483	0.38692089	Luc7l2	1
GO:0042611	MHC protein complex	1/69	20/14612	0.05	10.5884058	2.95557175	0.09038001	0.40527483	0.38692089	H2-T23	1
GO:0043197	dendritic spine	3/69	227/14612	0.01321586	2.79869757	1.88128094	0.09211894	0.40527483	0.38692089	Nr1d1/Tiam1/Abi3bp	3
GO:0045211	postsynaptic membrane	4/69	365/14612	0.0109589	2.32074648	1.76011764	0.09380662	0.40527483	0.38692089	Slc30a1/Gabrg1/Tiam1/Il1rapl1	4
GO:0005921	gap junction	1/69	21/14612	0.04761905	10.084196	2.86940023	0.09468126	0.40527483	0.38692089	Gja4	1
GO:0043601	nuclear replisome	1/69	21/14612	0.04761905	10.084196	2.86940023	0.09468126	0.40527483	0.38692089	Rpa2	1
GO:0044233	mitochondria-associated endoplasmic reticulum membrane contact site	1/69	21/14612	0.04761905	10.084196	2.86940023	0.09468126	0.40527483	0.38692089	Stx17	1
GO:0000781	chromosome, telomeric region	2/69	110/14612	0.01818182	3.85032938	2.06687854	0.09523184	0.40527483	0.38692089	Cdk2/Rpa2	2
GO:0044309	neuron spine	3/69	232/14612	0.01293103	2.73838081	1.83842951	0.09678774	0.40527483	0.38692089	Nr1d1/Tiam1/Abi3bp	3
GO:0030894	replisome	1/69	22/14612	0.04545455	9.62582345	2.78882828	0.09896246	0.40527483	0.38692089	Rpa2	1
GO:0005732	sno(s)RNA-containing ribonucleoprotein complex	1/69	23/14612	0.04347826	9.20730939	2.7132479	0.10322371	0.41075546	0.39215331	Rrp9	1
GO:0000242	pericentriolar material	1/69	24/14612	0.04166667	8.8236715	2.64214023	0.10746509	0.41075546	0.39215331	Tiam1	1
GO:0098890	extrinsic component of postsynaptic membrane	1/69	24/14612	0.04166667	8.8236715	2.64214023	0.10746509	0.41075546	0.39215331	Tiam1	1
GO:0090734	site of DNA damage	2/69	122/14612	0.01639344	3.47160846	1.88826485	0.11318405	0.42320992	0.40404374	Aim2/Rpa2	2
GO:0043202	lysosomal lumen	1/69	27/14612	0.03703704	7.84326355	2.45148616	0.12007098	0.43940871	0.41950893	Man2b1	1
GO:0012507	ER to Golgi transport vesicle membrane	1/69	29/14612	0.03448276	7.30234883	2.33999814	0.12837727	0.46001853	0.43918538	Stx17	1
GO:0097440	apical dendrite	1/69	30/14612	0.03333333	7.0589372	2.28815819	0.13250138	0.46510689	0.44404329	Enpp1	1
GO:0005775	vacuolar lumen	1/69	31/14612	0.03225806	6.83122955	2.23864301	0.13660626	0.46992555	0.44864373	Man2b1	1
GO:0016235	aggresome	1/69	33/14612	0.03030303	6.41721563	2.14588646	0.14475869	0.48820577	0.46609608	Xrn2	1
GO:0005790	smooth endoplasmic reticulum	1/69	34/14612	0.02941176	6.228474	2.10233999	0.1488064	0.49220579	0.46991496	Stx17	1
GO:0034707	chloride channel complex	1/69	35/14612	0.02857143	6.0505176	2.06050346	0.15283524	0.4959936	0.47353122	Gabrg1	1
GO:0000803	sex chromosome	1/69	36/14612	0.02777778	5.88244767	2.02025961	0.15684528	0.49958126	0.4769564	Cdk2	1
GO:0043596	nuclear replication fork	1/69	38/14612	0.02631579	5.57284516	1.94413503	0.16480932	0.50620007	0.48327546	Rpa2	1
GO:0099243	extrinsic component of synaptic membrane	1/69	38/14612	0.02631579	5.57284516	1.94413503	0.16480932	0.50620007	0.48327546	Tiam1	1
GO:0005667	transcription regulator complex	4/69	458/14612	0.00873362	1.8495032	1.27231801	0.17034622	0.51402719	0.49074811	Cdk2/Nfatc4/Nfe2l3/Dazap2	4
GO:0005911	cell-cell junction	4/69	467/14612	0.00856531	1.81385967	1.23124365	0.17870715	0.52624905	0.50241647	Ccdc88c/Ajap1/Gja4/Tiam1	4
GO:0033116	endoplasmic reticulum-Golgi intermediate compartment membrane	1/69	42/14612	0.02380952	5.042098	1.80692123	0.18051566	0.52624905	0.50241647	Stx17	1
GO:0031201	SNARE complex	1/69	45/14612	0.02222222	4.70595813	1.71498063	0.19210404	0.53041384	0.50639265	Stx17	1
GO:0031901	early endosome membrane	2/69	174/14612	0.01149425	2.43411628	1.31081977	0.19845484	0.53041384	0.50639265	Epha8/Ldlrad4	2
GO:0044232	organelle membrane contact site	1/69	47/14612	0.0212766	4.50570459	1.65808395	0.19973975	0.53041384	0.50639265	Stx17	1
GO:0044295	axonal growth cone	1/69	47/14612	0.0212766	4.50570459	1.65808395	0.19973975	0.53041384	0.50639265	Tiam1	1
GO:0008287	protein serine/threonine phosphatase complex	1/69	48/14612	0.02083333	4.41183575	1.63081956	0.20353089	0.53041384	0.50639265	Ppp2r3a	1
GO:0099092	postsynaptic density, intracellular component	1/69	48/14612	0.02083333	4.41183575	1.63081956	0.20353089	0.53041384	0.50639265	Slc30a1	1
GO:1903293	phosphatase complex	1/69	48/14612	0.02083333	4.41183575	1.63081956	0.20353089	0.53041384	0.50639265	Ppp2r3a	1
GO:0000307	cyclin-dependent protein kinase holoenzyme complex	1/69	51/14612	0.01960784	4.152316	1.55330502	0.21479843	0.53543957	0.51119078	Cdk2	1
GO:0000421	autophagosome membrane	1/69	51/14612	0.01960784	4.152316	1.55330502	0.21479843	0.53543957	0.51119078	Stx17	1
GO:0032590	dendrite membrane	1/69	51/14612	0.01960784	4.152316	1.55330502	0.21479843	0.53543957	0.51119078	Gabrg1	1
GO:0030670	phagocytic vesicle membrane	1/69	52/14612	0.01923077	4.07246377	1.52878097	0.21851926	0.53693303	0.5126166	H2-T23	1
GO:0015030	Cajal body	1/69	54/14612	0.01851852	3.92163178	1.48152511	0.22590885	0.54727215	0.52248748	Cdk2	1
GO:0031256	leading edge membrane	2/69	192/14612	0.01041667	2.20591787	1.15857254	0.22962621	0.5485515	0.52370889	Gabrg1/Tiam1	2
GO:0099091	postsynaptic specialization, intracellular component	1/69	56/14612	0.01785714	3.7815735	1.43648483	0.23322957	0.54952721	0.52464042	Slc30a1	1
GO:0030134	COPII-coated ER to Golgi transport vesicle	1/69	57/14612	0.01754386	3.7152301	1.41473623	0.23686431	0.55054947	0.52561638	Stx17	1
GO:0032421	stereocilium bundle	1/69	59/14612	0.01694915	3.5892901	1.37267576	0.2440829	0.55976346	0.53441309	Tiam1	1
GO:0097525	spliceosomal snRNP complex	1/69	63/14612	0.01587302	3.36139867	1.29378029	0.25831882	0.57915626	0.55292764	Luc7l2	1
GO:0000502	proteasome complex	1/69	64/14612	0.015625	3.30887681	1.27504824	0.26183631	0.57915626	0.55292764	Psmb9	1
GO:0005581	collagen trimer	1/69	68/14612	0.01470588	3.114237	1.20365966	0.27574262	0.57915626	0.55292764	C1qtnf7	1
GO:0005657	replication fork	1/69	70/14612	0.01428571	3.0252588	1.16991684	0.28259863	0.57915626	0.55292764	Rpa2	1
GO:0099023	vesicle tethering complex	1/69	70/14612	0.01428571	3.0252588	1.16991684	0.28259863	0.57915626	0.55292764	Stx17	1
GO:0030532	small nuclear ribonucleoprotein complex	1/69	71/14612	0.01408451	2.98264952	1.15349443	0.28600261	0.57915626	0.55292764	Luc7l2	1
GO:0043198	dendritic shaft	1/69	71/14612	0.01408451	2.98264952	1.15349443	0.28600261	0.57915626	0.55292764	Abi3bp	1
GO:0005769	early endosome	3/69	399/14612	0.0075188	1.59224147	0.82618994	0.29145163	0.57915626	0.55292764	Rftn1/Epha8/Ldlrad4	3
GO:0016234	inclusion body	1/69	73/14612	0.01369863	2.9009331	1.12149801	0.29276289	0.57915626	0.55292764	Xrn2	1
GO:0030666	endocytic vesicle membrane	1/69	73/14612	0.01369863	2.9009331	1.12149801	0.29276289	0.57915626	0.55292764	H2-T23	1
GO:0032589	neuron projection membrane	1/69	74/14612	0.01351351	2.8617313	1.10590556	0.29611934	0.57915626	0.55292764	Gabrg1	1
GO:0030315	T-tubule	1/69	75/14612	0.01333333	2.82357488	1.09057202	0.29946009	0.57915626	0.55292764	Slc30a1	1
GO:0032040	small-subunit processome	1/69	75/14612	0.01333333	2.82357488	1.09057202	0.29946009	0.57915626	0.55292764	Rrp9	1
GO:0030426	growth cone	2/69	232/14612	0.00862069	1.82558721	0.87310207	0.29967969	0.57915626	0.55292764	Ngef/Tiam1	2
GO:0030427	site of polarized growth	2/69	238/14612	0.00840336	1.779564	0.83519704	0.3101601	0.59275041	0.56590614	Ngef/Tiam1	2
GO:0045171	intercellular bridge	1/69	82/14612	0.01219512	2.582538	0.98984178	0.32241176	0.60113065	0.57390686	Pcif1	1
GO:1903561	extracellular vesicle	1/69	82/14612	0.01219512	2.582538	0.98984178	0.32241176	0.60113065	0.57390686	Hexd	1
GO:0120114	Sm-like protein family complex	1/69	83/14612	0.01204819	2.55142308	0.97631272	0.32562949	0.60113065	0.57390686	Luc7l2	1
GO:0010494	cytoplasmic stress granule	1/69	85/14612	0.01176471	2.4913896	0.94983926	0.33201984	0.60113065	0.57390686	Dazap2	1
GO:0044304	main axon	1/69	85/14612	0.01176471	2.4913896	0.94983926	0.33201984	0.60113065	0.57390686	Tiam1	1
GO:0043230	extracellular organelle	1/69	90/14612	0.01111111	2.35297907	0.88682235	0.34773578	0.61660364	0.58867912	Hexd	1
GO:0065010	extracellular membrane-bounded organelle	1/69	90/14612	0.01111111	2.35297907	0.88682235	0.34773578	0.61660364	0.58867912	Hexd	1
GO:1905369	endopeptidase complex	1/69	92/14612	0.01086957	2.30182735	0.86278286	0.35391961	0.62116504	0.59303394	Psmb9	1
GO:0031252	cell leading edge	3/69	454/14612	0.00660793	1.39934878	0.595411	0.36286405	0.62971689	0.6011985	Gabrg1/Tiam1/Abi3bp	3
GO:0005684	U2-type spliceosomal complex	1/69	96/14612	0.01041667	2.20591787	0.81652105	0.36611447	0.62971689	0.6011985	Luc7l2	1
GO:0005793	endoplasmic reticulum-Golgi intermediate compartment	1/69	99/14612	0.01010101	2.13907188	0.78329913	0.37511148	0.63254092	0.60389464	Stx17	1
GO:0032587	ruffle membrane	1/69	99/14612	0.01010101	2.13907188	0.78329913	0.37511148	0.63254092	0.60389464	Tiam1	1
GO:0005788	endoplasmic reticulum lumen	1/69	105/14612	0.00952381	2.0168392	0.72027052	0.3927296	0.65276918	0.6232068	Foxred2	1
GO:0016605	PML body	1/69	107/14612	0.00934579	1.97914127	0.70018998	0.39849281	0.65276918	0.6232068	Rpa2	1
GO:0030684	preribosome	1/69	107/14612	0.00934579	1.97914127	0.70018998	0.39849281	0.65276918	0.6232068	Rrp9	1
GO:0005776	autophagosome	1/69	111/14612	0.00900901	1.90782086	0.66130253	0.409858	0.65558979	0.62589967	Stx17	1
GO:1902554	serine/threonine protein kinase complex	1/69	111/14612	0.00900901	1.90782086	0.66130253	0.409858	0.65558979	0.62589967	Cdk2	1
GO:0009897	external side of plasma membrane	2/69	298/14612	0.00671141	1.42126252	0.50607859	0.41241189	0.65558979	0.62589967	Slamf9/H2-T23	2
GO:0019897	extrinsic component of plasma membrane	1/69	113/14612	0.00884956	1.87405412	0.64245992	0.41546097	0.65558979	0.62589967	Tiam1	1
GO:0045335	phagocytic vesicle	1/69	117/14612	0.00854701	1.8099839	0.60589549	0.42651009	0.65696365	0.62721132	H2-T23	1
GO:0097730	non-motile cilium	1/69	117/14612	0.00854701	1.8099839	0.60589549	0.42651009	0.65696365	0.62721132	Tiam1	1
GO:1905368	peptidase complex	1/69	122/14612	0.00819672	1.73580423	0.56214133	0.44003241	0.65696365	0.62721132	Psmb9	1
GO:0022626	cytosolic ribosome	1/69	123/14612	0.00813008	1.721692	0.553634	0.44269893	0.65696365	0.62721132	Zfp598	1
GO:0030662	coated vesicle membrane	1/69	123/14612	0.00813008	1.721692	0.553634	0.44269893	0.65696365	0.62721132	Stx17	1
GO:1902911	protein kinase complex	1/69	123/14612	0.00813008	1.721692	0.553634	0.44269893	0.65696365	0.62721132	Cdk2	1
GO:0016324	apical plasma membrane	2/69	319/14612	0.00626959	1.32769979	0.40761233	0.44655533	0.65696365	0.62721132	Ajap1/Ager	2
GO:0098687	chromosomal region	2/69	321/14612	0.00623053	1.31942751	0.39859426	0.44975164	0.65696365	0.62721132	Cdk2/Rpa2	2
GO:0043296	apical junction complex	1/69	127/14612	0.00787402	1.66746548	0.52036603	0.45324041	0.65696365	0.62721132	Ccdc88c	1
GO:0031253	cell projection membrane	2/69	324/14612	0.00617284	1.30721059	0.38517712	0.45452718	0.65696365	0.62721132	Gabrg1/Tiam1	2
GO:0005774	vacuolar membrane	2/69	334/14612	0.00598802	1.26807255	0.34137319	0.47027784	0.67406491	0.6435381	Fnip1/Stx17	2
GO:0048786	presynaptic active zone	1/69	136/14612	0.00735294	1.5571185	0.4496041	0.47624557	0.67697717	0.64631848	Cd200	1
GO:0001650	fibrillar center	1/69	144/14612	0.00694444	1.47061192	0.39091058	0.49589206	0.69344255	0.66203818	Ager	1
GO:0098685	Schaffer collateral - CA1 synapse	1/69	144/14612	0.00694444	1.47061192	0.39091058	0.49589206	0.69344255	0.66203818	Slc30a1	1
GO:0009898	cytoplasmic side of plasma membrane	1/69	147/14612	0.00680272	1.44059943	0.36981191	0.5030707	0.69780775	0.66620568	Ajap1	1
GO:0032993	protein-DNA complex	1/69	154/14612	0.00649351	1.37511764	0.32233781	0.51943141	0.71219589	0.67994223	Rpa2	1
GO:0098862	cluster of actin-based cell projections	1/69	155/14612	0.00645161	1.36624591	0.31574542	0.5217249	0.71219589	0.67994223	Tiam1	1
GO:0005912	adherens junction	1/69	161/14612	0.00621118	1.31532991	0.27711924	0.53526102	0.72492044	0.6920905	Ajap1	1
GO:0045177	apical part of cell	2/69	393/14612	0.00508906	1.07770034	0.10755291	0.55752277	0.7433637	0.70969851	Ajap1/Ager	2
GO:0150034	distal axon	2/69	393/14612	0.00508906	1.07770034	0.10755291	0.55752277	0.7433637	0.70969851	Ngef/Tiam1	2
GO:0019898	extrinsic component of membrane	1/69	174/14612	0.00574713	1.21705814	0.19839681	0.5633074	0.74529903	0.71154619	Tiam1	1
GO:0042383	sarcolemma	1/69	178/14612	0.00561798	1.18970852	0.17540461	0.57159652	0.75049314	0.71650508	Slc30a1	1
GO:0001726	ruffle	1/69	187/14612	0.00534759	1.13244982	0.12556013	0.58968428	0.75690819	0.7226296	Tiam1	1
GO:0005741	mitochondrial outer membrane	1/69	187/14612	0.00534759	1.13244982	0.12556013	0.58968428	0.75690819	0.7226296	Fundc2	1
GO:0055037	recycling endosome	1/69	187/14612	0.00534759	1.13244982	0.12556013	0.58968428	0.75690819	0.7226296	Dennd6b	1
GO:0098562	cytoplasmic side of membrane	1/69	190/14612	0.00526316	1.11456903	0.10948858	0.59554463	0.75876798	0.72440517	Ajap1	1
GO:0005681	spliceosomal complex	1/69	194/14612	0.00515464	1.09158823	0.08845564	0.60323029	0.7629089	0.72835856	Luc7l2	1
GO:0030139	endocytic vesicle	1/69	203/14612	0.00492611	1.04319269	0.04268535	0.62000068	0.77163485	0.73668933	H2-T23	1
GO:0000228	nuclear chromosome	1/69	204/14612	0.00490196	1.038079	0.03772561	0.62182037	0.77163485	0.73668933	Rpa2	1
GO:0030027	lamellipodium	1/69	212/14612	0.00471698	0.99890621	-0.001105	0.63607207	0.77163485	0.73668933	Abi3bp	1
GO:0010008	endosome membrane	2/69	456/14612	0.00438596	0.92880753	-0.1063858	0.63914203	0.77163485	0.73668933	Epha8/Ldlrad4	2
GO:0019867	outer membrane	1/69	214/14612	0.0046729	0.98957064	-0.0105865	0.63955148	0.77163485	0.73668933	Fundc2	1
GO:0031968	organelle outer membrane	1/69	214/14612	0.0046729	0.98957064	-0.0105865	0.63955148	0.77163485	0.73668933	Fundc2	1
GO:0005635	nuclear envelope	2/69	458/14612	0.00436681	0.9247516	-0.112701	0.64153362	0.77163485	0.73668933	Cdk2/Slc30a1	2
GO:0098797	plasma membrane protein complex	2/69	463/14612	0.00431965	0.91476508	-0.1283757	0.64745922	0.77328824	0.73826784	H2-T23/Gja4	2
GO:0090575	RNA polymerase II transcription regulator complex	1/69	227/14612	0.00440529	0.93289919	-0.0701816	0.66138188	0.77328824	0.73826784	Nfe2l3	1
GO:0036464	cytoplasmic ribonucleoprotein granule	1/69	229/14612	0.00436681	0.9247516	-0.0790547	0.66462268	0.77328824	0.73826784	Dazap2	1
GO:0030658	transport vesicle membrane	1/69	231/14612	0.004329	0.91674509	-0.0878534	0.6678329	0.77328824	0.73826784	Stx17	1
GO:0000323	lytic vacuole	2/69	487/14612	0.00410678	0.86968425	-0.2014673	0.67484803	0.77328824	0.73826784	Fnip1/Man2b1	2
GO:0005764	lysosome	2/69	487/14612	0.00410678	0.86968425	-0.2014673	0.67484803	0.77328824	0.73826784	Fnip1/Man2b1	2
GO:0005840	ribosome	1/69	240/14612	0.00416667	0.88236715	-0.1265649	0.68190815	0.77328824	0.73826784	Zfp598	1
GO:0030135	coated vesicle	1/69	240/14612	0.00416667	0.88236715	-0.1265649	0.68190815	0.77328824	0.73826784	Stx17	1
GO:0005802	trans-Golgi network	1/69	243/14612	0.00411523	0.87147373	-0.1391616	0.68646799	0.77328824	0.73826784	Sys1	1
GO:0000793	condensed chromosome	1/69	248/14612	0.00403226	0.85390369	-0.1598324	0.69392497	0.77328824	0.73826784	Cdk2	1
GO:0035770	ribonucleoprotein granule	1/69	248/14612	0.00403226	0.85390369	-0.1598324	0.69392497	0.77328824	0.73826784	Dazap2	1
GO:0061695	transferase complex, transferring phosphorus-containing groups	1/69	250/14612	0.004	0.84707246	-0.1679909	0.69685858	0.77328824	0.73826784	Cdk2	1
GO:0034702	monoatomic ion channel complex	1/69	271/14612	0.00369004	0.78143216	-0.2501605	0.72603664	0.80050193	0.76424909	Gabrg1	1
GO:0005765	lysosomal membrane	1/69	280/14612	0.00357143	0.7563147	-0.283591	0.73767733	0.80206971	0.76574586	Fnip1	1
GO:0098852	lytic vacuole membrane	1/69	280/14612	0.00357143	0.7563147	-0.283591	0.73767733	0.80206971	0.76574586	Fnip1	1
GO:0031965	nuclear membrane	1/69	283/14612	0.00353357	0.74829723	-0.2945173	0.74144816	0.80206971	0.76574586	Slc30a1	1
GO:0045121	membrane raft	1/69	299/14612	0.00334448	0.70825457	-0.3510846	0.76067487	0.8134263	0.77658815	Rftn1	1
GO:0098857	membrane microdomain	1/69	301/14612	0.00332226	0.70354856	-0.357964	0.76297686	0.8134263	0.77658815	Rftn1	1
GO:0062023	collagen-containing extracellular matrix	1/69	306/14612	0.00326797	0.69205267	-0.3749864	0.7686368	0.8134263	0.77658815	Abi3bp	1
GO:0043235	receptor complex	1/69	308/14612	0.00324675	0.68755882	-0.3817266	0.7708633	0.8134263	0.77658815	Gabrg1	1
GO:0005938	cell cortex	1/69	328/14612	0.00304878	0.6456345	-0.4470962	0.79199869	0.83063277	0.79301538	Gpsm1	1
GO:0098791	Golgi apparatus subcompartment	1/69	347/14612	0.00288184	0.61028275	-0.5060763	0.81029261	0.84335632	0.80516271	Sys1	1

GO:0005759	mitochondrial matrix	1/69	351/14612	0.002849	0.60332797	-0.5181406	0.81393692	0.84335632	0.80516271	Tst	1
GO:1902495	transmembrane transporter complex	1/69	388/14612	0.00257732	0.54579411	-0.6245903	0.84453756	0.8643447	0.82520057	Gabrg1	1
GO:0030312	external encapsulating structure	1/69	398/14612	0.00251256	0.53208069	-0.6519163	0.85191875	0.8643447	0.82520057	Abi3bp	1
GO:0031012	extracellular matrix	1/69	398/14612	0.00251256	0.53208069	-0.6519163	0.85191875	0.8643447	0.82520057	Abi3bp	1
GO:0030133	transport vesicle	1/69	411/14612	0.00243309	0.51525089	-0.6866193	0.86100001	0.8643447	0.82520057	Stx17	1
GO:1990351	transporter complex	1/69	412/14612	0.00242718	0.51400028	-0.6892519	0.86167539	0.8643447	0.82520057	Gabrg1	1
GO:0005874	microtubule	1/69	416/14612	0.00240385	0.50905797	-0.6997315	0.8643447	0.8643447	0.82520057	Tiam1	1

ID	Description	GeneRatio	BgRatio	RichFactor	FoldEnrichm	zScore	pvalue	p.adjust	qvalue	geneID	Count
GO:0098636	protein complex involved in cell adhesion	2/101	49/14612	0.04081633	5.90503132	2.86923676	0.04508586	0.83742825	0.83742825	Lyn/Jam2	2
GO:0031414	N-terminal protein acetyltransferase complex	1/101	10/14612	0.1	14.4673267	3.5540801	0.06703044	0.83742825	0.83742825	Naa50	1
GO:0097450	astrocyte end-foot	1/101	11/14612	0.09090909	13.1521152	3.36363534	0.07348366	0.83742825	0.83742825	Clcn2	1
GO:1904090	peptidase inhibitor complex	1/101	11/14612	0.09090909	13.1521152	3.36363534	0.07348366	0.83742825	0.83742825	Serpine1	1
GO:1990062	RPAP3/R2TP/prefoldin-like complex	1/101	11/14612	0.09090909	13.1521152	3.36363534	0.07348366	0.83742825	0.83742825	Rpap3	1
GO:0043073	germ cell nucleus	2/101	66/14612	0.03030303	4.3840384	2.29872726	0.07631587	0.83742825	0.83742825	Ankrd37/Ncapd3	2
GO:0001674	female germ cell nucleus	1/101	12/14612	0.08333333	12.0561056	3.19645327	0.07989269	0.83742825	0.83742825	Ncapd3	1
GO:0044666	MLL3/4 complex	1/101	12/14612	0.08333333	12.0561056	3.19645327	0.07989269	0.83742825	0.83742825	Paxip1	1
GO:0098895	postsynaptic endosome membrane	1/101	12/14612	0.08333333	12.0561056	3.19645327	0.07989269	0.83742825	0.83742825	Myo5b	1
GO:0070062	extracellular exosome	2/101	68/14612	0.02941176	4.2550961	2.24454504	0.08034241	0.83742825	0.83742825	Serpine1/Rbmx	2
GO:0030061	mitochondrial crista	1/101	14/14612	0.07142857	10.3338048	2.91492939	0.09257934	0.83742825	0.83742825	Lyn	1
GO:0042583	chromaffin granule	1/101	14/14612	0.07142857	10.3338048	2.91492939	0.09257934	0.83742825	0.83742825	Serpine1	1
GO:0098837	postsynaptic recycling endosome	1/101	14/14612	0.07142857	10.3338048	2.91492939	0.09257934	0.83742825	0.83742825	Myo5b	1
GO:0016586	RSC-type complex	1/101	15/14612	0.06666667	9.64488449	2.79463444	0.09885756	0.83742825	0.83742825	Arid2	1
GO:0030127	COPII vesicle coat	1/101	15/14612	0.06666667	9.64488449	2.79463444	0.09885756	0.83742825	0.83742825	Sar1b	1
GO:1903561	extracellular vesicle	2/101	82/14612	0.02439024	3.52861628	1.91561855	0.11020676	0.83742825	0.83742825	Serpine1/Rbmx	2
GO:0032591	dendritic spine membrane	1/101	18/14612	0.05555556	8.03740374	2.49237585	0.11743533	0.83742825	0.83742825	Clcn2	1
GO:0042575	DNA polymerase complex	1/101	18/14612	0.05555556	8.03740374	2.49237585	0.11743533	0.83742825	0.83742825	Pole	1
GO:0045120	pronucleus	1/101	18/14612	0.05555556	8.03740374	2.49237585	0.11743533	0.83742825	0.83742825	Tet3	1
GO:0098871	postsynaptic actin cytoskeleton	1/101	18/14612	0.05555556	8.03740374	2.49237585	0.11743533	0.83742825	0.83742825	Myo5b	1
GO:0071013	catalytic step 2 spliceosome	2/101	89/14612	0.02247191	3.25108466	1.77709502	0.12607754	0.83742825	0.83742825	Srrm2/Rbmx	2
GO:0043230	extracellular organelle	2/101	90/14612	0.02222222	3.2149615	1.75843454	0.12838795	0.83742825	0.83742825	Serpine1/Rbmx	2
GO:0065010	extracellular membrane-bounded organelle	2/101	90/14612	0.02222222	3.2149615	1.75843454	0.12838795	0.83742825	0.83742825	Serpine1/Rbmx	2
GO:0005811	lipid droplet	2/101	93/14612	0.02150538	3.11125306	1.70398356	0.1353784	0.83742825	0.83742825	Pnpla7/Lss	2
GO:0031528	microvillus membrane	1/101	22/14612	0.04545455	6.57605761	2.18354895	0.14161703	0.83742825	0.83742825	Slc6a6	1
GO:0031248	protein acetyltransferase complex	2/101	96/14612	0.02083333	3.0140264	1.65169283	0.14245261	0.83742825	0.83742825	Brpf1/Naa50	2
GO:0097449	astrocyte projection	1/101	23/14612	0.04347826	6.29014206	2.11821713	0.14755923	0.83742825	0.83742825	Clcn2	1
GO:1902493	acetyltransferase complex	2/101	100/14612	0.02	2.89346535	1.58506002	0.15200429	0.83742825	0.83742825	Brpf1/Naa50	2
GO:0002080	acrosomal membrane	1/101	24/14612	0.04166667	6.02805281	2.05664607	0.15346069	0.83742825	0.83742825	Spaca6	1
GO:0031985	Golgi cisterna	2/101	101/14612	0.01980198	2.86481717	1.56891806	0.15441203	0.83742825	0.83742825	Sar1b/Rab34	2
GO:0005796	Golgi lumen	1/101	25/14612	0.04	5.78693069	1.99846312	0.15932171	0.83742825	0.83742825	Agrn	1
GO:0099571	postsynaptic cytoskeleton	1/101	25/14612	0.04	5.78693069	1.99846312	0.15932171	0.83742825	0.83742825	Myo5b	1
GO:0032585	multivesicular body membrane	1/101	26/14612	0.03846154	5.56435644	1.94334589	0.16514254	0.83742825	0.83742825	Gimap5	1
GO:0016605	PML body	2/101	107/14612	0.01869159	2.70417322	1.4760421	0.16900822	0.83742825	0.83742825	Ciart/Rere	2
GO:0098845	postsynaptic endosome	1/101	27/14612	0.03703704	5.35826916	1.89101381	0.17092347	0.83742825	0.83742825	Myo5b	1
GO:0016514	SWI/SNF complex	1/101	28/14612	0.03571429	5.1669024	1.84122135	0.17666476	0.83742825	0.83742825	Arid2	1
GO:0005640	nuclear outer membrane	1/101	29/14612	0.03448276	4.98873336	1.79375264	0.18236668	0.83742825	0.83742825	Nav3	1
GO:0012507	ER to Golgi transport vesicle membrane	1/101	29/14612	0.03448276	4.98873336	1.79375264	0.18236668	0.83742825	0.83742825	Sar1b	1
GO:0043194	axon initial segment	1/101	29/14612	0.03448276	4.98873336	1.79375264	0.18236668	0.83742825	0.83742825	Clcn2	1
GO:0005721	pericentric heterochromatin	1/101	30/14612	0.03333333	4.82244224	1.74841691	0.18802951	0.83742825	0.83742825	Ncapd3	1
GO:0008305	integrin complex	1/101	30/14612	0.03333333	4.82244224	1.74841691	0.18802951	0.83742825	0.83742825	Lyn	1
GO:0070971	endoplasmic reticulum exit site	1/101	30/14612	0.03333333	4.82244224	1.74841691	0.18802951	0.83742825	0.83742825	Sar1b	1
GO:0071007	U2-type catalytic step 2 spliceosome	1/101	30/14612	0.03333333	4.82244224	1.74841691	0.18802951	0.83742825	0.83742825	Srrm2	1
GO:1990752	microtubule end	1/101	31/14612	0.03225806	4.66687959	1.70504493	0.1936535	0.83742825	0.83742825	Nav3	1
GO:0016363	nuclear matrix	2/101	121/14612	0.01652893	2.39129367	1.28207757	0.20386736	0.83742825	0.83742825	Paxip1/Arid2	2
GO:0034707	chloride channel complex	1/101	35/14612	0.02857143	4.13352192	1.54840537	0.21576636	0.83742825	0.83742825	Clcn2	1
GO:0101031	protein folding chaperone complex	1/101	35/14612	0.02857143	4.13352192	1.54840537	0.21576636	0.83742825	0.83742825	Rpap3	1
GO:0005795	Golgi stack	2/101	134/14612	0.01492537	2.1593025	1.12472635	0.23689089	0.83742825	0.83742825	Sar1b/Rab34	2
GO:0032391	photoreceptor connecting cilium	1/101	39/14612	0.02564103	3.70957096	1.41354727	0.23727865	0.83742825	0.83742825	Alpk1	1
GO:0099738	cell cortex region	1/101	40/14612	0.025	3.61683168	1.38260521	0.24256479	0.83742825	0.83742825	Myo5b	1
GO:0097386	glial cell projection	1/101	43/14612	0.02325581	3.36449459	1.29541722	0.25820652	0.83742825	0.83742825	Clcn2	1
GO:0005814	centriole	2/101	148/14612	0.01351351	1.95504415	0.97423132	0.27275485	0.83742825	0.83742825	Ccdc57/Rab34	2
GO:0034399	nuclear periphery	2/101	148/14612	0.01351351	1.95504415	0.97423132	0.27275485	0.83742825	0.83742825	Paxip1/Arid2	2
GO:0016459	myosin complex	1/101	46/14612	0.02173913	3.14507103	1.21563285	0.27352837	0.83742825	0.83742825	Myo5b	1
GO:0044295	axonal growth cone	1/101	47/14612	0.0212766	3.07815462	1.19048391	0.2785657	0.83742825	0.83742825	Agrn	1
GO:0016607	nuclear speck	4/101	389/14612	0.01028278	1.48764285	0.8132668	0.28223606	0.83742825	0.83742825	Tcim/Fam76a/Srrm2/Ylpm1	4
GO:0030964	NADH dehydrogenase complex	1/101	48/14612	0.02083333	3.0140264	1.16599699	0.28356843	0.83742825	0.83742825	ND5	1
GO:0045271	respiratory chain complex I	1/101	48/14612	0.02083333	3.0140264	1.16599699	0.28356843	0.83742825	0.83742825	ND5	1
GO:0030312	external encapsulating structure	4/101	398/14612	0.01005025	1.45400269	0.76611469	0.29618878	0.83742825	0.83742825	Angptl2/Serpine1/Lrrc17/Agrn	4
GO:0031012	extracellular matrix	4/101	398/14612	0.01005025	1.45400269	0.76611469	0.29618878	0.83742825	0.83742825	Angptl2/Serpine1/Lrrc17/Agrn	4
GO:0000776	kinetochore	2/101	158/14612	0.01265823	1.83130718	0.87649216	0.29836637	0.83742825	0.83742825	Ncapd3/Arid2	2
GO:0032590	dendrite membrane	1/101	51/14612	0.01960784	2.83673073	1.09619285	0.29837148	0.83742825	0.83742825	Clcn2	1
GO:0005771	multivesicular body	1/101	52/14612	0.01923077	2.78217822	1.07404902	0.30323821	0.83742825	0.83742825	Gimap5	1
GO:0030670	phagocytic vesicle membrane	1/101	52/14612	0.01923077	2.78217822	1.07404902	0.30323821	0.83742825	0.83742825	Rab34	1
GO:0071005	U2-type precatalytic spliceosome	1/101	53/14612	0.01886792	2.72968429	1.05242459	0.30807152	0.83742825	0.83742825	Srrm2	1
GO:0001673	male germ cell nucleus	1/101	54/14612	0.01851852	2.67913458	1.03129643	0.31287162	0.83742825	0.83742825	Ankrd37	1
GO:0015030	Cajal body	1/101	54/14612	0.01851852	2.67913458	1.03129643	0.31287162	0.83742825	0.83742825	Srrm2	1
GO:0071011	precatalytic spliceosome	1/101	55/14612	0.01818182	2.63042304	1.01064282	0.31763876	0.83742825	0.83742825	Srrm2	1
GO:0099091	postsynaptic specialization, intracellular component	1/101	56/14612	0.01785714	2.5834512	0.9904434	0.32237315	0.83742825	0.83742825	Lyn	1
GO:0000779	condensed chromosome, centromeric region	2/101	168/14612	0.01190476	1.7223008	0.78556293	0.32384525	0.83742825	0.83742825	Ncapd3/Arid2	2
GO:0030134	COPII-coated ER to Golgi transport vesicle	1/101	57/14612	0.01754386	2.5381275	0.97067902	0.32707501	0.83742825	0.83742825	Sar1b	1
GO:0030120	vesicle coat	1/101	59/14612	0.01694915	2.45208928	0.9323845	0.33638204	0.83742825	0.83742825	Sar1b	1
GO:0000794	condensed nuclear chromosome	1/101	60/14612	0.01666667	2.41122112	0.91382143	0.34098765	0.83742825	0.83742825	Ncapd3	1
GO:0000791	euchromatin	1/101	61/14612	0.01639344	2.37169291	0.89562741	0.34556161	0.83742825	0.83742825	Rbmx	1
GO:0032580	Golgi cisterna membrane	1/101	63/14612	0.01587302	2.29640107	0.86029039	0.35461544	0.83742825	0.83742825	Sar1b	1
GO:0062023	collagen-containing extracellular matrix	3/101	306/14612	0.00980392	1.41836537	0.61703431	0.35485639	0.83742825	0.83742825	Angptl2/Serpine1/Agrn	3
GO:0001726	ruffle	2/101	187/14612	0.01069519	1.54730767	0.62841481	0.37155455	0.83742825	0.83742825	Rab34/Abl1	2
GO:0005657	replication fork	1/101	70/14612	0.01428571	2.06676096	0.74637322	0.38533807	0.83742825	0.83742825	Xrcc3	1
GO:0005681	spliceosomal complex	2/101	194/14612	0.01030928	1.49147698	0.57491485	0.38881427	0.83742825	0.83742825	Srrm2/Rbmx	2
GO:0035097	histone methyltransferase complex	1/101	71/14612	0.01408451	2.03765165	0.73119903	0.38960715	0.83742825	0.83742825	Paxip1	1
GO:0035869	ciliary transition zone	1/101	71/14612	0.01408451	2.03765165	0.73119903	0.38960715	0.83742825	0.83742825	Alpk1	1
GO:0048787	presynaptic active zone membrane	1/101	71/14612	0.01408451	2.03765165	0.73119903	0.38960715	0.83742825	0.83742825	Celsr3	1
GO:0005667	transcription regulator complex	4/101	458/14612	0.00873362	1.26352199	0.47803752	0.39052941	0.83742825	0.83742825	Dbp/Hlf/Bhlhe41/Arid2	4
GO:0030666	endocytic vesicle membrane	1/101	73/14612	0.01369863	1.98182558	0.70158532	0.39805742	0.83742825	0.83742825	Rab34	1
GO:0017053	transcription repressor complex	1/101	74/14612	0.01351351	1.95504415	0.68713009	0.40223901	0.83742825	0.83742825	Bhlhe41	1
GO:0032589	neuron projection membrane	1/101	74/14612	0.01351351	1.95504415	0.68713009	0.40223901	0.83742825	0.83742825	Clcn2	1
GO:0032040	small-subunit processome	1/101	75/14612	0.01333333	1.9289769	0.67289939	0.40639184	0.83742825	0.83742825	Wdr46	1
GO:0000228	nuclear chromosome	2/101	204/14612	0.00980392	1.41836537	0.50201991	0.41310767	0.83742825	0.83742825	Pole/Ncapd3	2
GO:0005876	spindle microtubule	1/101	77/14612	0.01298701	1.8788736	0.64508397	0.414612	0.83742825	0.83742825	Ccdc57	1
GO:0030667	secretory granule membrane	1/101	77/14612	0.01298701	1.8788736	0.64508397	0.414612	0.83742825	0.83742825	Spaca6	1
GO:0097733	photoreceptor cell cilium	1/101	79/14612	0.01265823	1.83130718	0.61808674	0.42271943	0.83742825	0.83742825	Alpk1	1
GO:0000118	histone deacetylase complex	1/101	81/14612	0.01234568	1.78608972	0.59185982	0.43071568	0.83742825	0.83742825	Rere	1
GO:0044291	cell-cell contact zone	1/101	82/14612	0.01219512	1.76430814	0.57902137	0.43467258	0.83742825	0.83742825	Jam2	1
GO:0045171	intercellular bridge	1/101	82/14612	0.01219512	1.76430814	0.57902137	0.43467258	0.83742825	0.83742825	Fan1	1
GO:0099572	postsynaptic specialization	4/101	487/14612	0.00821355	1.18828146	0.35255736	0.43585237	0.83742825	0.83742825	Abl1/Rbmx/Celsr3/Lyn	4
GO:0044304	main axon	1/101	85/14612	0.01176471	1.70203844	0.54154478	0.44638062	0.83742825	0.83742825	Clcn2	1
GO:0000123	histone acetyltransferase complex	1/101	86/14612	0.01162791	1.68224729	0.52938303	0.4502297	0.83742825	0.83742825	Brpf1	1
GO:0005902	microvillus	1/101	87/14612	0.01149425	1.66291112	0.51737906	0.45405228	0.83742825	0.83742825	Slc6a6	1
GO:0000775	chromosome, centromeric region	2/101	225/14612	0.00888889	1.2859846	0.36066232	0.46252058	0.83742825	0.83742825	Ncapd3/Arid2	2
GO:0045211	postsynaptic membrane	3/101	365/14612	0.00821918	1.18909535	0.30522321	0.46418795	0.83742825	0.83742825	Slc6a6/Clcn2/Celsr3	3
GO:0000792	heterochromatin	1/101	90/14612	0.01111111	1.60748075	0.48227255	0.46536284	0.83742825	0.83742825	Ncapd3	1
GO:0043197	dendritic spine	2/101	227/14612	0.00881057	1.27465434	0.34793366	0.46710249	0.83742825	0.83742825	Myo5b/Clcn2	2
GO:0090575	RNA polymerase II transcription regulator complex	2/101	227/14612	0.00881057	1.27465434	0.34793366	0.46710249	0.83742825	0.83742825	Dbp/Hlf	2
GO:0097731	9+0 non-motile cilium	1/101	92/14612	0.01086957	1.57253551	0.45958413	0.472774	0.83742825	0.83742825	Alpk1	1
GO:0035861	site of double-strand break	1/101	93/14612	0.01075269	1.55562653	0.44844386	0.47644134	0.83742825	0.83742825	Peli1	1
GO:0030426	growth cone	2/101	232/14612	0.00862069	1.24718334	0.31661809	0.47845712	0.83742825	0.83742825	Abl1/Agrn	2
GO:0044309	neuron spine	2/101	232/14612	0.00862069	1.24718334	0.31661809	0.47845712	0.83742825	0.83742825	Myo5b/Clcn2	2
GO:0070603	SWI/SNF superfamily-type complex	1/101	94/14612	0.0106383	1.53907731	0.43743504	0.48008342	0.83742825	0.83742825	Arid2	1
GO:0016323	basolateral plasma membrane	2/101	235/14612	0.00851064	1.23126185	0.29816439	0.48520004	0.83742825	0.83742825	Slc6a6/Clcn2	2
GO:0005684	U2-type spliceosomal complex	1/101	96/14612	0.01041667	1.5070132	0.41579895	0.4872925	0.83742825	0.83742825	Srrm2	1
GO:0030117	membrane coat	1/101	96/14612	0.01041667	1.5070132	0.41579895	0.4872925	0.83742825	0.83742825	Sar1b	1
GO:0048475	coated membrane	1/101	96/14612	0.01041667	1.5070132	0.41579895	0.4872925	0.83742825	0.83742825	Sar1b	1
GO:0030427	site of polarized growth	2/101	238/14612	0.00840336	1.21574174	0.27995345	0.49188966	0.83742825	0.83742825	Abl1/Agrn	2
GO:0005604	basement membrane	1/101	98/14612	0.01020408	1.47625783	0.39465135	0.4944026	0.83742825	0.83742825	Agrn	1
GO:0001669	acrosomal vesicle	1/101	99/14612	0.01010101	1.46134613	0.38425354	0.49792095	0.83742825	0.83742825	Spaca6	1
GO:0005903	brush border	1/101	102/14612	0.00980392	1.41836537	0.35373179	0.5083312	0.83742825	0.83742825	Myo5b	1
GO:0098803	respiratory chain complex	1/101	103/14612	0.00970874	1.40459483	0.34377333	0.51175357	0.83742825	0.83742825	ND5	1
GO:0000793	condensed chromosome	2/101	248/14612	0.00806452	1.1667199	0.2209162	0.51379509	0.83742825	0.83742825	Ncapd3/Arid2	2
GO:0005923	bicellular tight junction	1/101	104/14612	0.00961538	1.39108911	0.33391859	0.51515235	0.83742825	0.83742825	Jam2	1
GO:0034708	methyltransferase complex	1/101	104/14612	0.00961538	1.39108911	0.33391859	0.51515235	0.83742825	0.83742825	Paxip1	1
GO:0030684	preribosome	1/101	107/14612	0.00934579	1.35208661	0.30495413	0.52520877	0.83742825	0.83742825	Wdr46	1
GO:0009925	basal plasma membrane	2/101	258/14612	0.00775194	1.1214982	0.16426506	0.53507988	0.83742825	0.83742825	Slc6a6/Clcn2	2
GO:0034451	centriolar satellite	1/101	112/14612	0.00892857	1.2917256	0.25855417	0.54151251	0.83742825	0.83742825	Ccdc57	1
GO:0070160	tight junction	1/101	112/14612	0.00892857	1.2917256	0.25855417	0.54151251	0.83742825	0.83742825	Jam2	1
GO:0045335	phagocytic vesicle	1/101	117/14612	0.00854701	1.23652365	0.21429418	0.55726176	0.83742825	0.83742825	Rab34	1
GO:0097730	non-motile cilium	1/101	117/14612	0.00854701	1.23652365	0.21429418	0.55726176	0.83742825	0.83742825	Alpk1	1
GO:0005770	late endosome	2/101	270/14612	0.00740741	1.07165383	0.09914446	0.55978039	0.83742825	0.83742825	Gimap5/Rab34	2
GO:0045178	basal part of cell	2/101	275/14612	0.00727273	1.05216922	0.07286241	0.56979716	0.83742825	0.83742825	Slc6a6/Clcn2	2
GO:0090734	site of DNA damage	1/101	122/14612	0.00819672	1.18584645	0.17196972	0.57247519	0.83742825	0.83742825	Peli1	1
GO:0030662	coated vesicle membrane	1/101	123/14612	0.00813008	1.17620543	0.16372108	0.57545517	0.83742825	0.83742825	Sar1b	1
GO:1904949	ATPase complex	1/101	123/14612	0.00813008	1.17620543	0.16372108	0.57545517	0.83742825	0.83742825	Arid2	1
GO:0005765	lysosomal membrane	2/101	280/14612	0.00714286	1.03338048	0.04705114	0.5796507	0.83742825	0.83742825	Gimap5/Sar1b	2
GO:0098852	lytic vacuole membrane	2/101	280/14612	0.00714286	1.03338048	0.04705114	0.5796507	0.83742825	0.83742825	Gimap5/Sar1b	2
GO:0031965	nuclear membrane	2/101	283/14612	0.00706714	1.02242592	0.03178246	0.58548422	0.83742825	0.83742825	Il15ra/Nav3	2
GO:0043296	apical junction complex	1/101	127/14612	0.00787402	1.13915959	0.13140374	0.58717085	0.83742825	0.83742825	Jam2	1
GO:0014069	postsynaptic density	3/101	443/14612	0.00677201	0.97972867	-0.0361465	0.59495811	0.83742825	0.83742825	Abl1/Rbmx/Celsr3	3
GO:1990204	oxidoreductase complex	1/101	130/14612	0.00769231	1.11287129	0.10784323	0.595747	0.83742825	0.83742825	ND5	1
GO:0005777	peroxisome	1/101	131/14612	0.00763359	1.10437609	0.10011274	0.59856633	0.83742825	0.83742825	Pipox	1
GO:0042579	microbody	1/101	131/14612	0.00763359	1.10437609	0.10011274	0.59856633	0.83742825	0.83742825	Pipox	1
GO:0044853	plasma membrane raft	1/101	131/14612	0.00763359	1.10437609	0.10011274	0.59856633	0.83742825	0.83742825	Trem2	1
GO:0031252	cell leading edge	3/101	454/14612	0.00660793	0.95599075	-0.0794736	0.61169955	0.83742825	0.83742825	Rab34/Clcn2/Abl1	3
GO:0048786	presynaptic active zone	1/101	136/14612	0.00735294	1.06377402	0.06233629	0.6123736	0.83742825	0.83742825	Celsr3	1
GO:0045121	membrane raft	2/101	299/14612	0.00668896	0.96771416	-0.0470581	0.61559745	0.83742825	0.83742825	Trem2/Lyn	2
GO:0098857	membrane microdomain	2/101	301/14612	0.00664452	0.96128417	-0.0566225	0.61924328	0.83742825	0.83742825	Trem2/Lyn	2
GO:0032279	asymmetric synapse	3/101	465/14612	0.00645161	0.93337592	-0.1218085	0.62798146	0.83742825	0.83742825	Abl1/Rbmx/Celsr3	3
GO:0005911	cell-cell junction	3/101	467/14612	0.00642398	0.92937859	-0.1294033	0.63089189	0.83742825	0.83742825	Fzd4/Lyn/Jam2	3
GO:0098685	Schaffer collateral - CA1 synapse	1/101	144/14612	0.00694444	1.00467547	0.00470386	0.6334935	0.83742825	0.83742825	Myo5b	1
GO:0009898	cytoplasmic side of plasma membrane	1/101	147/14612	0.00680272	0.98417189	-0.0160909	0.64111623	0.83742825	0.83742825	Lyn	1
GO:0098802	plasma membrane signaling receptor complex	1/101	147/14612	0.00680272	0.98417189	-0.0160909	0.64111623	0.83742825	0.83742825	Lyn	1
GO:0098839	postsynaptic density membrane	1/101	147/14612	0.00680272	0.98417189	-0.0160909	0.64111623	0.83742825	0.83742825	Celsr3	1
GO:0030141	secretory granule	2/101	318/14612	0.00628931	0.90989476	-0.135531	0.64917581	0.83742825	0.83742825	Serpine1/Spaca6	2
GO:0031902	late endosome membrane	1/101	152/14612	0.00657895	0.95179781	-0.0498375	0.65347346	0.83742825	0.83742825	Gimap5	1
GO:0098687	chromosomal region	2/101	321/14612	0.00623053	0.90139107	-0.1490354	0.65426276	0.83742825	0.83742825	Ncapd3/Arid2	2
GO:0000323	lytic vacuole	3/101	487/14612	0.00616016	0.89121109	-0.2037074	0.65914257	0.83742825	0.83742825	Mt1/Gimap5/Sar1b	3
GO:0005764	lysosome	3/101	487/14612	0.00616016	0.89121109	-0.2037074	0.65914257	0.83742825	0.83742825	Mt1/Gimap5/Sar1b	3
GO:0031253	cell projection membrane	2/101	324/14612	0.00617284	0.89304486	-0.1624203	0.65929158	0.83742825	0.83742825	Slc6a6/Clcn2	2
GO:0098862	cluster of actin-based cell projections	1/101	155/14612	0.00645161	0.93337592	-0.0695681	0.6606846	0.83742825	0.83742825	Myo5b	1
GO:0098982	GABA-ergic synapse	1/101	158/14612	0.00632911	0.91565359	-0.088931	0.66774713	0.84032384	0.84032384	Slc6a6	1
GO:0005912	adherens junction	1/101	161/14612	0.00621118	0.89859172	-0.1079417	0.67466408	0.84032384	0.84032384	Lyn	1
GO:0005774	vacuolar membrane	2/101	334/14612	0.00598802	0.86630699	-0.2062055	0.67563726	0.84032384	0.84032384	Gimap5/Sar1b	2
GO:0000922	spindle pole	1/101	167/14612	0.00598802	0.86630699	-0.144964	0.68807309	0.85047544	0.85047544	Alpk1	1
GO:0098791	Golgi apparatus subcompartment	2/101	347/14612	0.00576369	0.83385168	-0.2613225	0.69593974	0.85488894	0.85488894	Sar1b/Rab34	2
GO:0042383	sarcolemma	1/101	178/14612	0.00561798	0.81277116	-0.2096725	0.71125051	0.86012934	0.86012934	Agrn	1
GO:0099634	postsynaptic specialization membrane	1/101	180/14612	0.00555556	0.80374037	-0.2210324	0.71527748	0.86012934	0.86012934	Celsr3	1
GO:0005925	focal adhesion	1/101	187/14612	0.00534759	0.77365384	-0.2598891	0.7289389	0.86012934	0.86012934	Tns2	1

GO:0055037 recycling endosome	1/101	187/14612	0.00534759	0.77365384	-0.2598891	0.7289389	0.86012934	0.86012934	Myo5b	1
GO:0098562 cytoplasmic side of membrane	1/101	190/14612	0.00526316	0.76143825	-0.2761321	0.73459321	0.86012934	0.86012934	Lyn	1
GO:0031256 leading edge membrane	1/101	192/14612	0.00520833	0.7535066	-0.2868305	0.73829771	0.86012934	0.86012934	Clcn2	1
GO:0043204 perikaryon	1/101	196/14612	0.00510204	0.73812891	-0.3079252	0.74555381	0.86012934	0.86012934	Clcn2	1
GO:0030055 cell-substrate junction	1/101	199/14612	0.00502513	0.72700134	-0.3234907	0.75086482	0.86012934	0.86012934	Tns2	1
GO:1902495 transmembrane transporter complex	2/101	388/14612	0.00515464	0.74573849	-0.4234846	0.75324046	0.86012934	0.86012934	ND5/Clcn2	2
GO:0030139 endocytic vesicle	1/101	203/14612	0.00492611	0.71267619	-0.3439178	0.75777579	0.86012934	0.86012934	Rab34	1
GO:0045177 apical part of cell	2/101	393/14612	0.00508906	0.73625072	-0.442186	0.75956052	0.86012934	0.86012934	Slc6a6/Myo5b	2
GO:0150034 distal axon	2/101	393/14612	0.00508906	0.73625072	-0.442186	0.75956052	0.86012934	0.86012934	Abl1/Agrn	2
GO:0005819 spindle	2/101	394/14612	0.00507614	0.73438207	-0.4459008	0.76080782	0.86012934	0.86012934	Ccdc57/Alpk1	2
GO:0043209 myelin sheath	1/101	210/14612	0.0047619	0.68892032	-0.3788104	0.76941625	0.86012934	0.86012934	Clcn2	1
GO:0019867 outer membrane	1/101	214/14612	0.0046729	0.67604331	-0.3982857	0.77581744	0.86012934	0.86012934	Nav3	1
GO:0031968 organelle outer membrane	1/101	214/14612	0.0046729	0.67604331	-0.3982857	0.77581744	0.86012934	0.86012934	Nav3	1
GO:0098858 actin-based cell projection	1/101	216/14612	0.00462963	0.66978365	-0.4079026	0.77895173	0.86012934	0.86012934	Slc6a6	1
GO:0030133 transport vesicle	2/101	411/14612	0.00486618	0.70400617	-0.507804	0.78117852	0.86012934	0.86012934	Sar1b/Galnt15	2
GO:1990351 transporter complex	2/101	412/14612	0.00485437	0.70229741	-0.5113745	0.7823287	0.86012934	0.86012934	ND5/Clcn2	2
GO:0005874 microtubule	2/101	416/14612	0.00480769	0.69554455	-0.5255807	0.78687719	0.8603767	0.8603767	Ccdc57/Nav3	2
GO:0036464 cytoplasmic ribonucleoprotein granule	1/101	229/14612	0.00436681	0.63176099	-0.4685703	0.79829314	0.86642452	0.86642452	Eid1	1
GO:0030658 transport vesicle membrane	1/101	231/14612	0.004329	0.6262912	-0.4776357	0.80111614	0.86642452	0.86642452	Sar1b	1
GO:0030135 coated vesicle	1/101	240/14612	0.00416667	0.60280528	-0.5176098	0.81334315	0.86915271	0.86915271	Sar1b	1
GO:0035770 ribonucleoprotein granule	1/101	248/14612	0.00403226	0.58335995	-0.5520789	0.82358499	0.86915271	0.86915271	Eid1	1
GO:0042734 presynaptic membrane	1/101	250/14612	0.004	0.57869307	-0.5605484	0.82605727	0.86915271	0.86915271	Celsr3	1
GO:0061695 transferase complex, transferring phosphorus-containing groups	1/101	250/14612	0.004	0.57869307	-0.5605484	0.82605727	0.86915271	0.86915271	Pole	1
GO:0010008 endosome membrane	2/101	456/14612	0.00438596	0.63453187	-0.6614753	0.8279868	0.86915271	0.86915271	Gimap5/Myo5b	2
GO:0005635 nuclear envelope	2/101	458/14612	0.00436681	0.63176099	-0.6679976	0.8298443	0.86915271	0.86915271	Il15ra/Nav3	2
GO:0098797 plasma membrane protein complex	2/101	463/14612	0.00431965	0.62493852	-0.6841984	0.83441017	0.86935928	0.86935928	Slc6a6/Lyn	2
GO:0099568 cytoplasmic region	1/101	270/14612	0.0037037	0.53582692	-0.6422572	0.84896879	0.87645826	0.87645826	Myo5b	1
GO:0034702 monoatomic ion channel complex	1/101	271/14612	0.00369004	0.53384969	-0.6462088	0.85003239	0.87645826	0.87645826	Clcn2	1
GO:0015629 actin cytoskeleton	2/101	495/14612	0.0040404	0.58453845	-0.7845384	0.8611242	0.88331813	0.88331813	Myo5b/Abl1	2
GO:0043235 receptor complex	1/101	308/14612	0.00324675	0.4697184	-0.7847027	0.88457905	0.90272426	0.90272426	Lyn	1
GO:0016324 apical plasma membrane	1/101	319/14612	0.0031348	0.45352121	-0.8233016	0.89323689	0.90690888	0.90690888	Slc6a6	1
GO:0005938 cell cortex	1/101	328/14612	0.00304878	0.44107703	-0.8541142	0.89983977	0.9089752	0.9089752	Myo5b	1
GO:0005759 mitochondrial matrix	1/101	351/14612	0.002849	0.41217455	-0.9299914	0.91493312	0.919554	0.919554	Xrcc3	1
GO:0005769 early endosome	1/101	399/14612	0.00250627	0.36258964	-1.0769974	0.93955616	0.93955616	0.93955616	Rab34	1

ID	Description	GeneRatio	BgRatio	RichFactor	FoldEnrichm	zScore	pvalue	p.adjust	qvalue	geneID	Count
GO:0097136	Bcl-2 family protein complex	1/48	10/14612	0.1	30.4416667	5.34658497	0.03237817	0.49346845	0.49346845	Bcl2l1	1
GO:0061700	GATOR2 complex	1/48	11/14612	0.09090909	27.6742424	5.08062791	0.03555896	0.49346845	0.49346845	Sesn1	1
GO:0044322	endoplasmic reticulum quality control compartment	1/48	13/14612	0.07692308	23.4166667	4.64196017	0.04188985	0.49346845	0.49346845	Herpud1	1
GO:0035859	Seh1-associated complex	1/48	15/14612	0.06666667	20.2944444	4.29206566	0.04818004	0.49346845	0.49346845	Sesn1	1
GO:0097730	non-motile cilium	2/48	117/14612	0.01709402	5.2037037	2.62081383	0.05648398	0.49346845	0.49346845	Mchr1/Mertk	2
GO:0098871	postsynaptic actin cytoskeleton	1/48	18/14612	0.05555556	16.912037	3.8778867	0.05753958	0.49346845	0.49346845	Fam107a	1
GO:0032039	integrator complex	1/48	19/14612	0.05263158	16.0219298	3.76140842	0.06063935	0.49346845	0.49346845	Ints1	1
GO:0031965	nuclear membrane	3/48	283/14612	0.01060071	3.2270318	2.17186061	0.06572639	0.49346845	0.49346845	Bcl2l1/Ints1/Maco1	3
GO:0005777	peroxisome	2/48	131/14612	0.01526718	4.6475827	2.40747492	0.06887554	0.49346845	0.49346845	Xdh/Pxmp2	2
GO:0042579	microbody	2/48	131/14612	0.01526718	4.6475827	2.40747492	0.06887554	0.49346845	0.49346845	Xdh/Pxmp2	2
GO:0033268	node of Ranvier	1/48	24/14612	0.04166667	12.6840278	3.28867213	0.0759891	0.49346845	0.49346845	Kcnk4	1
GO:0099571	postsynaptic cytoskeleton	1/48	25/14612	0.04	12.1766667	3.21084666	0.07902944	0.49346845	0.49346845	Fam107a	1
GO:0001650	fibrillar center	2/48	144/14612	0.01388889	4.22800926	2.23476583	0.08110414	0.49346845	0.49346845	Sesn1/Ip6k2	2
GO:0001891	phagocytic cup	1/48	27/14612	0.03703704	11.2746914	3.06773403	0.08508077	0.49346845	0.49346845	Trip10	1
GO:0030867	rough endoplasmic reticulum membrane	1/48	27/14612	0.03703704	11.2746914	3.06773403	0.08508077	0.49346845	0.49346845	Maco1	1
GO:0005925	focal adhesion	2/48	187/14612	0.01069519	3.25579323	1.78230668	0.12557448	0.53676081	0.53676081	Fam107a/Ppfia1	2
GO:0016607	nuclear speck	3/48	389/14612	0.00771208	2.34768638	1.54663661	0.1352878	0.53676081	0.53676081	Hif3a/Fam107a/Klf15	3
GO:0030055	cell-substrate junction	2/48	199/14612	0.01005025	3.05946399	1.67928265	0.13886389	0.53676081	0.53676081	Fam107a/Ppfia1	2
GO:0044306	neuron projection terminus	2/48	204/14612	0.00980392	2.98447712	1.63862503	0.14449134	0.53676081	0.53676081	Fstl3/Maco1	2
GO:0060170	ciliary membrane	1/48	54/14612	0.01851852	5.63734568	1.95990828	0.16306093	0.53676081	0.53676081	Mchr1	1
GO:0001750	photoreceptor outer segment	1/48	55/14612	0.01818182	5.53484848	1.93432052	0.16582045	0.53676081	0.53676081	Mertk	1
GO:0005778	peroxisomal membrane	1/48	56/14612	0.01785714	5.4360119	1.90935176	0.16857106	0.53676081	0.53676081	Pxmp2	1
GO:0005791	rough endoplasmic reticulum	1/48	56/14612	0.01785714	5.4360119	1.90935176	0.16857106	0.53676081	0.53676081	Maco1	1
GO:0031903	microbody membrane	1/48	56/14612	0.01785714	5.4360119	1.90935176	0.16857106	0.53676081	0.53676081	Pxmp2	1
GO:0043197	dendritic spine	2/48	227/14612	0.00881057	2.68208517	1.46631147	0.17095703	0.53676081	0.53676081	Ppfia1/Mtmr2	2
GO:0044309	neuron spine	2/48	232/14612	0.00862069	2.62428161	1.43168057	0.17681889	0.53676081	0.53676081	Ppfia1/Mtmr2	2
GO:0005635	nuclear envelope	3/48	458/14612	0.00655022	1.99399563	1.24078883	0.19004102	0.53676081	0.53676081	Bcl2l1/Ints1/Maco1	3
GO:0005667	transcription regulator complex	3/48	458/14612	0.00655022	1.99399563	1.24078883	0.19004102	0.53676081	0.53676081	Per2/Tead3/Mxi1	3
GO:0005905	clathrin-coated pit	1/48	68/14612	0.01470588	4.47671569	1.64968853	0.20089358	0.53676081	0.53676081	Bcl2l1	1
GO:0016234	inclusion body	1/48	73/14612	0.01369863	4.17009132	1.5587849	0.21399519	0.53676081	0.53676081	Herpud1	1
GO:0017053	transcription repressor complex	1/48	74/14612	0.01351351	4.11373874	1.54157959	0.21659016	0.53676081	0.53676081	Per2	1
GO:0097733	photoreceptor cell cilium	1/48	79/14612	0.01265823	3.85337553	1.45987306	0.22943968	0.53676081	0.53676081	Mertk	1
GO:0008021	synaptic vesicle	2/48	277/14612	0.00722022	2.19795427	1.15558403	0.23075946	0.53676081	0.53676081	Bcl2l1/Mtmr2	2
GO:0016529	sarcoplasmic reticulum	1/48	80/14612	0.0125	3.80520833	1.44433413	0.23198471	0.53676081	0.53676081	Xdh	1
GO:0034705	potassium channel complex	1/48	85/14612	0.01176471	3.58137255	1.37022673	0.24458691	0.53676081	0.53676081	Kcnk4	1
GO:0044304	main axon	1/48	85/14612	0.01176471	3.58137255	1.37022673	0.24458691	0.53676081	0.53676081	Kcnk4	1
GO:0070382	exocytic vesicle	2/48	293/14612	0.00682594	2.07792947	1.07001056	0.25025875	0.53676081	0.53676081	Bcl2l1/Mtmr2	2
GO:0009897	external side of plasma membrane	2/48	298/14612	0.00671141	2.04306488	1.04438157	0.25636805	0.53676081	0.53676081	Cd163/Icosl	2
GO:0097731	9+0 non-motile cilium	1/48	92/14612	0.01086957	3.30887681	1.27535656	0.26189041	0.53676081	0.53676081	Mertk	1
GO:0005811	lipid droplet	1/48	93/14612	0.01075269	3.27329749	1.26255309	0.26433044	0.53676081	0.53676081	Rdh10	1
GO:0042470	melanosome	1/48	98/14612	0.01020408	3.10629252	1.20104262	0.27641262	0.53676081	0.53676081	Slc1a5	1
GO:0048770	pigment granule	1/48	98/14612	0.01020408	3.10629252	1.20104262	0.27641262	0.53676081	0.53676081	Slc1a5	1
GO:0032587	ruffle membrane	1/48	99/14612	0.01010101	3.07491582	1.18921324	0.27880563	0.53676081	0.53676081	Fam107a	1
GO:0001725	stress fiber	1/48	100/14612	0.01	3.04416667	1.17753255	0.2811909	0.53676081	0.53676081	Fam107a	1
GO:0097517	contractile actin filament bundle	1/48	100/14612	0.01	3.04416667	1.17753255	0.2811909	0.53676081	0.53676081	Fam107a	1
GO:0031253	cell projection membrane	2/48	324/14612	0.00617284	1.87911523	0.91865584	0.28816855	0.53676081	0.53676081	Fam107a/Mchr1	2
GO:0016528	sarcoplasm	1/48	108/14612	0.00925926	2.81867284	1.08903746	0.29999695	0.53676081	0.53676081	Xdh	1
GO:0032432	actin filament bundle	1/48	109/14612	0.00917431	2.79281346	1.07854851	0.30231356	0.53676081	0.53676081	Fam107a	1
GO:0042641	actomyosin	1/48	109/14612	0.00917431	2.79281346	1.07854851	0.30231356	0.53676081	0.53676081	Fam107a	1
GO:0005759	mitochondrial matrix	2/48	351/14612	0.00569801	1.7345679	0.79970676	0.32107577	0.55867184	0.55867184	Prodh/Bcl2l1	2
GO:0048786	presynaptic active zone	1/48	136/14612	0.00735294	2.23835784	0.8329359	0.36210079	0.61770135	0.61770135	Ppfia1	1
GO:0098685	Schaffer collateral - CA1 synapse	1/48	144/14612	0.00694444	2.11400463	0.77123059	0.37883105	0.63381348	0.63381348	Ppfia1	1
GO:0030133	transport vesicle	2/48	411/14612	0.00486618	1.48134631	0.56824987	0.39275762	0.64471534	0.64471534	Bcl2l1/Mtmr2	2
GO:0030672	synaptic vesicle membrane	1/48	164/14612	0.00609756	1.85619919	0.63301372	0.41879955	0.64780735	0.64780735	Bcl2l1	1
GO:0099501	exocytic vesicle membrane	1/48	164/14612	0.00609756	1.85619919	0.63301372	0.41879955	0.64780735	0.64780735	Bcl2l1	1
GO:0014069	postsynaptic density	2/48	443/14612	0.00451467	1.37434161	0.45932557	0.42970113	0.64780735	0.64780735	Pcbp2/Mtmr2	2
GO:0036064	ciliary basal body	1/48	170/14612	0.00588235	1.79068627	0.59529913	0.43029111	0.64780735	0.64780735	Cfap157	1
GO:0031901	early endosome membrane	1/48	174/14612	0.00574713	1.74952107	0.57098567	0.43782824	0.64780735	0.64780735	Mtmr2	1
GO:0034703	cation channel complex	1/48	176/14612	0.00568182	1.72964015	0.55906445	0.4415601	0.64780735	0.64780735	Kcnk4	1
GO:0032279	asymmetric synapse	2/48	465/14612	0.00430108	1.309319	0.38915368	0.45444123	0.64780735	0.64780735	Pcbp2/Mtmr2	2
GO:0001726	ruffle	1/48	187/14612	0.00534759	1.62789661	0.49610235	0.46165581	0.64780735	0.64780735	Fam107a	1
GO:0005741	mitochondrial outer membrane	1/48	187/14612	0.00534759	1.62789661	0.49610235	0.46165581	0.64780735	0.64780735	Bcl2l1	1
GO:0031256	leading edge membrane	1/48	192/14612	0.00520833	1.58550347	0.4688325	0.47055448	0.64981333	0.64981333	Fam107a	1
GO:0099572	postsynaptic specialization	2/48	487/14612	0.00410678	1.25017112	0.32235	0.47858822	0.65058086	0.65058086	Pcbp2/Mtmr2	2
GO:0019867	outer membrane	1/48	214/14612	0.0046729	1.42250779	0.35744627	0.50802744	0.66967254	0.66967254	Bcl2l1	1
GO:0031968	organelle outer membrane	1/48	214/14612	0.0046729	1.42250779	0.35744627	0.50802744	0.66967254	0.66967254	Bcl2l1	1
GO:0090575	RNA polymerase II transcription regulator complex	1/48	227/14612	0.00440529	1.34104258	0.29729449	0.52893643	0.68473232	0.68473232	Mxi1	1
GO:0030658	transport vesicle membrane	1/48	231/14612	0.004329	1.31782107	0.27952096	0.53519308	0.68473232	0.68473232	Bcl2l1	1
GO:0009925	basal plasma membrane	1/48	258/14612	0.00387597	1.17990956	0.16737781	0.57534774	0.72543846	0.72543846	Slc1a5	1
GO:0034702	monoatomic ion channel complex	1/48	271/14612	0.00369004	1.12330873	0.11762754	0.59344984	0.73382185	0.73382185	Kcnk4	1
GO:0045178	basal part of cell	1/48	275/14612	0.00363636	1.1069697	0.10280594	0.5988661	0.73382185	0.73382185	Slc1a5	1
GO:0062023	collagen-containing extracellular matrix	1/48	306/14612	0.00326797	0.99482571	-0.0052514	0.63851352	0.76385438	0.76385438	Lgalsl	1
GO:0043235	receptor complex	1/48	308/14612	0.00324675	0.9883658	-0.0118469	0.64093528	0.76385438	0.76385438	Mertk	1
GO:0030141	secretory granule	1/48	318/14612	0.00314465	0.95728512	-0.0442116	0.65280778	0.76749023	0.76749023	Fstl3	1
GO:0005938	cell cortex	1/48	328/14612	0.00304878	0.92809959	-0.0756073	0.66429563	0.76812849	0.76812849	Trip10	1
GO:0005774	vacuolar membrane	1/48	334/14612	0.00299401	0.91142715	-0.0940071	0.6710088	0.76812849	0.76812849	Mtmr2	1
GO:1902495	transmembrane transporter complex	1/48	388/14612	0.00257732	0.78457904	-0.2468955	0.72580261	0.7963545	0.7963545	Kcnk4	1
GO:0030312	external encapsulating structure	1/48	398/14612	0.00251256	0.764866	-0.2730355	0.73491921	0.7963545	0.7963545	Lgalsl	1
GO:0031012	extracellular matrix	1/48	398/14612	0.00251256	0.764866	-0.2730355	0.73491921	0.7963545	0.7963545	Lgalsl	1
GO:0005769	early endosome	1/48	399/14612	0.00250627	0.76294904	-0.2756167	0.73581437	0.7963545	0.7963545	Mtmr2	1
GO:1990351	transporter complex	1/48	412/14612	0.00242718	0.7388754	-0.3086544	0.7471856	0.7963545	0.7963545	Kcnk4	1
GO:0005874	microtubule	1/48	416/14612	0.00240385	0.73177083	-0.3186324	0.750587	0.7963545	0.7963545	Trip10	1
GO:0031252	cell leading edge	1/48	454/14612	0.00220264	0.67052129	-0.4094252	0.78074461	0.80928103	0.80928103	Fam107a	1
GO:0010008	endosome membrane	1/48	456/14612	0.00219298	0.66758041	-0.4140178	0.78222882	0.80928103	0.80928103	Mtmr2	1
GO:0000323	lytic vacuole	1/48	487/14612	0.00205339	0.62508556	-0.483084	0.80401466	0.80928103	0.80928103	Trip10	1
GO:0005764	lysosome	1/48	487/14612	0.00205339	0.62508556	-0.483084	0.80401466	0.80928103	0.80928103	Trip10	1
GO:0015629	actin cytoskeleton	1/48	495/14612	0.0020202	0.61498316	-0.500301	0.80928103	0.80928103	0.80928103	Fam107a	1

ID	Description	GeneRatio	BgRatio	RichFactor	FoldEnrichm	zScore	pvalue	p.adjust	qvalue	geneID	Count
GO:0099738	cell cortex region	2/40	40/14612	0.05	18.265	5.72858527	0.00533676	0.2946151	0.26916176	Ctbp2/Tchp	2
GO:0099568	cytoplasmic region	4/40	270/14612	0.01481481	5.41185185	3.83361352	0.00617917	0.2946151	0.26916176	Cys1/Cntf/Ctbp2/Tchp	4
GO:0060170	ciliary membrane	2/40	54/14612	0.03703704	13.5296296	4.83275346	0.00955762	0.2946151	0.26916176	Cys1/Evc2	2
GO:0005741	mitochondrial outer membrane	3/40	187/14612	0.01604278	5.86042781	3.50467519	0.01440068	0.2946151	0.26916176	Bok/Capn10/Rhot1	3
GO:0019867	outer membrane	3/40	214/14612	0.01401869	5.12102804	3.18179178	0.02056102	0.2946151	0.26916176	Bok/Capn10/Rhot1	3
GO:0031968	organelle outer membrane	3/40	214/14612	0.01401869	5.12102804	3.18179178	0.02056102	0.2946151	0.26916176	Bok/Capn10/Rhot1	3
GO:0031904	endosome lumen	1/40	11/14612	0.09090909	33.2090909	5.59878634	0.02971348	0.2946151	0.26916176	Ptpn1	1
GO:0005677	chromatin silencing complex	1/40	13/14612	0.07692308	28.1	5.12141298	0.03502265	0.2946151	0.26916176	Rrp8	1
GO:0030061	mitochondrial crista	1/40	14/14612	0.07142857	26.0928571	4.9212773	0.0376666	0.2946151	0.26916176	Ptpn1	1
GO:0005852	eukaryotic translation initiation factor 3 complex	1/40	15/14612	0.06666667	24.3533333	4.7410342	0.04030349	0.2946151	0.26916176	Eif3j1	1
GO:0033106	cis-Golgi network membrane	1/40	16/14612	0.0625	22.83125	4.57753899	0.04293334	0.2946151	0.26916176	Bok	1
GO:0098684	photoreceptor ribbon synapse	1/40	16/14612	0.0625	22.83125	4.57753899	0.04293334	0.2946151	0.26916176	Ctbp2	1
GO:0030057	desmosome	1/40	18/14612	0.05555556	20.2944444	4.29132834	0.04817197	0.2946151	0.26916176	Tchp	1
GO:0033290	eukaryotic 48S preinitiation complex	1/40	18/14612	0.05555556	20.2944444	4.29132834	0.04817197	0.2946151	0.26916176	Eif3j1	1
GO:0016282	eukaryotic 43S preinitiation complex	1/40	19/14612	0.05263158	19.2263158	4.16498829	0.05078079	0.2946151	0.26916176	Eif3j1	1
GO:0031082	BLOC complex	1/40	20/14612	0.05	18.265	4.04794455	0.05338264	0.2946151	0.26916176	Hps5	1
GO:0070993	translation preinitiation complex	1/40	20/14612	0.05	18.265	4.04794455	0.05338264	0.2946151	0.26916176	Eif3j1	1
GO:0098554	cytoplasmic side of endoplasmic reticulum membrane	1/40	20/14612	0.05	18.265	4.04794455	0.05338264	0.2946151	0.26916176	Ptpn1	1
GO:0097228	sperm principal piece	1/40	21/14612	0.04761905	17.3952381	3.9390839	0.05597753	0.2946151	0.26916176	Catsperz	1
GO:0031462	Cul2-RING ubiquitin ligase complex	1/40	22/14612	0.04545455	16.6045455	3.83747184	0.05856549	0.2946151	0.26916176	Lrrc75a	1
GO:0005938	cell cortex	3/40	328/14612	0.00914634	3.34115854	2.24674203	0.0601907	0.2946151	0.26916176	Iqgap2/Ctbp2/Tchp	3
GO:0098831	presynaptic active zone cytoplasmic component	1/40	23/14612	0.04347826	15.8826087	3.74231727	0.06114653	0.2946151	0.26916176	Ctbp2	1
GO:0030131	clathrin adaptor complex	1/40	26/14612	0.03846154	14.05	3.48930928	0.06884833	0.30871731	0.2820456	Lrrn3	1
GO:0016342	catenin complex	1/40	27/14612	0.03703704	13.5296296	3.41410822	0.07140187	0.30871731	0.2820456	Cdh19	1
GO:0000922	spindle pole	2/40	167/14612	0.01197605	4.3748503	2.29807586	0.07637094	0.30871731	0.2820456	Plk5/Fam83d	2
GO:0005640	nuclear outer membrane	1/40	29/14612	0.03448276	12.5965517	3.27502648	0.07648851	0.30871731	0.2820456	Bok	1
GO:0097470	ribbon synapse	1/40	30/14612	0.03333333	12.1766667	3.21051545	0.07902162	0.30871731	0.2820456	Ctbp2	1
GO:0005891	voltage-gated calcium channel complex	1/40	31/14612	0.03225806	11.783871	3.14899709	0.08154797	0.30871731	0.2820456	Catsperz	1
GO:0031461	cullin-RING ubiquitin ligase complex	2/40	190/14612	0.01052632	3.84526316	2.0682207	0.09517496	0.33646876	0.30739946	Lrrc75a/Fbh1	2
GO:0005769	early endosome	3/40	399/14612	0.0075188	2.74661654	1.85332474	0.09522701	0.33646876	0.30739946	Bok/Hps5/Ptpn1	3
GO:0097431	mitotic spindle pole	1/40	39/14612	0.02564103	9.36666667	2.74107345	0.10151693	0.34257945	0.31298222	Fam83d	1
GO:0005637	nuclear inner membrane	1/40	41/14612	0.02439024	8.9097561	2.65717848	0.10644264	0.34257945	0.31298222	Smad1	1
GO:0030119	AP-type membrane coat adaptor complex	1/40	43/14612	0.02325581	8.49534884	2.57882333	0.11134202	0.34257945	0.31298222	Lrrn3	1
GO:0097386	glial cell projection	1/40	43/14612	0.02325581	8.49534884	2.57882333	0.11134202	0.34257945	0.31298222	Cntf	1
GO:0030118	clathrin coat	1/40	48/14612	0.02083333	7.61041667	2.40336373	0.12347618	0.34257945	0.31298222	Lrrn3	1
GO:0008023	transcription elongation factor complex	1/40	49/14612	0.02040816	7.45510204	2.37129787	0.12588355	0.34257945	0.31298222	Aff1	1
GO:0099523	presynaptic cytosol	1/40	49/14612	0.02040816	7.45510204	2.37129787	0.12588355	0.34257945	0.31298222	Ctbp2	1
GO:0034704	calcium channel complex	1/40	50/14612	0.02	7.306	2.34012383	0.12828447	0.34257945	0.31298222	Catsperz	1
GO:0032588	trans-Golgi network membrane	1/40	51/14612	0.01960784	7.1627451	2.3097984	0.13067897	0.34257945	0.31298222	Bok	1
GO:0032838	plasma membrane bounded cell projection cytoplasm	2/40	232/14612	0.00862069	3.14913793	1.72877799	0.13241299	0.34257945	0.31298222	Cys1/Cntf	2
GO:0098797	plasma membrane protein complex	3/40	463/14612	0.00647948	2.36695464	1.56600128	0.13250715	0.34257945	0.31298222	Catsperz/Cdh19/Evc2	3
GO:0005801	cis-Golgi network	1/40	58/14612	0.01724138	6.29827586	2.1182039	0.14726178	0.3687948	0.33693269	Bok	1
GO:0019005	SCF ubiquitin ligase complex	1/40	59/14612	0.01694915	6.19152542	2.09341394	0.14960544	0.3687948	0.33693269	Fbh1	1
GO:0005882	intermediate filament	1/40	61/14612	0.01639344	5.98852459	2.04550714	0.15427392	0.37165989	0.33955025	Tchp	1
GO:0000502	proteasome complex	1/40	64/14612	0.015625	5.7078125	1.97750633	0.16122982	0.3797858	0.34697412	Zfand2b	1
GO:0099522	cytosolic region	1/40	71/14612	0.01408451	5.14507042	1.83431676	0.17724404	0.40636793	0.37125967	Ctbp2	1
GO:0031965	nuclear membrane	2/40	283/14612	0.00706714	2.58162544	1.40766497	0.18121965	0.40636793	0.37125967	Bok/Smad1	2
GO:0017053	transcription repressor complex	1/40	74/14612	0.01351351	4.93648649	1.77861798	0.18401566	0.40636793	0.37125967	Ctbp2	1
GO:0045171	intercellular bridge	1/40	82/14612	0.01219512	4.45487805	1.64367983	0.20180875	0.42407425	0.38743626	Fam83d	1
GO:0000151	ubiquitin ligase complex	2/40	306/14612	0.00653595	2.3875817	1.28519951	0.20407266	0.42407425	0.38743626	Lrrc75a/Fbh1	2
GO:0005902	microvillus	1/40	87/14612	0.01149425	4.19885057	1.567855	0.21273673	0.42407425	0.38743626	Iqgap2	1
GO:0000792	heterochromatin	1/40	90/14612	0.01111111	4.05888889	1.52504313	0.21922335	0.42407425	0.38743626	Rrp8	1
GO:0031253	cell projection membrane	2/40	324/14612	0.00617284	2.25493827	1.19679436	0.22219157	0.42407425	0.38743626	Cys1/Evc2	2
GO:0055038	recycling endosome membrane	1/40	92/14612	0.01086957	3.97065217	1.49752056	0.22351878	0.42407425	0.38743626	Bok	1
GO:1905369	endopeptidase complex	1/40	92/14612	0.01086957	3.97065217	1.49752056	0.22351878	0.42407425	0.38743626	Zfand2b	1
GO:0045111	intermediate filament cytoskeleton	1/40	94/14612	0.0106383	3.88617021	1.47076354	0.22779117	0.42407425	0.38743626	Tchp	1
GO:0030117	membrane coat	1/40	96/14612	0.01041667	3.80520833	1.44473316	0.23204063	0.42407425	0.38743626	Lrrn3	1
GO:0048475	coated membrane	1/40	96/14612	0.01041667	3.80520833	1.44473316	0.23204063	0.42407425	0.38743626	Lrrn3	1
GO:0005604	basement membrane	1/40	98/14612	0.01020408	3.72755102	1.41939328	0.23626729	0.42448022	0.38780715	Matn2	1
GO:0005759	mitochondrial matrix	2/40	351/14612	0.00569801	2.08148148	1.07450401	0.24961657	0.44098927	0.4028899	Capn10/Ptpn1	2
GO:0030175	filopodium	1/40	109/14612	0.00917431	3.35137615	1.29097199	0.25911162	0.44966265	0.41081394	Iqgap2	1
GO:0005776	autophagosome	1/40	111/14612	0.00900901	3.29099099	1.26939353	0.26319294	0.44966265	0.41081394	Rab24	1
GO:0019897	extrinsic component of plasma membrane	1/40	113/14612	0.00884956	3.23274336	1.2483012	0.26725233	0.44966265	0.41081394	Cdh19	1
GO:1905368	peptidase complex	1/40	122/14612	0.00819672	2.9942623	1.15887978	0.28525137	0.46639439	0.42610014	Zfand2b	1
GO:0005819	spindle	2/40	394/14612	0.00507614	1.85431472	0.90065223	0.29351665	0.46639439	0.42610014	Plk5/Fam83d	2
GO:0030312	external encapsulating structure	2/40	398/14612	0.00502513	1.83567839	0.88559048	0.29759619	0.46639439	0.42610014	Matn2/Lrrn3	2
GO:0031012	extracellular matrix	2/40	398/14612	0.00502513	1.83567839	0.88559048	0.29759619	0.46639439	0.42610014	Matn2/Lrrn3	2
GO:0005930	axoneme	1/40	134/14612	0.00746269	2.7261194	1.05166963	0.30858087	0.46639439	0.42610014	Cys1	1
GO:0036126	sperm flagellum	1/40	135/14612	0.00740741	2.70592593	1.04327344	0.31049113	0.46639439	0.42610014	Catsperz	1
GO:0097014	ciliary plasm	1/40	135/14612	0.00740741	2.70592593	1.04327344	0.31049113	0.46639439	0.42610014	Cys1	1
GO:0048786	presynaptic active zone	1/40	136/14612	0.00735294	2.68602941	1.03495317	0.31239624	0.46639439	0.42610014	Ctbp2	1
GO:0097729	9+2 motile cilium	1/40	152/14612	0.00657895	2.40328947	0.91116137	0.34218914	0.48524319	0.44332049	Catsperz	1
GO:0000776	kinetochore	1/40	158/14612	0.00632911	2.31202532	0.8687346	0.3530338	0.48524319	0.44332049	Plk5	1
GO:0098982	GABA-ergic synapse	1/40	158/14612	0.00632911	2.31202532	0.8687346	0.3530338	0.48524319	0.44332049	Ctbp2	1
GO:0005635	nuclear envelope	2/40	458/14612	0.00436681	1.59519651	0.67805341	0.35825486	0.48524319	0.44332049	Bok/Smad1	2
GO:0005667	transcription regulator complex	2/40	458/14612	0.00436681	1.59519651	0.67805341	0.35825486	0.48524319	0.44332049	Ctbp2/Smad1	2
GO:0005912	adherens junction	1/40	161/14612	0.00621118	2.2689441	0.84823632	0.35839056	0.48524319	0.44332049	Cdh19	1
GO:0005911	cell-cell junction	2/40	467/14612	0.00428266	1.56445396	0.649525	0.36722503	0.48524319	0.44332049	Cdh19/Tchp	2
GO:0000779	condensed chromosome, centromeric region	1/40	168/14612	0.00595238	2.17440476	0.80211953	0.37072205	0.48524319	0.44332049	Plk5	1
GO:0036064	ciliary basal body	1/40	170/14612	0.00588235	2.14882353	0.78935888	0.37420268	0.48524319	0.44332049	Cys1	1
GO:0072686	mitotic spindle	1/40	170/14612	0.00588235	2.14882353	0.78935888	0.37420268	0.48524319	0.44332049	Fam83d	1
GO:0019898	extrinsic component of membrane	1/40	174/14612	0.00574713	2.09942529	0.7643587	0.38110773	0.48524319	0.44332049	Cdh19	1
GO:0031901	early endosome membrane	1/40	174/14612	0.00574713	2.09942529	0.7643587	0.38110773	0.48524319	0.44332049	Bok	1
GO:0034703	cation channel complex	1/40	176/14612	0.00568182	2.07556818	0.75210977	0.38453234	0.48524319	0.44332049	Catsperz	1
GO:0055037	recycling endosome	1/40	187/14612	0.00534759	1.95347594	0.68751632	0.40303997	0.50261456	0.45919105	Bok	1
GO:0098562	cytoplasmic side of membrane	1/40	190/14612	0.00526316	1.92263158	0.67066054	0.4079926	0.50287461	0.45942864	Ptpn1	1
GO:0031514	motile cilium	1/40	204/14612	0.00490196	1.79068627	0.59583748	0.43058014	0.52461488	0.47929066	Catsperz	1
GO:0030027	lamellipodium	1/40	212/14612	0.00471698	1.72311321	0.55565262	0.4431077	0.53374336	0.48763048	Iqgap2	1
GO:0098858	actin-based cell projection	1/40	216/14612	0.00462963	1.6912037	0.53619455	0.44927029	0.53508597	0.48885709	Iqgap2	1
GO:0000775	chromosome, centromeric region	1/40	225/14612	0.00444444	1.62355556	0.49384615	0.46289403	0.54266944	0.49578539	Plk5	1
GO:0090575	RNA polymerase II transcription regulator complex	1/40	227/14612	0.00440529	1.6092511	0.48469072	0.4658766	0.54266944	0.49578539	Smad1	1
GO:0016323	basolateral plasma membrane	1/40	235/14612	0.00425532	1.55446809	0.44893838	0.47764622	0.55033152	0.5027855	Slc43a3	1
GO:0005802	trans-Golgi network	1/40	243/14612	0.00411523	1.50329218	0.41449608	0.48916284	0.55754045	0.50937161	Bok	1
GO:0000793	condensed chromosome	1/40	248/14612	0.00403226	1.47298387	0.39359073	0.49623461	0.55958371	0.51123835	Plk5	1
GO:0009925	basal plasma membrane	1/40	258/14612	0.00387597	1.41589147	0.35311306	0.51009296	0.56915635	0.51998396	Slc43a3	1
GO:0034702	monoatomic ion channel complex	1/40	271/14612	0.00369004	1.34797048	0.30293379	0.52755419	0.58223911	0.53193643	Catsperz	1
GO:0045178	basal part of cell	1/40	275/14612	0.00363636	1.32836364	0.28800674	0.53280371	0.58223911	0.53193643	Slc43a3	1
GO:0062023	collagen-containing extracellular matrix	1/40	306/14612	0.00326797	1.19379085	0.17949207	0.5716039	0.61826545	0.56485026	Matn2	1
GO:0098687	chromosomal region	1/40	321/14612	0.00311526	1.13800623	0.13098763	0.58923205	0.63089493	0.57638861	Plk5	1
GO:0098791	Golgi apparatus subcompartment	1/40	347/14612	0.00288184	1.05273775	0.05209078	0.61812649	0.65521408	0.5986067	Bok	1
GO:1902495	transmembrane transporter complex	1/40	388/14612	0.00257732	0.94149485	-0.0611941	0.65970963	0.69052577	0.63086763	Catsperz	1
GO:0045177	apical part of cell	1/40	393/14612	0.00254453	0.92951654	-0.0742095	0.66446819	0.69052577	0.63086763	Tchp	1
GO:1990351	transporter complex	1/40	412/14612	0.00242718	0.88665049	-0.1222741	0.68196613	0.69871824	0.63835231	Catsperz	1
GO:0005874	microtubule	1/40	416/14612	0.00240385	0.878125	-0.1321261	0.68553488	0.69871824	0.63835231	Iqgap2	1
GO:0031252	cell leading edge	1/40	454/14612	0.00220264	0.80462555	-0.221567	0.71754777	0.71914157	0.65701117	Iqgap2	1
GO:0010008	endosome membrane	1/40	456/14612	0.00219298	0.80109649	-0.2260815	0.71914157	0.71914157	0.65701117	Bok	1

ID	Description	GeneRatio	BgRatio	RichFactor	FoldEnrichm	zScore	pvalue	p.adjust	qvalue	geneID	Count
GO:0140534	endoplasmic reticulum protein-containing complex	6/98	119/14612	0.05042017	7.51774996	5.86612724	0.00014701	0.03043037	0.0300203	P4ha1/Pdia6/Mlec/Traf2/Caml/Pigc	6
GO:0001650	fibrillar center	6/98	144/14612	0.04166667	6.21258503	5.1652292	0.00041276	0.04272032	0.04214463	Lipa/Ubtf/Pafah1b2/Sap30l/Nhej1/Taf4b	6
GO:0005788	endoplasmic reticulum lumen	4/98	105/14612	0.03809524	5.68007775	3.95474386	0.00542384	0.37424488	0.36920166	P4ha1/Pdia6/Pcsk9/Uggt1	4
GO:1990391	DNA repair complex	2/98	22/14612	0.09090909	13.554731	4.84228732	0.00942475	0.48773092	0.48115839	Lig4/Nhej1	2
GO:0005879	axonemal microtubule	2/98	32/14612	0.0625	9.31887755	3.87097537	0.01937805	0.58253279	0.57468275	Efhc1/Tuba1a	2
GO:0016235	aggresome	2/98	33/14612	0.06060606	9.03648732	3.79768391	0.02053926	0.58253279	0.57468275	Hspa1b/Edem1	2
GO:0101031	protein folding chaperone complex	2/98	35/14612	0.05714286	8.52011662	3.66002431	0.02294647	0.58253279	0.57468275	Ptges3/Cct7	2
GO:0005881	cytoplasmic microtubule	3/98	94/14612	0.03191489	4.75857577	3.00395408	0.02527799	0.58253279	0.57468275	Efhc1/Tuba1a/Togaram2	3
GO:0042470	melanosome	3/98	98/14612	0.03061224	4.56434819	2.90910267	0.02814168	0.58253279	0.57468275	Pdia6/Trpv2/Atp1b3	3
GO:0048770	pigment granule	3/98	98/14612	0.03061224	4.56434819	2.90910267	0.02814168	0.58253279	0.57468275	Pdia6/Trpv2/Atp1b3	3
GO:0045335	phagocytic vesicle	3/98	117/14612	0.02564103	3.82312925	2.51926604	0.04398664	0.58482147	0.57694058	Rab39/Becn1/Cd82	3
GO:0031514	motile cilium	4/98	204/14612	0.01960784	2.92356943	2.27342457	0.04836244	0.58482147	0.57694058	Efhc1/Atp1b3/Tuba1a/Dnai4	4
GO:0090734	site of DNA damage	3/98	122/14612	0.02459016	3.66644363	2.43017516	0.04875919	0.58482147	0.57694058	Spindoc/Nhej1/Eloa	3
GO:0045121	membrane raft	5/98	299/14612	0.01672241	2.49334516	2.14382784	0.05066362	0.58482147	0.57694058	Hspa1b/Traf2/Atp1b3/Tuba1a/Ctnnd1	5
GO:0098857	membrane microdomain	5/98	301/14612	0.0166113	2.47677809	2.12727162	0.05185883	0.58482147	0.57694058	Hspa1b/Traf2/Atp1b3/Tuba1a/Ctnnd1	5
GO:0019867	outer membrane	4/98	214/14612	0.01869159	2.78695403	2.16385738	0.0558094	0.58482147	0.57694058	Smpd4/Becn1/Nme1/Mief1	4
GO:0031968	organelle outer membrane	4/98	214/14612	0.01869159	2.78695403	2.16385738	0.0558094	0.58482147	0.57694058	Smpd4/Becn1/Nme1/Mief1	4
GO:0005930	axoneme	3/98	134/14612	0.02238806	3.33810539	2.23419793	0.06119745	0.58482147	0.57694058	Efhc1/Tuba1a/Dnai4	3
GO:0036126	sperm flagellum	3/98	135/14612	0.02222222	3.31337868	2.2188798	0.06229509	0.58482147	0.57694058	Efhc1/Atp1b3/Tuba1a	3
GO:0097014	ciliary plasm	3/98	135/14612	0.02222222	3.31337868	2.2188798	0.06229509	0.58482147	0.57694058	Efhc1/Tuba1a/Dnai4	3
GO:0035631	CD40 receptor complex	1/98	10/14612	0.1	14.9102041	3.61565329	0.06509923	0.58482147	0.57694058	Traf2	1
GO:0034663	endoplasmic reticulum chaperone complex	1/98	11/14612	0.09090909	13.554731	3.42272417	0.07137373	0.58482147	0.57694058	Pdia6	1
GO:0016323	basolateral plasma membrane	4/98	235/14612	0.01702128	2.53790708	1.95294197	0.07334287	0.58482147	0.57694058	Hspa1b/Abcc5/Atp1b3/Slc16a5	4
GO:0000800	lateral element	1/98	12/14612	0.08333333	12.4251701	3.25339142	0.07760655	0.58482147	0.57694058	Smc1a	1
GO:0097729	9+2 motile cilium	3/98	152/14612	0.01973684	2.94280344	1.97845401	0.08232172	0.58482147	0.57694058	Efhc1/Atp1b3/Tuba1a	3
GO:0044322	endoplasmic reticulum quality control compartment	1/98	13/14612	0.07692308	11.4693878	3.10306488	0.08379796	0.58482147	0.57694058	Edem1	1
GO:0000793	condensed chromosome	4/98	248/14612	0.01612903	2.40487163	1.83351401	0.08544722	0.58482147	0.57694058	Tuba1a/Lig4/Dctn5/Smc1a	4
GO:0016234	inclusion body	2/98	73/14612	0.02739726	4.08498742	2.17123588	0.08615813	0.58482147	0.57694058	Hspa1b/Edem1	2
GO:0008250	oligosaccharyltransferase complex	1/98	14/14612	0.07142857	10.6501458	2.96831961	0.08994823	0.58482147	0.57694058	Mlec	1
GO:0160111	axonemal A tubule inner sheath	1/98	14/14612	0.07142857	10.6501458	2.96831961	0.08994823	0.58482147	0.57694058	Efhc1	1
GO:0009925	basal plasma membrane	4/98	258/14612	0.01550388	2.31165955	1.74664223	0.09538312	0.58482147	0.57694058	Hspa1b/Abcc5/Atp1b3/Slc16a5	4
GO:0005614	interstitial matrix	1/98	15/14612	0.06666667	9.94013605	2.84654073	0.09605763	0.58482147	0.57694058	Vwa1	1
GO:0005858	axonemal dynein complex	1/98	15/14612	0.06666667	9.94013605	2.84654073	0.09605763	0.58482147	0.57694058	Dnai4	1
GO:0005942	phosphatidylinositol 3-kinase complex	1/98	15/14612	0.06666667	9.94013605	2.84654073	0.09605763	0.58482147	0.57694058	Becn1	1
GO:0120293	dynein axonemal particle	1/98	16/14612	0.0625	9.31887755	2.73569228	0.10212644	0.58915965	0.5812203	Dnai4	1
GO:0072686	mitotic spindle	3/98	170/14612	0.01764706	2.63121248	1.7578458	0.10614032	0.58915965	0.5812203	Efhc1/Tbck/Smc1a	3
GO:0099568	cytoplasmic region	4/98	270/14612	0.01481481	2.20891912	1.64753147	0.10799431	0.58915965	0.5812203	Efhc1/Tuba1a/Ctnnd1/Dnai4	4
GO:0016327	apicolateral plasma membrane	1/98	17/14612	0.05882353	8.77070828	2.63416191	0.10815491	0.58915965	0.5812203	Ctnnd1	1
GO:0045178	basal part of cell	4/98	275/14612	0.01454545	2.16875696	1.60775868	0.11346242	0.60222362	0.59410822	Hspa1b/Abcc5/Atp1b3/Slc16a5	4
GO:0005697	telomerase holoenzyme complex	1/98	20/14612	0.05	7.45510204	2.37366895	0.12600099	0.61881588	0.61047689	Ptges3	1
GO:0005741	mitochondrial outer membrane	3/98	187/14612	0.01604278	2.39201135	1.57421888	0.13080796	0.61881588	0.61047689	Becn1/Nme1/Mief1	3
GO:0070822	Sin3-type complex	1/98	21/14612	0.04761905	7.10009718	2.29859957	0.13187078	0.61881588	0.61047689	Sap30l	1
GO:0030496	midbody	3/98	190/14612	0.01578947	2.35424275	1.54390304	0.13535712	0.61881588	0.61047689	Ankrd54/Ctnnd1/Tbck	3
GO:0005901	caveola	2/98	98/14612	0.02040816	3.04289879	1.66734618	0.14029656	0.61881588	0.61047689	Atp1b3/Ctnnd1	2
GO:0005793	endoplasmic reticulum-Golgi intermediate compartment	2/98	99/14612	0.02020202	3.01216244	1.65067467	0.14259374	0.61881588	0.61047689	Pdia6/Uggt1	2
GO:0097649	A axonemal microtubule	1/98	23/14612	0.04347826	6.48269743	2.16224491	0.14349354	0.61881588	0.61047689	Efhc1	1
GO:0098831	presynaptic active zone cytoplasmic component	1/98	23/14612	0.04347826	6.48269743	2.16224491	0.14349354	0.61881588	0.61047689	Ctnnd1	1
GO:0160110	axonemal microtubule doublet inner sheath	1/98	23/14612	0.04347826	6.48269743	2.16224491	0.14349354	0.61881588	0.61047689	Efhc1	1
GO:0005796	Golgi lumen	1/98	25/14612	0.04	5.96408163	2.04119914	0.15496226	0.62740326	0.61894855	Abcc5	1
GO:0097545	axonemal doublet microtubule	1/98	25/14612	0.04	5.96408163	2.04119914	0.15496226	0.62740326	0.61894855	Efhc1	1
GO:0030139	endocytic vesicle	3/98	203/14612	0.01477833	2.20347844	1.41882509	0.15567897	0.62740326	0.61894855	Rab39/Becn1/Cd82	3
GO:0000795	synaptonemal complex	1/98	26/14612	0.03846154	5.73469388	1.98550014	0.16063948	0.62740326	0.61894855	Smc1a	1
GO:0099086	synaptonemal structure	1/98	26/14612	0.03846154	5.73469388	1.98550014	0.16063948	0.62740326	0.61894855	Smc1a	1
GO:0016342	catenin complex	1/98	27/14612	0.03703704	5.52229781	1.93262357	0.16627895	0.63740266	0.6288132	Ctnnd1	1
GO:0005776	autophagosome	2/98	111/14612	0.01801802	2.68652326	1.46559609	0.17073291	0.64257659	0.63391741	Becn1/Pip4k2c	2
GO:0005640	nuclear outer membrane	1/98	29/14612	0.03448276	5.14144968	1.83437194	0.17744562	0.65209772	0.64331024	Smpd4	1
GO:0005669	transcription factor TFIID complex	1/98	31/14612	0.03225806	4.80974325	1.74478673	0.18846424	0.65209772	0.64331024	Taf4b	1
GO:0005775	vacuolar lumen	1/98	31/14612	0.03225806	4.80974325	1.74478673	0.18846424	0.65209772	0.64331024	Cln5	1
GO:0000314	organellar small ribosomal subunit	1/98	32/14612	0.03125	4.65943878	1.70282556	0.19391863	0.65209772	0.64331024	Mrps27	1
GO:0005763	mitochondrial small ribosomal subunit	1/98	32/14612	0.03125	4.65943878	1.70282556	0.19391863	0.65209772	0.64331024	Mrps27	1
GO:0005875	microtubule associated complex	2/98	121/14612	0.01652893	2.46449654	1.32920298	0.19482938	0.65209772	0.64331024	Dnai4/Dctn5	2
GO:0098797	plasma membrane protein complex	5/98	463/14612	0.01079914	1.61017323	1.09632728	0.19963862	0.65209772	0.64331024	Shc1/Traf2/Atp1b3/Ctnnd1/Aph1b	5
GO:0032838	plasma membrane bounded cell projection cytoplasm	3/98	232/14612	0.01293103	1.92804363	1.17082609	0.2040142	0.65209772	0.64331024	Efhc1/Tuba1a/Dnai4	3
GO:0005790	smooth endoplasmic reticulum	1/98	34/14612	0.02941176	4.38535414	1.62388041	0.20471879	0.65209772	0.64331024	Pdia6	1
GO:0005840	ribosome	3/98	240/14612	0.0125	1.86377551	1.10868291	0.21792828	0.65209772	0.64331024	Nufip2/Mrps27/Hbs1l	3
GO:0044853	plasma membrane raft	2/98	131/14612	0.01526718	2.27636704	1.20578923	0.2193293	0.65209772	0.64331024	Atp1b3/Ctnnd1	2
GO:0000407	phagophore assembly site	1/98	37/14612	0.02702703	4.02978489	1.51623956	0.22065103	0.65209772	0.64331024	Becn1	1
GO:0090533	cation-transporting ATPase complex	1/98	37/14612	0.02702703	4.02978489	1.51623956	0.22065103	0.65209772	0.64331024	Atp1b3	1
GO:0098533	ATPase dependent transmembrane transport complex	1/98	37/14612	0.02702703	4.02978489	1.51623956	0.22065103	0.65209772	0.64331024	Atp1b3	1
GO:0000323	lytic vacuole	5/98	487/14612	0.01026694	1.53082177	0.97899039	0.22851371	0.65209772	0.64331024	Rab39/Pcsk9/Lipa/Sphk2/Cln5	5
GO:0005764	lysosome	5/98	487/14612	0.01026694	1.53082177	0.97899039	0.22851371	0.65209772	0.64331024	Rab39/Pcsk9/Lipa/Sphk2/Cln5	5
GO:0033017	sarcoplasmic reticulum membrane	1/98	39/14612	0.02564103	3.82312925	1.4506012	0.2310966	0.65209772	0.64331024	Fkbp1b	1
GO:0097431	mitotic spindle pole	1/98	39/14612	0.02564103	3.82312925	1.4506012	0.2310966	0.65209772	0.64331024	Smc1a	1
GO:0061695	transferase complex, transferring phosphorus-containing groups	3/98	250/14612	0.012	1.78922449	1.03424289	0.23559136	0.65209772	0.64331024	Cdkn1a/Becn1/Taf4b	3
GO:0099738	cell cortex region	1/98	40/14612	0.025	3.72755102	1.41939328	0.23626729	0.65209772	0.64331024	Ctnnd1	1
GO:0030286	dynein complex	1/98	44/14612	0.02272727	3.38868275	1.30390002	0.25660814	0.69891953	0.68950109	Dnai4	1
GO:0044232	organelle membrane contact site	1/98	47/14612	0.0212766	3.17238385	1.22571556	0.27151101	0.72054845	0.71083854	Stard3	1
GO:0044295	axonal growth cone	1/98	47/14612	0.0212766	3.17238385	1.22571556	0.27151101	0.72054845	0.71083854	Trpv2	1
GO:0099092	postsynaptic density, intracellular component	1/98	48/14612	0.02083333	3.10629252	1.20104262	0.27641262	0.7228793	0.71313798	Ctnnd1	1
GO:0008023	transcription elongation factor complex	1/98	49/14612	0.02040816	3.04289879	1.17700665	0.28128158	0.7228793	0.71313798	Eloa	1
GO:0034704	calcium channel complex	1/98	50/14612	0.02	2.98204082	1.15357685	0.28611811	0.7228793	0.71313798	Fkbp1b	1
GO:0000776	kinetochore	2/98	158/14612	0.01265823	1.88736761	0.92150466	0.28635798	0.7228793	0.71313798	Dctn5/Smc1a	2
GO:0000307	cyclin-dependent protein kinase holoenzyme complex	1/98	51/14612	0.01960784	2.92356943	1.1307245	0.29092243	0.72555352	0.71577617	Cdkn1a	1
GO:0030670	phagocytic vesicle membrane	1/98	52/14612	0.01923077	2.86734694	1.10842279	0.29569474	0.72867631	0.71885688	Rab39	1
GO:0005874	microtubule	4/98	416/14612	0.00961538	1.43367347	0.73737086	0.30496889	0.7314034	0.72154722	Efhc1/Tuba1a/Togaram2/Cct7	4
GO:0000922	spindle pole	2/98	167/14612	0.01197605	1.78565318	0.83905376	0.30868344	0.7314034	0.72154722	Efhc1/Smc1a	2
GO:0000786	nucleosome	1/98	55/14612	0.01818182	2.7109462	1.04457879	0.30982174	0.7314034	0.72154722	Sphk2	1
GO:0000779	condensed chromosome, centromeric region	2/98	168/14612	0.01190476	1.7750243	0.83020562	0.31115709	0.7314034	0.72154722	Dctn5/Smc1a	2
GO:0099091	postsynaptic specialization, intracellular component	1/98	56/14612	0.01785714	2.66253644	1.02424443	0.31446813	0.7314034	0.72154722	Ctnnd1	1
GO:0030134	COPII-coated ER to Golgi transport vesicle	1/98	57/14612	0.01754386	2.61582528	1.0043502	0.31908355	0.73389217	0.72400245	Pcsk9	1
GO:0019898	extrinsic component of membrane	2/98	174/14612	0.01149425	1.71381656	0.7783361	0.32595967	0.7414687	0.73147688	Ctnnd1/Becn1	2
GO:0000794	condensed nuclear chromosome	1/98	60/14612	0.01666667	2.48503401	0.94713118	0.33274609	0.74867869	0.73858971	Smc1a	1
GO:0005882	intermediate filament	1/98	61/14612	0.01639344	2.44429575	0.92882539	0.33723969	0.75063027	0.74051499	Nme1	1
GO:0098686	hippocampal mossy fiber to CA3 synapse	1/98	62/14612	0.01612903	2.40487163	0.91087847	0.34170333	0.75100257	0.74088228	Ctnnd1	1
GO:0032580	Golgi cisterna membrane	1/98	63/14612	0.01587302	2.36669906	0.89327679	0.34613722	0.75100257	0.74088228	Fut11	1
GO:0016324	apical plasma membrane	3/98	319/14612	0.00940439	1.40221355	0.59682883	0.36153001	0.75100257	0.74088228	Hspa1b/Abcc5/Atp1b3	3
GO:0031252	cell leading edge	4/98	454/14612	0.00881057	1.31367437	0.55791168	0.36297669	0.75100257	0.74088228	Trpv2/Raph1/Ctnnd1/Nme1	4
GO:0098562	cytoplasmic side of membrane	2/98	190/14612	0.01052632	1.56949517	0.64925237	0.36499592	0.75100257	0.74088228	Traf2/Becn1	2
GO:0010008	endosome membrane	4/98	456/14612	0.00877193	1.30791264	0.54890743	0.36603936	0.75100257	0.74088228	Shc1/Abcc5/Stard3/Becn1	4
GO:0016591	RNA polymerase II, holoenzyme	1/98	68/14612	0.01470588	2.19267707	0.81001832	0.36786712	0.75100257	0.74088228	Taf4b	1
GO:0005938	cell cortex	3/98	328/14612	0.00914634	1.36373818	0.54747001	0.3780047	0.75100257	0.74088228	Traf2/Ctnnd1/Cap1	3
GO:0030666	endocytic vesicle membrane	1/98	73/14612	0.01369863	2.04249371	0.73371442	0.388882	0.75100257	0.74088228	Rab39	1
GO:0032040	small-subunit processome	1/98	75/14612	0.01333333	1.98802721	0.70489039	0.39709299	0.75100257	0.74088228	Rps19bp1	1
GO:0000228	nuclear chromosome	2/98	204/14612	0.00980392	1.46178471	0.54577324	0.39845449	0.75100257	0.74088228	Sap30l/Smc1a	2
GO:0005643	nuclear pore	1/98	77/14612	0.01298701	1.93639014	0.67694615	0.40519476	0.75100257	0.74088228	Bicd2	1
GO:0015935	small ribosomal subunit	1/98	77/14612	0.01298701	1.93639014	0.67694615	0.40519476	0.75100257	0.74088228	Mrps27	1
GO:0098791	Golgi apparatus subcompartment	3/98	347/14612	0.00864553	1.28906664	0.44780224	0.41248514	0.75100257	0.74088228	Smpd4/Becn1/Fut11	3
GO:0043209	myelin sheath	2/98	210/14612	0.00952381	1.42001944	0.50376394	0.41255158	0.75100257	0.74088228	Tuba1a/Nme1	2
GO:0016529	sarcoplasmic reticulum	1/98	80/14612	0.0125	1.86377551	0.63656482	0.41714579	0.75100257	0.74088228	Fkbp1b	1
GO:0030027	lamellipodium	2/98	212/14612	0.00943396	1.40662303	0.49004741	0.41721532	0.75100257	0.74088228	Trpv2/Ctnnd1	2
GO:0005759	mitochondrial matrix	3/98	351/14612	0.00854701	1.27437642	0.42754792	0.41967656	0.75100257	0.74088228	Hspa1b/Shc1/Mrps27	3
GO:0000118	histone deacetylase complex	1/98	81/14612	0.01234568	1.84076594	0.62348974	0.42107641	0.75100257	0.74088228	Sap30l	1
GO:0005758	mitochondrial intermembrane space	1/98	82/14612	0.01219512	1.81831757	0.61059809	0.42498079	0.75100257	0.74088228	Coa4	1
GO:0010494	cytoplasmic stress granule	1/98	85/14612	0.01176471	1.75414166	0.57297263	0.43653822	0.75100257	0.74088228	Nufip2	1
GO:0030864	cortical actin cytoskeleton	1/98	86/14612	0.01162791	1.73374466	0.56076462	0.44033936	0.75100257	0.74088228	Cap1	1
GO:0000775	chromosome, centromeric region	2/98	225/14612	0.00888889	1.32535147	0.40412746	0.44706983	0.75100257	0.74088228	Dctn5/Smc1a	2
GO:0000313	organellar ribosome	1/98	88/14612	0.01136364	1.69434137	0.5368225	0.44786568	0.75100257	0.74088228	Mrps27	1
GO:0005761	mitochondrial ribosome	1/98	88/14612	0.01136364	1.69434137	0.5368225	0.44786568	0.75100257	0.74088228	Mrps27	1
GO:0090575	RNA polymerase II transcription regulator complex	2/98	227/14612	0.00881057	1.31367437	0.39137806	0.45158875	0.75100257	0.74088228	Rfxap/Taf4b	2
GO:0036464	cytoplasmic ribonucleoprotein granule	2/98	229/14612	0.00873362	1.30220123	0.37874654	0.45608711	0.75100257	0.74088228	Nufip2/Tuba1a	2
GO:0055029	nuclear DNA-directed RNA polymerase complex	1/98	91/14612	0.01098901	1.63848397	0.50203255	0.45896769	0.75100257	0.74088228	Taf4b	1
GO:0030426	growth cone	2/98	232/14612	0.00862069	1.28536242	0.36001515	0.46279551	0.75100257	0.74088228	Trpv2/Ctnnd1	2
GO:0035861	site of double-strand break	1/98	93/14612	0.01075269	1.60324775	0.4795438	0.46624599	0.75100257	0.74088228	Nhej1	1
GO:0000428	DNA-directed RNA polymerase complex	1/98	94/14612	0.0106383	1.58619192	0.46850023	0.46984871	0.75100257	0.74088228	Taf4b	1
GO:0045111	intermediate filament cytoskeleton	1/98	94/14612	0.0106383	1.58619192	0.46850023	0.46984871	0.75100257	0.74088228	Nme1	1
GO:0030880	RNA polymerase complex	1/98	95/14612	0.01052632	1.56949517	0.45758613	0.47342736	0.75100257	0.74088228	Taf4b	1
GO:0031970	organelle envelope lumen	1/98	95/14612	0.01052632	1.56949517	0.45758613	0.47342736	0.75100257	0.74088228	Coa4	1
GO:0030427	site of polarized growth	2/98	238/14612	0.00840336	1.25295833	0.32330177	0.47606842	0.75100257	0.74088228	Trpv2/Ctnnd1	2
GO:0005604	basement membrane	1/98	98/14612	0.01020408	1.5214494	0.42558969	0.48402046	0.75100257	0.74088228	Vwa1	1
GO:0016607	nuclear speck	3/98	389/14612	0.00771208	1.1498872	0.24620859	0.48637088	0.75100257	0.74088228	Hspa1b/Rfxap/Mettl3	3
GO:0005802	trans-Golgi network	2/98	243/14612	0.00823045	1.22717729	0.29343656	0.48697907	0.75100257	0.74088228	Smpd4/Becn1	2
GO:0032587	ruffle membrane	1/98	99/14612	0.01010101	1.50608122	0.41516303	0.48750441	0.75100257	0.74088228	Nme1	1
GO:0045177	apical part of cell	3/98	393/14612	0.00763359	1.13818352	0.22817993	0.49318968	0.75100257	0.74088228	Hspa1b/Abcc5/Atp1b3	3
GO:0031985	Golgi cisterna	1/98	101/14612	0.00990099	1.47625783	0.39465135	0.4944026	0.75100257	0.74088228	Fut11	1
GO:0005819	spindle	3/98	394/14612	0.00761421	1.13529473	0.22370164	0.49488763	0.75100257	0.74088228	Efhc1/Tbck/Smc1a	3
GO:0035770	ribonucleoprotein granule	2/98	248/14612	0.00806452	1.20243581	0.26420129	0.49774991	0.75100257	0.74088228	Nufip2/Tuba1a	2
GO:0005769	early endosome	3/98	399/14612	0.0075188	1.12106798	0.20148017	0.50333589	0.75100257	0.74088228	Pcsk9/Nme1/Tbck	3
GO:0005923	bicellular tight junction	1/98	104/14612	0.00961538	1.43367347	0.36469953	0.50457792	0.75100257	0.74088228	Ctnnd1	1
GO:0034708	methyltransferase complex	1/98	104/14612	0.00961538	1.43367347	0.36469953	0.50457792	0.75100257	0.74088228	Mettl3	1
GO:0031594	neuromuscular junction	1/98	105/14612	0.00952381	1.42001944	0.35492344	0.50792445	0.75100257	0.74088228	Tuba1a	1
GO:0030684	preribosome	1/98	107/14612	0.00934579	1.39347702	0.33566947	0.51455051	0.75486537	0.74469302	Rps19bp1	1
GO:0016528	sarcoplasm	1/98	108/14612	0.00925926	1.38057445	0.32618731	0.51783035	0.75486537	0.74469302	Fkbp1b	1
GO:1902554	serine/threonine protein kinase complex	1/98	111/14612	0.00900901	1.34326163	0.29829586	0.52753884	0.75697264	0.74677189	Cdkn1a	1
GO:0030863	cortical cytoskeleton	1/98	112/14612	0.00892857	1.33126822	0.28917732	0.5307318	0.75697264	0.74677189	Cap1	1
GO:0070160	tight junction	1/98	112/14612	0.00892857	1.33126822	0.28917732	0.5307318	0.75697264	0.74677189	Ctnnd1	1
GO:0019897	extrinsic component of plasma membrane	1/98	113/14612	0.00884956	1.31948709	0.28014505	0.53390341	0.75697264	0.74677189	Ctnnd1	1
GO:0005770	late endosome	2/98	270/14612	0.00740741	1.10445956	0.14235892	0.54341759	0.76522069	0.75490879	Pcsk9/Stard3	2
GO:0016363	nuclear matrix	1/98	121/14612	0.00826446	1.23224827	0.21079264	0.55852408	0.76872628	0.75836714	Smc1a	1
GO:0005765	lysosomal membrane	2/98	280/14612	0.00714286	1.06501458	0.0902601	0.56321763	0.76872628	0.75836714	Sphk2/Cln5	2
GO:0098852	lytic vacuole membrane	2/98	280/14612	0.00714286	1.06501458	0.0902601	0.56321763	0.76872628	0.75836714	Sphk2/Cln5	2
GO:0022626	cytosolic ribosome	1/98	123/14612	0.00813008	1.21221171	0.19420578	0.56447533	0.76872628	0.75836714	Hbs1l	1
GO:1902911	protein kinase complex	1/98	123/14612	0.00813008	1.21221171	0.19420578	0.56447533	0.76872628	0.75836714	Cdkn1a	1
GO:0031965	nuclear membrane	2/98	283/14612	0.00706714	1.05372467	0.07499261	0.56903851	0.76969492	0.75932273	Smpd4/Dctn5	2
GO:0043296	apical junction complex	1/98	127/14612	0.00787402	1.17403182	0.16185671	0.57614064	0.76969492	0.75932273	Ctnnd1	1
GO:0005884	actin filament	1/98	128/14612	0.0078125	1.16485969	0.15393404	0.57900831	0.76969492	0.75932273	Ptges3	1
GO:1990204	oxidoreductase complex	1/98	130/14612	0.00769231	1.14693878	0.13827806	0.58468617	0.76969492	0.75932273	P4ha1	1
GO:0005777	peroxisome	1/98	131/14612	0.00763359	1.13818352	0.13054254	0.58749661	0.76969492	0.75932273	Pmvk	1
GO:0042579	microbody	1/98	131/14612	0.00763359	1.13818352	0.13054254	0.58749661	0.76969492	0.75932273	Pmvk	1
GO:0005795	Golgi stack	1/98	134/14612	0.00746269	1.1127018	0.10769323	0.59581546	0.77262296	0.76221131	Fut11	1
GO:0005635	nuclear envelope	3/98	458/14612	0.00655022	0.97665092	-0.0417179	0.59719649	0.77262296	0.76221131	Bicd2/Smpd4/Dctn5	3
GO:0048786	presynaptic active zone	1/98	136/14612	0.00735294	1.09633854	0.09274802	0.60126889	0.77306001	0.76264247	Ctnnd1	1
GO:0062023	collagen-containing extracellular matrix	2/98	306/14612	0.00653595	0.97452314	-0.037009	0.61182053	0.77769506	0.76721506	Vwa1/Adamts8	2
GO:0030018	Z disc	1/98	141/14612	0.0070922	1.05746128	0.05633717	0.6145859	0.77769506	0.76721506	Fkbp1b	1
GO:0098685	Schaffer collateral - CA1 synapse	1/98	144/14612	0.00694444	1.03543084	0.03510895	0.62236379	0.77769506	0.76721506	Ctnnd1	1
GO:0009898	cytoplasmic side of plasma membrane	1/98	147/14612	0.00680272	1.0142996	0.01431801	0.62998629	0.77769506	0.76721506	Traf2	1

GO:0098802	plasma membrane signaling receptor complex	1/98	147/14612	0.00680272	1.0142996	0.01431801	0.62998629	0.77769506	0.76721506	Traf2	1
GO:0005814	centriole	1/98	148/14612	0.00675676	1.00744622	0.00748139	0.63249312	0.77769506	0.76721506	Hspa1b	1
GO:0034399	nuclear periphery	1/98	148/14612	0.00675676	1.00744622	0.00748139	0.63249312	0.77769506	0.76721506	Smc1a	1
GO:0098687	chromosomal region	2/98	321/14612	0.00623053	0.92898468	-0.1057141	0.63795945	0.77769506	0.76721506	Dctn5/Smc1a	2
GO:0031902	late endosome membrane	1/98	152/14612	0.00657895	0.98093448	-0.0194154	0.64235345	0.77769506	0.76721506	Stard3	1
GO:0032993	protein-DNA complex	1/98	154/14612	0.00649351	0.96819507	-0.0326032	0.64718496	0.77769506	0.76721506	Sphk2	1
GO:0031674	I band	1/98	155/14612	0.00645161	0.96194865	-0.0391342	0.64957643	0.77769506	0.76721506	Fkbp1b	1
GO:0015629	actin cytoskeleton	3/98	495/14612	0.00606061	0.90364873	-0.1792042	0.64995771	0.77769506	0.76721506	Ptges3/Cap1/Dctn5	3
GO:0098982	GABA-ergic synapse	1/98	158/14612	0.00632911	0.9436838	-0.0584827	0.65665499	0.78008905	0.76957679	Nrg2	1
GO:0005774	vacuolar membrane	2/98	334/14612	0.00598802	0.89282659	-0.1628119	0.65949557	0.78008905	0.76957679	Sphk2/Cln5	2
GO:0005912	adherens junction	1/98	161/14612	0.00621118	0.92609963	-0.0774765	0.66359199	0.78047467	0.76995722	Ctnnd1	1
GO:0098798	mitochondrial protein-containing complex	1/98	172/14612	0.00581395	0.86687233	-0.144314	0.68786127	0.80444793	0.79360742	Mrps27	1
GO:0034703	cation channel complex	1/98	176/14612	0.00568182	0.84717069	-0.1676096	0.69624984	0.8096838	0.79877273	Fkbp1b	1
GO:0042383	sarcolemma	1/98	178/14612	0.00561798	0.83765191	-0.1790702	0.70036005	0.80991357	0.79899941	Smpd4	1
GO:0001726	ruffle	1/98	187/14612	0.00534759	0.79733712	-0.2291905	0.71818511	0.81683691	0.80582945	Nme1	1
GO:0005925	focal adhesion	1/98	187/14612	0.00534759	0.79733712	-0.2291905	0.71818511	0.81683691	0.80582945	Shc1	1
GO:0055037	recycling endosome	1/98	187/14612	0.00534759	0.79733712	-0.2291905	0.71818511	0.81683691	0.80582945	Tuba1a	1
GO:0031256	leading edge membrane	1/98	192/14612	0.00520833	0.77657313	-0.2560724	0.72763013	0.82066633	0.80960726	Nme1	1
GO:0044391	ribosomal subunit	1/98	193/14612	0.00518135	0.77254943	-0.2613711	0.72948118	0.82066633	0.80960726	Mrps27	1
GO:1902495	transmembrane transporter complex	2/98	388/14612	0.00515464	0.76856722	-0.3796552	0.73822654	0.82252228	0.8114382	Fkbp1b/Atp1b3	2
GO:0030055	cell-substrate junction	1/98	199/14612	0.00502513	0.74925649	-0.2926432	0.74032891	0.82252228	0.8114382	Shc1	1
GO:0150034	distal axon	2/98	393/14612	0.00508906	0.75878901	-0.3983073	0.74468005	0.82252228	0.8114382	Trpv2/Ctnnd1	2
GO:0030312	external encapsulating structure	2/98	398/14612	0.00502513	0.74925649	-0.4167471	0.7509986	0.82252228	0.8114382	Vwa1/Adamts8	2
GO:0031012	extracellular matrix	2/98	398/14612	0.00502513	0.74925649	-0.4167471	0.7509986	0.82252228	0.8114382	Vwa1/Adamts8	2
GO:1990351	transporter complex	2/98	412/14612	0.00485437	0.72379631	-0.4672974	0.7679877	0.83670239	0.82542723	Fkbp1b/Atp1b3	2
GO:0030017	sarcomere	1/98	221/14612	0.00452489	0.67466987	-0.4004372	0.77654548	0.84159641	0.83025529	Fkbp1b	1
GO:0043197	dendritic spine	1/98	227/14612	0.00440529	0.65683718	-0.4281714	0.78552308	0.84689207	0.83547959	Ctnnd1	1
GO:0044309	neuron spine	1/98	232/14612	0.00431034	0.64268121	-0.4507958	0.79273098	0.85023478	0.83877726	Ctnnd1	1
GO:0030135	coated vesicle	1/98	240/14612	0.00416667	0.6212585	-0.4861266	0.80376805	0.85465111	0.84313407	Pcsk9	1
GO:0030016	myofibril	1/98	241/14612	0.00414938	0.61868067	-0.490471	0.80510612	0.85465111	0.84313407	Fkbp1b	1
GO:0043292	contractile muscle fiber	1/98	249/14612	0.00401606	0.59880338	-0.5246792	0.81549055	0.85842978	0.84686182	Fkbp1b	1
GO:0005667	transcription regulator complex	2/98	458/14612	0.00436681	0.65110061	-0.6233799	0.81695974	0.85842978	0.84686182	Rfxap/Taf4b	2
GO:0034702	monoatomic ion channel complex	1/98	271/14612	0.00369004	0.55019203	-0.61416	0.8413076	0.87954886	0.86769631	Fkbp1b	1
GO:0009897	external side of plasma membrane	1/98	298/14612	0.0033557	0.50034242	-0.7160767	0.86815067	0.9030512	0.89088193	Antxr2	1
GO:0000151	ubiquitin ligase complex	1/98	306/14612	0.00326797	0.48726157	-0.7448296	0.87520325	0.90308357	0.89091387	Traf2	1
GO:0043235	receptor complex	1/98	308/14612	0.00324675	0.48409754	-0.7519235	0.87690723	0.90308357	0.89091387	Traf2	1
GO:0031253	cell projection membrane	1/98	324/14612	0.00308642	0.46019148	-0.8073948	0.88973534	0.9117585	0.8994719	Nme1	1
GO:0030133	transport vesicle	1/98	411/14612	0.00243309	0.36277869	-1.0767408	0.93952153	0.95803427	0.94512407	Aph1b	1
GO:0014069	postsynaptic density	1/98	443/14612	0.00225734	0.33657346	-1.1651573	0.95155402	0.96364839	0.95066254	Ctnnd1	1
GO:0032279	asymmetric synapse	1/98	465/14612	0.00215054	0.32064955	-1.2233412	0.95841898	0.96364839	0.95066254	Ctnnd1	1
GO:0005911	cell-cell junction	1/98	467/14612	0.00214133	0.31927632	-1.2285343	0.95899309	0.96364839	0.95066254	Ctnnd1	1
GO:0099572	postsynaptic specialization	1/98	487/14612	0.00205339	0.30616435	-1.2796356	0.96431966	0.96431966	0.95132476	Ctnnd1	1

ID	Description	GeneRatio	BgRatio	RichFactor	FoldEnrichm	zScore	pvalue	p.adjust	qvalue	geneID	Count
GO:0034663	endoplasmic reticulum chaperone complex	5/140	11/14612	0.45454545	47.4415584	15.1548464	3.3154E-08	8.9515E-06	8.5502E-06	Sdf2l1/Hspa5/Hsp90b1/Dnajb11/P4hb	5
GO:0005788	endoplasmic reticulum lumen	9/140	105/14612	0.08571429	8.94612245	8.03712658	7.2458E-07	9.7819E-05	9.3433E-05	Sdf2l1/Hspa5/Manf/Calr/Hsp90b1/Pdia3/Dnajc3/Dnajb11/P4hb	9
GO:0005790	smooth endoplasmic reticulum	5/140	34/14612	0.14705882	15.3487395	8.23842036	1.6724E-05	0.00125774	0.00120135	Hspa5/Calr/Hsp90b1/Pdia3/Dnajc3	5
GO:0140534	endoplasmic reticulum protein-containing complex	8/140	119/14612	0.06722689	7.01656663	6.48160821	1.8633E-05	0.00125774	0.00120135	Sdf2l1/Hspa5/Calr/Hsp90b1/Pdia3/Dnajb11/Srprb/P4hb	8
GO:0042470	melanosome	7/140	98/14612	0.07142857	7.45510204	6.30612103	4.2679E-05	0.00192055	0.00183444	Hspa5/Mmp14/Hsp90b1/Pdia3/Rab38/P4hb/Sec22b	7
GO:0048770	pigment granule	7/140	98/14612	0.07142857	7.45510204	6.30612103	4.2679E-05	0.00192055	0.00183444	Hspa5/Mmp14/Hsp90b1/Pdia3/Rab38/P4hb/Sec22b	7
GO:0016528	sarcoplasm	5/140	108/14612	0.0462963	4.83201058	3.93128377	0.00380604	0.14680425	0.14022239	Manf/Calr/Hsp90b1/Sri/Bag5	5
GO:0101031	protein folding chaperone complex	3/140	35/14612	0.08571429	8.94612245	4.62908238	0.00450248	0.15195859	0.14514563	Sdf2l1/Stip1/Dnajb11	3
GO:0042824	MHC class I peptide loading complex	2/140	12/14612	0.16666667	17.3952381	5.58819605	0.00564879	0.16946356	0.16186578	Calr/Pdia3	2
GO:0016529	sarcoplasmic reticulum	4/140	80/14612	0.05	5.21857143	3.72123774	0.00727671	0.19465482	0.18592761	Manf/Calr/Hsp90b1/Sri	4
GO:1903561	extracellular vesicle	4/140	82/14612	0.04878049	5.0912892	3.65404599	0.00793038	0.19465482	0.18592761	Has2/Hspd1/Snx18/Sri	4
GO:0043230	extracellular organelle	4/140	90/14612	0.04444444	4.63873016	3.40563108	0.01092783	0.21283359	0.20329134	Has2/Hspd1/Snx18/Sri	4
GO:0065010	extracellular membrane-bounded organelle	4/140	90/14612	0.04444444	4.63873016	3.40563108	0.01092783	0.21283359	0.20329134	Has2/Hspd1/Snx18/Sri	4
GO:0030057	desmosome	2/140	18/14612	0.11111111	11.5968254	4.42450379	0.01261236	0.21283359	0.20329134	Pkp3/Pkp2	2
GO:0030992	intraciliary transport particle B	2/140	18/14612	0.11111111	11.5968254	4.42450379	0.01261236	0.21283359	0.20329134	Ift172/Ift80	2
GO:0045120	pronucleus	2/140	18/14612	0.11111111	11.5968254	4.42450379	0.01261236	0.21283359	0.20329134	Hnrnpc/Cbx1	2
GO:0005793	endoplasmic reticulum-Golgi intermediate compartment	4/140	99/14612	0.04040404	4.21702742	3.15888251	0.01507751	0.23946641	0.2287301	Hspa5/Lman2/P4hb/Sec22b	4
GO:0031462	Cul2-RING ubiquitin ligase complex	2/140	22/14612	0.09090909	9.48831169	3.91872443	0.01857325	0.26914696	0.25707994	Arih1/Cul2	2
GO:0019005	SCF ubiquitin ligase complex	3/140	59/14612	0.05084746	5.30702179	3.26036725	0.01893997	0.26914696	0.25707994	Arih1/Cul2/Fbxo25	3
GO:0030990	intraciliary transport particle	2/140	24/14612	0.08333333	8.69761905	3.71196497	0.0219171	0.29588079	0.28261518	Ift172/Ift80	2
GO:0070062	extracellular exosome	3/140	68/14612	0.04411765	4.60462185	2.93029906	0.02744735	0.34451115	0.32906523	Hspd1/Snx18/Sri	3
GO:0030135	coated vesicle	6/140	240/14612	0.025	2.60928571	2.47240654	0.02807128	0.34451115	0.32906523	Slc17a8/Hspd1/Lman2/Snx18/Atp6v0b/Sec22b	6
GO:0046540	U4/U6 x U5 tri-snRNP complex	2/140	37/14612	0.05405405	5.64169884	2.78043823	0.04880248	0.52706679	0.50343612	Prpf31/Prpf18	2
GO:0097225	sperm midpiece	2/140	37/14612	0.05405405	5.64169884	2.78043823	0.04880248	0.52706679	0.50343612	Hspd1/Ift172	2
GO:0097526	spliceosomal tri-snRNP complex	2/140	37/14612	0.05405405	5.64169884	2.78043823	0.04880248	0.52706679	0.50343612	Prpf31/Prpf18	2
GO:0005912	adherens junction	4/140	161/14612	0.02484472	2.59307897	1.99912985	0.0691203	0.55737159	0.53238222	Pkp3/Pkp2/Cdh10/Cd99l2	4
GO:0002102	podosome	2/140	48/14612	0.04166667	4.34880952	2.28565375	0.07727228	0.55737159	0.53238222	Scin/Sh3pxd2a	2
GO:0034708	methyltransferase complex	3/140	104/14612	0.02884615	3.01071429	2.02396487	0.0780973	0.55737159	0.53238222	Prpf31/Mettl14/Virma	3
GO:0030141	secretory granule	6/140	318/14612	0.01886792	1.96927224	1.71878738	0.08549159	0.55737159	0.53238222	Calr/Fzd8/Pdia3/Hspd1/Hexa/Sri	6
GO:0030670	phagocytic vesicle membrane	2/140	52/14612	0.03846154	4.01428571	2.1416333	0.08865145	0.55737159	0.53238222	Rab38/Rab9b	2
GO:0000153	cytoplasmic ubiquitin ligase complex	1/140	10/14612	0.1	10.4371429	2.93612181	0.09181158	0.55737159	0.53238222	Cul2	1
GO:0005687	U4 snRNP	1/140	10/14612	0.1	10.4371429	2.93612181	0.09181158	0.55737159	0.53238222	Prpf31	1
GO:0017119	Golgi transport complex	1/140	10/14612	0.1	10.4371429	2.93612181	0.09181158	0.55737159	0.53238222	Cog6	1
GO:0044194	cytolytic granule	1/140	10/14612	0.1	10.4371429	2.93612181	0.09181158	0.55737159	0.53238222	Calr	1
GO:0097441	basal dendrite	1/140	10/14612	0.1	10.4371429	2.93612181	0.09181158	0.55737159	0.53238222	Slc17a8	1
GO:0015030	Cajal body	2/140	54/14612	0.03703704	3.86560847	2.07492587	0.09451678	0.55737159	0.53238222	Prpf31/Arih1	2
GO:0000323	lytic vacuole	8/140	487/14612	0.0164271	1.71452039	1.57733736	0.09630711	0.55737159	0.53238222	Calr/Has2/Rab38/Hexa/Rab9b/Snx14/Rmc1/Wdr45b	8
GO:0005764	lysosome	8/140	487/14612	0.0164271	1.71452039	1.57733736	0.09630711	0.55737159	0.53238222	Calr/Has2/Rab38/Hexa/Rab9b/Snx14/Rmc1/Wdr45b	8
GO:0031463	Cul3-RING ubiquitin ligase complex	2/140	55/14612	0.03636364	3.79532468	2.04276013	0.09749055	0.55737159	0.53238222	Klhl5/Arih1	2
GO:0005766	primary lysosome	1/140	11/14612	0.09090909	9.48831169	2.76991264	0.10051905	0.55737159	0.53238222	Hexa	1
GO:0042582	azurophil granule	1/140	11/14612	0.09090909	9.48831169	2.76991264	0.10051905	0.55737159	0.53238222	Hexa	1
GO:0097450	astrocyte end-foot	1/140	11/14612	0.09090909	9.48831169	2.76991264	0.10051905	0.55737159	0.53238222	Slc17a8	1
GO:0045335	phagocytic vesicle	3/140	117/14612	0.02564103	2.67619048	1.79038694	0.10224504	0.55737159	0.53238222	Calr/Rab38/Rab9b	3
GO:0030134	COPII-coated ER to Golgi transport vesicle	2/140	57/14612	0.03508772	3.66215539	1.98063494	0.10351639	0.55737159	0.53238222	Lman2/Sec22b	2
GO:0005801	cis-Golgi network	2/140	58/14612	0.03448276	3.59901478	1.95061371	0.10656694	0.55737159	0.53238222	Fktn/Phtf1	2
GO:0072379	ER membrane insertion complex	1/140	12/14612	0.08333333	8.69761905	2.62367671	0.10914361	0.55737159	0.53238222	Ubl4a	1
GO:0031461	cullin-RING ubiquitin ligase complex	4/140	190/14612	0.02105263	2.19729323	1.63382032	0.10981391	0.55737159	0.53238222	Klhl5/Arih1/Cul2/Fbxo25	4
GO:0030662	coated vesicle membrane	3/140	123/14612	0.02439024	2.5456446	1.69310036	0.11427861	0.55737159	0.53238222	Slc17a8/Atp6v0b/Sec22b	3
GO:0030897	HOPS complex	1/140	13/14612	0.07692308	8.02857143	2.4935432	0.11768607	0.55737159	0.53238222	Aktip	1
GO:0031464	Cul4A-RING E3 ubiquitin ligase complex	1/140	13/14612	0.07692308	8.02857143	2.4935432	0.11768607	0.55737159	0.53238222	Arih1	1
GO:0033391	chromatoid body	1/140	13/14612	0.07692308	8.02857143	2.4935432	0.11768607	0.55737159	0.53238222	Bmal1	1
GO:0044322	endoplasmic reticulum quality control compartment	1/140	13/14612	0.07692308	8.02857143	2.4935432	0.11768607	0.55737159	0.53238222	Calr	1
GO:0097525	spliceosomal snRNP complex	2/140	63/14612	0.03174603	3.31337868	1.80983984	0.12216646	0.55737159	0.53238222	Prpf31/Prpf18	2
GO:0030061	mitochondrial crista	1/140	14/14612	0.07142857	7.45510204	2.37662226	0.1261472	0.55737159	0.53238222	Hspd1	1
GO:0042583	chromaffin granule	1/140	14/14612	0.07142857	7.45510204	2.37662226	0.1261472	0.55737159	0.53238222	Sri	1
GO:0051286	cell tip	1/140	14/14612	0.07142857	7.45510204	2.37662226	0.1261472	0.55737159	0.53238222	Pkp2	1
GO:0030139	endocytic vesicle	4/140	203/14612	0.01970443	2.05657987	1.49098455	0.1308809	0.55737159	0.53238222	Calr/Rab38/Rab9b/Gapvd1	4
GO:0010369	chromocenter	1/140	15/14612	0.06666667	6.95809524	2.27070637	0.13452776	0.55737159	0.53238222	Cbx1	1
GO:0019814	immunoglobulin complex	1/140	15/14612	0.06666667	6.95809524	2.27070637	0.13452776	0.55737159	0.53238222	Ighg2c	1
GO:0033106	cis-Golgi network membrane	1/140	16/14612	0.0625	6.52321429	2.17407573	0.14282851	0.55737159	0.53238222	Phtf1	1
GO:0033162	melanosome membrane	1/140	16/14612	0.0625	6.52321429	2.17407573	0.14282851	0.55737159	0.53238222	Rab38	1
GO:0045009	chitosome	1/140	16/14612	0.0625	6.52321429	2.17407573	0.14282851	0.55737159	0.53238222	Rab38	1
GO:0090741	pigment granule membrane	1/140	16/14612	0.0625	6.52321429	2.17407573	0.14282851	0.55737159	0.53238222	Rab38	1
GO:0120293	dynein axonemal particle	1/140	16/14612	0.0625	6.52321429	2.17407573	0.14282851	0.55737159	0.53238222	Stip1	1
GO:0043209	myelin sheath	4/140	210/14612	0.01904762	1.98802721	1.41842658	0.14287122	0.55737159	0.53238222	Hspa5/Stip1/Pdia3/Hspd1	4
GO:0099023	vesicle tethering complex	2/140	70/14612	0.02857143	2.98204082	1.6348927	0.14485065	0.55737159	0.53238222	Cog6/Aktip	2
GO:0008076	voltage-gated potassium channel complex	2/140	71/14612	0.02816901	2.94004024	1.61169362	0.14816035	0.55737159	0.53238222	Slc5a3/Kcnip2	2
GO:0030532	small nuclear ribonucleoprotein complex	2/140	71/14612	0.02816901	2.94004024	1.61169362	0.14816035	0.55737159	0.53238222	Prpf31/Prpf18	2
GO:0043198	dendritic shaft	2/140	71/14612	0.02816901	2.94004024	1.61169362	0.14816035	0.55737159	0.53238222	Hspa5/Zfp804a	2
GO:0005682	U5 snRNP	1/140	17/14612	0.05882353	6.1394958	2.08536768	0.15105022	0.55737159	0.53238222	Prpf18	1
GO:0042588	zymogen granule	1/140	17/14612	0.05882353	6.1394958	2.08536768	0.15105022	0.55737159	0.53238222	Hspd1	1
GO:0060187	cell pole	1/140	17/14612	0.05882353	6.1394958	2.08536768	0.15105022	0.55737159	0.53238222	Pkp2	1
GO:0090443	FAR/SIN/STRIPAK complex	1/140	17/14612	0.05882353	6.1394958	2.08536768	0.15105022	0.55737159	0.53238222	Ppp2cb	1
GO:0016234	inclusion body	2/140	73/14612	0.02739726	2.85949119	1.56649134	0.15482544	0.55737159	0.53238222	Bag5/Arih1	2
GO:0030666	endocytic vesicle membrane	2/140	73/14612	0.02739726	2.85949119	1.56649134	0.15482544	0.55737159	0.53238222	Rab38/Rab9b	2
GO:0030665	clathrin-coated vesicle membrane	2/140	74/14612	0.02702703	2.82084942	1.54446216	0.15817965	0.55821139	0.53318437	Slc17a8/Atp6v0b	2
GO:0000159	protein phosphatase type 2A complex	1/140	18/14612	0.05555556	5.7984127	2.00348636	0.15919362	0.55821139	0.53318437	Ppp2cb	1
GO:0030315	T-tubule	2/140	75/14612	0.02666667	2.7832381	1.52279794	0.16154752	0.55920296	0.53413149	Rrad/Sri	2
GO:0001533	cornified envelope	1/140	19/14612	0.05263158	5.49323308	1.92753898	0.16725946	0.56450068	0.53919168	Pkp3	1
GO:0032039	integrator complex	1/140	19/14612	0.05263158	5.49323308	1.92753898	0.16725946	0.56450068	0.53919168	Ints10	1
GO:0000151	ubiquitin ligase complex	5/140	306/14612	0.01633987	1.7054155	1.22655298	0.17068055	0.56843189	0.54294664	Klhl5/Itch/Arih1/Cul2/Fbxo25	5
GO:0005697	telomerase holoenzyme complex	1/140	20/14612	0.05	5.21857143	1.85678965	0.17524847	0.56843189	0.54294664	Hnrnpc	1
GO:0033177	proton-transporting two-sector ATPase complex, proton-transporting domain	1/140	20/14612	0.05	5.21857143	1.85678965	0.17524847	0.56843189	0.54294664	Atp6v0b	1
GO:0034045	phagophore assembly site membrane	1/140	21/14612	0.04761905	4.97006803	1.79062556	0.18316139	0.56843189	0.54294664	Wdr45b	1
GO:0044233	mitochondria-associated endoplasmic reticulum membrane contact site	1/140	21/14612	0.04761905	4.97006803	1.79062556	0.18316139	0.56843189	0.54294664	Rab38	1
GO:0070822	Sin3-type complex	1/140	21/14612	0.04761905	4.97006803	1.79062556	0.18316139	0.56843189	0.54294664	Brms1	1
GO:0097228	sperm principal piece	1/140	21/14612	0.04761905	4.97006803	1.79062556	0.18316139	0.56843189	0.54294664	Ift172	1
GO:0030136	clathrin-coated vesicle	3/140	155/14612	0.01935484	2.02009217	1.25575779	0.18608957	0.57095663	0.54535819	Slc17a8/Snx18/Atp6v0b	3
GO:0120114	Sm-like protein family complex	2/140	83/14612	0.02409639	2.51497418	1.3613385	0.18891701	0.57299675	0.54730684	Prpf31/Prpf18	2
GO:0016471	vacuolar proton-transporting V-type ATPase complex	1/140	22/14612	0.04545455	4.74415584	1.7285316	0.19099892	0.57299675	0.54730684	Atp6v0b	1
GO:0034705	potassium channel complex	2/140	85/14612	0.02352941	2.45579832	1.32392206	0.19585875	0.57705032	0.55117867	Slc5a3/Kcnip2	2
GO:0032045	guanyl-nucleotide exchange factor complex	1/140	23/14612	0.04347826	4.5378882	1.67007114	0.19876178	0.57705032	0.55117867	Rmc1	1
GO:0097449	astrocyte projection	1/140	23/14612	0.04347826	4.5378882	1.67007114	0.19876178	0.57705032	0.55117867	Slc17a8	1
GO:0033176	proton-transporting V-type ATPase complex	1/140	25/14612	0.04	4.17485714	1.56261113	0.21406631	0.60638973	0.57920267	Atp6v0b	1
GO:0005798	Golgi-associated vesicle	2/140	91/14612	0.02197802	2.29387755	1.21774211	0.2168605	0.60638973	0.57920267	Stk16/Sec22b	2
GO:0005811	lipid droplet	2/140	93/14612	0.02150538	2.24454685	1.18419735	0.223908	0.60638973	0.57920267	Dhrs3/Stard13	2
GO:0045111	intermediate filament cytoskeleton	2/140	94/14612	0.0212766	2.22066869	1.16774509	0.22743863	0.60638973	0.57920267	Mmp14/Pkp2	2
GO:0005782	peroxisomal matrix	1/140	27/14612	0.03703704	3.86560847	1.46583548	0.22908056	0.60638973	0.57920267	Hspd1	1
GO:0016342	catenin complex	1/140	27/14612	0.03703704	3.86560847	1.46583548	0.22908056	0.60638973	0.57920267	Cdh10	1
GO:0030867	rough endoplasmic reticulum membrane	1/140	27/14612	0.03703704	3.86560847	1.46583548	0.22908056	0.60638973	0.57920267	Srprb	1
GO:0031907	microbody lumen	1/140	27/14612	0.03703704	3.86560847	1.46583548	0.22908056	0.60638973	0.57920267	Hspd1	1
GO:0043202	lysosomal lumen	1/140	27/14612	0.03703704	3.86560847	1.46583548	0.22908056	0.60638973	0.57920267	Hexa	1
GO:0005684	U2-type spliceosomal complex	2/140	96/14612	0.02083333	2.17440476	1.13545428	0.23451161	0.61241958	0.58496218	Prpf31/Prpf18	2
GO:0012507	ER to Golgi transport vesicle membrane	1/140	29/14612	0.03448276	3.59901478	1.37792006	0.24381	0.61241958	0.58496218	Sec22b	1
GO:0001669	acrosomal vesicle	2/140	99/14612	0.02020202	2.10851371	1.08847831	0.24514454	0.61241958	0.58496218	Calr/Pdia3	2
GO:0005759	mitochondrial matrix	5/140	351/14612	0.01424501	1.48677249	0.90791314	0.24692024	0.61241958	0.58496218	Hspd1/Mrps6/Tfb1m/Pdk3/Yars2	5
GO:0005721	pericentric heterochromatin	1/140	30/14612	0.03333333	3.47904762	1.33683147	0.25106959	0.61241958	0.58496218	Cbx1	1
GO:0097440	apical dendrite	1/140	30/14612	0.03333333	3.47904762	1.33683147	0.25106959	0.61241958	0.58496218	Slc17a8	1
GO:0005775	vacuolar lumen	1/140	31/14612	0.03225806	3.36682028	1.29745464	0.25825998	0.61241958	0.58496218	Hexa	1
GO:0030137	COPI-coated vesicle	1/140	31/14612	0.03225806	3.36682028	1.29745464	0.25825998	0.61241958	0.58496218	Sec22b	1
GO:0005770	late endosome	4/140	270/14612	0.01481481	1.54624339	0.89105175	0.25984945	0.61241958	0.58496218	Slc17a8/Rab9b/Snx14/Rmc1	4
GO:0034702	monoatomic ion channel complex	4/140	271/14612	0.01476015	1.54053769	0.88340656	0.26195579	0.61241958	0.58496218	Slc5a3/Kcnk13/Slc17a8/Kcnip2	4
GO:0000314	organellar small ribosomal subunit	1/140	32/14612	0.03125	3.26160714	1.25965932	0.26538182	0.61241958	0.58496218	Mrps6	1
GO:0005763	mitochondrial small ribosomal subunit	1/140	32/14612	0.03125	3.26160714	1.25965932	0.26538182	0.61241958	0.58496218	Mrps6	1
GO:0008180	COP9 signalosome	1/140	32/14612	0.03125	3.26160714	1.25965932	0.26538182	0.61241958	0.58496218	Hspa5	1
GO:0071339	MLL1 complex	1/140	32/14612	0.03125	3.26160714	1.25965932	0.26538182	0.61241958	0.58496218	Prpf31	1
GO:0080008	Cul4-RING E3 ubiquitin ligase complex	1/140	32/14612	0.03125	3.26160714	1.25965932	0.26538182	0.61241958	0.58496218	Arih1	1
GO:0044665	MLL1/2 complex	1/140	33/14612	0.03030303	3.16277056	1.22332898	0.27243577	0.62336998	0.59542161	Prpf31	1
GO:0005681	spliceosomal complex	3/140	194/14612	0.01546392	1.61399116	0.84674021	0.28469262	0.63894615	0.61029944	Prpf31/Hnrnpc/Prpf18	3
GO:0034707	chloride channel complex	1/140	35/14612	0.02857143	2.98204082	1.15465503	0.28634253	0.63894615	0.61029944	Slc17a8	1
GO:0098992	neuronal dense core vesicle	1/140	35/14612	0.02857143	2.98204082	1.15465503	0.28634253	0.63894615	0.61029944	Fzd8	1
GO:0001772	immunological synapse	1/140	36/14612	0.02777778	2.89920635	1.12213197	0.29319662	0.64796835	0.61891714	Card11	1
GO:0000407	phagophore assembly site	1/140	37/14612	0.02702703	2.82084942	1.09071258	0.29998535	0.64796835	0.61891714	Wdr45b	1
GO:0090533	cation-transporting ATPase complex	1/140	37/14612	0.02702703	2.82084942	1.09071258	0.29998535	0.64796835	0.61891714	Atp6v0b	1
GO:0098533	ATPase dependent transmembrane transport complex	1/140	37/14612	0.02702703	2.82084942	1.09071258	0.29998535	0.64796835	0.61891714	Atp6v0b	1
GO:0033017	sarcoplasmic reticulum membrane	1/140	39/14612	0.02564103	2.67619048	1.03091035	0.31336918	0.66992809	0.63989233	Sri	1
GO:1902495	transmembrane transporter complex	5/140	388/14612	0.0128866	1.34499264	0.67741456	0.31511432	0.66992809	0.63989233	Slc5a3/Kcnk13/Slc17a8/Kcnip2/Atp6v0b	5
GO:0033116	endoplasmic reticulum-Golgi intermediate compartment membrane	1/140	42/14612	0.02380952	2.48503401	0.94791846	0.33297001	0.70235861	0.67086884	Sec22b	1
GO:0097386	glial cell projection	1/140	43/14612	0.02325581	2.42724252	0.92184275	0.33937935	0.70699381	0.67529624	Slc17a8	1
GO:0016469	proton-transporting two-sector ATPase complex	1/140	44/14612	0.02272727	2.37207792	0.89648878	0.34572755	0.70699381	0.67529624	Atp6v0b	1
GO:0031201	SNARE complex	1/140	45/14612	0.02222222	2.31936508	0.87181812	0.35201518	0.70699381	0.67529624	Sec22b	1
GO:0005777	peroxisome	2/140	131/14612	0.01526718	1.59345692	0.67106608	0.35803622	0.70699381	0.67529624	Hspd1/Far2	2
GO:0042579	microbody	2/140	131/14612	0.01526718	1.59345692	0.67106608	0.35803622	0.70699381	0.67529624	Hspd1/Far2	2
GO:0044853	plasma membrane raft	2/140	131/14612	0.01526718	1.59345692	0.67106608	0.35803622	0.70699381	0.67529624	Has2/Cr1l	2
GO:1990351	transporter complex	5/140	412/14612	0.01213592	1.26664355	0.53997696	0.36065588	0.70699381	0.67529624	Slc5a3/Kcnk13/Slc17a8/Kcnip2/Atp6v0b	5
GO:0009295	nucleoid	1/140	47/14612	0.0212766	2.22066869	0.82438713	0.36441101	0.70699381	0.67529624	Tfb1m	1
GO:0042645	mitochondrial nucleoid	1/140	47/14612	0.0212766	2.22066869	0.82438713	0.36441101	0.70699381	0.67529624	Tfb1m	1
GO:0044232	organelle membrane contact site	1/140	47/14612	0.0212766	2.22066869	0.82438713	0.36441101	0.70699381	0.67529624	Rab38	1
GO:0008287	protein serine/threonine phosphatase complex	1/140	48/14612	0.02083333	2.17440476	0.80156325	0.37052034	0.70699381	0.67529624	Ppp2cb	1
GO:1903293	phosphatase complex	1/140	48/14612	0.02083333	2.17440476	0.80156325	0.37052034	0.70699381	0.67529624	Ppp2cb	1
GO:0090575	RNA polymerase II transcription regulator complex	3/140	227/14612	0.01321586	1.37935809	0.56656149	0.37112594	0.70699381	0.67529624	Bmal1/Smad2/Tbpl1	3
GO:0036126	sperm flagellum	2/140	135/14612	0.01481481	1.54624339	0.62712411	0.37182638	0.70699381	0.67529624	Hspd1/Ift172	2
GO:0016323	basolateral plasma membrane	3/140	235/14612	0.01276596	1.33240122	0.5052451	0.39192547	0.72933306	0.69663392	Slc5a3/Slc51a/Cr1l	3
GO:0030018	Z disc	2/140	141/14612	0.0141844	1.4804458	0.56382508	0.39228762	0.72933306	0.69663392	Kcnn1/Sri	2
GO:0005771	multivesicular body	1/140	52/14612	0.01923077	2.00714286	0.71556942	0.3943801	0.72933306	0.69663392	Slc17a8	1
GO:0031045	dense core granule	1/140	52/14612	0.01923077	2.00714286	0.71556942	0.3943801	0.72933306	0.69663392	Fzd8	1
GO:0071005	U2-type precatalytic spliceosome	1/140	53/14612	0.01886792	1.96927224	0.69527665	0.40020337	0.73506741	0.70211117	Prpf31	1
GO:0001673	male germ cell nucleus	1/140	54/14612	0.01851852	1.93280423	0.67542362	0.40597104	0.74062284	0.70741753	Tbpl1	1
GO:0071011	precatalytic spliceosome	1/140	55/14612	0.01818182	1.89766234	0.65599136	0.41168364	0.74133064	0.7080936	Prpf31	1
GO:0005778	peroxisomal membrane	1/140	56/14612	0.01785714	1.86377551	0.63696209	0.4173417	0.74133064	0.7080936	Far2	1
GO:0005791	rough endoplasmic reticulum	1/140	56/14612	0.01785714	1.86377551	0.63696209	0.4173417	0.74133064	0.7080936	Srprb	1
GO:0031903	microbody membrane	1/140	56/14612	0.01785714	1.86377551	0.63696209	0.4173417	0.74133064	0.7080936	Far2	1
GO:0014704	intercalated disc	1/140	58/14612	0.01724138	1.79950739	0.60004664	0.42849622	0.74728724	0.71378313	Pkp2	1
GO:0031902	late endosome membrane	2/140	152/14612	0.01315789	1.37330827	0.45503518	0.42899823	0.74728724	0.71378313	Snx14/Rmc1	2
GO:0097729	9+2 motile cilium	2/140	152/14612	0.01315789	1.37330827	0.45503518	0.42899823	0.74728724	0.71378313	Hspd1/Ift172	2
GO:0031674	I band	2/140	155/14612	0.01290323	1.34672811	0.42683058	0.43881102	0.75948061	0.72542983	Kcnn1/Sri	2
GO:0010008	endosome membrane	5/140	456/14612	0.01096491	1.14442356	0.30816967	0.44430211	0.7601554	0.72607436	Snx21/Snx14/Snx18/Itch/Rmc1	5
GO:0005882	intermediate filament	1/140	61/14612	0.01639344	1.71100703	0.54730838	0.44483168	0.7601554	0.72607436	Pkp2	1
GO:0009925	basal plasma membrane	3/140	258/14612	0.01162791	1.21362126	0.34049252	0.45062135	0.76520607	0.73089858	Slc5a3/Slc51a/Cr1l	3
GO:0043073	germ cell nucleus	1/140	66/14612	0.01515152	1.58138528	0.4655903	0.47103351	0.79486904	0.75923164	Tbpl1	1
GO:0005905	clathrin-coated pit	1/140	68/14612	0.01470588	1.53487395	0.43481416	0.48116708	0.80194513	0.76599047	Hspd1	1
GO:0016591	RNA polymerase II, holoenzyme	1/140	68/14612	0.01470588	1.53487395	0.43481416	0.48116708	0.80194513	0.76599047	Tbpl1	1
GO:0036064	ciliary basal body	2/140	170/14612	0.01176471	1.22789916	0.29396338	0.48645238	0.80578001	0.76965342	Ift172/Ift80	2
GO:0045178	basal part of cell	3/140	275/14612	0.01090909	1.1385974	0.2282086	0.49252375	0.80633387	0.77018245	Slc5a3/Slc51a/Cr1l	3
GO:0035097	histone methyltransferase complex	1/140	71/14612	0.01408451	1.47002012	0.39047047	0.49600712	0.80633387	0.77018245	Prpf31	1

GO:0048787	presynaptic active zone membrane	1/140	71/14612	0.01408451	1.47002012	0.39047047	0.49600712	0.80633387	0.77018245	Cdh10	1
GO:0031901	early endosome membrane	2/140	174/14612	0.01149425	1.19967159	0.26060161	0.49873243	0.80633387	0.77018245	Snx21/Itch	2
GO:0034703	cation channel complex	2/140	176/14612	0.01136364	1.18603896	0.24421735	0.5048025	0.81128973	0.77491612	Slc5a3/Kcnip2	2
GO:0042383	sarcolemma	2/140	178/14612	0.01123596	1.17271268	0.22802401	0.51082532	0.81377775	0.77729259	Rrad/Sri	2
GO:0016607	nuclear speck	4/140	389/14612	0.01028278	1.07322806	0.14397809	0.51423077	0.81377775	0.77729259	Srpk1/Prpf31/Virma/Prpf18	4
GO:0150034	distal axon	4/140	393/14612	0.01017812	1.06230462	0.12314639	0.52230499	0.81377775	0.77729259	Slc17a8/Zfp804a/Snx18/Sri	4
GO:0043679	axon terminus	2/140	182/14612	0.01098901	1.14693878	0.19619081	0.52272758	0.81377775	0.77729259	Slc17a8/Sri	2
GO:0015935	small ribosomal subunit	1/140	77/14612	0.01298701	1.3554731	0.30759856	0.52443455	0.81377775	0.77729259	Mrps6	1
GO:0030667	secretory granule membrane	1/140	77/14612	0.01298701	1.3554731	0.30759856	0.52443455	0.81377775	0.77729259	Sri	1
GO:0005769	early endosome	4/140	399/14612	0.01002506	1.04633011	0.09228829	0.53429819	0.82334082	0.78642691	Rab38/Hspd1/Snx21/Itch	4
GO:0000118	histone deacetylase complex	1/140	81/14612	0.01234568	1.28853616	0.25611482	0.5424958	0.82334082	0.78642691	Brms1	1
GO:0030496	midbody	2/140	190/14612	0.01052632	1.09864662	0.13461268	0.54594894	0.82334082	0.78642691	Hspa5/Hsp90b1	2
GO:0009897	external side of plasma membrane	3/140	298/14612	0.01006711	1.05071908	0.0870039	0.54661744	0.82334082	0.78642691	Calr/Cr1l/P4hb	3
GO:0044291	cell-cell contact zone	1/140	82/14612	0.01219512	1.2728223	0.24366531	0.54690366	0.82334082	0.78642691	Pkp2	1
GO:0045121	membrane raft	3/140	299/14612	0.01003344	1.04720497	0.08111435	0.54889388	0.82334082	0.78642691	Card11/Has2/Cr1l	3
GO:0098857	membrane microdomain	3/140	301/14612	0.00996678	1.0402468	0.06939358	0.55342691	0.82555395	0.78854081	Card11/Has2/Cr1l	3
GO:0043235	receptor complex	3/140	308/14612	0.00974026	1.01660482	0.02896814	0.56908071	0.83034853	0.79312042	Bmal1/Hspd1/Cr1l	3
GO:0000313	organellar ribosome	1/140	88/14612	0.01136364	1.18603896	0.1721638	0.57247918	0.83034853	0.79312042	Mrps6	1
GO:0005761	mitochondrial ribosome	1/140	88/14612	0.01136364	1.18603896	0.1721638	0.57247918	0.83034853	0.79312042	Mrps6	1
GO:0071013	catalytic step 2 spliceosome	1/140	89/14612	0.01123596	1.17271268	0.16074252	0.57660015	0.83034853	0.79312042	Hnrnpc	1
GO:0000792	heterochromatin	1/140	90/14612	0.01111111	1.15968254	0.14945319	0.58068167	0.83034853	0.79312042	Cbx1	1
GO:0031514	motile cilium	2/140	204/14612	0.00980392	1.0232493	0.03288992	0.58467467	0.83034853	0.79312042	Hspd1/Ift172	2
GO:0044306	neuron projection terminus	2/140	204/14612	0.00980392	1.0232493	0.03288992	0.58467467	0.83034853	0.79312042	Slc17a8/Sri	2
GO:0055029	nuclear DNA-directed RNA polymerase complex	1/140	91/14612	0.01098901	1.14693878	0.13829248	0.58472413	0.83034853	0.79312042	Tbpl1	1
GO:0055038	recycling endosome membrane	1/140	92/14612	0.01086957	1.13447205	0.12725719	0.58872789	0.83034853	0.79312042	Snx18	1
GO:0097731	9+0 non-motile cilium	1/140	92/14612	0.01086957	1.13447205	0.12725719	0.58872789	0.83034853	0.79312042	Ift80	1
GO:0016324	apical plasma membrane	3/140	319/14612	0.00940439	0.98154948	-0.0327704	0.59299549	0.83034853	0.79312042	Slc5a3/Pdia3/Akr1a1	3
GO:0000428	DNA-directed RNA polymerase complex	1/140	94/14612	0.0106383	1.11033435	0.10555066	0.59662079	0.83034853	0.79312042	Tbpl1	1
GO:0005881	cytoplasmic microtubule	1/140	94/14612	0.0106383	1.11033435	0.10555066	0.59662079	0.83034853	0.79312042	Srprb	1
GO:0030880	RNA polymerase complex	1/140	95/14612	0.01052632	1.09864662	0.09487358	0.60051066	0.8314763	0.79419763	Tbpl1	1
GO:0060076	excitatory synapse	1/140	99/14612	0.01010101	1.05425685	0.05327621	0.6157012	0.84178744	0.80404648	Slc17a8	1
GO:0031985	Golgi cisterna	1/140	101/14612	0.00990099	1.03338048	0.03310887	0.62307998	0.84178744	0.80404648	Hspd1	1
GO:0005774	vacuolar membrane	3/140	334/14612	0.00898204	0.93746792	-0.1137057	0.62421167	0.84178744	0.80404648	Snx14/Rmc1/Atp6v0b	3
GO:0005903	brush border	1/140	102/14612	0.00980392	1.0232493	0.0231748	0.62671645	0.84178744	0.80404648	Scin	1
GO:0030017	sarcomere	2/140	221/14612	0.00904977	0.94453782	-0.0817123	0.62837979	0.84178744	0.80404648	Kcnn1/Sri	2
GO:0000775	chromosome, centromeric region	2/140	225/14612	0.00888889	0.92774603	-0.1074255	0.63813426	0.84178744	0.80404648	Ppp2cb/Cbx1	2
GO:0043197	dendritic spine	2/140	227/14612	0.00881057	0.91957206	-0.1201171	0.64293636	0.84178744	0.80404648	Zfp804a/Sri	2
GO:0005667	transcription regulator complex	4/140	458/14612	0.00873362	0.91154086	-0.1891801	0.64351737	0.84178744	0.80404648	Bmal1/Smad2/Tbpl1/Taf1a	4
GO:0016605	PML body	1/140	107/14612	0.00934579	0.97543391	-0.0250847	0.64438284	0.84178744	0.80404648	Bmal1	1
GO:0098791	Golgi apparatus subcompartment	3/140	347/14612	0.00864553	0.90234664	-0.1810738	0.64992793	0.84178744	0.80404648	Rab38/Hspd1/Lap3	3
GO:0098797	plasma membrane protein complex	4/140	463/14612	0.00863931	0.90169701	-0.2114142	0.65197349	0.84178744	0.80404648	Slc5a3/Fzd8/Kcnip2/Cdh10	4
GO:0030658	transport vesicle membrane	2/140	231/14612	0.00865801	0.90364873	-0.1451806	0.65239097	0.84178744	0.80404648	Slc17a8/Sec22b	2
GO:0000781	chromosome, telomeric region	1/140	110/14612	0.00909091	0.94883117	-0.0529819	0.65458155	0.84178744	0.80404648	Cbx1	1
GO:0030426	growth cone	2/140	232/14612	0.00862069	0.89975369	-0.1513814	0.65472356	0.84178744	0.80404648	Zfp804a/Snx18	2
GO:0044309	neuron spine	2/140	232/14612	0.00862069	0.89975369	-0.1513814	0.65472356	0.84178744	0.80404648	Zfp804a/Sri	2
GO:0005911	cell-cell junction	4/140	467/14612	0.00856531	0.89397369	-0.2290395	0.65864307	0.84281341	0.80502645	Pkp3/Pkp2/Cdh10/Cd99l2	4
GO:0019897	extrinsic component of plasma membrane	1/140	113/14612	0.00884956	0.92364096	-0.0801439	0.66448981	0.84554761	0.80763807	Cdh10	1
GO:0030427	site of polarized growth	2/140	238/14612	0.00840336	0.87707083	-0.1880591	0.66845981	0.84554761	0.80763807	Zfp804a/Snx18	2
GO:0005840	ribosome	2/140	240/14612	0.00833333	0.8697619	-0.2000897	0.67294034	0.84554761	0.80763807	Calr/Mrps6	2
GO:0030016	myofibril	2/140	241/14612	0.00829876	0.86615293	-0.2060694	0.6751623	0.84554761	0.80763807	Kcnn1/Sri	2
GO:0097730	non-motile cilium	1/140	117/14612	0.00854701	0.89206349	-0.1152901	0.67726316	0.84554761	0.80763807	Ift80	1
GO:0005802	trans-Golgi network	2/140	243/14612	0.00823045	0.8590241	-0.2179588	0.67956975	0.84554761	0.80763807	Rab38/Lap3	2
GO:0016363	nuclear matrix	1/140	121/14612	0.00826446	0.86257379	-0.1492976	0.68955355	0.84973438	0.81163713	Srpk1	1
GO:0043292	contractile muscle fiber	2/140	249/14612	0.00803213	0.83832473	-0.2530815	0.69250232	0.84973438	0.81163713	Kcnn1/Sri	2
GO:0090734	site of DNA damage	1/140	122/14612	0.00819672	0.85550351	-0.1576314	0.69255282	0.84973438	0.81163713	Cbx1	1
GO:1904949	ATPase complex	1/140	123/14612	0.00813008	0.8485482	-0.1659004	0.69552333	0.84973438	0.81163713	Atp6v0b	1
GO:1990204	oxidoreductase complex	1/140	130/14612	0.00769231	0.80285714	-0.222064	0.71553395	0.87023604	0.83121961	P4hb	1
GO:0005795	Golgi stack	1/140	134/14612	0.00746269	0.77889126	-0.252897	0.72637651	0.87023604	0.83121961	Hspd1	1
GO:0005930	axoneme	1/140	134/14612	0.00746269	0.77889126	-0.252897	0.72637651	0.87023604	0.83121961	Ift172	1
GO:0097014	ciliary plasm	1/140	135/14612	0.00740741	0.77312169	-0.2604715	0.7290224	0.87023604	0.83121961	Ift172	1
GO:0045177	apical part of cell	3/140	393/14612	0.00763359	0.79672846	-0.4017705	0.73079982	0.87023604	0.83121961	Slc5a3/Pdia3/Akr1a1	3
GO:0048786	presynaptic active zone	1/140	136/14612	0.00735294	0.76743697	-0.2679943	0.73164289	0.87023604	0.83121961	Cdh10	1
GO:0030312	external encapsulating structure	3/140	398/14612	0.00753769	0.78671931	-0.4243016	0.73864879	0.87089595	0.83184993	Calr/Mmp14/Lrrn1	3
GO:0031012	extracellular matrix	3/140	398/14612	0.00753769	0.78671931	-0.4243016	0.73864879	0.87089595	0.83184993	Calr/Mmp14/Lrrn1	3
GO:0008021	synaptic vesicle	2/140	277/14612	0.00722022	0.75358432	-0.4072387	0.74729516	0.87592173	0.83665038	Slc17a8/Sec22b	2
GO:0005765	lysosomal membrane	2/140	280/14612	0.00714286	0.7455102	-0.4228981	0.75264385	0.87592173	0.83665038	Snx14/Rmc1	2
GO:0098852	lytic vacuole membrane	2/140	280/14612	0.00714286	0.7455102	-0.4228981	0.75264385	0.87592173	0.83665038	Snx14/Rmc1	2
GO:0009898	cytoplasmic side of plasma membrane	1/140	147/14612	0.00680272	0.71000972	-0.3475543	0.75885927	0.8782998	0.83892183	Snx18	1
GO:0034399	nuclear periphery	1/140	148/14612	0.00675676	0.70521236	-0.3545158	0.76119316	0.8782998	0.83892183	Srpk1	1
GO:0005874	microtubule	3/140	416/14612	0.00721154	0.75267857	-0.5033443	0.76542477	0.8794242	0.83999582	Zfp804a/Tppp3/Srprb	3
GO:0070382	exocytic vesicle	2/140	293/14612	0.00682594	0.71243296	-0.4890534	0.7747159	0.88509214	0.84540965	Slc17a8/Sec22b	2
GO:0098862	cluster of actin-based cell projections	1/140	155/14612	0.00645161	0.67336406	-0.4020966	0.77691422	0.88509214	0.84540965	Scin	1
GO:0098982	GABA-ergic synapse	1/140	158/14612	0.00632911	0.66057866	-0.4219037	0.78333311	0.88865521	0.84881297	Cdh10	1
GO:0030672	synaptic vesicle membrane	1/140	164/14612	0.00609756	0.63641115	-0.460541	0.79562587	0.89507911	0.85494886	Slc17a8	1
GO:0099501	exocytic vesicle membrane	1/140	164/14612	0.00609756	0.63641115	-0.460541	0.79562587	0.89507911	0.85494886	Slc17a8	1
GO:0000922	spindle pole	1/140	167/14612	0.00598802	0.62497861	-0.4793969	0.80151	0.89795726	0.85769797	Ppp2cb	1
GO:0098798	mitochondrial protein-containing complex	1/140	172/14612	0.00581395	0.60681063	-0.5101784	0.81094539	0.90352714	0.86301812	Mrps6	1
GO:0019898	extrinsic component of membrane	1/140	174/14612	0.00574713	0.5998358	-0.5222748	0.81459363	0.90352714	0.86301812	Cdh10	1
GO:0098687	chromosomal region	2/140	321/14612	0.00623053	0.65028927	-0.6231174	0.81652082	0.90352714	0.86301812	Ppp2cb/Cbx1	2
GO:0099634	postsynaptic specialization membrane	1/140	180/14612	0.00555556	0.57984127	-0.5578611	0.82512418	0.90640478	0.86576675	Cdh10	1
GO:0005938	cell cortex	2/140	328/14612	0.00609756	0.63641115	-0.6550316	0.82583547	0.90640478	0.86576675	Itch/Ric8b	2
GO:0005741	mitochondrial outer membrane	1/140	187/14612	0.00534759	0.55813598	-0.598124	0.83666134	0.9108813	0.87004256	S2bpcox16	1
GO:0055037	recycling endosome	1/140	187/14612	0.00534759	0.55813598	-0.598124	0.83666134	0.9108813	0.87004256	Snx18	1
GO:0098562	cytoplasmic side of membrane	1/140	190/14612	0.00526316	0.54932331	-0.6149911	0.84137144	0.91233048	0.87142678	Snx18	1
GO:0044391	ribosomal subunit	1/140	193/14612	0.00518135	0.5407846	-0.6316365	0.84594667	0.9136224	0.87266077	Mrps6	1
GO:0043204	perikaryon	1/140	196/14612	0.00510204	0.53250729	-0.6480674	0.85039085	0.91476306	0.87375029	Slc17a8	1
GO:0015629	actin cytoskeleton	3/140	495/14612	0.00606061	0.63255411	-0.8180211	0.85780634	0.91907823	0.87787199	Scin/Hnrnpc/Sh3pxd2a	3
GO:0000228	nuclear chromosome	1/140	204/14612	0.00490196	0.51162465	-0.6908865	0.86162934	0.91952538	0.8782991	Brms1	1
GO:0030027	lamellipodium	1/140	212/14612	0.00471698	0.49231806	-0.732349	0.87202918	0.92233001	0.88097799	P4hb	1
GO:0019867	outer membrane	1/140	214/14612	0.0046729	0.48771696	-0.7425155	0.87450549	0.92233001	0.88097799	S2bpcox16	1
GO:0031968	organelle outer membrane	1/140	214/14612	0.0046729	0.48771696	-0.7425155	0.87450549	0.92233001	0.88097799	S2bpcox16	1
GO:0036464	cytoplasmic ribonucleoprotein granule	1/140	229/14612	0.00436681	0.45577043	-0.8164223	0.89162223	0.93286981	0.89104523	Bmal1	1
GO:0032838	plasma membrane bounded cell projection cytoplasm	1/140	232/14612	0.00431034	0.44987685	-0.8307382	0.8947565	0.93286981	0.89104523	Ift172	1
GO:0005819	spindle	2/140	394/14612	0.00507614	0.52980421	-0.9305663	0.894864	0.93286981	0.89104523	Ppp2cb/Cbx1	2
GO:0030133	transport vesicle	2/140	411/14612	0.00486618	0.50789016	-0.9953211	0.90800783	0.9360265	0.8940604	Slc17a8/Sec22b	2
GO:0035770	ribonucleoprotein granule	1/140	248/14612	0.00403226	0.42085253	-0.9047259	0.91001351	0.9360265	0.8940604	Bmal1	1
GO:0042734	presynaptic membrane	1/140	250/14612	0.004	0.41748571	-0.913711	0.91175915	0.9360265	0.8940604	Cdh10	1
GO:0061695	transferase complex, transferring phosphorus-containing groups	1/140	250/14612	0.004	0.41748571	-0.913711	0.91175915	0.9360265	0.8940604	Tbpl1	1
GO:0099568	cytoplasmic region	1/140	270/14612	0.0037037	0.38656085	-1.0006639	0.92746826	0.94854708	0.90601963	Ift172	1
GO:0031965	nuclear membrane	1/140	283/14612	0.00353357	0.36880363	-1.0546042	0.9361557	0.95090929	0.90827593	Phf11b	1
GO:0005635	nuclear envelope	2/140	458/14612	0.00436681	0.45577043	-1.1638982	0.93682174	0.95090929	0.90827593	Phf11b/Calr	2
GO:0062023	collagen-containing extracellular matrix	1/140	306/14612	0.00326797	0.3410831	-1.1457028	0.94907017	0.95973388	0.91670488	Calr	1
GO:0045211	postsynaptic membrane	1/140	365/14612	0.00273973	0.28594912	-1.3587939	0.97152463	0.97877481	0.93489212	Cdh10	1
GO:0031252	cell leading edge	1/140	454/14612	0.00220264	0.22989301	-1.6395239	0.98820819	0.99150829	0.9470547	P4hb	1
GO:0099572	postsynaptic specialization	1/140	487/14612	0.00205339	0.21431505	-1.7344365	0.99150829	0.99150829	0.9470547	Cdh10	1
